# Understanding divergent substrate stereoselectivity in the isothiourea-catalysed conjugate addition of cyclic α-substituted β-ketoesters to α,β-unsaturated aryl esters†

**DOI:** 10.1039/d3sc05470e

**Published:** 2023-11-21

**Authors:** Ding Yuan, Alister S. Goodfellow, Kevin Kasten, Zhuan Duan, Tengfei Kang, David B. Cordes, Aidan P. McKay, Michael Bühl, Gregory R. Boyce, Andrew D. Smith

**Affiliations:** a EaStCHEM, School of Chemistry, University of St Andrews St Andrews Fife KY16 9ST UK ads10@st-andrews.ac.uk buehl@st-andrews.ac.uk; b School of Biological and Chemical Engineering, Panzhihua University Panzhihua 617000 China; c Department of Chemistry and Physics, Florida Gulf Coast University Fort Myers Florida 33965 USA; d Department of Chemistry and Biochemistry, East Stroudsburg University East Stroudsburg Pennsylvania 18301 USA gboyce@esu.edu

## Abstract

The development of enantioselective synthetic methods capable of generating vicinal stereogenic centres, where one is tetrasubstituted (such as either an all-carbon quaternary centre or where one or more substituents are heteroatoms), is a recognised synthetic challenge. Herein, the enantioselective conjugate addition of a range of carbo- and heterocyclic α-substituted β-ketoesters to α,β-unsaturated aryl esters using the isothiourea HyperBTM as a Lewis base catalyst is demonstrated. Notably, divergent diastereoselectivity is observed through the use of either cyclopentanone-derived or indanone-derived substituted β-ketoesters with both generating the desired stereodefined products with high selectivity (>95 : 5 dr, up to 99 : 1 er). The scope and limitations of these processes are demonstrated, alongside application on gram scale. The origin of the divergent substrate selectivity has been probed through the use of DFT-analysis, with preferential orientation driven by dual stabilising CH⋯O interactions. The importance of solvation with strongly polar transition-states is highlighted and the SMD solvation model is demonstrated to capture solvation effects reliably.

## Abstract 2

Divergent stereoselectivity is rationalised in isothiourea catalysed conjugate additions of β-keto esters.

## Graphical abstract

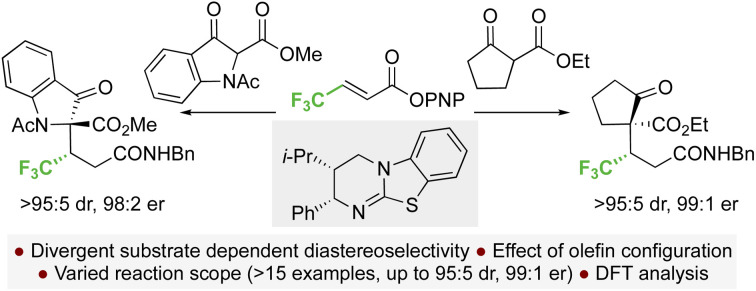

## 1 Introduction

**Scheme 1:**
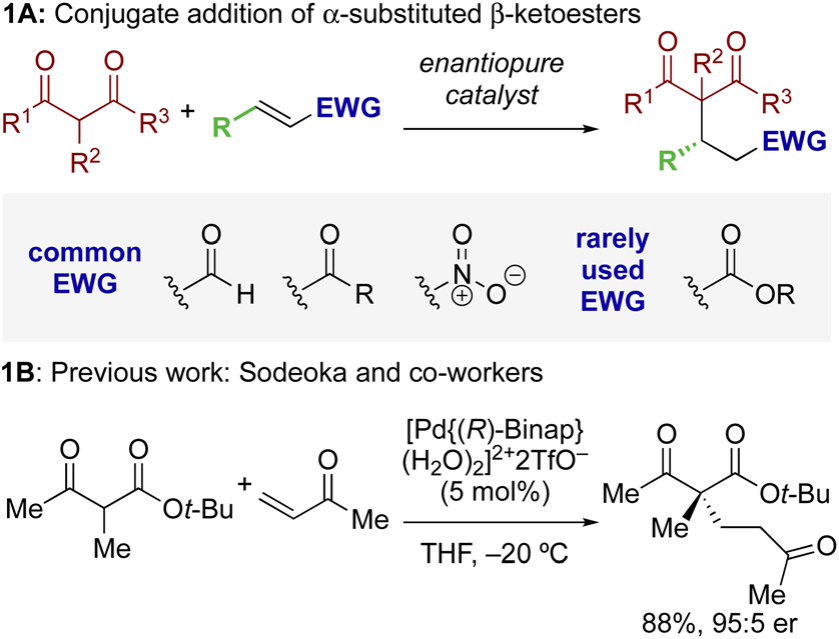
Context: enantioselective conjugate addition of α-substituted β-ketoesters.

The ability to selectively control the relative and absolute configuration in compounds containing vicinal stereogenic centres, where one is tetrasubstituted (defined herein as a stereogenic carbon that does not have a proton as a substituent) is of significant interest. Despite many advances in the development of synthetic methods in this area,^1,2^ this still remains a meaningful challenge due to the generally low reactivity of suitable precursors caused by steric encumbrance. Among methods developed in this area, the enantioselective conjugate addition of α-substituted β-ketoesters has been widely explored based on the ability to generate up to two contiguous stereocentres, one of them quaternary (Scheme 1A).^3,4^ As representative examples, this strategy has been applied to the stereoselective addition to nitro-olefins,^5–7^ di-*t*-butyl azodicarboxylate,^8,9^ vinyl ketones,^10^ and propargyl alcohols.^11^ For example, Sodeoka *et al.* showed that enantioselective addition of various α-substituted β-ketoesters to enones proceeded in good yields with excellent enantioselectivity using Pd-BINAP derived enolates (Scheme 1B).^12^ Alternatively, the Pd-catalysed allylic alkylation of Morita–Baylis–Hillman adducts has also been developed with β-ketocarbonyls.^13^ Despite these precedents, stereoselective conjugate additions of α-substituted β-ketoesters to α,β-unsaturated esters is rare due to the low inherent electrophilicity of these Michael acceptors.

**Scheme 2:**
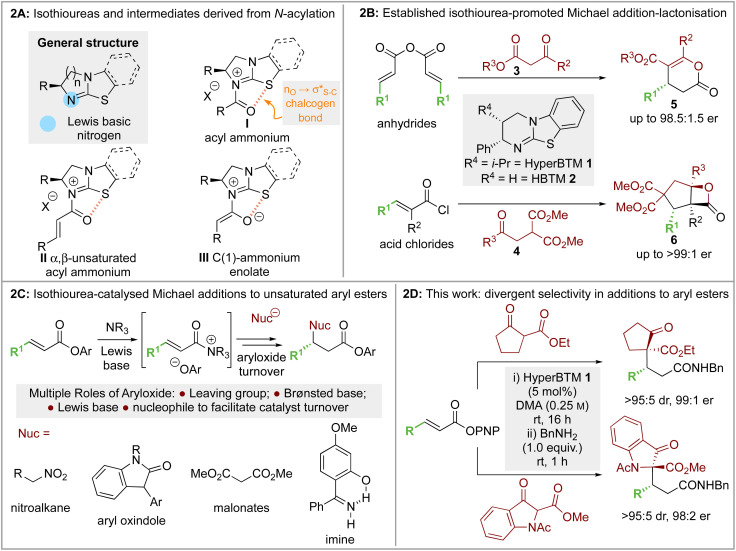
(A) Isothioureas and common intermediates derived from *N*-acylation; (B) established isothiourea-promoted Michael addition-lactonisation; (C) isothiourea-catalysed addition to α,β-unsaturated aryl esters; (D) this work: divergent substrate dependent selectivity in addition to α,β-unsaturated aryl esters.

The use of isothioureas as Lewis basic organocatalysts has expanded remarkably over the last fifteen years. Building upon the pioneering work of Birman who demonstrated their first use as acyl transfer catalysts for kinetic resolution processes, these readily prepared chiral Lewis bases have since been harnessed for a plethora of transformations.^14^ This broad range of reactivity incorporates the generation and reactivity of acyl ammonium I,^14^ α,β-unsaturated acyl ammonium II ^15^ and C(1)-ammonium enolate III ^16^ intermediates that exploit a key 1,5 S⋯O chalcogen bonding interaction (*n*_O_ to 
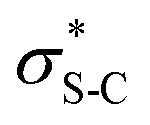
) to achieve stereocontrol (Scheme 2A). Of relevance to this work, the generation and exploitation of α,β-unsaturated acyl ammonium intermediates was initially achieved from acid chloride and anhydride starting materials. For example, isothioureas (2*S*,3*R*)-HyperBTM 1 and (*S*)-HBTM 2 have been employed in cascade processes with β-ketoesters 3 ^17^ and γ-ketomalonates 4,^18^ that involve initial Michael addition followed by *intramolecular* cyclisation to promote catalyst turnover, generating δ- and β-lactones 5 and 6 (Scheme 2B). Related conjugate addition/cyclisation processes with acyl benzazoles,^17,19^ aminothiophenols,^20^ heterocyclic nucleophiles,^21^ as well as Rh-metallacycles has been reported.^22^ Joint synthetic and computational analysis of the factors leading to stereocontrol and chemoselectivity in acyl benzazole addition has led to enhanced understanding of these processes.^23^

In recent work, the use of α,β-unsaturated electron-deficient aryl esters has been shown to offer a potential (albeit limited) solution to the recognised recalcitrance of α,β-unsaturated esters to enantioselective conjugate addition (Scheme 2C). Acylation of these species with an isothiourea generates an α,β-unsaturated acyl ammonium ion pair, with the enhanced electrophilicity of this intermediate species (compared to the parent ester) allowing for effective catalysis. Furthermore, the electron-deficient phenoxides generated *in situ* can be exploited to promote catalytic turnover, while their multifunctional nature can be exploited through acting as both Brønsted base and Lewis base.^24^ Initially demonstrated in the enantioselective conjugate addition of nitroalkanes,^19^ it has since been exploited using heterocyclic pro-nucleophiles,^21,25^ malonates,^26^ benzophenone imines,^27^ as well as co-operative Pd-isothiourea promoted cascade reactions.^28^ Conjugate addition and lactonisation processes using acylbenzothiazole and benzoxazole pronucleophile derivatives with α,β-unsaturated *para*-nitrophenyl esters has also been investigated synthetically.^29^ This study was complemented by a computational study by Wei and Ding who validated the postulated mechanism and outlined the key factors involved in stereoselective C–C bond-formation.^30^

Building upon these previous studies, in this manuscript the enantioselective conjugate addition of a range of carbo- and heterocyclic α-substituted β-ketoesters to α,β-unsaturated aryl esters using the isothiourea HyperBTM 1 as a Lewis base catalyst is reported. Interestingly, divergent diastereoselectivity is observed with the use of either cyclopentanone-derived or indanone-derived substituted β-ketoesters, with both generating the desired stereodefined products with high selectivity (>95 : 5 dr, up to 99 : 1 er, Scheme 2D). The origin of the divergent substrate selectivity has been probed using DFT-analysis at the M06-2X_SMD_/def2-TZVP//M06-2X_SMD_/def2-SVP level.

## 2 Results and discussion

### 2.1 Optimisation: conjugate addition of ethyl 2-oxocyclopentane-1-carboxylate to *p*-nitrophenyl 4,4,4-trifluorobut-2-enoate

Initial investigations began with developing the stereoselective reaction of ethyl 2-oxocyclopentane-1-carboxylate 7 (1.5 equiv.) with β-trifluoromethyl-α,β-unsaturated *p*-nitrophenyl (PNP) ester 8 using (2*S*,3*R*)-HyperBTM 1 (20 mol%) as the Lewis base catalyst. A benzylamine quench was performed after the catalytic transformation to aid product isolation. No reaction was observed in MeCN, CH_2_Cl_2_ or EtOAc (entries 1–3). However, the use of amide solvents gave promising results, with NMP, DMF, and DMA giving between 40-70% conversion to the desired product (entries 4–6). Using DMA, further optimisation showed that reducing the equivalents of β-ketoester 7 and the catalyst loading to 5 mol% led to good reaction conversion (entries 7–10), giving the desired product 9 in 57% isolated yield (>95 : 5 dr, 99 : 1 er). Reaction conditions implementing a catalyst loading of 5 mol% was chosen as optimal to showcase the efficiency of this process at low catalyst loading (Table 1).

**Table 1:** Initial optimisation^a^

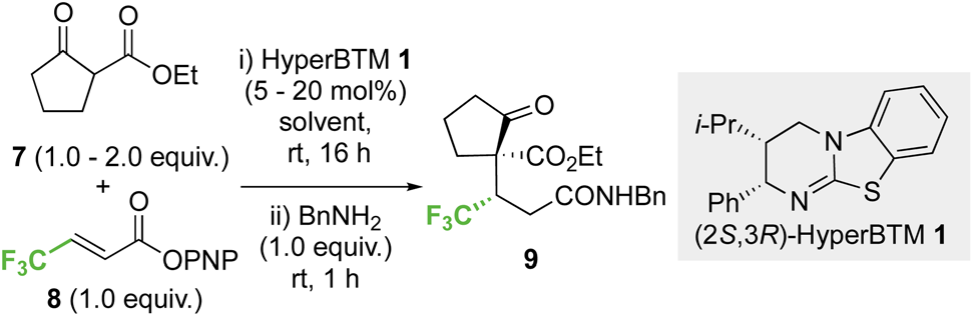				
Entry	Solvent	HyperBTM 1 (mol%)	7 (equiv.)	Conversion^b^
1	MeCN	20	1.5	0
2	CH_2_Cl_2_	20	1.5	0
3	EtOAc	20	1.5	0
4	NMP	20	1.5	41
5	DMF	20	1.5	60
6	DMA	20	1.5	70
7	DMA	20	2.0	70
8	DMA	20	1.0	66
9	DMA	10	1.0	66
10	DMA	5	1.0	61^c^

a Reactions performed on 0.5 mmol scale of 8.

b Formation of product determined by ^19^F NMR analysis of the crude reaction mixture by relative abundance of all fluorine signals in the sample.

c Isolated in 57% yield, >95 : 5 dr, 99 : 1 er; dr determined by ^1^H NMR analysis of the crude reaction mixture, er determined by HPLC analysis on a chiral stationary phase. NMP = *N*-methylpyrrolidine, DMF = *N*,*N*-dimethylformamide, DMA = *N*,*N*-dimethylacetamide.

### 2.2 Scope and limitations

#### 2.2.1 Conjugate additions of ethyl 2-oxocyclopentane-1-carboxylate to α,β-unsaturated *p*-nitrophenyl esters

**Scheme 3:**
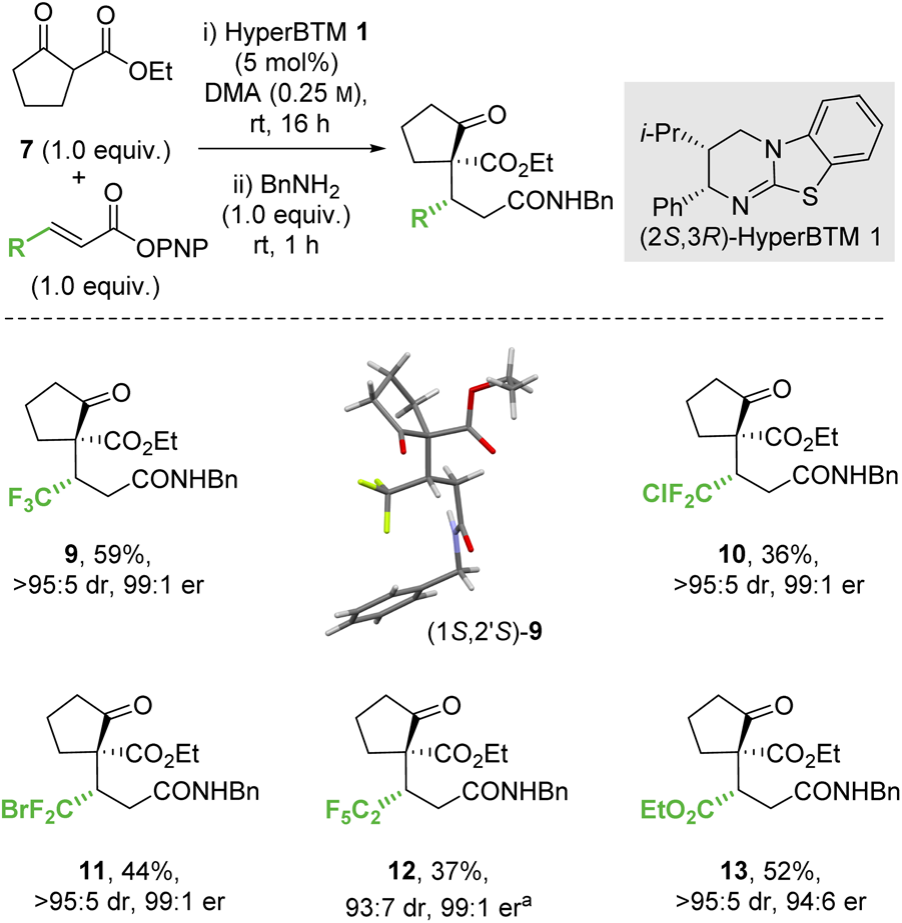
Conjugate addition of ethyl 2-oxocyclopentane-1-carboxylate. All product ers were determined by HPLC analysis on a chiral stationary phase. All product drs were determined by ^1^H NMR analysis of the crude reaction mixture. Following reaction conditions were applied unless stated otherwise: 0.2 mmol 7, 0.2 mmol *p*-nitrophenol ester, 0.01 mmol 1 were stirred in 0.8 mL DMA at rt for 16 h. Subsequently, 0.2 mmol BnNH_2_ was added and stirred at rt for 1 h. (a) Combined yield of inseparable diastereomers.

Further work considered the scope and limitations of this process initially through changing the α,β-unsaturated *para*-nitrophenyl ester component of the reaction (Scheme 3). Under the developed conditions the incorporation of an electron withdrawing β-substituent was necessary to promote reactivity, with β-perhalogenated substrates giving the corresponding CF_2_Cl, CF_2_Br and C_2_F_5_ variants 10–12 respectively in synthetically useful yield and excellent enantioselectivity (>95 : 5 dr, 99 : 1 er). Similarly, a β-ester substituent was also tolerated, giving 13 in 52% yield, >95 : 5 dr and 94 : 6 er. The relative and absolute configuration of (1*S*,2′*S*)-9 was confirmed by single crystal X-ray crystallography,^31^ with the configuration within all other products 10–13 assigned by analogy.

#### 2.2.2 Conjugate addition of α-substituted carbo- and heterocyclic β-ketoesters to *p*-nitrophenyl 4,4,4-trifluorobut-2-enoate

**Scheme 4:**
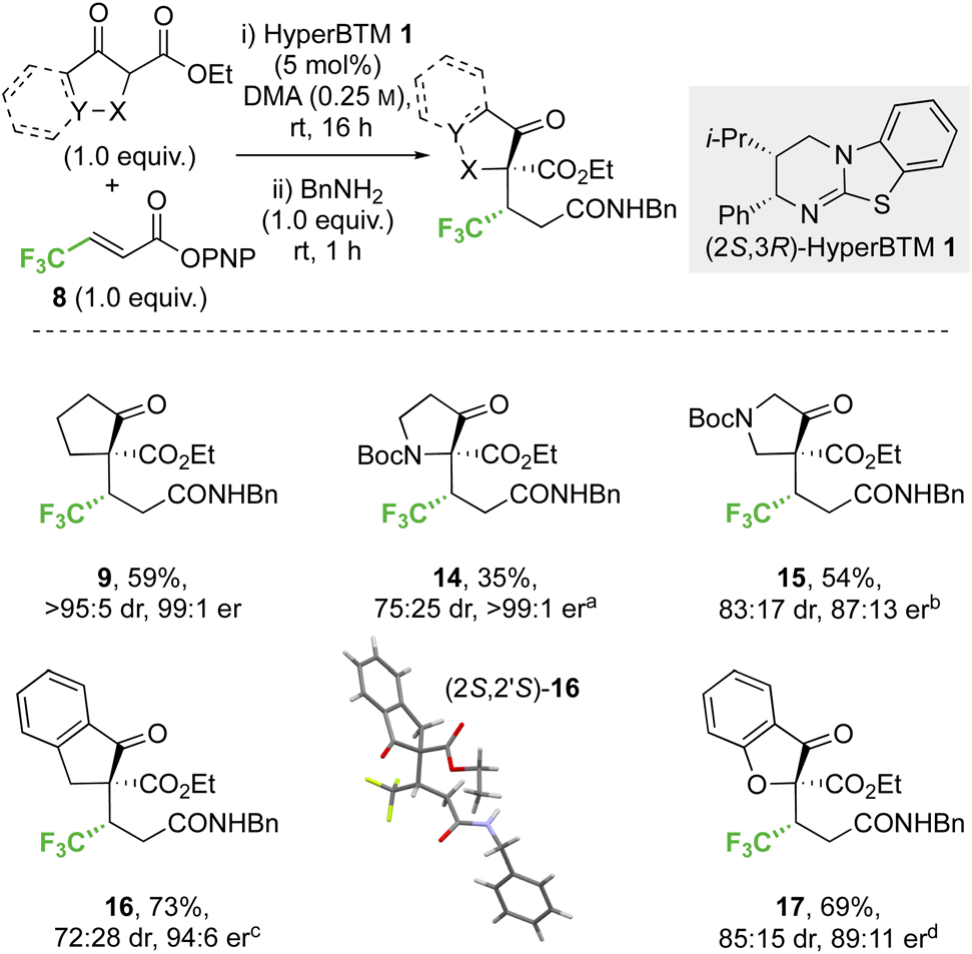
Conjugate addition of carbo- and heterocyclic α-substituted β-ketoesters to α,β-unsaturated *p*-nitrophenyl esters. All product ers were determined by HPLC analysis on a chiral stationary phase. All product drs were determined by ^1^H NMR analysis of the crude reaction mixture. Following reaction conditions were applied unless stated otherwise: 0.2 mmol ketoester, 0.2 mmol 8, 0.01 mmol 1 were stirred in 0.8 mL DMA at rt for 16 h. Subsequently, 0.2 mmol BnNH_2_ was added and stirred at rt for 1 h. (a) 59 : 41 er of minor diastereomer, combined yield of inseparable diastereomers. (b) 97 : 3 er of minor diastereomer, combined yield of inseparable diastereomers. (c) 96 : 4 er of minor diastereomer, diastereomers isolated yields (major/minor): 51%/22%. (d) 95 : 5 er of minor diastereomer, combined yield of inseparable diastereomers.

The effect of variation within the α-substituted β-ketoester reaction component in the addition to β-trifluoromethyl-α,β-unsaturated PNP ester 8 (Scheme 4) was then examined. The incorporation of 3-oxopyrrolidine and 4-oxopyrrolidine scaffolds were successful, generating 14 and 15 respectively. Reduced yield (35%) and diastereoselectivity (75 : 25 dr) but high enantioselectivity (>99 : 1 er) was observed with the 3-oxopyrrolidine scaffold, whereas improved yield and high stereocontrol (54%, 83 : 17 dr, 87 : 13 er) were obtained in the 4-oxopyrrolidine case. The effect of benzannulation was next explored, with the dihydro-1*H*-indene derivative giving 16 (73%, 72 : 28 dr, 94 : 6 er). The relative and absolute configuration within the major diastereoisomer (2*S*,2′*S*)-16 was confirmed by single crystal X-ray crystallography and correlates with that observed within product 9,^32^ with the configuration within all other products 14, 15 and 17 assigned by analogy. The dihydrobenzofuran derivative was also successfully employed, giving heterocyclic product 17 (69%, 85 : 15 dr, 89 : 11 er).

#### 2.2.3 Conjugate additions of methyl 1-acetyl-3-oxoindolinone-2-carboxylate to α,β-unsaturated *p*-nitrophenyl esters

**Scheme 5:**
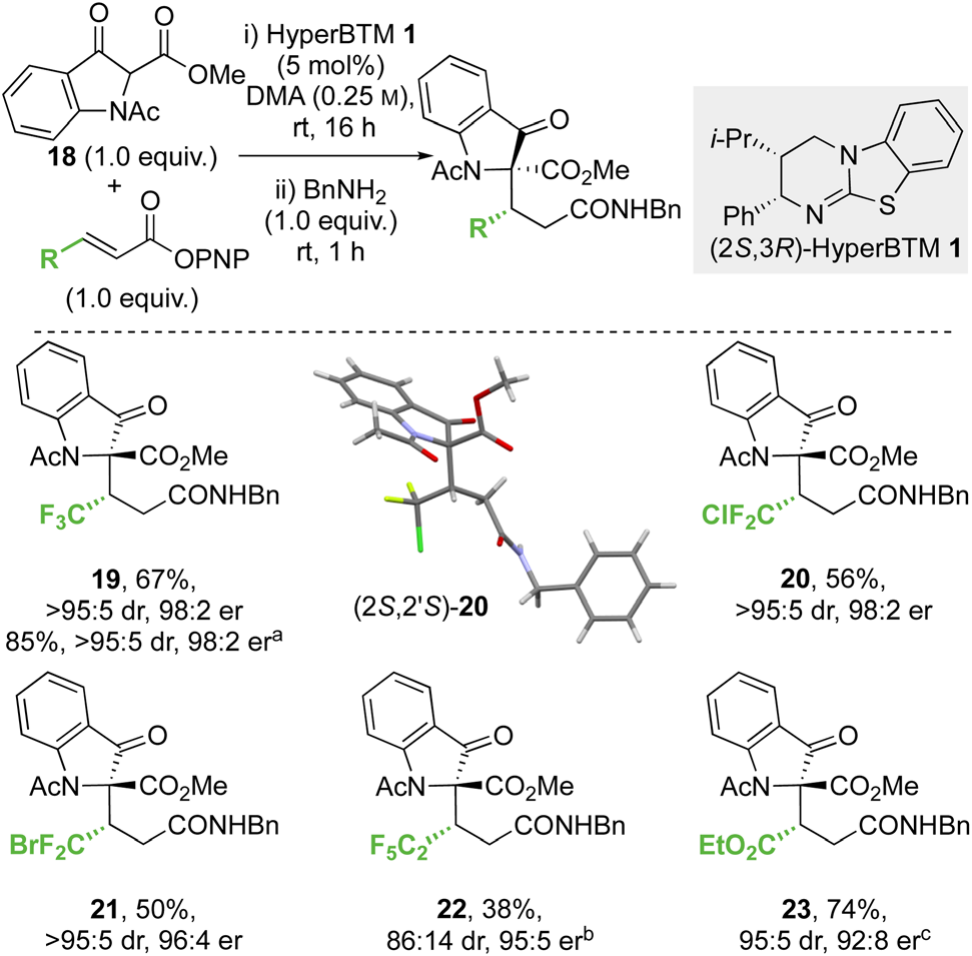
Conjugate addition of methyl 1-acetyl-3-oxoindolinone-2-carboxylate to α,β-unsaturated *p*-nitrophenyl esters. All product ers were determined by HPLC analysis on a chiral stationary phase. All product drs determined by ^1^H NMR analysis of the crude reaction mixture. Following reaction conditions were applied unless stated otherwise: 0.2 mmol 18, 0.2 mmol *p*-nitrophenol ester, 0.01 mmol 1 were stirred in 0.8 mL DMA at rt for 16 h. Subsequently, 0.2 mmol BnNH_2_ was added and stirred at rt for 1 h. (a) Reaction on a gram scale with purification by crystallisation. (b) 76 : 24 er of minor diastereomer, combined yield of inseparable diastereomers. (c) 72 : 18 er of minor diastereomer, combined yield of inseparable diastereomers.

As both benzannulation and α-heteroatom substitution within the β-ketoester reaction component had been tolerated their dual incorporation within an alternative scaffold, methyl 1-acetyl-3-oxoindolinone-2-carboxylate 18, and its conjugate addition was assessed (Scheme 5). The conjugate addition of 18 to the β-trifluoromethyl-α,β-unsaturated PNP ester gave 19 in 67% yield (>95 : 5 dr, 98 : 2 er) on an analytical scale. A scaled-up synthesis on a 4.4 mmol scale allowed the formation of 19 in >1 g and 85% yield (>95 : 5 dr, 98 : 2 er) after direct recrystallisation from the crude reaction mixture. Application to alternative β-perhalogenated PNP esters gave the corresponding CF_2_Cl, CF_2_Br and C_2_F_5_ variants 20–22 respectively in acceptable yield (38% to 56%) and excellent stereoselectivity (>95 : 5 dr, 95 : 5 to 98 : 2 er). Further application of this methodology gave the β-ester variant 23 in 74% yield, 95 : 5 dr and 92 : 8 er. The relative and absolute configuration of (2*S*,2′*S*)-20 was confirmed by single crystal X-ray crystallography,^33^ with the configuration within all other products 19 and 21–23 derived from 18 assigned by analogy. Notably, in this system the relative configuration within the products is *opposite* to that previously observed at the stereogenic centre derived from the β-ketoester (section 2.2.1 and 2.2.2, note: CIP priority changes) consistent with divergent selectivity being observed for this reaction component.

#### 2.2.4 Conjugate additions to (*Z*)-β-trifluoro- and (*Z*)-β-ethoxycarbonyl α,β-unsaturated *p*-nitrophenyl esters

**Scheme 6:**
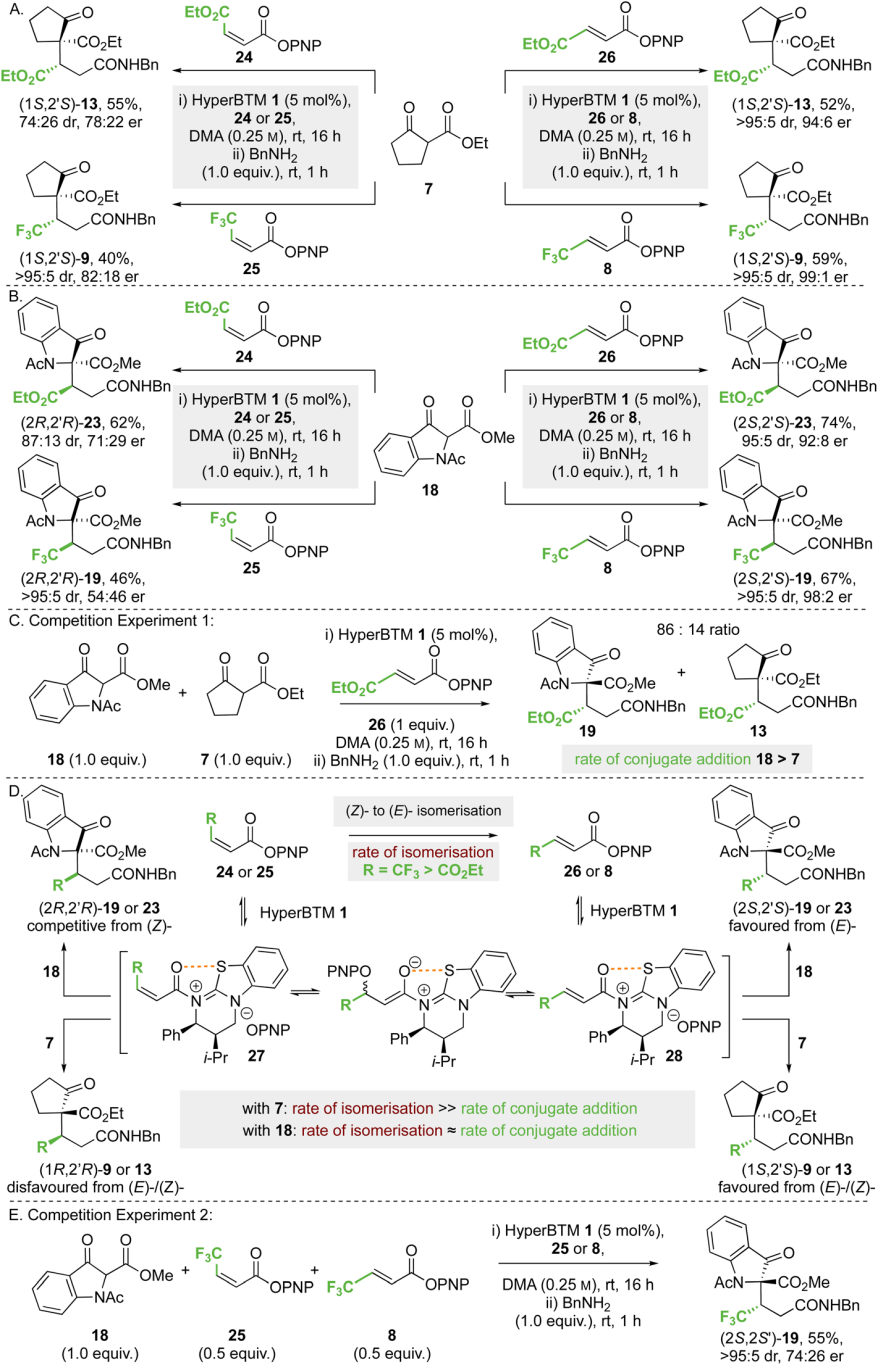
Effect of (*Z*)- or (*E*)-enoate configuration on stereoselectivity. All ers determined by HPLC analysis on a chiral stationary phase. All product drs determined by ^1^H NMR analysis of the crude reaction mixture. Yields are isolated yields after purification.

The effect of olefin configuration on product yield and stereoselectivity was next investigated using maleate 24 and fumarate 26, as well as CF_3_-substituted α,β-unsaturated PNP ester derivatives (*Z*)-25 and (*E*)-8 (Scheme 6). Using ethyl 2-oxocyclopentane-1-carboxylate 7 as the nucleophile (Scheme 5A), reaction with maleate 24 gave product (1*S*,2′*S*)-13 in the same enantiomeric series as fumarate 26, but with reduced stereoselectivity (74 : 26 dr, 78 : 22 er). Using either CF_3_-substituted (*Z*)-enoate 25 or (*E*)-enoate 8, the same (1*S*,2′*S*)-stereoisomer of product 9 was obtained in high dr and er (>95 : 5 dr, 82 : 18 er). Similar reactions were undertaken using methyl 1-acetyl-3-oxoindolinone-2-carboxylate 18 as the nucleophile (Scheme 6B); reaction with maleate 24 gave product (2*R*,2′*R*)-23 in the *opposite enantiomeric series* to that derived from fumarate 26, but with reduced stereoselectivity (87 : 13 dr, 71 : 29 er). Using CF_3_-substituted (*Z*)-enoate 25, the corresponding product 19 was obtained in high dr but in essentially *racemic* form (>95 : 5 dr, 54 : 46 er).

To decipher these results, competition experiment 1 (Scheme 6C) took a 50 : 50 ratio of nucleophiles 18 and 7 in the addition to fumarate 26, giving an 86 : 14 ratio of products 19 and 13. On the assumption of irreversible nucleophilic addition, this is consistent with the rate of addition of methyl 1-acetyl-3-oxoindolinone-2-carboxylate 18 being greater than that of ethyl 2-oxocyclopentane-1-carboxylate 7. In addition, we considered previous work that showed both isothioureas and NBu_4_OPNP can promote (*Z*)- to (*E*)-enoate isomerisation,^19,28*a*,34^ and that isomerisation of CF_3_-substituted (*Z*)-enoates occurred at a significantly faster rate than that of maleates. Application to this case (Scheme 5D) would involve *N*-acylation of HyperBTM 1 with (*Z*)-24 or (*Z*)-25 to give the corresponding (*Z*)-α,β-unsaturated acyl ammonium ion pair 27. Subsequent reversible conjugate addition of *para*-nitrophenolate (or HyperBTM 1), followed by bond rotation and elimination, will lead to the thermodynamically favoured (*E*)-enoate *via* the corresponding (*E*)-α,β-unsaturated acyl ammonium ion pair 27 (Scheme 6D). Bringing this together, the observed experimental results in Scheme 6A and B can be understood based on the relative rates of nucleophilic addition of the β-ketoesters 18 and 7 to the (*E*)- or (*Z*)-α,β-unsaturated acyl ammonium intermediate 28 or 27, alongside competitive (*Z*)- to (*E*)-enoate isomerisation. Using ethyl 2-oxocyclopentane-1-carboxylate 7 the rate of conjugate addition is slow relative to (*Z*)- to (*E*)-isomerisation, resulting in the same product enantiomer using either (*E*)- or (*Z*)-enoate as starting material. However, using methyl 1-acetyl-3-oxoindolinone-2-carboxylate 18, the conjugate addition to an intermediate (*Z*)-α,β-unsaturated acyl ammonium species must occur at a faster rate than isomerisation of maleate 24, giving preferentially the product antipode (2*R*,2′*R*)-23 compared to the reaction with fumarate 26. Using this argument, the effectively racemic nature of product 19 from CF_3_-substituted (*Z*)-enoate 25 and 18 is assumed to arise from *approximately identical* rates of (*Z*)- to (*E*)-isomerisation and conjugate addition to the (*Z*)-α,β-unsaturated acyl ammonium species. To test this hypothesis, competition experiment 2 was designed and carried out (Scheme 6E). Using a 50 : 50 mixture of CF_3_-substituted (*E*)-enoate 8 and (*Z*)-enoate 25 in the reaction of 18 was predicted to give an approximate 75 : 25 mixture of product (2*S*,2′*S*): (2*R*,2′*R*)-enantiomers; in practice product 19 was isolated in 55% yield (>95 : 5 dr, 74 : 26 er), supporting this hypothesis.

### 2.3 Proposed mechanism

**Scheme 7:**
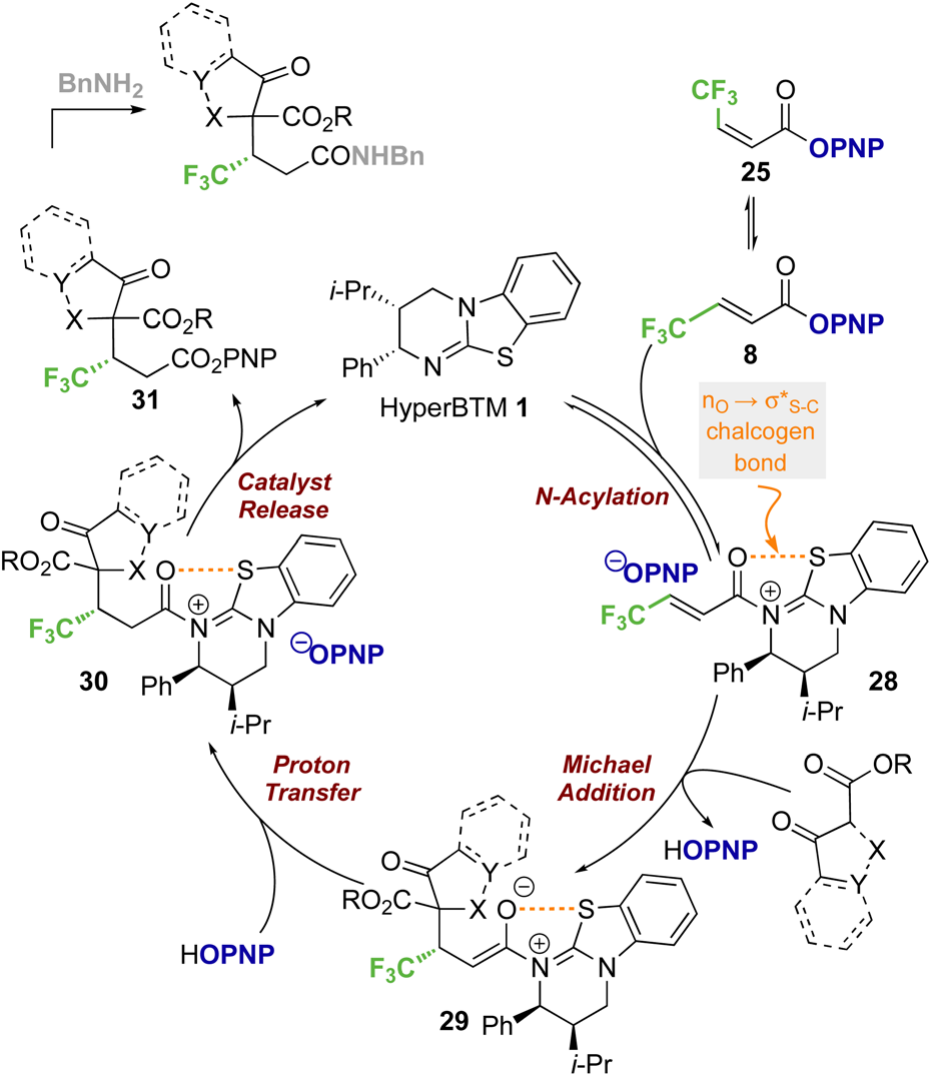
Proposed simplified catalytic cycle.

Based on previous studies and the observations reported herein,^19,26,28*a*,29,35^ a general and simplified catalytic cycle for this transformation can be proposed (Scheme 7). Isothiourea catalyst HyperBTM 1 undergoes reversible *N*-acylation with PNP ester 8, generating α,β-unsaturated acyl ammonium ion pair 28 with an S⋯O conformational lock.^23,36–43^ Deprotonation of the β-ketoester by the released *p*-nitrophenoxide, followed by subsequent stereoselective conjugate addition to the α,β-unsaturated acyl isothiouronium intermediate 28 in the assumed stereodetermining step gives enolate 29. Subsequent protonation, presumably by the generated *p*-nitrophenol, gives acyl-isothiouronium species 30. The aryloxide subsequently effects catalyst turnover to afford *p*-nitrophenyl ester 31, which upon treatment with benzylamine gives the corresponding amide product.

While this general mechanism accounts for the connectivity observed within the products, the divergent stereoselectivity observed with choice of pronucleophile was further investigated by DFT analysis.

### 2.4 DFT computation

To probe the origin of stereocontrol, DFT calculations at the M06-2X_SMD_/def2-TZVP//M06-2X_SMD_/def2-SVP level of theory were performed using Gaussian16.^44–50^ Following the pioneering computational work by Cheong *et al.*^23^ and Wang *et al.*^30^ who identified the Michael addition (C–C bond formation) between a cationic HyperBTM-*N*-acyl complex and a C nucleophile (Scheme 6) as the stereodetermining step in isothiourea-catalysed conjugate addition cyclisation reactions, our computational modelling concentrated on that step. An extensive transition-state (TS) search was conducted, exploring the conformational freedom of the approaching nucleophile (see ESI†). The diastereoselectivity for the reaction was evaluated as the difference in free energy (ΔΔ^‡^*G*) of the lowest TS leading to each of the diastereoisomeric products. From this extensive TS search, we found that the facial selectivity imposed by the catalyst was consistent with previous work, driven by the 1,5 S⋯O chalcogen interaction (*n*_O_ to 
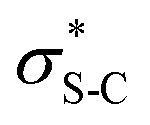
) between the acyl O and catalyst-derived S atom that provides a conformational lock, with reaction through the s-*cis* conformation.^23,41^ This places the phenyl substituent perpendicular to the plane of the catalyst, with conjugate addition *anti*-to this unit to the *si*-face of the α,β-unsaturated isothiouronium intermediate, consistent with the high enantioselectivity and absolute configuration observed.

**Fig. 1:**
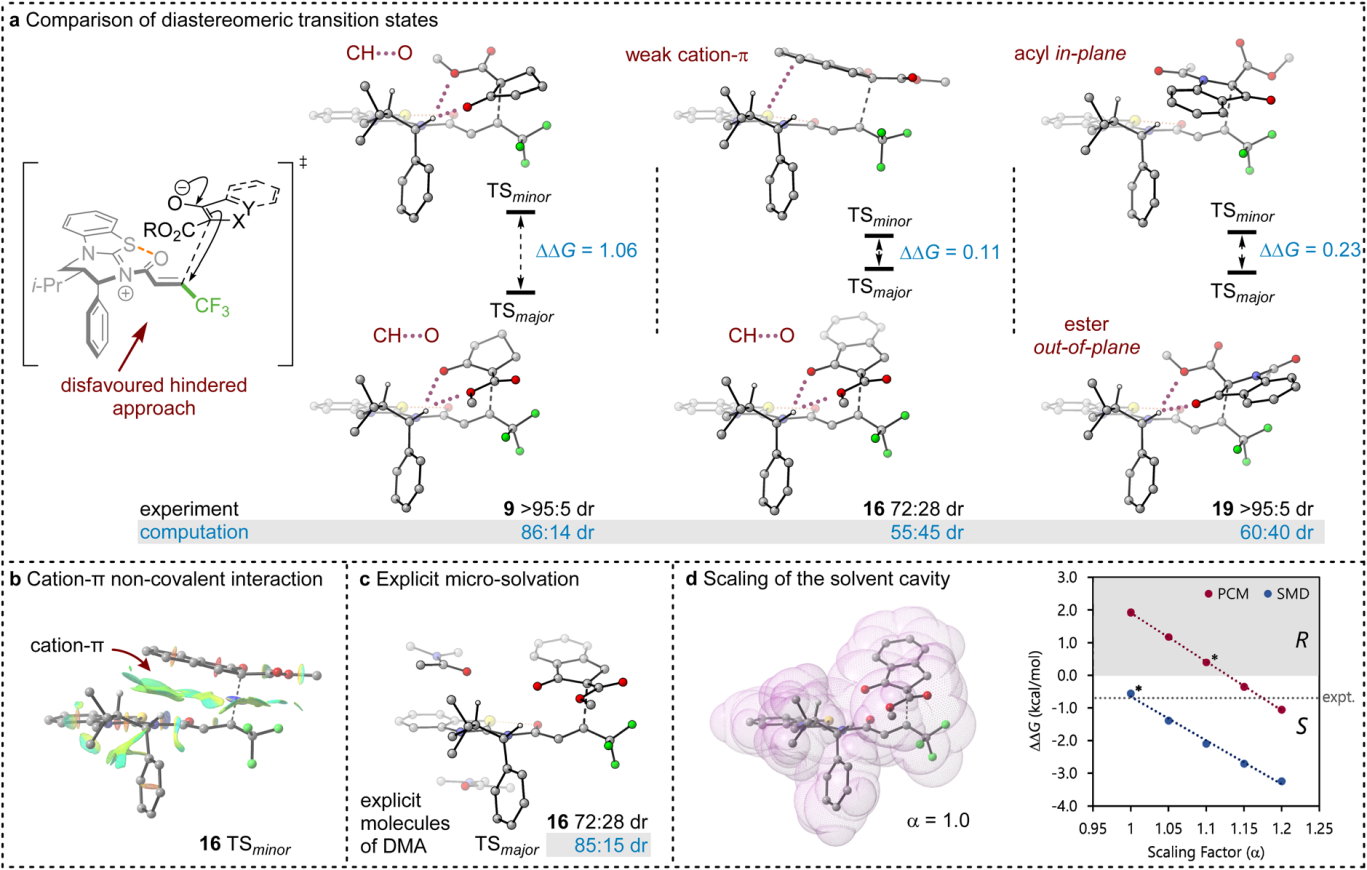
(a) DFT analysis of the most stable major and minor diastereomeric transition states. Important interactions are highlighted, with dual CH–O interactions present in all major TS. M06-2X_SMD_/def2-TZVP//M06-2X_SMD_/def2-SVP free energies (Δ*G*_298_) are shown in kcal mol^−1^ and selected hydrogen atoms have been removed for clarity. (b) Non-covalent interaction of key cation-π stabilisation of minor diastereomeric TS of 16. (c) Explicit micro-solvation with π-type interaction with conjugated DMA solvent (with PCM solvation model). (d) Solvent cavity as a sum of van der Waals radii and graph of the selectivity (ΔΔ*G*_298_) as a function of the scaling parameter. Asterisks indicate the default value of α for each solvent model.

Between the possible approaches towards the accessible *si*-face of the isothiouronium ion intermediate, substrate facial selectivity is driven by a variety of non-covalent interactions between the nucleophile and α,β-unsaturated isothiouronium intermediate. For the addition of 7 to give preferentially 9, dual non-classical CH⋯O stabilising interactions between the acidic ^+^NC-H derived from the catalyst and the ketoester of the nucleophile are observed, accounting for the preferential orientation of the major and minor TS (86 : 41 dr, Fig. 1). Experimentally, benzannulation of 7 to give ethyl 1-oxo-2,3-dihydro-1*H*-indene-2-carboxylate as the nucleophile leads to 16 with an observed reduction in dr (72 : 28 dr). This is qualitatively reproduced computationally (55 : 45 dr), with the major TS again stabilised by CH⋯O interactions between the benzylic ^+^NC–H derived from the catalyst and nucleophile. In this case, the minor diastereoisomeric TS for 16 does not contain CH⋯O interactions, but instead exhibits a stabilising cation–π interaction due to the orientation of the benzannulated ring of the nucleophile with the isothiouronium ion within the acylated catalyst (Fig. 1a and b). This interaction slightly stabilises the minor TS, reducing the energy difference between the two diastereoisomeric TS and lowering the dr. This subtle interplay between cation–π and CH⋯O interactions is fully in line with findings by Wang *et al.* for a related Michael addition reaction.^30^

The divergent selectivity observed with indoline derived substrate 19 was also investigated. The computed major TS aligns with experiment, with the minor TS lacking in CH⋯O interactions. Instead, the minor TS features π-type interactions between the substrate acyl group and the isothiouronium acylated catalyst, analogous to the cation–π interaction of 16 (see Fig. 1b).

Importantly, the choice of implicit solvent model was found to dramatically impact the ability of the computational method to reproduce experimental results. These models allow for simple representation of the solvent as a continuous dielectric but can become unreliable in the case of large dipole moments or charges. The chosen transition states are strongly zwitterionic composed of an anion derived from deprotonation of the β-ketoester and a cationic isothiouronium derived Michael acceptor.

Preliminary calculations using the Polarisable Continuum Model (PCM)^51,52^ employed in previous studies^26,50^ led to the incorrect prediction of the minor diastereoisomer of 16 (33 : 64 dr), arguably due to the poor treatment of solvation. Two alternate approaches identified the correct major diastereomer with PCM: explicit micro-solvation (85 : 15 dr, Fig. 1b) and increased scaling of the solvent cavity (≥1.15 van der Waals radii, Fig. 1c). Micro-solvation adds an explicit solvent molecule to the DFT calculation, allowing for more accurate interaction with the solvent than just an implicit model. We find stabilising π-type interactions with the conjugated DMA solvent (Fig. 1c) and calculate an improved 85 : 15 dr, in closer agreement with the experimental dr (72 : 28 dr). Similarly, the dr can be improved by scaling the solvent cavity, which in turn is constructed from the van der Waals radii (Fig. 1d). With a larger cavity, there is a greater separation between the large molecular dipole and the dielectric medium which leads to a reduction in coulombic interaction. This is larger for the minor TS than the major TS due to the larger dipole moment (*μ* = 29.54 and 19.84 D, respectively).

Both approaches introduce a greater separation between the highly charged or dipolar regions of the TS and the dielectric medium and reduce the relative overstabilisation of the minor diastereomeric TS. Unfortunately, both approaches are undesirable from a methodological point of view. Explicit micro-solvation requires vast exploration of solvation sites and conformers, ultimately calling for extensive molecular dynamics simulations to sample the relevant minima in an ensemble, and artificially tweaking parameters of a solvent model may reduce its predictive power and general applicability.

Pleasingly, the Solvation Model based on Density (SMD) correctly reproduces the major diastereoisomer without scaling the solvent cavity or introducing micro-solvation. This model includes parameterisation to account for some of the dispersive interactions between the solute and solvent, mimicking the increased accuracy brought about by micro-solvation, whilst using a smaller solvent cavity than PCM. This model appears to help in stabilising the major diastereoisomer, presumably due to the parameterised dispersive interactions in its construction even with a reduced solvent cavity compared to PCM.

## 3 Conclusions

In conclusion, the enantioselective conjugate addition of a range of carbo- and heterocyclic α-substituted β-ketoesters to α,β-unsaturated aryl esters using the isothiourea HyperBTM 1 as a Lewis base catalyst has been demonstrated. Divergent diastereoselectivity is observed through judicious choice of pro-nucleophile, with either cyclopentanone-derived or indanone-derived substituted β-ketoesters generating divergent stereodefined products with high stereoselectivity (>95 : 5 dr, up to 99 : 1 er). The scope and limitations of these processes are demonstrated, alongside application on gram scale. Competitive isomerisation alongside conjugate addition is observed with (*Z*)-enoates. The origin of the divergent stereoselectivity has been studied using DFT and can be attributed to a variety of weak non-covalent interactions between the α,β-unsaturated acyl isothiouronium intermediate and the incoming nucleophile. These weak attractive interactions modulate the steric repulsion from the stereodirecting C(2)-phenyl substituent within the catalyst–substrate α,β-unsaturated acyl isothiouronium intermediate and need to be accounted for by the computational methodology. Because the key TSs are highly polar, an adequate treatment of solvation effects in the model is instrumental. Building upon this work and the insights gained from DFT analysis, further applications of related reaction processes are currently under investigation in this laboratory.^53^

## Data availability

The research data supporting this publication can be accessed at: https://doi.org/10.17630/013114f4-2dbc-49ae-b64a-97d34f222a34.

## Author contributions

ADS and GRB conceived the project; DY, ZD and GRB carried out all experimental studies in consultation with TK and KK. ADS, DY, KK, ASG and GRB wrote the manuscript. DBC and APM carried out single crystal X-ray analysis. ASG carried out all computation in consultation with MB. All authors agreed on the finalised version of the manuscript.

## Conflicts of interest

There are no conflicts to declare.

## Supplementary Material

SC-014-D3SC05470E-s001

SC-014-D3SC05470E-s002

## References

[cit1] Liu Y., Han S. J., Liu W. B., Stoltz B. M. (2015). Catalytic Enantioselective Construction of Quaternary Stereocenters: Assembly of Key Building Blocks for the Synthesis of Biologically Active Molecules. Acc. Chem. Res..

[cit2] Ling T., Rivas F. (2016). All-Carbon Quaternary Centers in Natural Products and Medicinal Chemistry: Recent Advances. Tetrahedron.

[cit3] Hamashima Y., Hotta D., Umebayashi N., Tsuchiya Y., Suzuki T., Sodeoka M. (2005). Catalytic Enantioselective Michael Reaction of 1,3-Dicarbonyl Compounds *via* Formation of Chiral Palladium Enolate. Adv. Synth. Catal..

[cit4] Yang J., Li W., Jin Z., Liang X., Ye J. (2010). Enantioselective Michael Reaction of α-Alkyl-β-keto Esters and Enones under Multifunctional Catalysis. Org. Lett..

[cit5] Murai K., Fukushima S., Nakamura A., Shimura M., Fujioka H. (2011). C3-Symmetric chiral trisimidazoline: the role of a third imidazoline and its application to the nitro Michael reaction and the α-amination of β-ketoesters. Tetrahedron.

[cit6] Riko S., Svete J., Tefane B., Perdih A., Groelj U. (2016). 1,3-Diamine-Derived Bifunctional Organocatalyst Prepared from Camphor. Adv. Synth. Catal..

[cit7] Chen G., Liang G., Wang Y., Deng P., Zhou H. (2018). A homodinuclear cobalt complex for the catalytic asymmetric Michael reaction of β-ketoesters to nitroolefins. Org. Biomol. Chem..

[cit8] Wang L.-K., Zhou J.-J., Lan Y.-B., Ding S.-Y., Yu W., Wang W. (2019). Divergent Synthesis of Chiral Covalent Organic Frameworks. Angew. Chem., Int. Ed..

[cit9] Kabes C., Lucas R., Gunn J., Gladysz J. (2021). Chiral Cobalt(III) Tris(1,2-diamine) Catalysts That Incorporate Nitrogenous Base Containing Anions for the Bifunctional Activation of Nucleophiles and Electrophiles in Enantioselective Addition Reactions. ACS Catal..

[cit10] Wu F., Li H., Hong R., Deng L. (2005). Construction of Quaternary Stereocenters by Efficient and Practical Conjugate Additions to α,β-Unsaturated Ketones with a Chiral Organic Catalyst. Angew. Chem., Int. Ed..

[cit11] Loui H. J., Schneider C. (2022). Cooperative Palladium/Brønsted Acid Catalysis toward the Highly Enantioselective Allenylation of β-Keto Esters. Org. Lett..

[cit12] Hamashima Y., Hotta D., Sodeoka M. (2002). Direct Generation of Nucleophilic Chiral Palladium Enolate from 1,3-Dicaronyl Compounds: Catalytic Enantioselective Michael Reaction with Enones. J. Am. Chem. Soc..

[cit13] Liu J., Han Z., Wang X., Meng F., Wang Z., Ding K. (2017). Palladium-Catalyzed Asymmetric Construction of Vicinal Tertiary and All-Carbon Quaternary Stereocenters by Allylation of β-Ketocarbonyls with Morita–Baylis–Hillman Adducts. Angew. Chem., Int. Ed..

[cit14] Taylor J. E., Bull S. D., Williams J. M. (2012). Amidines, isothioureas, and guanidines as nucleophilic catalysts. Chem. Soc. Rev..

[cit15] Vellalath S., Romo D. (2016). Asymmetric Organocatalysis: The Emerging Utility of α,β-Unsaturated Acylammonium Salts. Angew. Chem., Int. Ed..

[cit16] McLaughlin C., Smith A. D. (2021). Generation and Reactivity of C(1)-Ammonium Enolates by Using Isothiourea Catalysis. Chem.–Eur. J..

[cit17] Robinson E. R. T., Fallan C., Simal C., Slawin A. M. Z., Smith A. D. (2013). Anhydrides as α,β-unsaturated acyl ammonium precursors: isothiourea-promoted catalytic asymmetric annulation processes. Chem. Sci..

[cit18] Liu G., Shirley M. E., Van K. N., McFarlin R. L., Romo D. (2013). Rapid assembly of complex cyclopentanes employing chiral, α,β-unsaturated acylammonium intermediates. Nat. Chem..

[cit19] Matviitsuk A., Greenhalgh M. D., Barrios Antuñez D. J., Slawin A. M. Z., Smith A. D. (2017). Aryloxide-Facilitated Catalyst Turnover in Enantioselective α,β-Unsaturated Acyl Ammonium Catalysis. Angew. Chem., Int. Ed..

[cit20] Fukata Y., Asano K., Matsubara S. (2015). Facile Net Cycloaddition Approach to Optically Active 1,5-Benzothiazepines. J. Am. Chem. Soc..

[cit21] Jin J.-H., Li X.-Y., Luo X., Deng W.-P. (2018). Enantioselective synthesis of indolo[2,3-*b*]-dihydrothiopyranones *via* [3+3] cycloaddition of chiral α,β-unsaturated acylammonium salts. Tetrahedron.

[cit22] Kurihara T., Kojima M., Yoshino T., Matsunaga S. (2022). Achiral Cp*Rh(III)/Chiral Lewis Base Cooperative Catalysis for Enantioselective Cyclisation *via* C–H Activation. J. Am. Chem. Soc..

[cit23] Robinson E. R. T., Walden D. M., Fallan C., Greenhalgh M. D., Cheong P. H.-Y., Smith A. D. (2016). Non-bonding 1,5-S···O interactions govern chemo- and enantioselectivity in isothiourea-catalyzed annulations of benzazoles. Chem. Sci..

[cit24] Hartley W. C., O'Riordan T. J. C., Smith A. D. (2017). Aryloxide-Promoted Catalyst Turnover in Lewis Base Organocatalysis. Synthesis.

[cit25] Liu H., Slawin A. M. Z., Smith A. D. (2020). Isothiourea-Catalyzed Enantioselective Synthesis of Tetrahydro-α-carbolinones. Org. Lett..

[cit26] Wu J., Young C. M., Watts A. A., Slawin A. M. Z., Boyce G. R., Bühl M., Smith A. D. (2022). Isothiourea-Catalyzed Enantioselective Michael Addition of Malonates to α,β-Unsaturated Aryl Esters. Org. Lett..

[cit27] Lapetaje J. E., Young C. M., Shu C., Smith A. D. (2022). Isothiourea-catalyzed formal enantioselective conjugate addition of benzophenone imines to β-fluorinated α,β-unsaturated esters. Chem. Commun..

[cit28] Bitai J., Nimmo A. J., Slawin A. M. Z., Smith A. D. (2022). Cooperative Palladium/Isothiourea Catalyzed Enantioselective Formal (3+2) Cycloaddition of Vinylcyclopropanes and α,β-Unsaturated Esters. Angew. Chem., Int. Ed..

[cit29] Greenhalgh M. D., Qu S., Slawin A. M. Z., Smith A. D. (2018). Multiple orles of aryloxide leaving groups in enantioselective annulations employing α,β-unsaturated acyl ammonium catalysis. Chem. Sci..

[cit30] Wang C., Li S.-J., Zhang Q.-C., Wei D., Ding L. (2020). Insights into isothiourea-catalyzed asymmetric [3 + 3] annulation of α,β-unsaturated aryl esters with 2-acylbenzazoles: mechanism, origin of selectivity and switchable chemoselectivity. Catal. Sci. Technol..

[cit31] CCDC 2298980 contains the supplementary crystallographic data for (1S,2’S)–9

[cit32] CCDC 2298981 contains the supplementary crystallographic data for (2*S*,2’*S*)–16

[cit33] CCDC 2298982 contains the supplementary crystallographic data for (2*S*,2’S)–20

[cit34] Morrill L. C., Douglas J. J., Lebl T., Slawin A. M. Z., Fox D. J., Smith A. D. (2013). Isothiourea-mediated asymmetric Michael-lactonisation of trifluoromethylenones: a synthetic and mechanistic study. Chem. Sci..

[cit35] Shu C., Liu H., Slawin A. M. Z., Carpenter-Warren C., Smith A. D. (2020). Isothiourea-catalysed enantioselective Michael addition of N-heterocyclic pronucleophiles to α,β-unsaturated aryl esters. Chem. Sci..

[cit36] Bleiholder C., Gleiter R., Werz D. B., Köppel H. (2007). Theoretical Investigations on Heteronuclear Chalcogen–Chalcogen Interactions: On the Nature of Weak Bonds between Chalcogen Centers. Inorg. Chem..

[cit37] Gleiter R., Haberhauer G., Werz D. B., Rominger F., Bleiholder C. (2018). From Noncovalent Chalcogen–Chalcogen Interactions to Supramolecular Aggregates: Experiments and Calculations. Chem. Rev..

[cit38] Kolb S., Oliver G. A., Werz D. B. (2020). Chemistry Evolves, Terms Evolve, but Phenomena Do Not Evolve: From Chalcogen–Chalcogen Interactions to Calcogen Bonding. Angew. Chem., Int. Ed..

[cit39] Pascoe D. J., Ling K. B., Cockroft S. L. (2017). The Origin of Chalcogen-Bonding Interactions. J. Am. Chem. Soc..

[cit40] Benz S., López-Andarias J., Mareda J., Sakai N., Matile S. (2017). Catalysis with Chalcogen Bonds. Angew. Chem., Int. Ed..

[cit41] Birman V. B., Li X., Han Z. (2007). Nonaromatic Amidine Derivatives as Acylation Catalysts. Org. Lett..

[cit42] Nagao Y., Miyamoto S., Miyamoto M., Takeshige H., Hayashi K., Sano S., Shiro M., Yamaguchi K., Sei Y. (2006). Highly Stereoselective Asymmetric Pummerer Reactions That Incorporate Intermolecular and Intramolecular Nonbonded S⋯O Interactions. J. Am. Chem. Soc..

[cit43] Beno B. R., Yeung K.-S., Bartberger M. D., Pennington L. D., Meanwell N. A. (2015). J. Med. Chem.. A Survey of the Role of Noncovalent Sulfur Interactions in Drug Design.

[cit44] Zhao Y., Truhlar D. G. (2008). The M06 suite of density functionals for main group thermochemistry, thermochemical kinetics, noncovalent interactions, excited states, and transition elements: Two new functionals and systematic testing of four M06-class functionals and 12 other functionals. Theor. Chem. Acc..

[cit45] Schäfer A., Horn H., Ahlrichs R. (1992). Fully optimized contracted Gaussian basis sets for atoms Li to Kr. J. Chem. Phys..

[cit46] Schäfer A., Huber C., Ahlrichs R. (1994). Fully optimized contracted Gaussian basis sets of triple zeta valence quality for atoms Li to Kr. J. Chem. Phys..

[cit47] Weigend F., Ahlrichs R. (2005). Balanced basis sets of split valence, triple zeta valence and quadruple zeta valence quality for H to Rn: design and assessment of accuracy. Phys. Chem. Chem. Phys..

[cit48] Weigend F. (2006). Accurate Coulomb-fitting basis sets for H to Rn. Phys. Chem. Chem. Phys..

[cit49] Marenich A. V., Cramer C. J., Truhlar D. G. (2009). Universal solvation model based on solute electron density and on a continuum model of the solvent defined by the bulk dielectric constant and atomic surface tensions. J. Phys. Chem. B.

[cit50] Frisch M. J. , Trucks G. W., Schlegel H. B., Scuseria G. E., Robb M. A., Cheeseman J. R., Scalmani G., Barone V., Petersson G. A., Nakatsuji H., Li X., Caricato M., V Marenich A., Bloino J., Janesko B. G., Gomperts R., Mennucci B., Hratchian H. P., V Ortiz J., Izmaylov A. F., Sonnenberg J. L., Williams-Young D., Ding F., Lipparini F., Egidi F., Goings J., Peng B., Petrone A., Henderson T., Ranasinghe D., Zakrzewski V. G., Gao J., Rega N., Zheng G., Liang W., Hada M., Ehara M., Toyota K., Fukuda R., Hasegawa J., Ishida M., Nakajima T., Honda Y., Kitao O., Nakai H., Vreven T., Throssell K., Montgomery, Jr J. A., Peralta J. E., Ogliaro F., Bearpark M. J., Heyd J. J., Brothers E. N., Kudin K. N., Staroverov V. N., Keith T. A., Kobayashi R., Normand J., Raghavachari K., Rendell A. P., Burant J. C., Iyengar S. S., Tomasi J., Cossi M., Millam J. M., Klene M., Adamo C., Cammi R., Ochterski J. W., Martin R. L., Morokuma K., Farkas O., Foresman J. B. and Fox D. J., Gaussian 16, Revision C.01, Gaussian Inc., Wallingford CT, 2019

[cit51] Mennucci B., Tomasi J. (1997). Continuum solvation models: a new approach to the problem of solute's charge distribution and cavity boundaries. J. Chem. Phys..

[cit52] Tomasi J., Mennucci B., Cancès E. (1999). The IEF version of the PCM solvation method: an overview of a new method addressed to study molecular solutes at the QM *ab initio* level. J. Mol. Struct..

[cit53] Understanding Divergent Substrate Stereoselectivity in the Enantioselective Isothiourea-Catalysed Conjugate Addition of Cyclic α-substituted β-ketoesters to α,β-unsaturated Aryl Esters, University of St Andrews Research Portal, PURE ID: 29438761

