# Supplementary material for: Understanding divergent substrate stereoselectivity in the isothiourea-catalysed conjugate addition of cyclic α-substituted β-ketoesters to α,β-unsaturated aryl esters
Source: Chem Sci. 2023 Nov 21;14(48):14146–56. doi: 10.1039/d3sc05470e (PMC10717594; doi:10.1039/d3sc05470e)
Supplement: SC-014-D3SC05470E-s001 [file SC-014-D3SC05470E-s001.pdf]

Electronic Supplementary Material (ESI) for Chemical Science.
This journal is © The Royal Society of Chemistry 2023

Supporting Information

Understanding Divergent Substrate Stereoselectivity in the Enantioselective Isothiourea-Catalysed Conjugate Addition of Carbo- and Heterocyclic α-substituted β-ketoesters to α,β-unsaturated Aryl Esters

Ding Yuan,[a,b] Alister S. Goodfellow,[a] Tengfei Kang,[a] Kevin Kasten,[a] Zhuan Duan,[a] David B. Cordes,[a] Michael Bühl,[a]* Aidan. P. McKay,[a] Gregory R. Boyce[a,c,d]* and Andrew D. Smith[a]*

[a] EaStCHEM, School of Chemistry, University of St Andrews, St Andrews, Fife, KY16 9ST, UK. E-mail: ads10@st-andrews.ac.uk
[b] School of Biological and Chemical Engineering, Panzhihua University, Panzhihua 617000, China
[c] Department of Chemistry and Physics, Florida Gulf Coast University, Fort Myers, Florida 33965, United States
[d] Department of Chemistry and Biochemistry, East Stroudsburg University, East Stroudsburg, Pennsylvania 18301, United States. E-mail: gboyce@esu.edu

Contents

General Information 3
General Procedures 4
Optimisation 5
Synthesis Procedures 6
Synthesis of *α*, *β*-Unsaturated Aryl Esters 6
4-Nitrophenyl (*E*)-4,4,4-trifluorobut-2-enoate (8) 6
Ethyl (4-nitrophenyl) maleate (24) 7
Ethyl (4-nitrophenyl) fumarate (26) 8
4-Nitrophenyl (*E*)-4-chloro-4,4-difluorobut-2-enoate (S1) 9
4-Nitrophenyl (*E*)-4-bromo-4,4-difluorobut-2-enoate (S2) 11
4-Nitrophenyl (*E*)-4,4,5,5,5-pentafluoropent-2-enoate (S3) 12
4-Nitrophenyl (*E*)-4,4-difluorobut-2-enoate (S4) 14
Synthesis of β-Ketoesters 15
Methyl 1-acetyl-3-oxoindoline-2-carboxylate (18) 15
Ethyl 1-oxo-2,3-dihydro-1*H*-indene-2-carboxylate (S5) 16
Ethyl 3-oxo-2,3-dihydrobenzofuran-2-carboxylate (S6) 17
Synthesis of the Michael Addition Products 19
Ethyl (S)-1-((S)-4'-(benzylamino)-1',1',1'-trifluoro-4'-oxobutan-2'-yl)-2-oxocyclopentane-1-carboxylate (1S,2'S)-9 19
Ethyl (S)-1-((S)-4'-(benzylamino)-1'-chloro-1',1'-difluoro-4'-oxobutan-2'-yl)-2-oxocyclopentane-1-carboxylate (1S,2'S)-10 24
Ethyl (*S*)-1-((*S*)-4'-(benzylamino)-1'-bromo-1',1'-difluoro-4'-oxobutan-2'-yl)-2-oxocyclopentane-1-carboxylate (1*S*,2'*S*)-11 28
Ethyl (*S*)-1-((*S*)-5-(benzylamino)-1,1,1,2,2-pentafluoro-5-oxopentan-3-yl)-2-oxocyclopentane-1-carboxylate (1*S*,2'*S*)-12 32

Ethyl (S)-1-((S)-4'-(benzylamino)-1'-ethoxy-1',4'-dioxobutan-2'-yl)-2-oxocyclopentane -1-carboxylate (1S,2'S)-13 36

1-(*tert*-Butyl) 2-ethyl (*S*)-2-((*S*)-4'-(benzylamino)-1',1',1'-trifluoro-4'-oxobutan-2'-yl)-3-oxopyrrolidine-1,2-dicarboxylate (2*R*,2'*S*)-14 43

1-(*tert*-butyl) 3-ethyl (*R*)-3-((*S*)-4'-(benzylamino)-1',1',1'-trifluoro-4'-oxobutan-2'-yl)-4-oxopyrrolidine-1,3-dicarboxylate (3*R*,2'*S*)-15 48

Ethyl (S)-2-((S)-4'-(benzylamino)-1',1',1'-trifluoro-4'-oxobutan-2'-yl)-1-oxo-2,3-dihydro-1*H*-indene-2-carboxylate (2*S*,2'*S*)-16 52

Ethyl (S)-2-((S)-4'-(benzylamino)-1',1',1'-trifluoro-4'-oxobutan-2'-yl)-3-oxo-2,3-dihydrobenzofuran-2-carboxylate (2S,2'S)-17 59

Methyl (S)-1-acetyl-2-((S)-4'-(benzylamino)-1',1',1'-trifluoro-4'-oxobutan-2'-yl)-3-oxoindoline-2-carboxylate (2S,2'S)-19 63

Methyl (S)-1-acetyl-2-((S)-4-(benzylamino)-1-chloro-1,1-difluoro-4-oxobutan-2-yl)-3-oxoindoline-2-carboxylate (2S,2'S)-20 68

Methyl (S)-1-acetyl-2-((S)-4'-(benzylamino)-1'-bromo-1',1'-difluoro-4'-oxobutan-2'-yl)-3-oxoindoline-2-carboxylate (2S,2'S)-21 73

Methyl (S)-1-acetyl-2-((S)-4-(benzylamino)-1,1,1,2,2-pentafluoro-5-oxopentan-3-yl)-3-oxoindoline-2-carboxylate (2S,2'S)-22 76

Methyl (S)-1-acetyl-2-((S)-4-(benzylamino)-1-ethoxy-1,4-dioxobutan-2-yl)-3-oxoindoline-2-carboxylate (2S,2'S)-23 80

Ethyl (S)-1-((S)-4'-(benzylamino)-1',1'-difluoro-4'-oxobutan-2'-yl)-2-oxocyclopentane-carboxylate (2*S*,2'*S*)-S7 and *rel*-(2*S*,2'*R*)-S7 86

Determination of Product Configuration by X-Ray Crystallography 100

Computational Details 108

Conformational Flexibility (PCM//PCM) 109

Conformational Flexibility (SMD//SMD) 110

Visualisation of non-covalent interactions (SMD//SMD) 111

Comparison of solvent models 112

Computational Data 116

References 476

General Information

Reactions involving moisture sensitive reagents were carried out in flame-dried glassware under an argon or nitrogen atmosphere using standard vacuum line techniques and using anhydrous solvents. Anhydrous solvents (CH_2Cl_2 and toluene) were obtained from an anhydrous solvent system (purified using an alumina column, Mbraun SPS-800). All other reactions were performed in standard glassware with no precautions to exclude air or moisture. Solvents and commercial reagents were used as supplied without further purification unless otherwise stated. (2*S*,3*R*)-HyperBTM **1** was prepared from a literature procedure.[1] Pyrrolidine dicarboxylates and β-ketoester **7** were purchased from fluorochem and used without further purification. All diastereomeric ratios were determined by ^{1}H NMR analysis of the purified reaction mixtures after column chromatography.

Room temperature (r.t.) refers to 20−25 °C. Temperatures of 0 °C and −78 °C were obtained using ice/water and CO_2(s)/acetone baths, respectively. Reflux conditions were obtained using a DrySyn, oil bath, or sand bath equipped with a contact thermometer.

Analytical thin layer chromatography was performed on pre-coated aluminium plates (Kieselgel 60 F_{254} silica).[2] TLC visualisation was carried out with ultraviolet light (254 nm), followed by staining with a 1% aqueous $KMnO_4$ solution. Manual column chromatography was performed in glass columns fitted with porosity 3 sintered discs over Kieselgel 60 silica using the solvent system stated.[3] Automated chromatography was performed on a Biotage® Selekt™ SEL-2SV running Selekt 1.4.2-13403 software on OS 6.1-i386-182-6 with a 200 – 400 nm UV-detector using the method stated and cartridges filled with Kieselgel 60 silica.

Melting points were recorded on an Electrothermal 9100 melting point apparatus and are uncorrected.

Optical rotations $[\alpha]_D^{20}$ were measured on a PerkinElmer Model 341 polarimeter operating at the sodium D line with a 100 mm path cell at 20 °C.

HPLC analyses were obtained using either a Shimadzu HPLC consisting of a DGU-20A5 degassing unit, LC-20AT liquid chromatography pump, SIL-20AHT autosampler, CMB-20A communications bus module, SPD-M20A diode array detector and a CTO-20A column oven; or a Shimadzu HPLC consisting of a DGU-20A5R degassing unit, LC-20AD liquid chromatography pump, SIL-20AHT autosampler, SPD-20A UV/Vis detector and a CTO-20A column oven. Separation was achieved using DAICEL CHIRALCEL OD-H or DAICEL CHIRALPAK AD-H or AS-H columns. All HPLC traces of enantiomerically-enriched compounds were compared with authentic racemic spectra.

^{1}H, ^{13}C, ^{19}F nuclear magnetic resonance (NMR) spectra were acquired on either a Bruker Avance 300, Bruker Avance II 400 or a Bruker Avance II 500 spectrometer at ambient temperature in the deuterated solvent stated. All chemical shifts are quoted in parts per million (ppm) and referenced to residual solvent peak reported in the literature.[4-7] All coupling constants, *J*, are quoted in Hz.

Multiplicities are indicated by s (singlet), d (doublet), t (triplet), q (quartet), and combinations thereof, and m (multiplet). The abbreviation Ar is used to denote aromatic, Ph to denote phenyl, Bn to denote benzyl, br to denote broad and app to denote apparent.[8-10]

Infrared spectra (*ν*$_{max}$) were recorded on a Shimadzu IRAffinity-1 Fourier transform IR spectrophotometer fitted with a Specac Quest ATR accessory (diamond puck). Spectra were recorded of either thin films or solids, with characteristic absorption wave numbers (max) reported in cm^{-1}.

High Resolution Mass spectrometry (HRMS) data were acquired by electrospray ionization time-of-flight (ESI-TOF) on a Micromass GCT at the University of St Andrews with values reported in Daltons [Da].[11, 12]

Common chemical abbreviations were used to indicate chemical groups or environments such as Ph (phenyl), Ar (aromatic, not confuse with Argon), Bn (benzyl), Et (ethyl), Me (methyl). To indicate atoms numbering schemes are displayed with the spectrum and deviate from IUPAC numbering for clarity. For names and numbering concerning stereodiscriptors IUPAC nomenclature was applied.[13-49]

General Procedures

General Procedure 1: *Synthesis of α,β-unsaturated PNP esters*

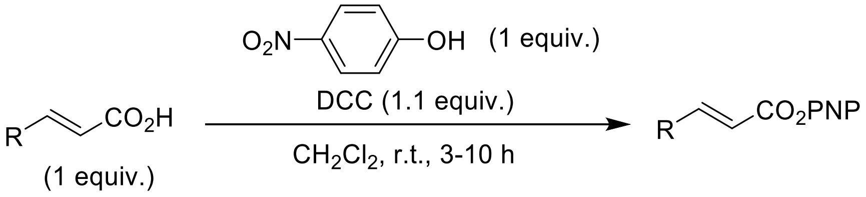

To a solution of corresponding α,β-unsaturated carboxylic acid (1.0 equiv.) in anhydrous CH_2Cl_2 (0.1 M) was added *N,N'*-dicyclohexylcarbodiimide (1.1 equiv.) at room temperature and stirred for 0.5 h. 4-Nitrophenol (1.0 equiv.) was added and the mixture was stirred until the reaction was complete. The reaction was then filtered, and the solvent was removed under reduced pressure. The residue was purified by column chromatography to afford the title compound.

General Procedure 2: *Asymmetric Michael addition of β-ketoester to α,β-unsaturated aryl esters*

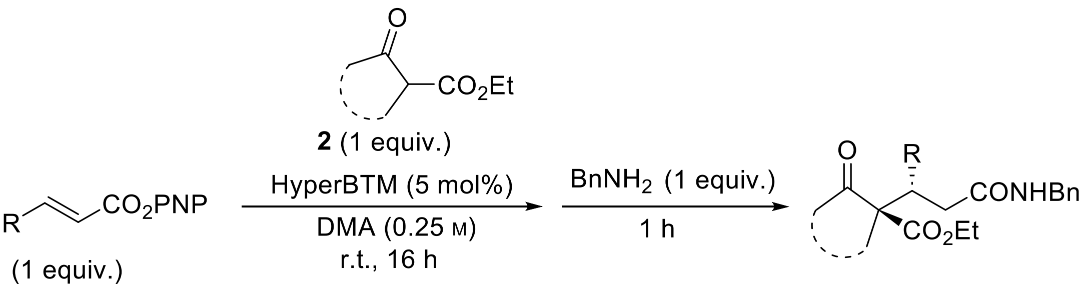

A mixture of α,β-unsaturated ester (1.0 equiv.), β-ketoester (1.0 equiv.), and (2*S*,3*R*)-HyperBTM **1** (5 mol%) in DMA (0.1 M) was stirred for 16 h at room temperature. Benzylamine (1.0 equiv.) was added,

and the reaction was permitted to stir for 1 h. The solution was diluted with Et_2O or CH_2Cl_2, washed sequentially with saturated aqueous Na_2CO_3 (3×), brine, dried over $MgSO_4$, filtered, and concentrated under reduced pressure. The residue was purified by column chromatography to afford the title compound.

Optimisation

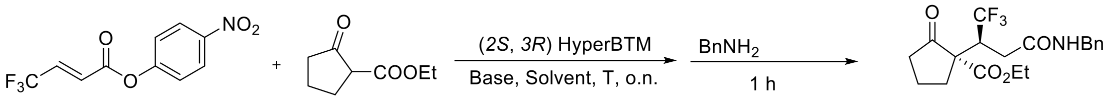

entry	solvent (0.25 M)	base	T	catalyst (mol %)	nucleophile equivalence	conversion(%)[a]
**1**	DCM	-	rt	20	1.5	0
**2**	DCM	-	0 °C	20	1.5	0
**3**	DCM	10% Hünig's	rt	20	1.5	0
**4**	MeCN	10% Hünig's	rt	20	1.5	0
**5**	EtOAc	-	rt	20	1.5	0
**6**	EtOAc	10% Hünig's	rt	20	1.5	0
**7**	EtOAc	10% NEt_3	rt	20	1.5	0
**8**	EtOAc	10% DBU	rt	20	1.5	0
**9**	DMA	-	rt	20	1.5	70
**10**	DMF	-	rt	20	1.5	67
**11**	NMP	-	rt	20	1.5	41
**12**	DMA	-	rt	20	1.5	63
**13**	DMA	-	rt	20	1	66
**14**	DMA	-	rt	20	1.2	68
**15**	DMA	-	rt	20	2	70
**16**	DMA	-	rt	20	3	60
**17**	DMA	-	rt	10	1	66
**18**	DMA	-	rt	5	1	61
**19**	DMA	-	rt	2.5	1	55
**20**	DMA	-	rt	1	1	41

[a]Conversion was determined by F NMR.

Synthesis Procedures

Synthesis of α, β-Unsaturated Aryl Esters

4-Nitrophenyl (*E*)-4,4,4-trifluorobut-2-enoate (8)

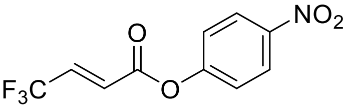

The title compound was prepared according to **General Procedure 1**, (*E*)-4,4,4-trifluorobut-2-enoic acid (140 mg, 1.0 mmol) in anhydrous CH_2Cl_2 (0.1 M) was added *N,N'*-dicyclohexylcarbodiimide (226.6 mg, 1.1 mmol) at room temperature and stirred for 0.5 h. 4-Nitrophenol (139 mg, 1.0 mmol) was added and the mixture was stirred for 8 h. The reaction was then filtered, and the solvent was removed under reduced pressure. The residue was purified by column chromatography (2:1 Hexane:EtOAc) to give the title compound (161.9 mg, 62%) as a white solid. **mp** 88–90 ºC (lit.[50] 86-88 ºC); **IR ν_{max}** (film) / cm^{-1} 3123, 3094, 2934, 1937, 1738, 1618, 1526, 1487, 1306, 1128, 972, 869; **^{1}H NMR** (400 MHz, $CDCl_3$) δ_H: 6.74 (1H, dq, *J* 15.7, 1.9, $F_3CCH{=}CH$), 7.02 (1H, dq, *J* 15.8, 6.3, $F_3CCH{=}CH$), 7.34-7.38 (2H, m, $ArC^{2,6}H$), 8.30-8.34 (2H, m, $ArC^{3,5}H$); **^{19}F{^{1}H} NMR** (376 MHz, $CDCl_3$) δ_F: –65.37 (CF_3). Data in accordance with literature.[50]

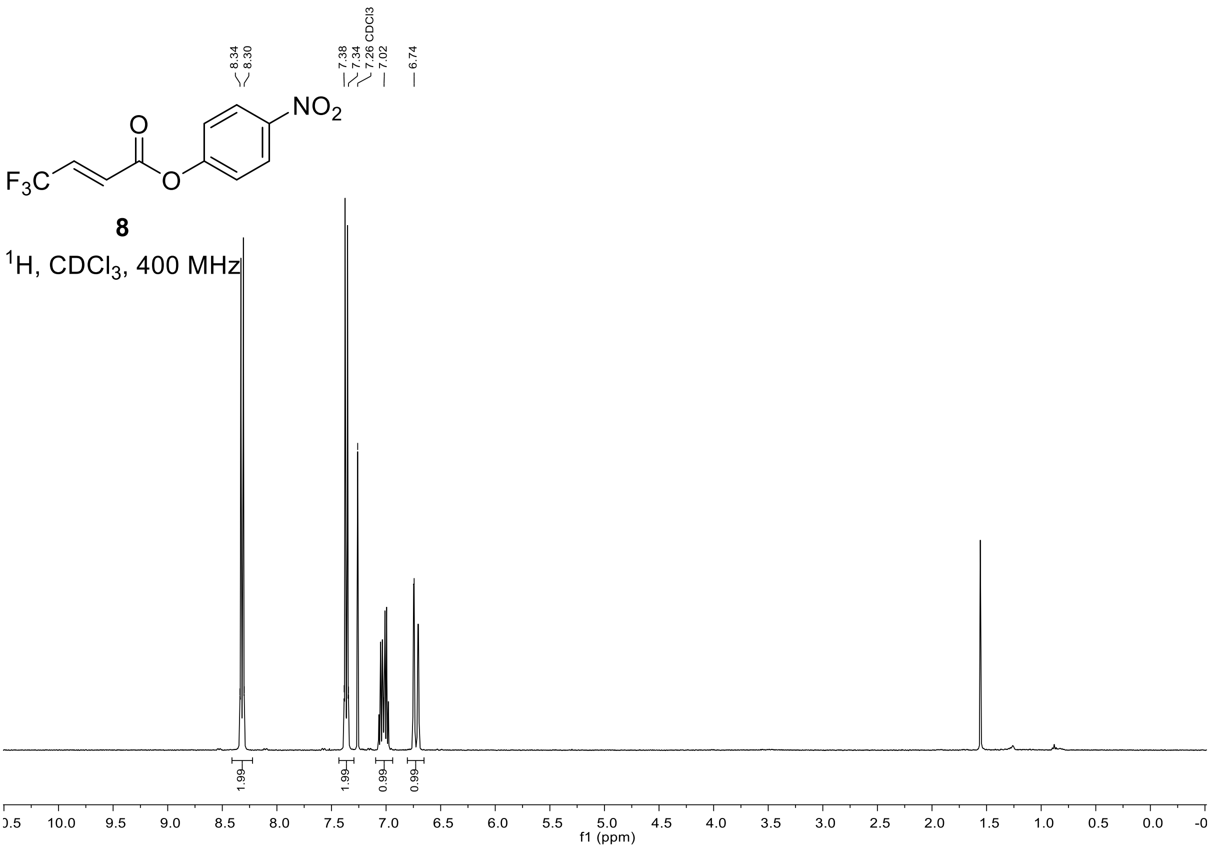

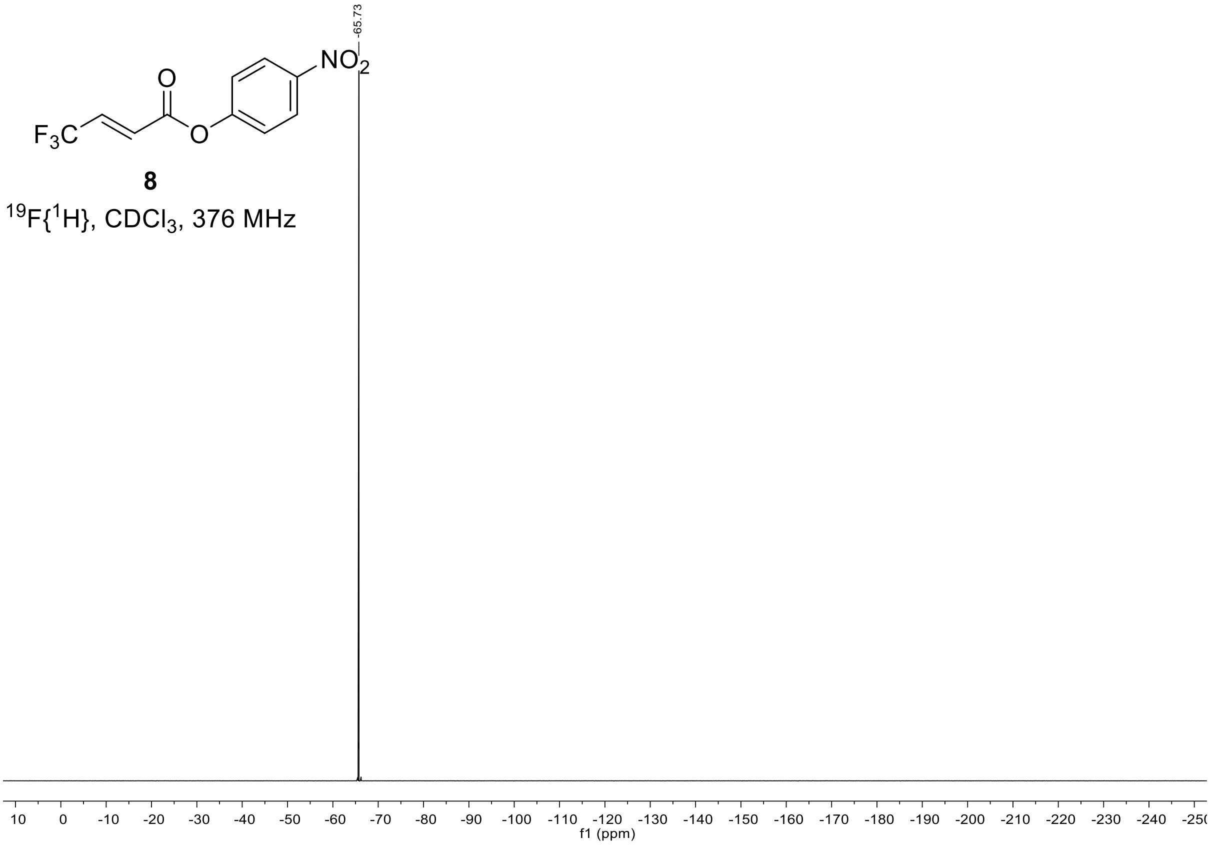

Ethyl (4-nitrophenyl) maleate (24)

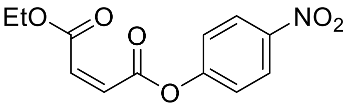

The title compound was prepared according to **General Procedure 1**, (*Z*)-4-ethoxy-4-oxobut-2-enoic acid (144 mg, 1.0 mmol) in anhydrous CH_2Cl_2 (0.1 M) was added *N,N'*-dicyclohexylcarbodiimide (226.6 mg, 1.1 mmol) at room temperature and stirred for 0.5 h. 4-Nitrophenol (139 mg, 1.0 mmol) was added and the mixture was stirred for 2 h. The reaction was then filtered, and the solvent was removed under reduced pressure. The residue was purified by column chromatography (2:1 Hexane:EtOAc) to give the title compound (191.9 mg, 73%) as a white solid. **mp** 58–60 ºC (lit.[51] 59–61 ºC); **IR ν_{max}** (film) / cm^{-1} 3121, 2988, 1732, 1531, 1396, 1348, 1211, 1152, 1013, 866, 841; **^{1}H NMR** (400 MHz, $CDCl_3$) δ_H: 1.30 (3H, t, *J* 7.1, C*H*$_3$), 4.26 (2H, q, *J* 7.1, C*H*$_2$), 6.43 (1H, d, *J* 11.8, C*H*=CH), 6.48 (1H, d, *J* 11.8, CH=C*H*), 7.38-7.42 (2H, m, ArC^{2,6}*H*), 8.26-8.30 (2H, m, ArC^{3,5}*H*). Data in accordance with literature.[51]

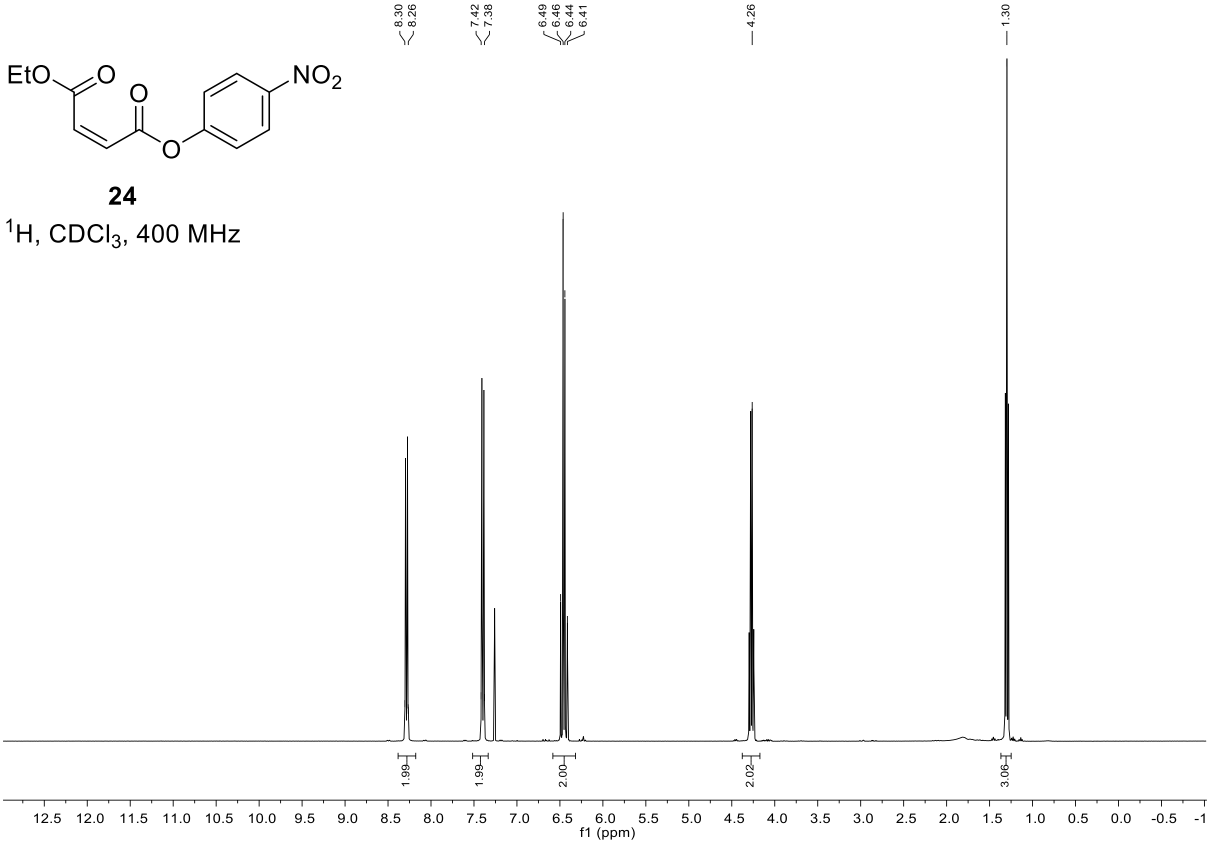

Ethyl (4-nitrophenyl) fumarate (26)

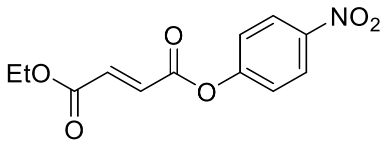

The title compound was prepared according to **General Procedure 1**, (*E*)-4-ethoxy-4-oxobut-2-enoic acid (144 mg, 1.0 mmol) in anhydrous CH_2Cl_2 (0.1 M) was added *N,N'*-dicyclohexylcarbodiimide (226.6 mg, 1.1 mmol) at r.t. and stirred for 0.5 h. 4-Nitrophenol (139 mg, 1.0 mmol) was added and the mixture was stirred for 2 h. The reaction was then filtered, and the solvent was removed under reduced pressure. The residue was purified by column chromatography (2:1 Hexane:EtOAc) to give the title compound (201.4 mg, 76%) as a white solid. **mp** 68–70 ºC (lit.[50] 67–68 ºC); **IR** ν_{max} (film) / cm^{-1} 3115, 3073, 2997, 2909, 1717, 1593, 1512, 1489, 1350, 1211, 991, 935, 874; **^{1}H NMR** (400 MHz, $CDCl_3$) δ_H: 1.35 (3H, t, *J* 7.1, C*H*$_3$), 4.21 (2H, q, *J* 7.1, C*H*$_2$), 7.06 (1H, d, *J* 15.7, C*H*=CH), 7.07 (1H, d, *J* 15.7, CH=C*H*), 7.34-7.38 (2H, m, ArC^{2,6}*H*), 8.29-8.32 (2H, m, ArC^{3,5}*H*). Data in accordance with literature.[50]

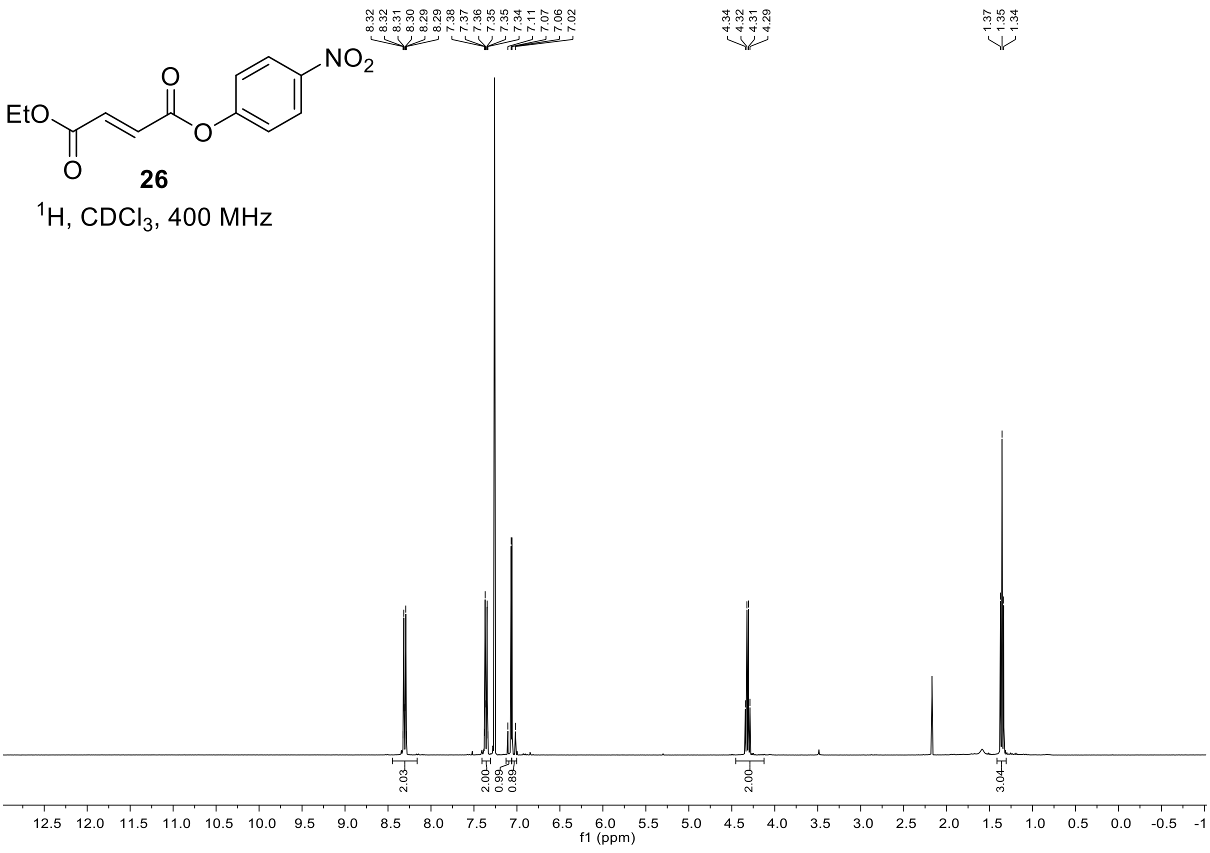

4-Nitrophenyl (*E*)-4-chloro-4,4-difluorobut-2-enoate (S1)

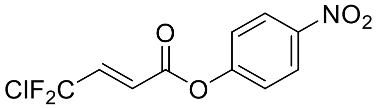

The title compound was prepared according to **General Procedure 1**, (*E*)-4-chloro-4,4-difluorobut-2-enoic acid (156 mg, 1.0 mmol) in anhydrous CH_2Cl_2 (0.1 M) was added *N,N'*-dicyclohexylcarbodiimide (226.6 mg, 1.1 mmol) at room temperature and stirred for 0.5 h. 4-Nitrophenol (139mg, 1.0 mmol) were added and the mixture was stirred for 3 h. The reaction was then filtered, and the solvent was removed under reduced pressure. The residue was purified by column chromatography (2:1 Hexane:EtOAc) to give the title compound (141.3 mg, 51%) as a white solid. **mp** 73–76 ºC (lit.[50] 74-76 ºC); **IR** ν_{max} (film) / cm^{-1} 3119, 3084, 1740, 1524, 1350, 1314, 1277, 1202, 1155, 1103, 949, 860, 816; **^{1}H NMR** (400 MHz, $CDCl_3$) δ_H: 6.62 (1H, dt, *J* 15.6, 1.8, $ClF_2CCH{=}C\mathit{H}$), 7.16 (1H, dt, *J* 15.5, 9.0, $ClF_2CC\mathit{H}{=}CH$), 7.34-7.38 (2H, m, ArC^{2,6}*H*), 8.30-8.34 (2H, m, ArC^{3,5}*H*); **^{19}F{^{1}H} NMR** (376 MHz, $CDCl_3$) δ_F: –54.47 (CF_2Cl). Data in accordance with literature.[50]

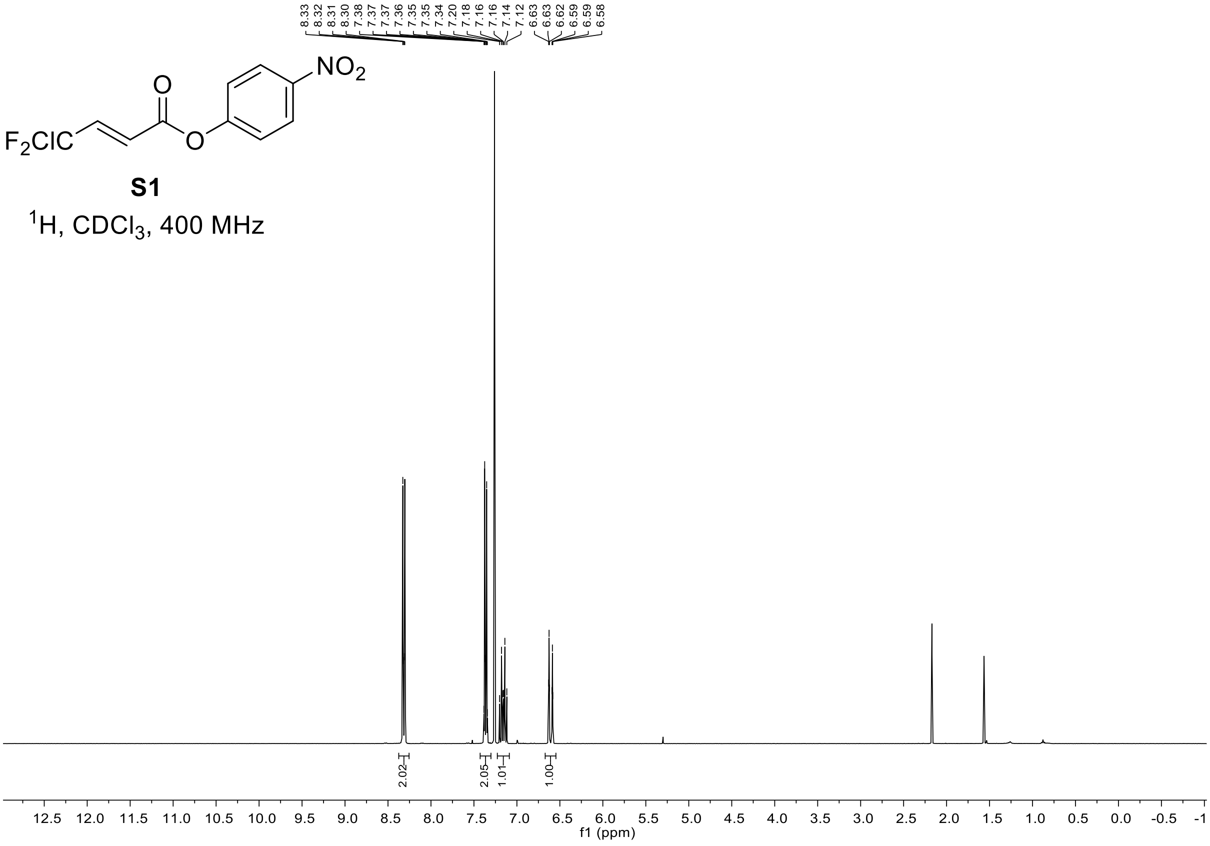
8.33
8.32
8.31
8.30
7.38
7.37
7.37
7.36
7.35
7.35
7.34
7.20
7.18
7.16
7.14
7.12
6.63
6.63
6.62
6.59
6.59
6.58
NO2
O
F2ClC
O
S1
1H, CDCl3, 400 MHz
2.02
2.05
1.01
1.00
12.5 12.0 11.5 11.0 10.5 10.0 9.5 9.0 8.5 8.0 7.5 7.0 6.5 6.0 5.5 5.0 4.5 4.0 3.5 3.0 2.5 2.0 1.5 1.0 0.5 0.0 -0.5 -1
f1 (ppm)

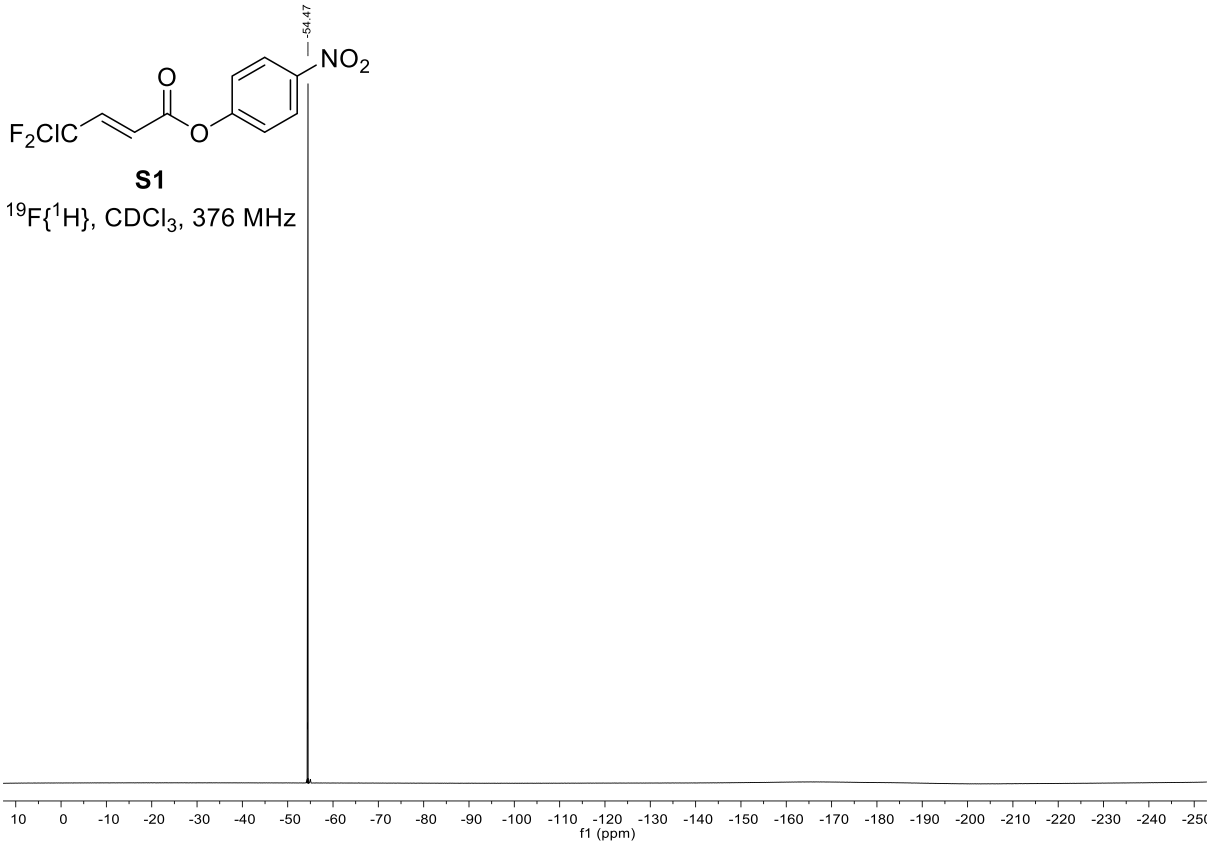
-54.47
NO2
O
F2ClC
O
S1
19F{1H}, CDCl3, 376 MHz
10 0 -10 -20 -30 -40 -50 -60 -70 -80 -90 -100 -110 -120 -130 -140 -150 -160 -170 -180 -190 -200 -210 -220 -230 -240 -250
f1 (ppm)

4-Nitrophenyl (*E*)-4-bromo-4,4-difluorobut-2-enoate (S2)

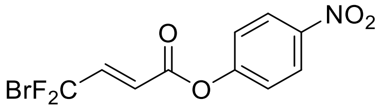

The title compound was prepared according to **General Procedure 1**, (*E*)-4-bromo-4,4-difluorobut-2-enoic acid (200 mg, 1.0 mmol) in anhydrous CH_2Cl_2 (0.1 M) was added *N,N'*-dicyclohexylcarbodiimide (226.6 mg, 1.1 mmol) at room temperature and stirred for 0.5 h. 4-Nitrophenol (139 mg, 1 mmol) were added and the mixture was stirred for 3 h. The reaction was then filtered, and the solvent was removed under reduced pressure. The residue was purified by column chromatography (2:1 Hexane:EtOAc) to give the title compound (183.5 mg, 57%) as a white solid. **mp** 77–79 ºC (lit.[50] 79-80 ºC); **IR** $\mathbf{\nu_{max}}$ (film) / cm^{-1} 3080, 1740, 1593, 1524, 1487, 1348, 1312, 1279, 1204, 1161, 1105, 972, 932, 858, 793; $\mathbf{^1H}$ **NMR** (400 MHz, $CDCl_3$) δ_H: 6.51 (1H, dt, *J* 15.5, 1.7, $BrF_2CCH=CH$), 7.21 (1H, dt, *J* 15.5, 9.8, 9.8, $BrF_2CCH=CH$), 7.34-7.38 (2H, m, $ArC^{2,6}H$), 8.29-8.34 (2H, m, $ArC^{3,5}H$); $\mathbf{^{19}F\{^1H\}}$ **NMR** (376 MHz, $CDCl_3$) δ_F: –50.65 (CF_2Br). Data in accordance with literature.[50]

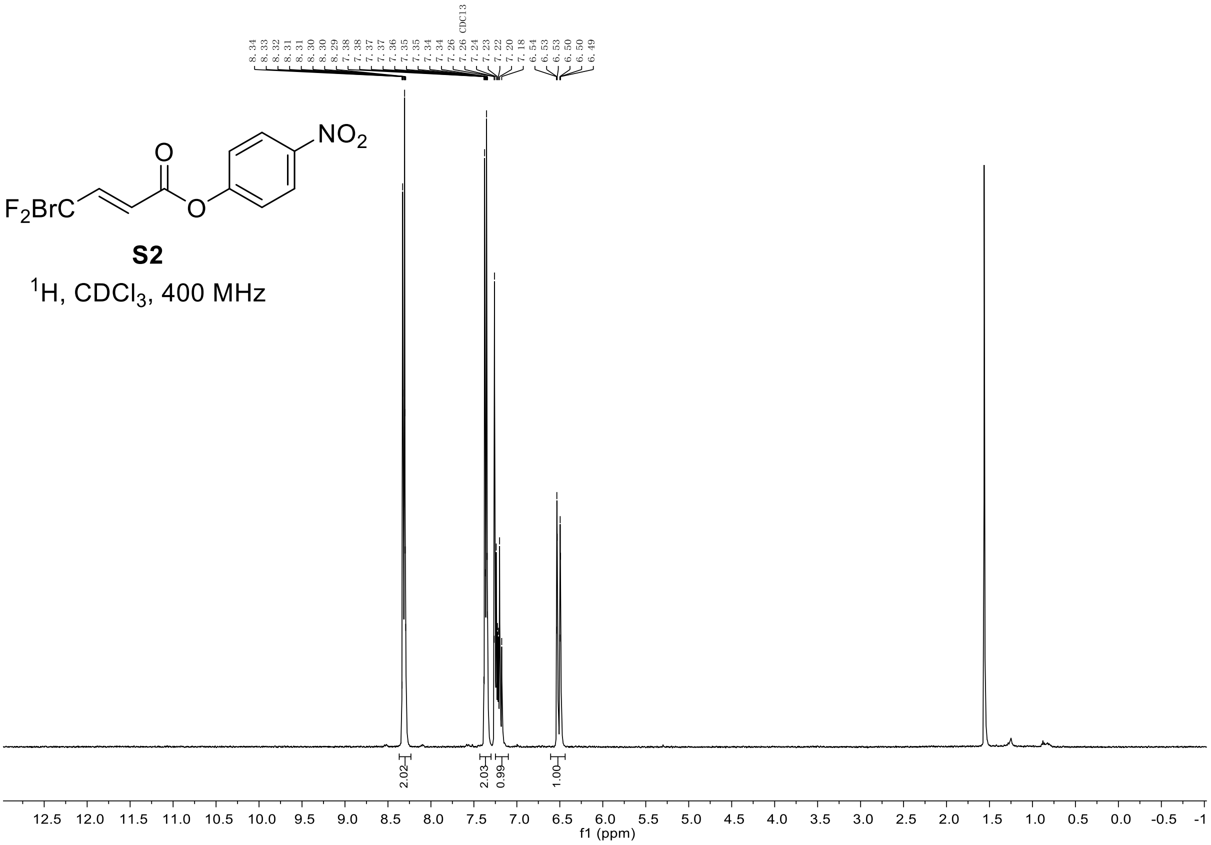

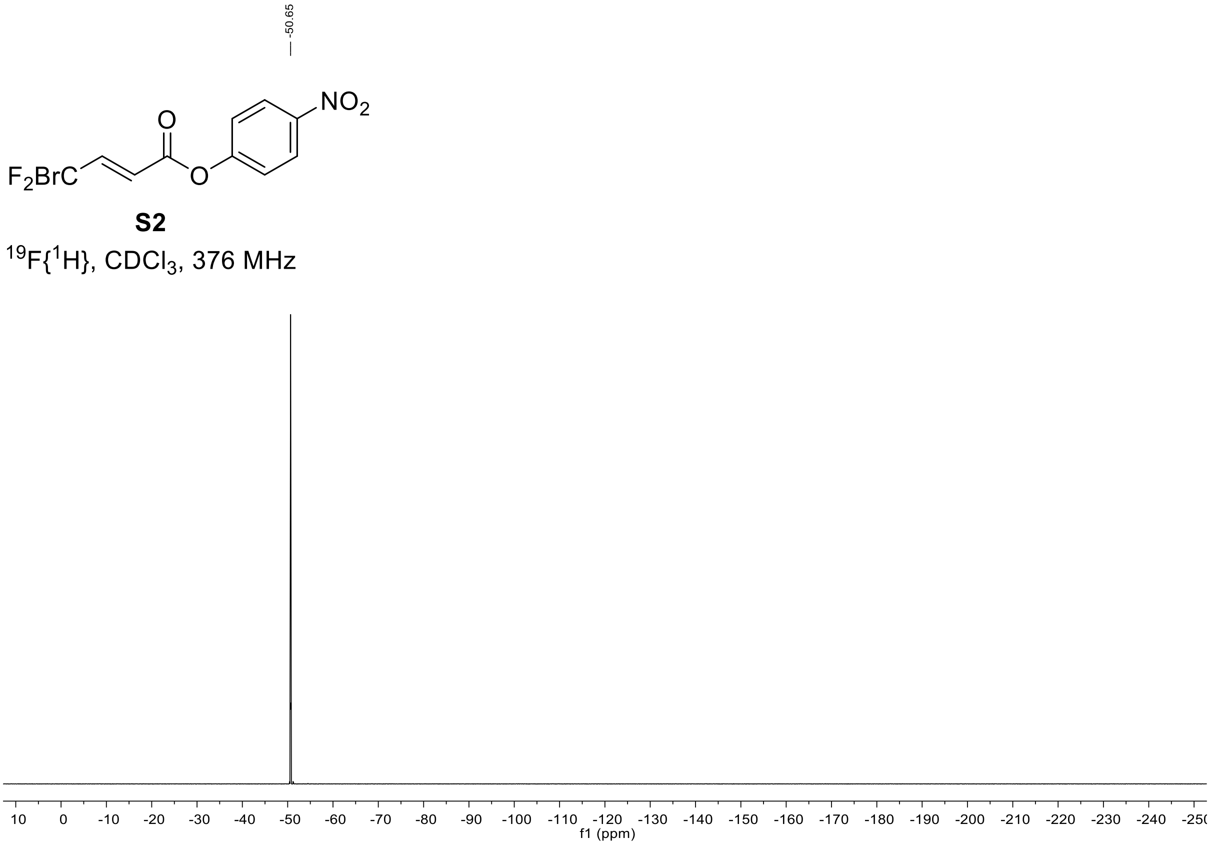

4-Nitrophenyl (*E*)-4,4,5,5,5-pentafluoropent-2-enoate (S3)

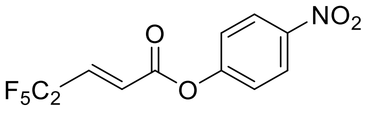

The title compound was prepared according to the literature,[52] (*E*)-4,4,5,5,5-pentafluoropent-2-enoic acid (190 mg, 1.0 mmol) in anhydrous CH_2Cl_2 (0.33 M) was added oxalyl chloride (127 mg, 1.0 mmol) and a few drops of DMF at room temperature under a N_2 atmosphere and allowed to stir for 1 h. A solution of diisopropylethylamine (258 mg, 2.0 mmol) and 4-nitrophenol (139 mg, 1.0 mmol) in anhydrous CH_2Cl_2 (0.33 M) were added dropwise and the mixture was allowed to stir overnight. The solvent was removed *in vacuo* and the residue was purified by column chromatography (4:1 Petrol:EtOAc) to give the title compound (236.4 mg, 76%) as a colourless oil. **IR** $\mathbf{\nu_{max}}$ (film) / cm^{-1} 3119, 3082, 3059, 1744, 1593, 1526, 1348, 1308, 1279, 1192, 1125, 1040, 970, 858, 823; **^{1}H NMR** (400 MHz, $CDCl_3$) δ_H: 6.78 (1H, dt, *J* 15.9, 2.1, $F_5C_2CH{=}C\mathit{H}$), 7.04 (1H, dtq, *J* 15.8, 11.5, 0.8, $F_5C_2C\mathit{H}{=}CH$), 7.35-7.39 (2H, m, $ArC^{2,6}\mathit{H}$), 8.29-8.33 (2H, m, $ArC^{3,5}\mathit{H}$); **^{19}F{^{1}H} NMR** (376 MHz, $CDCl_3$) δ_F: –117.39 (2F, d, *J* 2.3, $C\mathit{F}_2$), –84.42 (3F, t, *J* 3.2, $C\mathit{F}_3$). Data in accordance with literature.[52]

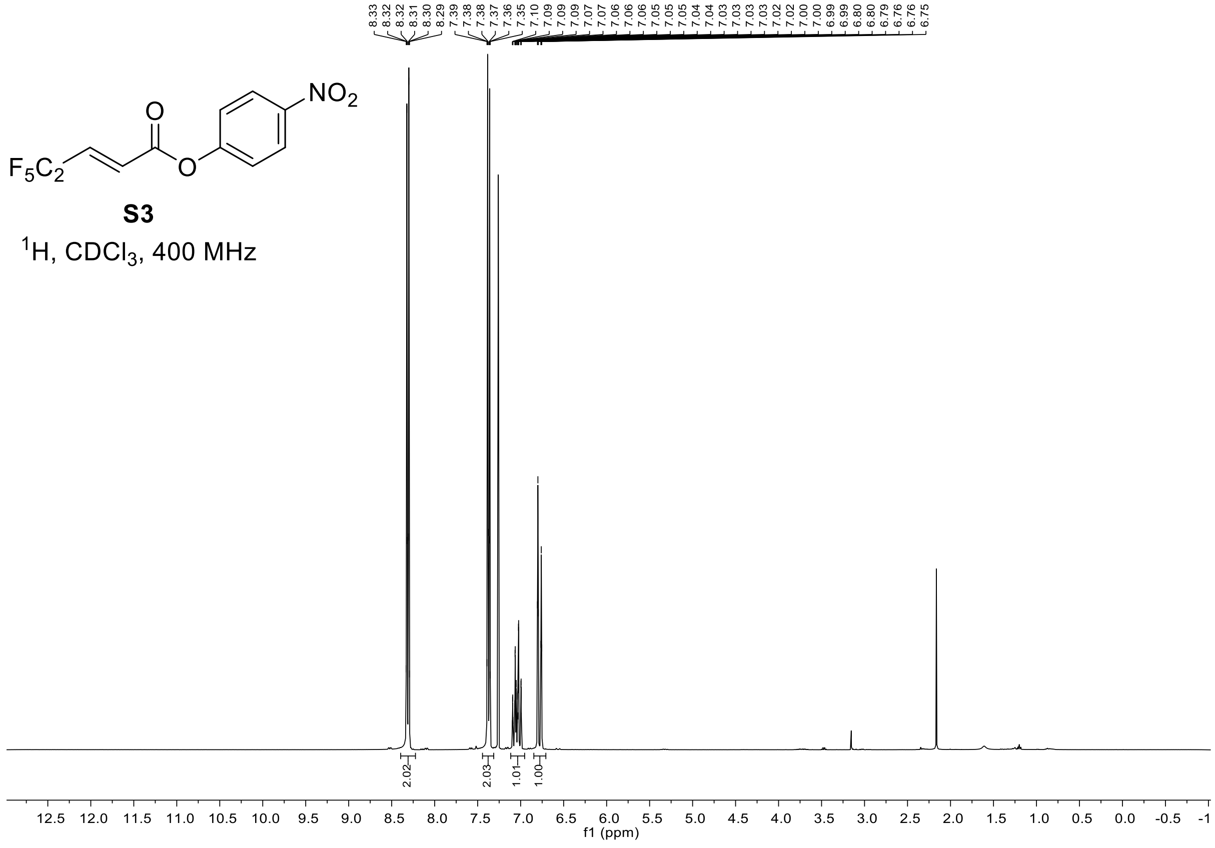
NO2
F5C2
O
O
S3
1H, CDCl3, 400 MHz
f1 (ppm)

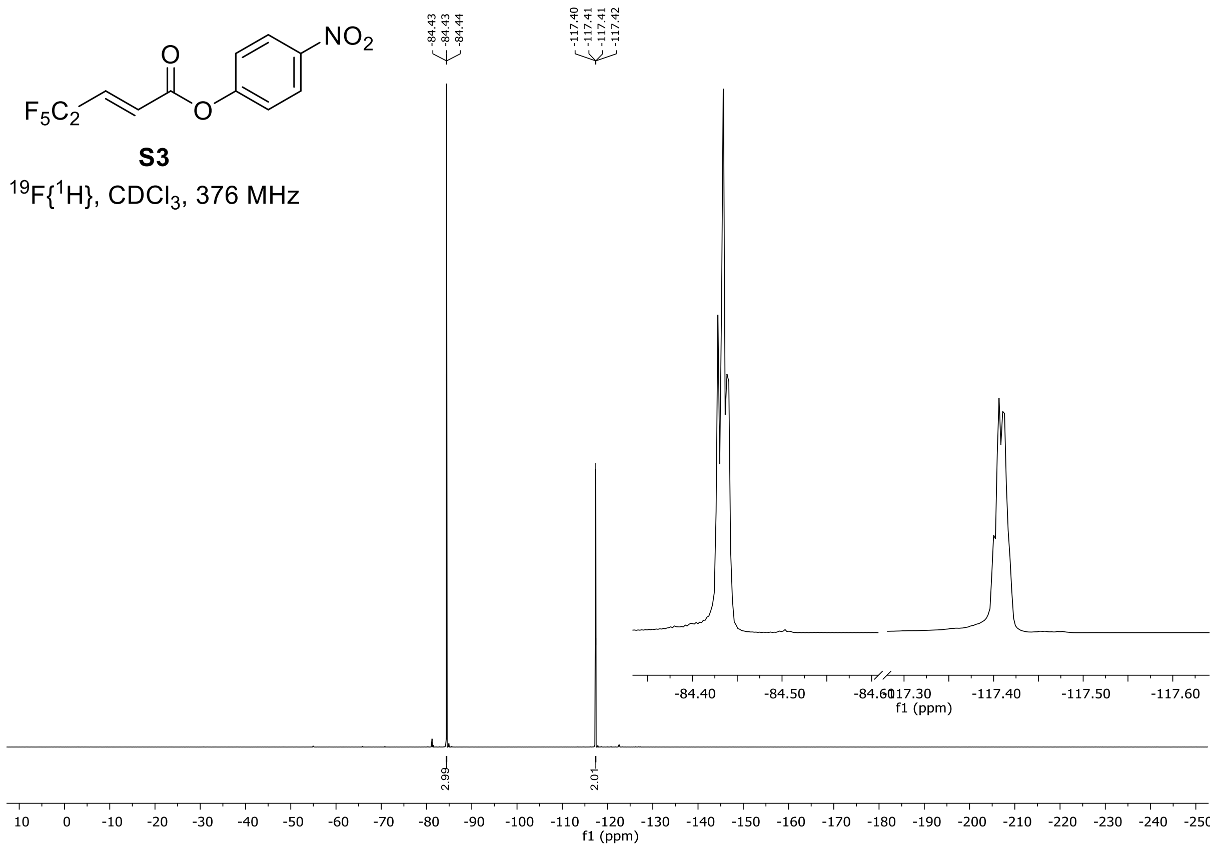
NO2
F5C2
O
O
S3
19F{1H}, CDCl3, 376 MHz
f1 (ppm)

4-Nitrophenyl (*E*)-4,4-difluorobut-2-enoate (S4)

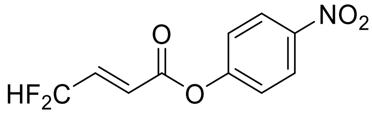

The title compound was prepared according to literature,[52] (*E*)-4,4-difluorobut-2-enoic acid (122 mg, 1.0 mmol) in anhydrous CH_2Cl_2 (0.33 M) was added 4-nitrophenol (208.5 mg, 1.5 mmol), EDCI (38.5 mg, 2.0 mmol), DMAP (6.7 mg, 0.1 mmol) and stirred for 16 h. The solvent was then removed under reduced pressure and the residue was purified by column chromatography (4:1 Petrol:EtOAc) to give the title compound (102.7 mg, 32%) as a white solid. **mp** 74–76 ºC (lit.[50] 74–76 ºC); **IR** ν_{max} (film) / cm^{-1} 3119, 3080, 1736, 1530, 1391, 1344, 1169, 1036, 964, 856, 812; **^{1}H NMR** (400 MHz, $CDCl_3$) δ_H: 6.34 (1H, tdd, *J* 54.5, 3.8, 1.1, CF_2H), 6.52 (1H, dtd, *J* 15.9, 2.9, 1.1, $F_2CHCH{=}CH$), 7.06 (1H, dtd, *J* 15.9, 10.3, 3.8, $F_2CHCH{=}CH$), 7.33-7.37 (1H, m, ArC^{2,6}*H*), 8.29-8.33 (2H, m, ArC^{3,5}*H*); **^{19}F{^{1}H} NMR** (376 MHz, $CDCl_3$) δ_F: –116.91 (CHF_2). Data in accordance with literature.[52]

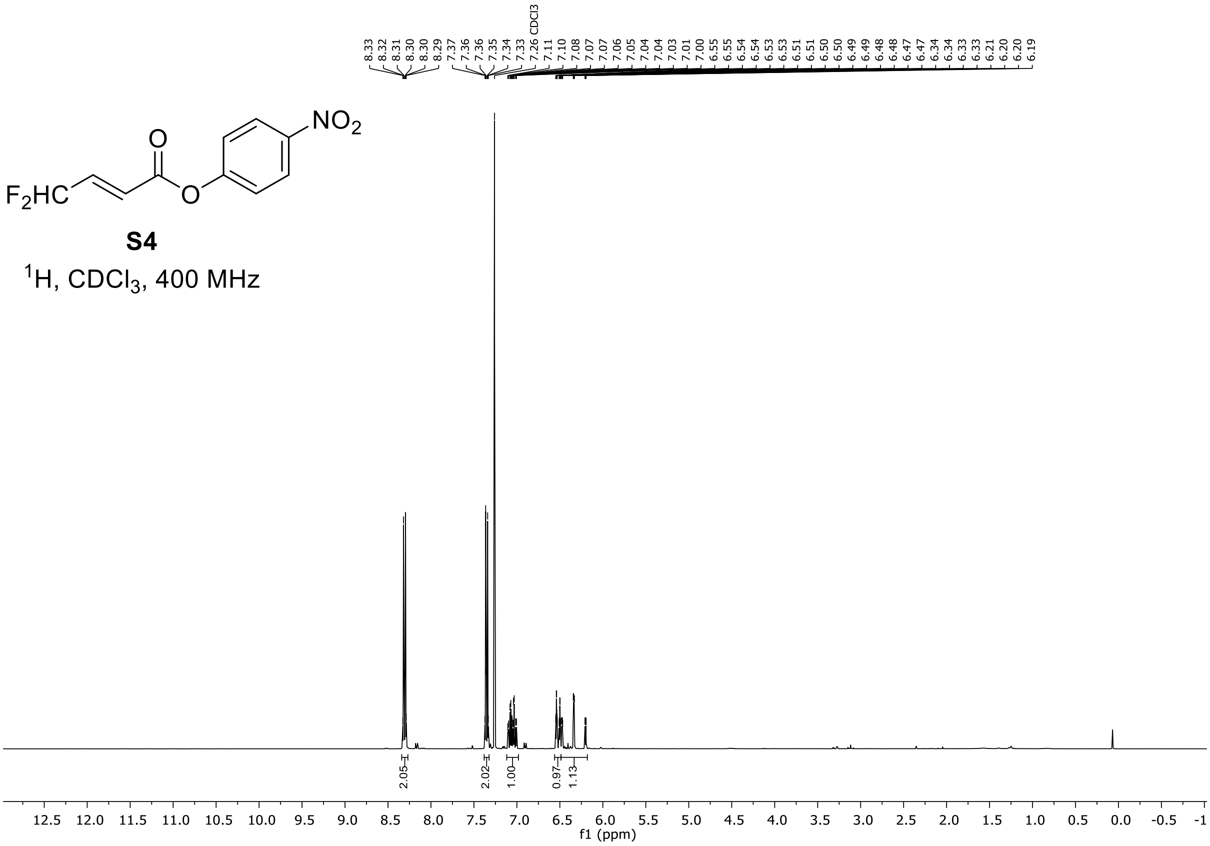

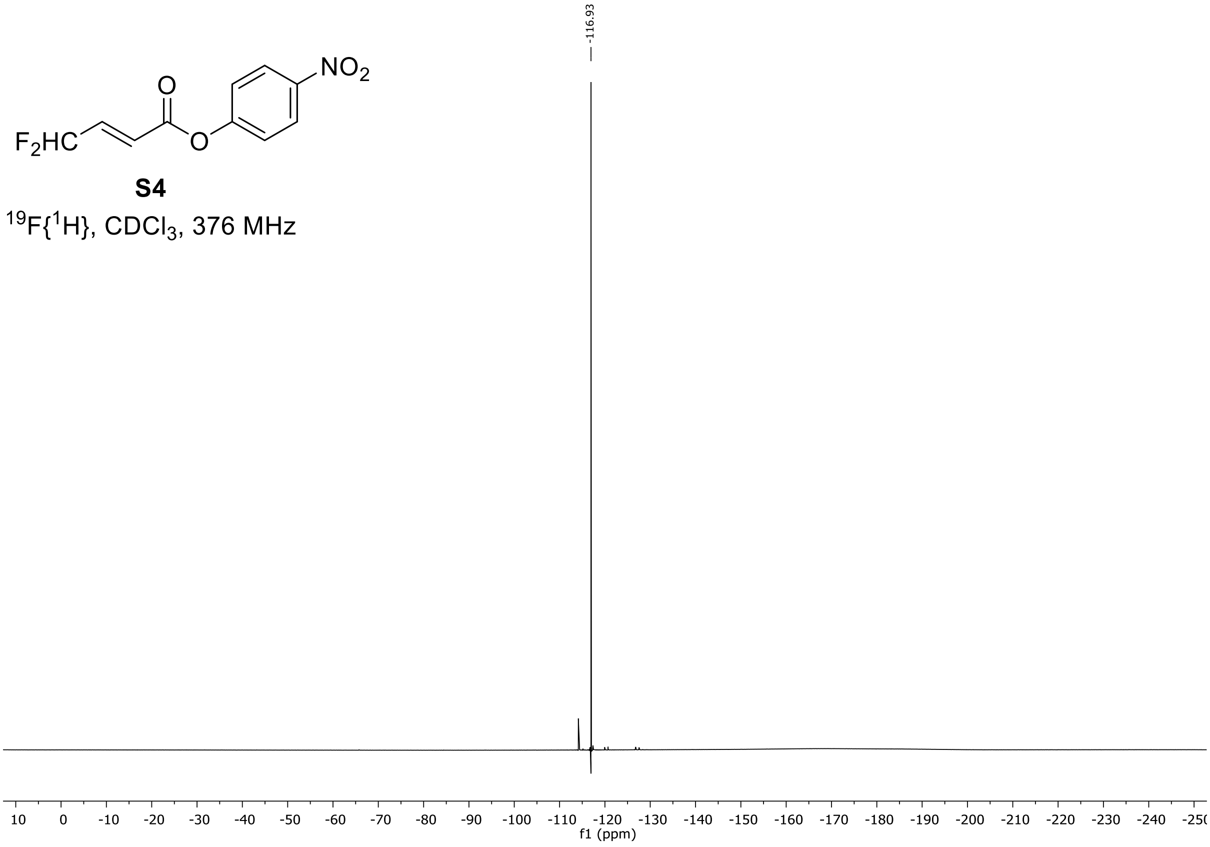

Synthesis of β-Ketoesters

(±)-Methyl 1-acetyl-3-oxoindoline-2-carboxylate (18)

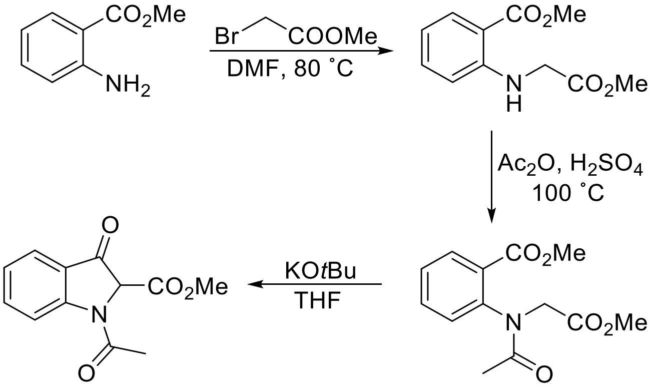

The title compound was prepared according to literature.[53] To methyl 2-aminobenzoate (4.00 g, 26.4 mmol) in DMF was added methyl bromoacetate(2.02 g, 13.2 mmol) at room temperature and the mixture was stirred at 80 ºC for 12 h. The mixture was poured into water and extracted with Et_2O and washed sequentially with water and brine, then dried over $MgSO_4$ and filtered. The solvent was removed under reduced pressure to give methyl 2-((2-methoxy-2-oxoethyl)amino)benzoate (5.17 g, 86%) as a yellow solid.

Methyl 2-((2-methoxy-2-oxoethyl)amino)benzoate (5.00 g, 22.4 mmol) was added to acetic anhydride/sulfuric acid (25 mL and a catalytic amount of conc. H_2SO_4 (2 drops)) and heated to 100 ºC for 5 h. The mixture was washed with saturated aqueous $NaHCO_3$. The solvent was reduced in *vacuo*

and purified by column chromatography (1:1 Petrol:EtOAc) to give methyl 2-(*N*-(2-methoxy-2-oxoethyl)acetamido)benzoate (5.46 g, 89%) as a yellow oil.

Methyl 2-(*N*-(2-methoxy-2-oxoethyl)acetamido)benzoate (5.20 g, 19.6 mmol) and THF (40 mL) were added to a flask and stirred in ice bath under a N_2 atmosphere. KO^{t}Bu (2.86 g, 25.5 mmol) was added to the solution, and it was stirred for an additional hour in an ice bath. Acetic acid (1.46 mL, 1.3 equiv.) was added to adjust the pH to about 6. The mixture was extracted with CH_2Cl_2 and washed with brine. The solvent was removed under reduced pressure and the residue was purified by column chromatography (1:1 Petrol:EtOAc) to give the title compound as a white solid (3.29 g, 72%). The ratio of keto form to enol form in $CDCl_3$ is 74:26. **mp** 124–127 ºC; **IR ν$_{max}$** (film) / cm^{-1} 3036, 2978, 2953, 1755, 1680, 1462, 1383, 1342, 1294, 1244, 1144, 933, 802; **^{1}H NMR** (400 MHz, $CDCl_3$) δ$_H$: 2.20 (2.22H, s, C*H*$_3$ (keto)), 2.60 (0.78H, s, C*H*$_3$ (enol)), 3.86 (3H, s, OC*H*$_3$), 5.00 (1H, s, NC*H*), 7.21-7.77 (4H, m, ArC^{4,5,6,7}*H*), 8.59 (0.74H, br, O*H* (enol)). Data in accordance with literature.[53]

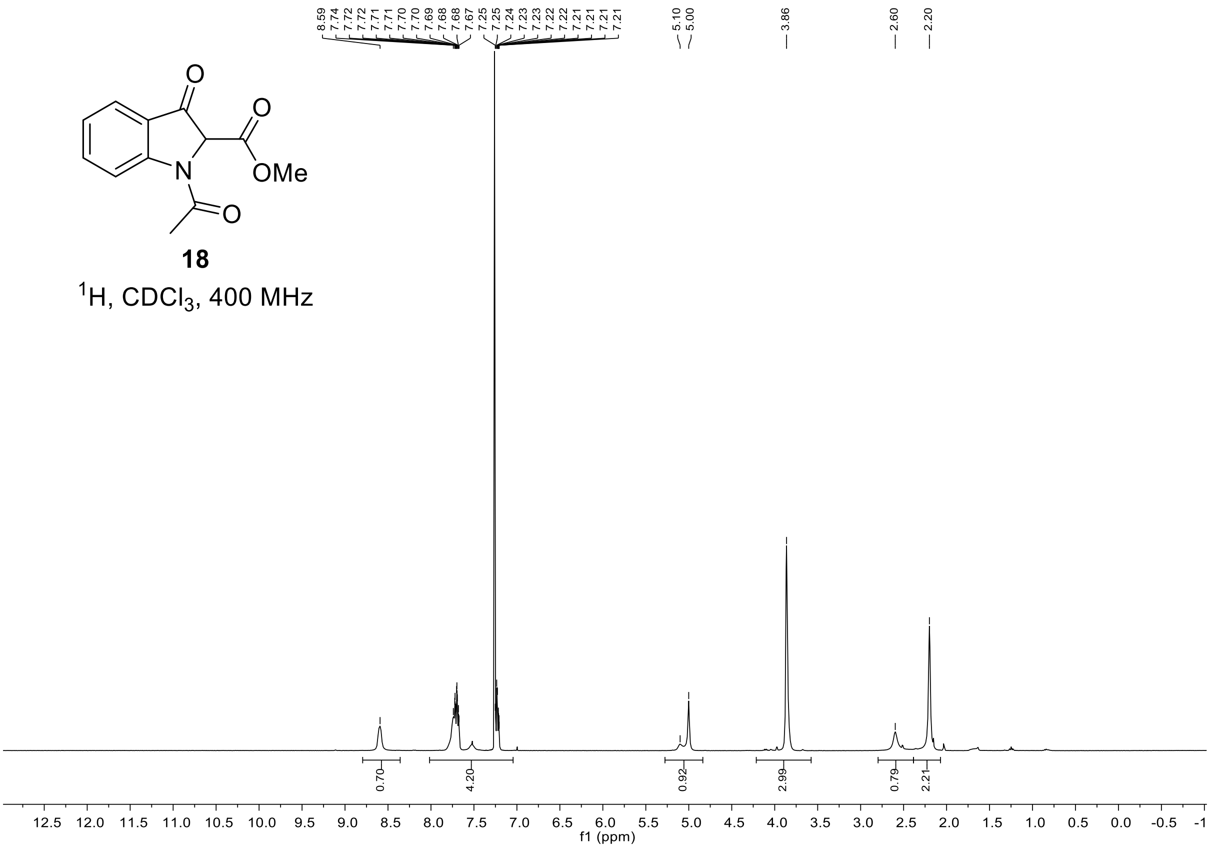

(±)-Ethyl 1-oxo-2,3-dihydro-1*H*-indene-2-carboxylate (S5)

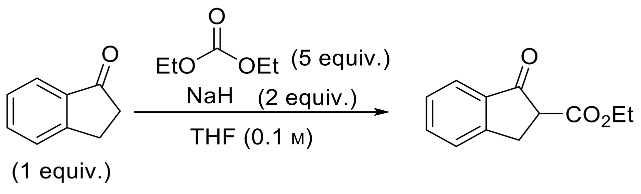

The title compound was prepared according to literature,[54] 2,3-dihydro-1*H*-inden-1-one (132 mg, 1.0 mmol) in anhydrous THF (0.1 M) was added diethyl carbonate (590 mg, 5.0 mmol) and NaH (48 mg, 2.0 mmol) and heated to reflux for 4 h. The mixture was poured into water and extracted with EtOAc. The solvent was removed under reduced pressure and the residue was purified by column chromatography (3:1 Petrol:EtOAc) to give the title compound (144.8 mg, 71%) as a brown oil. The ratio of the keto form to the enol form in $CDCl_3$ is 85:15. **IR ν_{max}** (film) / cm^{-1} 2980, 2934, 1736, 1709, 1464, 1254, 1207, 1013, 858; **^{1}H NMR** (400 MHz, $CDCl_3$, 86:14 mixture of keto : enol form) δ_H: 1.31 (2.58H, t, *J* 7.1, OCH_2CH_3), 1.37 (0.42H, t, *J* 7.1, OCH_2CH_3), 3.37 (0.86H, dd, *J* 17.2, 8.3, $ArCH_AH_BCH$), 3.52 (0.28H, s, $ArCH_2$, enol), 3.56 (0.86H, dd, *J* 17.2, 4.0, $ArCH_AH_BCH$), 3.71 (0.86H, dd, J 8.3, 4.0, $ArCH_2CH$), 4.24 (0.86H, dq, *J* 10.8, 7.1, $OCH_AH_BCH_3$), 4.26 (0.86H, dq, *J* 10.8, 7.1, $OCH_AH_BCH_3$), 4.32 (0.28H, *J* 7.1, OCH_2CH_3), 7.36-7.40 (0.14H, m, ArC^5H), 7.39 (0.86H, app tq, J 7.5, 0.8, ArC^5H), 7.42 (0.14H, app td, *J* 7.3, 1.4, ArC^6H), 7.47 (0.14H, app ddq, *J* 7.4, 1.4, 0.7, ArC^4H), 7.50 (0.86H, app dp, *J* 7.6, 0.9, ArC^4H), 7.62 (0.86H, app td, *J* 7.5, 1.2, ArC^6H), 7.64 (0.14H, ddd, *J* 7.6, 1.4, 0.9, ArC^7H), 7.77 (1H, ddd, J 7.6, 1.3, 0.8, ArC^7H). Data in accordance with literature.[54]

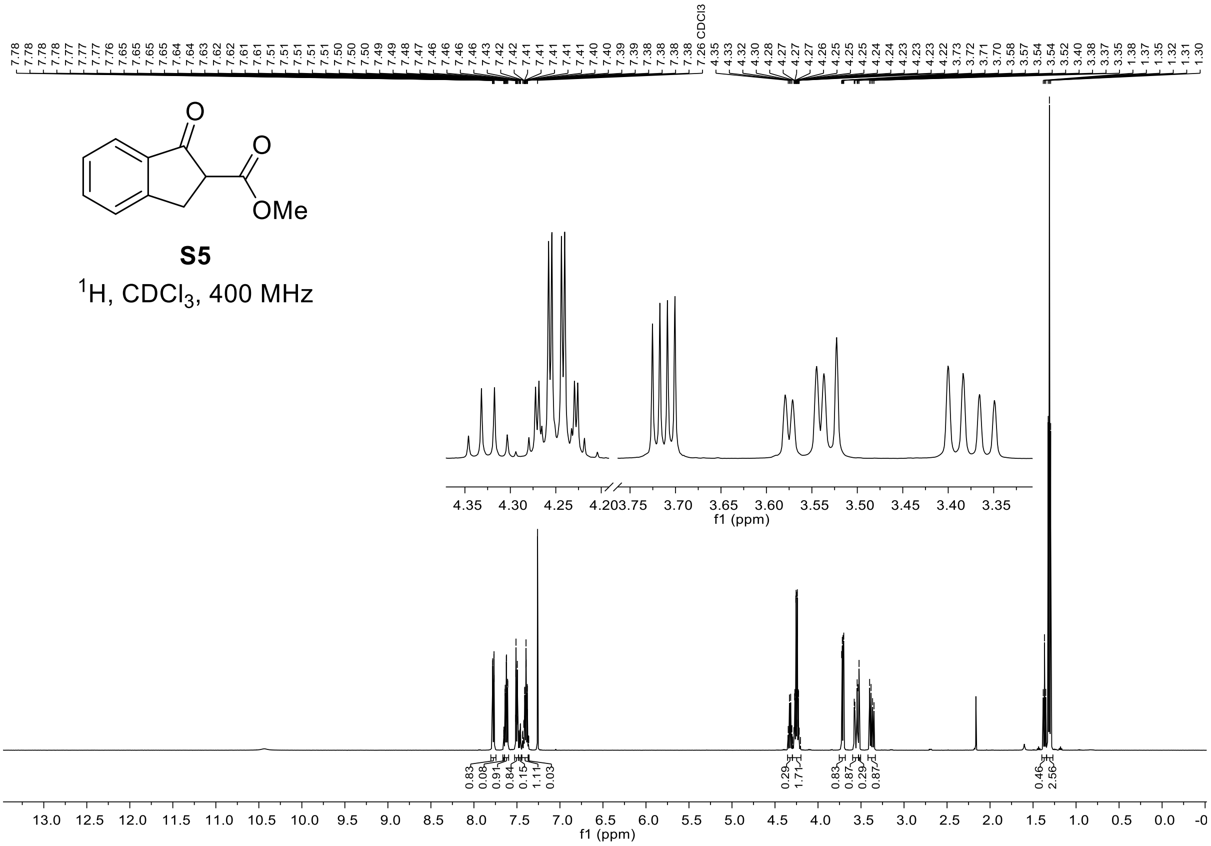

(±)-Ethyl 3-oxo-2,3-dihydrobenzofuran-2-carboxylate (S6)

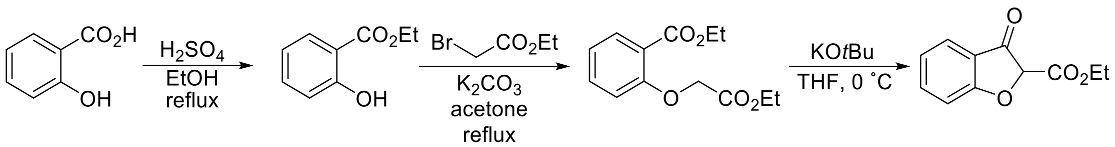

The title compound was prepared according to literature.[55] To a solution of 2-hydroxybenzoic acid (500 mg, 3.6 mmol) in anhydrous EtOH (15 mL) was added concentrated H_2SO_4 (0.4 mL). The reaction was stirred under refluxed for 5 h. The mixture was poured into water and extracted with CH_2Cl_2. The organic extract was dried over $MgSO_4$ and filtered. The solvent was removed under reduced pressure and the residue was purified by column chromatography (3:1 Petrol:EtOAc) to give ethyl 2-hydroxybenzoate (0.6 g, 100%) as colourless oil.

A mixture of ethyl 2-hydroxybenzoate (0.6 g, 3.6 mmol), potassium carbonate (1.0 g, 0.73 mmol) and ethyl bromoacetate (0.48 mL, 4.3 mmol) in acetone (5 mL) was stirred at 60 °C for 3 h. The reaction was filtered and concentrated under reduced pressure to give ethyl 2-(2-ethoxy-2-oxoethoxy)benzoate (1.0 g, 95%) as orange oil.

Potassium *tert*-butoxide (1.0 g, 17.8 mmol) in anhydrous THF (15 mL) was added to a flame dried flask and placed in an ice bath under an N_2 atmosphere. A solution of ethyl 2-(2-ethoxy-2-oxoethoxy)benzoate in anhydrous THF (5 mL) was then added dropwise over 1 h. The mixture was quenched with saturated aqueous NH_4Cl and extracted with EtOAc. The organic extract was washed with saturated aqueous NH_4Cl, dried over $MgSO_4$ and filtered. The solvent was removed under reduced pressure and the residue was purified by column chromatography (3:1 Petrol:EtOAc) to give the title compound (1.29 g, 72%) as a white solid. **mp** 75–77 ºC; **IR** ν_{max} (film) / cm^{-1} 3345, 3075, 2967, 1736, 1680, 1609, 1584, 1466, 1441, 1263, 1134, 1016, 852; **^{1}H NMR** (400 MHz, $CDCl_3$) δ_H: 1.46 (3H, t, *J* 7.1, OCH_2CH_3), 4.48 (2H, q, *J* 7.2, OCH_2CH_3), 7.30 (1H, ddd, *J* 8.0, 6.0, 2.1, ArC^5H), 7.44 -7.53 (2H, m, $ArC^{6,7}H$), 7.74 (1H, app dt, *J* 8.0, 1.0, ArC^4H), 8.15 (1H, br, O*H*); Data in accordance with literature.[55]

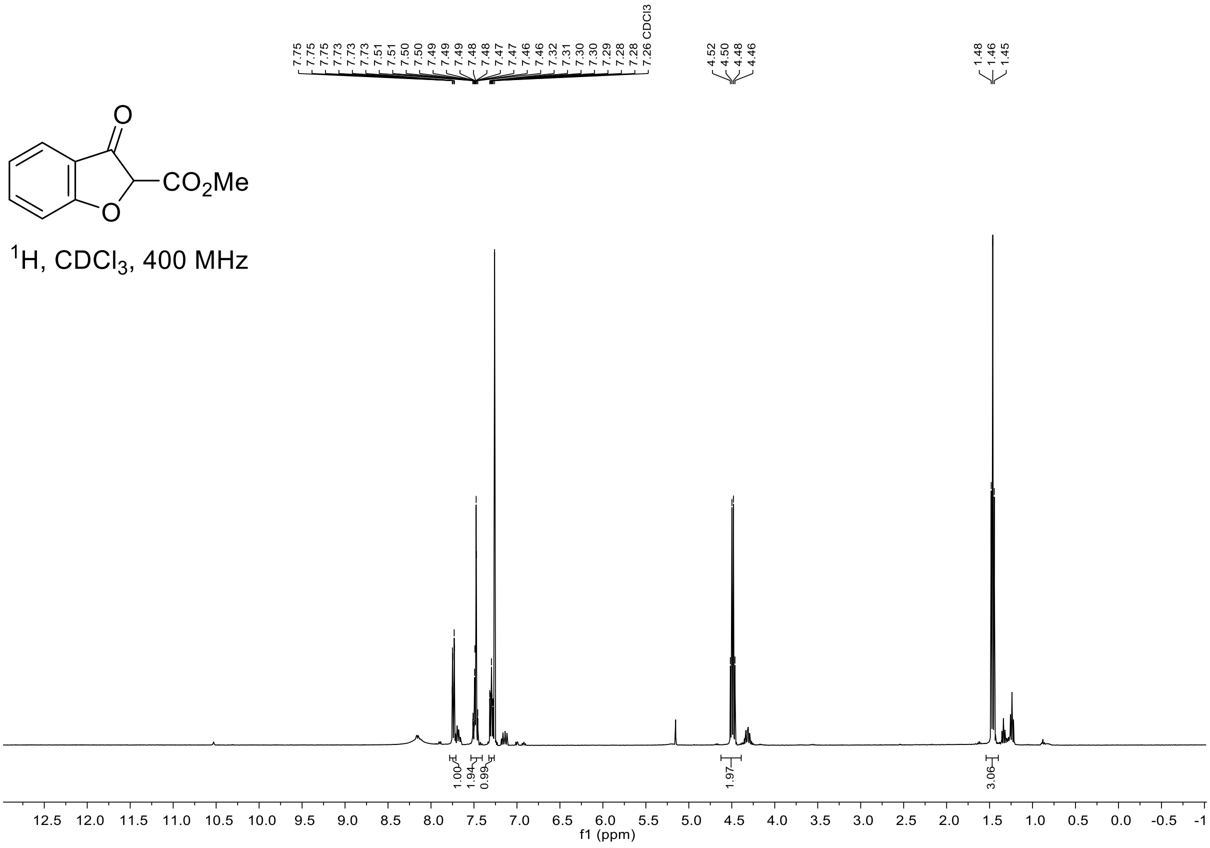

Synthesis of the Michael Addition Products

(+)-Ethyl (*S*)-1-((*S*)-4′-(benzylamino)-1′,1′,1′-trifluoro-4′-oxobutan-2′-yl)-2-oxocyclopentane-1-carboxylate (1*S*,2′*S*)-9

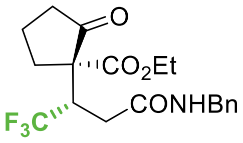

(1*S*,2'*S*)-**9**

Preparation from (*E*)-enoate:

The title compound was prepared according to **General Procedure 2**. A mixture of 4-nitrophenyl (*E*)-4,4,4-trifluorobut-2-enoate **8** (26.1 mg, 0.1 mmol), (2*S*,3*R*)-HyperBTM **1** (1.5 mg, 5 µmol) and methyl 2-oxocyclopentane-1-carboxylate (15 µL, 0.1 mmol) in DMA (0.4 mL) was stirred for 16 h at room temperature. Benzylamine (11 µL, 0.1 mmol) was then added, and the reaction was permitted to stir for 1 h. The solution was diluted with EtOAc, washed sequentially with saturated aqueous Na_2CO_3 (×3) and brine, then dried over $MgSO_4$ and filtered. The solvent was removed under reduced pressure and the residue was purified by column chromatography (1:4 EtOAc:Hexane) to give the title compound (23.2 mg, 59%, >95:5 d.r.) as a white solid.

Preparation from (*Z*)-enoate:

Following **General Procedure 2**, a mixture of 4-nitrophenyl (*Z*)-4,4,4-trifluorobut-2-enoate **25** (26.1 mg, 0.1 mmol), (2*S*,3*R*)-HyperBTM **1** (1.5 mg, 5 μmol) and methyl 2-oxocyclopentane-1-carboxylate **7** (15 μL, 0.1 mmol) in DMA (0.4 mL) was stirred for 16 h at room temperature. Benzylamine (11 μL, 0.1 mmol) was then added, and the reaction was permitted to stir for 1 h. The solution was diluted with Et_2O, washed sequentially with saturated aqueous Na_2CO_3 (×3) and brine, dried over $MgSO_4$ and filtered. The solvent was removed under reduced pressure and the residue was purified by column chromatography (1:4 EtOAc:Hexane) to give the title compound (15.7 mg, 40%, >95:5 d.r.) as a colourless oil.

Data for (1*S*,2'*S*)-**9**: **mp** 65-70 °C; $[\alpha]_D^{20}$+0.25 (*c* 0.8 in $CHCl_3$); **IR ν_{max}** (film) / cm^{-1} 3294, 1749, 1641, 1560, 1225, 1145, 1118, 989, 858; **^{1}H NMR** (500 MHz, $CDCl_3$) δ_H: 1.24 (3H, t, *J* 7.1, OCH_2CH_3), 1.91-2.00 (1H, m, $COCH_2CH_AH_B$), 2.00-2.08 (1H, m, $COCH_2CH_AH_B$), 2.13 (1H, dddd, *J* 13.5, 9.3, 7.3, 1.1, $CCH_AH_BCH_2$), 2.31-2.44 (2H, m, $COCH_2CH_2$), 2.50 (1H, dd, *J* 16.1, 7.1, $CHCH_AH_BCONH$), 2.67 (1H, ddd, *J* 13.5, 7.1, 4.2, $CCH_AH_BCH_2$), 2.75 (1H, dd, *J* 16.1, 4.3, $CHCH_AH_BCONH$), 3.76 (1H, qdd, *J* 9.4, 7.1, 4.3, $CHCH_2CONH$), 4.14 (2H, q, *J* 7.1, OCH_2CH_3), 4.40 (1H, dd, *J* 14.7, 5.6, $NHCH_AH_BPh$), 4.46 (1H, dd, 14.7, 5.8, $NHCH_AH_BPh$), 5.96 (1H, app t (br), *J* 5.8, 5.6, N*H*), 7.25-7.30 (3H, m, $PhC^{2,4,6}H$), 7.30-7.35 (2H, $PhC^{3,5}H$); **^{13}C{^{1}H} NMR** (126 MHz, $CDCl_3$) δ_C: 14.0 (OCH_2CH_3), 19.5 ($COCH_2CH_2$), 30.6 (CCH_2CH_2), 33.1 ($CHCH_2CONH$), 37.6 ($COCH_2CH_2$), 43.6 (q, *J* 25.9, $CHCH_2CONH$), 44.0 ($NHCH_2Ph$), 60.5 (CCH_2CH_2), 62.6 (OCH_2CH_3), 126.8 (q, *J* 282.2, CF_3), 127.7 (PhC^4H), 127.9 ($PhC^{2,6}H$), 128.8 ($PhC^{3,5}H$), 138.1 (PhC^1), 168.6 (*C*OOEt), 169.3 (*C*ONH), 211.0 ($COCH_2CH_2$); **^{19}F NMR** (471 MHz, $CDCl_3$) δ_F: −63.66 (d, *J* 9.4, CF_3); **HRMS** (ESI^+) $C_{19}H_{23}F_3O_4N$ $[M+H]^+$ found 386.1563, requires 386.1579 (–2.8 ppm).

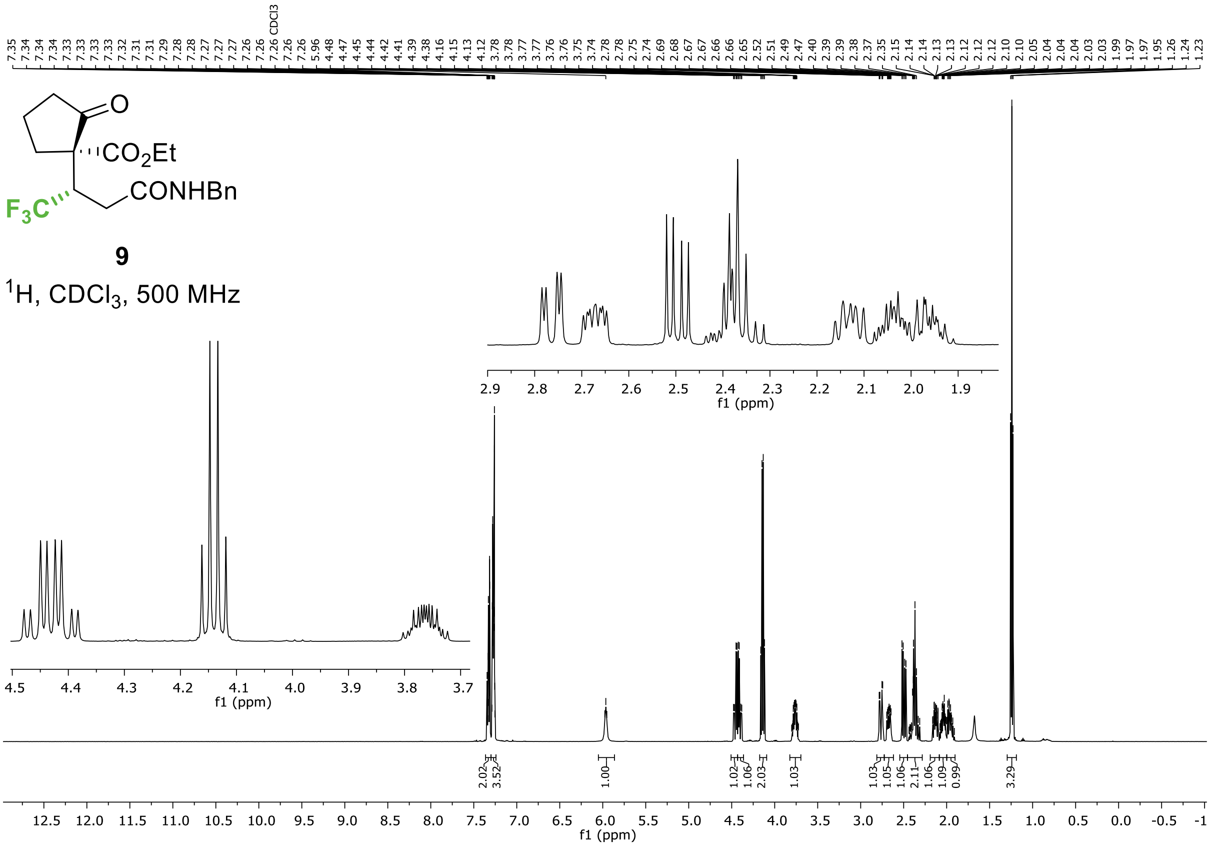

O
CO2Et
CONHBn
F3C
9
1H, CDCl3, 500 MHz
f1 (ppm)

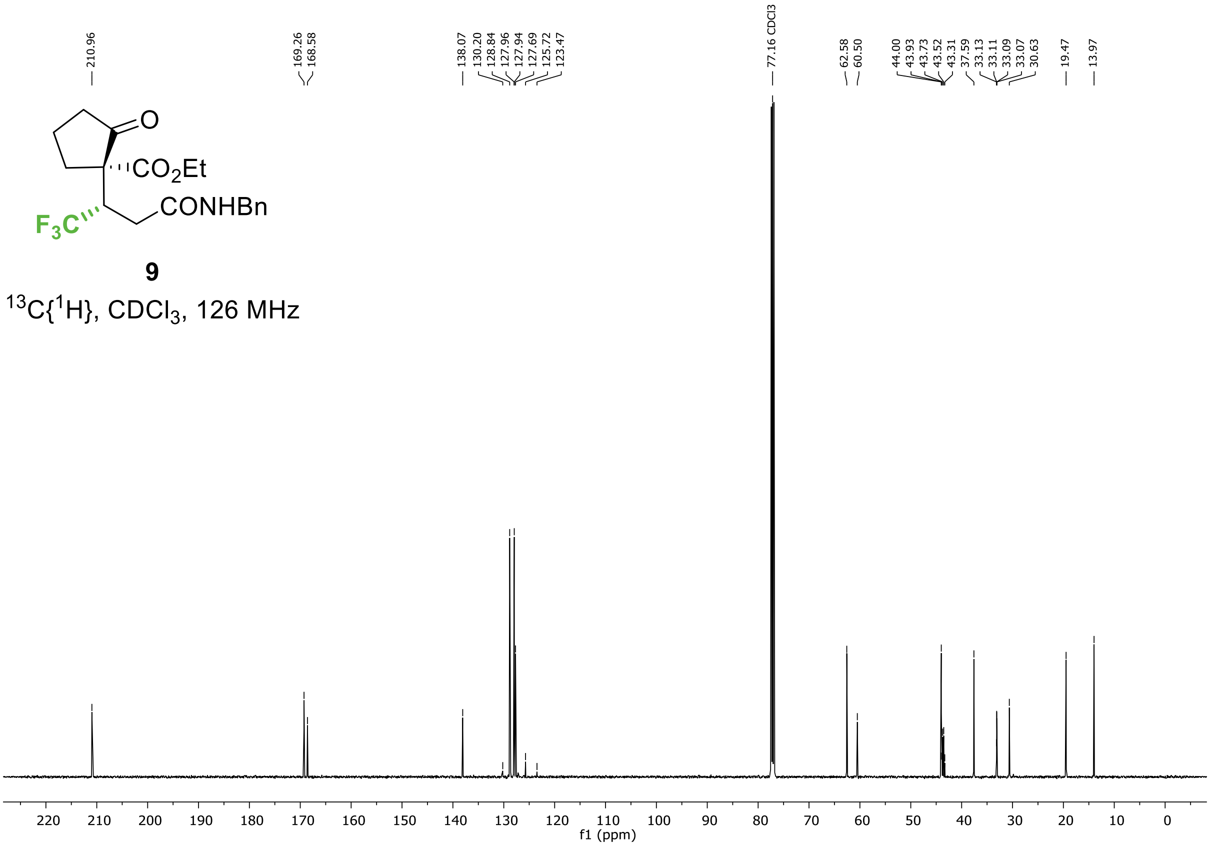

210.96
169.26
168.58
138.07
130.20
128.84
127.96
127.94
127.69
125.72
123.47
77.16 CDCl3
62.58
60.50
44.00
43.93
43.73
43.52
43.31
37.59
33.13
33.11
33.09
33.07
30.63
19.47
13.97
O
CO2Et
CONHBn
F3C
9
13C{1H}, CDCl3, 126 MHz
f1 (ppm)

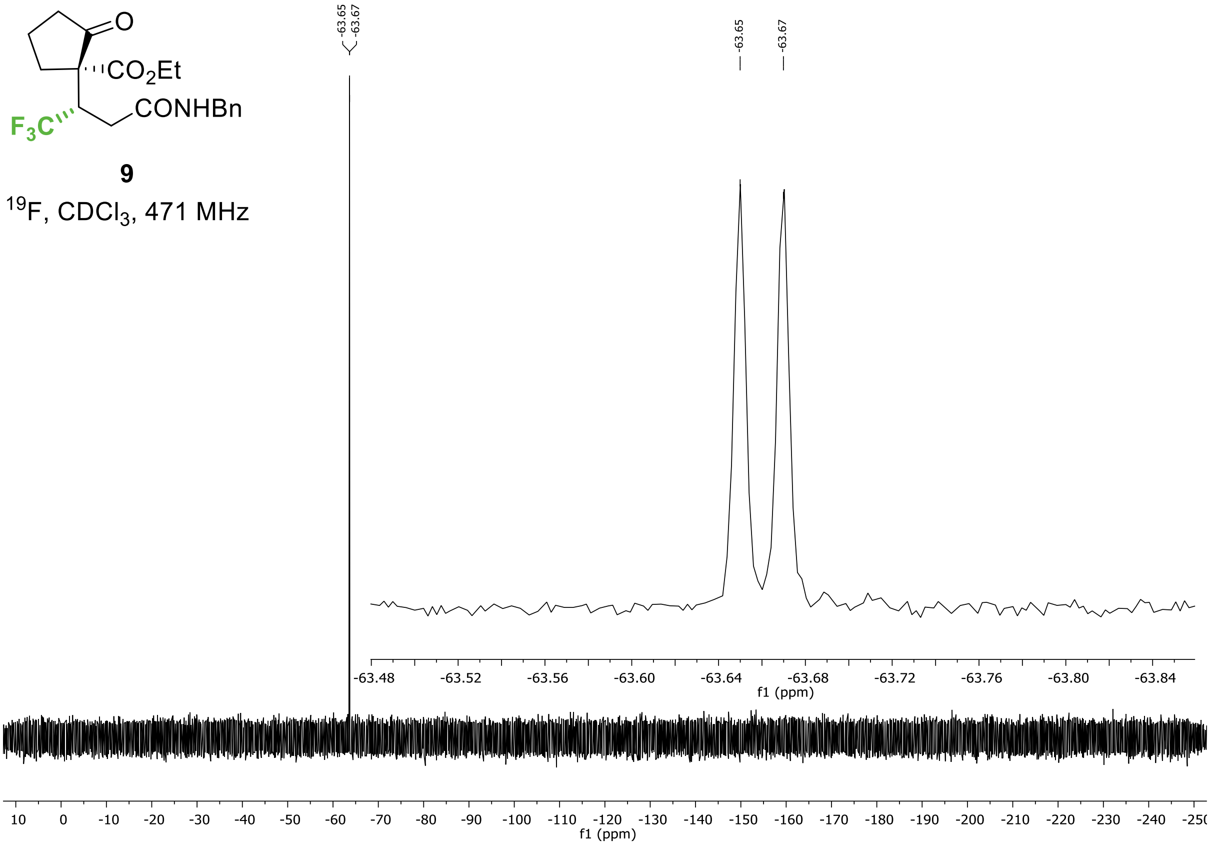

HPLC Data for 9 from (*E*)-enoate: Chiralpak AD-H (95:5 hexane:^{i}PrOH, flow rate 1.00 mL·min^{-1}, 211 nm, 30 °C) t_R (1*S*,2'*S*): 21.2 min, t_R (1*R*,2'*R*): 27.9 min, 99:1 er.

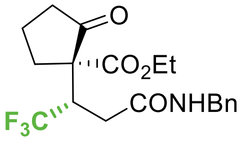

(1*S*,2'*S*)-**9**

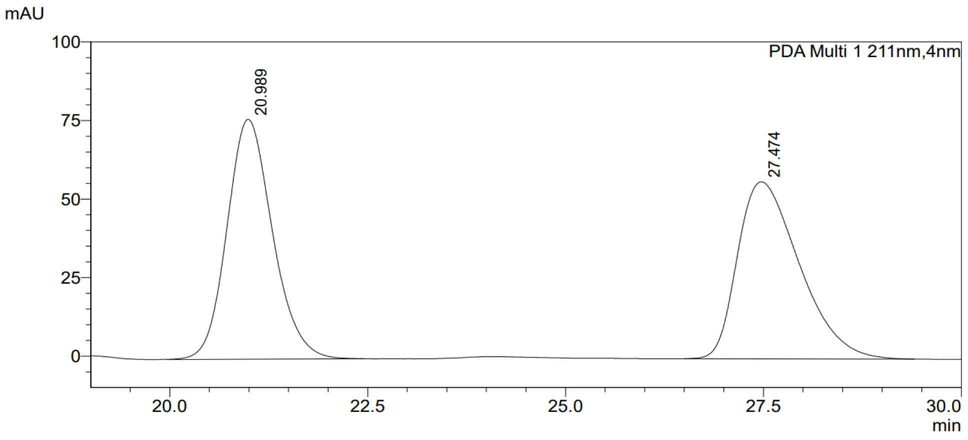

<Peak Table>

PDA Ch1 211nm

Peak#	Ret. Time	Area%
1	20.989	49.968
2	27.474	50.032
Total		100.000

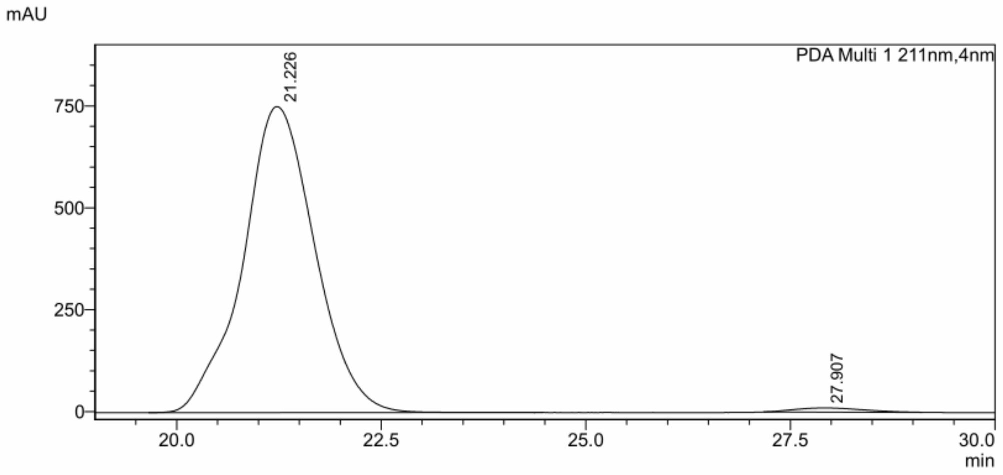

<Peak Table>

PDA Ch1 211nm

Peak#	Ret. Time	Area%
1	21.226	98.742
2	27.907	1.258
Total		100.000

HPLC Data for 9 from (*Z*)-enoate: Chiralpak AD-H (95:5 hexane:^{i}PrOH, flow rate 1.00 mL·min^{-1}, 211 nm, 30 °C) t_R (1*S*,2'*S*): 21.3 min, t_R (1*R*,2'*R*): 27.8 min, 82:18 er.

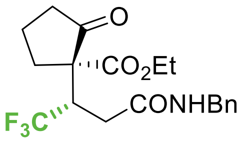

(1*S*,2'*S*)-**9**

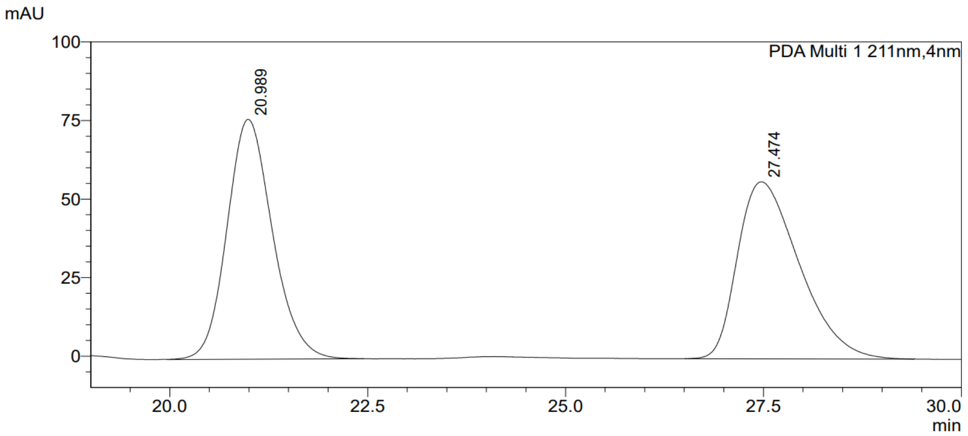

<Peak Table>

PDA Ch1 211nm

Peak#	Ret. Time	Area%
1	20.989	49.968
2	27.474	50.032
Total		100.000

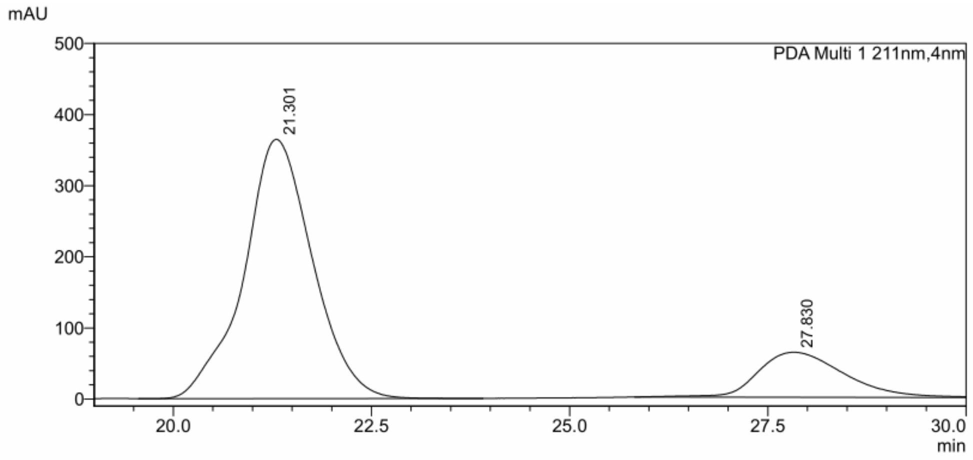

<Peak Table>

PDA Ch1 211nm

Peak#	Ret. Time	Area%
1	21.301	82.106
2	27.830	17.894
Total		100.000

(+)-Ethyl (*S*)-1-((*S*)-4'-(benzylamino)-1'-chloro-1',1'-difluoro-4'-oxobutan-2'-yl)-2-oxocyclopentane-1-carboxylate (1*S*,2'*S*)-10

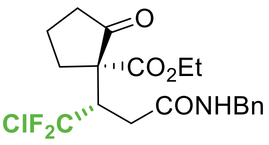

(1*S*,2'*S*)-**10**

The title compound was prepared according to **General Procedure 2**. A mixture of 4-nitrophenyl (*E*)-4-chloro-4,4-difluorobut-2-enoate **S1** (27.7 mg, 0.1 mmol), (2*S*,3*R*)-HyperBTM **1** (1.5 mg, 5 µmol) and

methyl 2-oxocyclopentane-1-carboxylate **7** (15 μL, 0.1 mmol) in DMA (0.4 mL) was stirred for 16 h at room temperature. Benzylamine (11 μL, 0.1 mmol) was added, and the reaction was permitted to stir for 1 h. The solution was diluted with EtOAc, washed sequentially with saturated aqueous Na_2CO_3 (×3), and brine, dried over $MgSO_4$ and filtered. The EtOAc was removed under reduced pressure and the residue was purified by column chromatography (1:4 EtOAc:Hexane) to give the title compound (14.7 mg, 36%, >95:5 d.r.) as a white solid.

Data for (1*S*,2'*S*)-**10**: **mp** 48-52 ºC; $[\alpha]_D^{20}$ +2.41 (*c* 1.16 in $CHCl_3$); **IR** ν_{max} (film) / cm^{-1} 3292, 2965, 1726, 1651, 1182, 1105, 957, 908, 868; **^{1}H NMR** (500 MHz, $CDCl_3$) δ_H: 1.23 (3H, t, *J* 7.1, OCH_2CH_3), 1.85-1.97 (1H, m, $COCH_2CH_AH_B$), 2.04 (1H, app dtdd, *J* 13.3, 7.2, 5.9, 4.1, $COCH_2CH_AH_B$), 2.19 (1H, app dddt, *J* 13.8, 9.6, 7.2, 1.2, $CCH_AH_BCH_2$), 2.30-2.43 (2H, m, $COCH_2CH_2$), 2.61 (1H, dd, *J* 16.5, 5.7, $CHCH_AH_BCONH$), 2.70 (1H, ddd, *J* 13.8, 7.2, 4.2, $CCH_AH_BCH_2$), 2.86 (1H, dd, *J* 16.5, 4.8, $CHCH_AH_BCONH$), 3.95 (1H, dddd, *J* 19.0, 5.7, 4.9, 4.1, $CHCH_2CONH$), 4.09 (1H, dq, *J* 10.5, 7.1, $OCH_AH_BCH_3$), 4.13 (1H, dq, *J* 10.5, 7.1, $OCH_AH_BCH_3$), 4.42 (1H, dd, J 14.6, 5.7, $NHCH_AH_BPh$), 4.42 (1H, dd, *J* 14.6, 5.8, $NHCH_AH_BPh$), 6.01 (1H, app t (br), *J* 5.7, N*H*), 7.25-7.30 (3H, m, $PhC^{2,4,6}H$), 7.30-7.35 (2H, m, $PhC^{3,5}H$); **^{13}C{^{1}H} NMR** (126 MHz, $CDCl_3$) δ_C: 13.9 (OCH_2CH_3), 19.5 ($COCH_2CH_2$), 30.9 (CCH_2CH_2), 34.3 ($CHCH_2$), 37.6 ($COCH_2CH_2$), 44.0 ($NHCH_2Ph$), 49.8 (app t, *J* 21.0, $CHCH_2$), 61.5 (CCH_2CH_2), 62.6 (OCH_2CH_3), 127.7 (PhC^4H), 128.0 ($PhC^{2,6}H$), 128.8 ($PhC^{3,5}H$), 130.7 (app t, *J* 297.8, CF_2Cl), 138.1 (PhC^1), 168.5 (*C*OOEt), 169.4 (*C*ONH), 210.8 ($COCH_2CH_2$); **^{19}F NMR** (471 MHz, $CDCl_3$) δ_F: −51.40 (1F, dd, *J* 162.9, 19.1, CF_AF_BCl), −46.99 (1F, dd, *J* 162.4, 4.1, CF_AF_BCl); **HRMS** (ESI^+) $C_{19}H_{23}F_2O_4NCl$ $[M]^+$ found 402.1270, requires 402.1278 (–2.0 ppm).

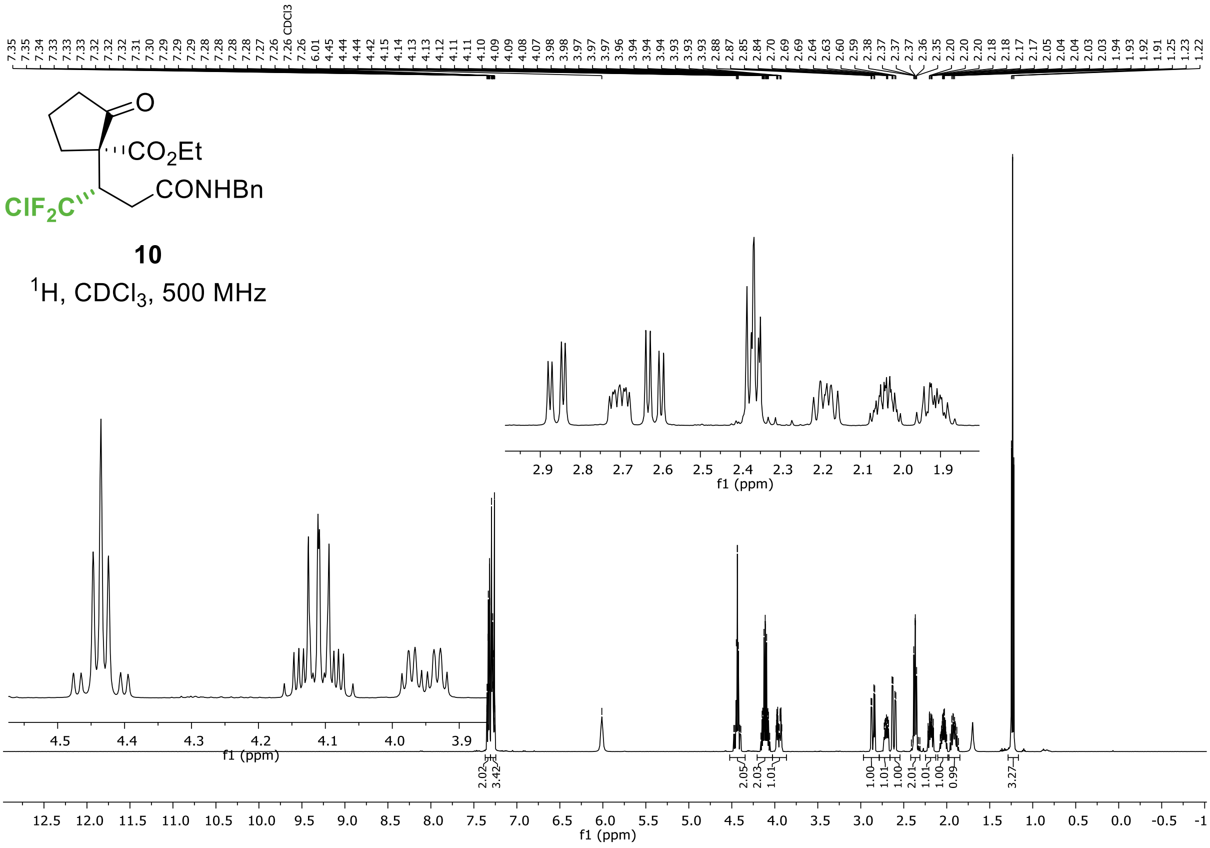
CO2Et
CONHBn
ClF2C
10
1H, CDCl3, 500 MHz
f1 (ppm)

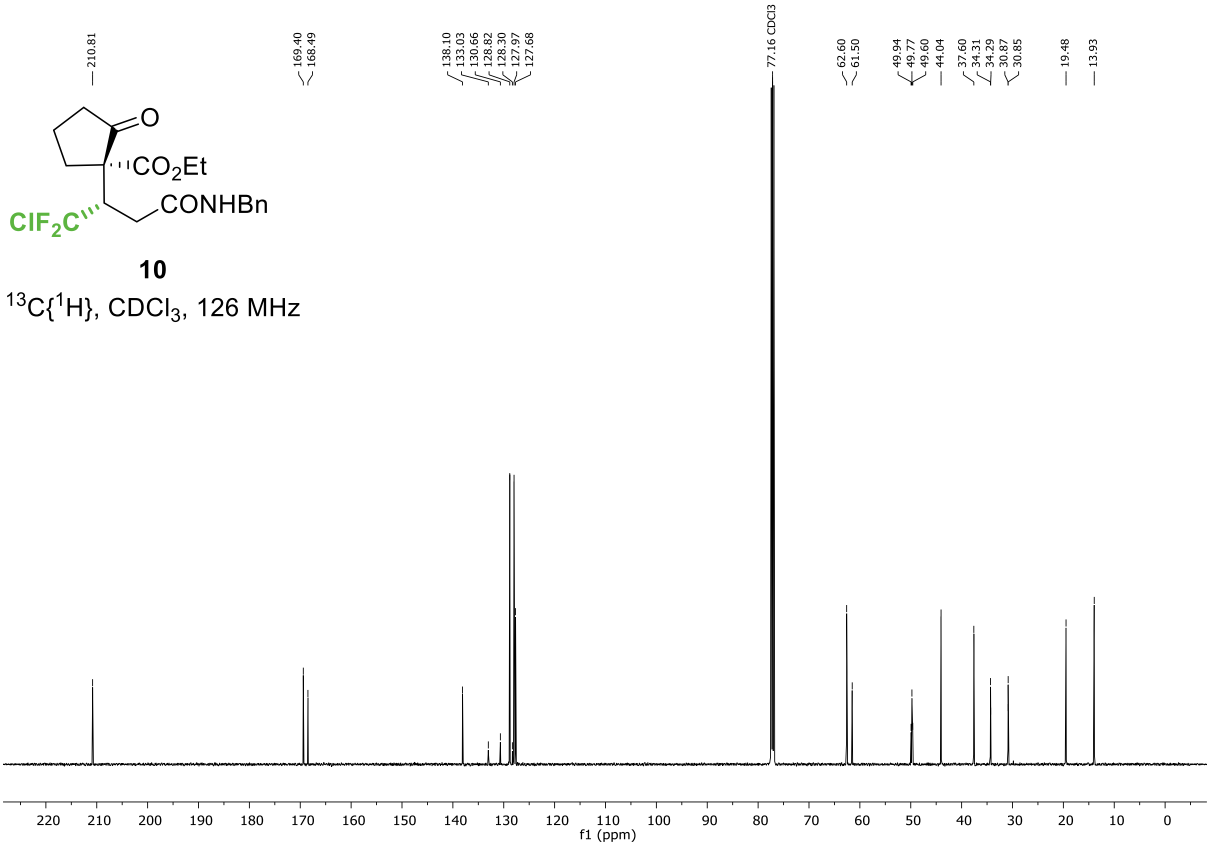
CO2Et
CONHBn
ClF2C
10
13C{1H}, CDCl3, 126 MHz
f1 (ppm)

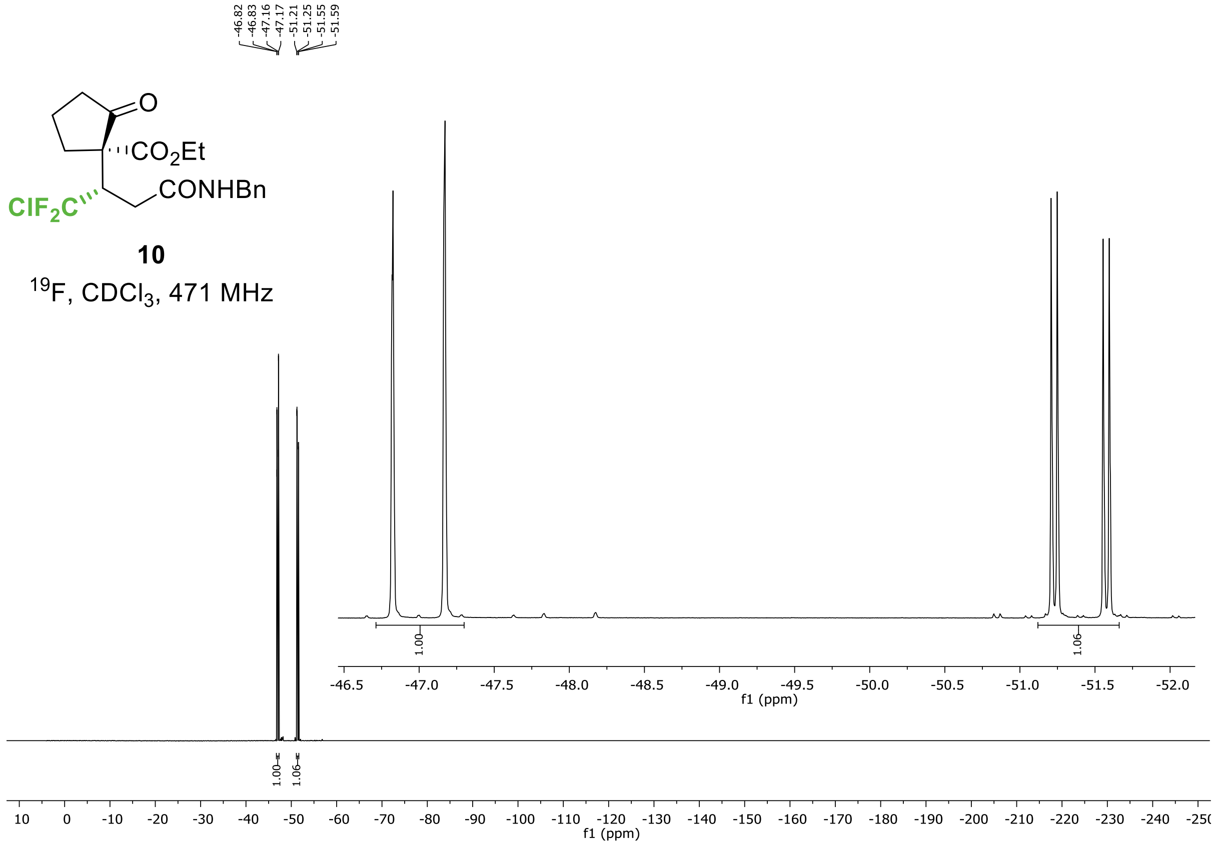

HPLC Data for 10: Chiralpak AD-H (90:10 hexane:^{i}PrOH, flow rate 1.00 mL·min^{-1}, 254 nm, 30 °C) t$_R$ (1*S*,2'*S*): 10.2 min, t$_R$ (1*R*,2'*R*): 12.7 min, 99:1 er.

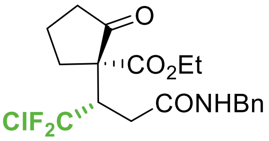

(1*S*,2'*S*)-**10**

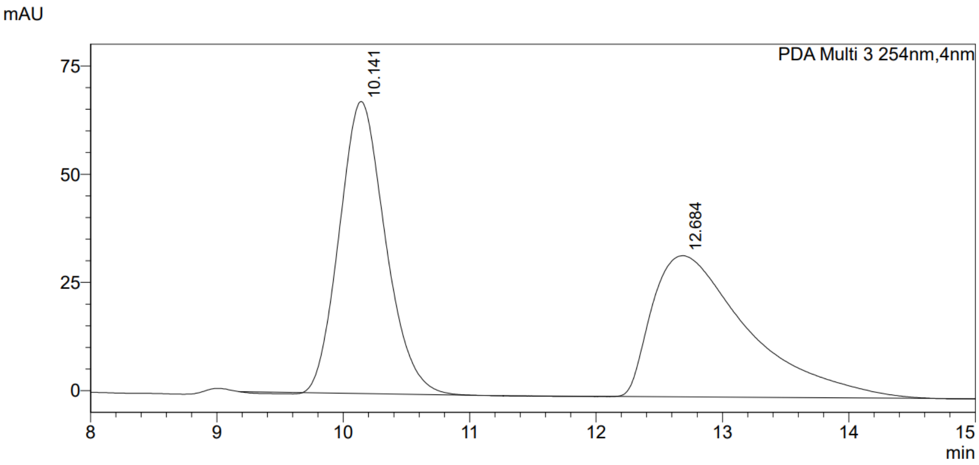

<Peak Table>

PDA Ch3 254nm

Peak#	Ret. Time	Area%
1	10.141	49.726
2	12.684	50.274
Total		100.000

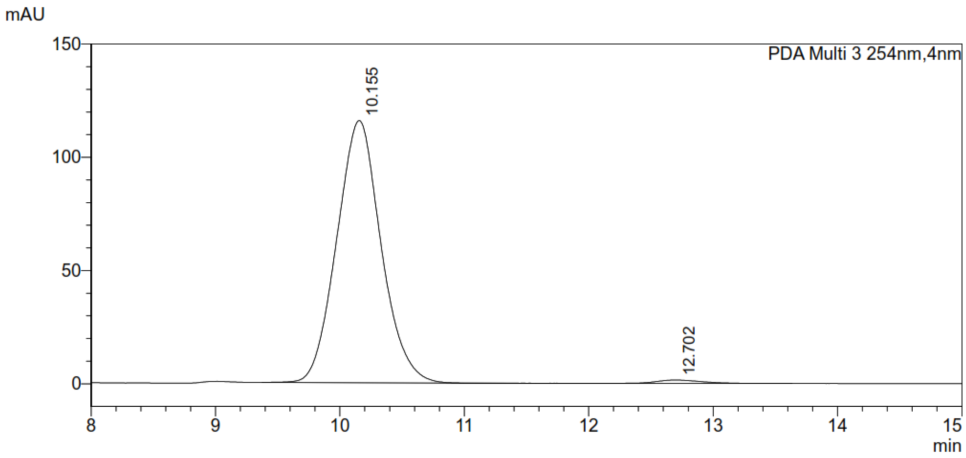

<Peak Table>

PDA Ch3 254nm

Peak#	Ret. Time	Area%
1	10.155	98.726
2	12.702	1.274
Total		100.000

(+)-Ethyl (*S*)-1-((*S*)-4'-(benzylamino)-1'-bromo-1',1'-difluoro-4'-oxobutan-2'-yl)-2-oxocyclopentane-1-carboxylate (1*S*,2'*S*)-11

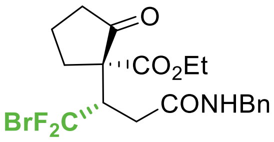

(1*S*,2'*S*)-**11**

The title compound was prepared according to **General Procedure 2**. A mixture of 4-nitrophenyl (*E*)-4-bromo-4,4-difluorobut-2-enoate **S2** (32.1 mg, 0.1 mmol), (2*S*,3*R*)-HyperBTM **1** (1.5 mg, 5 µmol) and methyl 2-oxocyclopentane-1-carboxylate **7** (15 µL, 0.1 mmol) in DMA (0.4 mL) was stirred for 16 h at room temperature. Benzylamine (11 µL, 0.1 mmol) was added, and the reaction was permitted to stir for 1 h. The solution was diluted with EtOAc, washed sequentially with saturated aqueous Na_2CO_3 (×3), brine, dried over $MgSO_4$ and filtered. The solvent was removed under reduced pressure and the residue was purified by column chromatography (1:4 EtOAc:Hexane) to give the title compound (19.7 mg, 44%, >95:5 d.r.) as white solid.

Data for (1*S*,2'*S*)-**11**: **mp** 47-52 ºC; $[\alpha]_D^{20}$ +11.38 (*c* 0.65 in $CHCl_3$); **IR ν$_{max}$** (film) / cm^{-1} 3383, 3306, 2978, 1755, 1722, 1653, 1541, 1225, 1101, 987, 822; **^{1}H NMR** (500 MHz, $CDCl_3$) δ_H: 1.23 (3H, t, *J* 7.1, C*H*$_3$), 1.83-1.95 (1H, m, $COCH_2CH_AH_B$ with *H*$_A$), 2.04 (1H, app dtdd, *J* 13.4, 7.2, 5.9, 4.1, $COCH_2CH_A$*H*$_B$), 2.18 (1H, ddd, *J* 13.8, 9.6, 6.8, CC*H*$_A$H$_B$CH$_2$), 2.29-2.42 (2H, m, COC*H*$_2$CH$_2$), 2.66 (1H, dd, *J* 16.8, 5.1, CHC*H*$_A$H$_B$CONH), 2.71 (1H, *J* 13.8, 7.2, 4.0, CCH$_A$*H*$_B$CH$_2$), 2.88 (1H, dd, *J* 16.7, 5.3, CHCH$_A$*H*$_B$CONH), 4.03 (1H, app dtd, *J* 22.6, 5.2, 2.6, C*H*CH$_2$CONH), 4.07 (1H, dq, *J* 10.7, 7.1, OC*H*$_A$H$_B$CH$_3$), 4.12 (1H, dq, *J* 10.7, 7.1, OCH$_A$*H*$_B$CH$_3$), 4.42 (1H, dd, *J* 14.9, 5.6, NHC*H*$_A$H$_B$Bn), 4.46 (1H, dd, *J* 14.9, 5.6, NHCH$_A$*H*$_B$Bn) 6.00 (1H, app t, *J* 5.6, N*H*), 7.25-7.31 (3H, m, PhC^{2,4,6}*H*), 7.31-7.36 (2H, m, PhC^{3,5}*H*); **^{13}C{^{1}H} NMR** (126 MHz, $CDCl_3$) δ_C: 13.9 (OCH$_2$*C*H$_3$), 19.5 (COCH$_2$*C*H$_2$), 31.1 (app t, *J* 2.9, C*C*H$_2$CH$_2$), 34.7 (CH*C*H$_2$CONH), 37.6 (CO*C*H$_2$CH$_2$), 44.1 (NH*C*H$_2$Ph), 51.6 (app t, *J* 18.6, *C*HCH$_2$CONH), 61.6 (*C*CH$_2$CH$_2$), 62.6 (O*C*H$_2$CH$_3$), 123.9 (app t, *J* 311.6, *C*F$_2$Br), 127.7 (Ph*C*^{4}H), 128.0 (Ph*C*^{2,6}H), 128.8 (Ph*C*^{3,5}H), 138.1 (Ph*C*^{1}), 168.4 (*C*OOEt), 169.3 (*C*ONH), 210.7 (*C*OCH$_2$CH$_2$); **^{19}F NMR** (471 MHz, $CDCl_3$) δ_F: −45.41 (1F, dd, *J* 158.2, 22.7, C*F*$_A$F$_B$Br), −40.49 (1F, d, *J* 158.3, CF$_A$*F*$_B$Br); **HRMS** (ESI$^+$) $C_{19}H_{23}F_3O_4NBr$ [M+H]$^+$ found 446.0768, requires 446.0779 (–1.2 ppm).

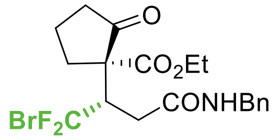

11

^{1}H, $CDCl_3$, 500 MHz

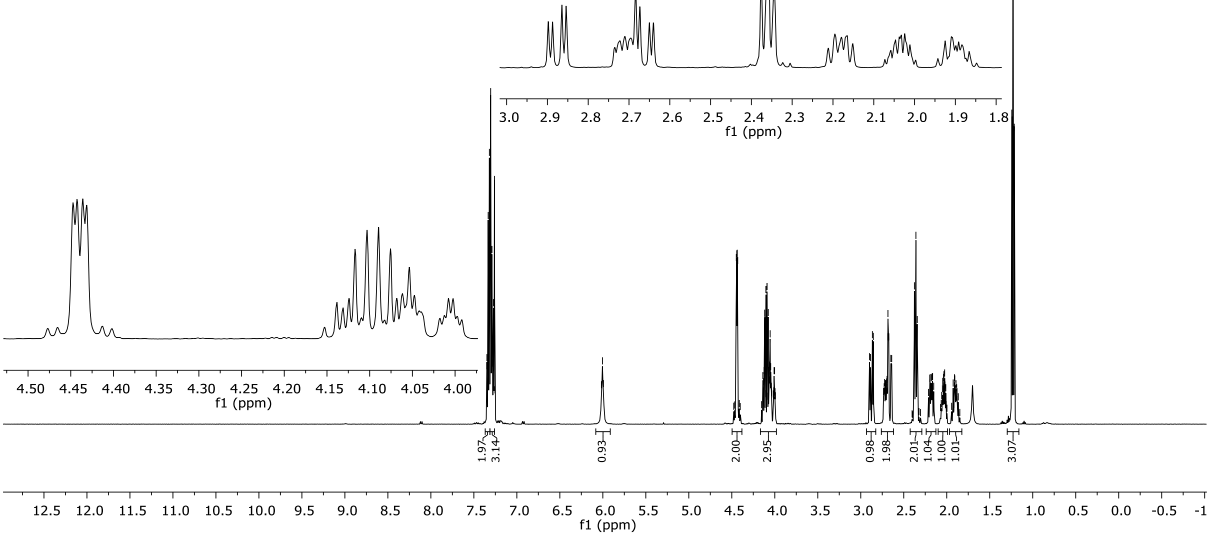

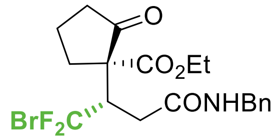

11

$^{13}C\{^{1}H\}$, $CDCl_3$, 126 MHz

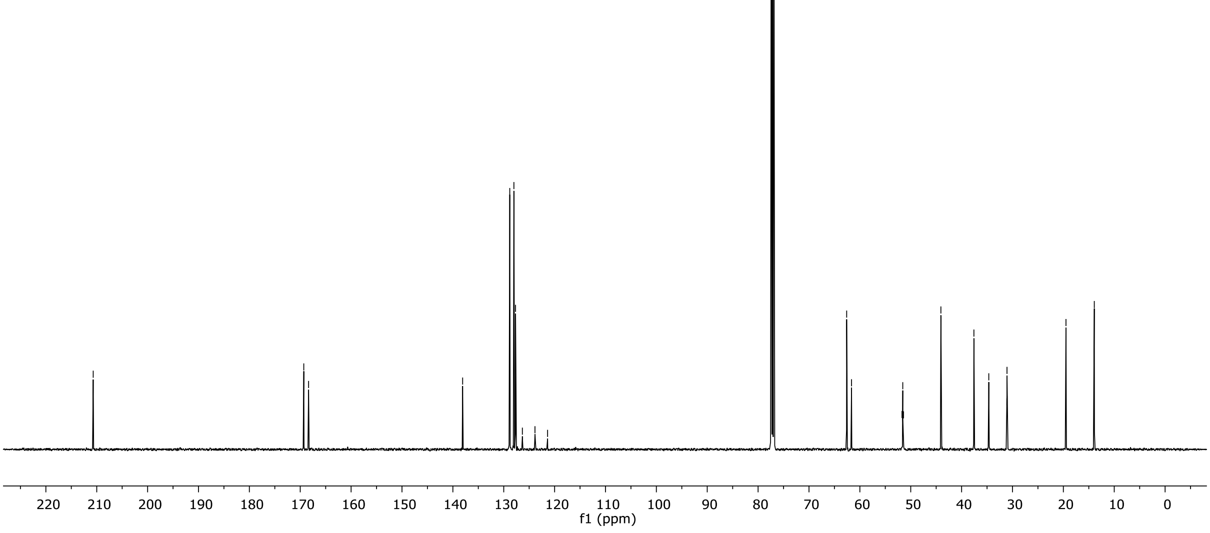

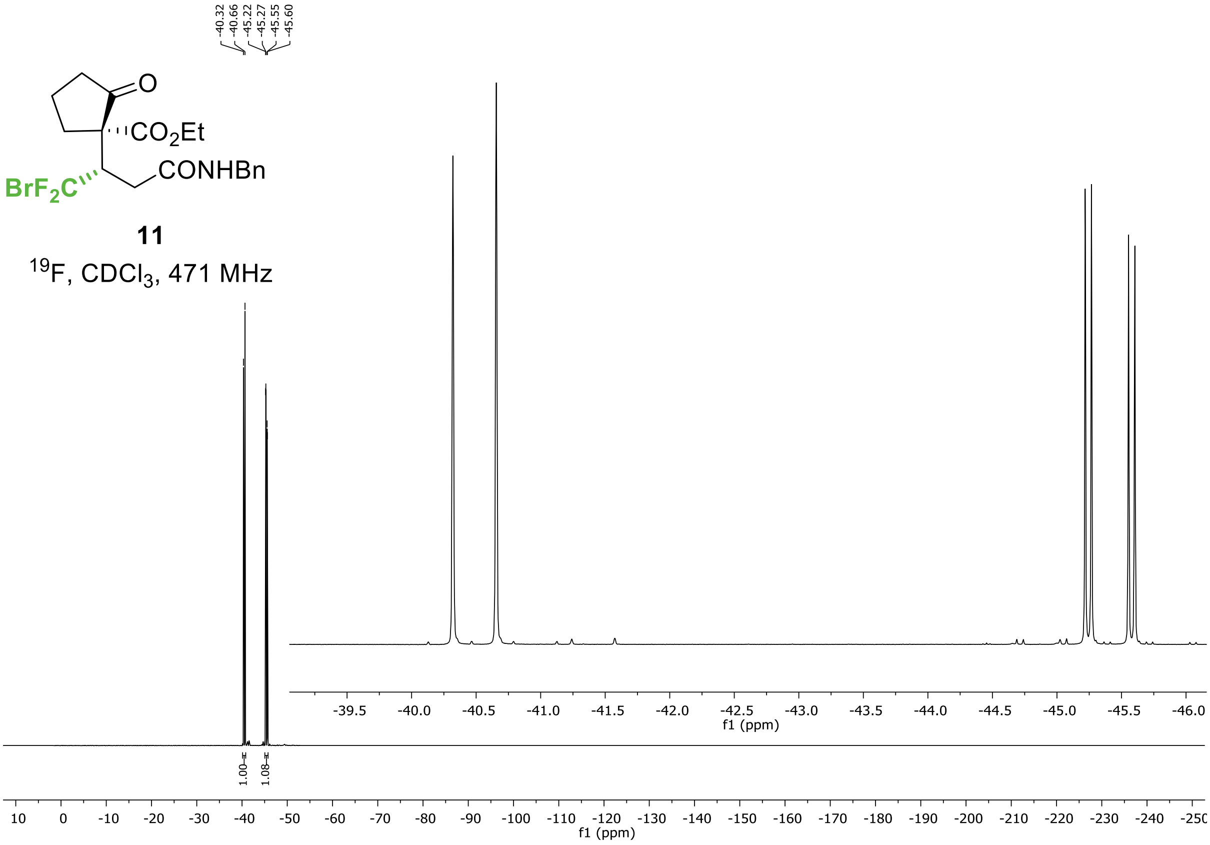

HPLC Data for 11: Chiralpak AD-H (90:10 hexane:^{i}PrOH, flow rate 1.00 mL·min^{-1}, 211 nm, 30 °C) t_R (1*S*,2’*S*): 10.5 min, t_R (1*R*,2’*R*): 13.6 min, 99:1 er.

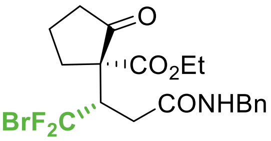

(1*S*,2’*S*)-**11**

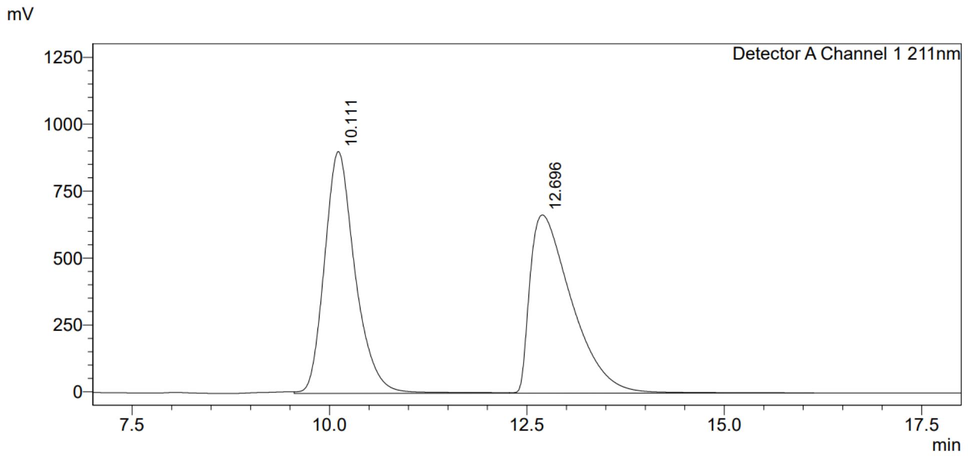

<Peak Table>

Detector A Channel 1 211nm

Peak#	Ret. Time	Area%
1	10.111	49.900
2	12.696	50.100
Total		100.000

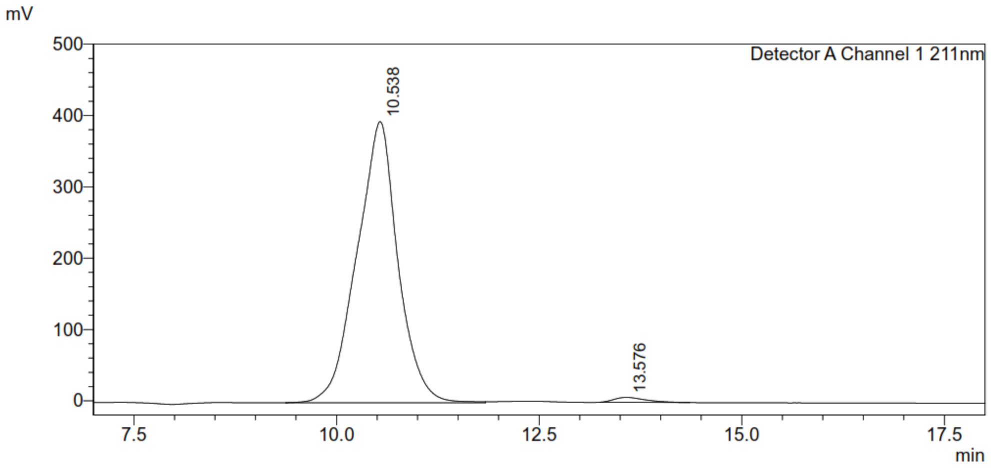

<Peak Table>

Detector A Channel 1 211nm

Peak#	Ret. Time	Area%
1	10.538	98.681
2	13.576	1.319
Total		100.000

(−)-Ethyl (*S*)-1-((*S*)-5-(benzylamino)-1,1,1,2,2-pentafluoro-5-oxopentan-3-yl)-2-oxocyclopentane-1-carboxylate (1*S*,3'*S*)-12

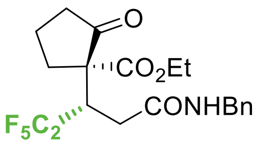

(1*S*,3'*S*)-**12**

The title compound was prepared according to **General Procedure 2**. A mixture of 4-nitrophenyl (*E*)-4,4,5,5,5-pentafluoropent-2-enoate **S3** (31.1mg, 0.1 mmol), (2*S*,3*R*)-HyperBTM **1** (1.5 mg, 5 µmol) and

methyl 2-oxocyclopentane-1-carboxylate **7** (15 μL, 0.1 mmol) in DMA (0.4 mL) was stirred for 16 h at room temperature. Benzylamine (11 μL, 0.1 mmol) was then added, and the reaction was permitted to stir for 1 h. The solution was diluted with EtOAc, washed sequentially with saturated aqueous Na_2CO_3 (×3) and brine, dried over $MgSO_4$ and filtered. The solvent was removed under reduced pressure and the residue was purified by column chromatography (1:4 EtOAc:Hexane) to give the title compound (16.3 mg, 37%, 93:7 d.r.), as a colourless oil.

Data for (1*S*,3'*S*)-**12**: $[\alpha]_D^{20}$ −3.49 (*c* 0.43 in $CHCl_3$); **IR** ν_{max} (film) / cm^{-1} 3308, 2967, 1757, 1724, 1655, 1541, 1182, 1125, 1001, 934, 858; **^{1}H NMR** (500 MHz, $CDCl_3$) δ_H: 1.23 (3H, t, *J* 7.1, OCH_2CH_3), 1.95-2.05 (1H, m, $COCH_2CH_AH_B$), 2.05-2.17 (3H, m, $CCH_AH_BCH_2$, $COCH_2CH_AH_B$), 2.33 (1H, ddd, *J* 18.6, 8.8, 5.5, $COCH_AH_BCH_2$), 2.42 (1H, app dt, *J* 18.6, 8.3, $COCH_AH_BCH_2$), 2.58 (1H, d (br), *J* 17.4, $CHCH_AH_BCONH$), 2.68-2.78 (1H, m, $CCH_AH_BCH_2$), 2.86 (1H, dd, *J* 17.4, 7.1, $CHCH_AH_BCONH$), 3.89 (1H, app ddt, *J* 27.2, 6.8, 3.0, $CHCH_2CONH$), 4.10 (1H, dq, *J* 10.7, 7.1, $OCH_AH_BCH_3$), 4.14 (1H, dq, *J* 10.7, 7.1, $OCH_AH_BCH_3$), 4.38 (1H, dd, *J* 14.8, 5.7, $NHCH_AH_BPh$), 4.42 (1H, dd, *J* 14.8, 5.7, $NHCH_AH_BPh$), 5.74 (1H, app t, J 5.7, N*H*), 7.25-7.30 (3H, m, $PhC^{2,4,6}H$), 7.30-7.37 (2H, m, $PhC^{3,5}H$); **$^{13}C\{^1H\}$ NMR** (126 MHz, $CDCl_3$) δ_C: 13.9 (OCH_2CH_3), 19.5 ($COCH_2CH_2$), 32.4 and 32.5 ($CHCH_2CONH$, CCH_2CH_2), 37.7 ($COCH_2CH_2$), 39.6 (app t, *J* 18.4, $CHCH_2CONH$), 44.1 ($NHCH_2Ph$), 61.0 (q, *J* 2.4, CCH_2CH_2), 62.4 (OCH_2CH_3), 115.8 (ddq. *J* 262.1, 258.0, 36.8, CF_2CF_3), 119.0 (app qt, *J* 286.9, 37.2, CF_2CF_3), 127.7 (PhC^4H), 128.0 ($PhC^{2,6}H$), 128.9 ($PhC^{3,5}H$), 138.0 (PhC^1), 169.1_7 and 169.2_1 (*C*OOEt, *C*ONH), 211.7 ($COCH_2CH_2$); **^{19}F NMR** (471 MHz, $CDCl_3$) δ_F: −114.5 (1F, dd, *J* 271.9, 27.1, $CF_AF_BCF_3$), −110.84 (1F, d, *J* 271.7, $CF_AF_BCF_3$), −85.10 (3F, s, CF_3); **HRMS** (ESI^+) $C_{20}H_{23}F_5O_4N$ $[M+H]^+$ found 436.1535, requires 436.1542 (–1.6 ppm).

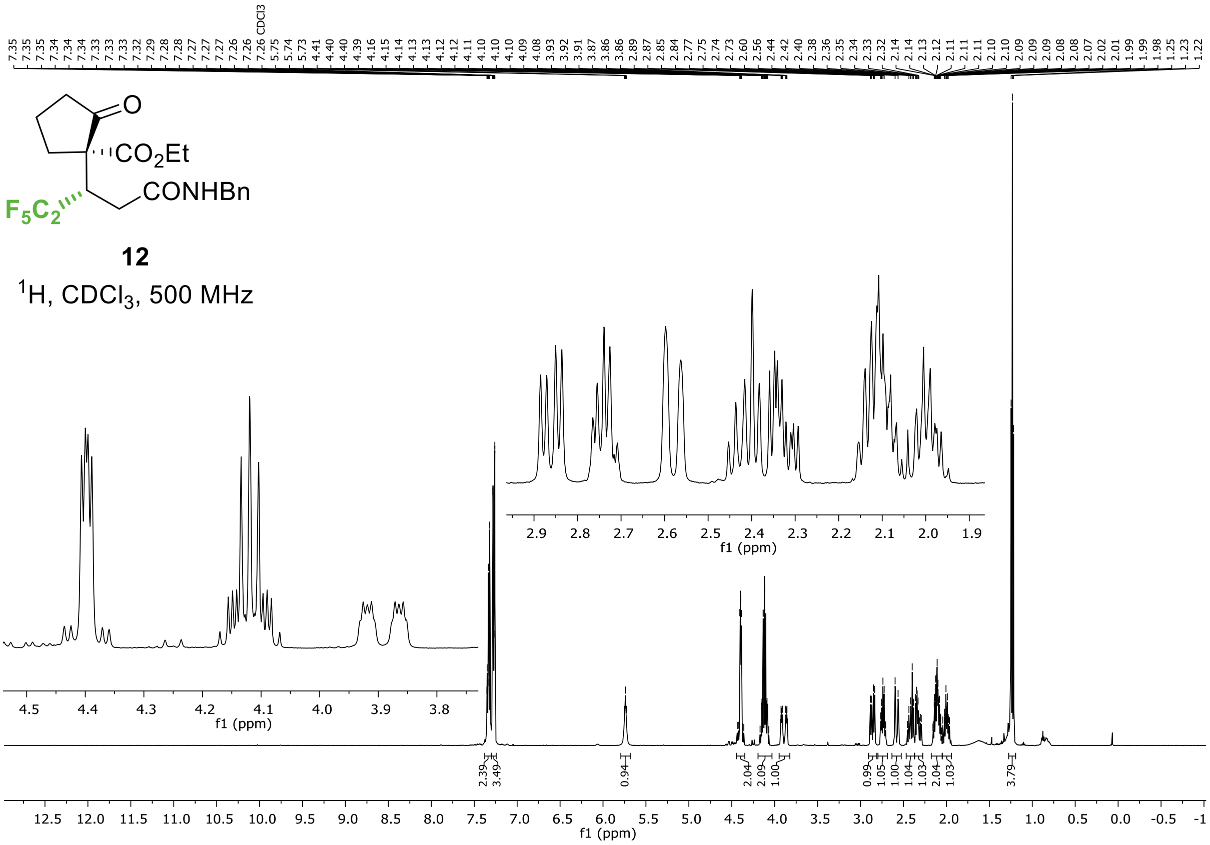
7.35 7.35 7.35 7.34 7.34 7.33 7.33 7.32 7.32 7.29 7.28 7.28 7.27 7.27 7.27 7.26 7.26 7.26 CDCl3 5.75 5.74 5.73 4.41 4.40 4.39 4.16 4.15 4.14 4.13 4.13 4.12 4.12 4.11 4.10 4.10 4.10 4.09 4.08 3.93 3.92 3.91 3.87 3.86 3.86 2.89 2.87 2.85 2.84 2.77 2.75 2.74 2.73 2.60 2.56 2.44 2.42 2.40 2.38 2.36 2.35 2.34 2.33 2.32 2.14 2.14 2.13 2.12 2.11 2.11 2.11 2.10 2.10 2.09 2.09 2.09 2.08 2.08 2.07 2.02 2.01 1.99 1.99 1.98 1.25 1.23 1.22
O
CO2Et
CONHBn
F5C2
12
1H, CDCl3, 500 MHz
2.39 3.49 0.94 2.04 2.09 1.00 0.99 1.05 1.00 1.04 1.03 2.04 1.03 3.79
f1 (ppm)

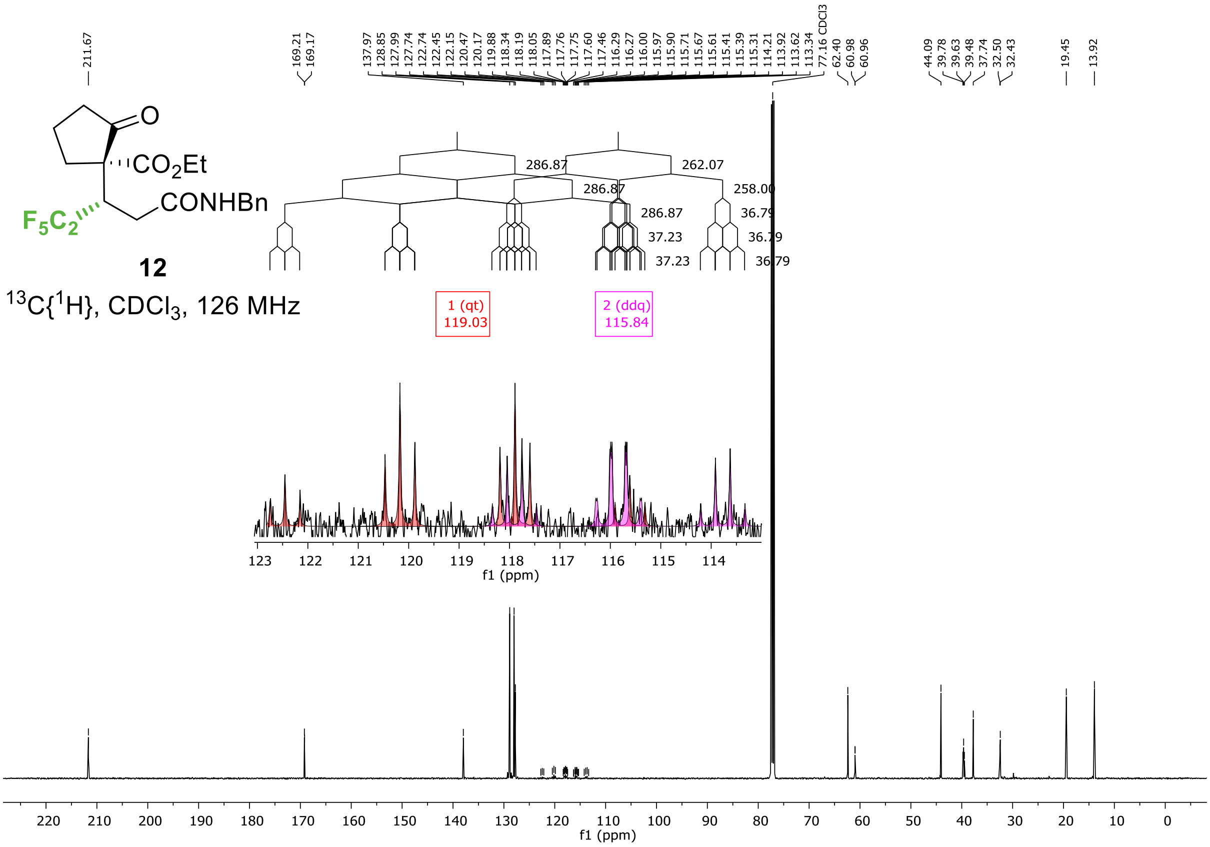
211.67
169.21 169.17
137.97 128.85 127.99 127.74 122.74 122.45 122.15 120.47 120.17 119.88 118.34 118.19 118.05 117.89 117.76 117.75 117.60 117.46 116.29 116.27 116.00 115.97 115.90 115.71 115.67 115.61 115.41 115.39 115.31 114.21 113.92 113.62 113.34 77.16 CDCl3 62.40 60.98 60.96
44.09 39.78 39.63 39.48 37.74 32.50 32.43
19.45
13.92
O
CO2Et
CONHBn
F5C2
12
13C{1H}, CDCl3, 126 MHz
286.87 262.07 286.87 258.00 286.87 36.79 37.23 36.79 37.23 36.79
1 (qt) 119.03
2 (ddq) 115.84
f1 (ppm)

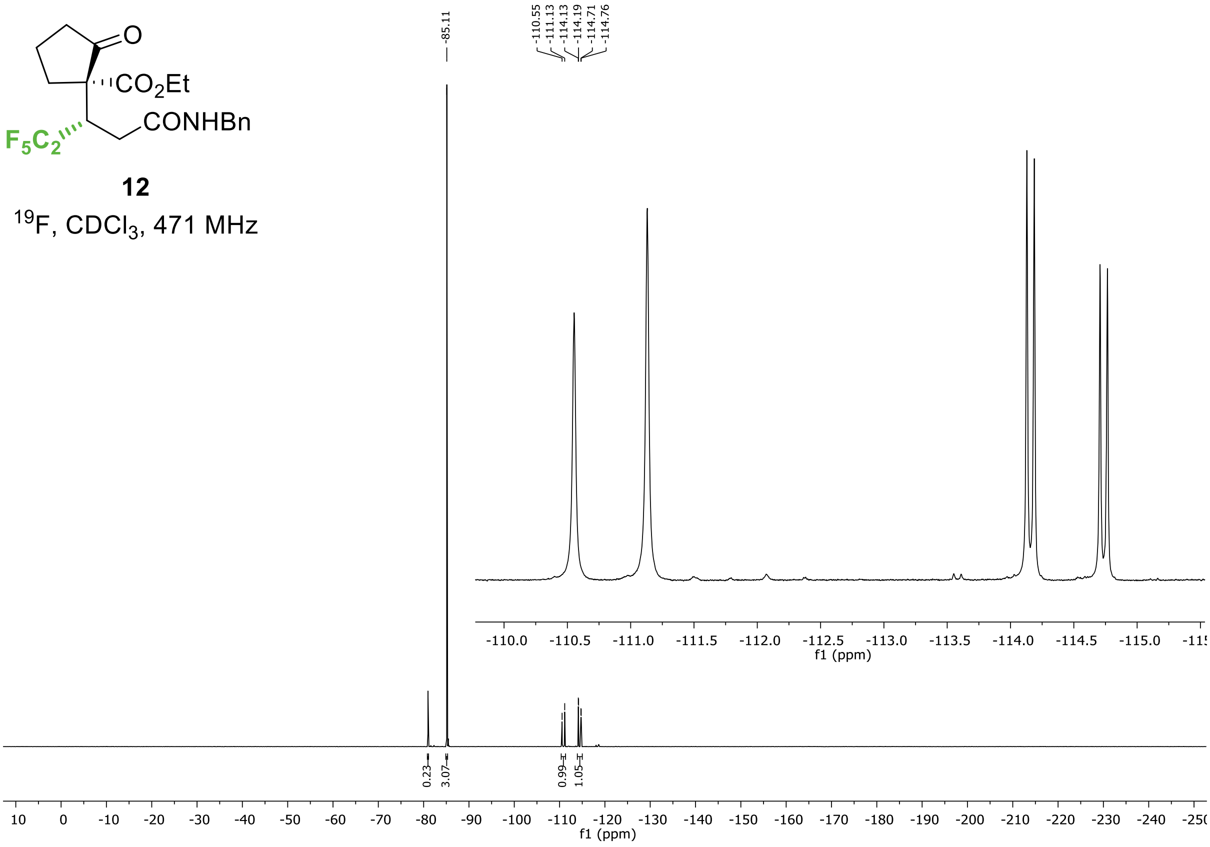

HPLC Data for 12: Chiralpak AD-H (95:5 hexane:^{i}PrOH, flow rate 1.00 mL·min^{-1}, 211 nm, 30 °C) t_R (1*R*,3'*R*): 9.4 min, t_R (1*S*,3'*S*): 12.2 min, 99:1 er.

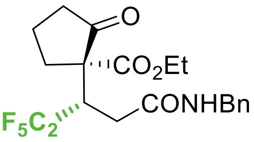

(1*S*,3'*S*)-**12**

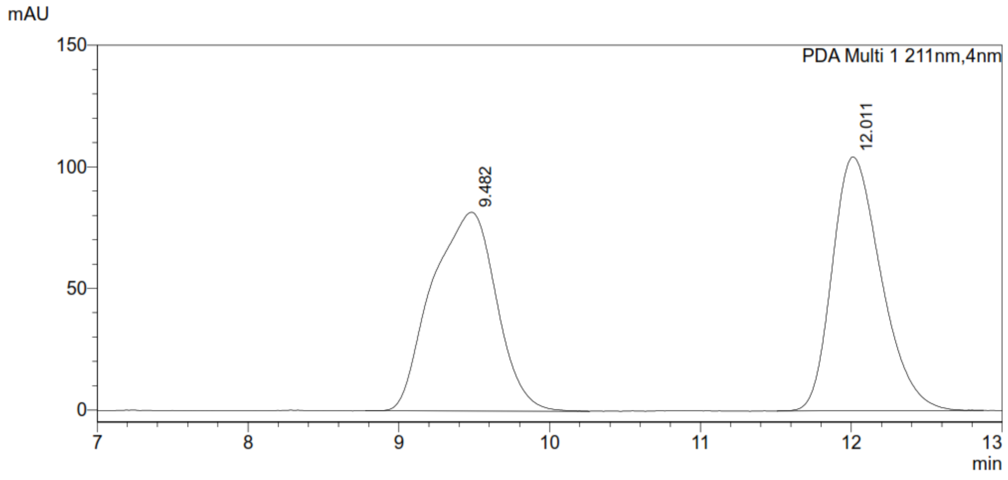

<Peak Table>

PDA Ch1 211nm

Peak#	Ret. Time	Area%
1	9.482	50.849
2	12.011	49.151
Total		100.000

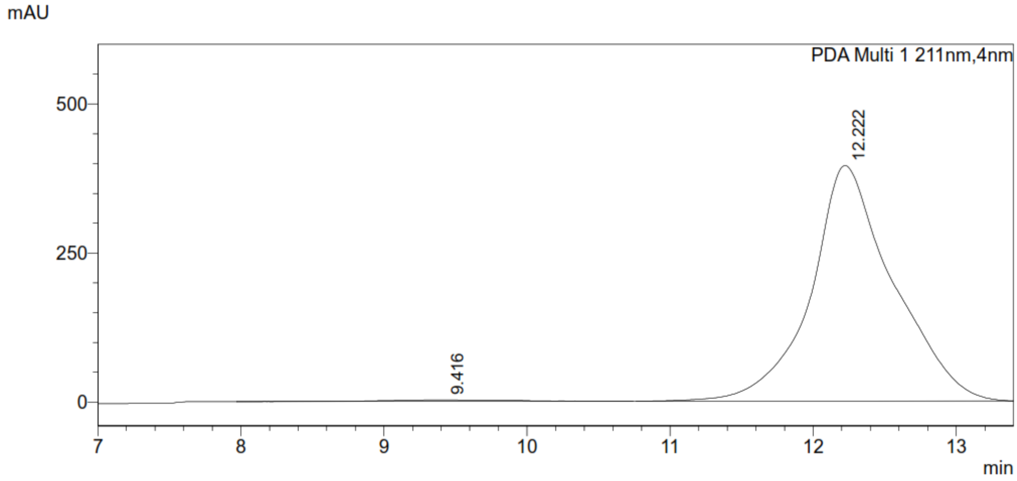

<Peak Table>

PDA Ch1 211nm

Peak#	Ret. Time	Area%
1	9.416	1.006
2	12.222	98.994
Total		100.000

(+)-Ethyl (*S*)-1-((*S*)-4'-(benzylamino)-1'-ethoxy-1',4'-dioxobutan-2'-yl)-2-oxocyclopentane-1-carboxylate (1*S*,2'S)-13

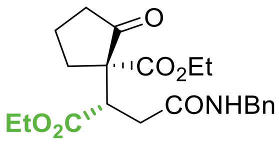

(1*S*,2'*S*)-**13**

Preparation from fumarate:

The title compound was prepared according to **General Procedure 2**. A mixture of ethyl 4-nitrophenyl fumarate **26** (26.5 mg, 0.1 mmol), (2*S*,3*R*)-HyperBTM **8** (1.5 mg, 5 μmol) and methyl 2-oxocyclopentane-1-carboxylate **7** (15 μL, 0.1 mmol) in DMA (0.4 mL) was stirred for 16 h at room temperature. Benzylamine (11 μL, 0.1 mmol) was added and the reaction was permitted to stir for 1 h. The solution was diluted with EtOAc, washed sequentially with saturated aqueous Na_2CO_3 (×3) and brine, dried over $MgSO_4$ and filtered. The solvent was removed under reduced pressure and the residue was purified by column chromatography (1:4 EtOAc:Hexane) to give the title compound (20.2 mg, 52%, >95:5 d.r.) as colourless oil.

Preparation from maleate:

The title compound was prepared according to **General Procedure 2**. A mixture of ethyl (4-nitrophenyl) maleate **24** (26.5 mg, 0.1 mmol), (2*S*,3*R*)-HyperBTM **1** (1.5 mg, 5 μmol) and methyl 2-oxocyclopentane-1-carboxylate **7** (15 μL, 0.1 mmol) in DMA (0.4 mL) was stirred for 16 h at room temperature. Benzylamine (11 μL, 0.1 mmol) was added, and the reaction was permitted to stir for 1 h. The solution was diluted with EtOAc, washed sequentially with saturated aqueous Na_2CO_3 (×3) and brine, dried over $MgSO_4$ and filtered. The solvent was removed under reduced pressure and the residue was purified by column chromatography (1:4 EtOAc:Hexane) to give the title compound in two fractions (major diastereomer: 15.9 mg, 41% as a colourless oil, and minor diastereomer: 5.6 mg, 14% as a colourless oil); combined (21.5 mg, 55%, 74:26 d.r.).

Data for major diastereomer (1*S*,2'*S*)-**13**: $[\alpha]_D^{20}$ +22.31 (*c* 0.65 in $CHCl_3$); **IR ν_{max}** (film) / cm^{-1} 3391, 3306, 2978, 1721, 1651, 1539, 1221, 1155, 1026, 916, 820; **^{1}H NMR** (500 MHz, $CDCl_3$) δ_H: 1.19 (3H, t, *J* 7.1, $CHCOOCH_2CH_3$), 1.23 (3H, t, *J* 7.1, $CCOOCH_2CH_3$), 1.94-2.07 (2H, m, $COCH_2CH_2$), 2.25 (1H, ddd, *J* 13.4, 9.4, 7.6, $CCH_AH_BCH_2$), 2.32-2.44 (2H, m, $COCH_2CH_2$), 2.50 (1H, ddd, *J* 13.4, 7.1, 4.0, $CCH_AH_BCH_2$), 2.60 (1H, dd, *J* 15.4, 7.7, $CHCH_AH_BCONH$), 2.64 (1H, dd, *J* 15.4, 4.9, $CHCH_AH_BCONH$), 3.77 (1H, dd, *J* 7.7, 4.8, $CHCH_2CONH$), 4.08 (1H, dq, *J* 10.8, 7.1, $CHCOOCH_AH_BCH_3$), 4.10 (1H, dq, *J* 10.8, 7.1, $CHCOOCH_AH_BCH_3$), 4.14 (2H, q, *J* 7.1, $CCOOOCH_2CH_3$), 4.39 (1H, dd, *J* 15.2, 5.7, $NHCH_AH_BPh$), 4.42 (1H, dd, *J* 15.2, 5.7, $NHCH_AH_BPh$), 6.05 (1H, app t, *J* 5.7, N*H*), 7.23-7.29 (3H, m, $PhC^{2,4,6}H$), (2H, m, $PhC^{3,5}H$); **^{13}C{^{1}H} NMR** (126 MHz, $CDCl_3$) δ_C: 14.1 ($CCOOCH_2CH_3$, $CHCOOCH_2CH_3$), 19.7 (OCH_2CH_2), 31.4 (CCH_2CH_2), 35.2 ($CHCH_2CONH$), 37.9 ($COCH_2CH_2$), 43.8 ($NHCH_2Ph$), 44.9 ($CHCH_2CONH$), 61.4 ($CHCOOCH_2CH_3$), 61.9$_6$ (CCH_2CH_2), 62.0$_4$ ($CCOOCH_2CH_3$), 127.5 (PhC^4H), 127.9 ($PhC^{2,6}H$), 128.7 ($PhC^{3,5}H$), 138.3

(Ph*C*[1]), 170.0 (C*C*OOEt), 170.5 (*C*ONHBn), 172.4 (CH*C*OOEt), 213.2 (*C*OCH_2CH_2); **HRMS** (ESI^+) $C_{21}H_{28}O_6N$ $[M+H]^+$ found 390.1914, requires 390.1917 (+0.8 ppm).

Data for minor diastereoisomer (1*R*,2'*S*)-**13**: $[\alpha]_D^{20}$ +18.3 (*c* 0.47 in $CHCl_3$); **IR ν_{max}** (film) / cm^{-1} 3242, 3067, 2972, 1719, 1641, 1557, 1213, 1153, 1026, 941, 860; **^{1}H NMR** (500 MHz, $CDCl_3$) δ_H: 1.21 (3H, t, *J* 7.2, $CHCOOCH_2C\mathit{H}_3$), 1.26 (3H, t, *J* 7.1, $COOCH_2C\mathit{H}_3$), 1.90-2.00 (1H, m, $COCH_2C\mathit{H}_2$), 2.04 (1H, app dt, *J* 13.1, 8.9, $CC\mathit{H}_A H_B$), 2.24 (1H, app dt, *J* 18.9, 9.4, $COC\mathit{H}_A H_B CH_2$), 2.36-2.46 (1H, m, $COCH_A\mathit{H}_B CH_2$), 2.38 (1H, dd, *J* 14.9, 4.0, $CHC\mathit{H}_A H_B CONH$), 2.44-2.53 (1H, m, $CCH_A\mathit{H}_B CH_2$), 2.67 (1H, dd, J 14.9, 10.1, $CHCH_A\mathit{H}_B CONH$), 3.73 (1H, dd, *J* 10.1, 3.9, $C\mathit{H}CH_2CONH$), 4.17 (2H, q, *J* 7.1, $CCOOC\mathit{H}_2CH_3$), 4.13 (1H, dq, *J* 10.8, 7.2, $CHCOOC\mathit{H}_A H_B CH_3$), 4.09 (1H, dq, *J* 10.8, 7.2, $CHCOOCH_A\mathit{H}_B CH_3$), 4.39 (1H, dd, *J* 15.0, 5.6, $NHC\mathit{H}_A H_B$), 4.42 (1H, dd, *J* 15.0, 5.6, $NHCH_A\mathit{H}_B Ph$), 5.94 (1H, app t, *J* 5.4, N*H*), 7.-25-7.30 (3H, m, $PhC^{2,4,6}\mathit{H}$), 7.30-7.35 (2H, m, $PhC^{3,5}\mathit{H}$); **^{13}C{^{1}H} NMR** (126 MHz, $CDCl_3$) δ_C:14.1 ($CCOOCH_2\mathit{C}H_3$, $CHCOOCH_2\mathit{C}H_3$), 19.5 ($COCH_2\mathit{C}H_2$), 31.4 ($C\mathit{C}H_2CH_2$), 35.3 ($CH\mathit{C}H_2CONH$), 38.4 ($CO\mathit{C}H_2CH_2$), 43.9 ($NH\mathit{C}H_2Ph$), 45.4 ($\mathit{C}HCH_2CONH$), 61.4 ($CHCOO\mathit{C}H_2CH_3$), 61.7 ($\mathit{C}CH_2CH_2$), 62.1 ($CCOO\mathit{C}H_2CH_3$), 127.6 ($Ph\mathit{C}^4H$), 128.0 ($Ph\mathit{C}^{2,6}H$), 128.8 ($Ph\mathit{C}^{3,5}H$), 138.2 (Ph*C*[1]), 169.9 (C*C*OOEt), 170.1 (*C*ONHBn), 172.8 (CH*C*OOEt), 213.2 (*C*OCH_2CH_2). **HRMS** (ESI^+) $C_{21}H_{27}O_6NNa$ $[M+Na]^+$ found 412.1731, requires 412.1721 (–2.3 ppm).

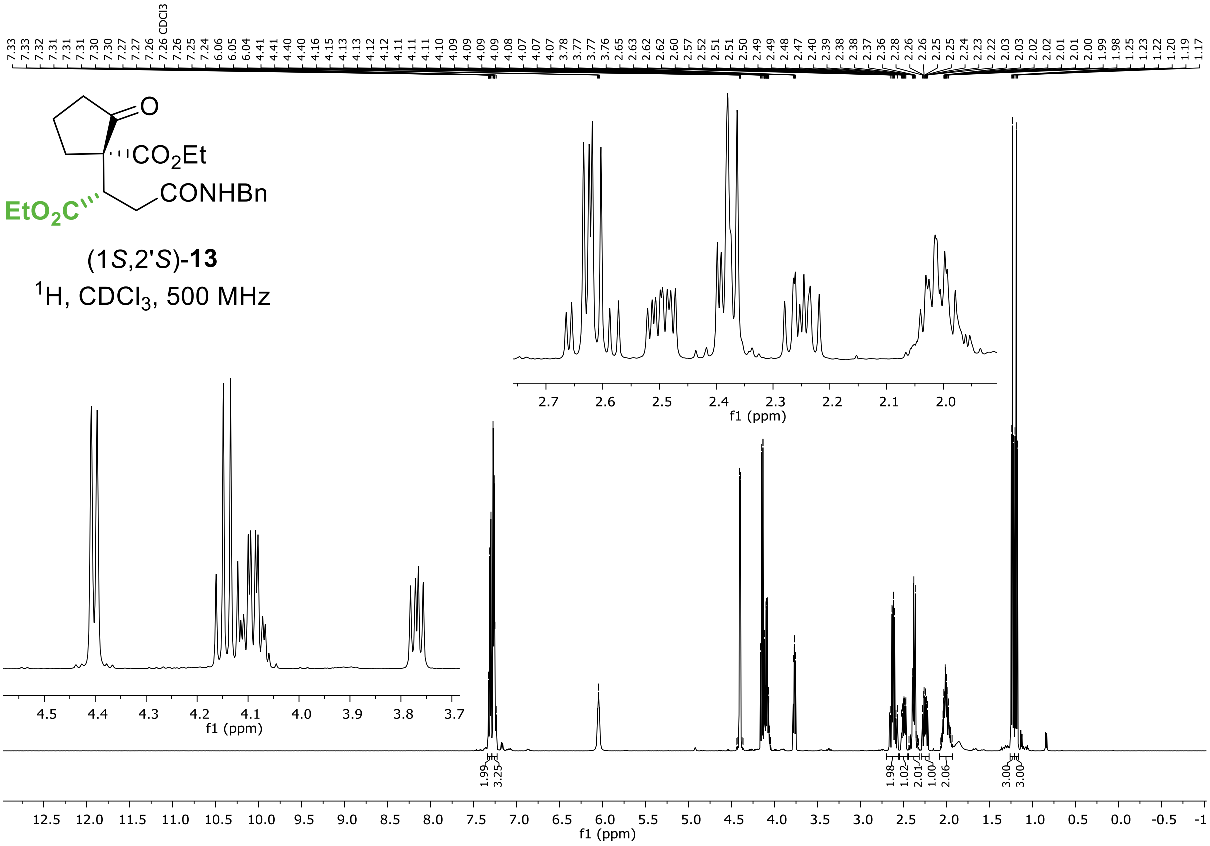

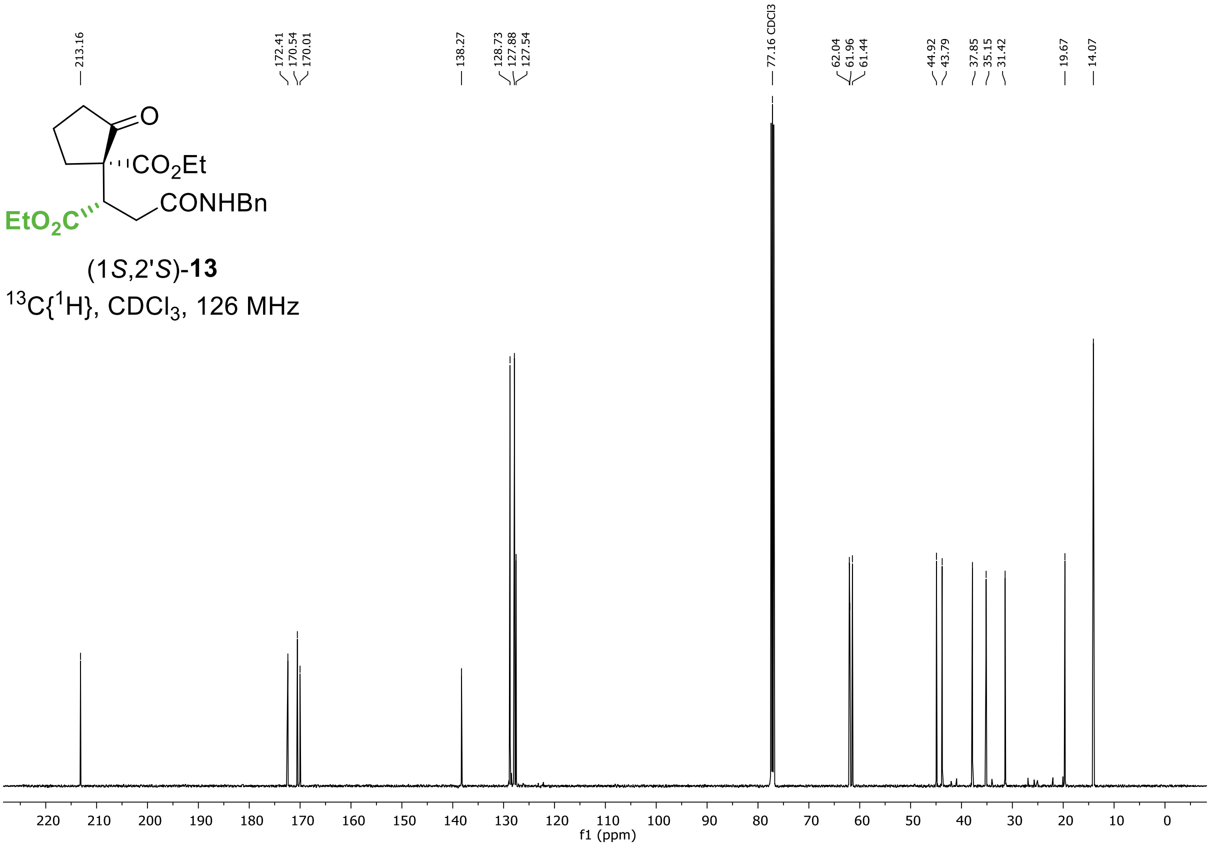
213.16
172.41
170.54
170.01
138.27
128.73
127.88
127.54
77.16 CDCl3
62.04
61.96
61.44
44.92
43.79
37.85
35.15
31.42
19.67
14.07
O
CO2Et
CONHBn
EtO2C
(1S,2'S)-13
13C{1H}, CDCl3, 126 MHz
f1 (ppm)

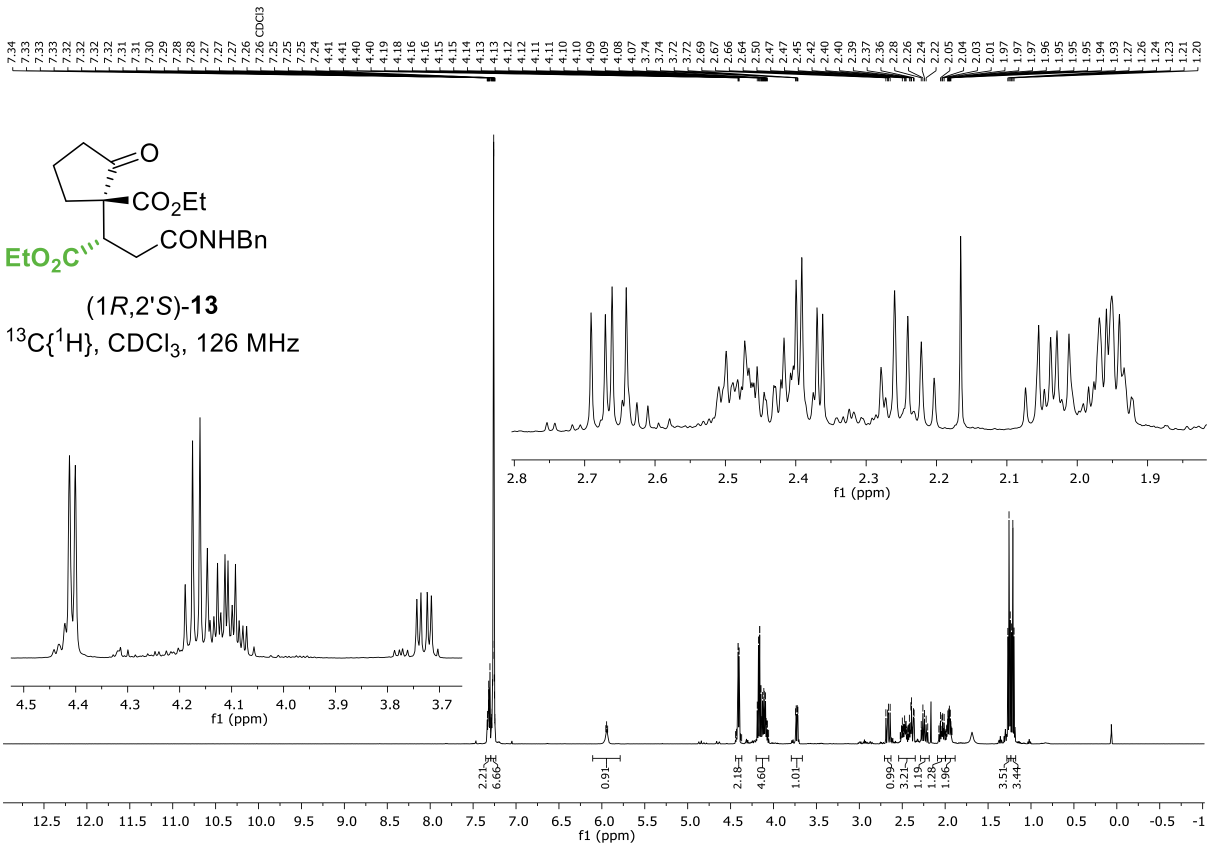
7.34
7.33
7.33
7.32
7.32
7.32
7.31
7.31
7.30
7.29
7.28
7.28
7.27
7.27
7.27
7.26 CDCl3
7.25
7.25
7.25
7.24
4.41
4.41
4.40
4.19
4.18
4.16
4.16
4.15
4.15
4.14
4.13
4.13
4.12
4.12
4.11
4.11
4.10
4.10
4.09
4.09
4.08
4.07
3.74
3.74
3.72
3.72
2.69
2.67
2.66
2.64
2.50
2.47
2.47
2.45
2.42
2.40
2.40
2.39
2.37
2.36
2.28
2.26
2.26
2.24
2.22
2.05
2.04
2.03
2.01
1.97
1.97
1.96
1.95
1.95
1.95
1.94
1.93
1.27
1.26
1.24
1.23
1.21
1.20
O
CO2Et
CONHBn
EtO2C
(1R,2'S)-13
13C{1H}, CDCl3, 126 MHz
2.21
6.60
0.91
2.18
4.60
1.01
0.99
3.21
1.19
1.28
1.96
3.51
3.44
f1 (ppm)

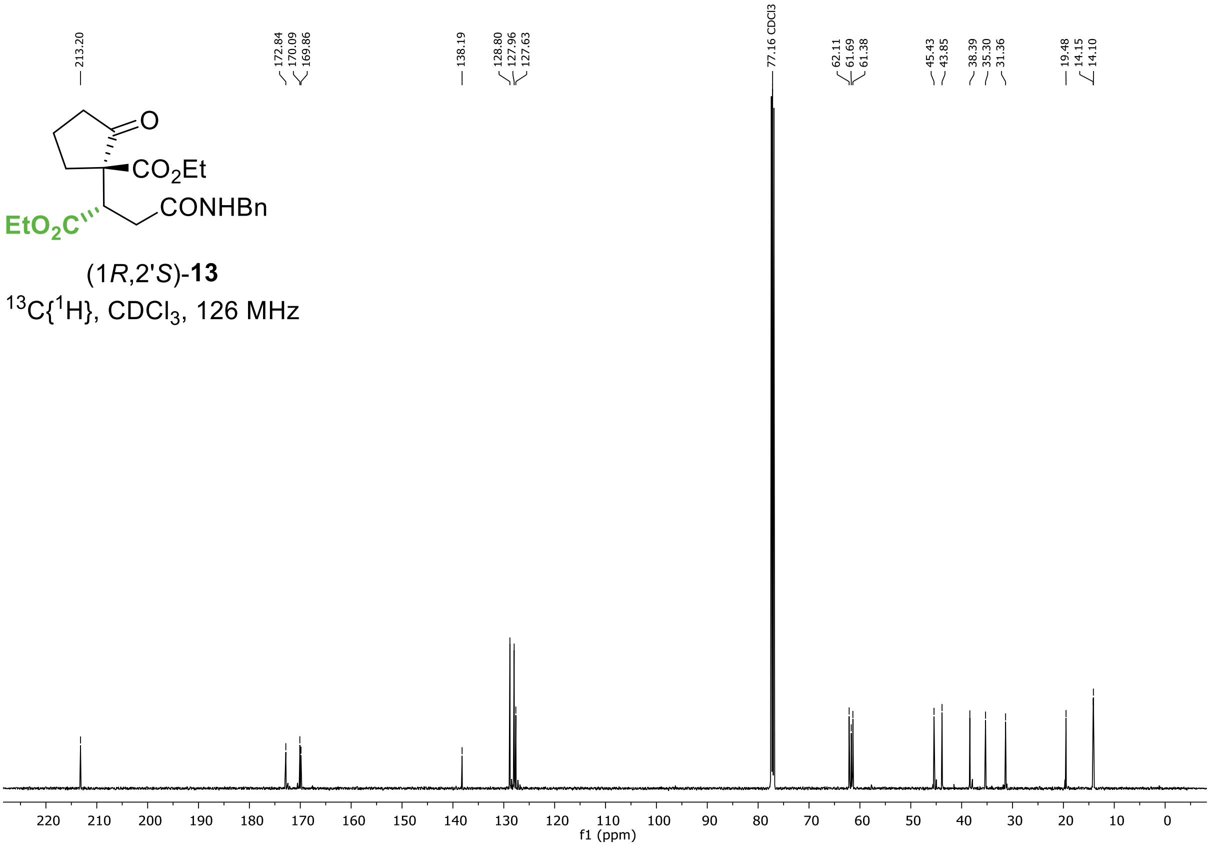

HPLC Data for 13 from fumarate: Chiralpak AD-H (90:10 hexane:^{i}PrOH, flow rate 1.00 mL·min^{-1}, 211 nm, 30 °C) t_R (1*R*,2’*R*): 20.4 min, t_R (1*S*,2’*S*): 22.4 min, 94:6 er.

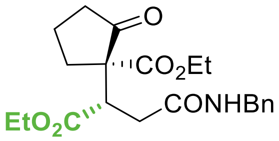

(1*S*,2’*S*)-**13**

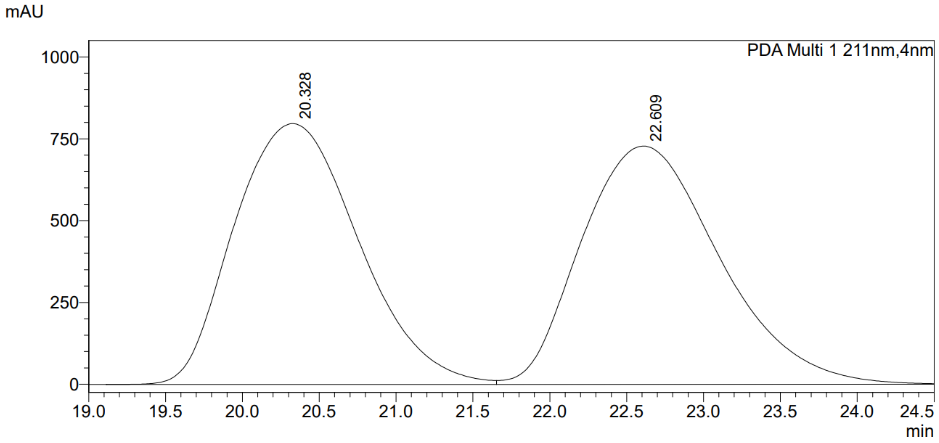

<Peak Table>

PDA Ch1 211nm

Peak#	Ret. Time	Area%
1	20.328	49.679
2	22.609	50.321
Total		100.000

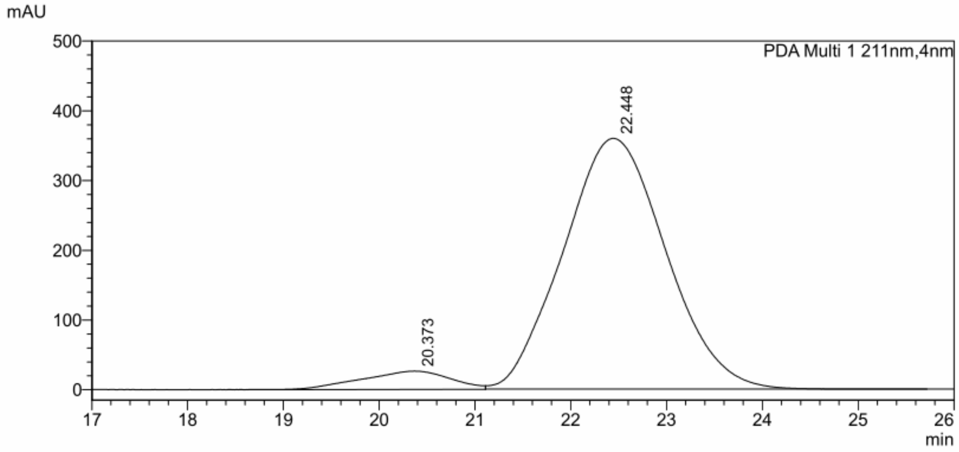

<Peak Table>

PDA Ch1 211nm

Peak#	Ret. Time	Area%
1	20.373	5.964
2	22.448	94.036
Total		100.000

HPLC Data for 13 from maleate, major diasteromer: Chiralpak AD-H (90:10 hexane:^{i}PrOH, flow rate 1.00 mL·min^{-1}, 211 nm, 30 °C) t_R (1*R*,2'*R*): 20.4 min, t_R (1*S*,2'*S*): 22.6 min, 78:22 er.

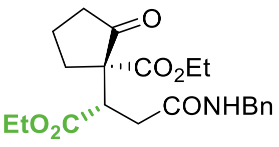

(1*S*,2'*S*)-**13**

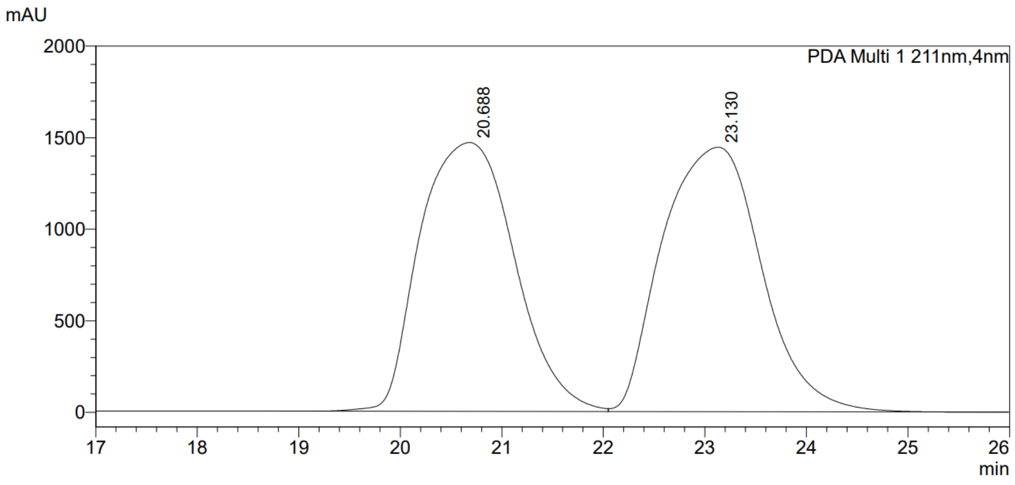

<Peak Table>

PDA Ch1 211nm

Peak#	Ret. Time	Area%
1	20.688	49.267
2	23.130	50.733
Total		100.000

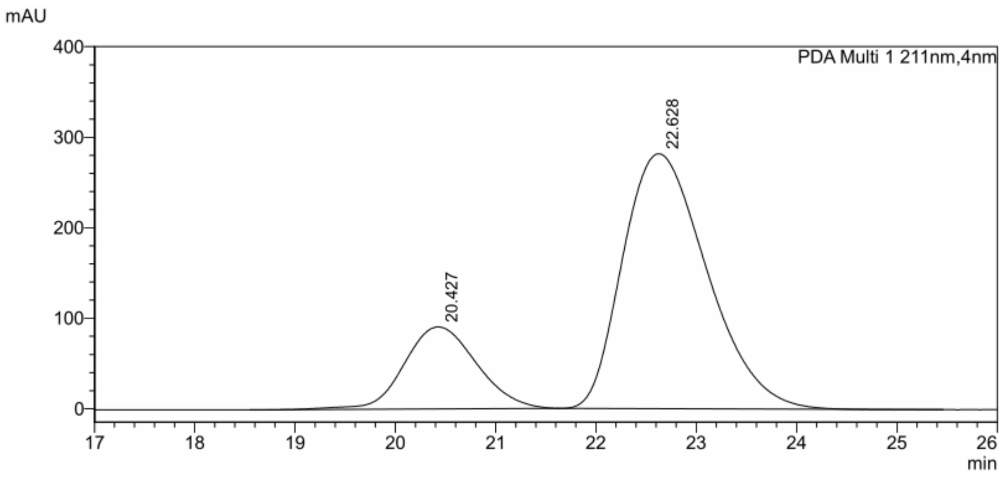

<Peak Table>

PDA Ch1 211nm

Peak#	Ret. Time	Area%
1	20.427	21.678
2	22.628	78.322
Total		100.000

HPLC Data for 13 from maleate, minor diastereomer: Chiralpak OD-H (95:5 hexane:IPA, flow rate 1.00 mL·min^{-1}, 211 nm, 30 °C) t_R (major): 65.4 min, t_R (minor): 78.8 min, 65:35 er.

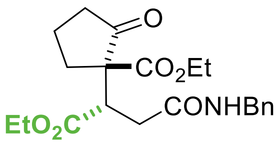

(1*R*,2’*S*)-**13**

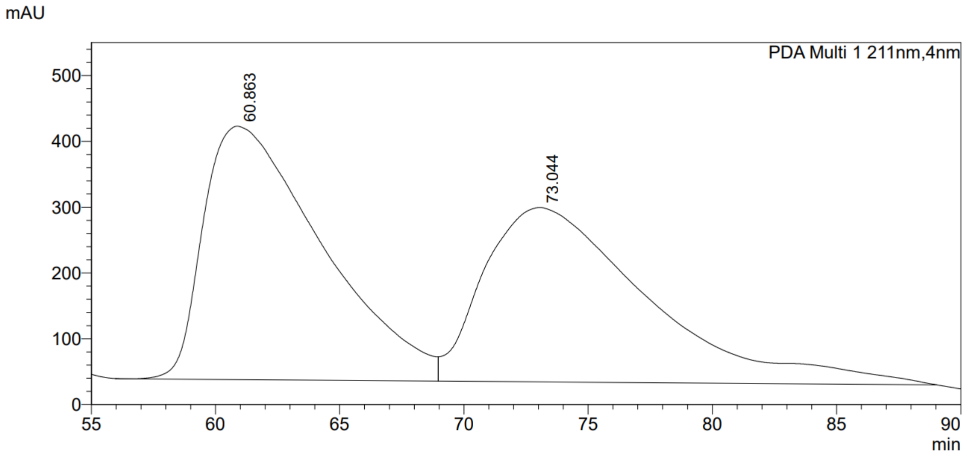

<Peak Table>

PDA Ch1 211nm

Peak#	Ret. Time	Area%
1	60.863	51.165
2	73.044	48.835
Total		100.000

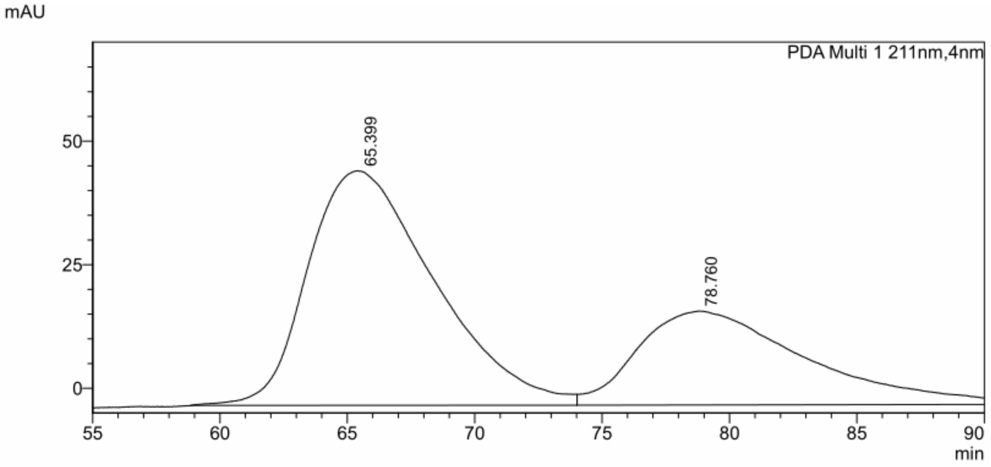

<Peak Table>

PDA Ch1 211nm

Peak#	Ret. Time	Area%
1	65.399	65.107
2	78.760	34.893
Total		100.000

(+)-1-(*tert*-Butyl) 2-ethyl (*R*)-2-((*S*)-4'-(benzylamino)-1',1',1'-trifluoro-4'-oxobutan-2'-yl)-3-oxopyrrolidine-1,2-dicarboxylate (2*R*,2'*S*)-14

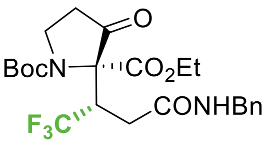

(2*R*,2'*S*)-**14**

The title compound was prepared according to **General Procedure 2**. A mixture of (*E*)-4-nitrophenyl (*E*)-4,4,4-trifluorobut-2-enoate **8** (50 mg, 0.19 mmol), (2*S*,3*R*)-HyperBTM **1** (3.0 mg, 9.5 µmol) and 1-(*tert*-butyl) 2-ethyl 3-oxopyrrolidine-1,2-dicarboxylate (50 mg, 0.19 mmol) in DMA (0.68 mL) was stirred for 16 h at room temperature. Benzylamine (21 µL, 0.19 mmol) was then added, and the reaction was permitted to stir for 1 h. The solution was diluted with Et_2O, washed sequentially with saturated aqueous Na_2CO_3 (×3) and brine, dried over $MgSO_4$ and filtered. The solvent was removed under reduced pressure and the residue was purified by column chromatography (1:3 EtOAc:Hexane) to give a mixture of diastereomers (32.7 mg, 35%, 75:25 d.r.) as a colourless oil.

Data for **14** (analysed as a 75:25 mixture of diastereomers): $[\alpha]_D^{20}$ +31.54 (*c* 0.26 in $CHCl_3$); **IR ν_{max}** (film) / cm^{-1} 3033, 2980, 1771, 1697, 1541, 1381, 1368, 1148, 1013, 941, 773; **^{1}H NMR** (500 MHz, $CDCl_3$, some signals of the minor diastereomer have been obscured by the major and are hence not reported) δ_H: 1.20 (0.75H, t, *J* 7.1, OCH_2CH_3), 1.23 (2.25H, t, *J* 7.1, OCH_2CH_3), 1.45 (6.75H, s, $C(CH_3)_3$), 1.47 (2.25H, s, $C(CH_3)_3$), 2.29 (1H, dd, *J* 16.9, 9.3, $CHCH_AH_BCONH$), 2.69 (1H, ddd, *J* 19.4, 8.9, 5.5, $COCH_AH_BCH_2$), 2.76 (1H, ddd, *J* 19.4, 9.5, 6.1, $COCH_AH_BCH_2$), 3.36 (1H, app dt, *J* 16.8, 1.9, $CHCH_AH_BCONH$), 3.62-3.71 (1H, m, $CH_2CH_AH_BNBoc$), 3.86 (0.25H, app ddd, *J* 10.2, 10.1, 5.6, $CH_2CH_AH_BNBoc$), 3.92 (0.75H, ddd, *J* 10.3, 10.2, 5.7, $CH_2CH_AH_BNBoc$), 4.09 (0.25H, dq, *J* 10.8, 7.1, $OCH_AH_BCH_3$), 4.15 (0.75H, dq, *J* 10.8, 7.1, $OCH_AH_BCH_3$), 4.18 (0.75H, dq, *J* 10.8, 7.1, $OCH_AH_BCH_3$), 4.28 (0.25H, *J* 10.8, 7.1, $OCH_AH_BCH_3$), 4.33 (0.25H, app pent, *J* 10.4, $CHCH_2CONH$), 4.36 (0.25H, dd, *J* 14.8, 5.3, $NHCH_AH_BPh$), 4.44 (0.75H, tdd, *J* 10.5, 9.3, 1.9, $CHCH_2CONH$), 4.46 (0.75H, dd, *J* 14.7, 5.6, $NHCH_AH_BPh$), 4.52 (0.75H, dd, *J* 14.7, 5.7, $NHCH_AH_BPh$), 4.60 (0.25H, dd, *J* 14.9, 6.5, $NHCH_AH_BPh$), 5.94 (0.75H, app t, *J* 5.8, N*H*), 6.32 (0.25H, app t, *J* 6.5, 5.3, N*H*), 7.23-7.30 (3H, $PhC^{2,4,6}H$), 7.30-7.34 (2H, m, $PhC^{3,5}H$); **^{13}C{^{1}H} NMR** (126 MHz, $CDCl_3$) δ_C: 14.1 (OCH_2CH_3 (major and minor)), 28.2 ($C(CH_3)_3$ (major)), 28.3 ($C(CH_3)_3$ (minor)), 31.6 ($CHCH_2CONH$ (major)), 32.0 ($CHCH_2CONH$ (minor)), 35.3 (CH_2CH_2NBoc (major)), 36.1 (CH_2CH_2NBoc (minor)), 41.3 (CH_2CH_2NBoc (major)), 41.7 (CH_2CH_2NBoc (minor)), 42.6 (q, *J* 24.6, $CHCH_2CONH$), 43.8 ($NHCH_2Ph$ (minor)), 43.9 ($NHCH_2Ph$ (major)), 62.8 (OCH_2CH_3 (minor)), 62.9 (OCH_2CH_3 (major)), 68.5_5 (*C*COOEt (major)), 68.6_2 (*C*COOEt (minor)), 81.7 ($C(CH_3)_3$ (minor)), 82.7 ($C(CH_3)_3$ (major)), 127.0 (q, *J* 280.9, CF_3, (major)) 127.4 (PhC^4H (minor)), 127.6 (PhC^4H (major)), 127.8 ($PhC^{2,6}H$ (minor)), 127.8 ($PhC^{2,6}H$ (major)), 128.7 ($PhC^{3,5}H$ (minor)), 128.8 ($PhC^{3,5}H$ (major)), 138.3 (PhC^1 (major)), 138.7 (PhC^1 (minor)), 153.2 (N*C*O^{t}Bu (major), 153.9 (N*C*O^{t}Bu (minor)), 167.2 (*C*OOEt (major), 167.6 (*C*OOEt (minor)), 169.7 (*C*ONHBn (major)), 170.0 (*C*ONHBn (minor)), 206.4 ($COCH_2CH_2$ (minor)), 207.3 ($COCH_2CH_2$ (major)); **^{19}F NMR** (471 MHz, $CDCl_3$) δ_F: −66.02 (0.75F, d, *J* 10.4, CF_3 (minor)), −65.80 (2.25F, d, *J* 10.5, CF_3 (major)); **HRMS** (ESI$^+$) $C_{23}H_{30}F_3N_2O_6$ $[M+H]^+$ found 487.2047, requires 487.2056 (−0.70 ppm).

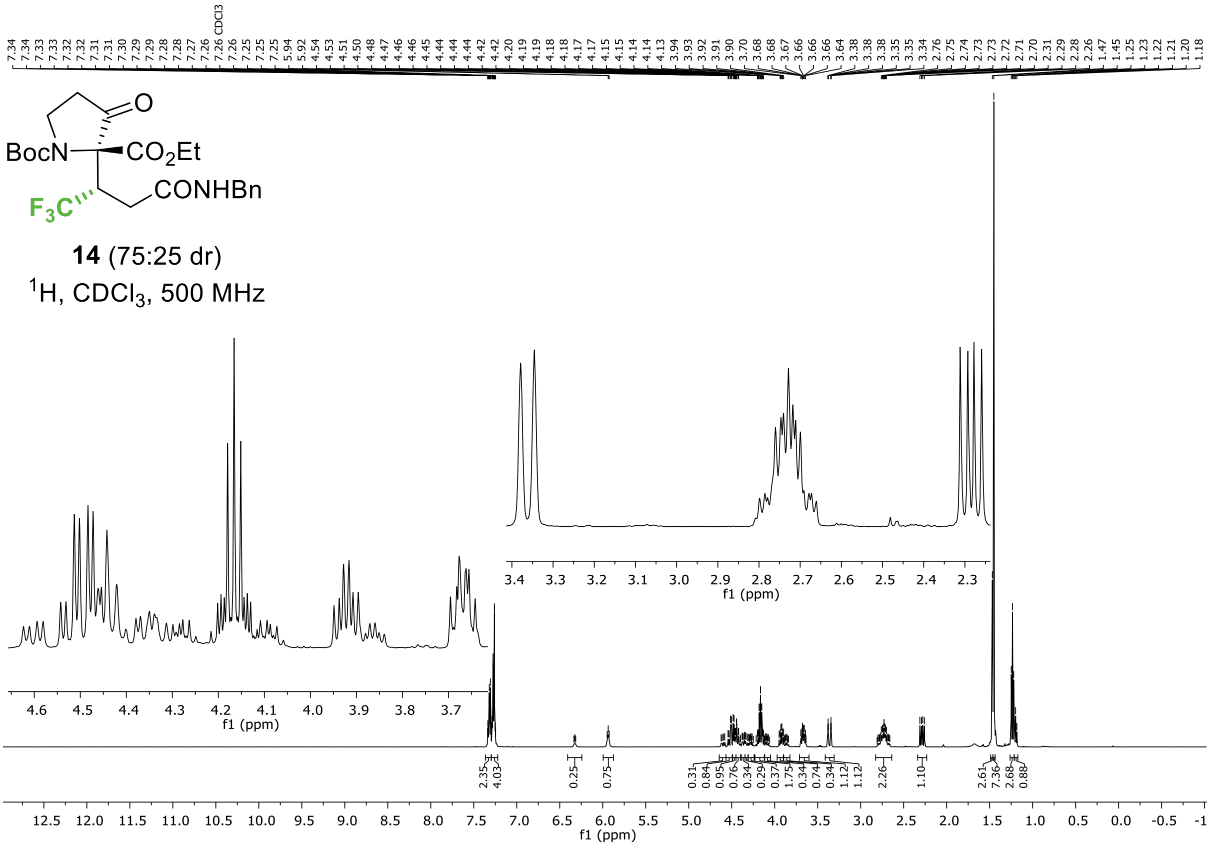

14 (75:25 dr)

^1H, $CDCl_3$, 500 MHz

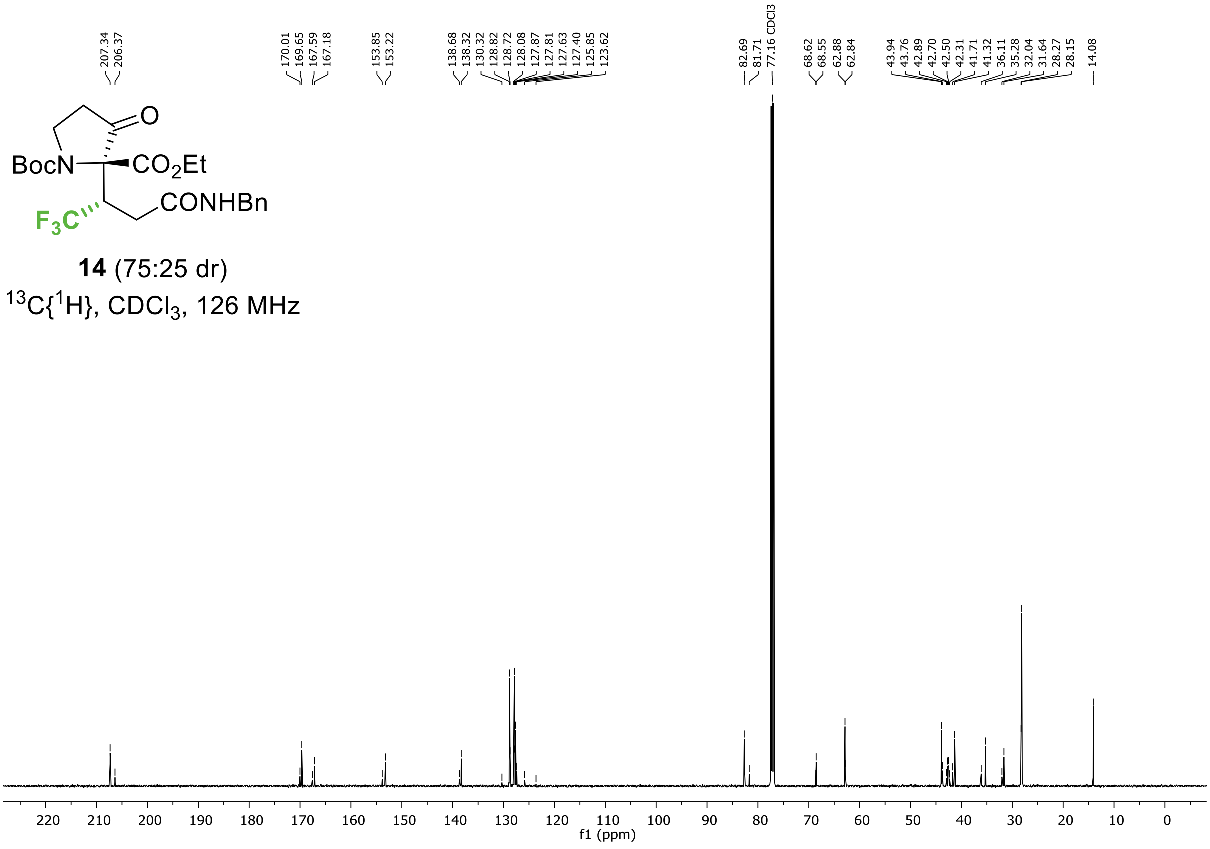

14 (75:25 dr)

$^{13}C\{^1H\}$, $CDCl_3$, 126 MHz

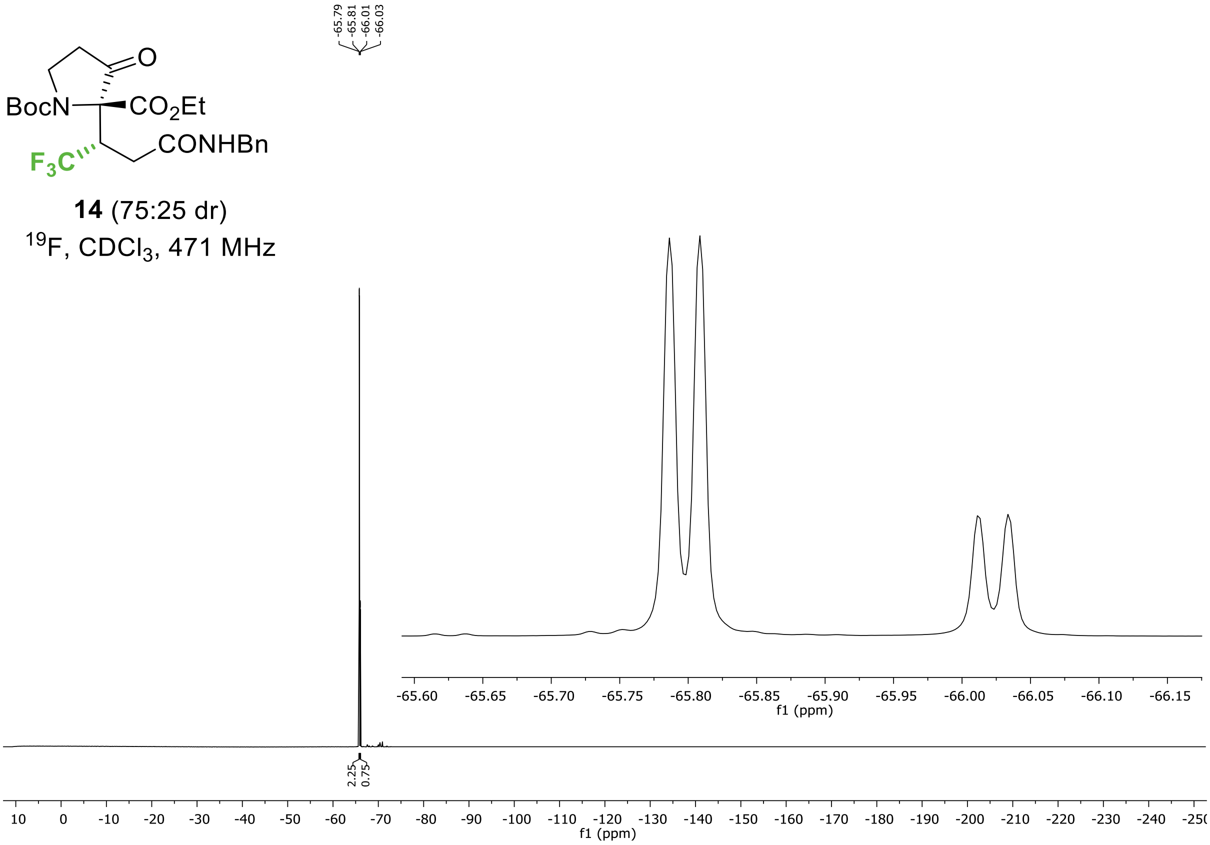

HPLC Data for 14: Chiralpak AS-H (98:2 hexane:^{i}PrOH, flow rate 1.00 mL·min^{-1}, 211 nm, 30 °C) t_R (2*S*,2'*R*)-**14**: 36.7 min, t_R (2*R*,2'*S*)-**14**: 42.8 min, <1:99 er; t_R *rel*-(2*S*,2'*S*): 62.3 min, t_R *rel*-(2*R*,2'*R*)-**14**: 86.3 min,41:59 er.

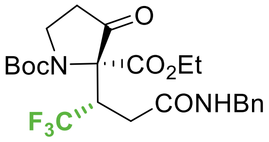

(2*R*,2'*S*)-**14**

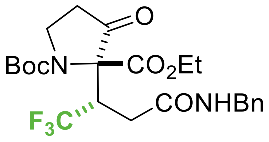

rel-(2*S*,2'*S*)-**14**

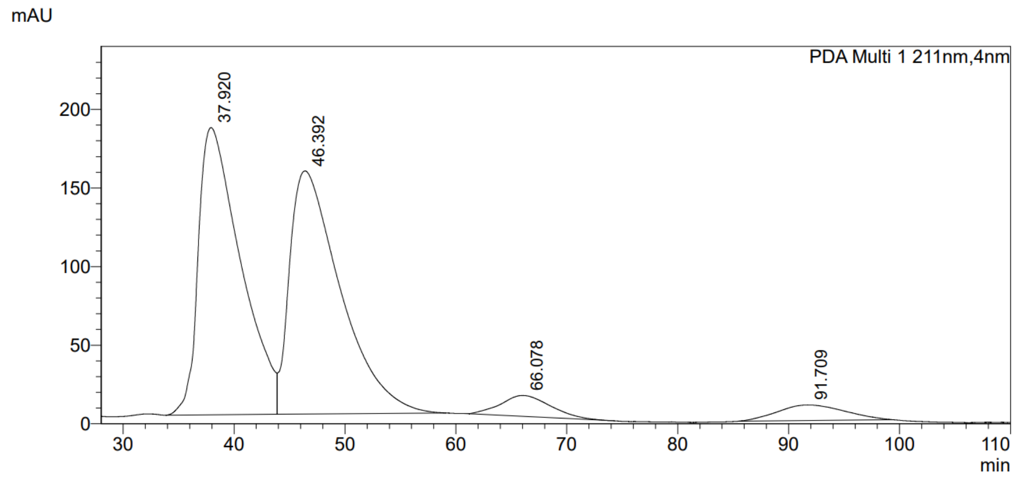

<Peak Table>

PDA Ch1 211nm

Peak#	Ret. Time	Area%
1	37.920	45.640
2	46.392	46.532
3	66.078	3.926
4	91.709	3.903
Total		100.000

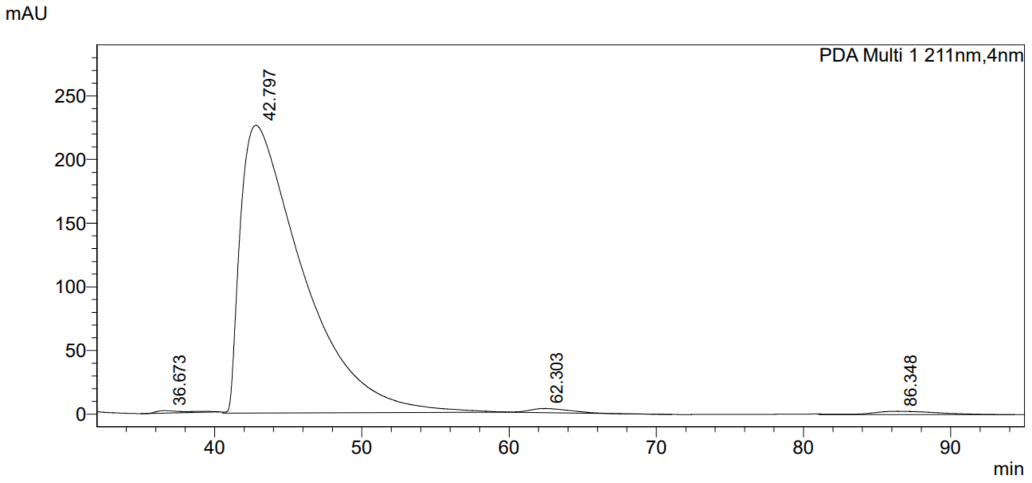

<Peak Table>

PDA Ch1 211nm

Peak#	Ret. Time	Area%
1	36.673	0.375
2	42.797	97.514
3	62.303	0.872
4	86.348	1.239
Total		100.000

(+)-1-(*tert*-Butyl) 3-ethyl (*R*)-3-((*S*)-4'-(benzylamino)-1',1',1'-trifluoro-4'-oxobutan-2'-yl)-4-oxopyrrolidine-1,3-dicarboxylate (3*R*,2'*S*)-15

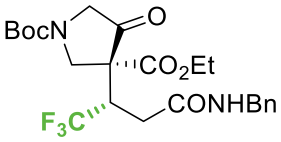

(3*R*,2'*S*)-**15**

The title compound was prepared according to **General Procedure 2**. A mixture of (*E*)-4-nitrophenyl (*E*)-4,4,4-trifluorobut-2-enoate **8** (80 mg, 0.3 mmol), (2*S*,3*R*)-HyperBTM **1** (4.6 mg, 15 µmol) and 1-(*tert*-butyl) 3-ethyl 4-oxopyrrolidine-1,3-dicarboxylate (77 mg, 0.3 mmol) in DMA (1.2 mL) was stirred for 16 h at room temperature. Benzylamine (32 µL, 0.3 mmol) was added and the reaction was permitted to stir for 1 h. The solution was diluted with Et_2O, washed sequentially with saturated aqueous Na_2CO_3 (×3) and brine, dried over $MgSO_4$ and filtered. The solvent was removed under reduced pressure and the residue was purified by column chromatography (1:3 EtOAc:Hexane) to give a mixture of diastereomers (78.9 mg, 54%, 83:17 d.r.) as a colourless oil.

Data for **15**: $[\alpha]_D^{20}$ +2.00 (*c* 0.65 in $CHCl_3$); **IR ν_{max}** (film) / cm^{-1} 3319, 2980, 2367, 1775, 1734, 1701, 1657, 1406, 1217, 1121, 1015, 885, 768; **^{1}H NMR** (500 MHz, $CDCl_3$, analysed as mixture of diastereomers and rotamers, 63:20:17 major (rot) : major (rot) : minor) δ_H: 1.23$_7$ (1.89H, t, *J* 7.1, OCH_2CH_3), 1.24$_3$ (0.51H t, *J* 7.1 Hz, OCH_2CH_3), 1.25 (0.60H, t, *J* 7.0 Hz, OCH_2CH_3), 1.47 (9H, s, $C(CH_3)_3$), 2.28 (0.17H, dd, *J* 15.7, 3.4, $CHCH_AH_BCONH$), 2.49-2.67 (0.37H, m, $CHCH_AH_BCONH$), 2.54 (0.63H, dd, *J* 16.5, 6.4, $CHCH_AH_BCONH$), 2.73-2.90 (0.83H, m, $CHCH_AH_BCONH$), 3.64 (0.17H, d, *J* 12.1, CCH_AH_BNBoc), 3.68-4.08 (4.49H, m, CCH_2NBoc, $COCH_2NBoc$, $CHCH_2CONH$ (major)), 4.07-4.26 (1.11H, m, OCH_2CH_3, $CHCH_2CONH$), 4.17 (1.26H, q, *J* 7.1, OCH_2CH_3), 4.38-4.54 (2.17H, m, $NHCH_2Ph$, CCH_AH_BNBoc), 5.99-6.14 (1H, m, N*H*), 7.23-7.29 (3H, m, $PhC^{2,4,6}H$), 2.29-2.35 (2H, m, $PhC^{3,5}H$); **^{13}C{^{1}H} NMR** (126 MHz, $CDCl_3$) δ_C: 13.9 (OCH_2CH_3 (major)), 14.2 (OCH_2CH_3 (minor)), 28.4 ($C(CH_3)_3$ (major and minor)), 32.2 ($CHCH_2CONH$ (minor)), 32.5 ($CHCH_2CONH$ (major)), 41.8 (q, *J* 26.0, $CHCH_2CONH$ (minor)), 42.7 (q, *J* 26.8, $CHCH_2CONH$ (major)), 44.0 ($NHCH_2Ph$ (major)), 48.8 and 48.3 (rotameric CCH_2NBoc (minor)), 49.3 and 50.0 (rotameric CC_HNBoc (major), $NHCH_2Bn$ (minor)), 51.8 and 52.4 (rotameric, $COCH_2NBoc$ (major)), 60.2 and 60.7 (rotameric, CCH_2CH_2 (major)), 60.5 (CCH_2CH_2 (minor)), 63.2 (OCH_2CH_3 (major)), 63.4 (OCH_2CH_3 (minor)), 80.9 ($COC(CH_3)_3$ (minor)), 81.1 ($COC(CH_3)_3$ (major)), 126.5 (q, *J* 281.8, CF_3, major), 127.7$_1$ (PhC^4H (major)), 127.7$_4$ (PhC^4H (minor)), 127.9$_1$ ($PhC^{2,6}H$ (major)), 127.9$_3$ ($PhC^{2,6}H$), 128.8 ($PhC^{3,5}H$ (major and minor)), 137.8 (PhC^1 (minor)), 137.9 (PhC^1 (major)), 154.0 ($NCOC(CH_3)_3$ (major)), 154.1 ($NCOC(CH_3)_3$ (minor)), 166.6 (*C*OOEt (major and minor)), 168.4 (*C*ONHBn (minor)), 168.7 (*C*ONHBn (major)), 203.4 and 203.9 (rotameric, $COCH_2NBoc$ (major)), 203.6 ($COCH_2NBoc$ (minor)) - CF_3 quartet not resolved for minor diastereomer; **^{19}F NMR** (471 MHz, $CDCl_3$) δ_F: −65.97 (0.51F, dd,

J 127.0, 9.7, C*F*$_3$ (minor)), −64.25 (2.49F, dd, *J* 134.3, 9.6, C*F*$_3$ (major)). **HRMS** (ESI$^+$) $C_{23}H_{29}F_3N_2O_6Na$ [M+Na]$^+$ found 509.1870, requires 509.1874 (+0.80 ppm).

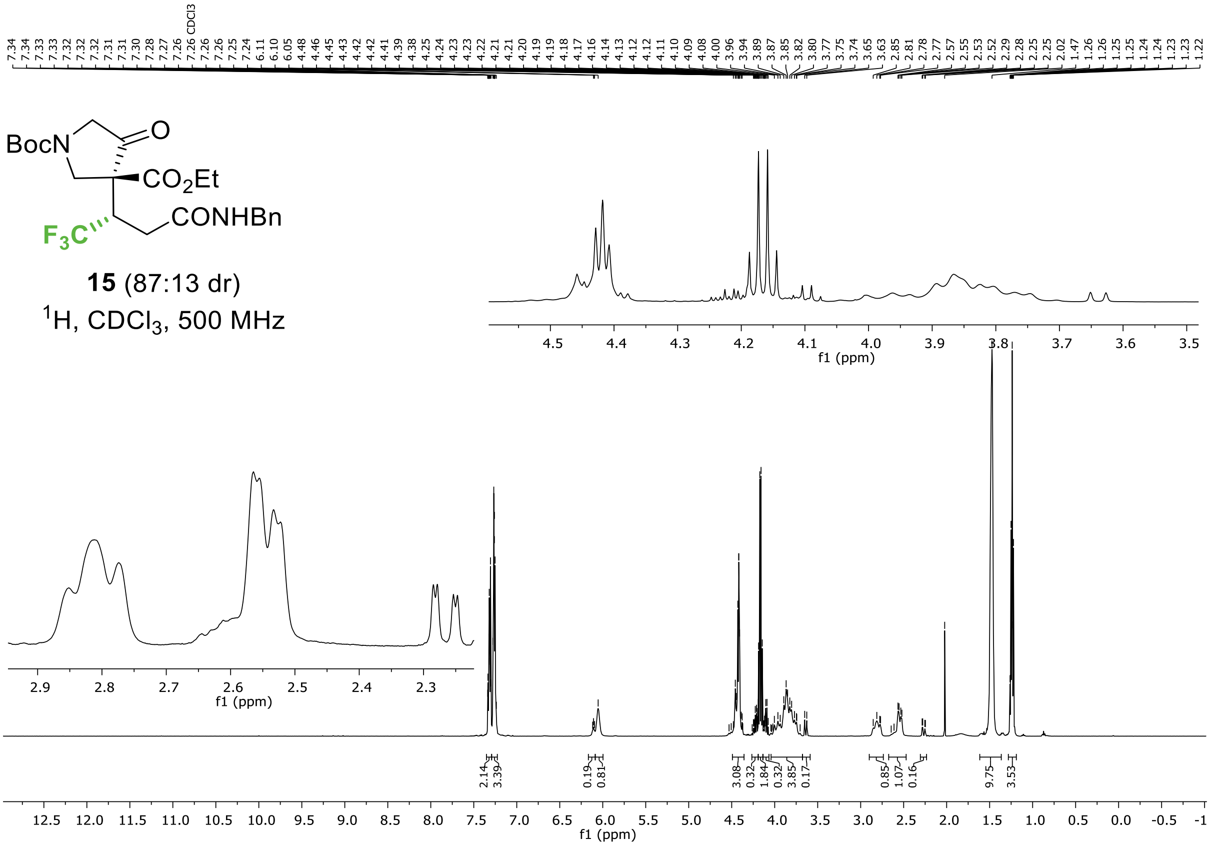

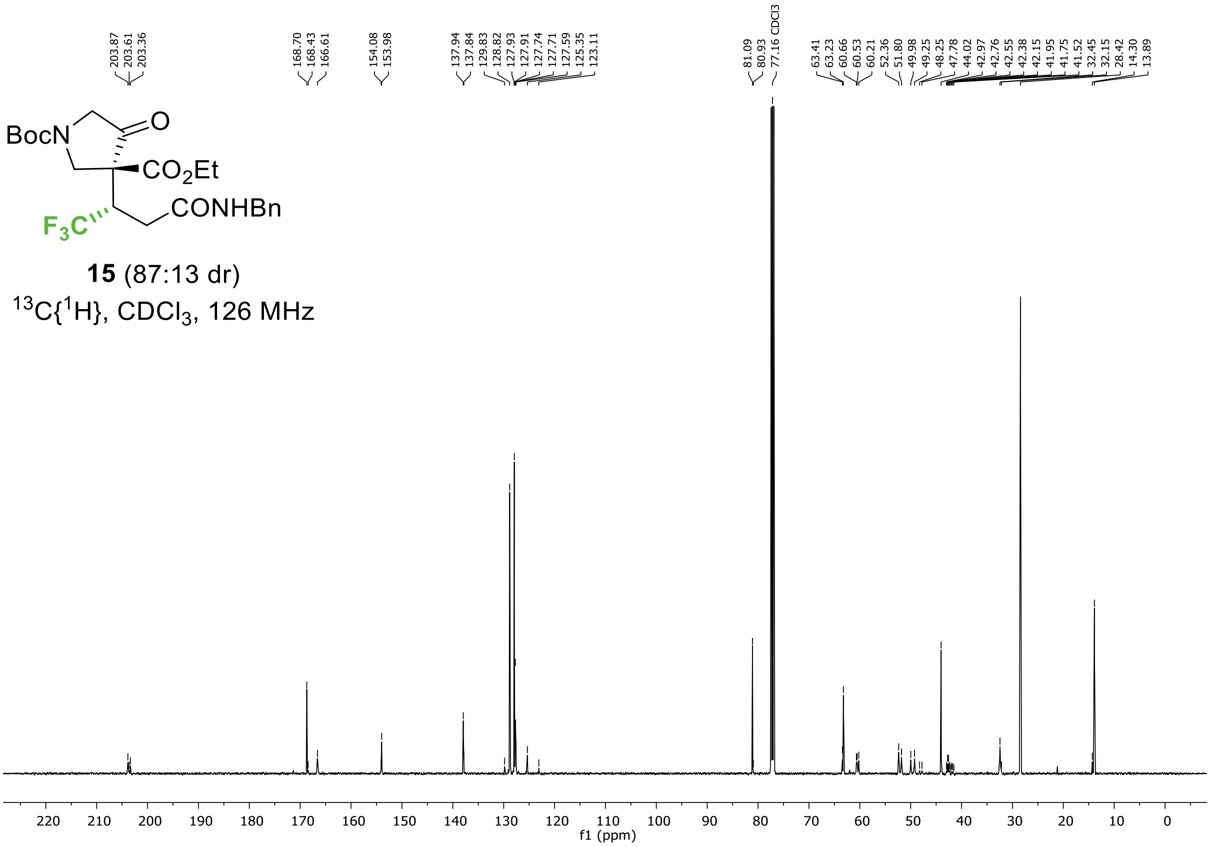

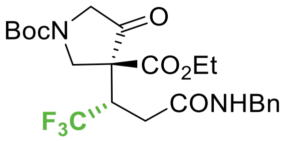

15 (87:13 dr)
^{19}F, $CDCl_3$, 471 MHz

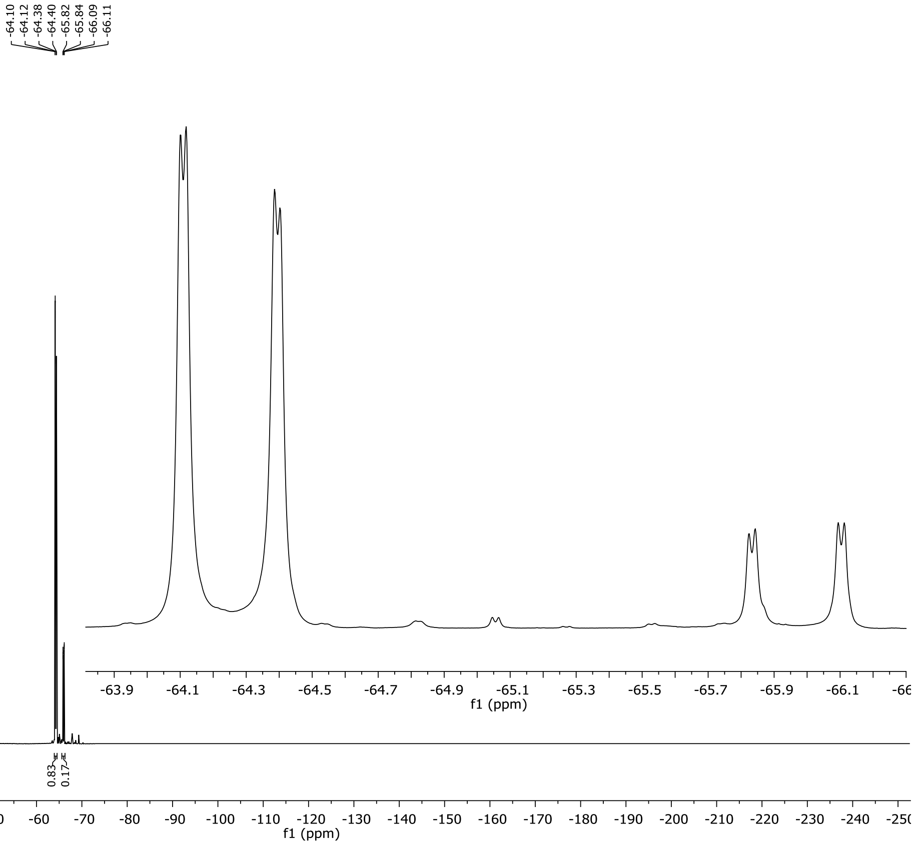

HPLC Data for 15: Chiralpak AS-H (98:2 hexane:^{i}PrOH, flow rate 1.00 mL·min^{-1}, 211 nm, 30 °C) t_R (3*R*,2'*S*)-**15**: 23.4 min, t_R (3*S*,2'*R*)-**15**: 39.0 min, 87:13 er; t_R *rel*-(3*R*,2'*R*)-**15**: 51.1 min, t_R *rel*-(3*S*,2'*S*)-**15**: 62.4 min, 3:97 er.

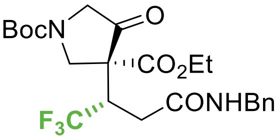

(3*S*,2'*S*)-**15**

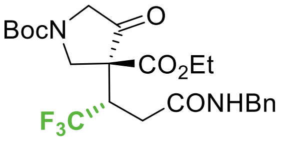

rel-(3*R*,2'*S*)-**15**

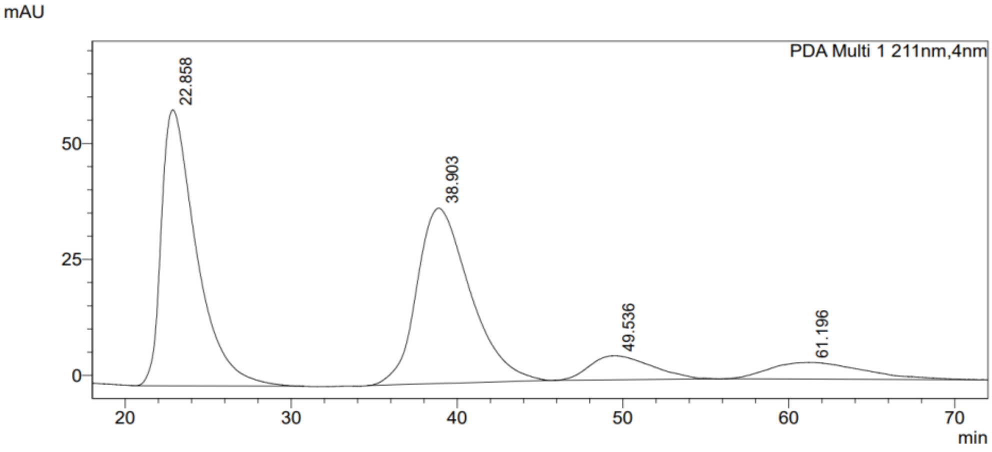

<Peak Table>

PDA Ch1 211nm

Peak#	Ret. Time	Area%
1	22.858	43.852
2	38.903	42.242
3	49.536	6.741
4	61.196	7.166
Total		100.000

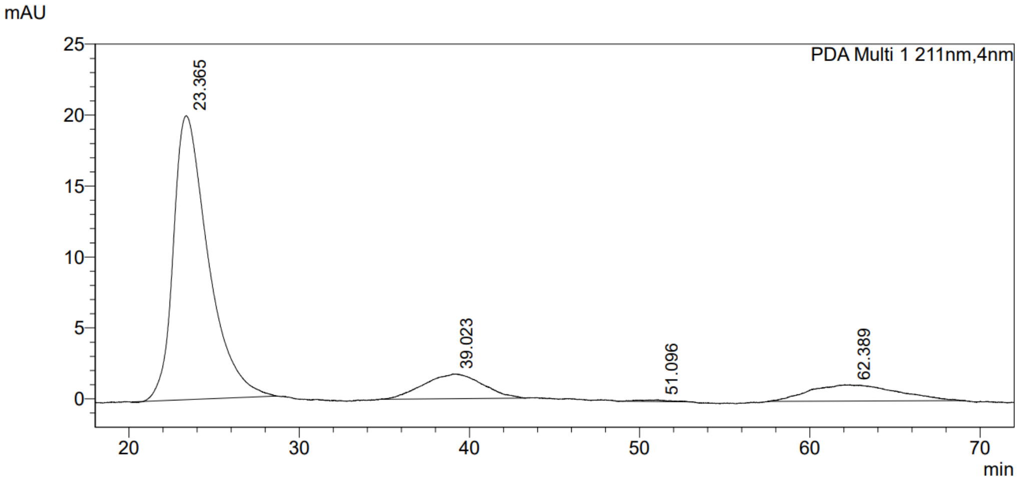

<Peak Table>

PDA Ch1 211nm

Peak#	Ret. Time	Area%
1	23.365	77.163
2	39.023	11.269
3	51.096	0.333
4	62.389	11.235
Total		100.000

(+)-Ethyl (*S*)-2-((*S*)-4’-(benzylamino)-1’,1’,1’-trifluoro-4’-oxobutan-2’-yl)-1-oxo-2,3-dihydro-1*H*-indene-2-carboxylate (2*S*,2’*S*)-16

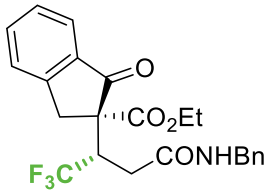

(2*S*,2’*S*)-**16**

The title compound was prepared according to **General Procedure 2**. A mixture of 4-nitrophenyl (*E*)-4,4,4-trifluorobut-2-enoate **8** (261 mg, 1.0 mmol), (2*S*,3*R*)-HyperBTM **1** (15.4 mg, 50 µmol) and methyl 1-oxo-2,3-dihydro-1*H*-indene-2-carboxylate **S4** (204 mg, 1.0 mmol) in DMA (4.0 mL) was stirred for 16 h at room temperature. Benzylamine (110 µL, 1.0 mmol) was added and the reaction was permitted to stir for 1 h. The solution was diluted with Et_2O, washed sequentially with saturated aqueous Na_2CO_3 (×3) and brine, dried over $MgSO_4$ and filtered. The solvent was removed under reduced pressure and the residue was purified by column chromatography (1:3 EtOAc:Hexane) to give the title compound in two fractions (major diastereomer: 220.8 mg, 51% as a colourless oil, and minor diastereomer: 93.4 mg, 22% as a colourless oil); combined (314.2 mg, 73%, 72:28 d.r.).

Data for major diastereoisomer (2*S*,2’*S*)-**16**: $[\alpha]_D^{20}$ +0.65 (*c* 1.00 in $CHCl_3$); **IR** ν_{max} (film) 3304, 2984, 1740, 1717, 1651, 1545, 1275, 1111, 1043, 962, 860; **^{1}H NMR** (500 MHz, $CDCl_3$) δ_H: 1.17 (3H, t, *J* 7.1, OCH_2CH_3), 2.66 (1H, dd, *J* 15.7, 7.8, $CHCH_AH_BCONH$), 2.74 (1H, dd, *J* 15.7, 3.6, $CHCH_AH_BCONH$), 3.32 (1H, d, *J* 17.6, CCH_AH_BAr), 3.83 (1H, d, *J* 17.6, CCH_AH_BAr), 4.06 (1H, qdd, *J* 9.1, 7.7, 3.6, $CHCH_2CONH$), 4.13 (2H, q, *J* 7.1, OCH_2CH_3), 4.42 (1H, dd, J 14.7, 5.7), 4.46 (2H, dd, *J* 14.7, 5.7, $NHCH_2Ph$), 6.30 (1H, app t, *J* 5.8, N*H*), 7.23-7.30 (3H, m, $PhC^{2,4,6}H$), 7.30-7.35 (2H, m, $PhC^{3,5}H$), 7.39 (1H, app td, *J* 7.5, 0.9 ArC^5H), 7.48 (1H, app dt, *J* 7.7, 1.0, PhC^4H), 7.63 (1H, app td, *J* 7.5, 1.3, ArC^5H), 7.75 (1H, app dt, *J* 7.7, 1.0, ArC^7H); **^{13}C{^{1}H} NMR** (126 MHz, $CDCl_3$) δ_C: 13.9 (OCH_2CH_3), 33.7 ($CHCH_2CONH$), 34.2 (CCH_2Ar), 44.0 ($NHCH_2Ph$), 44.1 (q, *J* 26.0, $CHCH_2CONH$), 61.4 (*C*COOEt), 62.8 (OCH_2CH_3), 125.2 (ArC^7H), 126.4 (ArC^4H), 126.8 (q, *J* 280.9, CF_3), 127.6 (PhC^4H), 127.9 ($PhC^{2,6}H$), 128.1 (ArC^6H), 128.8 ($PhC^{3,5}H$), 134.3 (ArC^{7a}), 135.8 (ArC^5H), 138.0 (PhC^1), 152.7 (ArC^{3a}), 168.9 (*C*OOEt), 169.1 (*C*ONHBn), 198.6 ($ArC^{7a}CO$); **^{19}F{^{1}H} NMR** (471 MHz, $CDCl_3$) δ_F: −64.11 (CF_3). **HRMS** (ESI^+) $C_{23}H_{23}F_3O_4N$ $[M+H]^+$ found 434.1568, requires 434.1574 (–1.3 ppm).

Data for minor diastereoisomer rel-(2*R*,2’*S*)-**16**: $[\alpha]_D^{20}$ −0.108 (*c* 0.5 in $CHCl_3$); **IR** ν_{max} (film) 3285, 3092, 1717, 1651, 1558, 1456, 1373, 1119, 964, 858; **^{1}H NMR** (500 MHz, $CDCl_3$) δ_H: 1.24 (3H, t, *J* 7.1, OCH_2CH_3), 2.06 (1H, dd, *J* 15.5, 2.9, $CHCH_AH_BCONH$), 2.37 (1H, dd, *J* 15.5, 8.8, $CHCH_AH_BCONH$), 3.15 (1H, d, *J* 17.4, CCH_AH_BAr), 3.93 (1H, d, *J* 17.4, CCH_AH_BAr), 4.13 (1H, dq, *J* 10.8, 7.1, $OCH_AH_BCH_3$), 4.20 (1H, dq, *J* 10.8, 7.1, $OCH_AH_BCH_3$), 4.36 (1H, app pd, *J* 8.9, 2.9,

C*H*CH_2CONH), 4.40 (1H, dd, *J* 14.6, 5.7, NHC$H_A$$H_B$Ph), 4.44 (1H, dd, *J* 14.6, 5.7, NHC$H_A$$H_B$Ph), 5.94 (1H, app t, *J* 5.7, N*H*), 7.23-7.28 (3H, m, Ph$C^{2,4,6}$*H*), 7.29-7.34 (2H, m, Ph$C^{2,6}$*H*), 7.39 (1H, app td, *J* 7.5, 1.0, ArC^6*H*), 7.53 (1H, app dt, *J* 7.8, 0.9, ArC^4*H*), 7.65 (1H, app td, *J* 7.5, 1.2, ArC^5*H*), 7.73 (1H, app dt, *J* 7.7, 1.0, ArC^7*H*); **^{13}C{^{1}H} NMR** (126 MHz, $CDCl_3$) δ_C: 13.9 (OCH_2*C*H_3), 32.6 (*C*CH_2Ar), 33.0 (CH*C*H_2CONH), 43.5 (q, *J* 26.4, *C*HCH_2CONH), 44.0 (NH*C*H_2Ph), 62.7 (*C*COOEt), 63.0 (O*C*$H_2$$CH_3$), 125.2 (Ar$C^7$H), 126.6 (Ar$C^4$H), 127.3 (q, *J* 281.7, *C*F_3), 127.7 (PhC^4H), 128.0 (Ph$C^{2,6}$H), 128.2 (ArC^6H), 128.8 (Ph$C^{3,5}$H), 134.1 (ArC^{7a}), 136.3 (ArC^5H), 138.0 (PhC^1), 153.9 (ArC^{3a}), 167.7 (*C*OOEt), 168.4 (*C*ONHBn), 199.0 (ArC^{7a}*C*O); **^{19}F{^{1}H} NMR** (471 MHz, $CDCl_3$) δ_F: −65.80 (C*F*$_3$); **HRMS** (ESI^+) $C_{23}H_{23}F_3O_4N$ $[M+H]^+$ found 434.1568, requires 434.1574 (–1.3 ppm).

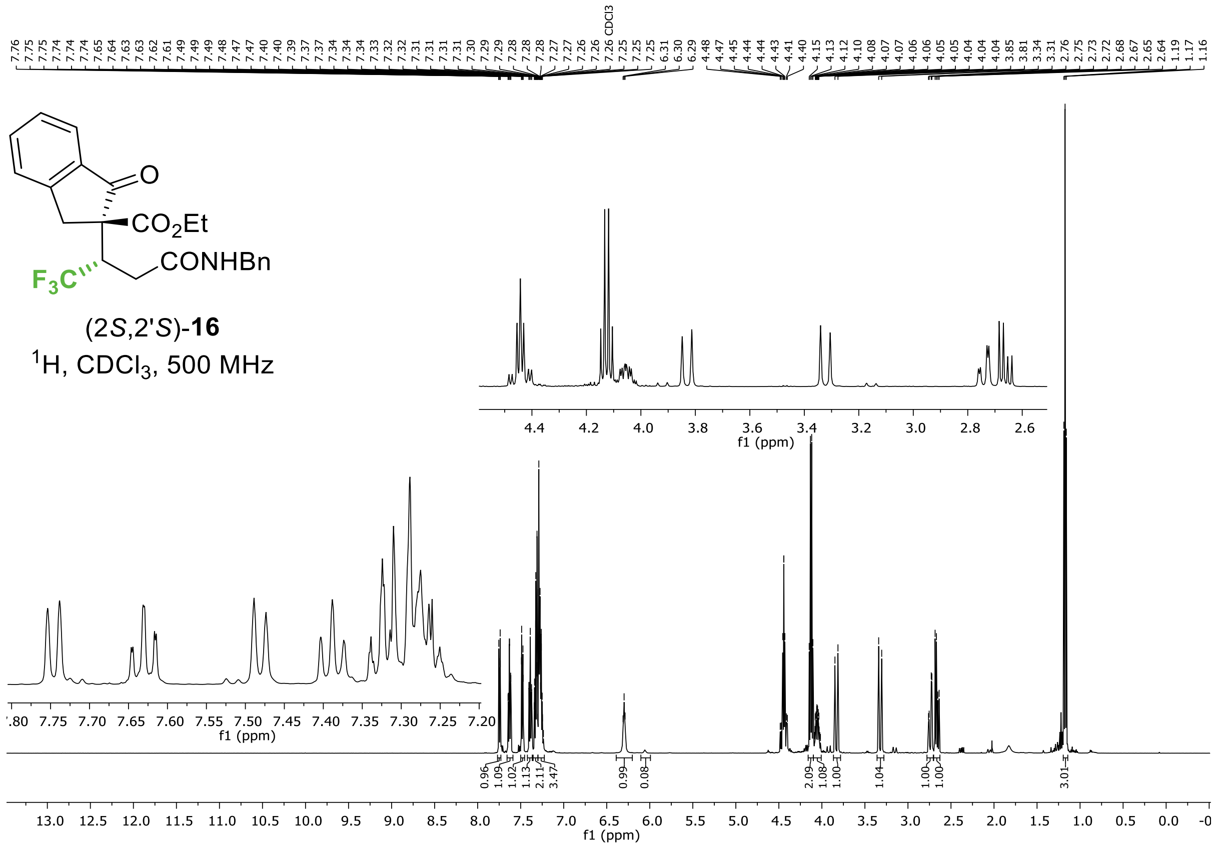

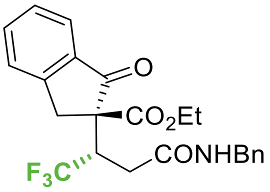

(2*S*,2'*S*)-**16**

$^{13}C\{^{1}H\}$, $CDCl_3$, 126 MHz

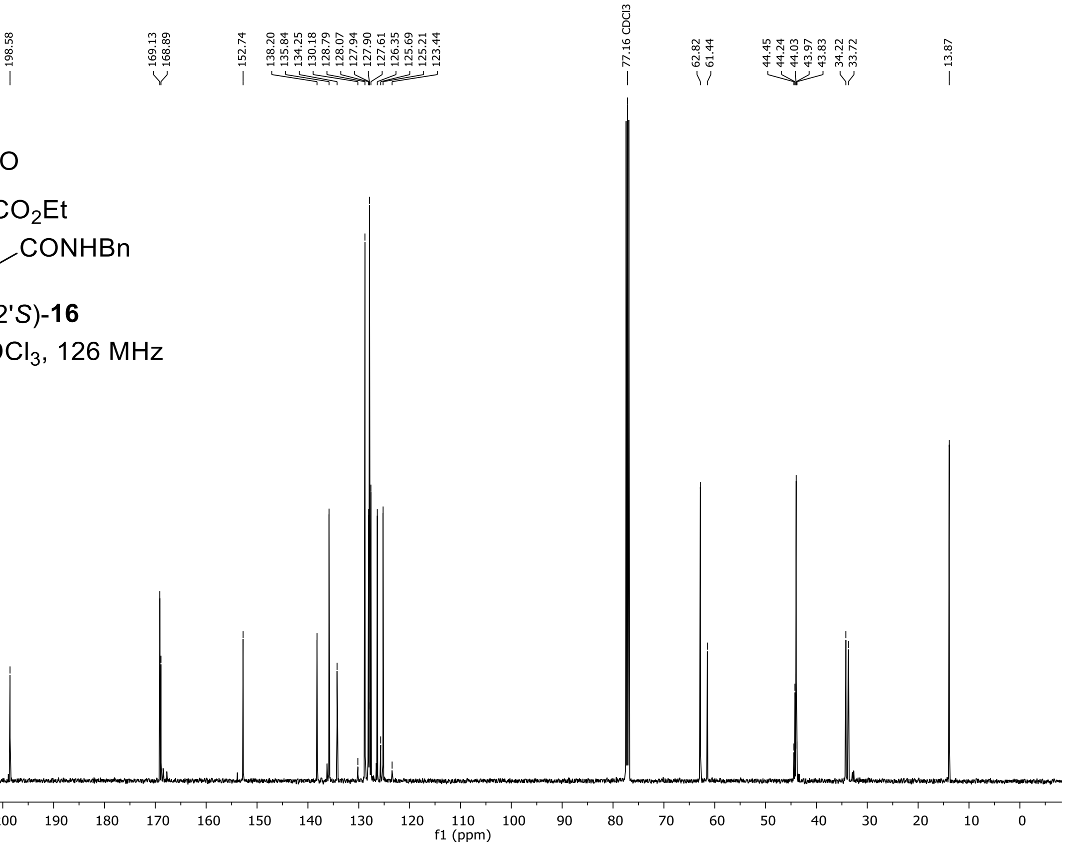

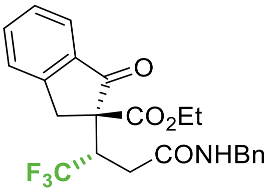

(2*S*,2'*S*)-**16**

$^{19}F\{^{1}H\}$, $CDCl_3$, 471 MHz

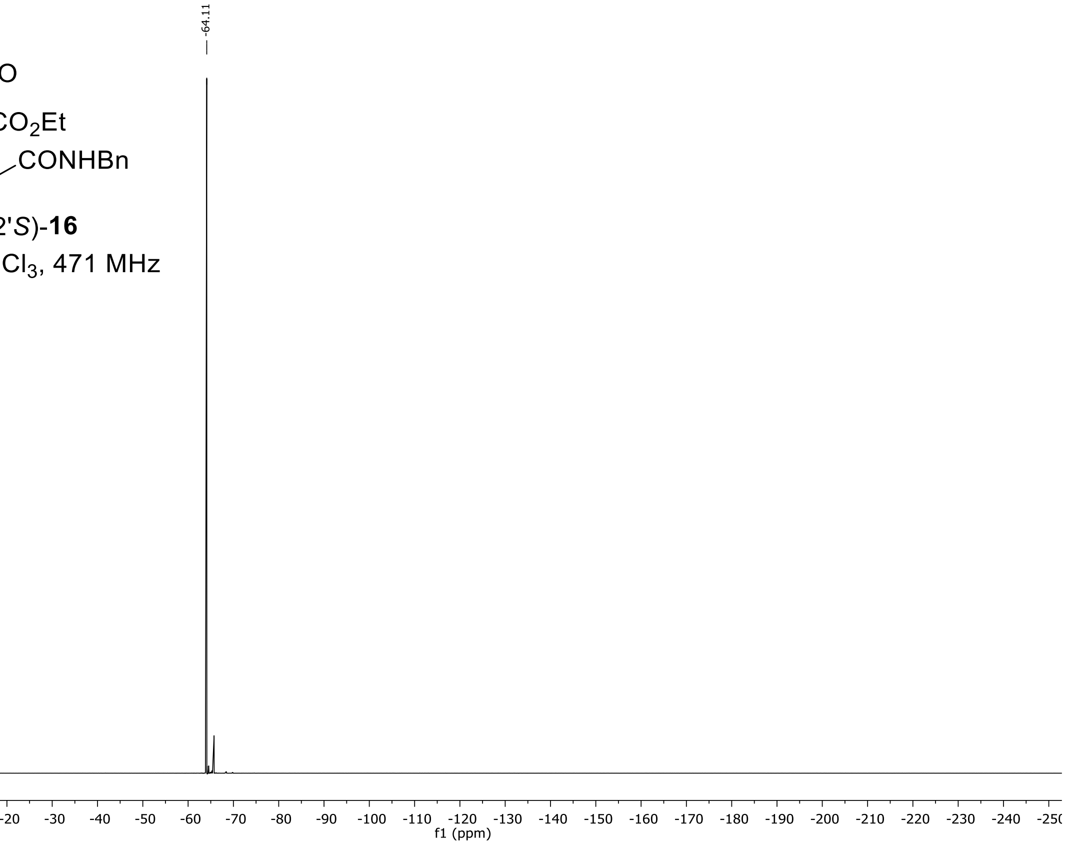

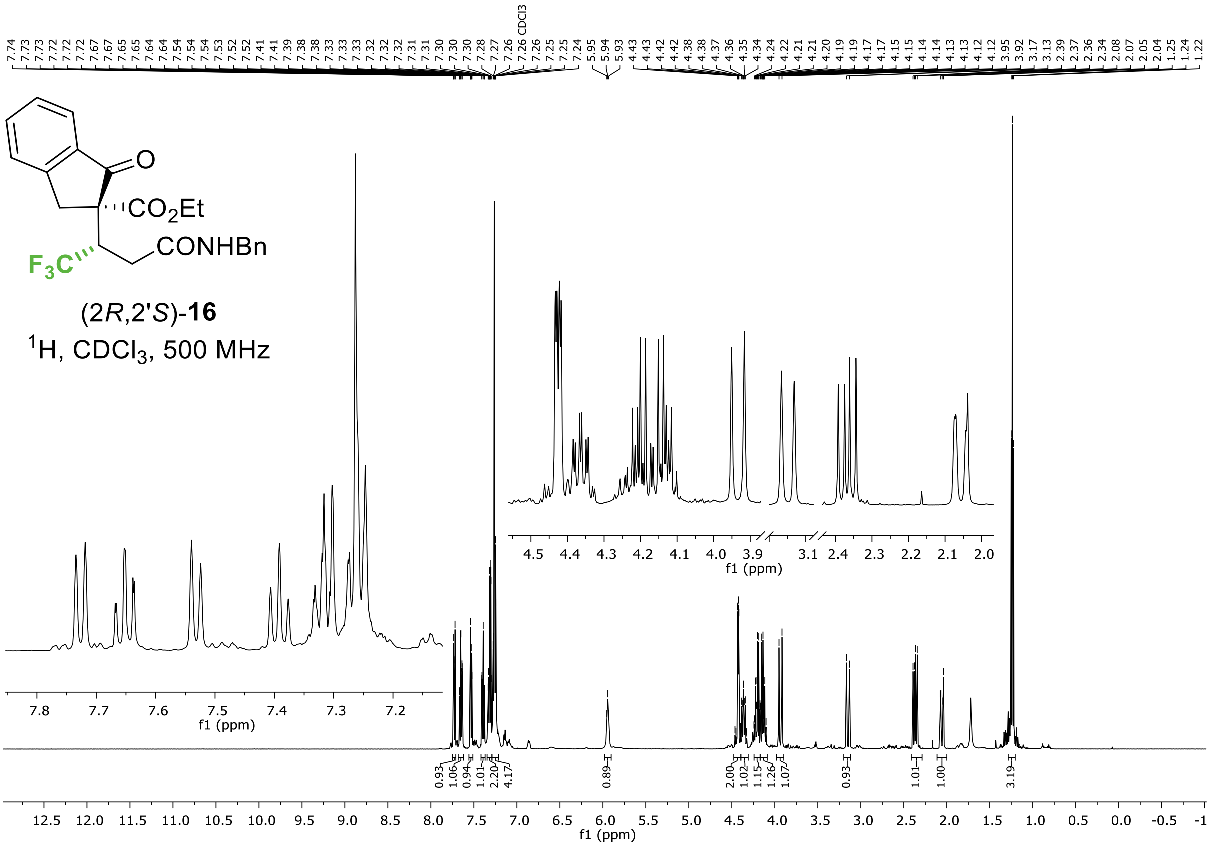
7.74 7.73 7.73 7.72 7.72 7.67 7.67 7.65 7.65 7.64 7.64 7.54 7.54 7.54 7.53 7.52 7.52 7.41 7.39 7.38 7.38 7.33 7.33 7.33 7.32 7.32 7.32 7.31 7.31 7.30 7.30 7.28 7.27 7.26 7.26 CDCl3 7.26 7.25 7.25 7.24 5.95 5.94 5.93 4.43 4.42 4.42 4.38 4.38 4.37 4.36 4.35 4.34 4.24 4.22 4.21 4.21 4.20 4.19 4.19 4.17 4.17 4.15 4.15 4.14 4.14 4.13 4.13 4.12 4.12 3.95 3.92 3.17 3.13 2.39 2.37 2.36 2.34 2.08 2.07 2.05 2.04 1.25 1.24 1.22
O
CO2Et
CONHBn
F3C
(2R,2'S)-16
1H, CDCl3, 500 MHz
0.93 1.06 0.94 1.01 2.20 4.17 0.89 2.00 1.02 1.15 1.26 1.07 0.93 1.01 1.00 3.19
f1 (ppm)

v

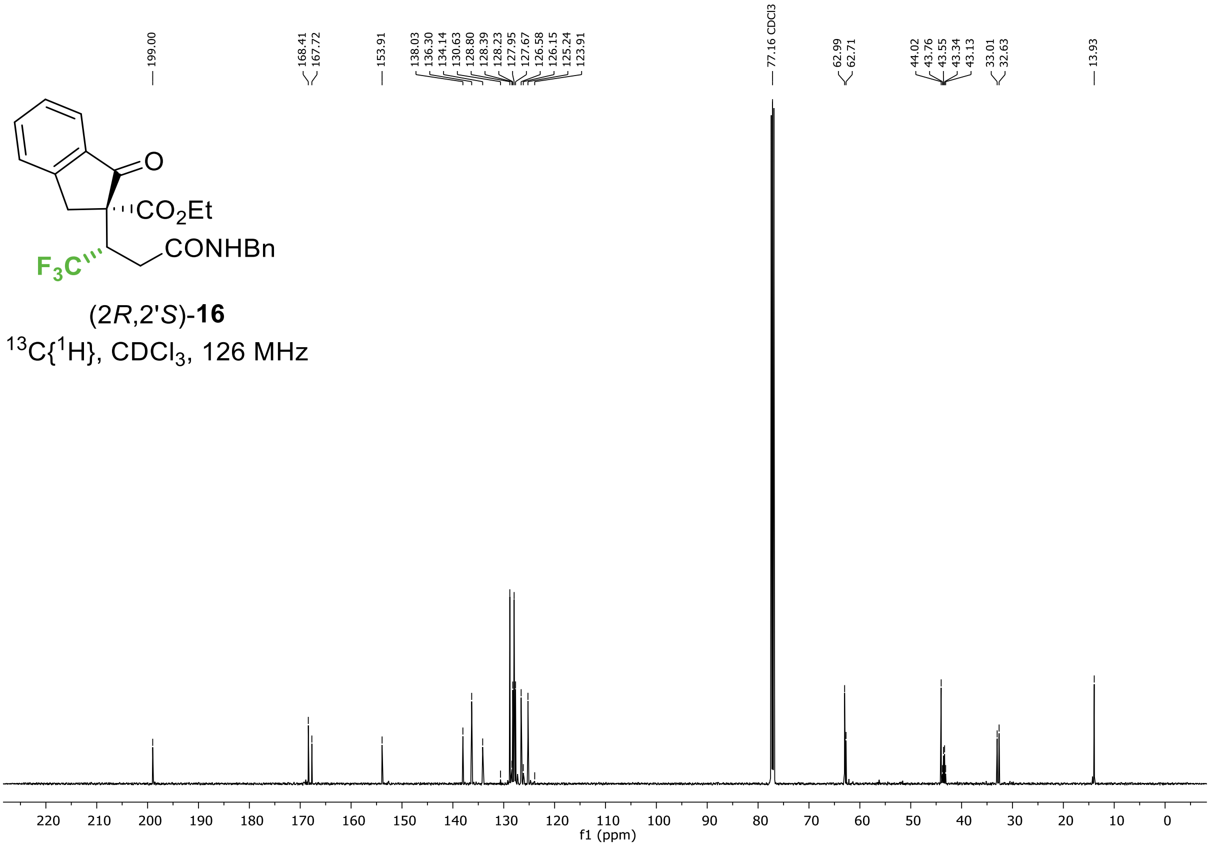
199.00 168.41 167.72 153.91 138.03 136.30 134.14 130.63 128.80 128.39 128.23 127.95 127.67 126.58 126.15 125.24 123.91 77.16 CDCl3 62.99 62.71 44.02 43.76 43.55 43.34 43.13 33.01 32.63 13.93
O
CO2Et
CONHBn
F3C
(2R,2'S)-16
13C{1H}, CDCl3, 126 MHz
f1 (ppm)

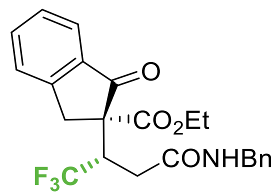

(2*R*,2'*S*)-**16**
$^{19}F\{^{1}H\}$, $CDCl_3$, 471 MHz

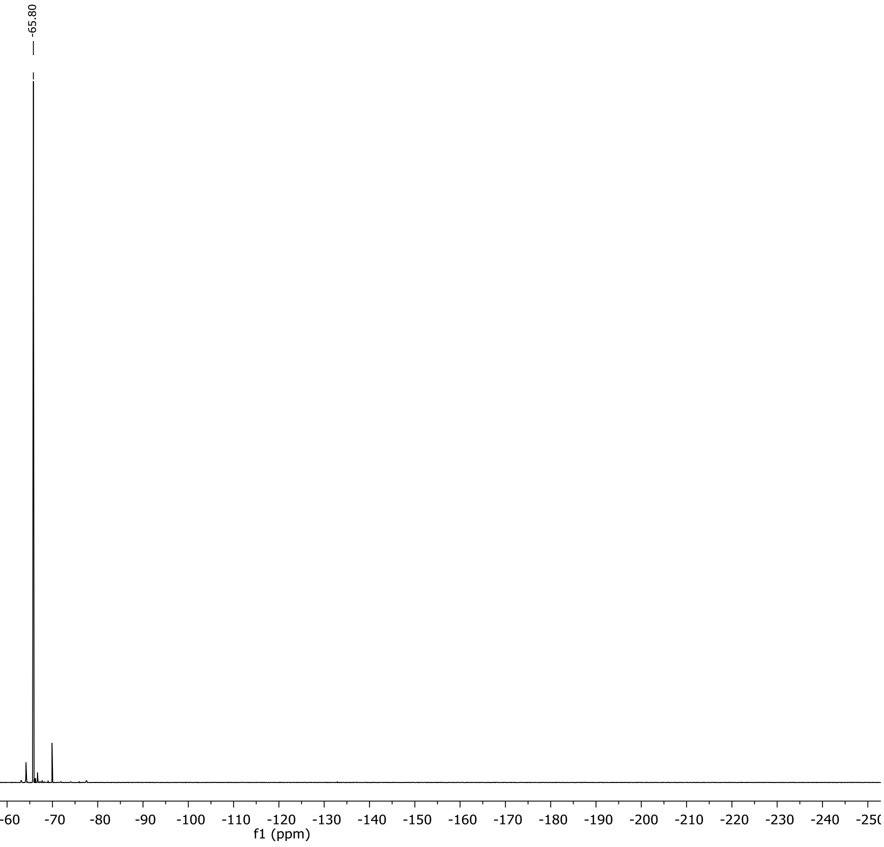

HPLC Data for (2*S*,2'*S*)-16: Chiralpak AD-H (95:5 hexane:^{i}PrOH, flow rate 1.00 mL·min^{-1}, 211 nm, 30 °C) t_R (2*S*,2'*S*)-**16**: 42.1 min, t_R (2*R*,2'*R*)-**16**: 53.1 min, 94:6 er.

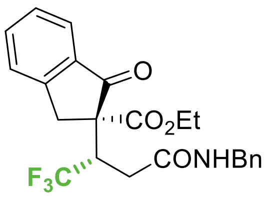

(2*S*,2'*S*)-**16**

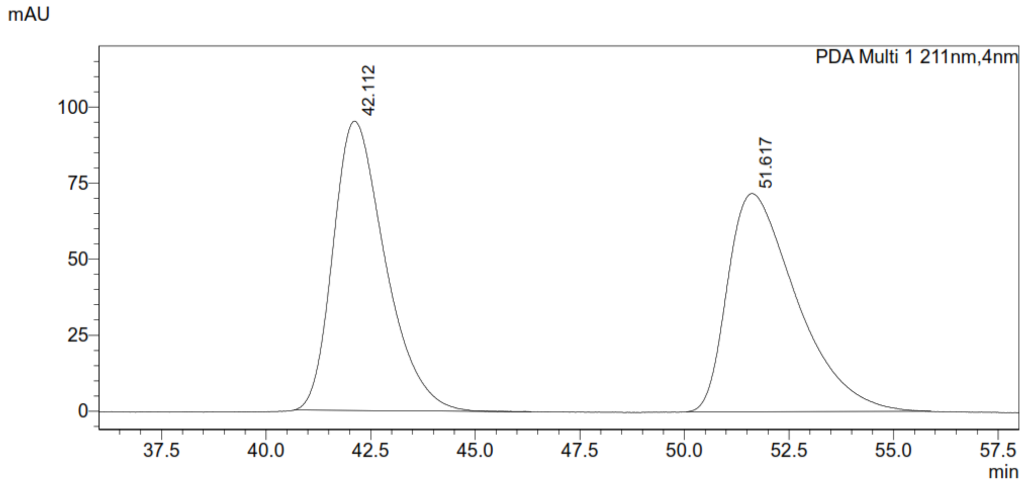

<Peak Table>

PDA Ch1 211nm

Peak#	Ret. Time	Area%
1	42.112	50.118
2	51.617	49.882
Total		100.000

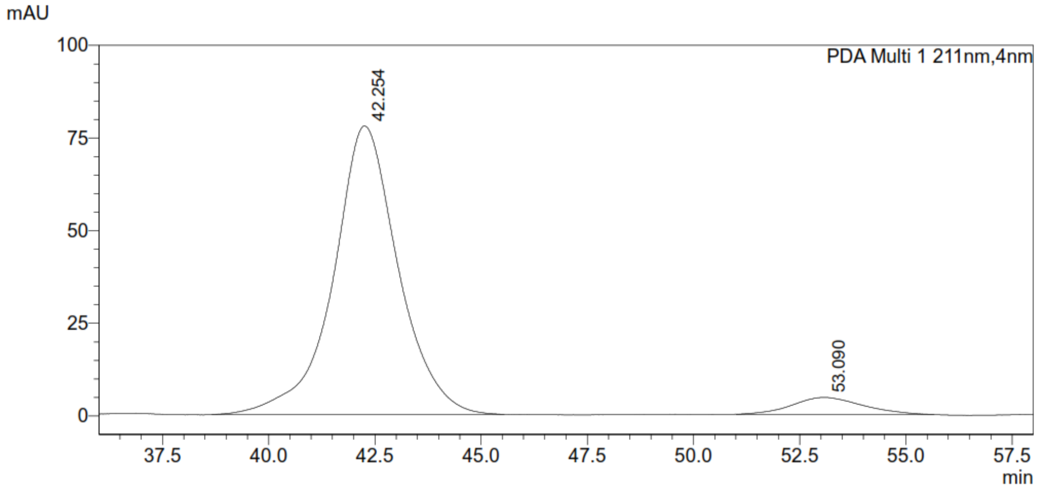

<Peak Table>

PDA Ch1 211nm

Peak#	Ret. Time	Area%
1	42.254	93.933
2	53.090	6.067
Total		100.000

HPLC Data for *rel*-(2*R*,2'*R*)-16: Chiralpak AD-H (90:5 hexane:^{i}PrOH, flow rate 1.00 mL·min^{-1}, 211 nm, 30 °C) t_R *rel*-(2*R*,2'*S*)-**16**: 41.1 min, t_R *rel*-(2*S*,2'*R*)-**16**: 81.6 min, 96:4 er.

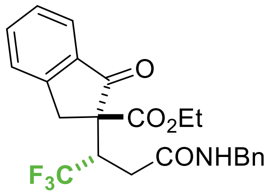

rel-(2*R*,2'*S*)-**16**

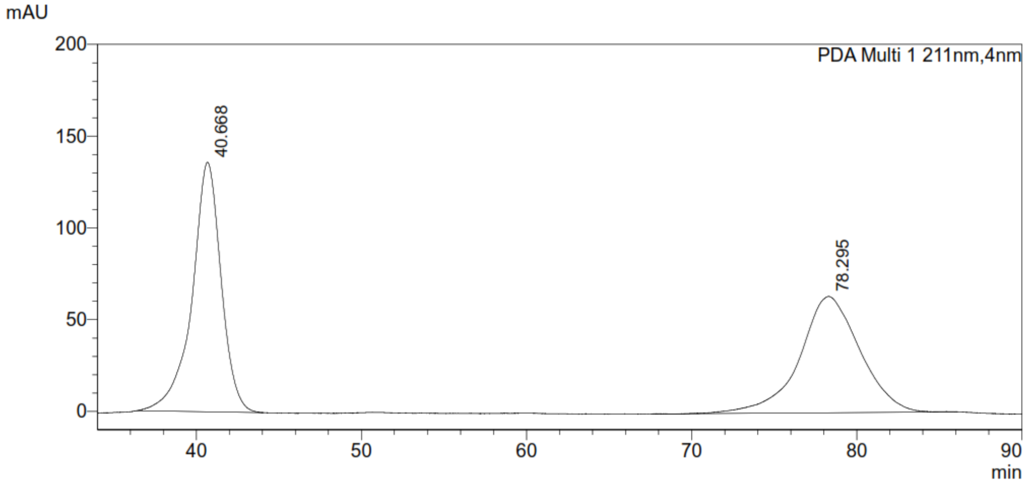

<Peak Table>

PDA Ch1 211nm

Peak#	Ret. Time	Area%
1	40.668	50.842
2	78.295	49.158
Total		100.000

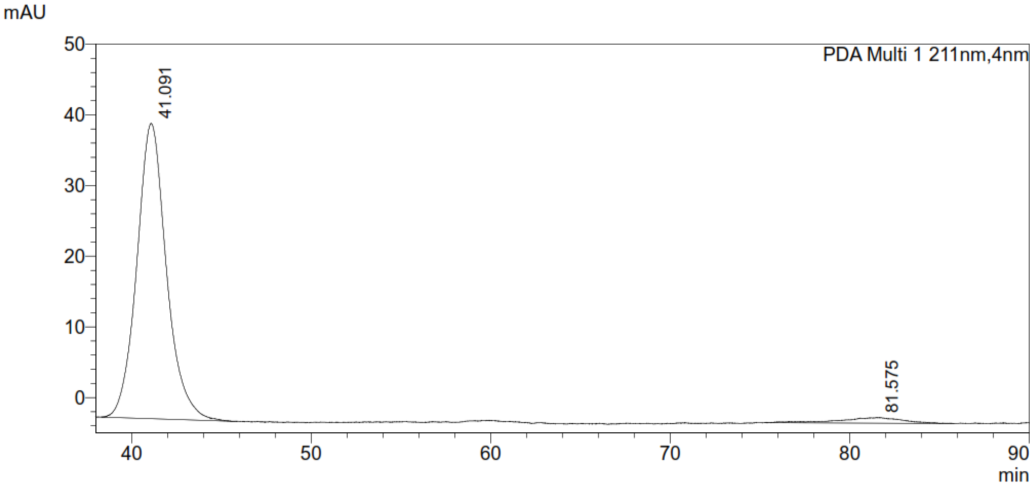

<Peak Table>

PDA Ch1 211nm

Peak#	Ret. Time	Area%
1	41.091	96.014
2	81.575	3.986
Total		100.000

(+)-Ethyl (*R*)-2-((*S*)-4'-(benzylamino)-1',1',1'-trifluoro-4'-oxobutan-2'-yl)-3-oxo-2,3-dihydrobenzofuran-2-carboxylate (2*R*,2'*S*)-17

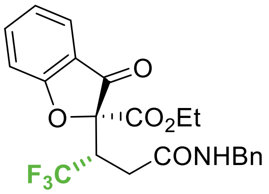

(2*R*,2'*S*)-**17**

The title compound was prepared according to **General Procedure 2**. A mixture of 4-nitrophenyl (*E*)-4,4,4-trifluorobut-2-enoate **8** (80 mg, 0.3 mmol), (2*S*,3*R*)-HyperBTM **1** (4.6 mg, 15 µmol) and methyl 3-oxo-2,3-dihydrobenzofuran-2-carboxylate S**5** (63 mg, 0.3 mmol) in DMA (1.2 mL) was stirred for 16 h at room temperature. Benzylamine (32 µL, 0.3 mmol) was added, and the reaction was permitted to stir for 1 h. The solution was diluted with Et_2O, washed sequentially with saturated aqueous Na_2CO_3 (×3) and brine, dried over $MgSO_4$ and filtered. The solvent was removed under reduced pressure and the residue was purified by column chromatography (1:3 EtOAc:Hexane) to give the title compound (91.3 mg, 69%, 85:15 d.r.) as a mixture of diastereomers as a white solid.

Data for **17** (analysed as 85:15 mixture of diastereomers): **mp** 65-70 °C; $[\alpha]_D^{20}$ +42.6 (*c* 0.5 in $CHCl_3$); **IR** ν_{max} (film) 3389, 3304, 2984, 1751, 1732, 1653, 1611, 1545, 1462, 1227, 1125, 1026, 891, 856; ^{1}**H NMR** (500 MHz, $CDCl_3$) δ_H: 1.26 (2.55H, t, *J* 7.1, OCH_2CH_3), 1.28 (0.45H, t, *J* 7.1, OCH_2CH_3), 2.21 (0.15H, dd, *J* 15.8, 4.0, $CHCH_AH_BCONH$), 2.41 (0.15H, dd, *J* 15.8, 8.3, $CHCH_AH_BCONH$), 2.72 (0.85H, ddd, *J* 16.2, 7.7, 2.5, $CHCH_AH_BCONH$), 2.83 (0.85H, dd, *J* 16.2, 4.0, $CHCH_AH_BCONH$), 4.15-4.30 (2.85H, m, OCH_2CH_3 (major and minor), $CHCH_2CONH$ (major)), 4.33-4.41 (0.45H, m, $CHCH_2CONHCH_2Ph$), 4.41 (0.85H, dd, *J* 14.7, 5.4, $NHCH_AH_BPh$), 4.51 (0.85H, dd, *J* 14.7, 5.9, $NHCH_AH_BPh$), 5.68 (0.15H, app t, *J* 5.0, N*H*), 5.96 (0.85H, app t, *J* 5.8, N*H*), 7.14 (0.15H, app td, *J* 7.4, ArC^5H), 7.16 (0.85H, app t, *J* 7.4, ArC^5H), 7.20-7.24 (1.00H, m, ArC^7H), 7.25-7.30 (3.00H, m, $PhC^{2,4,6}H$), 7.30-7.36 (2.00H, m, $PhC^{3,5}H$), 7.61 (0.15H, dd, *J* 7.8, 1.4, ArC^4H), 7.67 (0.85H, app ddd, *J* 7.7, 7.1, 1.5, ArC^6H), 7.68_2 (0.85H, d, *J* 7.6, ArC^4H), 7.68_5 (0.15H, ap td, *J* 8.3, 7.3, 1.5, ArC^6H); ^{13}**C{^{1}H} NMR** (126 MHz, $CDCl_3$) δ_C: 14.0 (OCH_2CH_3 (major and minor)), 31.1 ($CHCH_2CONH$ (minor)), 32.2 ($CHCH_2CONH$ (major)), 44.0 (q, *J* 27.0, $CHCH_2CONH$ (major)), 44.1 ($NHCH_2Ph$ (major and minor)), 44.2 (q, *J* 27.0, $CHCH_2CONH$ (minor)), 63.7 (OCH_2CH_3 (minor)), 63.8 (OCH_2CH_3 (major)), 89.2 (*C*COOEt (major and minor)), 113.7 (ArC^7H (major)), 113.9 (ArC^7H (minor)), 119.1 (ArC^{3a} (major)), 119.2 (ArC^{3a} (minor)), 123.4 (ArC^5H (major)), 123.5 (ArC^5H (minor)), 125.3 (ArC^4H (minor)), 125.4 (ArC^4H (major)), 125.6 (q, *J* 282.2, CF_3 (major)), 126.0 (q, *J* 282.0, CF_3 (minor)), 127.8 (PhC^4H (major and minor)), 128.0 ($PhC^{2,6}H$ (major)), 128.1 ($PhC^{2,6}H$ (minor)), 128.8_5 ($PhC^{3,5}H$ (minor)), 128.8_8 ($PhC^{3,5}H$ (major)), 137.8 (PhC^1 (minor)), 137.9 (PhC^1 (major)), 139.0 (ArC^6H (major)), 139.4 (ArC^6H (minor)), 163.9 (*C*OOEt (minor)), 164.2 (*C*OOEt (major)), 167.6 (*C*ONHBn (minor)),

168.2 (*C*ONHBn (major)), 171.7 (Ar*C*[7a] (major)), 172.8 (Ar*C*[7a] (minor)), 192.4 (ArC[4a]*C*O (major)), 193.0 (ArC[4a]*C*O (minor)); **^{19}F NMR** (471 MHz, $CDCl_3$) δ_F: −65.37 (0.45F, d, *J* 8.6, C*F*$_3$ (minor)), −65.34 (2.55F, d, *J* 8.5, C*F*$_3$ (major)); **HRMS** (ESI$^+$) $C_{22}H_{20}F_3NO_5Na$ $[M+Na]^+$ found 458.1176, requires 458.1186 (−2.14 ppm).

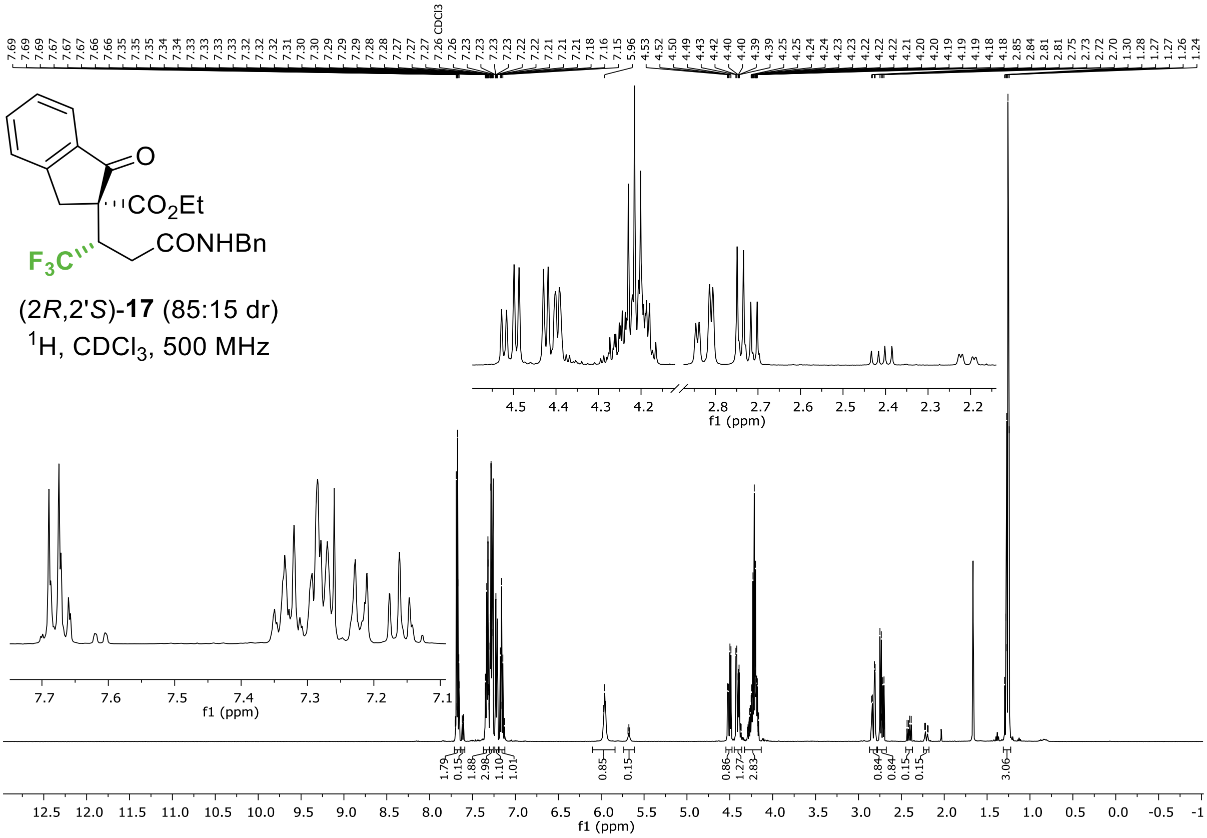

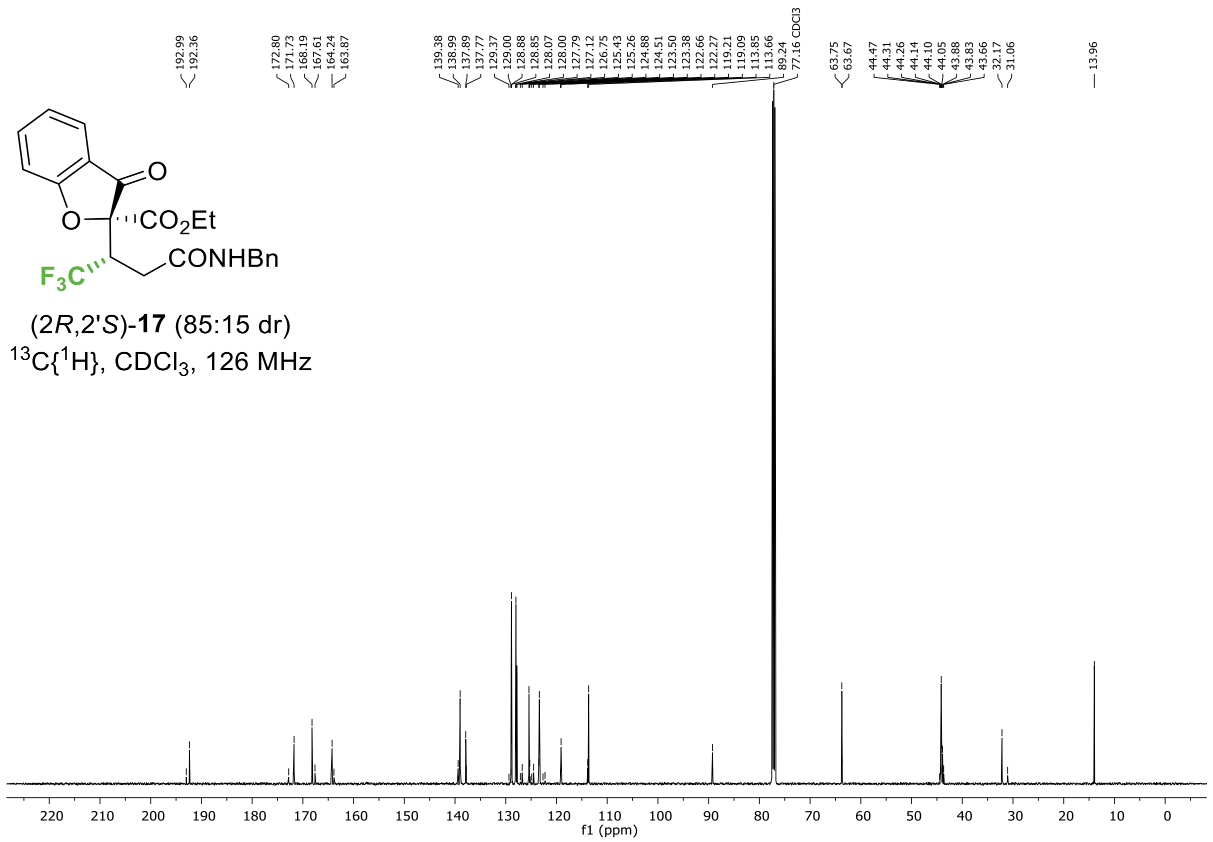

192.99
192.36
172.80
171.73
168.19
167.61
164.24
163.87
139.38
138.99
137.89
137.77
129.37
129.00
128.88
128.85
128.07
128.00
127.79
127.12
126.75
125.43
125.26
124.88
124.51
123.50
123.38
122.66
122.27
119.21
119.09
113.85
113.66
89.24
77.16 CDCl3
63.75
63.67
44.47
44.31
44.26
44.14
44.10
44.05
43.88
43.83
43.66
32.17
31.06
13.96
O
O
CO2Et
CONHBn
F3C
(2R,2'S)-17 (85:15 dr)
13C{1H}, CDCl3, 126 MHz
220 210 200 190 180 170 160 150 140 130 120 110 100 90 80 70 60 50 40 30 20 10 0
f1 (ppm)

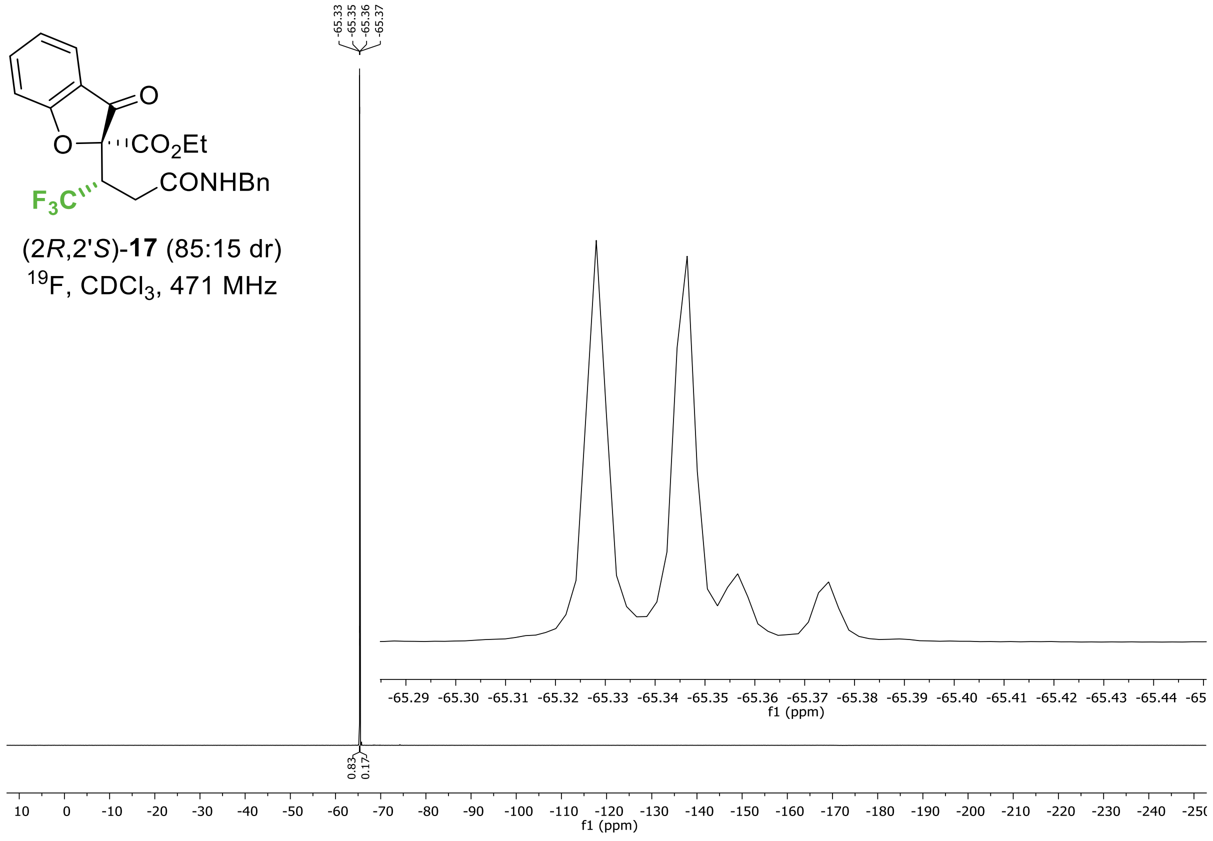

-65.33
-65.35
-65.36
-65.37
O
O
CO2Et
CONHBn
F3C
(2R,2'S)-17 (85:15 dr)
19F, CDCl3, 471 MHz
-65.29 -65.30 -65.31 -65.32 -65.33 -65.34 -65.35 -65.36 -65.37 -65.38 -65.39 -65.40 -65.41 -65.42 -65.43 -65.44 -65
f1 (ppm)
0.83
0.17
10 0 -10 -20 -30 -40 -50 -60 -70 -80 -90 -100 -110 -120 -130 -140 -150 -160 -170 -180 -190 -200 -210 -220 -230 -240 -250
f1 (ppm)

HPLC Data for 17: Chiralpak OD-H (95:5 hexane:^{i}PrOH, flow rate 1.00 mL·min^{-1}, 254 nm, 30 °C) t_R (2*R*,2'*S*)-**17**: 36.6 min, t_R (2*S*,2'*R*)-**17**: 41.7 min, 89:11 er; t_R *rel*-(2*S*,2'*S*)-**17**: 47.2 min, t_R *rel*-(2*R*,2'*R*)-**17**: 55.7 min, 94:6 er.

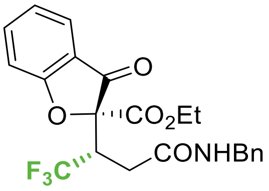

(2*R*,2'*S*)-**17**

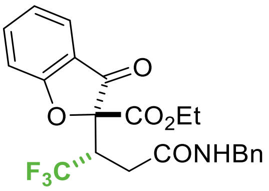

rel-(2*S*,2'*S*)-**17**

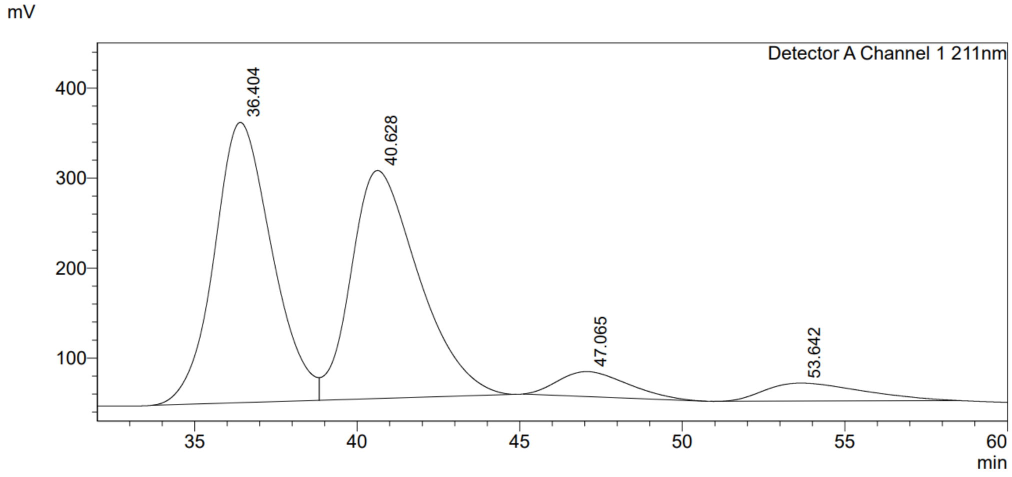

<Peak Table>

Detector A Channel 2 254nm

Peak#	Ret. Time	Area%
1	36.407	43.447
2	40.625	43.327
3	47.065	7.379
4	53.647	5.848
Total		100.000

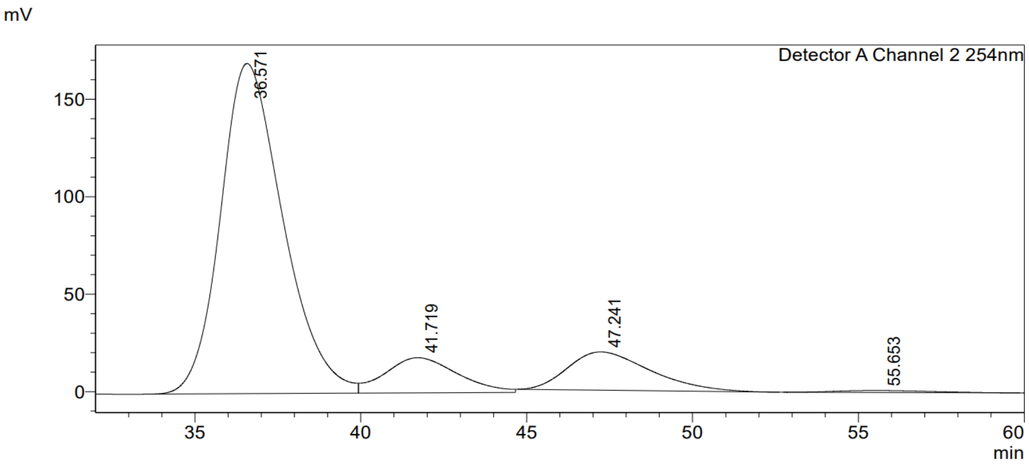

<Peak Table>

Detector A Channel 2 254nm

Peak#	Ret. Time	Area%
1	36.571	77.748
2	41.719	9.562
3	47.241	11.941
4	55.653	0.749
Total		100.000

(+)-Methyl (*S*)-1-acetyl-2-((*S*)-4'-(benzylamino)-1',1',1'-trifluoro-4'-oxobutan-2'-yl)-3-oxoindoline-2-carboxylate (2*S*,2'*S*)-19

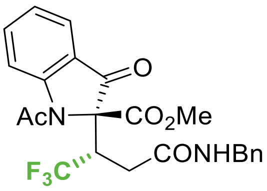

(2*S*,2'*S*)-**19**

Preparation from (E)-enoate:

The title compound was prepared according to **General Procedure 2**. A mixture of 4-nitrophenyl (*E*)-4,4,4-trifluorobut-2-enoate **8** (26.1 mg, 0.1 mmol), (2*S*,3*R*)-HyperBTM **1** (1.5 mg, 5 µmol) and methyl 1-acetyl-3-oxoindoline-2-carboxylate **18** (23.3 mg, 0.1 mmol) in DMA (0.4 mL) was stirred for 16 h at room temperature. Benzylamine (11 µL, 0.1 mmol) was added, and the reaction was permitted to stir for 1 h. The solution was diluted with EtOAc, washed sequentially with saturated aqueous Na_2CO_3 (×3) and brine, dried over $MgSO_4$ and filtered. The solvent was removed under reduced pressure and the residue was purified by column chromatography (1:1 EtOAc:Hexane) to give the title compound (30.9 mg, 67%, >95:5 d.r.) as white solid.

Gram-scale preparation from (*E*)-enoate:

The title compound was prepared according to **General Procedure 2**. A mixture of 4-nitrophenyl (*E*)-4,4,4-trifluorobut-2-enoate **8** (1.15 g, 4.4 mmol), (2*S*,3*R*)-HyperBTM **1** (67.8 mg, 0.22 mmol) and methyl 1-acetyl-3-oxoindoline-2-carboxylate **18** (1.03 g, 4.4 mmol) in DMA (17.6 mL) was stirred for 16 h at room temperature. Benzylamine (0.48 mL, 4.4 mmol) was then added, and the reaction was permitted to stir for 1 h. The solution was diluted with EtOAc, washed sequentially with saturated aqueous Na_2CO_3 (3×), brine, and dried over $MgSO_4$. The solvent was removed under reduced pressure and crystallization from mixed solvent (1:2 EtOAc:Hexane) to give the title compound (1.73 g, 85%, >95:5 dr, 99:1 er) as yellow solid.

Preparation from (*Z*)-enoate:

A mixture of 4-nitrophenyl (*Z*)-4,4,4-trifluorobut-2-enoate **8** (26.1 mg, 0.1 mmol), (2*S*,3*R*)-HyperBTM **1** (1.5 mg, 5 µmol) and methyl 1-acetyl-3-oxoindoline-2-carboxylate **18** (23.3 mg, 0.1 mmol) in DMA (0.4 mL) was stirred for 16 h at room temperature. Benzylamine (11 µL, 0.1 mmol) was added, and the reaction was permitted to stir for 1 h. The solution was diluted with EtOAc, washed sequentially with saturated aqueous Na_2CO_3 (×3) and brine, dried over $MgSO_4$ and filtered. The solvent was removed under reduced pressure and the residue was purified by column chromatography (1:1 EtOAc:Hexane) to give the title compound (21.4 mg, 46%, >95:5 d.r.) as white solid.

Data for (2*S*,2'*S*)-**19**: **mp** 140-145 °C; $[\alpha]_D^{20}$ +57.36 (*c* 0.53 in $CHCl_3$); **IR** ν_{max} (film) / cm^{-1} 3323, 2953, 2021, 1759, 1715, 1670, 1373, 1279, 1121, 1051, 966, 764; **^{1}H NMR** (500 MHz, $CDCl_3$) δ_H: 2.37 (1H, dd, *J* 16.6, 8.9, CHC$H_A$$H_B$CONH), 2.56 (3H, s, NCOC$H_3$), 3.66 (3H, s, COOC$H_3$), 3.72 (1H, d, *J* 16.5, CHC$H_A$$H_B$CONH), 4.46 (1H, dd, *J* 14.8, 5.4, NHC$H_A$$H_B$Ph), 4.57 (1H, dd, *J* 14.8, 6.0, NHC$H_A$$H_B$Ph), 4.66 (1H, qdd, *J* 9.9, 8.9, 1.6, C*H*CH_2CONH), 6.19 (1H, s (br), N*H*), 7.22-7.29 (2H, m, PhC^4*H*, ArC^5*H*), 7.29-7.37 (4H, m, Ph$C^{2,3,5,6}$*H*), 7.54 (1H, s (br), ArC^7*H*), 7.73 (1H, ddd, *J* 8.6, 7.3, 1.4, ArC^6*H*), 7.78 (1H, d, *J* 7.6, ArC^4*H*); **^{13}C{^{1}H} NMR** (126 MHz, $CDCl_3$) δ_C: 25.9 (CO*C*H_3), 31.4 (CH*C*H_2CONH), 42.6 (q, *J* 25.5, *C*HCH_2CONH), 43.9 (NH*C*H_2Ph), 71.4 (*C*COOMe), 115.0 (PhC^7H), 124.1 (ArC^{3a}), 124.4 (ArC^5H), 125.8 (ArC^4H), 126.5 (q, *J* 280.8, *C*F_3), 127.5 (PhC^4H), 127.9 (Ph$C^{2,6}$H), 128.8 (Ph$C^{3,5}$H), 137.9 (ArC^6H), 138.5 (PhC^1), 151.6 (ArC^{7a}), 166.1 (C*C*OOMe), 168.7 (N*C*OMe), 169.7 (*C*ONHBn), 192.0 (*C*O$CH_2$$CH_2$); **^{19}F NMR** (471 MHz, $CDCl_3$) δ_F: −66.23 (s, CF_3). **HRMS** (ESI^+) $C_{23}H_{22}F_3O_5N_2$ $[M+H]^+$ found 463.1470, requires 463.1475 (–2.2 ppm).

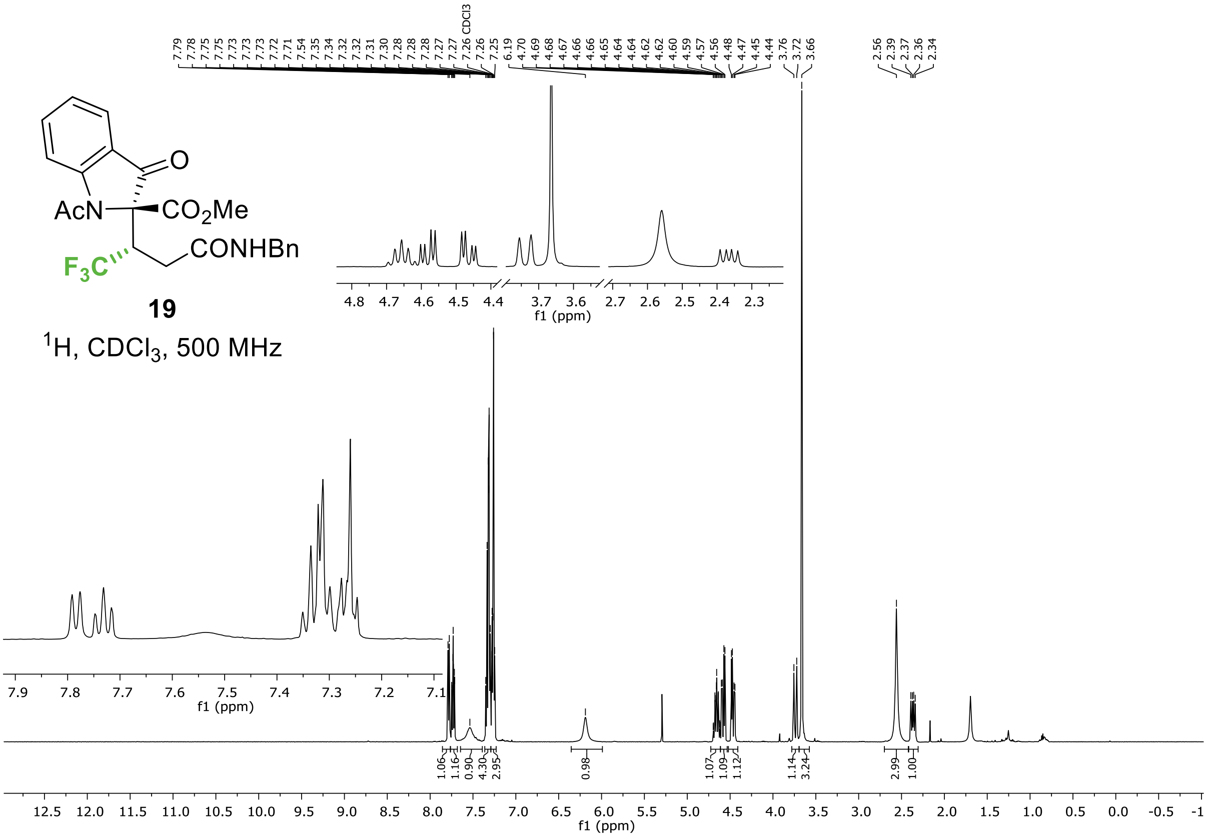

19

^{1}H, $CDCl_3$, 500 MHz

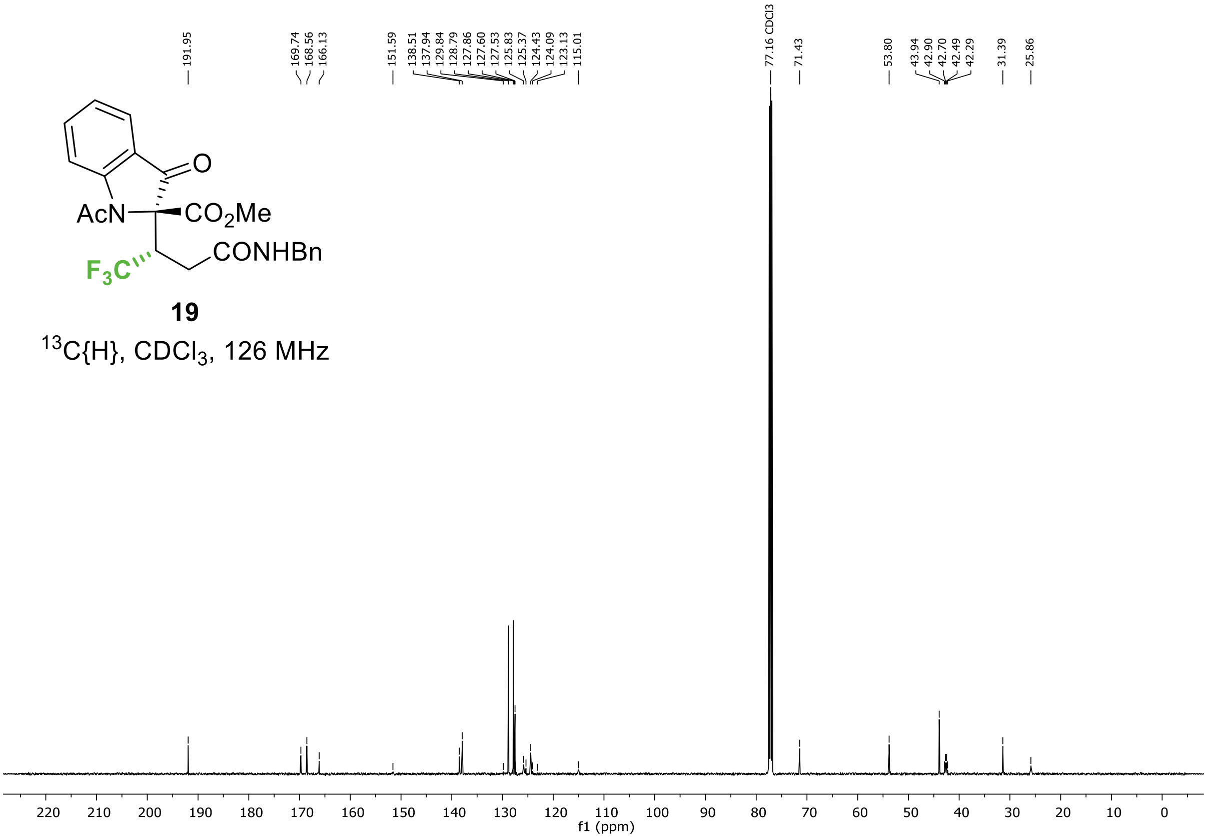

19

^{13}C{H}, $CDCl_3$, 126 MHz

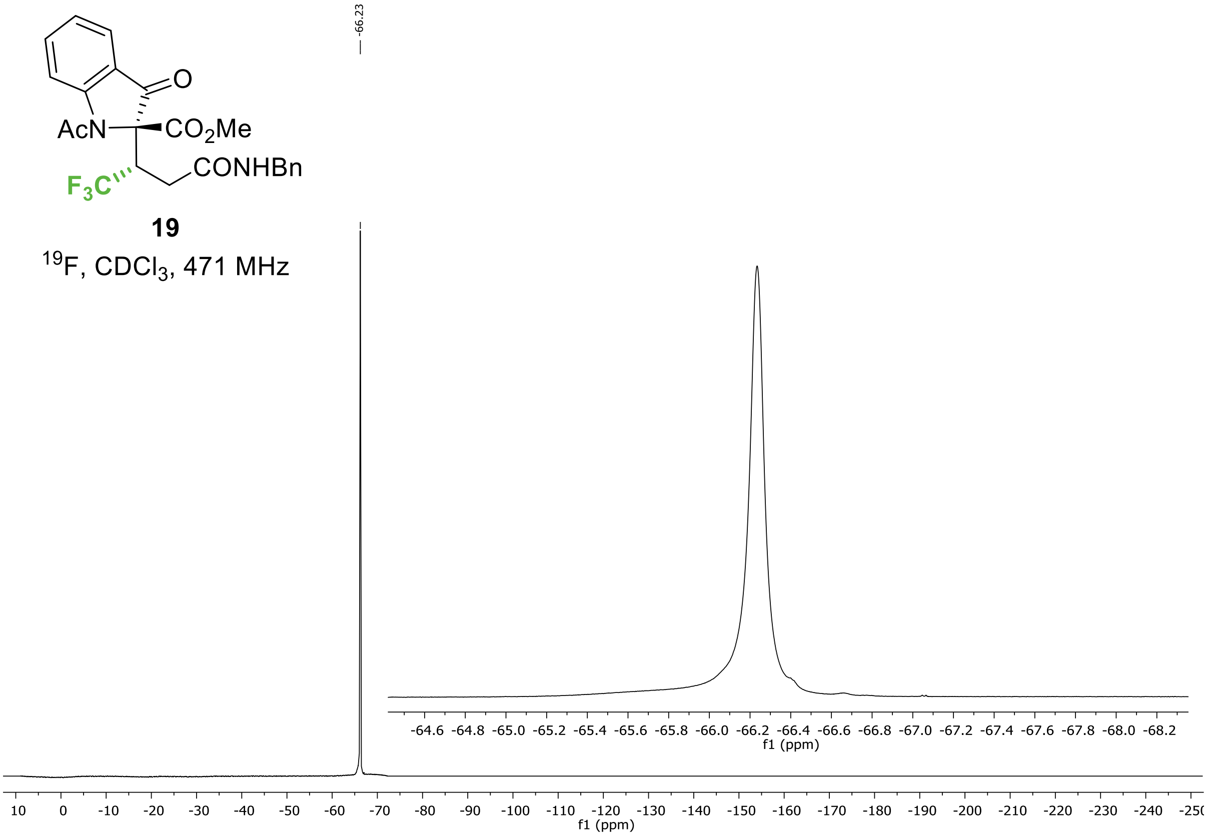

HPLC Data for 19 from (*E*)-ester: Chiralpak AD-H (90:10 hexane:^{i}PrOH, flow rate 1.00 mL·min^{-1}, 270 nm, 30 °C) t_R (2*S*,2'*S*)-**19**: 49.4 min, t_R (2*R*,2'*R*)-**19**: 75.7 min, 98:2 er.

HPLC Data for 19 from (*E*)-ester gram-scale: Chiralpak AD-H (90:10 hexane:^{i}PrOH, flow rate 1.00 mL·min^{-1}, 270 nm, 30 °C) t_R (2*S*,2'*S*)-**19**: 49.4 min, t_R (2*R*,2'*R*)-**19**: 75.7 min, 98:2 er.

HPLC Data for 19 from (*Z*)-ester: Chiralpak AD-H (90:10 hexane:^{i}PrOH, flow rate 1.00 mL·min^{-1}, 270 nm, 30 °C) t_R (2*S*,2'*S*)-**19**: 50.7 min, t_R (2*R*,2'*R*)-**19**: 73.5 min, 54:46 er.

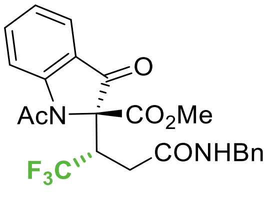

(2*S*,2'*S*)-**19**

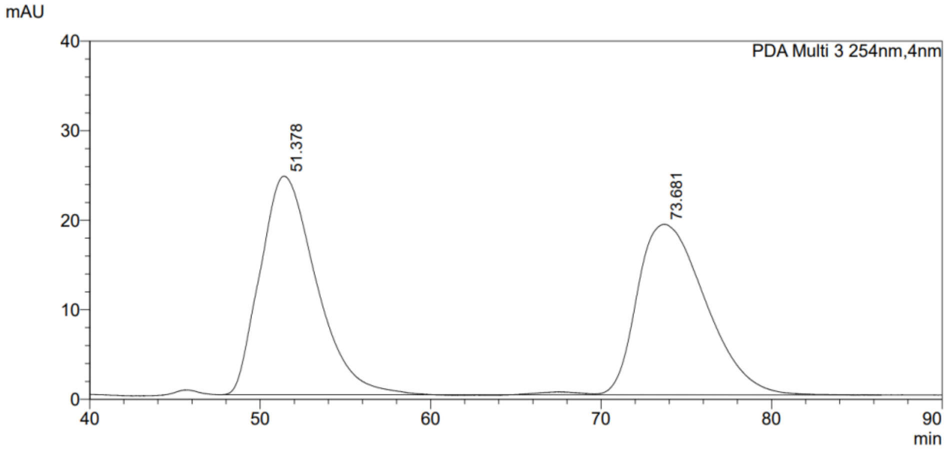

<Peak Table>

PDA Ch3 254nm

Peak#	Ret. Time	Area%
1	51.378	51.052
2	73.681	48.948
Total		100.000

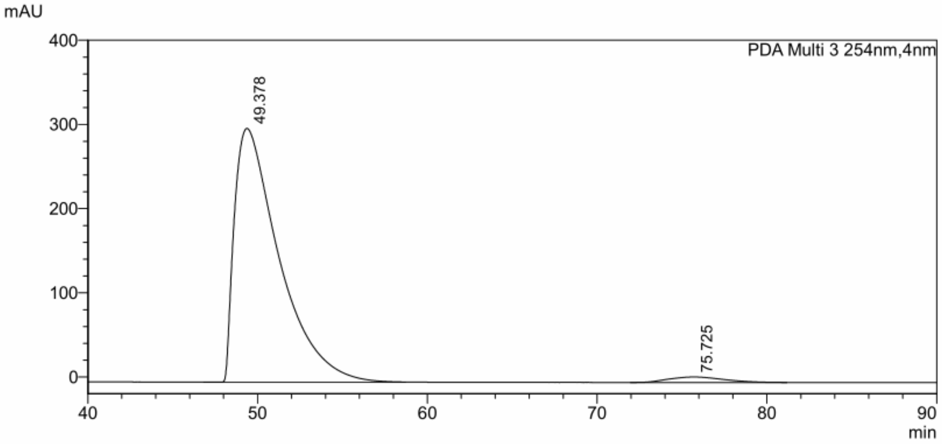

<Peak Table>

PDA Ch3 254nm

Peak#	Ret. Time	Area%
1	49.378	97.412
2	75.725	2.588
Total		100.000

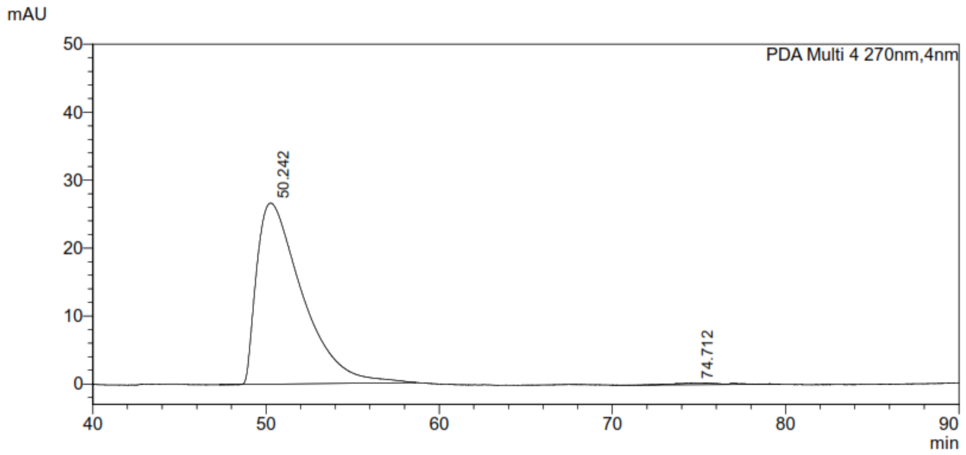

<Peak Table>

PDA Ch4 270nm

Peak#	Ret. Time	Area%
1	50.242	98.830
2	74.712	1.170
Total		100.000

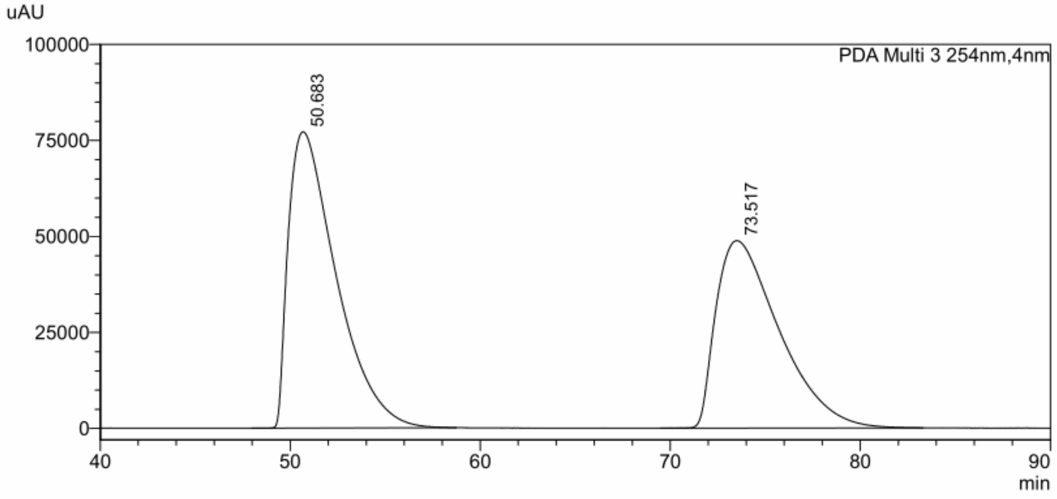

<Peak Table>

PDA Ch3 254nm

Peak#	Ret. Time	Area%
1	50.683	54.447
2	73.517	45.553
Total		100.000

(−)-Methyl (*S*)-1-acetyl-2-((*S*)-4-(benzylamino)-1-chloro-1,1-difluoro-4-oxobutan-2-yl)-3-oxoindoline-2-carboxylate (2*S*,2'*S*)-20

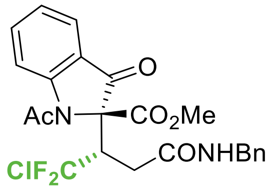

(2*S*,2'*S*)-**20**

The title compound was prepared according to **General Procedure 2**. A mixture of 4-nitrophenyl (*E*)-4-chloro-4,4-difluorobut-2-enoate **S1** (27.7 mg, 0.1 mmol), (2*S*,3*R*)-HyperBTM **1** (1.5 mg, 5 µmol) and methyl 1-acetyl-3-oxoindoline-2-carboxylate **18** (23.3 mg, 0.1 mmol) in DMA (0.4 mL) was stirred for 16 h at room temperature. Benzylamine (11 µL, 0.1 mmol) was added, and the reaction was permitted to stir for 1 h. The solution was diluted with EtOAc, washed sequentially with saturated aqueous Na_2CO_3 (×3) and brine, dried over $MgSO_4$ and filtered. The solvent was removed under reduced pressure and the residue was purified by column chromatography (1:1 EtOAc:Hexane) to give the title compound (27.0 mg, 56%, >95:5 d.r.) as white solid.

Data for (2*S*,2'*S*)-**20**: **mp** 138-142 ºC; $[\alpha]_D^{20}$ –42.83 (*c* 0.60 in $CHCl_3$); **IR** ν_{max} (film) / cm^{-1} 3312, 2953, 1755, 1715, 1670, 1655, 1470, 1375, 1277, 1238, 945, 901; **^{1}H NMR** (500 MHz, $CDCl_3$) δ_H: 2.42 (1H, dd, *J* 16.8, 8.3, CHC$H_A$$H_B$CONH), 2.56 (3H, s, NCOC$H_3$), 3.65 (3H, s, COOC$H_3$), 3.85 (1H, dq, *J* 16.8, 2.0, CHC$H_A$$H_B$CONH), 4.48 (1H, dd, *J* 14.8, 5.4, NHC$H_A$$H_B$Ph), 4.59 (1H, dd, *J* 14.8, 6.0, NHC$H_A$$H_B$Ph), 4.79 (1H, dddd, *J* 12.3, 8.4, 6.9, 1.5, C*H*CH_2CONH), 6.30 (1H, s (br), N*H*), 7.23-7.29 (2H, m, PhC^4*H*, ArC^5*H*), 7.29-7.36 (4H, m, Ph$C^{2,3,5,6}$*H*), 7.53 (1H, s (br), ArC^7*H*), 7.73 (1H, ddd, *J* 8.6, 7.3, 1.5, ArC^6*H*), 7.78 (1H, d (br), *J* 7.7, ArC^4*H*); **^{13}C{^{1}H} NMR** (126 MHz, $CDCl_3$) δ_C: 26.0 (NCOCH_3), 32.9 (CHCH_2CONH), 43.8 (NHCH_2Ph), 48.6 (dd, *J* 22.9, 21.3, *C*HCH_2CONH), 53.8 (COOCH_3), 72.1 (d, *J* 3.7, *C*COOMe), 115.0 (ArC^7H), 124.4 (ArC^5H), 124.8 (ArC^{3a}), 125.7 (ArC^4H), 127.5 (PhC^4H), 127.9 (Ph$C^{2,6}$H), 128.8 (Ph$C^{3,5}$H), 130.0 (app t, *J* 297.0, CF_2Cl), 137.8 (ArC^6H), 138.4 (PhC^1), 151.6 (ArC^{7a}), 166.3 (*C*OOMe), 168.8 (N*C*OMe), 169.9 (*C*ONHBn), 192.1 (Ar*C*OC); **^{19}F NMR** (471 MHz, $CDCl_3$) δ_F: −50.48 (1F, dd, *J* 170.8, 12.4, C$F_A$$F_B$Cl), −48.83 (1F, dd, *J* 170.5, 6.6, C$F_A$$F_B$Cl); **HRMS** ($ESI^+$) $C_{23}H_{22}F_2O_5N_2Cl$ $[M+H]^+$ found 479.1180, requires 479.1180 (+0.04 ppm).

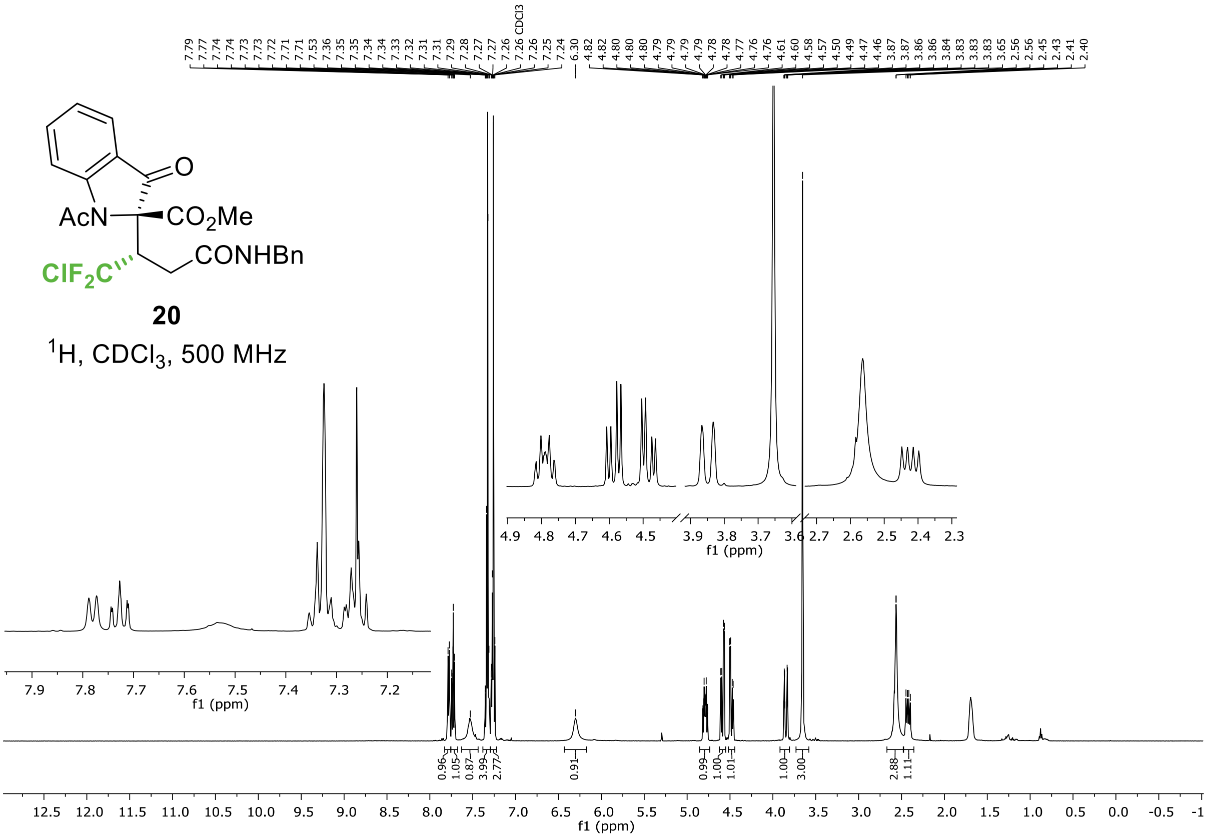

20

^{1}H, $CDCl_3$, 500 MHz

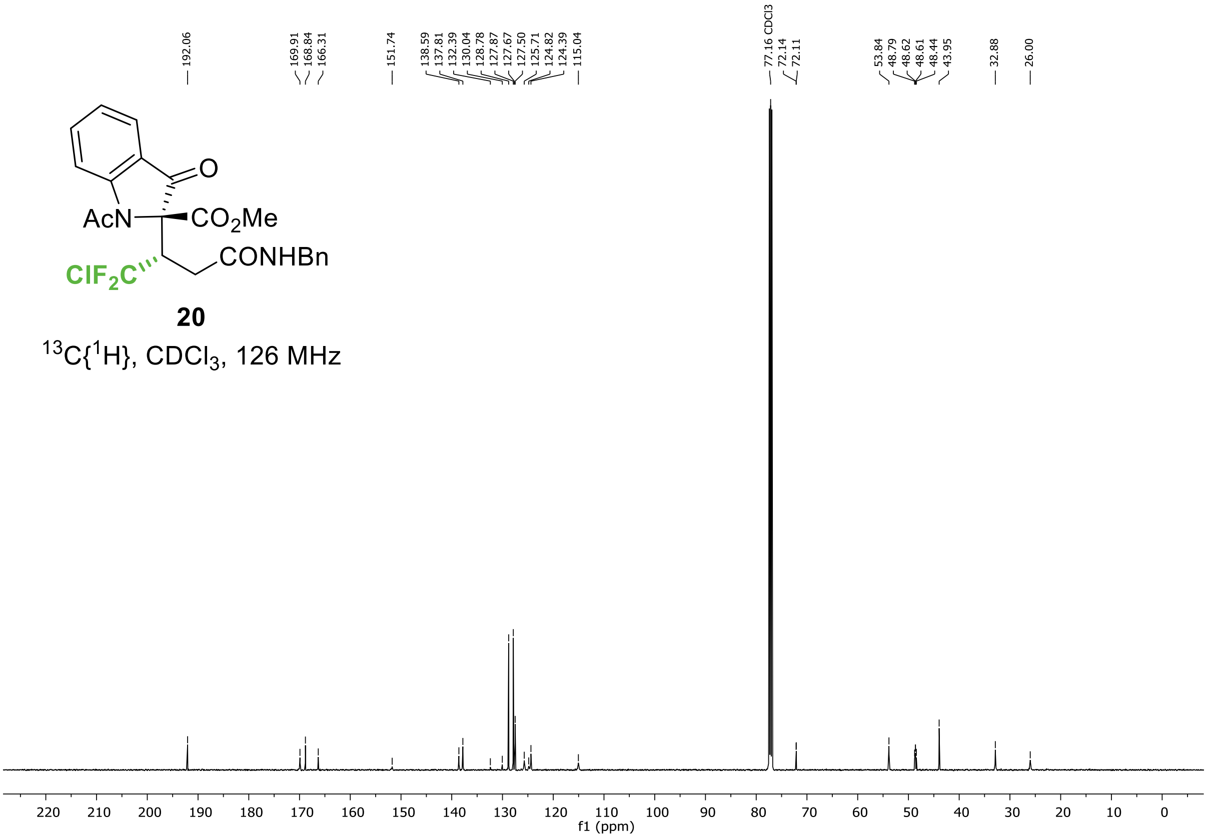

20

$^{13}C\{^{1}H\}$, $CDCl_3$, 126 MHz

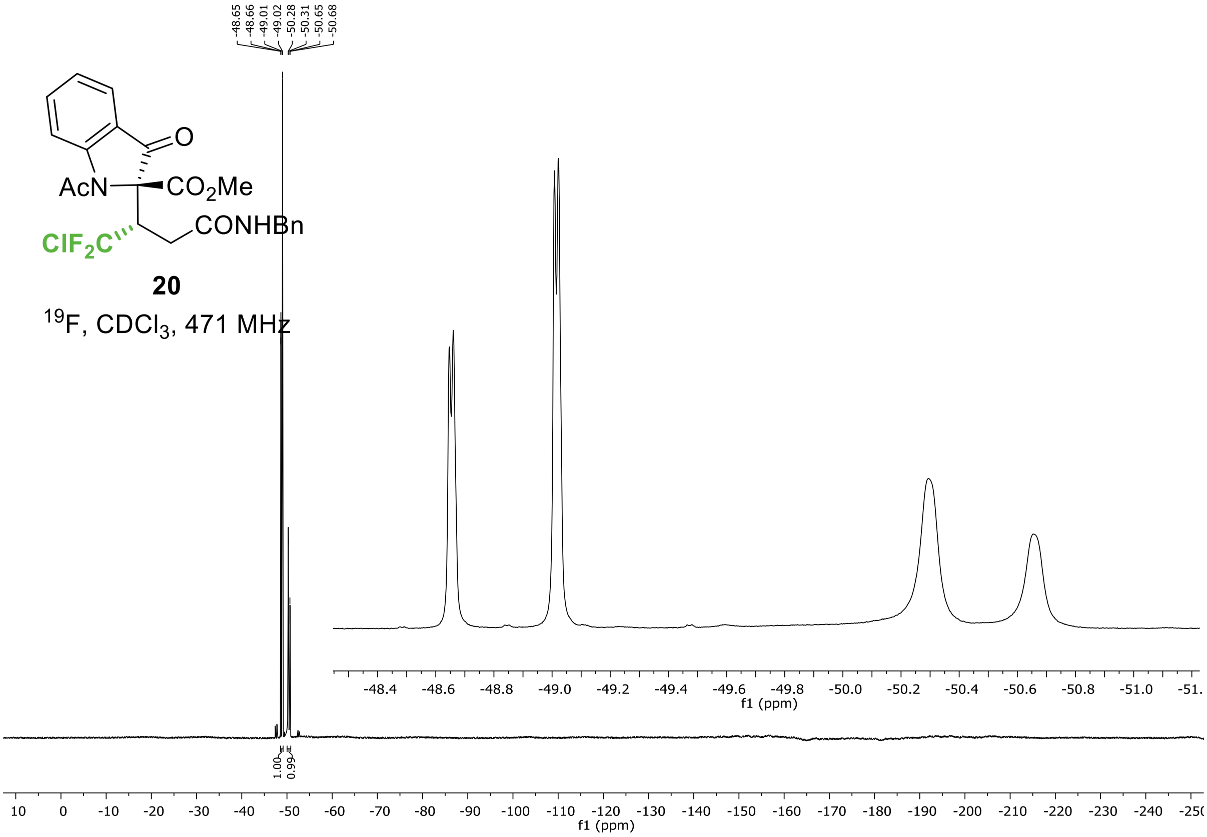

HPLC Data for 20: Chiralpak OD-H (90:10 hexane:^{i}PrOH, flow rate 1.00 mL·min^{-1}, 270 nm, 30 °C) t_R (2*R*,2'*R*)-**20**: 41.3 min, t_R (2*S*,2'*S*)-**20**: 57.2 min, 98:2 er.

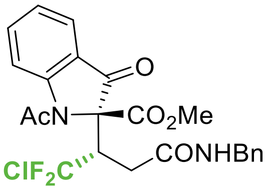

(2*S*,2'*S*)-**20**

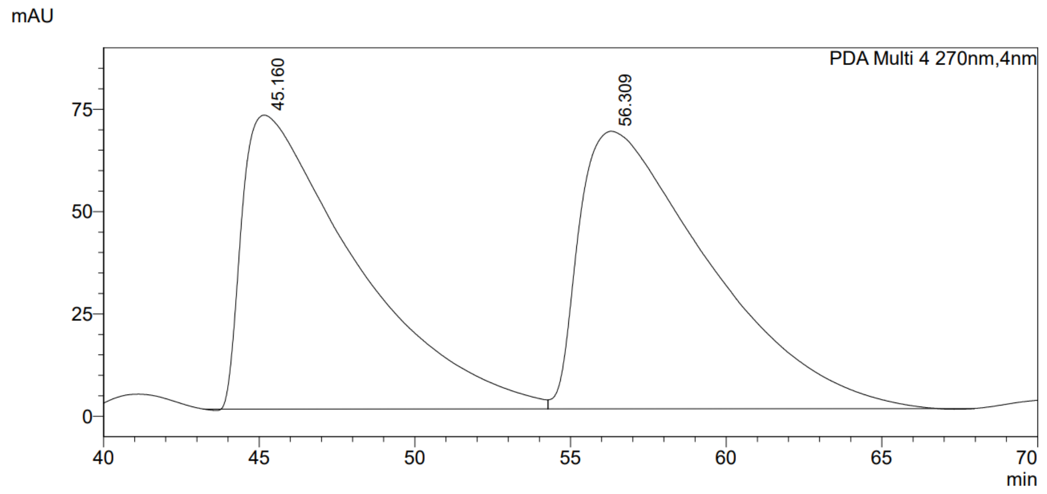

<Peak Table>

PDA Ch4 270nm

Peak#	Ret. Time	Area%
1	45.160	48.113
2	56.309	51.887
Total		100.000

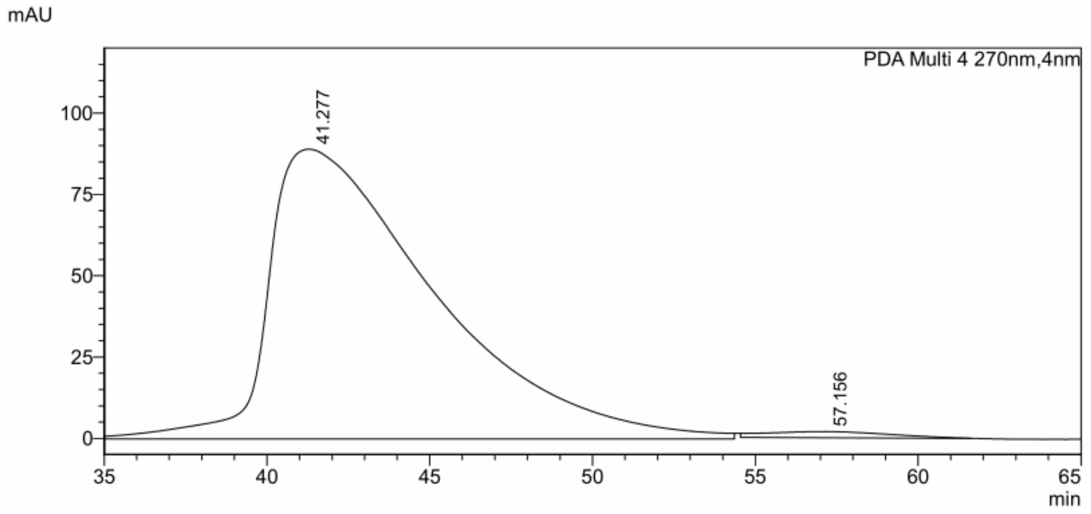

<Peak Table>

PDA Ch4 270nm

Peak#	Ret. Time	Area%
1	41.277	98.422
2	57.156	1.578
Total		100.000

(−)-Methyl (*S*)-1-acetyl-2-((*S*)-4'-(benzylamino)-1'-bromo-1',1'-difluoro-4'-oxobutan-2'-yl)-3-oxoindoline-2-carboxylate (2*S*,2'*S*)-21

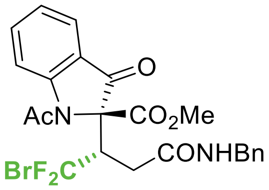

(2*S*,2'*S*)-**21**

The title compound was prepared according to **General Procedure 2**. A mixture of 4-nitrophenyl (*E*)-4-bromo-4,4-difluorobut-2-enoate **S2** (32.1 mg, 0.1 mmol), (2*S*,3*R*)-HyperBTM **1** (1.5 mg, 5 µmol) and methyl 1-acetyl-3-oxoindoline-2-carboxylate **18** (23.3 mg, 0.1 mmol) in DMA (0.4 mL) was stirred for 16 h at room temperature. Benzylamine (11 µL, 0.1 mmol) was added, and the reaction was permitted to stir for 1 h. The solution was diluted with EtOAc, washed sequentially with saturated aqueous Na_2CO_3 (×3) and brine, dried over $MgSO_4$ and filtered. The solvent was removed under reduced pressure and the residue was purified by column chromatography (1:1 EtOAc:Hexane) to give the title compound (26.2 mg, 50%, >95:5 d.r.) as white solid.

Data for (2*S*,2'*S*)-**21**: **mp** 140-145 °C; $[\alpha]_D^{20}$ –43.28 (*c* 1.16 in $CHCl_3$); **IR** ν_{max} (film) / cm^{-1} 3312, 2953, 1755, 1717, 1670, 1655, 1470, 1375, 1238, 893, 770; **^{1}H NMR** (500 MHz, $CDCl_3$) δ_H: 2.42 (1H, dd, *J* 16.9, 8.0, CHC$H_A$$H_B$CONH), 2.56 (3H, s (br), NCOCH_3), 3.65 (3H, s, COOCH_3), 3.87 (1H, dq, *J* 16.9, 1.7, CHC$H_A$$H_B$CONH), 4.49 (1H, dd, *J* 14.8, 5.5, NHC$H_A$$H_B$Ph), 4.58 (1H, dd, *J* 14.8, 6.0, NHC$H_A$$H_B$Ph), 4.78 (1H, dddd, *J* 13.6, 7.8, 7.7, 1.6, C*H*CH_2CONH), 6.31 (1H, s (br), N*H*), 7.23-7.30 (2H, m, PhC^4*H*, ArC^5*H*), 7.30-7.36 (4H, Ph$C^{2,3,5,6}$*H*), 7.53 (1H, s (br), ArC^7*H*), 7.73 (1H, ddd, *J* 8.6, 7.3, 1.5, ArC^6*H*), 7.78 (1H, d (br), *J* 7.7,ArC^4*H*); **^{13}C{^{1}H} NMR** (126 MHz, $CDCl_3$) δ_C: 26.0 (NCOCH_3), 33.5 (CHCH_2CONH), 44.0 (NHCH_2Ph), 50.0 (dd, *J* 21.3, 18.2, *C*HCH_2CONH), 53.8 (COOCH_3), 72.3 (*C*COOMe), 115.1 (ArC^7H), 123.3 (app t, *J* 309.8, *C*BrF_2), 124.4 (ArC^5H), 125.2 (ArC^{3a}), 125.7 (ArC^4H), 127.5 (PhC^4H), 127.9 (Ph$C^{2,6}$H), 128.8 (Ph$C^{3,5}$H), 137.8 (ArC^6H), 138.6 (PhC^1), 151.8 (ArC^{7a}), 166.2 (N*C*OMe), 168.9 (*C*OOMe), 169.8 (*C*ONH), 192.0 (Ar*C*OC); **^{19}F NMR** (471 MHz, $CDCl_3$) δ_F: −43.61 (1F, d, *J* 167.4, C$F_A$$F_B$Br), −41.8 (1F, d, *J* 167.4, C$F_A$$F_B$Br); **HRMS** (ESI$^+$) $C_{23}H_{22}F_2O_5N_2Br$ $[M+H]^+$ found 523.0675, requires 523.0663 (–2.2 ppm).

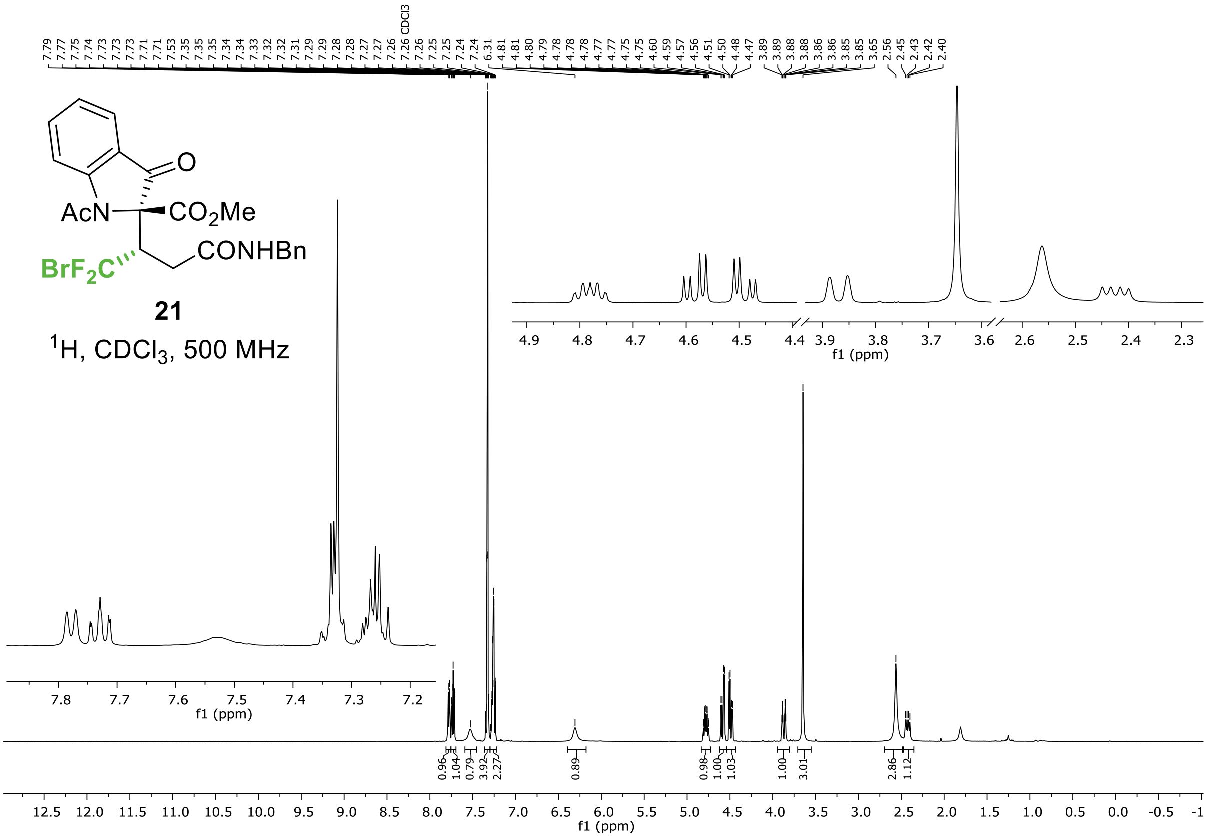
AcN
CO2Me
CONHBn
BrF2C
21
1H, CDCl3, 500 MHz
7.79 7.77 7.75 7.74 7.73 7.73 7.73 7.73 7.71 7.71 7.53 7.35 7.35 7.35 7.34 7.34 7.33 7.32 7.32 7.31 7.29 7.29 7.28 7.28 7.27 7.27 7.26 7.26 CDCl3 7.26 7.25 7.25 7.24 7.24 6.31 4.81 4.81 4.80 4.79 4.78 4.78 4.78 4.77 4.77 4.75 4.75 4.60 4.59 4.57 4.56 4.51 4.50 4.48 4.47 3.89 3.89 3.88 3.88 3.86 3.86 3.85 3.85 3.65 2.56 2.45 2.43 2.42 2.40
0.96 1.04 0.79 3.92 2.27 0.89 0.98 1.00 1.03 1.00 3.01 2.86 1.12
f1 (ppm)

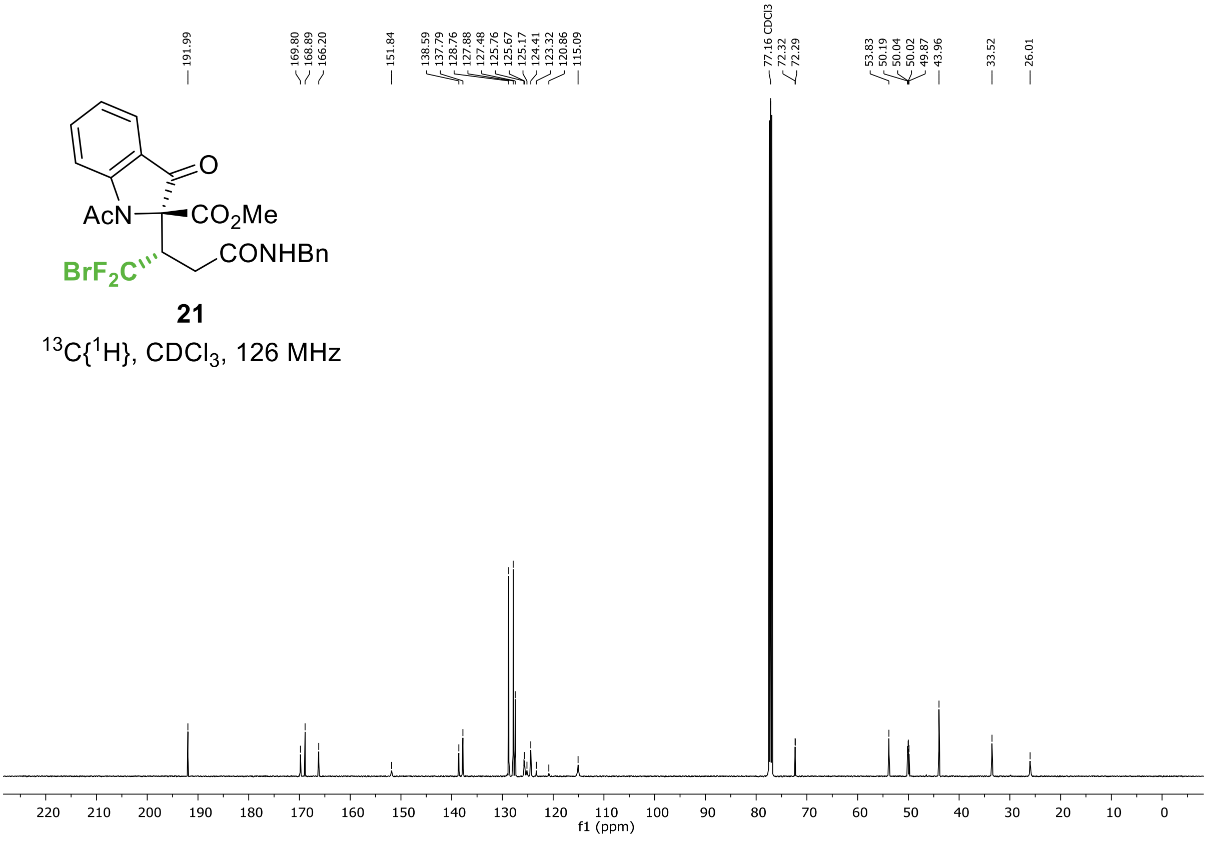
AcN
CO2Me
CONHBn
BrF2C
21
13C{1H}, CDCl3, 126 MHz
191.99 169.80 168.89 166.20 151.84 138.59 137.79 128.76 127.88 127.48 125.76 125.67 125.17 124.41 123.32 120.86 115.09 77.16 CDCl3 72.32 72.29 53.83 50.19 50.04 50.02 49.87 43.96 33.52 26.01
f1 (ppm)

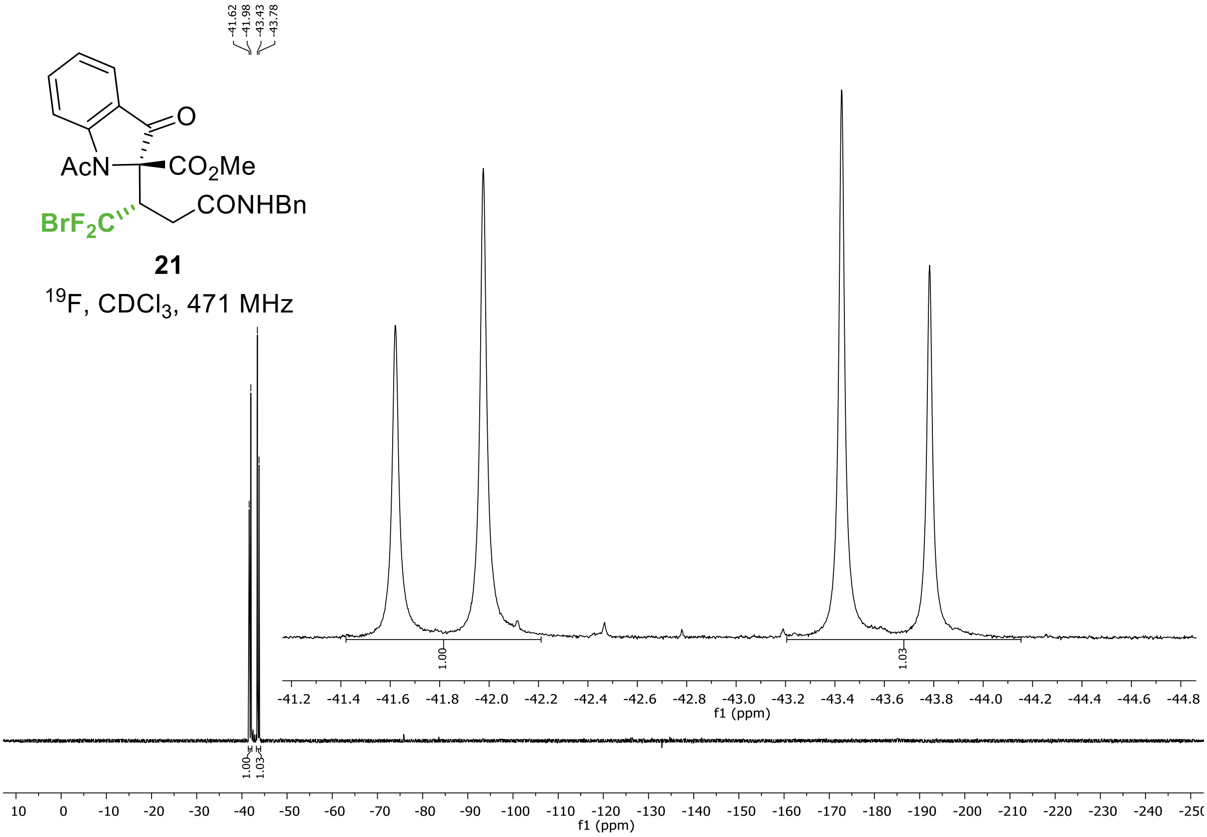

HPLC Data for 21: Chiralpak OD-H (90:10 hexane:^{i}PrOH, flow rate 1.00 mL·min^{-1}, 254 nm, 30 °C) t$_R$ (2*S*,2'*S*)-**21**: 46.9 min, t$_R$ (2*R*,2'*R*)-**21**: 63.7 min, 96:4 er.

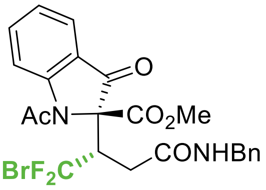

(2*S*,2'*S*)-**21**

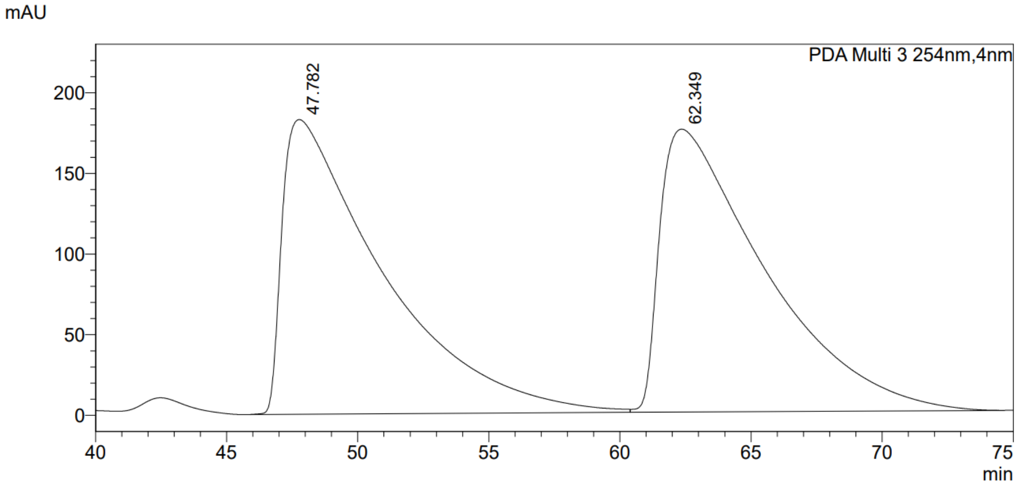

<Peak Table>

PDA Ch3 254nm

Peak#	Ret. Time	Area%
1	47.782	49.940
2	62.349	50.060
Total		100.000

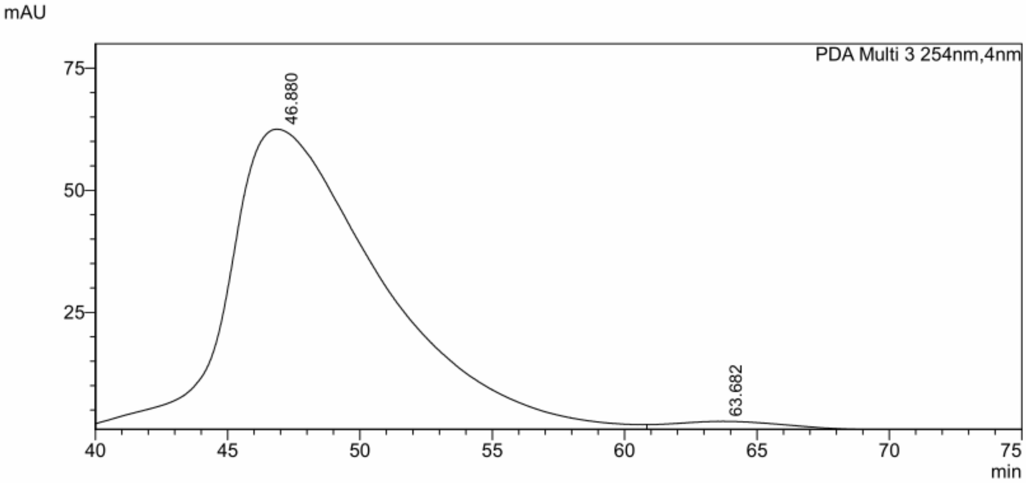

<Peak Table>

PDA Ch3 254nm

Peak#	Ret. Time	Area%
1	46.880	95.715
2	63.682	4.285
Total		100.000

(−)-Methyl (*S*)-1-acetyl-2-((*S*)-4-(benzylamino)-1,1,1,2,2-pentafluoro-5-oxopentan-3-yl)-3-oxoindoline-2-carboxylate (2*S*,2'*S*)-22

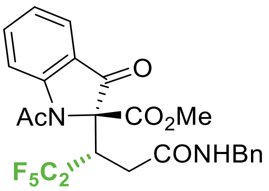

(2*S*,2'*S*)-**22**

The title compound was prepared according to **General Procedure 2**. A mixture of 4-nitrophenyl (*E*)-4,4,5,5,5-pentafluoropent-2-enoate **S3** (31.1 mg, 0.1 mmol), (2*S*,3*R*)-HyperBTM **1** (1.5 mg, 5 µmol) and methyl 1-acetyl-3-oxoindoline-2-carboxylate **18** (23.3 mg, 0.1 mmol) in DMA (0.4 mL) was stirred for 16 h at room temperature. Benzylamine (11 µL, 0.1 mmol) was added, and the reaction was permitted to stir for 1 h. The solution was diluted with EtOAc, washed sequentially with saturated aqueous Na_2CO_3 (×3) and brine, dried over $MgSO_4$ and filtered. The solvent was removed under reduced pressure and the residue was purified by column chromatography (1:1 EtOAc:Hexane) to give the title compound (19.5 mg, 38%, 86:14 d.r.) as a mixture of diastereomers as a colourless oil.

Data for **22**: $[\alpha]_D^{20}$ −23.10 (*c* 0.87 in $CHCl_3$); **IR** ν_{max} (film) / cm^{-1} 3333, 2957, 2851, 1761, 1672, 1472, 1373, 1206, 1179, 1115, 955, 912; **^{1}H NMR** (500 MHz, $CDCl_3$) δ_H: 2.52 (0.86H, dd, *J* 17.6, 7.6, CHH_AH_BCONH), 2.54 (2.58H, s, $NCOCH_3$), 2.61$_0$ (0.14H, dd, *J* 18.1, 6.3, CHH_AH_BCONH), 2.61$_3$ (0.42H, s, $NCOCH_3$), 3.51 (0.14H, d (br), *J* 18.3, $CHCH_AH_BCONH$), 3.61 (0.42H, s, $COOCH_3$), 3.64 (2.58H, 2, $COOCH_3$), 3.82 (0.86H, d (br), *J* 17.6, $CHCH_AH_BCONH$), 4.44-4.58 (0.28H, 2 x dd obscured by major signal, $NHCH_2Ph$), 4.50 (0.86H, dd, *J* 14.8, 5.5, $NHCH_AH_BPh$), 4.56 (0.86H, dd, *J* 14.8, 5.8, $NHCH_AH_BPh$), 4.80 (1.00H, *J* 20.5, 14.9, 7.6, 1.6, $CHCH_2CONH$, minor obscured), 5.80-5.85 (0.14H, m, N*H*), 6.12 (0.86H, s (br), N*H*), 7.22-7.30 (2.00H, m, PhC^4H, ArC^5H), 7.30-7.37 (4.00H, m, $PhC^{2,3,5,6}H$), 7.56 (0.14H, d, *J* 8.5, ArC^7H), 7.60 (0.86H, s (br), ArC^7H), 7.72$_5$ (0.14H, ddd, *J* 8.6, 7.3, 1.5, ArC^6H), 7.73$_3$ (0.86H, ddd, *J* 8.6, 7.2, 1.2, ArC^6H), 7.80 (0.86H, d, *J* 7.7, ArC^4H), 7.86 (0.14H, dd, *J* 7.7, 1.4, ArC^4H); **^{13}C{^{1}H} NMR** (126 MHz, $CDCl_3$) δ_C: 25.8 ($NCOCH_3$ (major)), 26.4 ($NCOCH_3$ (minor)), 30.5 ($CHCH_2CONH$ (major)), 34.0 ($CHCH_2CONH$ (minor)), 40.1 (app t, *J* 18.4, $CHCH_2CONH$ (major)), 42.0 (app t, *J* 17.5, $CHCH_2CONH$ (minor)), 44.0$_0$ ($NHCH_2Ph$ (major)), 44.0$_3$ ($NHCH_2Ph$ (minor)), 53.8 ($COOCH_3$ (major)), 53.9 ($COOCH_3$ (minor)), 72.2 (*C*COOMe (major and minor)), 115.4 (ArC^7H (major)), 116.4 (app tq, *J* 260.0, 37.3, CF_2CF_3 (major)), 118.7 (app qt, *J* 287.6, 36.5 CF_2CF_3 (major)), 122.8 (ArC^7H (minor)), 124.0 (ArC^5H (minor)), 124.5 (ArC^5H (major)), 125.8 (ArC^4H (major)), 126.4 (ArC^4H (minor)), 127.5 (PhC^4H, (major)), 127.6 (PhC^4H (minor)), 127.9 ($PhC^{2,6}H$ (major)), 128.0 ($PhC^{2,6}H$ (minor)), 128.8 ($PhC^{3,5}H$ (major and minor)), 137.5 (ArC^6H (minor)), 137.9 (ArC^6H (major)), 138.5$_5$ (PhC^1 (major)), 138.5$_8$ (PhC^1 (minor)), 151.9 (ArC^{7a}), 164.2 (*C*OOMe (minor)), 166.2 (*C*OOMe (major)), 168.8 (N*C*OMe (major)), 169.2 (*C*ONHBn (major)), 169.6 (*C*ONHBn (minor)), 170.1 (N*C*OMe (minor)), 190.4 (Ar*C*OC (minor)), 192.3 (Ar*C*OC (major)); **^{19}F NMR** (471 MHz, $CDCl_3$) δ_F: −123.96 (0.14F, d, *J* 273.0, $CF_AF_BCF_3$), −114.83 (0.86F, d, *J* 275.2, $CF_AF_BCF_3$), −113.93 (0.86F, d, *J* 275.2, $CF_AF_BCF_3$), −109.86 (0.14F, d, *J* 272.7, $CF_AF_BCF_3$), −82.84 (0.42F, s, CF_3), −81.13 (2.58F, s, CF_3 (major)); **HRMS** (ESI^+) $C_{24}H_{21}F_5O_5N_2Na$ $[M+Na]^+$ found 535.1263, requires 535.1253 (–1.84 ppm).

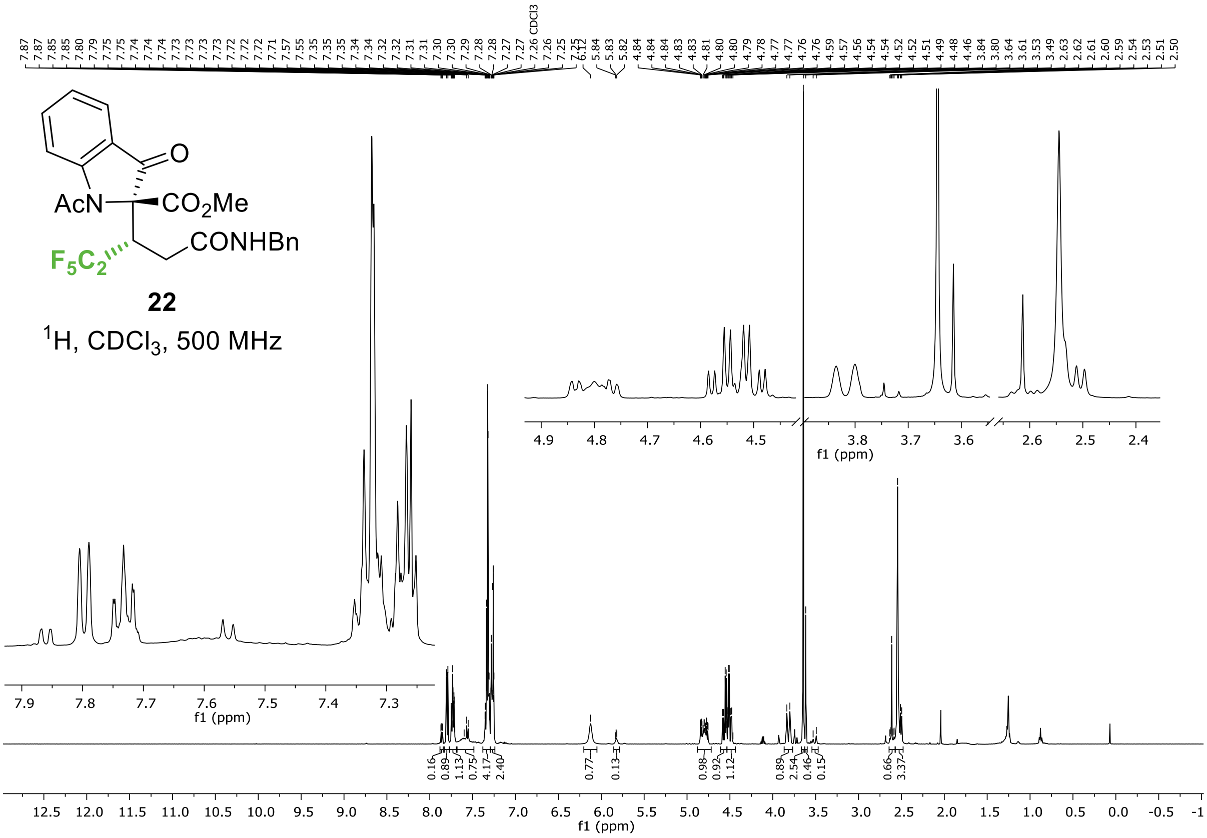
7.87 7.87 7.85 7.85 7.80 7.79 7.75 7.75 7.74 7.74 7.74 7.73 7.73 7.73 7.73 7.72 7.72 7.72 7.71 7.57 7.55 7.35 7.35 7.35 7.34 7.34 7.32 7.32 7.31 7.31 7.30 7.30 7.29 7.28 7.28 7.27 7.27 7.26 CDCl3 7.25 7.25 6.12 5.84 5.83 5.82 4.84 4.84 4.83 4.83 4.81 4.80 4.80 4.79 4.78 4.77 4.77 4.76 4.76 4.59 4.57 4.56 4.54 4.54 4.52 4.52 4.51 4.49 4.48 4.46 3.84 3.80 3.64 3.61 3.53 3.49 2.63 2.62 2.61 2.60 2.59 2.54 2.53 2.51 2.50
AcN
CO2Me
CONHBn
F5C2
O
22
1H, CDCl3, 500 MHz
0.16 0.89 1.13 0.75 4.17 2.40 0.77 0.13 0.98 0.92 1.12 0.89 2.54 0.46 0.15 0.66 3.37
f1 (ppm)
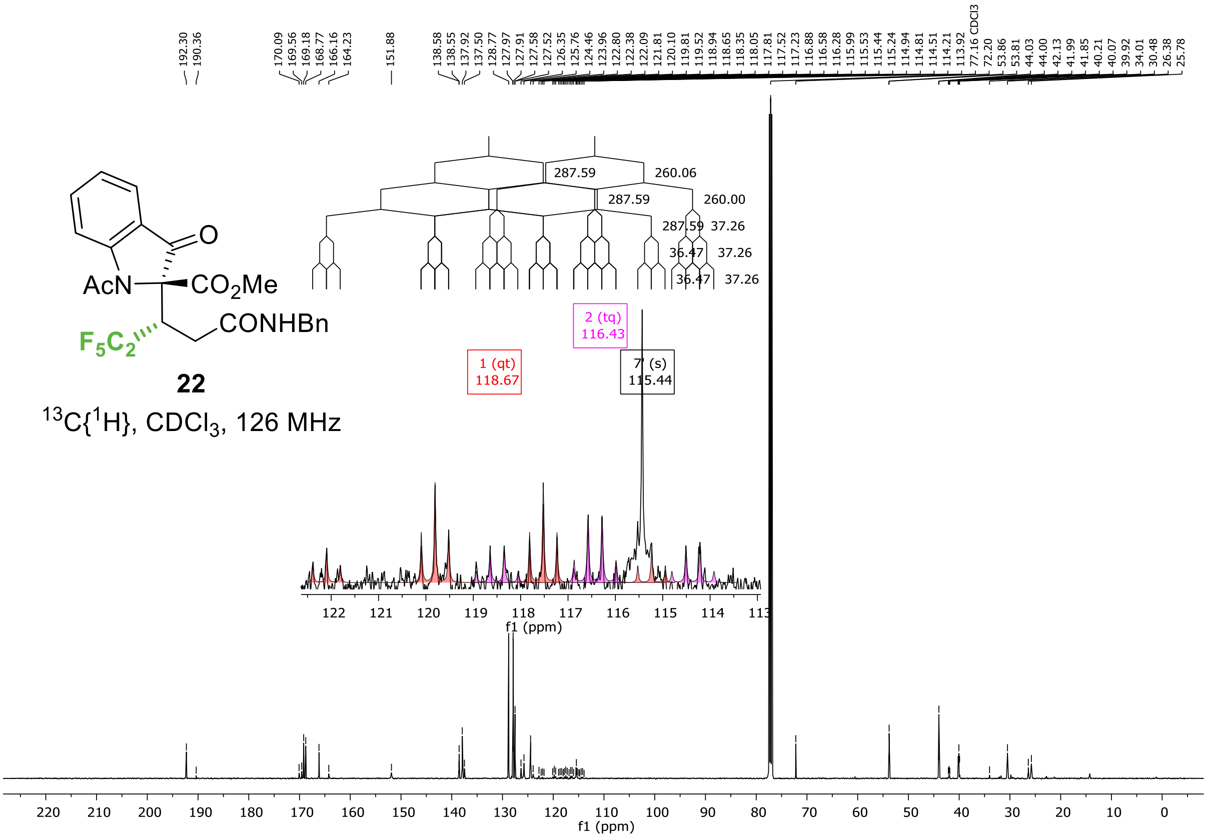
192.30 190.36 170.09 169.56 169.18 168.77 166.16 164.23 151.88 138.58 138.55 137.92 137.50 128.77 127.97 127.91 127.58 127.52 126.35 125.76 124.46 123.96 122.80 122.38 122.09 121.81 120.10 119.81 119.52 118.94 118.65 118.35 118.05 117.81 117.52 117.23 116.88 116.58 116.28 115.99 115.53 115.44 115.24 114.94 114.81 114.51 114.21 113.92 77.16 CDCl3 72.20 53.86 53.81 44.03 44.00 42.13 41.99 41.85 40.21 40.07 39.92 34.01 30.48 26.38 25.78
AcN
CO2Me
CONHBn
F5C2
O
22
13C{1H}, CDCl3, 126 MHz
287.59 260.06
287.59 260.00
287.59 37.26
36.47 37.26
36.47 37.26
2 (tq) 116.43
1 (qt) 118.67
7 (s) 115.44
f1 (ppm)

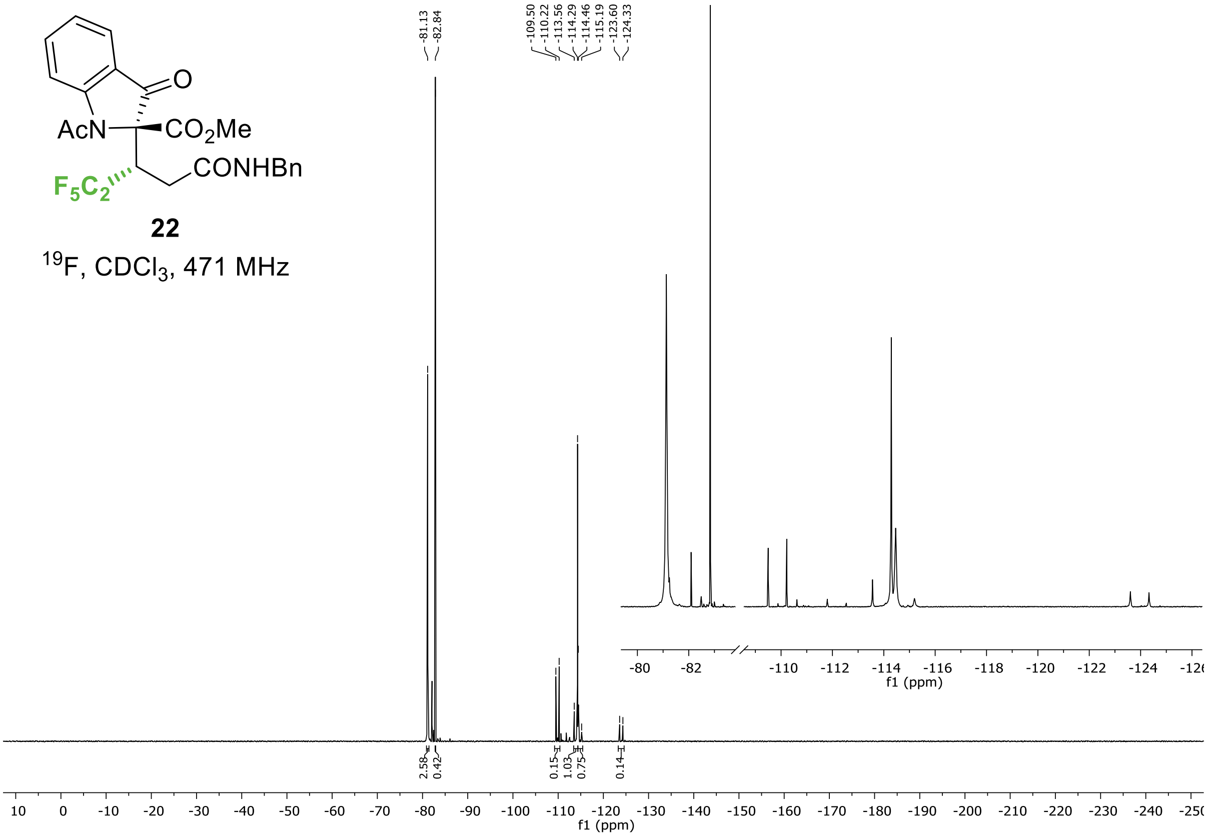

HPLC Data for 22: Chiralpak IB (95:5 hexane:^{i}PrOH, flow rate 1.00 mL·min^{-1}, 211 nm, 30 °C) t_R (2*S*,2’*S*)-**22**: 78.7 min, t_R (2*R*,2’*R*)-**22**: 112.3 min, 95:5 er; t_R *rel*-(2*R*,2’*S*)-**22**: 53.6 min, t_R *rel*-(2*S*,2’*R*)-**22**: 67.4 min, 76:24 er.

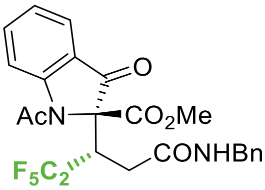

(2*S*,2’*S*)-**22**

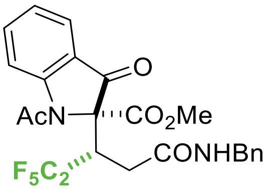

rel-(2*R*,2’*S*)-**22**

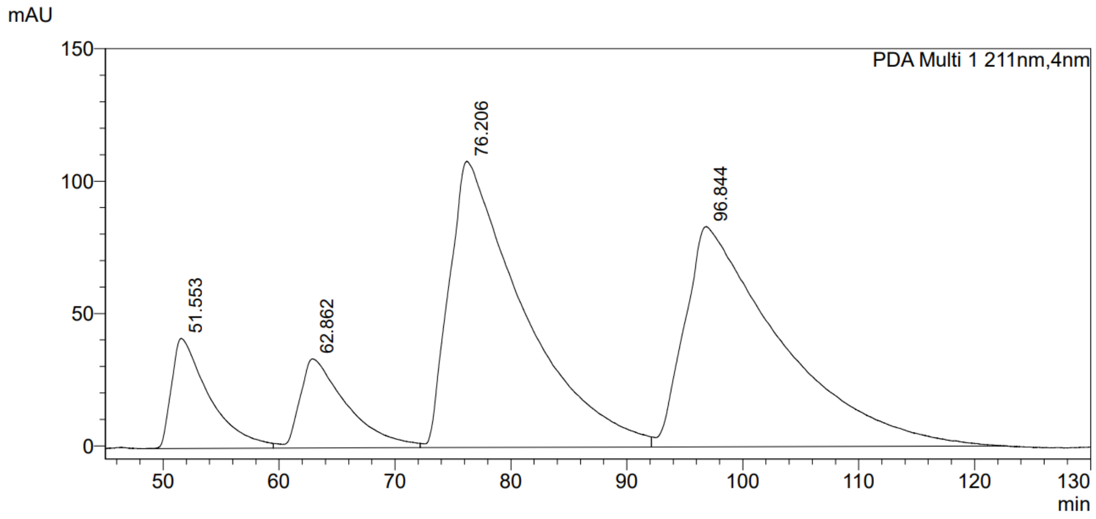

<Peak Table>

PDA Ch1 211nm

Peak#	Ret. Time	Area%
1	51.553	8.154
2	62.862	8.263
3	76.206	41.918
4	96.844	41.664
Total		100.000

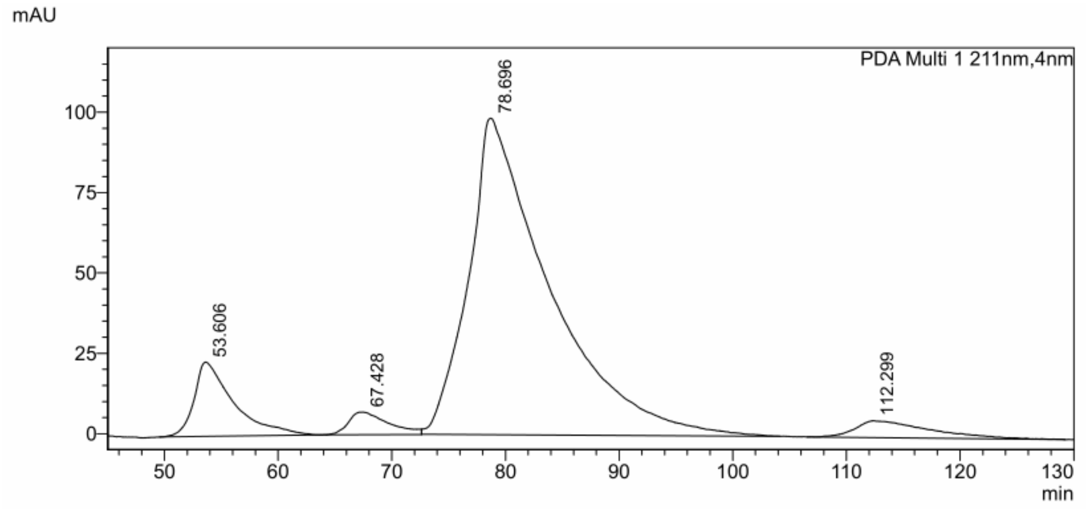

<Peak Table>

PDA Ch1 211nm

Peak#	Ret. Time	Area%
1	53.606	9.543
2	67.428	2.957
3	78.696	83.395
4	112.299	4.105
Total		100.000

(−)-Methyl (*S*)-1-acetyl-2-((*S*)-4-(benzylamino)-1-ethoxy-1,4-dioxobutan-2-yl)-3-oxoindoline-2-carboxylate (2*S*,2'*S*)-23

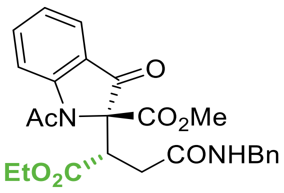

(2*S*,2'*S*)-**23**

Preparation from fumarate:

The title compound was prepared according to **General Procedure 2**. A mixture of ethyl (4-nitrophenyl) fumarate **26** (53.0 mg, 0.2 mmol), (2*S*,3*R*)-HyperBTM **1** (3.0 mg, 10 µmol) and methyl 1-acetyl-3-oxoindoline-2-carboxylate **18** (46.6 mg, 0.2 mmol) in DMA (0.8 mL) was stirred for 16 h at room temperature. Benzylamine (22 µL, 0.2 mmol) was then added, and the reaction was permitted to stir for 1 h. The solution was diluted with Et_2O, washed sequentially with saturated aqueous Na_2CO_3 (3×), brine, and dried over $MgSO_4$. The solvent was removed under reduced pressure and the residue was purified by column chromatography (1:1 EtOAc:Hexane) to give the title compound (69.1 mg, 74%, 95:5 d.r.) as a mixture of diastereomers as a colourless oil. $[\alpha]_D^{20}$–55.5 (*c* 2.88 in $CHCl_3$).

Preparation from maleate:

The title compound was prepared according to **General Procedure 2**. A mixture of ethyl (4-nitrophenyl) maleate **24** (53.0 mg, 0.2 mmol), (2*S*,3*R*)-HyperBTM **1** (3.0 mg, 10 µmol) and methyl 1-acetyl-3-oxoindoline-2-carboxylate **18** (46.6 mg, 0.2 mmol) in DMA (0.8 mL) was stirred for 16 h at room temperature. Benzylamine (22 µL, 0.2 mmol) was then added, and the reaction was permitted to stir for 1 h. The solution was diluted with Et_2O, washed sequentially with saturated aqueous Na_2CO_3 (3×), brine, and dried over $MgSO_4$. The solvent was removed under reduced pressure and the residue was purified by column chromatography (1:1 EtOAc:Hexane) to give the title compound (58.2 mg, 62%, 89:11 d.r.) as a mixture of diastereomers as a colourless oil.

Data for (2*R*,2'*S*)-**23**: $[\alpha]_D^{20}$–51.4 (*c* 1.2 in $CHCl_3$); **IR ν_{max}** (film) / cm^{-1} 3379, 2955, 2245, 1757, 1721, 1668, 1608, 1470, 1371, 1335, 1238, 1204, 1020, 910, 856; **Chiral HPLC analysis**: Chiralpak AD-H (80:20 hexane:IPA, flow rate 1.00 mL·min^{-1}, 254 nm, 30 °C) t_R (2*R*,2'*S*)-**23**: 22.5 min, t_R 33.3 min, 71:29 er, (2*S*,2'*S*)-**23** t_R (major): 29.2 min, t_R (minor): 17.8 min, 70:30 er; **^{1}H NMR** (500 MHz, $CDCl_3$) δ_H: 0.79 (0.33H, t, *J* 7.1, OCH_2CH_3), 0.94 (2.67, t, *J* 7.1, OCH_2CH_3), 2.48 (2.67H, s, $NCOCH_3$), 2.52 (0.33H, s, $NCOCH_3$), 2.92 (0.89H, dd, *J* 15.6, 11.6, $CHCH_AH_BCONH$), 2.99 (0.11H, dd, *J* 16.3, 10.2, CH_AH_BCONH), 3.22 (0.11H, dd, *J* 16.3, 1.8, $CHCH_AH_BCONH$), 3.42 (0.89H, dd, *J* 15.6, 2.8, $CHCH_AH_BCONH$), 3.57 (0.11H, dq, *J* 10.8, $OCH_AH_BCH_3$), 3.67 (0.33H, s, $COOCH_3$), 3.68 (2.67H, s, $COOCH_3$), 3.79 (0.89H, dq, *J* 10.8, 7.1, $OCH_AH_BCH_3$), 3.86 (0.89H, dq, *J* 10.9, 7.1, $OCH_AH_BCH_3$),

4.16 (0.11H, dd, *J* 10.2, 3.1, C*H*CH_2CONH), 4.31 (0.89H, dd, *J* 11.5, 2.8, C*H*CH_2CONH), 4.38 (0.89H, dd, *J* 14.9, 5.5, NHC$H_A$$H_B$Ph), 4.45 (0.89H, dd, *J* 14.8, 6.0, NHC$H_A$$H_B$Ph), 5.81 (0.11H, app t, *J* 5.2, N*H*), 5.93 (0.89H, app t, *J* 5.8, N*H*), 7.19-7.28 (4H, m, Ph$C^{2,4,6}$*H*, ArC^5*H*), 7.28-7.34 (2H, m, Ph$C^{3,5}$*H*), 7.50 (0.11H,), 7.67 (1.00H, app t, *J* 7.6, ArC^6*H*, minor obscured), 7.77 (0.89H, d, *J* 7.6, ArC^4*H*), 7.82 (0.11H, d, *J* 7.6, ArC^4*H*), ArC^7*H* shows coalescence signal between 7.56-8.00; **^{13}C{^{1}H} NMR** (126 MHz, $CDCl_3$) δ_C: 13.5 (OCH_2*C*H_3 (minor)), 14.0 (OCH_2*C*H_3 (major)), 25.5 (NCO*C*H_3 (major)), 25.8 (NCO*C*H_3 (minor)), 33.6 (CH*C*H_2CONH (major)), 35.2 (CH*C*H_2CONH (minor)), 43.6$_8$ (NH*C*H_2Ph (minor)), 43.7$_2$ (NH*C*H_2Ph (major)), 46.1 (*C*HCH_2CONH (major)), 47.7 (*C*HCH_2CONH (minor)), 53.4 (COO*C*H_3 (minor)), 53.6 (COO*C*H_3 (major)), 61.3 (O*C*H_2CH_3 (minor)), 61.4 (O*C*H_2CH_3 (major)), 73.1 (Ar*C*OC (major)), 75.7 (Ar*C*OC (minor)) 115.9 (ArC^7H (minor)), 124.4 (ArC^{3a} (major)), 124.6 (ArC^5H (major)), 125.4 (ArC^4H (major)), 125.7 (ArC^4H (minor)), 127.5 (PhC^4H (minor)), 127.6 (PhC^4H, (major)) 127.8 (Ph$C^{2,6}$H (minor)), 127.9 (Ph$C^{2,6}$H (major)), 128.7 (Ph$C^{3,5}$H (minor)), 128.8 (Ph$C^{3,5}$H (major)), 137.2 (ArC^6H (minor)), 137.6 (ArC^6H (major)), 138.2 (PhC^1 (major)), 138.4 (PhC^1 (minor)), 152.2 (ArC^{7a} (major)), 166.2 (*C*OOMe (minor)), 166.3 (*C*OOMe (major)), 168.2 (N*C*OMe (major)), 169.3 (N*C*OMe (minor)), 170.6 (*C*ONHBn (major)), 170.7 (*C*OOEt (major)), 191.9 (Ar*C*OC (minor)), 192.0 (Ar*C*OC (major)); **HRMS** (ESI^+) $C_{25}H_{27}O_7N_2$ $[M+H]^+$ found 467.1809, requires 467.1813 (–0.73 ppm).

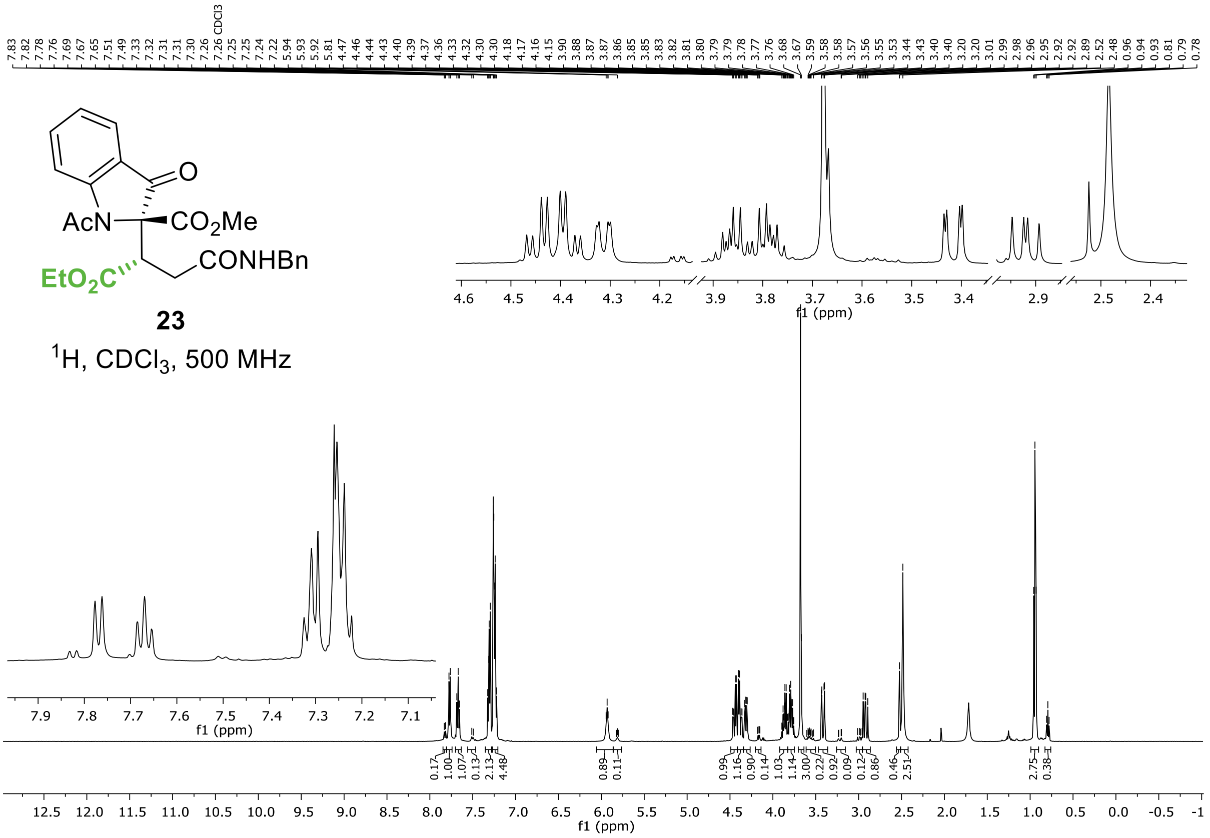

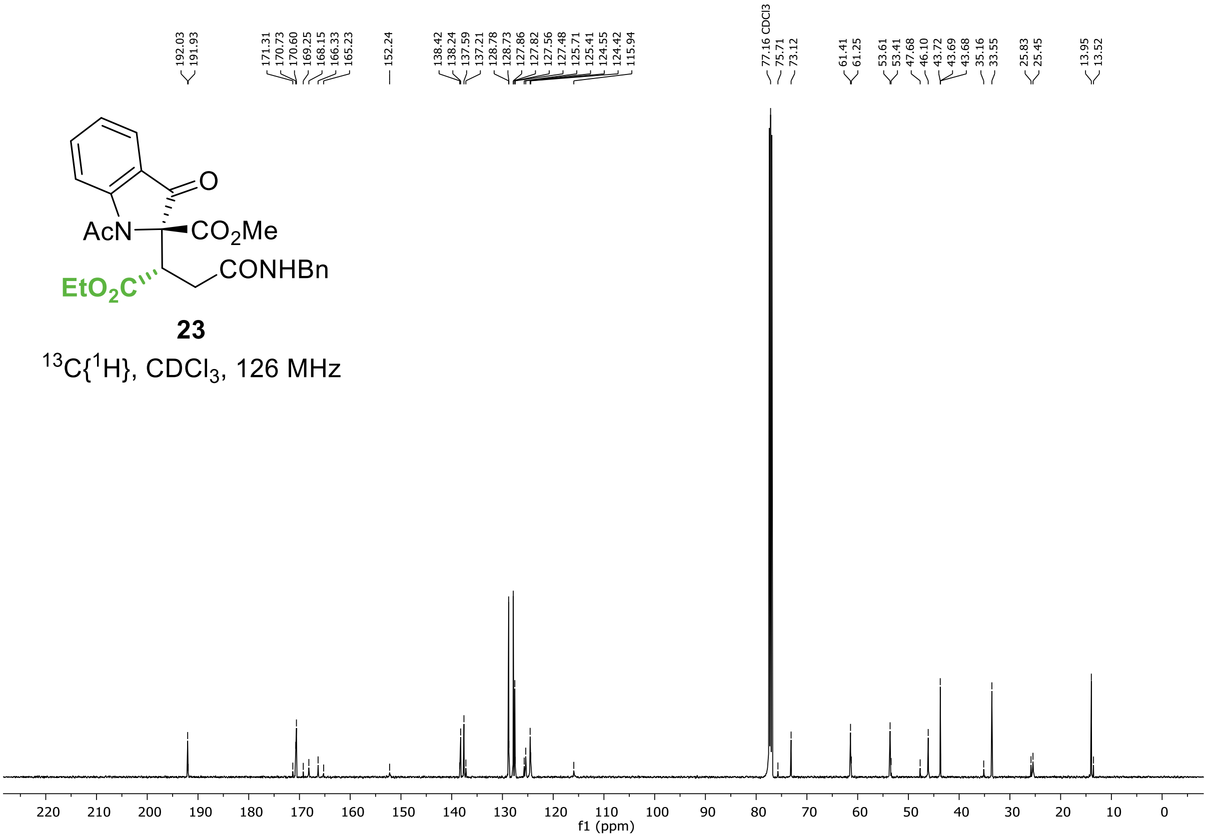

HPLC Data for (2*S*,2'*S*)-23 from fumarate: Chiralpak AD-H (80:20 hexane:IPA, flow rate 1.00 mL·min^{-1}, 254 nm, 30 °C) t_R (2*S*,2'*S*)-**23**: 32.7 min, t_R (2*R*,2'*R*)-**23**: 22.9 min, 92:8 er, t_R (*rel*-2*R*,2'*S*)-**23**: 28.8 min, t_R (*rel*-2*S*,2*R*)-**23**: 17.7 min, 72:28 er.

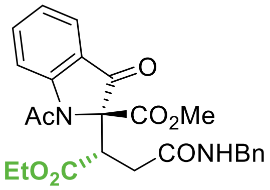

(2*S*,2'*S*)-**23**

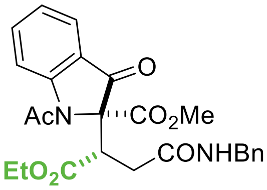

rel-(2*R*,2'*S*)-**23**

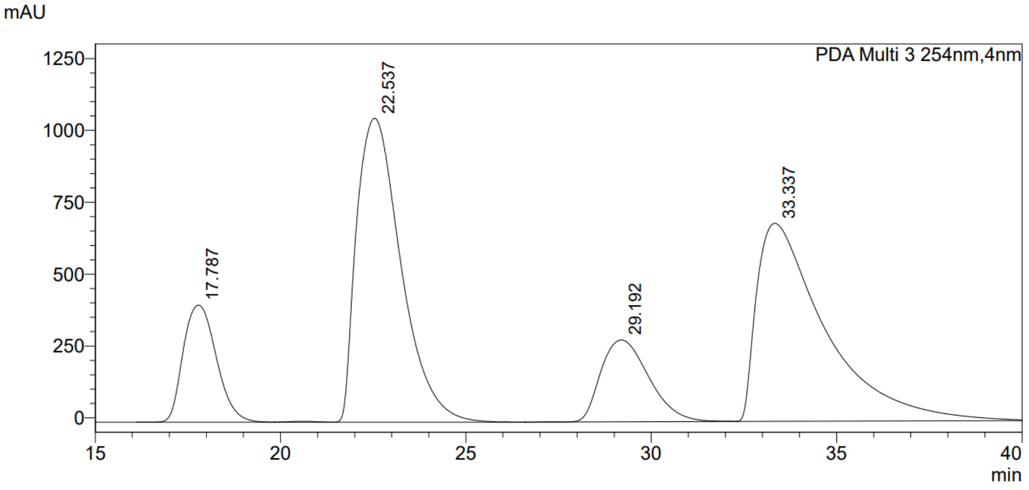

<Peak Table>

PDA Ch3 254nm

Peak#	Ret. Time	Area%
1	17.787	11.099
2	22.537	38.581
3	29.192	11.267
4	33.337	39.054
Total		100.000

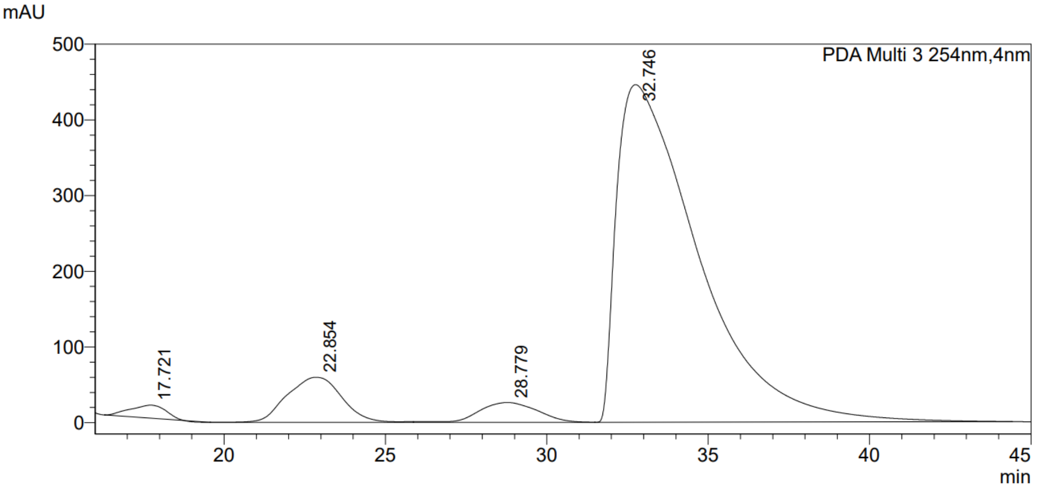

<Peak Table>

PDA Ch3 254nm

Peak#	Ret. Time	Area%
1	17.721	1.429
2	22.854	7.770
3	28.779	3.616
4	32.746	87.186
Total		100.000

HPLC Data for (2*R*,2'*R*)-23 from maleate: Chiralpak AD-H (80:20 hexane:IPA, flow rate 1.00 mL·min^{-1}, 254 nm, 30 °C) t_R (2*R*,2'*R*)-**23**: 22.5 min, t_R (2*S*,2'*S*): 33.3 min, 71:29 er, (2*R*,2'*S*)-**23**: t_R (major): 29.2 min, t_R (minor): 17.8 min, 66:34 er.

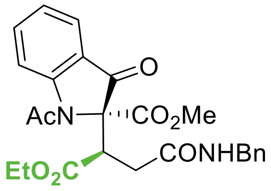

(2*S*,2'*S*)-**23**

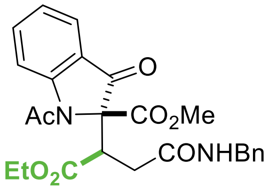

rel-(2*R*,2'*S*)-**23**

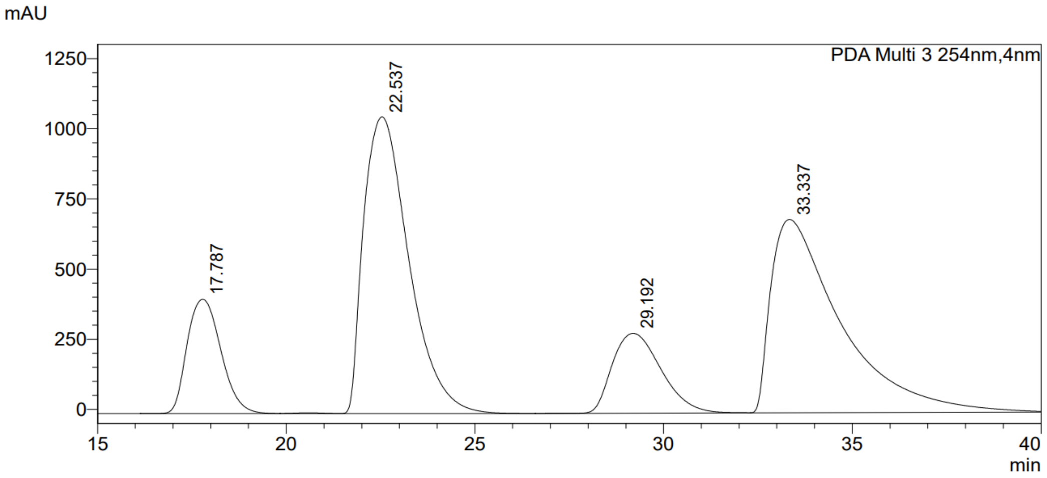

<Peak Table>

PDA Ch3 254nm

Peak#	Ret. Time	Area%
1	17.787	11.099
2	22.537	38.581
3	29.192	11.267
4	33.337	39.054
Total		100.000

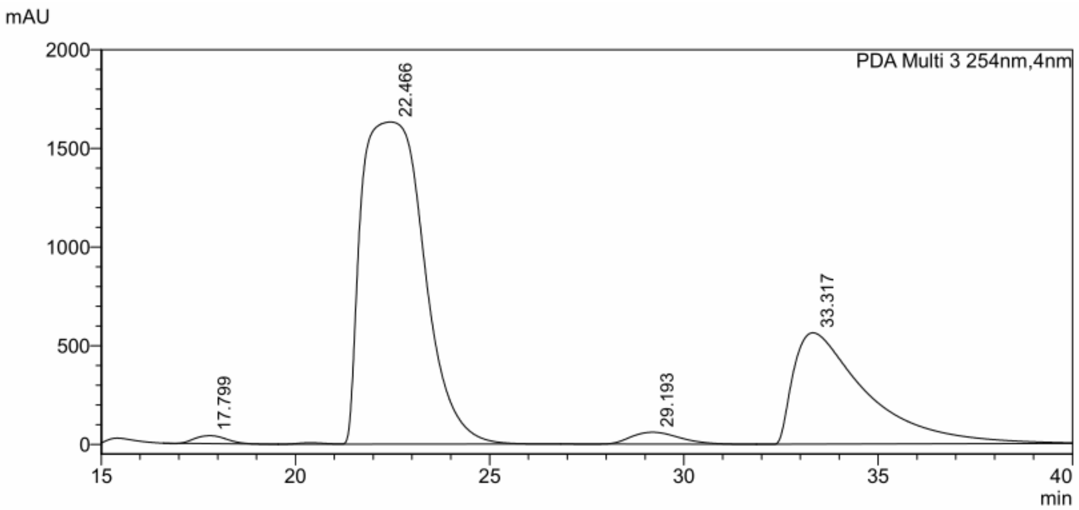

<Peak Table>

PDA Ch3 254nm

Peak#	Ret. Time	Area%
1	17.799	0.858
2	22.466	69.210
3	29.193	1.985
4	33.317	27.947
Total		100.000

(+)-Ethyl (*S*)-1-((*S*)-4'-(benzylamino)-1',1'-difluoro-4'-oxobutan-2'-yl)-2-oxocyclopentane-carboxylate (1*S*,2'*S*)-S7 and *rel*-(1*S*,2'*R*)-S7

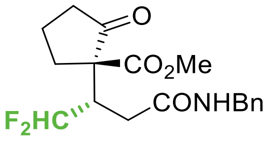

(1*S*,2'*S*)-**S7**

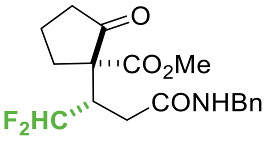

rel-(1*S*,2'*R*)-**S7**

The title compound was prepared according to **General Procedure 2**. A mixture of 4-nitrophenyl (*E*)-4,4-difluorobut-2-enoate **S4** (48.6 mg, 0.2 mmol), (2*S*,3*R*)-HyperBTM **1** (3.1 mg, 0.01 mmol) and methyl 2-oxocyclopentane-1-carboxylate **7** (31 µL, 0.2 mmol) in DMA (0.7 mL) was stirred for 16 h at room temperature. The solution was diluted with Et_2O, washed sequentially with saturated aqueous Na_2CO_3 (3×), brine, and dried over $MgSO_4$. The solvent was removed under reduced pressure and the residue was purified by column chromatography (1:3 EtOAc:Hexane) to give the title compound in two fractions (major diastereomer: 45.9 mg, 59% as a white solid, and minor diastereomer: 27.5 mg, 36% as a colourless oil); combined (73.4 mg, 95%, 62:38 d.r.).

Data for major diastereoisomer (1*S*,2'*S*)-**S7**: **mp** 69-73 °C; $[\alpha]_D^{20}$ +17.6 (*c* 0.75 in $CHCl_3$); **IR** ν_{max} (film) 3258, 3082, 2961, 2359, 1717, 1643, 1553, 1211, 1115, 980, 856; **^{1}H NMR** (500 MHz, $CDCl_3$) δ_H: 1.25 (3H, t, *J* 7.1, OCH_2CH_3), 1.85-1.96 (1H, m, $COCH_2CH_AH_B$), 1.96-2.06 (2H, m, CCH_AH_B, $COCH_2CH_AH_B$), 2.22 (1H, ddd, *J* 19.0, 10.5, 8.5, $COCH_AH_BCH_2$), 2.27 (1H, dd, *J* 15.5, 4.2,

$CHCH_AH_BCONH$), 2.46 (1H, app ddt, *J* 19.3, 9.3, 2.1, $COCH_AH_BCH_2$), 2.52 (2H, dd, J 15.5, 8.0, $CHCH_AH_BCONH$, $COCH_AH_BCH_2$ (obscured by previous)), 3.30 (1H, ddddd, *J* 20.7, 13.4, 8.0, 4.2, 2.1, $CHCH_2CONH$), 4.17 (2H, q, *J* 7.1, OCH_2CH_3), 4.41 (1H, dd, *J* 14.7, 5.3, $NHCH_AH_BPh$), 4.45 (1H, dd, *J* 14.7, 5.7, $NHCH_AH_BPh$), 5.90 (1H, app t, *J* 5.7, N*H*), 6.37 (1H, ddd, *J* 57.0, 55.5, 2.1, CHF_2), 7.24-7.30 (3H, m, $PhC^{2,4,6}H$), 7.30-7.36 (2H, m, $PhC^{3,5}H$); **$^{13}C\{^1H\}$ NMR** (126 MHz, $CDCl_3$) δ_C: 14.0 (OCH_2CH_3), 19.4 ($COCH_2CH_2$), 31.6 (CCH_2CH_2), 31.7 (t, *J* 4.5, $CHCH_2CONH$), 37.6 ($COCH_2CH_2$), 42.8 (t, *J* 19.7, $CHCH_2CONH$), 44.0 ($NHCH_2Ph$), 61.0 (d, *J* 5.0, CCH_2CH_2), 62.3 (OCH_2CH_3), 116.6 (app t, *J* 243.0, CHF_2), 127.7 (PhC^4H), 128.0 ($PhC^{2,6}H$), 128.9 ($PhC^{3,5}H$), 138.1 (PhC^1), 169.5 (*C*OOEt), 170.3 (*C*ONHBn), 213.4 ($COCH_2CH_2$); **^{19}F NMR** (471 MHz, $CDCl_3$) δ_F: −123.51 (1F, ddd, *J* 286.2, 56.5, 20.7, CHF_AF_B), −119.79 (1F, ddd, *J* 286.2, 55.4, 13.4, CHF_AF_B); **HRMS** (ESI^+) $C_{19}H_{24}F_2O_4N$ $[M]^+$ found 368.1660, requires 368.1668 (–2.2 ppm).

Data for minor diastereoisomer rel-(1*R*,2'*S*)-**S7**: $[\alpha]_D^{20}$ –3.76 (*c* 0.85 in $CHCl_3$); **IR ν_{max}** (film) 3298, 3088, 2980, 1753. 1659, 1557, 1225, 1061, 1026; **^1H NMR** (500 MHz, $CDCl_3$) δ_H: 1.24 (3H, t, *J* 7.1, OCH_2CH_3), 1.96 (1H, dddd, *J* 17.8, 13.0, 8.8, 7.3, $COCH_2CH_AH_B$), 2.06 (1H, dddd, *J* 17.8, 9.0, 7.2, 4.7, $COCH_2CH_AH_B$), 2.19 (1H, ddd, *J* 13.6, 9.0, 7.1, $CCH_AH_BCH_2$), 2.29-2.45 (2H, m, $COCH_2CH_2$), 2.42 (1H, dd, *J* 15.8, 6.8, $CHCH_AH_B$), 2.56 (1H, d, *J* 12.4, 7.0, 4.6, $CCH_AH_BCH_2$), 2.64 (1H, dd, *J* 15.9, 4.9, $CHCH_AH_BCONH$), 3.26 (1H, ddddd, *J* 16.3, 12.8, 6.8, 5.0, 4.9, $CHCH_2CONH$), 4.15 (2H, q, *J* 7.1, OCH_2CH_3), 4.40 (1H, dd, *J* 14.8, 5.7, $NHCH_AH_BPh$), 4.43 (1H, dd, *J* 14.8, 5.8, $NHCH_AH_BPh$), 5.90 (1H, app t, *J* 5.9, N*H*), 6.09 (1H, app td, *J* 56.4, 4.7, CHF_2), 7.24-7.30 (3H, m, $PhC^{2,4,6}H$), 7.30-7.35 (2H, m, $PhC^{3,5}H$); **$^{13}C\{^1H\}$ NMR** (126 MHz, $CDCl_3$) δ_C: 14.1 (OCH_2CH_3), 19.6 ($COCH_2CH_2$), 31.8 (d, *J* 2.6, CCH_2CH_2), 32.4 (t, *J* 4.7, $CHCH_2CONH$), 38.0 ($COCH_2CH_2$), 42.7 (t, *J* 19.8, $CHCH_2$), 43.9 ($NHCH_2Ph$), 60.7 (t, *J* 2.8, CCH_2CH_2), 62.3 (OCH_2CH_3), 117.1 (t, *J* 243.8, CHF_2), 127.7 (PhC^4H), 127.9 ($PhC^{2,6}H$), 128.8 ($PhC^{3,5}H$), 138.1 (PhC^7), 169.9 (*C*OOEt), 170.3 (*C*ONHBn), 213.1 ($COCH_2CH_2$); **^{19}F NMR** (471 MHz, $CDCl_3$) δ_F: −117.87 (1F, ddd, *J* 285.1, 56.0, 12.7, CHF_AF_B), −119.30 (1F, ddd, *J* 285.1, 56.7, 16.4, CHF_AF_B); **HRMS** (ESI^+) $C_{19}H_{24}F_2O_4N$ $[M+H]^+$ found 368.1660, requires 368.1668 (–2.0 ppm).

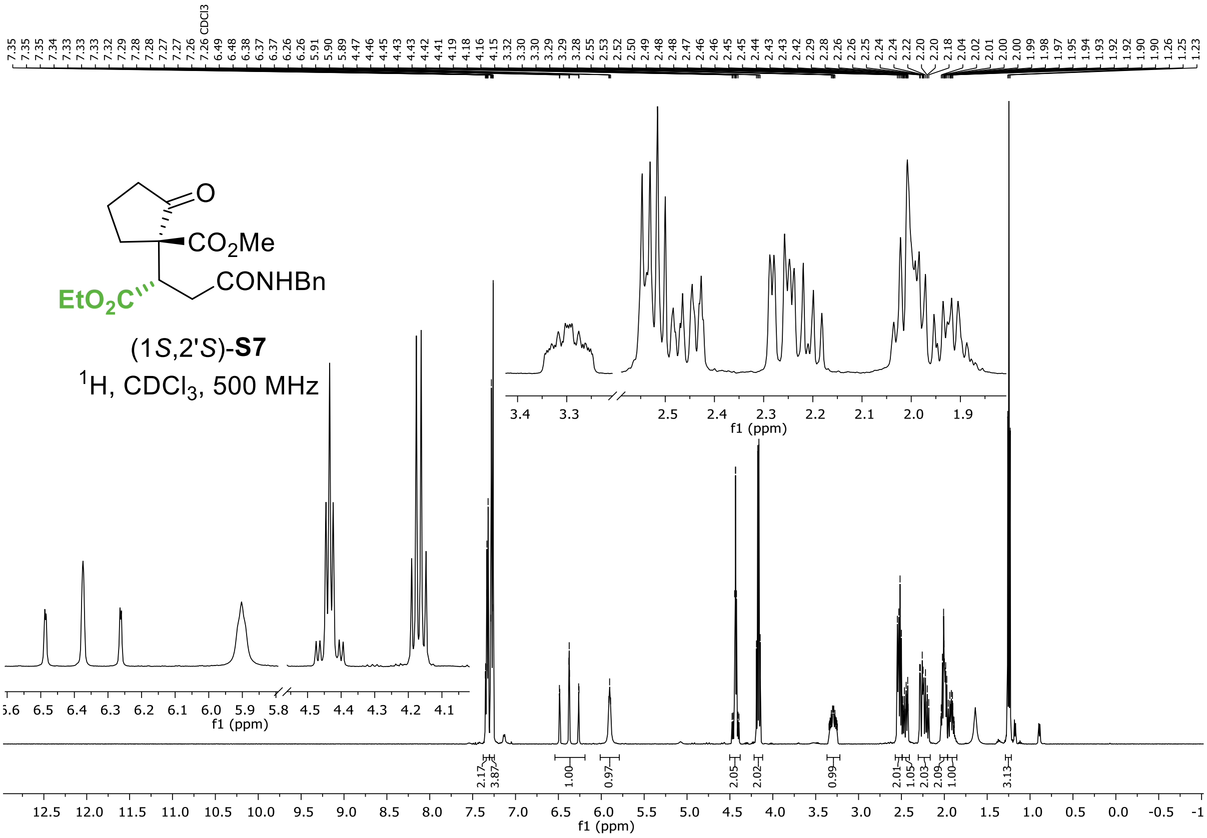
7.35 7.35 7.35 7.34 7.33 7.33 7.32 7.32 7.29 7.28 7.28 7.27 7.27 7.26 7.26 CDCl3 6.49 6.48 6.38 6.37 6.37 6.26 6.26 5.91 5.90 5.89 4.47 4.46 4.45 4.43 4.43 4.42 4.41 4.19 4.18 4.16 4.15 3.32 3.30 3.30 3.29 3.29 3.28 2.55 2.53 2.52 2.50 2.49 2.48 2.48 2.47 2.46 2.46 2.45 2.45 2.44 2.43 2.43 2.42 2.42 2.29 2.28 2.26 2.26 2.25 2.24 2.24 2.22 2.20 2.20 2.18 2.04 2.02 2.01 2.00 1.99 1.98 1.97 1.95 1.94 1.93 1.92 1.92 1.90 1.90 1.26 1.25 1.23
O
CO_2Me
CONHBn
EtO_2C
(1*S*,2'*S*)-**S7**
^1H, $CDCl_3$, 500 MHz
2.17 3.87 1.00 0.97 2.05 2.02 0.99 2.01 1.05 2.03 2.09 1.00 3.13
f1 (ppm)

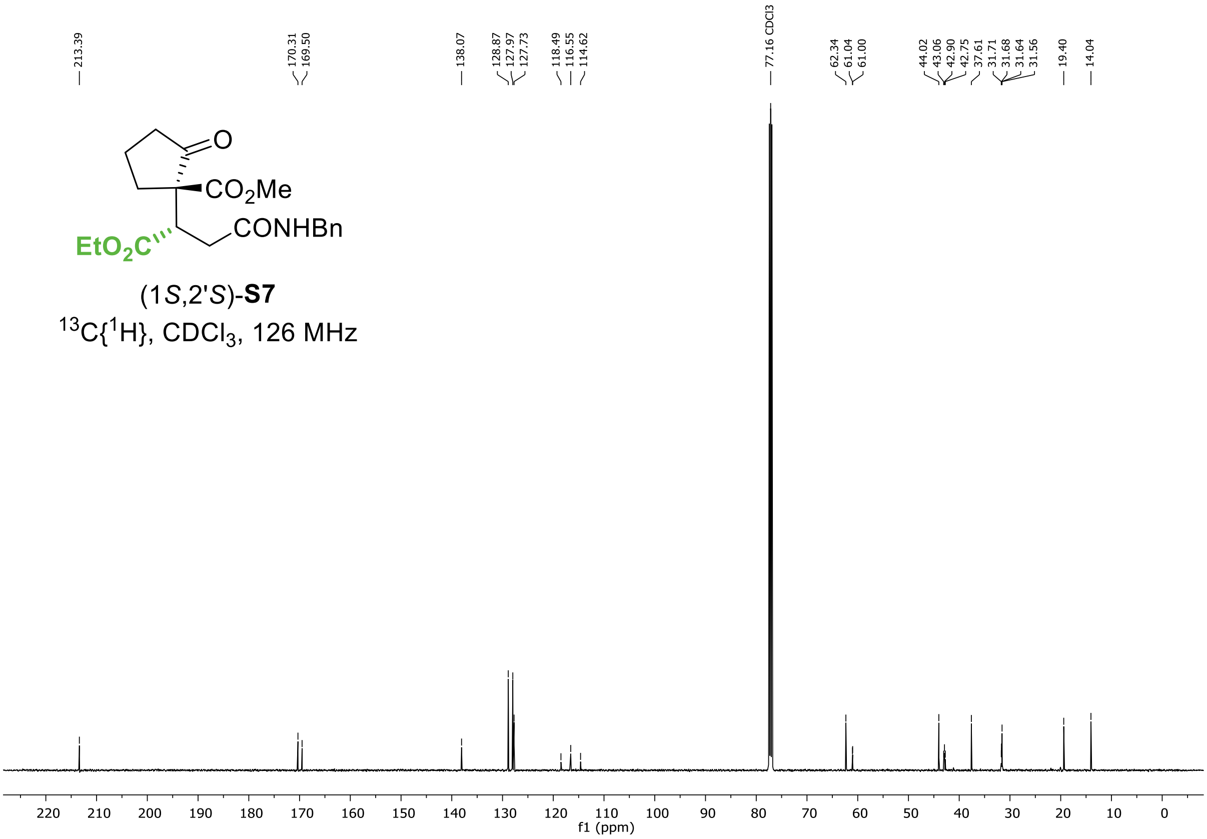
213.39 170.31 169.50 138.07 128.87 127.97 127.73 118.49 116.55 114.62 77.16 CDCl3 62.34 61.04 61.00 44.02 43.06 42.90 42.75 37.61 31.71 31.68 31.64 31.56 19.40 14.04
O
CO_2Me
CONHBn
EtO_2C
(1*S*,2'*S*)-**S7**
$^{13}C\{^1H\}$, $CDCl_3$, 126 MHz
f1 (ppm)

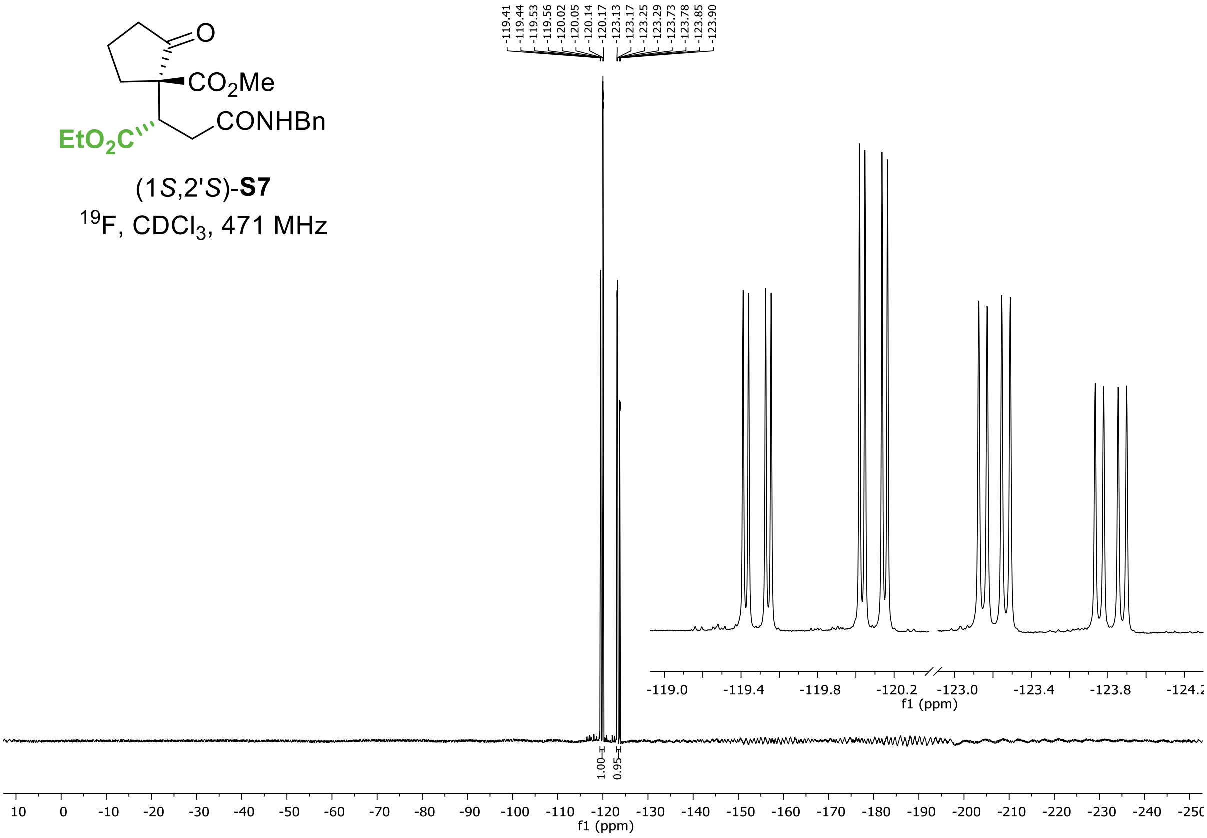
O
CO2Me
CONHBn
EtO2C
(1S,2'S)-S7
19F, CDCl3, 471 MHz
-119.41
-119.44
-119.53
-119.56
-120.02
-120.05
-120.14
-120.17
-123.13
-123.17
-123.25
-123.29
-123.73
-123.78
-123.85
-123.90
1.00
0.95
f1 (ppm)
f1 (ppm)

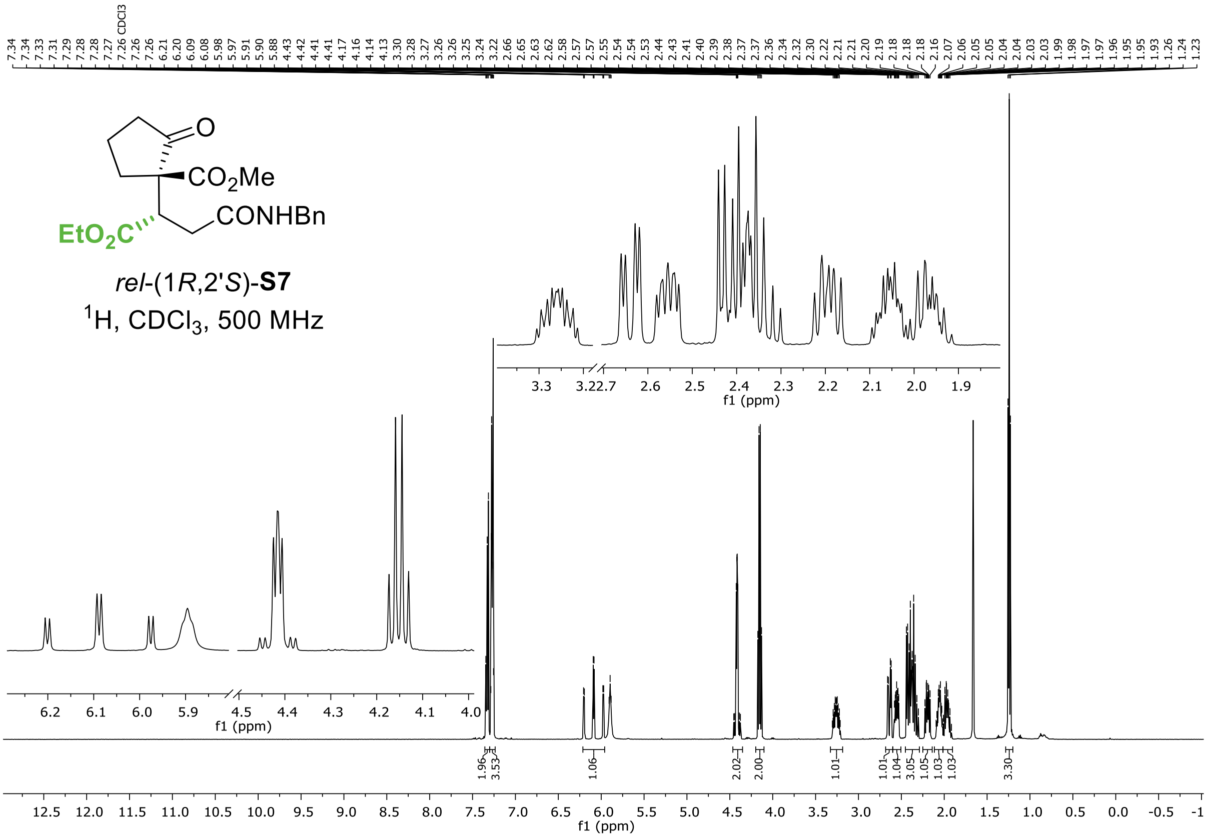

rel-(1*R*,2'*S*)-**S7**

^{1}H, $CDCl_3$, 500 MHz

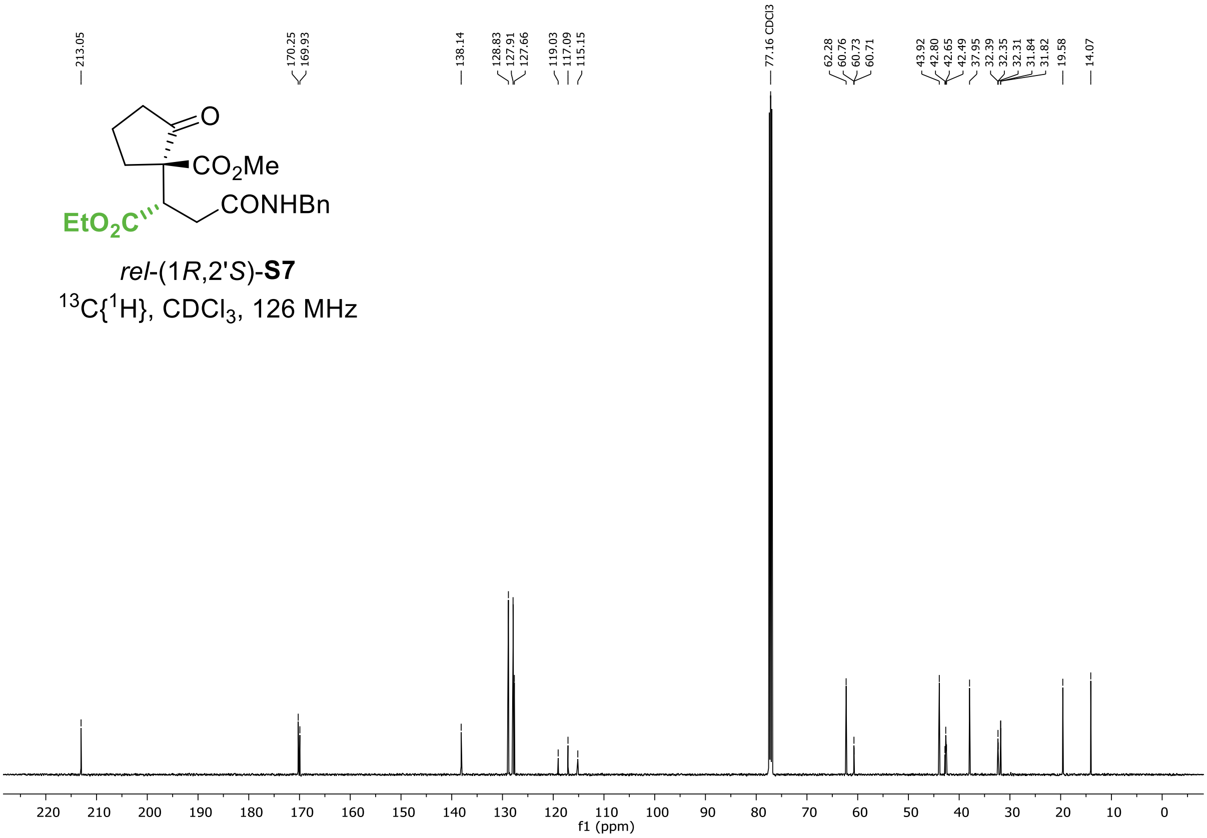

rel-(1*R*,2'*S*)-**S7**

$^{13}C\{^{1}H\}$, $CDCl_3$, 126 MHz

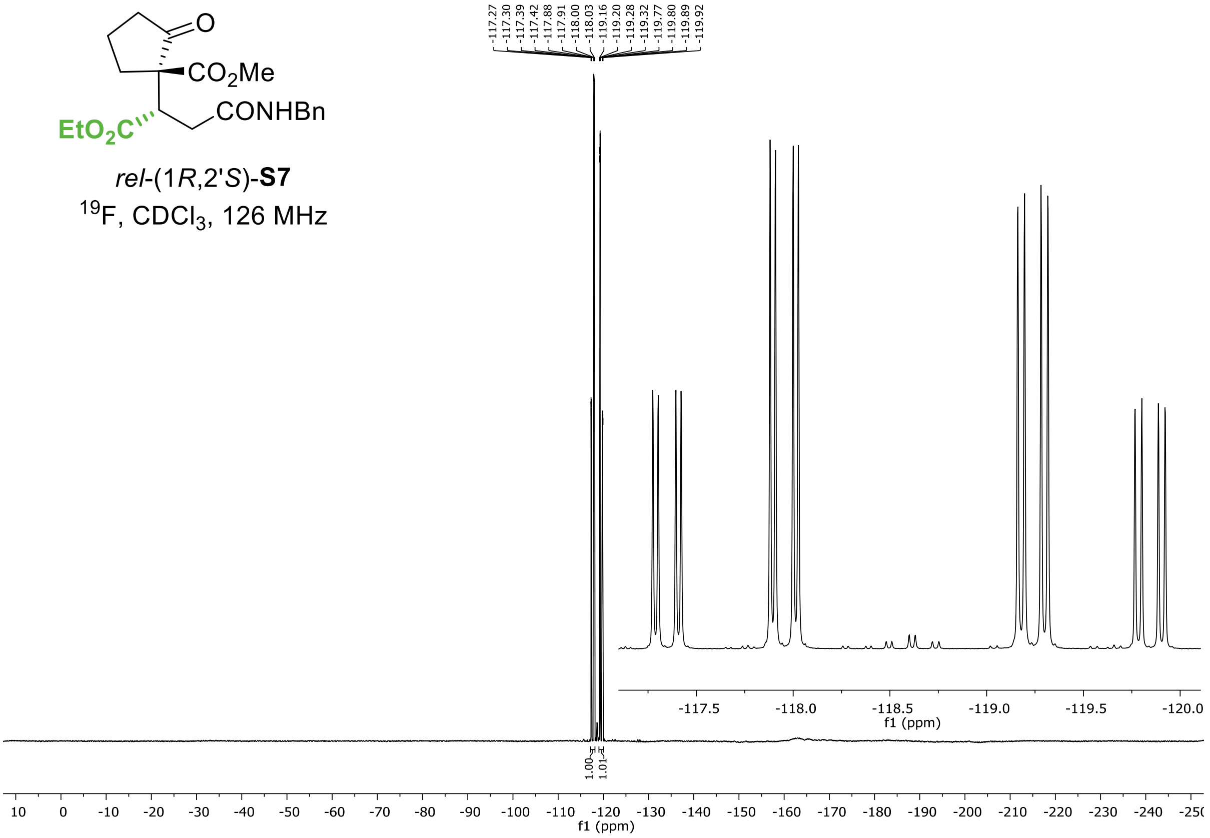

rel-(1*R*,2'*S*)-**S7**

^{19}F, $CDCl_3$, 126 MHz

HPLC Data for (1*S*,2'*S*)-**S7**: Chiralpak AD-H (90:10 hexane:IPA, flow rate 1.00 mL·min^{-1}, 254 nm, 30 °C) t_R (1*S*,2'*S*)-**S7**: 16.8 min, t_R (1*R*,2'*R*)-**S7**: 18.6 min, 36:64 er.

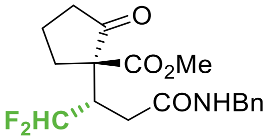

(1*S*,2'*S*)-**S7**

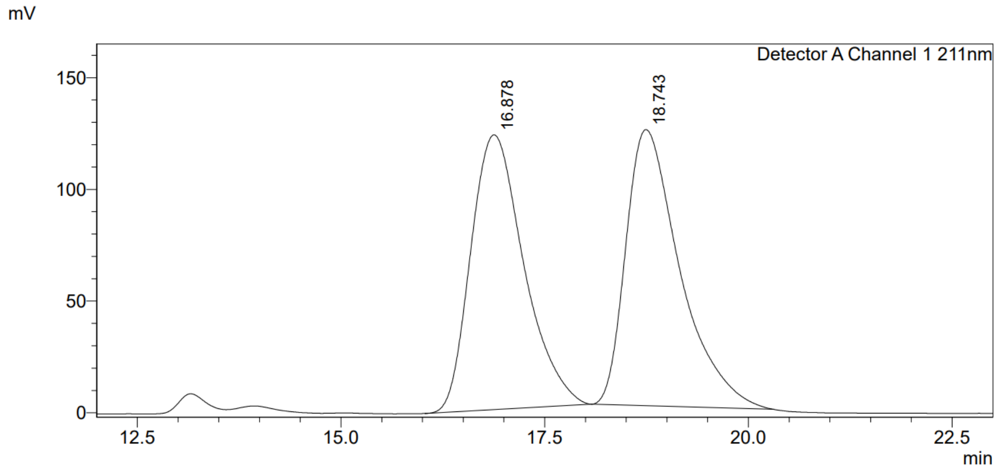

<Peak Table>

Detector A Channel 1 211nm

Peak#	Ret. Time	Area%
1	16.878	49.437
2	18.743	50.563
Total		100.000

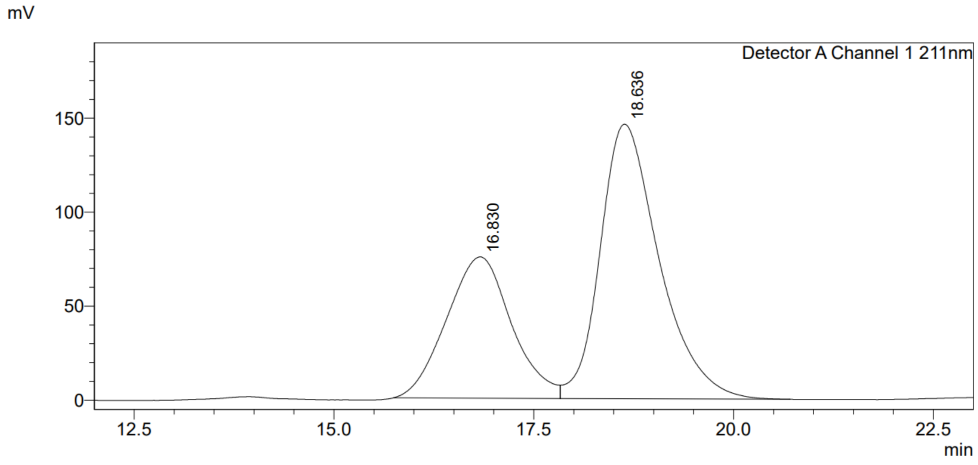

<Peak Table>

Detector A Channel 1 211nm

Peak#	Ret. Time	Area%
1	16.830	36.199
2	18.636	63.801
Total		100.000

HPLC Data for *rel*-(2*R*,2'*S*)-S7: Chiralpak AD-H (90:10 hexane:IPA, flow rate 1.00 mL·min^{-1}, 254 nm, 30 °C) t_R *rel*-(1*R*,2'*S*)-**S7**: 13.9 min, t_R *rel*-(1*S*,2'*R*)-**S7**: 18.8 min, 68:32 er.

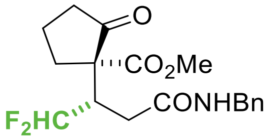

rel-(2*R*,2'*S*)-**S7**

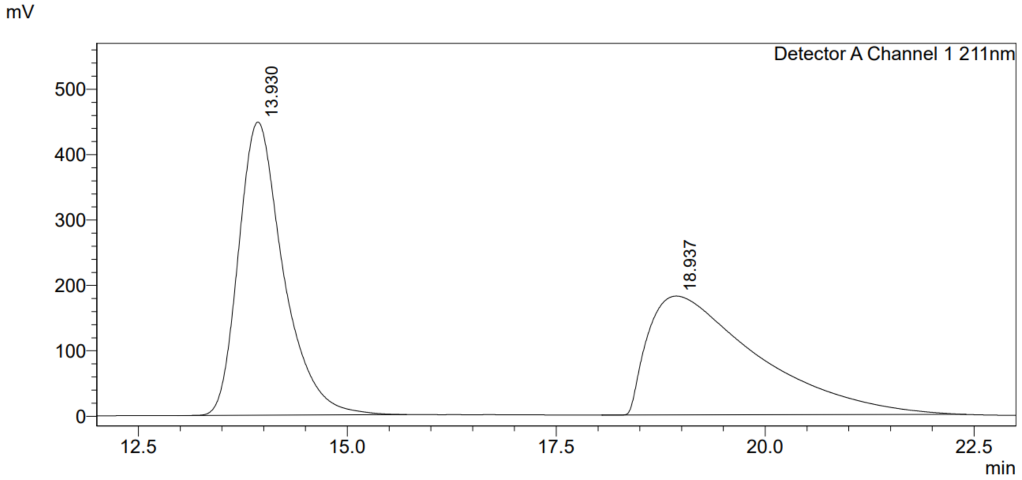

<Peak Table>

Detector A Channel 1 211nm

Peak#	Ret. Time	Area%
1	13.930	50.118
2	18.937	49.882
Total		100.000

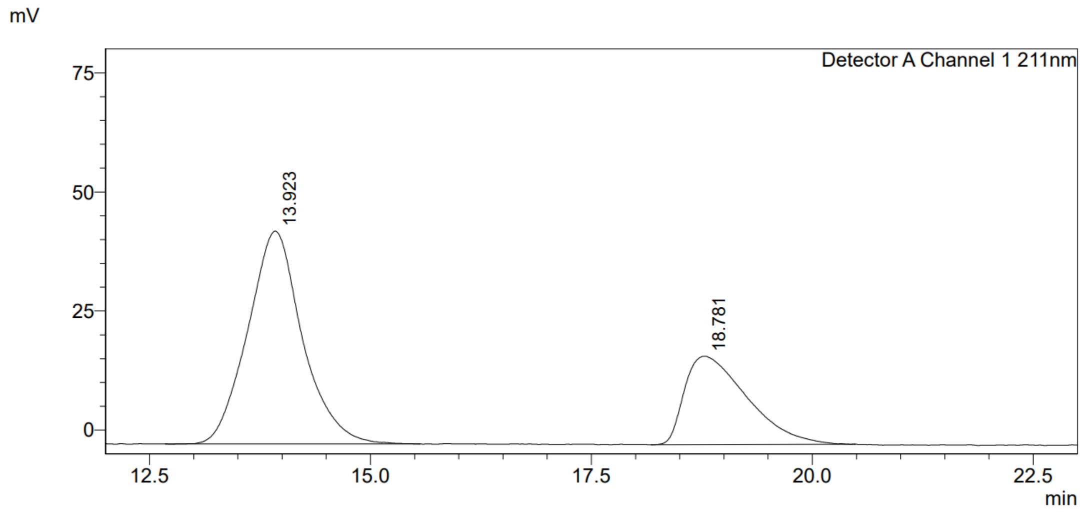

<Peak Table>

Detector A Channel 1 211nm

Peak#	Ret. Time	Area%
1	13.923	67.829
2	18.781	32.171
Total		100.000

(+)-(2*R*,3*R*) Ethyl 2-benzoyl-5-(benzylamino)-2-fluoro-5-oxo-3-(trifluoromethyl)pentanoate (2*R*,3*R*)-S8 and *rel*-(2*S*,3*R*)-S8

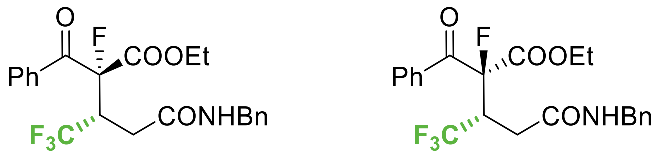

(3*R*,3*S*)-**S8** *rel*-(2*S*,3*R*)-**S8**

Analysed as a 67:33 mixture of diastereomers.

colorless oil; $[\alpha]_D^{20}$+0.25 (*c* 0.8 in $CHCl_3$); **IR ν$_{max}$** (film) / cm^{-1} 3302, 3067, 1755, 1653, 1541, 1231, 1128, 1028, 854; **^{1}H NMR** (500 MHz, $CDCl_3$) δ$_H$: 1.20 (3H, t, *J* 7.2, OCH_2CH_3), 2.45 (0.33H, dd, *J* 16.1, 3.0, CH_AH_BCONHBn), 2.56 (0.33H, dd, *J* 16.3, 8.1, CH_AH_BCONHBn), 2.60 (0.67H, dd, *J* 16.3, 5.4, CH_AH_BCONHBn), 2.65 (0.67H, dd, *J* 16.3, 6.6, CH_AH_BCONHBn), 4.07-4.33 (2H, m, OCH_2CH_3), 4.35-4.64 (2.33H, m, NHCH_2Bn, C*H*CF_3), 4.57 (0.67H, dqdd, *J* 29.5, 8.1, 6.6, 5.4, C*H*CF_3), 6.07 (0.33H, t (br), *J* 4.6, N*H*), 6.09 (0.67H, t (br), *J* 5.8, N*H*), 7.25-7.30 (3H, m, CH_2PhC^{2,4,6}*H*), 7.30-7.35 (2H, m, CH_2PhC^{3,5}*H*), 7.42-7.48 (2H, m, COPhC^{3,5}*H*), 7.57-8.63 (1H, m, COPhC^4*H*), 7.93-7.97 (1.34H, m, COPhC^{2,6}*H*), 8.03-8.07 (0.66H, m, COPhCC^{2,6}*H*); **^{13}C{^{1}H} NMR** (126 MHz, $CDCl_3$) δ$_C$ 13.7 (OCH_2CH_3, minor), 13.8 (OCH_2CH_3, major), 31.4 (CH_2CONHBn, major), 32.2 (CH_2CONHBn, minor), 44.1 (NHCH_2Ph, major and minor), 44.2 (qd, *J* 27.2, 19.8, *C*HCF_3, major), 44.6 (qd, *J* 27.1, 19.4, *C*HCF_3, minor), 63.7 (OCH_2CH_3, minor), 63.9 (OCH_2CH_3, major), 98.8 (d, *J* 207.3, CO*C*F, minor), 99.2 (d, *J* 208.7, CO*C*F, major), 125.5 (q, *J* 282.2, *C*F_3, major), 126.1 (q, *J* 281.6, *C*F_3, minor), 127.7

(CH_2PhC^4H, minor), 127.7 (CH_2PhC^4H, major), 127.9 ($CH_2PhC^{2,6}H$, major and minor), 128.7 ($COPhC^{3,5}H$, major), 128.8_1 ($CH_2PhC^{3,5}H$, minor), 128.8_4 ($COPhC^{3,5}H$, major), 128.9 ($CH2PhC^{3,5}H$, minor), 129.8 (d, *J* 6.6, $COPhC^{2,6}H$, major), 130.2 (d, *J* 6.3, $CH_2PhC^{2,6}H$, minor), 133.0 (d, *J* 3.5, $COPhC^1$, minor), 133.7 (d, *J* 3.5, $COPhC^1$, major), 134.1 ($COPhC^4H$, major), 134.8 ($COPhC^4$, minor), 137.9 (CH_2PhC^1, major), 138.0 (CH_2PhC^1, minor), 165.0 (d, *J* 25.7, *C*OOEt, major), 165.3 (d, *J* 26.5, *C*OOEt, minor), 168.0 (*C*ONHBn, major), 168.3 (*C*ONHBn, minor), 189.3 (d, *J* 24.9, *C*OPh, minor), 190.9 (d, *J* 25.8, *C*OPh, major); **^{19}F NMR** (471 MHz, $CDCl_3$) δ_F: −169.02 (0.67F, dq, *J* 29.5, 11.5, C*F*), −167.97 (0.33F, dq, *J* 31.0, 8.6, C*F*), −66.44 (0.99F, app t, *J* 8.6, CF_3), −64.00 (2.01F, dd, *J* 11.8, 8.1, CF_3); **HRMS** (ESI^+) $C_{22}H_{21}F_4O_4NNa$ $[M]^+$ found 462.1299, requires 462.1304 (–2.2 ppm).

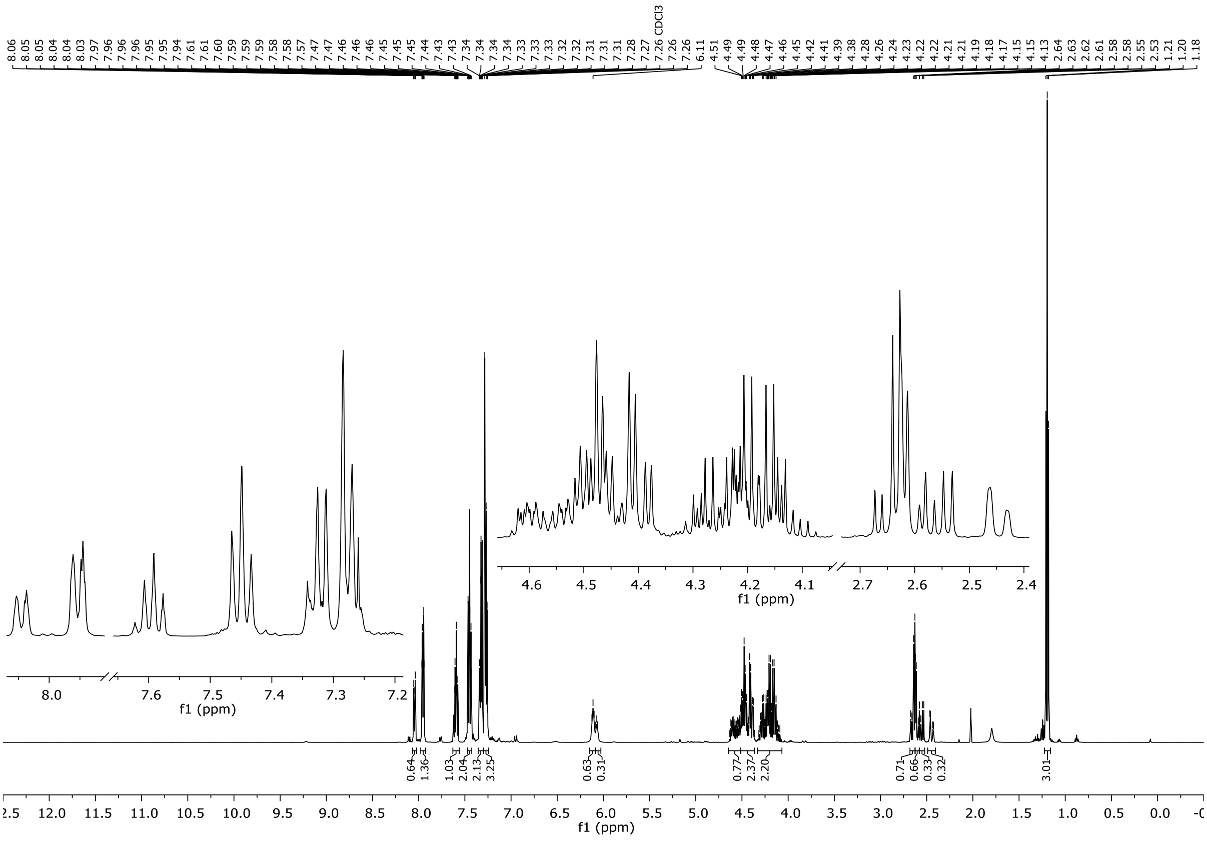

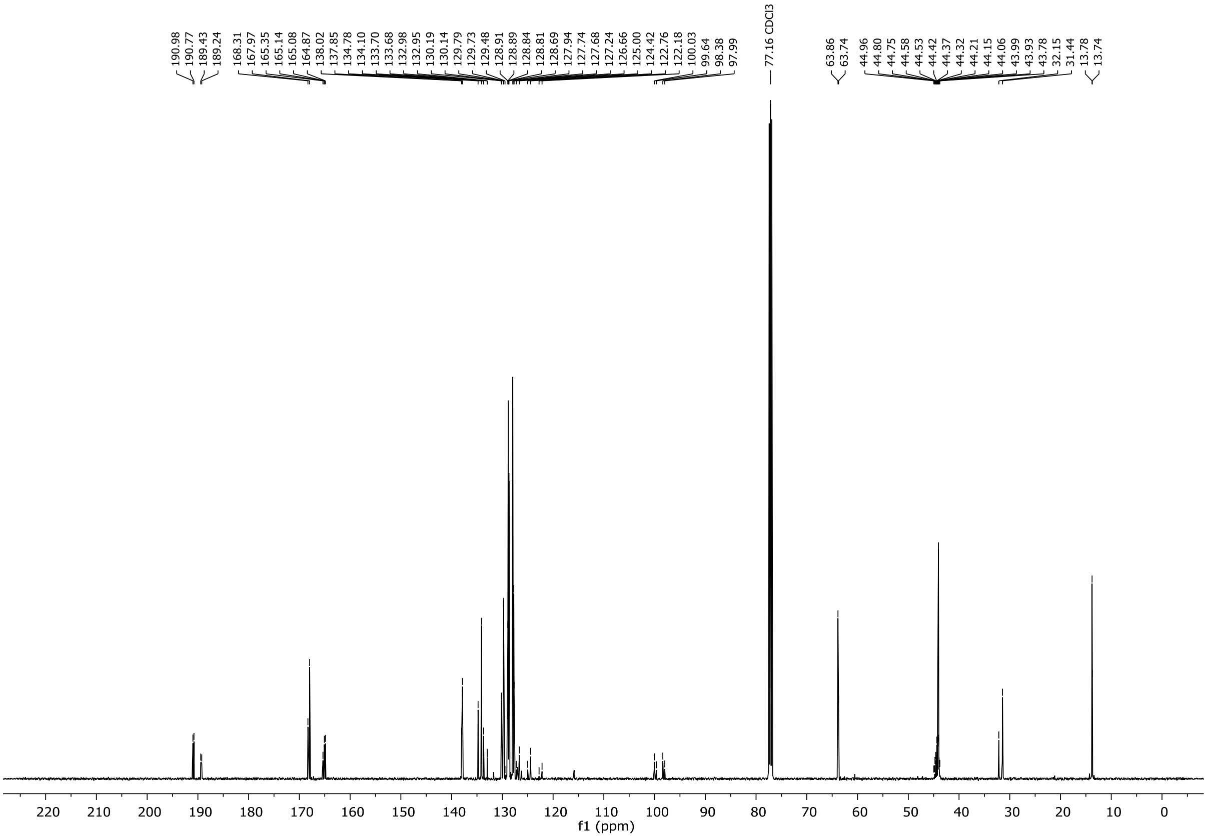
190.98
190.77
189.43
189.24
168.31
167.97
165.35
165.14
165.08
164.87
138.02
137.85
134.78
134.10
133.70
133.68
132.98
132.95
130.19
130.14
129.79
129.73
129.48
128.91
128.89
128.84
128.81
128.69
127.94
127.74
127.68
127.24
126.66
125.00
124.42
122.76
122.18
100.03
99.64
98.38
97.99
77.16 CDCl3
63.86
63.74
44.96
44.80
44.75
44.58
44.53
44.42
44.37
44.32
44.21
44.15
44.06
43.99
43.93
43.78
32.15
31.44
13.78
13.74
f1 (ppm)

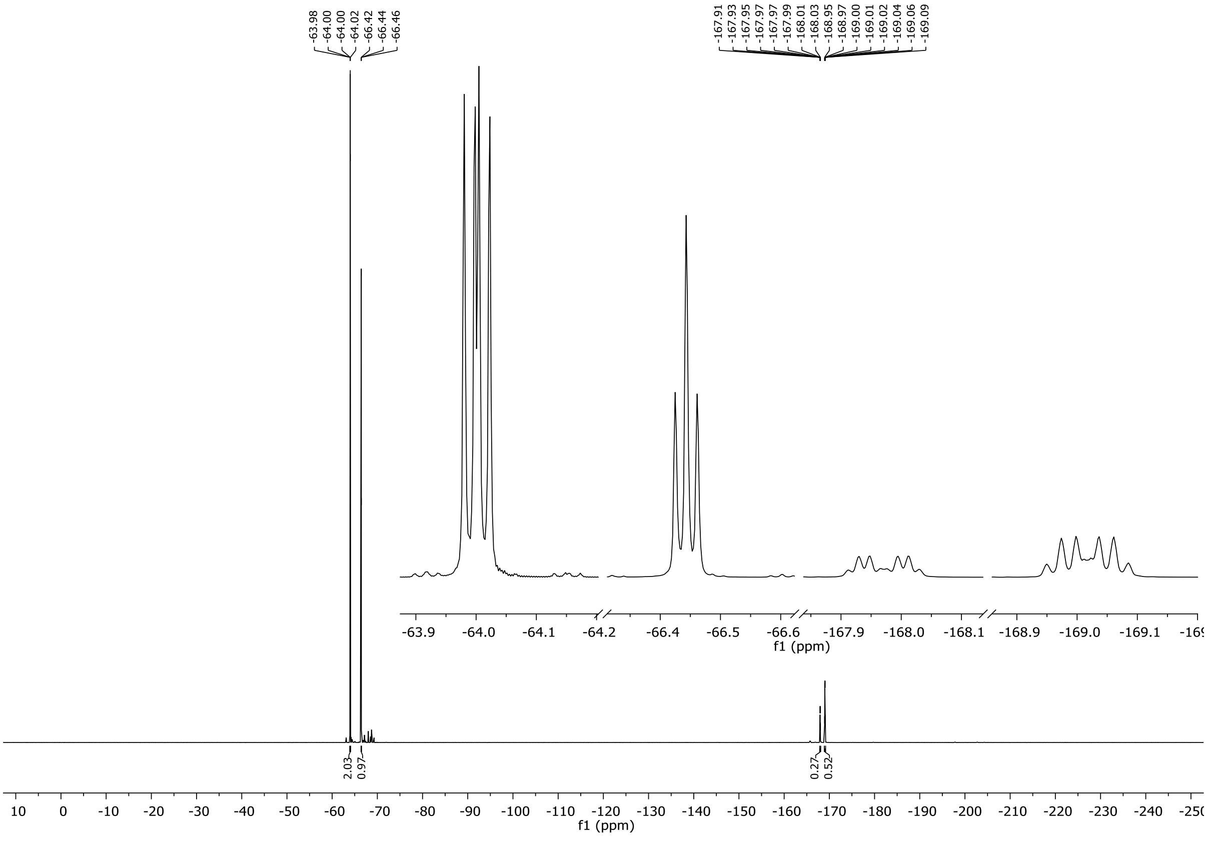
-63.98
-64.00
-64.00
-64.02
-66.42
-66.44
-66.46
-167.91
-167.93
-167.95
-167.97
-167.97
-167.99
-168.01
-168.03
-168.95
-168.97
-169.00
-169.01
-169.02
-169.04
-169.06
-169.09
2.03
0.97
0.27
0.52
f1 (ppm)

Chiral HPLC analysis: Chiralpak IA (95:5 hexane:IPA, flow rate 1.00 mL·min^{-1}, 254 nm, 30 °C) t_R (2*R*,2'*R*): 21.2 min, t_R (2*S*,2'*S*): 33.8 min, 98:2 er (2*R*,3*R*)-S8; t_R (major): 29.0 min, t_R (minor): 37.0 min, 95:5 er (*anti*-),

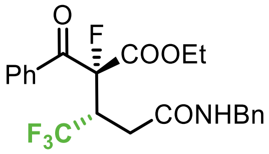

(3*R*,3*R*)-**S8**

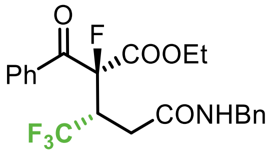

rel-(2*S*,3*R*)-**S8**

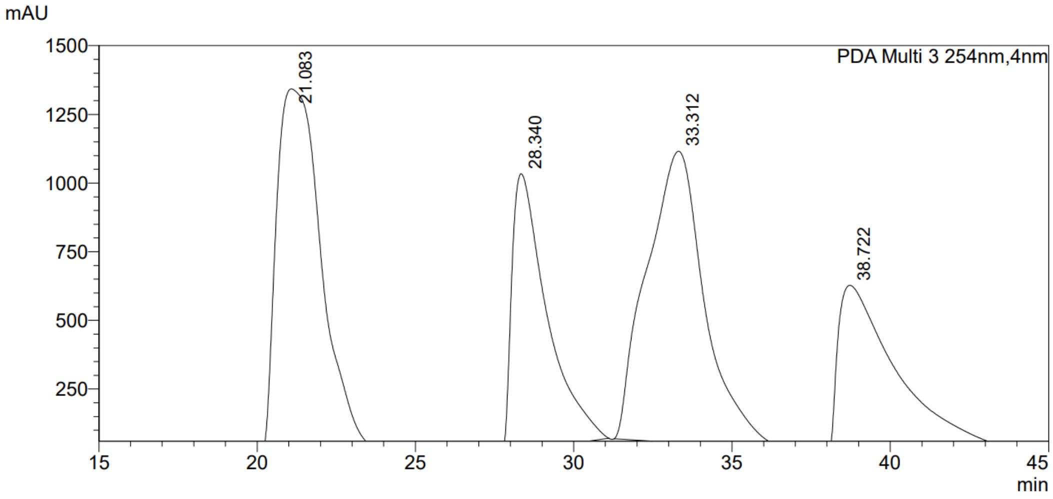

<Peak Table>

PDA Ch3 254nm

Peak#	Ret. Time	Area%
1	21.083	31.312
2	28.340	18.001
3	33.312	31.441
4	38.722	19.246
Total		100.000

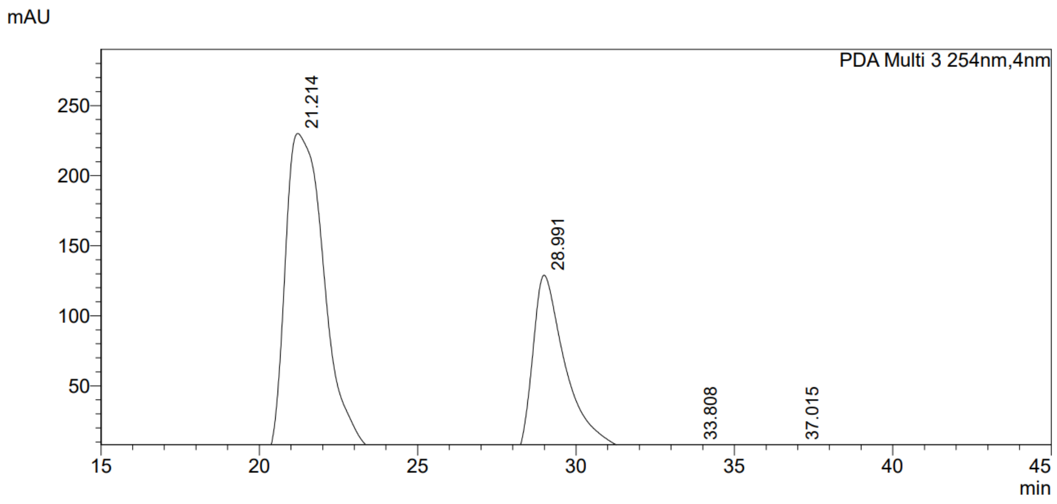

<Peak Table>

PDA Ch3 254nm

Peak#	Ret. Time	Area%
1	21.214	63.772
2	28.991	31.994
3	33.808	3.314
4	37.015	0.920
Total		100.000

Competition Experiments

Scheme 5C

A mixture of (4-nitrophenyl) fumarate **26** (79.5 mg, 0.3 mmol), (2*S*,3*R*)-HyperBTM **1** (4.6 mg, 15 µmol), methyl 2-oxocyclopentane-1-carboxylate **7** (44 µL, 0.3 mmol), methyl 1-acetyl-3-oxoindoline-2-carboxylate **18** (69.9 mg, 0.3 mmol) in DMA (1.2 mL) was stirred for 16 h at room temperature. Benzylamine (33 µL, 0.3 mmol) was then added, and the reaction was permitted to stir for 1 h. The solution was diluted with EtOAc, washed sequentially with saturated aqueous Na_2CO_3 (3×), brine, and dried over $MgSO_4$. The solvent was removed under reduced pressure. The ratio of product **19** and **13** was 86:14 calculated from the crude proton spectrum.

Scheme 5E

A mixture of methyl 1-acetyl-3-oxoindoline-2-carboxylate **18** (35.7 mg, 153 µmol), (2*S*,3*R*)-HyperBTM **1** (2.4 mg, 7.65 µmol)，4-nitrophenyl (*E*)-4,4,4-trifluorobut-2-enoate **8** (20 mg, 76.5 µmol), 4-nitrophenyl (*Z*)-4,4,4-trifluorobut-2-enoate **25** (20 mg, 76.5 µmol) in DMA (0.61 mL) was stirred for 16 h at room temperature. Benzylamine (16.7 µL, 153 µmol) was then added, and the reaction was permitted to stir for 1 h. The solution was diluted with EtOAc, washed sequentially with saturated aqueous Na_2CO_3 (3×), brine, and dried over $MgSO_4$. The solvent was removed under reduced pressure

and the residue was purified by column chromatography (1:1 EtOAc:Hexane) to give the title compound (38.9 mg, 55%, >95:5 dr, 74:26 er) as colourless oil.

HPLC Data for 19 from 1:1 (*E*)/(*Z*)-ester mixture: Chiralpak AD-H (90:10 hexane:^{i}PrOH, flow rate 1.00 mL·min^{-1}, 270 nm, 30 °C) t_R (2*S*,2'*S*)-**19**: 48.2 min, t_R (2*R*,2'*R*)-**19**: 70.8 min, 74:26 er.

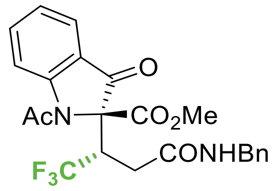

(2*S*,2'*S*)-**19**

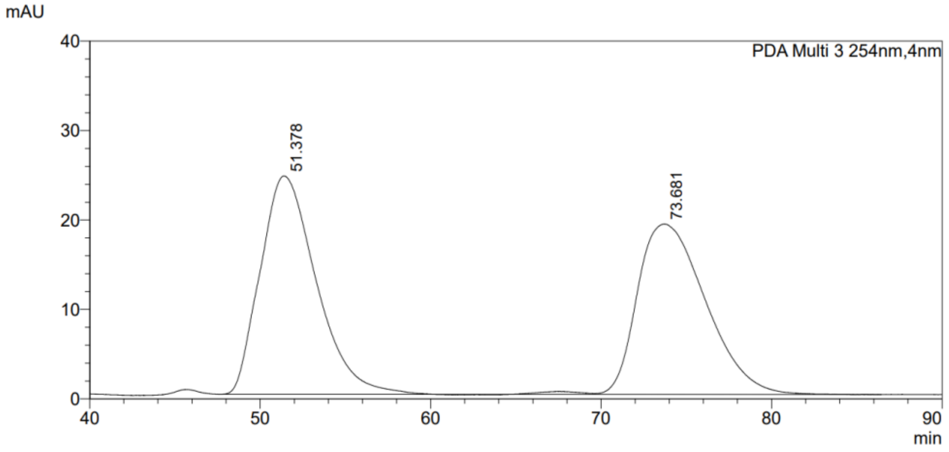

<Peak Table>

PDA Ch3 254nm

Peak#	Ret. Time	Area%
1	51.378	51.052
2	73.681	48.948
Total		100.000

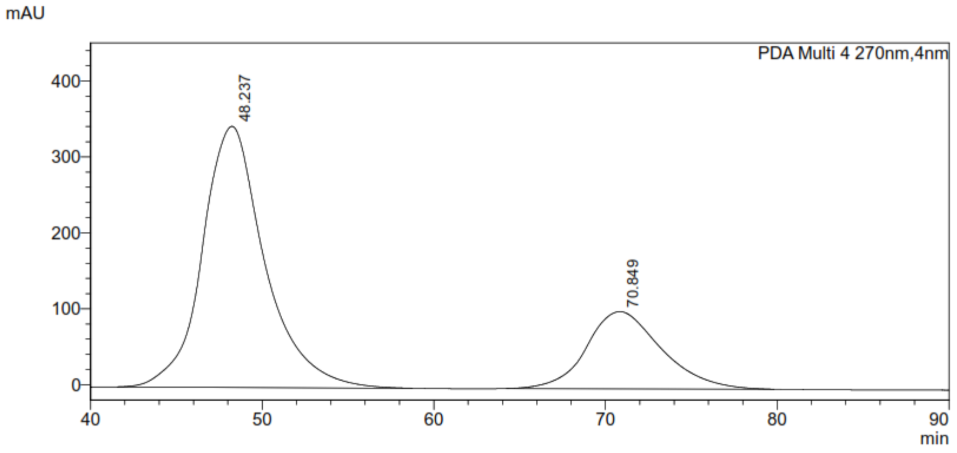

<Peak Table>

PDA Ch4 270nm

Peak#	Ret. Time	Area%
1	48.237	73.802
2	70.849	26.198
Total		100.000

Determination of Product Configuration by X-Ray Crystallography

X-ray diffraction data for all compounds were collected at low temperature using a Rigaku MM-007HF High Brilliance RA generator/confocal optics [Cu Kα radiation (λ = 1.54187 Å)] with XtaLAB P200 or P100 diffractometer. Intensity data for all compounds analysed were collected using either CrystalClear[56] (using either both ω and φ steps, or just ω steps, and accumulating area detector images spanning at least a hemisphere of reciprocal space) or CrysAlisPro[57] (using a calculated strategy), and processed (including correction for Lorentz, polarization and absorption) using CrysAlisPro. Structures were solved by direct (SIR2011)[58] or dual-space (SHELXT)[59] methods and refined by full-matrix least-squares against F^2 (SHELXL-2019/3).[60] Non-hydrogen atoms were refined anisotropically, and hydrogen atoms were refined using a riding model, except for nitrogen-bound hydrogens, which were located from the difference Fourier map and refined isotropically subject to a distance restraint. All calculations were performed using the Olex2[61] interface. Selected crystallographic data are presented in Table 1. CCDC 2298980-2298982 contains the supplementary crystallographic data for this paper. These data can be obtained free of charge from The Cambridge Crystallographic Data Centre via www.ccdc.cam.ac.uk/structures.

Table S1. Selected crystallographic data.

	**9**	**16**	**20**
CCDC	2298980	2298981	2298982

formula	$C_{19}H_{22}F_3NO_4$	$C_{23}H_{22}F_3NO_4$	$C_{23}H_{21}ClF_2N_2O_5$
fw	385.37	433.41	478.87
crystal description	Colourless prism	Colourless rod	Colourless prism
crystal size [mm^3]	0.18×0.15×0.13	0.49×0.09×0.09	0.10×0.09×0.05
temperature [K]	173	173	125
space group	$P2_12_12_1$	$P2_12_12_1$	$P2_12_12_1$
a [Å]	9.15002(4)	9.9425(2)	9.32874(6)
b [Å]	14.07250(7)	18.8834(3)	14.99320(9)
c [Å]	14.39225(7)	22.2420(4)	16.18409(11)
vol [Å]^3	1853.199(15)	4175.90(13)	2263.63(3)
Z	4	8	4
ρ (calc) [g/cm^3]	1.381	1.379	1.405
μ [mm^{-1}]	0.994	0.951	1.976
F(000)	808	1808	992
reflections collected	21885	50310	26847
independent reflections (R_{int})	3801 (0.0124)	7389 (0.0384)	4647 (0.0230)
parameters, restraints	249, 1	569, 2	304, 1
GoF on F^2	1.105	1.014	1.047
R_1 [$I > 2\sigma(I)$]	0.0315	0.0257	0.0254
wR_2 (all data)	0.0826	0.0677	0.0678
Flack x	0.025(18)	-0.02(3)	0.000(4)
largest diff. peak/hole [e/Å^3]	0.320, -0.279	0.116, -0.174	0.150, -0.333

checkCIF/PLATON report

Structure factors have been supplied for datablock(s) 16, 20, 9

THIS REPORT IS FOR GUIDANCE ONLY. IF USED AS PART OF A REVIEW PROCEDURE FOR PUBLICATION, IT SHOULD NOT REPLACE THE EXPERTISE OF AN EXPERIENCED CRYSTALLOGRAPHIC REFEREE.

No syntax errors found. CIF dictionary Interpreting this report

Datablock: 9

Bond precision: C-C = 0.0028 A Wavelength=1.54184

Cell:	a=9.15002(4)	b=14.07250(7)	c=14.39225(7)
	alpha=90	beta=90	gamma=90
Temperature:	173 K		

	Calculated	Reported
Volume	1853.199(15)	1853.199(15)
Space group	P 21 21 21	P 21 21 21
Hall group	P 2ac 2ab	P 2ac 2ab
Moiety formula	C19 H22 F3 N O4	C19 H22 F3 N O4
Sum formula	C19 H22 F3 N O4	C19 H22 F3 N O4
Mr	385.38	385.37
Dx,g cm-3	1.381	1.381
Z	4	4
Mu (mm-1)	0.994	0.994
F000	808.0	808.0
F000'	811.06	
h,k,lmax	11,17,18	11,17,18
Nref	3842[2196]	3801
Tmin,Tmax	0.836,0.879	0.957,1.000
Tmin'	0.836	

Correction method= # Reported T Limits: Tmin=0.957 Tmax=1.000
AbsCorr = MULTI-SCAN

Data completeness= 1.73/0.99 Theta(max)= 75.488

R(reflections)= 0.0315(3790) wR2(reflections)= 0.0826(3801)

S = 1.105 Npar= 249

	Calculated	Reported
Volume	4175.90(13)	4175.90(13)
Space group	P 21 21 21	P 21 21 21
Hall group	P 2ac 2ab	P 2ac 2ab
Moiety formula	C23 H22 F3 N O4	C23 H22 F3 N O4
Sum formula	C23 H22 F3 N O4	C23 H22 F3 N O4
Mr	433.42	433.41
Dx,g cm-3	1.379	1.379
Z	8	8
Mu (mm-1)	0.951	0.951
F000	1808.0	1808.0
F000'	1814.67	
h,k,lmax	11,22,26	11,22,26
Nref	7428[4161]	7389
Tmin,Tmax	0.902,0.918	0.925,1.000
Tmin'	0.634	

Correction method= # Reported T Limits: Tmin=0.925 Tmax=1.000
AbsCorr = MULTI-SCAN

Data completeness= 1.78/0.99 Theta(max)= 66.880

R(reflections)= 0.0257(7225) wR2(reflections)= 0.0677(7389)

S = 1.014 Npar= 569

The following ALERTS were generated. Each ALERT has the format
test-name_ALERT_alert-type_alert-level.
Click on the hyperlinks for more details of the test.

Alert level C

PLAT089_ALERT_3_C Poor Data / Parameter Ratio (Zmax < 18) .... 7.31 Note
PLAT911_ALERT_3_C Missing FCF Refl Between Thmin & STh/L= 0.596 5 Report

---

Alert level G

PLAT002_ALERT_2_G Number of Distance or Angle Restraints on AtSite 4 Note
PLAT172_ALERT_4_G The CIF-Embedded .res File Contains DFIX Records 1 Report
PLAT242_ALERT_2_G Low 'MainMol' Ueq as Compared to Neighbors of C21 Check
PLAT242_ALERT_2_G Low 'MainMol' Ueq as Compared to Neighbors of C51 Check
PLAT860_ALERT_3_G Number of Least-Squares Restraints .... 2 Note
PLAT909_ALERT_3_G Percentage of I>2sig(I) Data at Theta(Max) Still 92% Note
PLAT978_ALERT_2_G Number C-C Bonds with Positive Residual Density. 2 Info

---

0 **ALERT level A** = Most likely a serious problem - resolve or explain
0 **ALERT level B** = A potentially serious problem, consider carefully
2 **ALERT level C** = Check. Ensure it is not caused by an omission or oversight

7 **ALERT level G** = General information/check it is not something unexpected

0 ALERT type 1 CIF construction/syntax error, inconsistent or missing data
4 ALERT type 2 Indicator that the structure model may be wrong or deficient
4 ALERT type 3 Indicator that the structure quality may be low
1 ALERT type 4 Improvement, methodology, query or suggestion
0 ALERT type 5 Informative message, check

Datablock: 20

Bond precision: C-C = 0.0025 A Wavelength=1.54184

Cell:	a=9.32874(6)	b=14.99320(9)	c=16.18409(11)
	alpha=90	beta=90	gamma=90
Temperature:	125 K		

	Calculated	Reported
Volume	2263.63(2)	2263.63(3)
Space group	P 21 21 21	P 21 21 21
Hall group	P 2ac 2ab	P 2ac 2ab
Moiety formula	C23 H21 Cl F2 N2 O5	C23 H21 Cl F2 N2 O5
Sum formula	C23 H21 Cl F2 N2 O5	C23 H21 Cl F2 N2 O5
Mr	478.87	478.87
Dx,g cm-3	1.405	1.405
Z	4	4
Mu (mm-1)	1.976	1.976
F000	992.0	992.0
F000'	996.81	
h,k,lmax	11,18,20	11,18,20
Nref	4699[2667]	4647
Tmin,Tmax	0.821,0.906	0.867,1.000
Tmin'	0.821	

Correction method= # Reported T Limits: Tmin=0.867 Tmax=1.000
AbsCorr = MULTI-SCAN

Data completeness= 1.74/0.99 Theta(max)= 75.471

R(reflections)= 0.0254(4562) wR2(reflections)= 0.0678(4647)

S = 1.047 Npar= 304

The following ALERTS were generated. Each ALERT has the format
test-name_ALERT_alert-type_alert-level.
Click on the hyperlinks for more details of the test.

Alert level C

PLAT911_ALERT_3_C Missing FCF Refl Between Thmin & STh/L= 0.600 4 Report

---

Alert level G

PLAT002_ALERT_2_G Number of Distance or Angle Restraints on AtSite 2 Note
PLAT142_ALERT_4_G s.u. on b - Axis Small or Missing .... 0.00009 Ang.
PLAT143_ALERT_4_G s.u. on c - Axis Small or Missing .... 0.00011 Ang.
PLAT172_ALERT_4_G The CIF-Embedded .res File Contains DFIX Records 1 Report
PLAT850_ALERT_4_G Check Flack Parameter Exact Value 0.00 with s.u. 0.00 Check
PLAT860_ALERT_3_G Number of Least-Squares Restraints .... 1 Note
PLAT912_ALERT_4_G Missing # of FCF Reflections Above STh/L= 0.600 13 Note
PLAT913_ALERT_3_G Missing # of Very Strong Reflections in FCF .... 2 Note
PLAT961_ALERT_5_G Dataset Contains no Negative Intensities .... Please Check
PLAT978_ALERT_2_G Number C-C Bonds with Positive Residual Density. 6 Info

---

0 **ALERT level A** = Most likely a serious problem - resolve or explain
0 **ALERT level B** = A potentially serious problem, consider carefully
1 **ALERT level C** = Check. Ensure it is not caused by an omission or oversight
10 **ALERT level G** = General information/check it is not something unexpected

0 ALERT type 1 CIF construction/syntax error, inconsistent or missing data
2 ALERT type 2 Indicator that the structure model may be wrong or deficient
3 ALERT type 3 Indicator that the structure quality may be low
5 ALERT type 4 Improvement, methodology, query or suggestion
1 ALERT type 5 Informative message, check

The following ALERTS were generated. Each ALERT has the format
test-name_ALERT_alert-type_alert-level.
Click on the hyperlinks for more details of the test.

Alert level C

```
PLAT911_ALERT_3_C Missing FCF Refl Between Thmin & STh/L=    0.600           4 Report
PLAT918_ALERT_3_C Reflection(s) with I(obs) much Smaller I(calc) .           4 Check
PLAT934_ALERT_3_C Number of (Iobs-Icalc)/Sigma(W) > 10 Outliers ..           1 Check
PLAT939_ALERT_3_C Large Value of Not (SHELXL) Weight Optimized S .       18.78 Check
```

Alert level G

```
PLAT002_ALERT_2_G Number of Distance or Angle Restraints on AtSite           2 Note
PLAT142_ALERT_4_G s.u. on b - Axis Small or Missing .............      0.00007 Ang.
PLAT143_ALERT_4_G s.u. on c - Axis Small or Missing .............      0.00007 Ang.
PLAT172_ALERT_4_G The CIF-Embedded .res File Contains DFIX Records           1 Report
PLAT242_ALERT_2_G Low    'MainMol' Ueq as Compared to Neighbors of         C17 Check
PLAT860_ALERT_3_G Number of Least-Squares Restraints .............           1 Note
PLAT912_ALERT_4_G Missing # of FCF Reflections Above STh/L=  0.600          11 Note
PLAT913_ALERT_3_G Missing # of Very Strong Reflections in FCF ....           3 Note
PLAT961_ALERT_5_G Dataset Contains no Negative Intensities .......      Please Check
PLAT978_ALERT_2_G Number C-C Bonds with Positive Residual Density.           2 Info
```

0 **ALERT level A** = Most likely a serious problem - resolve or explain
0 **ALERT level B** = A potentially serious problem, consider carefully
4 **ALERT level C** = Check. Ensure it is not caused by an omission or oversight
10 **ALERT level G** = General information/check it is not something unexpected

0 ALERT type 1 CIF construction/syntax error, inconsistent or missing data
3 ALERT type 2 Indicator that the structure model may be wrong or deficient
6 ALERT type 3 Indicator that the structure quality may be low
4 ALERT type 4 Improvement, methodology, query or suggestion
1 ALERT type 5 Informative message, check

Datablock: 16

Bond precision:	C-C = 0.0028 A		Wavelength=1.54184
Cell:	a=9.9425(2)	b=18.8834(3)	c=22.2420(4)
	alpha=90	beta=90	gamma=90
Temperature:	173 K		

It is advisable to attempt to resolve as many as possible of the alerts in all categories. Often the minor alerts point to easily fixed oversights, errors and omissions in your CIF or refinement strategy, so attention to these fine details can be worthwhile. In order to resolve some of the more serious problems it may be necessary to carry out additional measurements or structure refinements. However, the purpose of your study may justify the reported deviations and the more serious of these should normally be commented upon in the discussion or experimental section of a paper or in the "special_details" fields of the CIF. checkCIF was carefully designed to identify outliers and unusual parameters, but every test has its limitations and alerts that are not important in a particular case may appear. Conversely, the absence of alerts does not guarantee there are no aspects of the results needing attention. It is up to the individual to critically assess their own results and, if necessary, seek expert advice.

Publication of your CIF in IUCr journals

A basic structural check has been run on your CIF. These basic checks will be run on all CIFs submitted for publication in IUCr journals (*Acta Crystallographica*, *Journal of Applied Crystallography*, *Journal of Synchrotron Radiation*); however, if you intend to submit to *Acta Crystallographica Section C* or *E* or *IUCrData*, you should make sure that full publication checks are run on the final version of your CIF prior to submission.

Publication of your CIF in other journals

Please refer to the *Notes for Authors* of the relevant journal for any special instructions relating to CIF submission.

PLATON version of 06/07/2023; check.def file version of 30/06/2023

Datablock 9 - ellipsoid plot

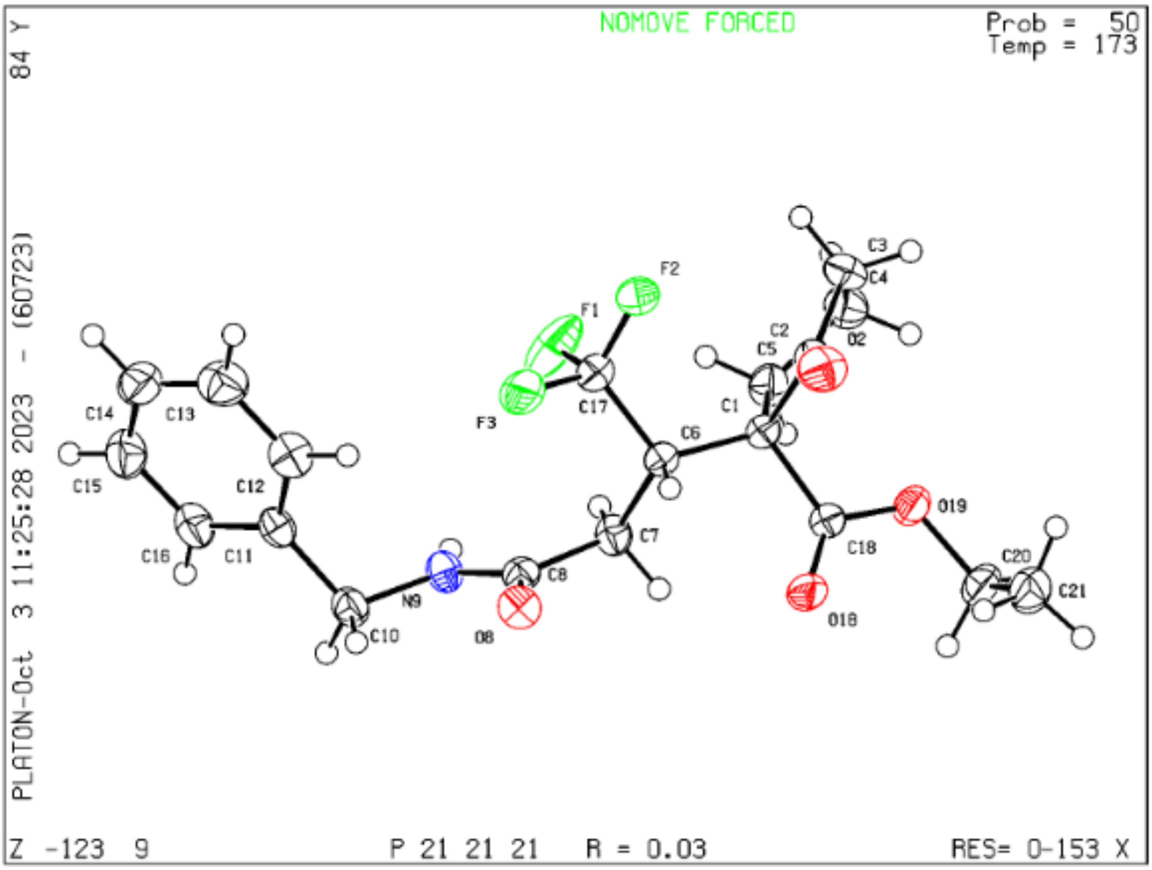

Datablock 16 - ellipsoid plot

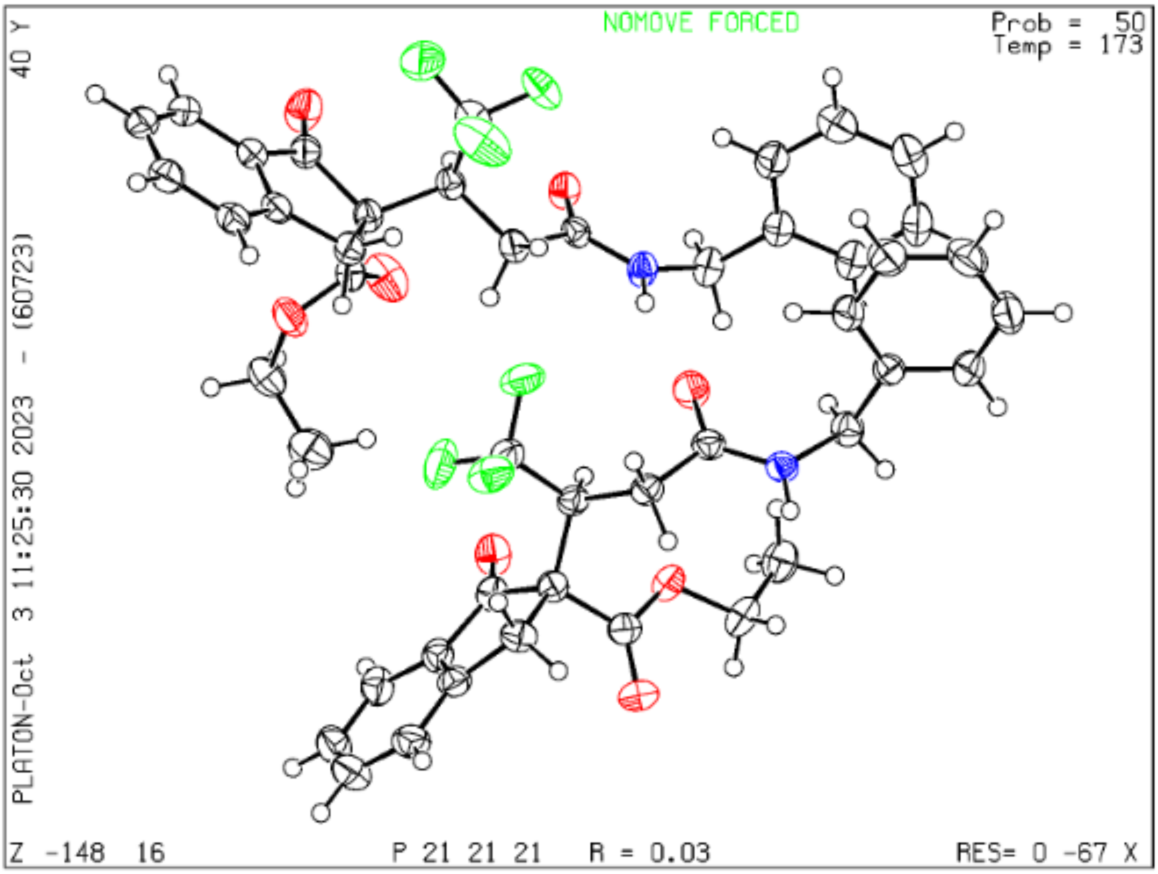

Datablock 20 - ellipsoid plot

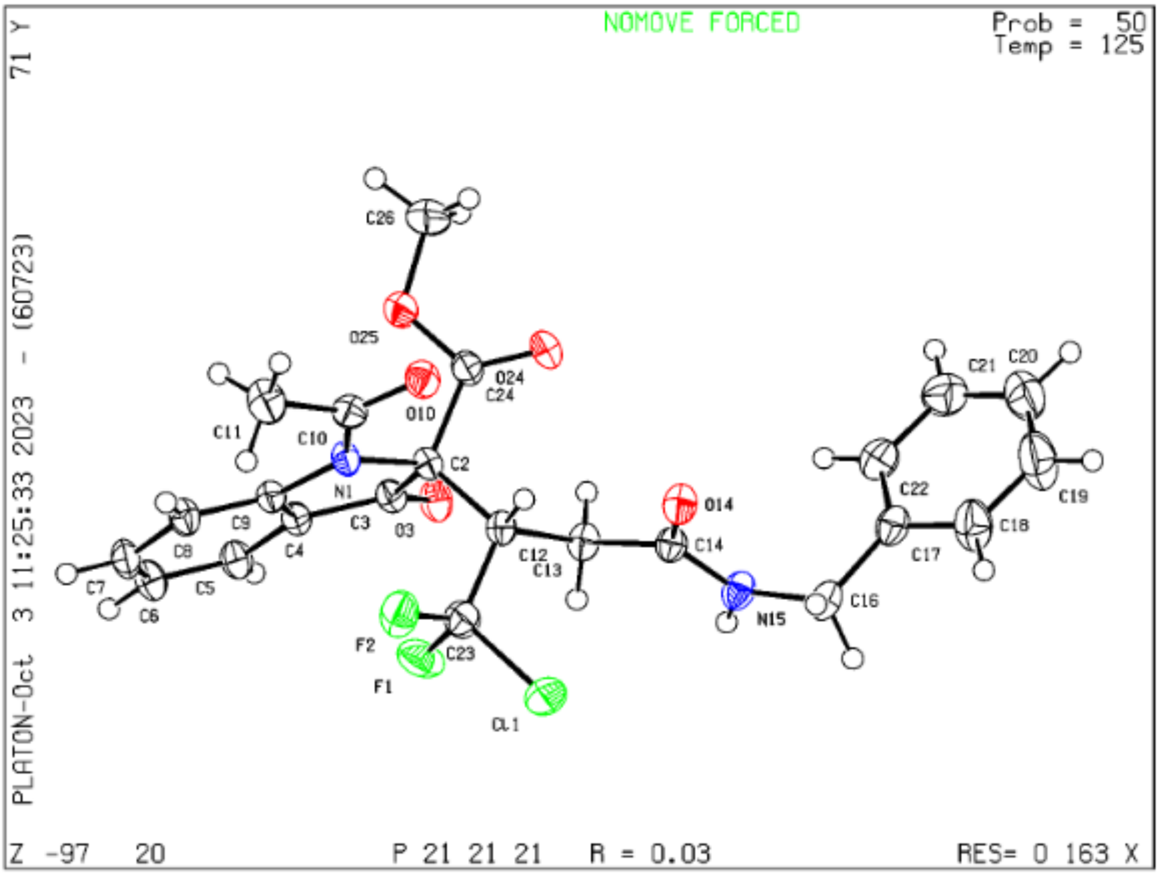

Computational Details

The DFT methodology was chosen based on previous work in the group,[62] originally based on methodology by Wang *et al*.[63] Geometry optimisations were performed with the *meta*-hybrid M06-2X functional[64] using the double-ζ, def2-SVP basis set from the redefinition of the Ahlrichs family of basis sets.[65-68] Implicit solvation was considered through the use of the SMD model employing the parameters of *N,N*-dimethylacetamide ($\varepsilon = 37.781$).[69] An ultrafine integration grid (99 radial shells with 590 angular points per shell) was used for all calculations and all species were formally treated as closed-shell systems with restricted Kohn-Sham DFT used throughout. The nature of minima and transition states located were verified by the computation of harmonic frequencies at the same level of theory. Single-point energies (E_{sp}) were also evaluated using the M06-2X functional[64] with a larger, triple-ζ, def2-TZVP basis. Implicit solvation was included at this level of theory using the same ultrafine integration grid (99,590). Additional empirical dispersion corrections were not included as the functional implicitly accounts for dispersion due to the nature of its construction. Preliminary calculations were performed using IEF-PCM[70, 71] as a solvent model and will be made clear where relevant. Thermochemistry was evaluated at 1 atm and 298.15 K using thermodynamic calculations at the level of geometry optimisation (thermal corrections to enthalpy, $dH_{298.15}$, and entropies $S_{298.15}$) in combination with energetics obtained from single-point calculations. Gibbs free energy was calculated at 298 K using Equation **1**. All

computations were performed using the Gaussian16, C.01 programme[72] with visualisation of structures using CYLview20[73] and GaussView6.1.1[72] and the visualisation of non-covalent interactions performed using NCIPlot 4.0[74] and VMD.[75]

$$G_{298.15} = E_{sp} + \mathrm{d}H_{298.15} - TS_{298.15} \qquad (1)$$

Conformational Flexibility (PCM//PCM)

Approach of the three substrates can be from either face of the catalyst, with either face of the substrate, with rotations around staggered conformations of the forming bond and with possible inversion of the ester group.

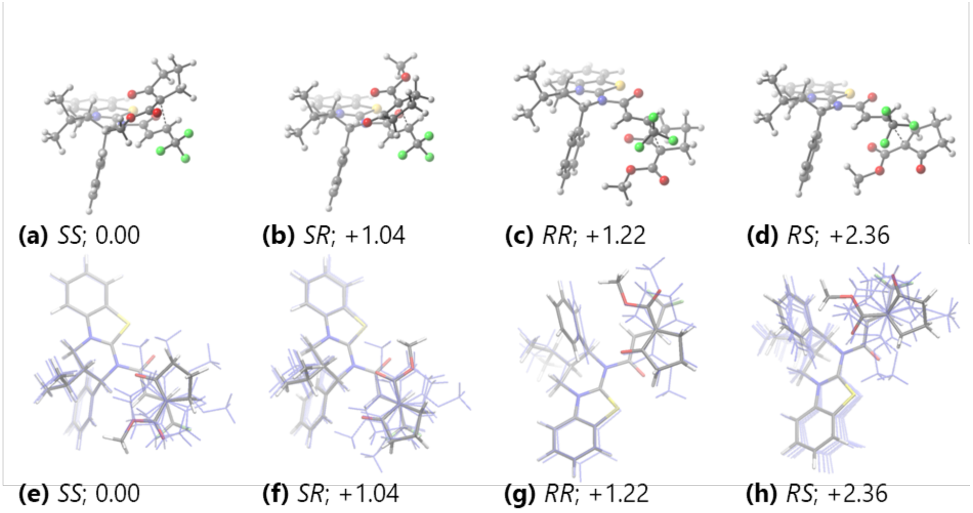

Figure S1 Transition states for the four different diastereoisomers of substrate **9** at the M06-2X$_{PCM}$/def2-TZVP//M06-2X$_{PCM}$/def2-SVP level. The most stable located transition state for each is highlighted **a-d**, with additional conformations shown as stick representations in **e-h**. Relative energies are shown as DDG$_{298}$ in kcal/mol.

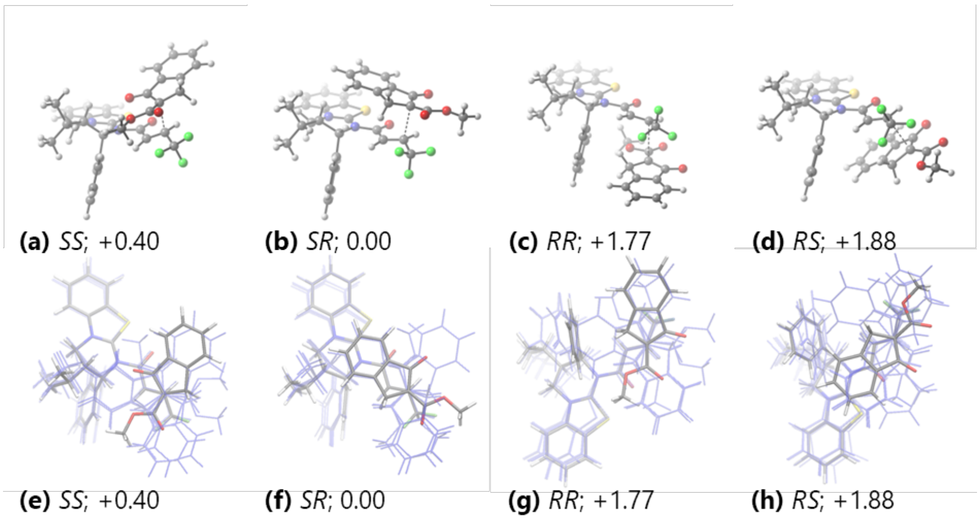

Figure S2 Transition states for the four different diastereoisomers of substrate **16** at the M06-2X$_{PCM}$/def2-TZVP//M06-2X$_{PCM}$/def2-SVP level. The most stable located transition state for each is highlighted **a-d**, with additional conformations shown as stick representations in **e-h**. *This leads to the incorrect identification of the major diastereomer*. Relative energies are shown as DDG$_{298}$ in kcal/mol.

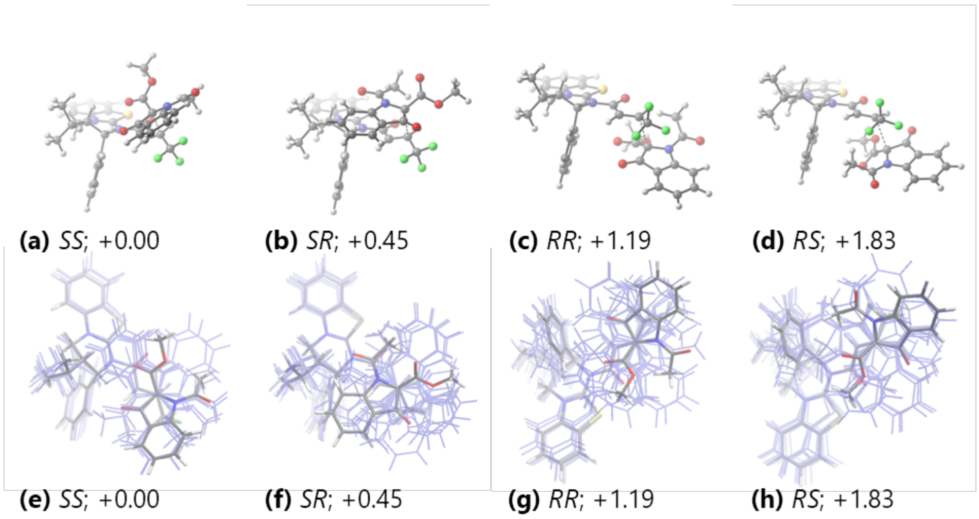

Figure S3 Transition states for the four different diastereoisomers of substrate **19** at the M06-2X$_{PCM}$/def2-TZVP//M06-2X$_{PCM}$/def2-SVP level. The most stable located transition state for each is highlighted **a-d**, with additional conformations shown as stick representations in **e-h**. Relative energies are shown as DDG$_{298}$ in kcal/mol.

Conformational Flexibility (SMD//SMD)

Following incorrect identification of one major diastereomer with PCM solvation, SMD was used as the solvent model to repeat the conformational search for the top-face approach.

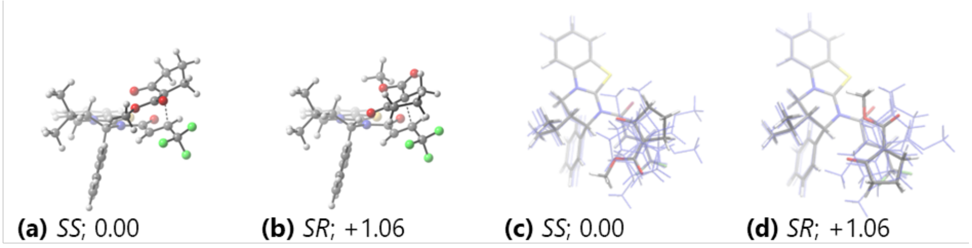

Figure S4 Transition states for the two diastereoisomers of substrate **9** arising from the top-face approach of the nucleophile (M06-2X$_{SMD}$/def2-TZVP//M06-2X$_{SMD}$/def2-SVP level). The most stable located transition state for each is highlighted **a**-**b**, with additional conformations shown as stick representations in **c-d**. Relative energies are shown as DDG$_{298}$ in kcal/mol.

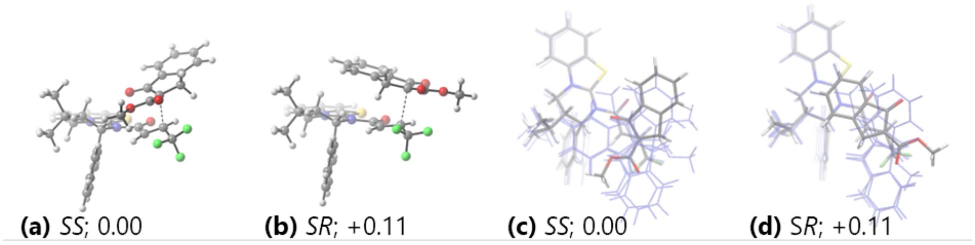

Figure S5 Transition states for the two diastereoisomers of substrate **16** arising from the top-face approach of the nucleophile (M06-2X$_{SMD}$/def2-TZVP//M06-2X$_{SMD}$/def2-SVP level). The most stable located transition state for each is highlighted **a**-**b**, with additional conformations shown as stick representations in **c**-**d**. Relative energies are shown as DDG$_{298}$ in kcal/mol.

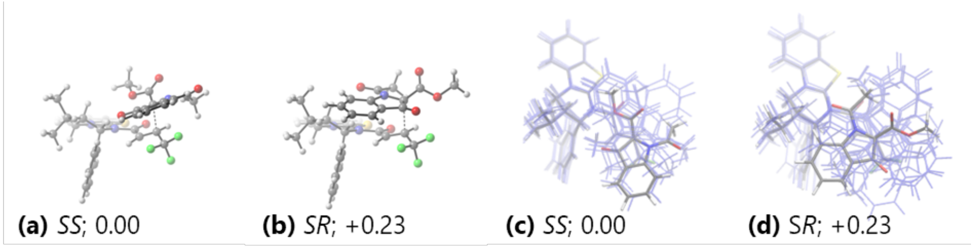

Figure S6 Transition states for the two diastereoisomers of substrate **19** arising from the top-face approach of the nucleophile (M06-2X$_{SMD}$/def2-TZVP//M06-2X$_{SMD}$/def2-SVP level). The most stable located transition state for each is highlighted **a**-**b**, with additional conformations shown as stick representations in **c**-**d**. Relative energies are shown as DDG$_{298}$ in kcal/mol.

Visualisation of non-covalent interactions (SMD//SMD)

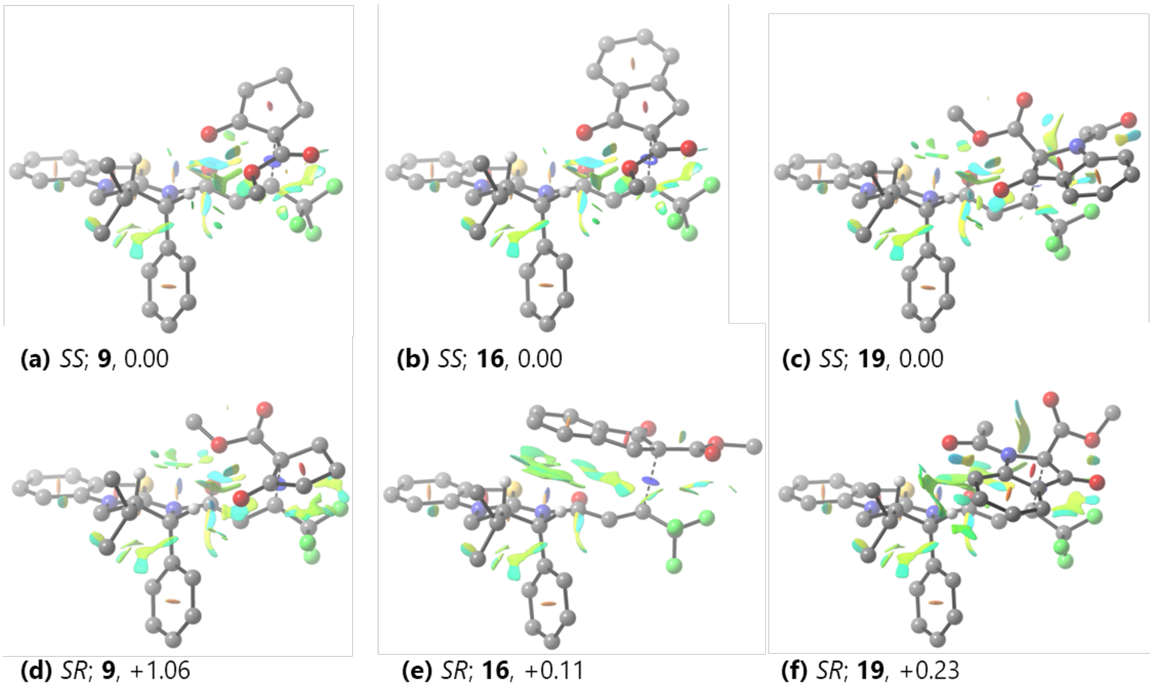

Figure S7 Non-covalent interactions for the most favourable diastereomeric transition states have been visualised, with areas of attractive interactions shown in green (M06-2X$_{SMD}$/def2-TZVP//M06-2X$_{SMD}$/def2-SVP level). **a-c)** major TS for each substrate; **d–f)** minor TS for each substrate. Visualisation has been performed using NCIPlot 4.0.[74] Relative energies are shown as DDG$_{298}$ in kcal/mol.

Comparison of solvent models

The PCM solvent model was found to incorrectly identify the major diastereomer of **16**. These are strongly zwitterionic TSs with very strong dipole moments (m_{SS} = 19.84 D; m_{SR} = 29.54 D). SMD performs better than PCM which appears to overstabilise the solvent contribution for the minor diastereoisomer relative to the major diastereomer (Table **S2**).

Table S2 Difference in Gibbs Free Energy between the two diasteromeric transition states with different solvent models in single-point calculations (corresponding to the top-face approach). Energies are shown in kcal/mol.

**Method**	**|DD*G*₂₉₈|**	**dr**
PCM//PCM	0.40 (*SR*)	66:34 (***SR***:*SS*)
none//PCM	10.97 (*SS*)	<1:99 (*SR*:***SS***)
SMD//PCM	0.55 (*SS*)	28:72 (*SR*:***SS***)
SMD//SMD	0.11 (*SS*)	45:55 (*SR*:***SS***)
none/SMD	10.97 (*SS*)	<1:99 (*SR*:***SS***)
PCM//SMD	0.76 (*SR*)	78:22 (***SR***:*SS*)

Between the two diastereoisomers, there is a significant difference of 10 D between the dipole moments, strongly impacting the magnitude of solvent correction for each structure. This "solvent correction" can be simply calculated as the difference between solvated single-point energy and gas phase single-point energy ($dE_{solv} = E_{solv} - E_{gas}$).

Table S3 Solvent correction obtained from different solvent models. Energies are shown in kcal/mol.

**Method**	**dE_{solv} *SS***	**dE_{solv} *SR***	**DdE_{solv}**
PCM//PCM	−20.28	−31.65	11.37
SMD//PCM	−33.39	−43.80	10.42
SMD//SMD	−33.86	−44.17	10.31
PCM//SMD	−20.51	−31.69	11.18

SMD has a larger solvent correction (Table **S3**) arising from stronger interactions with a smaller solvent shell and from parameterised dispersive interactions between the solute and the 1st solvent shell. Importantly there is a smaller difference between solvent contribution of the two diastereoisomers (DdE_{solv}) slightly reducing the relative stability of the observed minor TS.

One influencing factor may be in the construction of the cavity, which slightly varies between the two methods. PCM uses scaled vdW radii of 1.10 and SMD uses unscaled vdW radii ($\alpha = 1.00$). The scaling of the PCM radii has been explored (Table **S4**). Note the larger change in solvation energy for the *SS* structure between the unscaled PCM$^{\alpha=1.00}$//PCM (−26.52) and SMD//PCM (−33.39) compared to the *SR* structure, PCM$^{\alpha=1.00}$//PCM (−39.41) and SMD//PCM (−43.80) which indicates stronger additional stabilisation for *SS* with the same radii.

Table S4 Comparison of energetics using PCM with varied vdW scaling (α). Energies are shown in kcal/mol.

**Method**	**dE_{solv} *SS***	**dE_{solv} *SR***	**DdE_{solv}**	**|DDG_{298}|**	**dr**
PCM$^{\alpha=1.00}$//PCM	−26.52	−39.41	12.89	1.92 (*SR*)	96:4 (***SR***:*SS*)
PCM$^{\alpha=1.05}$//PCM	−23.08	−35.23	12.15	1.18 (*SR*)	88:12 (***SR***:*SS*)
PCM$^{\alpha=1.10}$//PCM	−20.28	−31.65	11.37	0.40 (*SR*)	66:34 (***SR***:*SS*)

PCM$^{\alpha=1.15}$//PCM	−17.90	−28.52	10.62	0.35 (*SS*)	36:64 (*SR*:***SS***)
PCM$^{\alpha=1.20}$//PCM	−15.93	−25.85	9.92	1.05 (*SS*)	14:86 (*SR*:***SS***)

Clearly the solvation energy is strongly influenced by the cavity size and is in a fine balance to lead to the correct diastereoisomer.

SMD builds on the IEF-PCM model and accounts for both bulk electrostatic contributions and an additional short-range interaction between the solute and solvent molecules in the first solvation shell.[69] This model is designed to improve the description of solvent interacting in ways other than by pure electrostatics. Elsewhere, SMD has been found to offer an improvement over PCM in highly polar solvents in the context of solvatochromism.[76]

Similarly, the inclusion of a single explicit solvent molecule placed below the isothiouronium catalyst (PCM//PCM) reduced DDG_{298} from 0.40 (*SR*) to 0.24 (*SR*), perhaps indicating an improvement in solvent description. Extending this to a second solvent molecule, placed above the isothiouronium catalyst led to DDG_{298} = 1.05 (*SS*) in agreement with the observed major diastereomer (PCM//PCM). This form of QM described, explicit solvation allows for additional non-covalent interactions between the solute and solvent alongside the electrostatic stabilisation from implicit solvation.

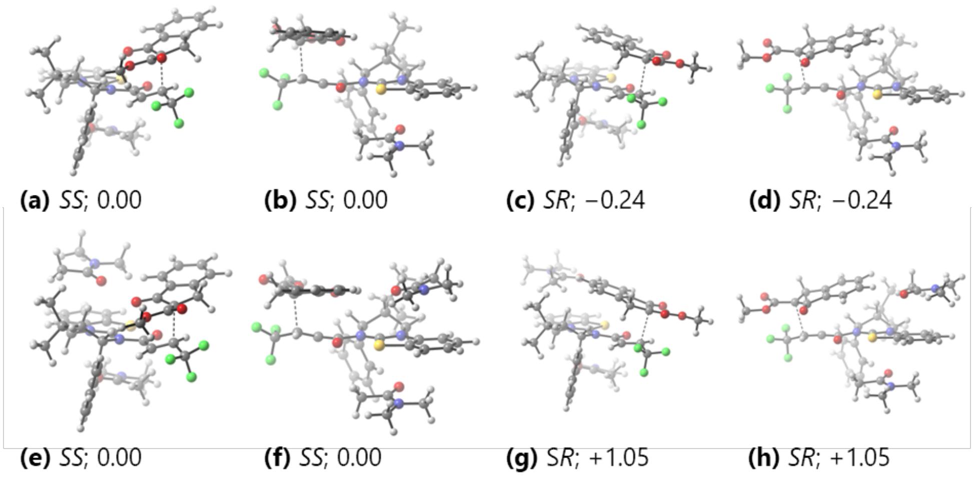

Figure S8 Explicit solvation of diastereomeric transition states for substrate **16** (M06-2X$_{PCM}$/def2-TZVP//M06-2X$_{PCM}$/def2-SVP level). **a,b)** alternate views of mono-solvated *SS* TS; **c,d)** alternate views of mono-solvated *SR* TS; **e,f)** alternate views of bi-solvated *SS* TS; **g,h)** alternate views of bi-solvated *SR* TS. Energies are shown as DDG_{298} in kcal/mol.

If the micro-solvation has improved the description of solvation, the difference between PCM and SMD should be reduced. SMD single-points on the mono-solvated transition state (SMD//PCM), calculated DD*G* = 0.89, 82:18 dr (*SS*) – different from the PCM//PCM single-point which still favoured *SR*. However, with the bi-solvated transition states (SMD//PCM), calculated DD*G* = 0.43, 67:33 dr (*SS*) – same as the PCM//PCM single-point. The inclusion of these solvent molecules both improves the description of the solute to the 1st coordination sphere and reduces the difference between the two solvent models.

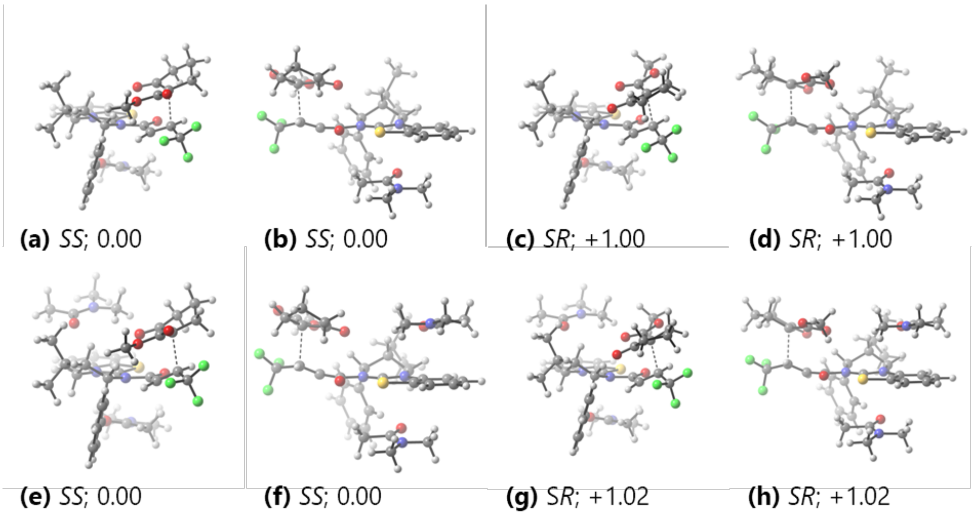

Figure S9 Explicit solvation of diastereomeric transition states for substrate **9** (M06-2X$_{PCM}$/def2-TZVP//M06-2X$_{PCM}$/def2-SVP level). **a,b)** alternate views of mono-solvated *SS* TS; **c**,**d)** alternate views of mono-solvated *SR* TS; **e**,**f)** alternate views of bi-solvated *SS* TS; **g**,**h)** alternate views of bi-solvated *SR* TS. Energies are shown as DDG_{298} kcal/mol.

To ensure this was not artificial, we found that explicit solvation made no change to the predicted major diastereomer of substrate **9** unlike substrate **16**. The DD*G* was 1.04 kcal/mol (*SS*) with no explicit solvent (PCM//PCM) which is in line with the computed DD*G* = 1.02 (*SS*) with explicit solvation in Figure **S9** (PCM//PCM). SMD single-points of these led to a DD*G* of 1.84 and 1.53 for the mono- and bi-solvated structures respectively (SMD//PCM). This leads to the same diastereomer prediction, validating that the SMD computation or explicit solvation does not change the predicted diastereomer for the cases where the PCM calculation agreed with experiment.

Table S5 Selectivity of the different substrates. Diastereomeric ratios have been calculated from DDG_{298} between the most stable conformation of each diastereomer.

Substrate	dr	er_{major}	er_{minor}
**9_{exp}**	>95:5 (*SS*)	99:1 (*SS*:*RR*)	
**$9_{PCM//PCM}$**	85:15 (*SS*:*SR*)	89:11 (*SS*:*RR*)	90:10 (*SR*:*RS*)
**$9_{SMD//PCM}$**	96:4 (*SS*:*SR*)		
**$9_{SMD//SMD}$**	86:14 (*SS*:*SR*)		
**16_{exp}**	72:28 (*SS*)	94:6 (*SS*:*RR*)	98:2 (*SR*:*RS*)
**$16_{PCM//PCM}$**	66:34 (*SR*:*SS*)	96:4 (*SR*:*RS*)	91:9 (*SS*:*RR*)
**$16_{SMD//PCM}$**	72:28 (*SS*:*SR*)		
**$16_{SMD//SMD}$**	55:45 (*SS*:*SR*)		
**19_{exp}**	>95:5 (*SS*)	98:2 (*SS*:*RR*)	
**$19_{PCM//PCM}$**	68:32 (*SS*:*SR*)	88:12 (*SS*:*RR*)	91:9 (*SR*:*RS*)
**$19_{SMD//PCM}$**	87:13 (*SS*:*SR*)		
**$19_{SMD//SMD}$**	60:40 (*SS*:*SR*)		

Computational Data

Raw data and cartesian coordinates obtained from geometry optimisation and frequency calculations and subsequent single-point energy calculations.

Dispersion corrections using Grimme's D3 correction were evaluated at single-point; however, these have been subtracted from the computed single-point energies to avoid over estimation since the Minnesota functionals already account for dispersive interactions.

PCMopt/16_TS_RRa

Frequencies, energies and thermodynamic properties:

Lowest Vibrational Mode (1/cm) = -134.8159

2nd Lowest Vibrational Mode (1/cm) = 22.9392

E(RM062X) (a.u.) = -2420.00511470

Thermal correction to Enthalpy (a.u.) = 0.632933

Thermal correction to Gibbs Free Energy (a.u.) = 0.522632

Total Entropy (cal/Kmol) = 232.148

Esp(RM062X+D3) (a.u.) = -2422.42363995

Esp(D3) (a.u.) = -0.0093925601

Esp(RM062X) (a.u.) = -2422.4142473899

Optimised cartesian coordinates (Angstrom):

C	7.198590	-0.973448	-1.068923
C	7.054820	-2.349886	-0.834155
C	5.867711	-2.869355	-0.331958
C	4.829851	-1.976178	-0.061422
C	4.978147	-0.607238	-0.288682
H	7.883826	-3.023030	-1.052657
H	5.748294	-3.938067	-0.154037
C	2.815190	-0.663735	0.486578
C	1.413212	1.332731	0.912547
C	2.776389	2.031772	1.057752
C	3.721321	1.557240	-0.029535
H	4.727295	1.963749	0.127235
S	3.231500	-2.351207	0.541223
N	3.817961	0.098960	0.049028
N	1.615133	-0.142533	0.850178
C	2.684124	3.573684	1.137183
C	2.748190	4.279238	-0.221240
C	3.782307	4.110799	2.057279
H	1.715266	3.812881	1.604083
H	3.754080	4.192919	-0.660792
H	2.020329	3.887371	-0.944853
H	2.544931	5.350715	-0.086635
H	3.690083	3.701579	3.072989
H	4.781183	3.850823	1.672827
H	3.726740	5.206347	2.121331
H	3.188063	1.676547	2.016441
H	0.888153	1.525181	1.855721
C	0.564361	1.822015	-0.240751
C	-0.358804	2.849650	-0.017073
C	0.720322	1.309879	-1.531654

C	-1.082609	3.389313	-1.078336
H	-0.512452	3.234563	0.994217
C	-0.003072	1.850891	-2.593992
H	1.399746	0.474512	-1.719095
C	-0.897935	2.896905	-2.371288
H	-1.795846	4.193888	-0.893717
H	0.126440	1.443589	-3.597623
H	-1.466201	3.316567	-3.202701
C	-0.750180	-0.434381	1.537150
H	-0.871753	0.637696	1.672337
C	-1.799469	-1.247452	1.718785
C	-3.061871	-0.694902	2.317907
F	-2.915181	-0.563779	3.646004
F	-3.355138	0.517774	1.845041
F	-4.113946	-1.481818	2.128164
O	0.739900	-2.221746	1.126324
C	0.543352	-1.028967	1.157868
H	3.380410	1.838989	-1.036930
C	6.166176	-0.082185	-0.803092
H	6.283847	0.982164	-1.001772
H	8.136762	-0.591934	-1.472000
H	-1.733966	-2.330289	1.591900
C	0.365449	-2.315743	-2.006396
H	0.576202	-2.014893	-3.042856
H	1.252478	-2.113816	-1.390345
H	0.150833	-3.389901	-1.962531
C	-1.851216	-2.248495	-1.100047
O	-0.729739	-1.566140	-1.528266
O	-1.814246	-3.457629	-0.926255
C	-4.293183	-1.747889	-0.643473
C	-2.941007	-1.350370	-0.905542
O	-4.797717	-2.853808	-0.426353
C	-4.263588	0.603196	-1.022452
C	-4.797489	1.886290	-1.120169
C	-5.093331	-0.473756	-0.697049
C	-6.165437	2.067543	-0.887796
H	-4.161992	2.738480	-1.372884

C -6.455360 -0.302938 -0.466116
C -6.990607 0.983299 -0.562665
H -6.596954 3.067740 -0.960545
H -7.076925 -1.165190 -0.214458
H -8.054695 1.150187 -0.385134
C -2.846359 0.122560 -1.211990
H -2.141348 0.665513 -0.552787
H -2.493645 0.323910 -2.238721

PCMopt/16_TS_RRb

Frequencies, energies and thermodynamic properties:

Lowest Vibrational Mode (1/cm) = -177.0764

2nd Lowest Vibrational Mode (1/cm) = 14.1665

E(RM062X) (a.u.) = -2420.01022521

Thermal correction to Enthalpy (a.u.) = 0.632879

Thermal correction to Gibbs Free Energy (a.u.) = 0.523964

Total Entropy (cal/Kmol) = 229.230

Esp(RM062X+D3) (a.u.) = -2422.42417039

Esp(D3) (a.u.) = -0.0093167934

Esp(RM062X) (a.u.) = -2422.4148535966

Optimised cartesian coordinates (Angstrom):

C 6.677099 -1.419908 -1.610143
C 6.379164 -2.775728 -1.410642
C 5.214096 -3.162637 -0.756186
C 4.354535 -2.161251 -0.304712
C 4.659250 -0.812987 -0.497108
H 7.066986 -3.538558 -1.775686
H 4.976119 -4.215399 -0.603019
C 2.633532 -0.627127 0.578528
C 1.475025 1.492234 0.995672
C 2.900546 2.066852 1.091145
C 3.767815 1.480387 -0.007333
H 4.821377 1.745533 0.149189
S 2.832464 -2.355781 0.541456
N 3.670111 0.021376 0.034443
N 1.554136 0.013277 1.058391
C 2.951747 3.612312 1.131390

C	3.059223	4.279938	-0.243395
C	4.104947	4.071461	2.026216
H	2.013368	3.948175	1.602135
H	4.048287	4.098564	-0.692237
H	2.291045	3.934842	-0.949179
H	2.949156	5.367627	-0.131413
H	3.989897	3.695773	3.052563
H	5.071187	3.713197	1.637390
H	4.148881	5.168876	2.064614
H	3.297455	1.697777	2.050879
H	0.961001	1.803061	1.914002
C	0.683961	1.975067	-0.202549
C	-0.158062	3.083416	-0.058186
C	0.816322	1.374941	-1.457709
C	-0.830883	3.608307	-1.160074
H	-0.290188	3.541245	0.925427
C	0.152724	1.907762	-2.562671
H	1.411174	0.467607	-1.578298
C	-0.666771	3.027059	-2.418528
H	-1.485742	4.471805	-1.034993
H	0.264194	1.433169	-3.538613
H	-1.190502	3.437076	-3.283392
C	-0.826554	-0.079002	1.672800
H	-0.908838	1.001320	1.589603
C	-1.962054	-0.830467	1.752955
C	-3.223177	-0.157177	2.211641
F	-3.151051	0.098462	3.529150
F	-3.421464	1.019104	1.609382
F	-4.316659	-0.896031	2.037749
O	0.630094	-1.937455	1.792603
C	0.439782	-0.763293	1.537839
H	3.464295	1.825275	-1.007053
C	5.823627	-0.418689	-1.158591
H	6.056380	0.632163	-1.326066
H	7.592810	-1.140855	-2.131645
H	-1.918783	-1.909640	1.914042
C	-0.342475	-4.116227	-0.384774

H 0.137975 -4.025695 -1.368905
H 0.375941 -3.815168 0.388589
H -0.666200 -5.149585 -0.219378
C -1.370193 -2.019378 -0.658344
O -1.508800 -3.316210 -0.311906
O -0.281107 -1.555780 -0.975647
C -3.932241 -1.755691 -0.514007
C -2.572901 -1.237683 -0.556018
O -4.357853 -2.866665 -0.217526
C -4.018764 0.493987 -1.275582
C -4.626576 1.682521 -1.675463
C -4.805910 -0.604212 -0.920741
C -6.023031 1.742404 -1.717648
H -4.025869 2.552574 -1.950747
C -6.196873 -0.554682 -0.961324
C -6.805447 0.634366 -1.364965
H -6.513196 2.666657 -2.029450
H -6.782500 -1.431827 -0.677891
H -7.893530 0.708197 -1.405787
C -2.555976 0.151262 -1.143616
H -2.005484 0.884239 -0.525086
H -2.055011 0.168145 -2.126729

PCMopt/16_TS_RRc

Frequencies, energies and thermodynamic properties:

Lowest Vibrational Mode (1/cm) = -147.6723

2nd Lowest Vibrational Mode (1/cm) = 24.7359

E(RM062X) (a.u.) = -2420.01236128

Thermal correction to Enthalpy (a.u.) = 0.633088

Thermal correction to Gibbs Free Energy (a.u.) = 0.525933

Total Entropy (cal/Kmol) = 225.526

Esp(RM062X+D3) (a.u.) = -2422.42560894

Esp(D3) (a.u.) = -0.0093168478

Esp(RM062X) (a.u.) = -2422.4162920922

Optimised cartesian coordinates (Angstrom):

C 5.953131 2.833875 0.846464
C 5.254555 4.033394 0.646423

C	3.947624	4.028193	0.169337
C	3.356499	2.794775	-0.103578
C	4.058265	1.603793	0.088339
H	5.739385	4.983629	0.870671
H	3.398948	4.957641	0.015324
C	2.038859	0.777571	-0.642931
C	1.497119	-1.606047	-0.823524
C	2.998479	-1.750771	-1.142132
C	3.808392	-0.876560	-0.201713
H	4.858681	-0.838010	-0.518422
S	1.739833	2.492132	-0.710782
N	3.285031	0.488307	-0.250322
N	1.132026	-0.173302	-0.921402
C	3.501178	-3.213611	-1.173686
C	4.028109	-3.732553	0.168125
C	4.576648	-3.369905	-2.250907
H	2.643454	-3.838661	-1.469935
H	4.977631	-3.241773	0.433141
H	3.319769	-3.582953	0.994626
H	4.231077	-4.809789	0.089941
H	4.188085	-3.110302	-3.245581
H	5.440500	-2.719664	-2.040958
H	4.941036	-4.406205	-2.283620
H	3.119992	-1.337790	-2.156846
H	0.959324	-2.107576	-1.638139
C	1.088513	-2.225221	0.496109
C	0.647953	-3.553607	0.511768
C	1.219559	-1.534099	1.703583
C	0.398847	-4.201595	1.720102
H	0.510156	-4.091026	-0.429772
C	0.961527	-2.179512	2.912784
H	1.491683	-0.476807	1.708720
C	0.566662	-3.517471	2.925107
H	0.065288	-5.240288	1.719757
H	1.066696	-1.631583	3.850059
H	0.370556	-4.021128	3.872784
C	-1.229962	-0.790058	-1.291777

H	-1.036702	-1.813609	-0.980633
C	-2.502930	-0.371907	-1.523112
C	-3.569664	-1.375754	-1.840306
F	-3.488722	-1.736781	-3.131943
F	-3.450757	-2.499024	-1.125693
F	-4.804465	-0.908313	-1.661777
O	-0.376679	1.387334	-1.610792
C	-0.199261	0.226541	-1.294137
H	3.768679	-1.234618	0.837722
C	5.367891	1.602205	0.571583
H	5.913049	0.674046	0.738887
H	6.974013	2.862520	1.227728
H	-2.693079	0.635145	-1.900316
C	-3.152208	-2.954173	1.959494
H	-3.691095	-3.500733	1.172979
H	-2.223667	-3.475432	2.214021
H	-3.802244	-2.885021	2.842955
C	-3.791391	-0.863539	1.135303
O	-2.774072	-1.668677	1.503159
O	-4.950945	-1.228724	1.232652
C	-2.116118	1.067715	0.894173
C	-3.377514	0.427866	0.626010
O	-1.024953	0.569042	1.189650
C	-3.669866	2.757527	0.301097
C	-4.104290	4.052879	0.021220
C	-2.338457	2.530584	0.658980
C	-3.192266	5.106218	0.128415
H	-5.139409	4.246352	-0.268450
C	-1.425651	3.576679	0.778650
C	-1.863195	4.872896	0.509724
H	-3.519635	6.126216	-0.081160
H	-0.395509	3.367904	1.072720
H	-1.171876	5.713376	0.593903
C	-4.434650	1.456824	0.299623
H	-4.926470	1.277380	-0.670880
H	-5.240675	1.465950	1.053061

PCMopt/16_TS_RRd

Frequencies, energies and thermodynamic properties:

Lowest Vibrational Mode (1/cm) = -154.3214

2nd Lowest Vibrational Mode (1/cm) = 24.8761

E(RM062X) (a.u.) = -2420.01197583

Thermal correction to Enthalpy (a.u.) = 0.633104

Thermal correction to Gibbs Free Energy (a.u.) = 0.526231

Total Entropy (cal/Kmol) = 224.933

Esp(RM062X+D3) (a.u.) = -2422.42447811

Esp(D3) (a.u.) = -0.0091050739

Esp(RM062X) (a.u.) = -2422.4153730361

Optimised cartesian coordinates (Angstrom):

C	6.114806	2.644814	0.829880
C	5.456824	3.863985	0.612218
C	4.150418	3.895564	0.134549
C	3.518305	2.678937	-0.121220
C	4.180115	1.468140	0.087534
H	5.973092	4.800639	0.823023
H	3.633194	4.840593	-0.033026
C	2.135352	0.698717	-0.635912
C	1.513913	-1.667090	-0.776196
C	3.009810	-1.871640	-1.084742
C	3.847730	-1.006545	-0.160699
H	4.899080	-1.009806	-0.476350
S	1.892011	2.421704	-0.724547
N	3.370860	0.374102	-0.236655
N	1.199179	-0.225350	-0.905367
C	3.457793	-3.352298	-1.082163
C	3.950570	-3.864010	0.275367
C	4.537231	-3.570713	-2.144526
H	2.579509	-3.950030	-1.375023
H	4.914836	-3.403765	0.541885
H	3.239048	-3.672099	1.090408
H	4.114106	-4.949367	0.220055
H	4.169635	-3.314152	-3.147946
H	5.424183	-2.951548	-1.936801
H	4.860447	-4.621035	-2.154837

H	3.149589	-1.485826	-2.107801
H	0.962873	-2.166561	-1.582909
C	1.074178	-2.240097	0.554048
C	0.570251	-3.544779	0.596256
C	1.232234	-1.529051	1.746487
C	0.274014	-4.149070	1.816710
H	0.413829	-4.096729	-0.334023
C	0.936580	-2.133167	2.967963
H	1.557656	-0.487101	1.730318
C	0.468739	-3.447016	3.006853
H	-0.113494	-5.168485	1.837286
H	1.064946	-1.570450	3.893535
H	0.238936	-3.917491	3.963871
C	-1.180144	-0.770258	-1.284216
H	-1.021596	-1.802925	-0.983972
C	-2.438862	-0.303706	-1.513038
C	-3.536114	-1.261913	-1.869133
F	-3.421123	-1.625618	-3.157747
F	-3.496519	-2.389395	-1.153645
F	-4.755457	-0.740746	-1.738458
O	-0.257184	1.377472	-1.607453
C	-0.118275	0.212344	-1.284559
H	3.794587	-1.341746	0.885828
C	5.488668	1.429649	0.572079
H	6.002186	0.486148	0.753104
H	7.136003	2.644703	1.211374
H	-2.586309	0.713210	-1.883270
C	-5.295960	-2.403813	1.503614
H	-5.029002	-2.574242	2.555942
H	-6.382500	-2.462047	1.379614
H	-4.804922	-3.171982	0.890200
C	-3.581242	-0.837962	1.150787
O	-4.910644	-1.109954	1.082303
O	-2.799639	-1.653569	1.597926
C	-2.010632	1.117498	0.896253
C	-3.273737	0.478257	0.631394
O	-0.927520	0.606515	1.196525

C -3.559787 2.807845 0.306750
C -3.992856 4.103578 0.025890
C -2.225320 2.578698 0.652174
C -3.075617 5.153774 0.115967
H -5.030629 4.299425 -0.252455
C -1.306243 3.621601 0.752164
C -1.742215 4.917600 0.480540
H -3.401694 6.173784 -0.095511
H -0.272500 3.410635 1.032341
H -1.046499 5.755794 0.549261
C -4.333109 1.510979 0.316353
H -5.128064 1.532400 1.080843
H -4.839697 1.336534 -0.646376

PCMopt/16_TS_RRe

Frequencies, energies and thermodynamic properties:

Lowest Vibrational Mode (1/cm) = -140.3745
2nd Lowest Vibrational Mode (1/cm) = 11.4565
E(RM062X) (a.u.) = -2420.00249821
Thermal correction to Enthalpy (a.u.) = 0.633203
Thermal correction to Gibbs Free Energy (a.u.) = 0.522342
Total Entropy (cal/Kmol) = 233.328
Esp(RM062X+D3) (a.u.) = -2422.41965777
Esp(D3) (a.u.) = -0.0092556162
Esp(RM062X) (a.u.) = -2422.4104021538

Optimised cartesian coordinates (Angstrom):

C -6.615158 -2.516625 0.465746
C -6.028770 -3.764969 0.723406
C -4.657280 -3.952870 0.590404
C -3.885621 -2.859837 0.195655
C -4.473669 -1.622265 -0.068858
H -6.655473 -4.600617 1.035033
H -4.196845 -4.920363 0.790628
C -2.266661 -1.118580 -0.462268
C -1.442629 1.134235 -0.965197
C -2.791866 1.296651 -1.689035
C -3.906064 0.687496 -0.858883

H	-4.827854	0.605496	-1.449423
S	-2.155279	-2.809331	-0.069316
N	-3.525016	-0.669745	-0.458920
N	-1.212358	-0.309710	-0.705378
C	-3.089256	2.751879	-2.122315
C	-3.826206	3.592979	-1.075222
C	-3.869683	2.753475	-3.438517
H	-2.112753	3.226780	-2.314138
H	-4.863408	3.245237	-0.948784
H	-3.335634	3.583149	-0.091868
H	-3.874836	4.636621	-1.415829
H	-3.307962	2.250605	-4.238051
H	-4.836426	2.238430	-3.323640
H	-4.078312	3.783278	-3.760258
H	-2.699847	0.691673	-2.605768
H	-0.676990	1.431474	-1.690406
C	-1.297894	1.980493	0.280942
C	-0.591476	3.185587	0.197674
C	-1.937089	1.646794	1.480289
C	-0.564642	4.066562	1.277968
H	-0.069188	3.444844	-0.727005
C	-1.921917	2.533717	2.555640
H	-2.453433	0.690069	1.591754
C	-1.245333	3.749052	2.453384
H	-0.014110	5.004714	1.198021
H	-2.434579	2.267955	3.481276
H	-1.234431	4.441183	3.296539
C	1.208254	0.068898	-0.880922
H	1.055592	1.110167	-0.601991
C	2.423801	-0.325739	-1.334990
C	3.362116	0.738755	-1.829878
F	2.996330	1.108364	-3.071849
F	3.319486	1.840080	-1.080336
F	4.625271	0.339677	-1.918819
O	0.199666	-2.085105	-0.839117
C	0.102362	-0.876488	-0.798669
H	-4.123188	1.272030	0.047028

C	-5.849492	-1.427093	0.065979
H	-6.309569	-0.458333	-0.124831
H	-7.692005	-2.393837	0.582083
H	2.611131	-1.341363	-1.693333
C	6.901211	-1.959300	-1.360404
H	6.451465	-1.884472	-2.361424
H	7.848138	-1.409393	-1.336481
H	7.078844	-3.020876	-1.139232
C	4.825659	-1.886145	-0.280358
O	6.064935	-1.363565	-0.389377
O	4.520972	-2.905477	-0.888262
C	4.140796	0.130317	1.195309
C	3.927203	-1.150637	0.570439
O	5.057719	0.947953	1.096223
C	2.090354	-0.773140	1.998359
C	0.881545	-0.775201	2.695668
C	2.913414	0.356469	2.028764
C	0.523367	0.364800	3.418787
H	0.225962	-1.649545	2.676889
C	2.555839	1.501054	2.739572
C	1.350261	1.497390	3.440253
H	-0.419450	0.380589	3.968463
H	3.216576	2.370780	2.737884
H	1.038656	2.378894	4.003637
C	2.729672	-1.852379	1.164982
H	2.038579	-2.268419	0.420432
H	3.030437	-2.696830	1.813012

PCMopt/16_TS_RRf

Frequencies, energies and thermodynamic properties:

Lowest Vibrational Mode (1/cm) = -145.0859

2nd Lowest Vibrational Mode (1/cm) = 15.6463

E(RM062X) (a.u.) = -2420.00176969

Thermal correction to Enthalpy (a.u.) = 0.633197

Thermal correction to Gibbs Free Energy (a.u.) = 0.522622

Total Entropy (cal/Kmol) = 232.725

Esp(RM062X+D3) (a.u.) = -2422.41913829

Esp(D3) (a.u.) = -0.0093082442

Esp(RM062X) (a.u.) = -2422.4098300458

Optimised cartesian coordinates (Angstrom):

C	6.485593	2.632676	0.577297
C	5.862241	3.856420	0.864140
C	4.487256	4.010136	0.722885
C	3.749906	2.907834	0.290429
C	4.374672	1.694811	-0.002478
H	6.462513	4.699820	1.205494
H	3.998086	4.958264	0.946140
C	2.186124	1.140430	-0.428523
C	1.429444	-1.122470	-0.987961
C	2.785735	-1.229140	-1.708991
C	3.878617	-0.607891	-0.859425
H	4.799627	-0.484081	-1.443959
S	2.024104	2.816995	0.006058
N	3.456577	0.727264	-0.427143
N	1.156869	0.308340	-0.696516
C	3.126754	-2.664953	-2.174161
C	3.881763	-3.509350	-1.142725
C	3.914338	-2.614032	-3.485090
H	2.165129	-3.162642	-2.382896
H	4.907309	-3.134120	-1.000823
H	3.385092	-3.538334	-0.162842
H	3.963629	-4.542352	-1.508474
H	3.344854	-2.105519	-4.275513
H	4.867327	-2.078363	-3.351143
H	4.150519	-3.629986	-3.830926
H	2.680061	-0.606129	-2.612055
H	0.674709	-1.423914	-1.722981
C	1.304637	-2.003445	0.236357
C	0.629044	-3.223515	0.120287
C	1.934203	-1.685616	1.445008
C	0.624092	-4.133345	1.176684
H	0.113310	-3.470645	-0.811402
C	1.941130	-2.600945	2.496301
H	2.425063	-0.719057	1.583231

C	1.296297	-3.830193	2.360716
H	0.097559	-5.082694	1.071250
H	2.445753	-2.346655	3.429571
H	1.302687	-4.544632	3.185106
C	-1.247964	-0.138574	-0.881130
H	-1.063076	-1.170720	-0.587935
C	-2.472743	0.208100	-1.349410
C	-3.366181	-0.894954	-1.843609
F	-2.997488	-1.231602	-3.094882
F	-3.254854	-2.001723	-1.107828
F	-4.646758	-0.561449	-1.909935
O	-0.304051	2.045648	-0.825977
C	-0.172906	0.840217	-0.792756
H	4.109806	-1.208193	0.032597
C	5.754120	1.534118	0.139798
H	6.242563	0.584170	-0.073768
H	7.564342	2.536443	0.700757
H	-2.687338	1.211246	-1.725003
C	-5.511309	3.590746	-1.427092
H	-6.396536	3.834780	-0.823027
H	-4.999413	4.511310	-1.727389
H	-5.845200	3.040768	-2.318314
C	-5.035286	1.639611	-0.191710
O	-4.578877	2.833277	-0.681767
O	-6.171832	1.276646	-0.426757
C	-4.159955	-0.334855	1.162890
C	-4.009111	0.965545	0.563543
O	-5.048304	-1.181948	1.050982
C	-2.149627	0.639163	1.975742
C	-0.938205	0.681581	2.667808
C	-2.922043	-0.525818	1.987788
C	-0.528257	-0.453512	3.370416
H	-0.321023	1.583679	2.661356
C	-2.512250	-1.665483	2.678368
C	-1.305959	-1.620711	3.376184
H	0.416837	-0.437994	3.916327
H	-3.133923	-2.563500	2.664935

H -0.954577 -2.497029 3.923953
C -2.838269 1.707197 1.166759
H -2.165621 2.164725 0.429470
H -3.168855 2.525493 1.832858

PCMopt/16_TS_RSa
Frequencies, energies and thermodynamic properties:
Lowest Vibrational Mode (1/cm) = -100.3245
2nd Lowest Vibrational Mode (1/cm) = 11.5728
E(RM062X) (a.u.) = -2420.00645894
Thermal correction to Enthalpy (a.u.) = 0.632965
Thermal correction to Gibbs Free Energy (a.u.) = 0.522515
Total Entropy (cal/Kmol) = 232.462
Esp(RM062X+D3) (a.u.) = -2422.42341849
Esp(D3) (a.u.) = -0.0094799054
Esp(RM062X) (a.u.) = -2422.4139385846
Optimised cartesian coordinates (Angstrom):
C -6.531233 -2.188567 -0.129982
C -5.983560 -3.464042 -0.336507
C -4.627198 -3.627232 -0.595023
C -3.830161 -2.482696 -0.641868
C -4.380346 -1.215541 -0.444409
H -6.629064 -4.341216 -0.291164
H -4.196502 -4.615847 -0.753992
C -2.172715 -0.661963 -0.759486
C -1.289071 1.621264 -0.586568
C -2.669493 2.029173 -1.130669
C -3.757976 1.211632 -0.461087
H -4.715373 1.343193 -0.981644
S -2.109894 -2.388215 -0.951407
N -3.412059 -0.210151 -0.542071
N -1.101626 0.162631 -0.795469
C -2.930058 3.552944 -1.066135
C -3.568464 4.037139 0.239329
C -3.784281 3.986633 -2.259224
H -1.947387 4.043237 -1.166059
H -4.607916 3.683810 0.325185

H	-3.017921	3.712668	1.133235
H	-3.599143	5.135582	0.243756
H	-3.293370	3.745622	-3.212437
H	-4.765585	3.486999	-2.243597
H	-3.963414	5.070394	-2.229287
H	-2.655045	1.742275	-2.194750
H	-0.559533	2.112381	-1.239576
C	-1.028246	2.033084	0.845908
C	-0.249352	3.168124	1.095320
C	-1.614051	1.358716	1.922648
C	-0.084935	3.642112	2.396436
H	0.224986	3.695444	0.263440
C	-1.460388	1.838365	3.222400
H	-2.186488	0.442329	1.761646
C	-0.700780	2.984127	3.461475
H	0.523372	4.529171	2.576873
H	-1.928373	1.307980	4.053187
H	-0.579954	3.357445	4.479208
C	1.334232	0.523646	-1.010598
H	1.245539	1.512842	-0.566270
C	2.520576	0.107482	-1.507522
C	3.592031	1.136669	-1.741441
F	3.243943	1.922966	-2.774009
F	3.766579	1.944851	-0.695452
F	4.770062	0.606156	-2.059355
O	0.259186	-1.531554	-1.466054
C	0.186683	-0.378212	-1.106305
H	-3.897095	1.481100	0.595881
C	-5.741014	-1.045904	-0.180473
H	-6.170880	-0.059580	-0.009970
H	-7.596716	-2.087918	0.076961
H	2.628923	-0.849697	-2.021100
C	7.089224	0.435996	0.164929
H	7.141633	0.566785	-0.925505
H	7.387295	1.363428	0.666369
H	7.772492	-0.377205	0.448369
C	5.201092	-0.961168	0.040508

O	5.765839	0.162412	0.572917
O	5.854884	-1.694059	-0.680833
C	3.046734	-2.283097	0.177286
C	3.829126	-1.104031	0.446552
O	3.231246	-3.210748	-0.614985
C	1.918483	-1.010196	1.832317
C	0.886044	-0.689131	2.713678
C	1.849646	-2.181373	1.075129
C	-0.195039	-1.566129	2.830398
H	0.915925	0.232664	3.299616
C	0.775913	-3.062104	1.187511
C	-0.250473	-2.747888	2.076639
H	-1.009622	-1.329511	3.518535
H	0.745089	-3.963361	0.571301
H	-1.108279	-3.414911	2.183420
C	3.197183	-0.268978	1.533856
H	2.984994	0.770148	1.227687
H	3.828238	-0.199464	2.436829

PCMopt/16_TS_RSb

Frequencies, energies and thermodynamic properties:

Lowest Vibrational Mode (1/cm) =	-119.9487
2nd Lowest Vibrational Mode (1/cm) =	18.2077
E(RM062X) (a.u.) =	-2420.00581683
Thermal correction to Enthalpy (a.u.) =	0.632871
Thermal correction to Gibbs Free Energy (a.u.) =	0.523181
Total Entropy (cal/Kmol) =	230.860
Esp(RM062X+D3) (a.u.) =	-2422.42269554
Esp(D3) (a.u.) =	-0.0097405822
Esp(RM062X) (a.u.) =	-2422.4129549578

Optimised cartesian coordinates (Angstrom):

C	-6.020622	-2.320021	0.317008
C	-5.412603	-3.524564	-0.068649
C	-4.117063	-3.542005	-0.573922
C	-3.443543	-2.324889	-0.683853
C	-4.052821	-1.128606	-0.300556
H	-5.961613	-4.461005	0.030976

H	-3.638158	-4.474720	-0.873239
C	-1.986154	-0.361472	-0.938860
C	-1.235082	1.962191	-0.682664
C	-2.711615	2.301842	-0.954211
C	-3.614918	1.341112	-0.200984
H	-4.654881	1.444818	-0.536837
S	-1.819662	-2.053919	-1.275148
N	-3.200456	-0.034770	-0.483222
N	-0.994669	0.547203	-1.058896
C	-3.060078	3.786402	-0.691923
C	-3.508598	4.092508	0.740520
C	-4.125269	4.255673	-1.685029
H	-2.142701	4.364392	-0.892221
H	-4.499031	3.656980	0.945214
H	-2.805099	3.722973	1.499419
H	-3.600685	5.179936	0.869122
H	-3.780202	4.149110	-2.722942
H	-5.053341	3.673632	-1.571344
H	-4.372914	5.312211	-1.511851
H	-2.858882	2.113020	-2.030071
H	-0.648122	2.563313	-1.388177
C	-0.767059	2.256574	0.727415
C	-0.063722	3.437922	0.983524
C	-1.059991	1.393084	1.787637
C	0.323121	3.762548	2.283030
H	0.186352	4.110742	0.159120
C	-0.675436	1.717789	3.088051
H	-1.578326	0.448432	1.606906
C	0.013091	2.905018	3.338898
H	0.872610	4.685968	2.469584
H	-0.912813	1.039856	3.909558
H	0.315462	3.156987	4.356066
C	1.422804	1.003354	-1.257346
H	1.297020	1.988889	-0.816824
C	2.665648	0.519258	-1.496288
C	3.807923	1.492836	-1.430211
F	3.808418	2.276928	-2.520448

F	3.716450	2.304631	-0.376267
F	5.000766	0.902314	-1.394371
O	0.417252	-1.016009	-1.923092
C	0.302134	0.102857	-1.466846
H	-3.583017	1.502179	0.886545
C	-5.352839	-1.105166	0.208473
H	-5.826921	-0.175234	0.520293
H	-7.035600	-2.333349	0.714464
H	2.845028	-0.423868	-2.015311
C	6.744818	-0.909156	0.334130
H	7.030984	-1.025295	1.389140
H	7.372094	-1.550960	-0.294221
H	6.883780	0.143533	0.051044
C	4.467217	-0.618397	0.794781
O	5.409546	-1.315373	0.116845
O	4.788650	0.255131	1.587182
C	2.735422	-2.246760	-0.172858
C	3.114236	-1.032610	0.522638
O	3.300866	-2.863268	-1.074427
C	1.011940	-1.730978	1.378758
C	-0.153370	-1.958064	2.111976
C	1.430303	-2.675896	0.438279
C	-0.865242	-3.145670	1.905407
H	-0.485228	-1.246108	2.870272
C	0.724955	-3.856274	0.219576
C	-0.428053	-4.092365	0.971042
H	-1.766126	-3.342082	2.490160
H	1.086129	-4.578212	-0.516087
H	-0.995047	-5.015167	0.832012
C	2.022125	-0.606527	1.464822
H	1.554176	0.356748	1.168098
H	2.384379	-0.460040	2.495435

PCMopt/16_TS_RSc

Frequencies, energies and thermodynamic properties:

Lowest Vibrational Mode (1/cm) = -137.3030

2nd Lowest Vibrational Mode (1/cm) = 16.4839

E(RM062X) (a.u.) = -2420.00890798

Thermal correction to Enthalpy (a.u.) = 0.633346

Thermal correction to Gibbs Free Energy (a.u.) = 0.525263

Total Entropy (cal/Kmol) = 227.482

Esp(RM062X+D3) (a.u.) = -2422.42303914

Esp(D3) (a.u.) = -0.0087687558

Esp(RM062X) (a.u.) = -2422.4142703842

Optimised cartesian coordinates (Angstrom):

C	6.846428	-1.963745	-1.039296
C	6.401518	-3.280099	-0.847177
C	5.127098	-3.539138	-0.353707
C	4.308570	-2.449391	-0.056430
C	4.758485	-1.141344	-0.239171
H	7.061115	-4.113067	-1.090789
H	4.774897	-4.560171	-0.205677
C	2.628766	-0.737384	0.532959
C	1.637797	1.494495	0.756139
C	3.086663	1.921514	1.061475
C	4.035407	1.236793	0.095818
H	5.078026	1.395993	0.399387
S	2.675767	-2.476791	0.577929
N	3.785981	-0.204301	0.126810
N	1.553676	0.015398	0.834895
C	3.297936	3.453114	1.115650
C	3.688862	4.087170	-0.223385
C	4.343652	3.795136	2.179193
H	2.341879	3.895431	1.438807
H	4.711144	3.796719	-0.511719
H	3.008822	3.815727	-1.042644
H	3.678418	5.181836	-0.126832
H	4.031566	3.447787	3.174031
H	5.312732	3.328362	1.942345
H	4.501347	4.881615	2.228081
H	3.299952	1.522850	2.066784
H	1.026994	1.873213	1.584705
C	1.096017	2.034783	-0.549561
C	0.415567	3.257736	-0.545422

C	1.321887	1.382744	-1.765191
C	0.009106	3.844020	-1.742237
H	0.205213	3.759564	0.402165
C	0.914236	1.969244	-2.963540
H	1.800967	0.401256	-1.788375
C	0.268402	3.205395	-2.955606
H	-0.516047	4.799870	-1.726328
H	1.098293	1.453412	-3.906738
H	-0.048281	3.662845	-3.893840
C	-0.861977	0.177548	1.317399
H	-0.872714	1.226425	1.031097
C	-2.018202	-0.447691	1.657019
C	-3.191796	0.360810	2.121257
F	-3.010279	0.720317	3.404487
F	-3.356362	1.489660	1.431826
F	-4.341292	-0.313492	2.086470
O	0.397557	-1.811536	1.545589
C	0.338498	-0.634435	1.246778
H	3.911094	1.595691	-0.936585
C	6.034485	-0.875207	-0.739397
H	6.383121	0.144057	-0.901284
H	7.846843	-1.786056	-1.434163
H	-2.008512	-1.486667	1.992979
C	-0.595903	-3.981462	-0.690084
H	0.188504	-3.653626	0.004029
H	-0.834169	-5.036911	-0.517740
H	-0.235730	-3.842752	-1.719416
C	-1.749662	-1.925283	-0.748357
O	-1.788863	-3.257413	-0.464729
O	-0.722762	-1.398965	-1.149657
C	-3.345692	0.017295	-1.007404
C	-3.009782	-1.289984	-0.483509
O	-2.622412	0.913896	-1.433393
C	-5.373177	-1.097765	-0.475240
C	-6.752265	-1.235818	-0.329369
C	-4.841335	0.106799	-0.940695
C	-7.575717	-0.156054	-0.662496

H -7.187102 -2.168073 0.037645

C -5.653207 1.188279 -1.273381

C -7.033935 1.048638 -1.131131

H -8.657919 -0.251399 -0.555702

H -5.203721 2.116391 -1.632901

H -7.698815 1.876875 -1.382332

C -4.265404 -2.083263 -0.189474

H -4.349958 -2.971336 -0.838426

H -4.322577 -2.451795 0.848510

PCMopt/16_TS_RSd

Frequencies, energies and thermodynamic properties:

Lowest Vibrational Mode (1/cm) = -143.2623

2nd Lowest Vibrational Mode (1/cm) = 23.3705

E(RM062X) (a.u.) = -2420.00565045

Thermal correction to Enthalpy (a.u.) = 0.632936

Thermal correction to Gibbs Free Energy (a.u.) = 0.523535

Total Entropy (cal/Kmol) = 230.255

Esp(RM062X+D3) (a.u.) = -2422.42178984

Esp(D3) (a.u.) = -0.0098076255

Esp(RM062X) (a.u.) = -2422.4119822145

Optimised cartesian coordinates (Angstrom):

C -6.134969 -1.962717 0.447607

C -5.617777 -3.208937 0.062277

C -4.338105 -3.318257 -0.471524

C -3.587177 -2.150400 -0.610078

C -4.106725 -0.912281 -0.227502

H -6.225642 -4.105454 0.184692

H -3.930070 -4.284048 -0.770984

C -2.008220 -0.290682 -0.918610

C -1.099999 1.979162 -0.704521

C -2.555807 2.414503 -0.949226

C -3.504790 1.523720 -0.166648

H -4.542566 1.692706 -0.482473

S -1.961888 -1.994279 -1.238873

N -3.188506 0.120586 -0.441564

N -0.962398 0.548028 -1.069340

C	-2.798784	3.921636	-0.696989
C	-3.190414	4.272246	0.741987
C	-3.854669	4.450117	-1.670170
H	-1.850359	4.435181	-0.926385
H	-4.202019	3.905564	0.975803
H	-2.494209	3.865089	1.488342
H	-3.207295	5.364734	0.859235
H	-3.543483	4.308484	-2.714575
H	-4.816633	3.933427	-1.526541
H	-4.026455	5.522902	-1.505156
H	-2.738057	2.225082	-2.019647
H	-0.489208	2.531744	-1.429186
C	-0.582231	2.255465	0.691946
C	0.205229	3.388683	0.919727
C	-0.907980	1.423739	1.767785
C	0.644043	3.697782	2.206367
H	0.481424	4.035604	0.083072
C	-0.471179	1.732914	3.055550
H	-1.494639	0.515773	1.609172
C	0.302338	2.872501	3.278034
H	1.259015	4.583442	2.370399
H	-0.734488	1.080024	3.889352
H	0.645482	3.112107	4.285205
C	1.472933	0.842030	-1.318436
H	1.420901	1.833382	-0.876956
C	2.680764	0.281291	-1.583570
C	3.871203	1.199001	-1.566107
F	3.886125	1.947523	-2.681712
F	3.840221	2.048995	-0.537756
F	5.036436	0.557783	-1.531659
O	0.327447	-1.113843	-1.944432
C	0.295846	0.015385	-1.497876
H	-3.439245	1.693560	0.918104
C	-5.390053	-0.796516	0.309846
H	-5.793390	0.166349	0.621233
H	-7.139148	-1.904004	0.867742
H	2.789666	-0.659943	-2.124475

C	6.081527	0.374908	1.497403
H	6.540845	0.620333	0.529516
H	6.096705	1.253516	2.151543
H	6.654125	-0.442868	1.957310
C	4.484497	-1.058078	0.531316
O	4.724186	0.020824	1.329226
O	5.407543	-1.694075	0.057095
C	2.585231	-2.502486	-0.270845
C	3.070489	-1.301131	0.379205
O	3.091001	-3.172889	-1.168136
C	0.958595	-1.841740	1.324057
C	-0.195356	-1.973676	2.097908
C	1.275705	-2.829022	0.387511
C	-0.997439	-3.109197	1.933412
H	-0.450724	-1.228326	2.853670
C	0.481450	-3.958671	0.211516
C	-0.660525	-4.099254	1.002322
H	-1.891186	-3.230444	2.548747
H	0.765960	-4.715064	-0.523145
H	-1.297071	-4.980153	0.896962
C	2.048928	-0.789554	1.360562
H	2.453418	-0.666666	2.377975
H	1.638947	0.202378	1.074433

PCMopt/16_TS_RSe

Frequencies, energies and thermodynamic properties:

Lowest Vibrational Mode (1/cm) =	-162.4163
2nd Lowest Vibrational Mode (1/cm) =	17.8621
E(RM062X) (a.u.) =	-2420.00531249
Thermal correction to Enthalpy (a.u.) =	0.633087
Thermal correction to Gibbs Free Energy (a.u.) =	0.523779
Total Entropy (cal/Kmol) =	230.059
Esp(RM062X+D3) (a.u.) =	-2422.42138536
Esp(D3) (a.u.) =	-0.0095639577
Esp(RM062X) (a.u.) =	-2422.4118214023

Optimised cartesian coordinates (Angstrom):

C	6.738340	1.554640	0.400727

C	6.354453	2.870751	0.103186
C	5.061768	3.159674	-0.320598
C	4.162180	2.100058	-0.443622
C	4.550503	0.791569	-0.154085
H	7.077257	3.679916	0.208410
H	4.757671	4.181600	-0.548087
C	2.348685	0.450768	-0.729333
C	1.222026	-1.725009	-0.654965
C	2.618729	-2.295297	-0.946571
C	3.669045	-1.553892	-0.142506
H	4.676473	-1.818842	-0.487686
S	2.481805	2.174110	-0.933571
N	3.500696	-0.113573	-0.345575
N	1.219726	-0.263769	-0.908225
C	2.696187	-3.830792	-0.778038
C	3.044168	-4.299711	0.638114
C	3.689310	-4.417915	-1.782841
H	1.696755	-4.223135	-1.030057
H	4.087696	-4.054666	0.890239
H	2.391287	-3.864064	1.406987
H	2.946209	-5.392791	0.694302
H	3.398228	-4.185954	-2.816825
H	4.701859	-4.018712	-1.614533
H	3.742055	-5.510468	-1.677364
H	2.806097	-2.069660	-2.009050
H	0.566162	-2.164687	-1.413967
C	0.662090	-2.031685	0.718088
C	-0.286065	-3.048438	0.862384
C	1.075139	-1.317958	1.848339
C	-0.809849	-3.354007	2.117933
H	-0.626947	-3.601585	-0.016710
C	0.555647	-1.624832	3.105085
H	1.796220	-0.501792	1.758377
C	-0.387476	-2.644162	3.242043
H	-1.556718	-4.142682	2.215909
H	0.883215	-1.059189	3.978526
H	-0.800054	-2.878289	4.224223

C	-1.217011	-0.362245	-1.316127
H	-1.222178	-1.440369	-1.183170
C	-2.399806	0.290091	-1.513752
C	-3.571531	-0.447538	-2.088866
F	-3.321976	-0.766795	-3.372822
F	-3.825191	-1.602309	-1.463155
F	-4.682818	0.276956	-2.093634
O	0.070775	1.613652	-1.485758
C	-0.001441	0.422500	-1.252000
H	3.602784	-1.767995	0.934576
C	5.844859	0.496156	0.278538
H	6.147872	-0.521545	0.521182
H	7.755411	1.353785	0.737710
H	-2.393907	1.359145	-1.735513
C	-6.623290	-1.031415	0.485648
H	-6.830297	-1.324445	1.524301
H	-7.524123	-0.606855	0.030170
H	-6.306109	-1.924638	-0.071560
C	-4.422650	-0.365970	0.914093
O	-5.629770	-0.027130	0.422991
O	-4.248371	-1.433972	1.478284
C	-3.556169	2.021469	0.352846
C	-3.384659	0.626205	0.716078
O	-4.495032	2.612898	-0.170220
C	-1.419287	1.767029	1.366298
C	-0.151916	2.165454	1.786544
C	-2.251897	2.678062	0.711371
C	0.260720	3.475535	1.526888
H	0.509333	1.472332	2.311267
C	-1.853415	3.987065	0.457538
C	-0.579154	4.381491	0.866911
H	1.254743	3.798704	1.842761
H	-2.529916	4.674429	-0.054938
H	-0.231756	5.398586	0.677060
C	-2.112317	0.433814	1.489554
H	-1.487826	-0.386236	1.087403
H	-2.305765	0.172701	2.544502

PCMopt/16_TS_RSf

Frequencies, energies and thermodynamic properties:

Lowest Vibrational Mode (1/cm) = -230.2568

2nd Lowest Vibrational Mode (1/cm) = 15.2805

E(RM062X) (a.u.) = -2420.00465567

Thermal correction to Enthalpy (a.u.) = 0.633427

Thermal correction to Gibbs Free Energy (a.u.) = 0.524505

Total Entropy (cal/Kmol) = 229.247

Esp(RM062X+D3) (a.u.) = -2422.41815470

Esp(D3) (a.u.) = -0.0090710052

Esp(RM062X) (a.u.) = -2422.4090836948

Optimised cartesian coordinates (Angstrom):

C	6.637704	-1.546008	-1.463878
C	6.214245	-2.867139	-1.665435
C	4.940379	-3.275898	-1.281118
C	4.099648	-2.331681	-0.693154
C	4.529205	-1.020261	-0.485452
H	6.889344	-3.584791	-2.131877
H	4.605740	-4.301783	-1.437195
C	2.385154	-0.883556	0.344708
C	1.335999	1.185701	1.097858
C	2.759178	1.557159	1.562925
C	3.772007	1.151509	0.507654
H	4.792546	1.216662	0.907725
S	2.463686	-2.568448	-0.103444
N	3.540369	-0.240928	0.124633
N	1.294799	-0.265528	0.816827
C	2.912511	3.033106	2.001016
C	3.328033	3.991632	0.880110
C	3.902322	3.131151	3.163911
H	1.927112	3.354821	2.376004
H	4.369109	3.808268	0.571689
H	2.686509	3.918257	-0.009279
H	3.276686	5.026378	1.246925
H	3.567291	2.537794	4.026174
H	4.897774	2.767073	2.864395

H	4.014100	4.175413	3.487743
H	2.942144	0.928530	2.449569
H	0.682636	1.341073	1.964237
C	0.840695	2.031922	-0.056343
C	0.071519	3.167255	0.220689
C	1.243736	1.789609	-1.374438
C	-0.232100	4.082417	-0.787105
H	-0.274731	3.350657	1.241016
C	0.948072	2.707848	-2.381272
H	1.796703	0.882311	-1.628287
C	0.225921	3.865895	-2.086404
H	-0.824890	4.968050	-0.554174
H	1.281568	2.515493	-3.401905
H	-0.000171	4.585283	-2.874897
C	-1.101318	-0.326914	1.359340
H	-1.146888	0.756329	1.269693
C	-2.265340	-1.049358	1.533583
C	-3.401506	-0.407423	2.268195
F	-3.052544	-0.119263	3.533124
F	-3.792874	0.745202	1.716210
F	-4.469600	-1.206313	2.355305
O	0.233840	-2.262618	1.087635
C	0.099832	-1.050850	1.087343
H	3.710933	1.779790	-0.392970
C	5.803672	-0.602703	-0.871382
H	6.135605	0.424552	-0.724827
H	7.637944	-1.247050	-1.777894
H	-2.163505	-2.118325	1.733083
C	-1.767197	-4.685369	-0.335806
H	-2.380313	-5.299131	-1.010244
H	-0.703977	-4.872263	-0.517342
H	-2.021315	-4.946842	0.701378
C	-3.210105	-2.841014	-0.344747
O	-1.974682	-3.306229	-0.570027
O	-4.130520	-3.581048	-0.052076
C	-2.387590	-0.539329	-1.174274
C	-3.320723	-1.388565	-0.444942

O	-1.185148	-0.683007	-1.367935
C	-4.497311	0.533430	-1.214910
C	-5.392655	1.571551	-1.465427
C	-3.151550	0.684853	-1.552034
C	-4.911722	2.745195	-2.054966
H	-6.449121	1.477782	-1.205622
C	-2.661930	1.847698	-2.139755
C	-3.557701	2.887160	-2.389291
H	-5.601970	3.566721	-2.255696
H	-1.599921	1.928503	-2.378207
H	-3.206317	3.816713	-2.841513
C	-4.724706	-0.819493	-0.590016
H	-5.327781	-1.461841	-1.255046
H	-5.289019	-0.744658	0.348588

PCMopt/16_TS_SRa

Frequencies, energies and thermodynamic properties:

Lowest Vibrational Mode (1/cm) =	-149.7240
2nd Lowest Vibrational Mode (1/cm) =	18.2337
E(RM062X) (a.u.) =	-2420.01167034
Thermal correction to Enthalpy (a.u.) =	0.633051
Thermal correction to Gibbs Free Energy (a.u.) =	0.523822
Total Entropy (cal/Kmol) =	229.891
Esp(RM062X+D3) (a.u.) =	-2422.42779951
Esp(D3) (a.u.) =	-0.0095449338
Esp(RM062X) (a.u.) =	-2422.4182545762
Esp(RM062X+D3) a=1.00 (a.u.) =	-2422.44017264
Esp(RM062X) a=1.00 (a.u.) =	-2422.4306277062
Esp(RM062X+D3) a=1.05 (a.u.) =	-2422.43350095
Esp(RM062X) a=1.05 (a.u.) =	-2422.4239560162
Esp(RM062X+D3) a=1.10 (a.u.) =	-2422.42779951
Esp(RM062X) a=1.10 (a.u.) =	-2422.4182545762
Esp(RM062X+D3) a=1.15 (a.u.) =	-2422.42281541
Esp(RM062X) a=1.15 (a.u.) =	-2422.4132704762
Esp(RM062X+D3) a=1.20 (a.u.) =	-2422.41855206
Esp(RM062X) a=1.20 (a.u.) =	-2422.4090071262
Esp(RM062X+D3) SMD (a.u.) =	-2422.44716859

Esp(RM062X) SMD (a.u.) = -2422.4376236562

Esp(RM062X+D3) GAS (a.u.) = -2422.37736112

Esp(RM062X) GAS (a.u.) = -2422.3678161862

Optimised cartesian coordinates (Angstrom):

C	6.413013	-2.251818	-0.681046
C	5.898146	-3.293324	-1.467805
C	4.545875	-3.346271	-1.786810
C	3.718960	-2.335257	-1.295140
C	4.232914	-1.306523	-0.504419
H	6.566934	-4.070265	-1.838375
H	4.141854	-4.149797	-2.403006
C	2.017702	-0.694038	-0.589529
C	1.073746	1.302463	0.497046
C	2.247503	1.130856	1.474827
C	3.510489	0.761759	0.721731
H	4.307319	0.482892	1.422006
S	1.998397	-2.140138	-1.556796
N	3.234734	-0.404921	-0.118349
N	0.943259	0.085683	-0.341607
C	2.440363	2.329117	2.432503
C	3.385773	3.413865	1.907358
C	2.920280	1.828766	3.796558
H	1.445932	2.781979	2.578246
H	4.424928	3.050340	1.881409
H	3.117829	3.765624	0.901370
H	3.362488	4.279759	2.583635
H	2.197273	1.132059	4.244591
H	3.886614	1.307781	3.707758
H	3.059425	2.670705	4.489004
H	1.980175	0.258477	2.088639
H	0.167919	1.332320	1.117488
C	1.145173	2.551522	-0.357293
C	0.480290	3.708839	0.062573
C	1.907905	2.589439	-1.529149
C	0.603640	4.895067	-0.658628
H	-0.137600	3.684807	0.963837
C	2.031617	3.776633	-2.251222

H	2.409276	1.690396	-1.896661
C	1.386319	4.932971	-1.813294
H	0.082294	5.791188	-0.319863
H	2.632084	3.794747	-3.161505
H	1.483049	5.860593	-2.378837
C	-1.426004	0.667321	-0.740122
H	-1.296195	1.643732	-0.280842
C	-2.661842	0.260178	-1.134358
C	-3.747220	1.296095	-1.219159
F	-3.478283	2.168370	-2.203854
F	-4.942386	0.777062	-1.498033
F	-3.858072	2.012910	-0.099103
O	-0.367666	-1.232660	-1.653020
C	-0.307737	-0.234612	-0.963295
H	3.877275	1.578328	0.081726
C	5.592125	-1.243309	-0.189163
H	6.003358	-0.432900	0.411252
H	7.478228	-2.227422	-0.450786
H	-2.809382	-0.648117	-1.721224
C	-7.051815	-1.337678	-0.434891
H	-7.218370	-0.300861	-0.759912
H	-7.392741	-2.032724	-1.209634
H	-7.616614	-1.505013	0.492949
C	-5.057319	-0.805500	0.664828
O	-5.672906	-1.587586	-0.248653
O	-5.686362	0.009297	1.320684
C	-2.919933	-2.194278	0.299821
O	-3.214465	-3.030875	-0.553320
C	-3.635132	-1.028471	0.773798
C	-1.585898	-1.125481	1.949819
C	-0.461414	-0.934329	2.756814
C	-1.623824	-2.196310	1.054288
C	0.604922	-1.830405	2.645334
H	-0.416902	-0.107262	3.470465
C	-0.557154	-3.085338	0.927467
C	0.564678	-2.895228	1.731301
H	1.483276	-1.707276	3.283006

H	-0.612562	-3.899465	0.201835
H	1.416724	-3.573857	1.656995
C	-2.872606	-0.342781	1.877763
H	-2.673131	0.723386	1.665906
H	-3.412421	-0.371415	2.839308

PCMopt/16_TS_SRb

Frequencies, energies and thermodynamic properties:

Lowest Vibrational Mode (1/cm) = -163.3170

2nd Lowest Vibrational Mode (1/cm) = 19.8151

E(RM062X) (a.u.) = -2420.01182238

Thermal correction to Enthalpy (a.u.) = 0.633077

Thermal correction to Gibbs Free Energy (a.u.) = 0.523546

Total Entropy (cal/Kmol) = 230.528

Esp(RM062X+D3) (a.u.) = -2422.42734382

Esp(D3) (a.u.) = -0.0095691408

Esp(RM062X) (a.u.) = -2422.4177746792

Optimised cartesian coordinates (Angstrom):

C	6.519312	-2.003423	-0.547679
C	6.072485	-3.063629	-1.350761
C	4.733158	-3.174295	-1.707321
C	3.849285	-2.202344	-1.236529
C	4.295726	-1.155156	-0.429321
H	6.784566	-3.809411	-1.704304
H	4.382045	-3.992820	-2.335949
C	2.058687	-0.640109	-0.572928
C	0.997270	1.304809	0.498336
C	2.154604	1.185437	1.502934
C	3.450871	0.875775	0.780460
H	4.242001	0.631668	1.499901
S	2.129763	-2.080576	-1.546652
N	3.248845	-0.299627	-0.068210
N	0.943857	0.087864	-0.348591
C	2.269549	2.390543	2.464592
C	3.175756	3.518369	1.961465
C	2.739428	1.911480	3.839663
H	1.252291	2.797401	2.586301

H	4.230804	3.202944	1.958183
H	2.914113	3.859365	0.950039
H	3.097704	4.380920	2.637913
H	2.038231	1.182447	4.270619
H	3.730129	1.434803	3.773600
H	2.823975	2.758199	4.535088
H	1.912813	0.301087	2.110357
H	0.078054	1.288607	1.099138
C	1.028261	2.559652	-0.349737
C	0.293111	3.679239	0.054485
C	1.821160	2.641998	-1.499152
C	0.376520	4.873946	-0.658413
H	-0.349358	3.618244	0.936636
C	1.905046	3.837588	-2.212908
H	2.377878	1.771303	-1.855126
C	1.189485	4.957521	-1.789497
H	-0.199483	5.740615	-0.331783
H	2.529586	3.890819	-3.105446
H	1.255414	5.891785	-2.348496
C	-1.439380	0.560848	-0.798468
H	-1.371438	1.526941	-0.305736
C	-2.644165	0.110465	-1.241927
C	-3.764587	1.109095	-1.331390
F	-3.519379	1.985923	-2.318514
F	-4.944160	0.556868	-1.609901
F	-3.898410	1.826052	-0.213370
O	-0.268506	-1.270064	-1.714710
C	-0.274490	-0.281591	-1.008507
H	3.796214	1.709825	0.151140
C	5.641635	-1.033695	-0.076449
H	6.000256	-0.208340	0.537010
H	7.575671	-1.933208	-0.287952
H	-2.732909	-0.781113	-1.865322
C	-7.004098	-0.100065	0.786038
H	-7.570385	-1.009876	1.029930
H	-7.334024	0.726711	1.424234
H	-7.181494	0.147090	-0.270197

C -5.015447 -1.259652 0.315808
O -5.623380 -0.274425 1.031767
O -5.658783 -1.972331 -0.430914
C -2.767323 -2.402688 0.130102
O -2.959989 -3.244588 -0.746027
C -3.595385 -1.302236 0.572582
C -1.609600 -1.257254 1.855566
C -0.548017 -0.992746 2.725253
C -1.517673 -2.317955 0.951925
C 0.585636 -1.807419 2.667965
H -0.603999 -0.172919 3.446170
C -0.382384 -3.124276 0.878190
C 0.675567 -2.862098 1.745476
H 1.414869 -1.628018 3.355965
H -0.337207 -3.931713 0.144387
H 1.578044 -3.475865 1.714892
C -2.948193 -0.572964 1.724818
H -2.820336 0.508198 1.540606
H -3.527211 -0.661486 2.659340

PCMopt/16_TS_SRc

Frequencies, energies and thermodynamic properties:

Lowest Vibrational Mode (1/cm) = -163.7332

2nd Lowest Vibrational Mode (1/cm) = 19.7263

E(RM062X) (a.u.) = -2420.01182238

Thermal correction to Enthalpy (a.u.) = 0.633079

Thermal correction to Gibbs Free Energy (a.u.) = 0.523555

Total Entropy (cal/Kmol) = 230.512

Esp(RM062X+D3) (a.u.) = -2422.42733272

Esp(D3) (a.u.) = -0.0095690114

Esp(RM062X) (a.u.) = -2422.4177637086

Optimised cartesian coordinates (Angstrom):

C 6.520000 -2.002644 -0.547693
C 6.073268 -3.063242 -1.350305
C 4.733902 -3.174323 -1.706601
C 3.849903 -2.202375 -1.236047
C 4.296256 -1.154786 -0.429310

H	6.785449	-3.809006	-1.703685
H	4.382850	-3.993171	-2.334844
C	2.059072	-0.640260	-0.572761
C	0.997332	1.304593	0.498272
C	2.154862	1.185657	1.502695
C	3.451082	0.876335	0.779983
H	4.242503	0.632716	1.499268
S	2.130286	-2.081133	-1.545888
N	3.249248	-0.299360	-0.068331
N	0.944156	0.087570	-0.348551
C	2.269622	2.390859	2.464239
C	3.175350	3.518923	1.960754
C	2.740048	1.912172	3.839259
H	1.252257	2.797385	2.586145
H	4.230538	3.203955	1.957699
H	2.913662	3.859393	0.949164
H	3.096836	4.381711	2.636843
H	2.038925	1.183512	4.270982
H	3.730607	1.435232	3.772934
H	2.825197	2.759190	4.534251
H	1.913447	0.301275	2.110207
H	0.078186	1.288170	1.099172
C	1.027842	2.559383	-0.349899
C	0.292561	3.678837	0.054456
C	1.820400	2.641826	-1.499537
C	0.375518	4.873518	-0.658533
H	-0.349674	3.617732	0.936768
C	1.903835	3.837394	-2.213388
H	2.377210	1.771223	-1.855593
C	1.188155	4.957200	-1.789847
H	-0.200584	5.740083	-0.331799
H	2.528112	3.890703	-3.106106
H	1.253732	5.891445	-2.348919
C	-1.439126	0.560057	-0.798350
H	-1.371155	1.526228	-0.305770
C	-2.644021	0.109568	-1.241662
C	-3.764165	1.108456	-1.331372

F	-3.518681	1.985140	-2.318563
F	-4.943883	0.556533	-1.609950
F	-3.897860	1.825547	-0.213397
O	-0.268222	-1.271166	-1.713912
C	-0.274286	-0.282341	-1.008166
H	3.795931	1.710374	0.150374
C	5.642191	-1.032926	-0.076683
H	6.000733	-0.207274	0.536422
H	7.576387	-1.932106	-0.288162
H	-2.732610	-0.781735	-1.865465
C	-7.004177	-0.099140	0.785579
H	-7.570557	-1.008732	1.030066
H	-7.333884	0.728050	1.423346
H	-7.181652	0.147432	-0.270776
C	-5.015758	-1.259239	0.315739
O	-5.623444	-0.273579	1.031231
O	-5.659229	-1.972263	-0.430506
C	-2.767990	-2.403060	0.130690
O	-2.961008	-3.245433	-0.744877
C	-3.595601	-1.301853	0.572325
C	-1.609859	-1.256743	1.855257
C	-0.547874	-0.991562	2.724269
C	-1.518334	-2.318182	0.952436
C	0.585700	-1.806348	2.667164
H	-0.603446	-0.171001	3.444390
C	-0.383217	-3.124783	0.879060
C	0.675099	-2.861954	1.745679
H	1.415446	-1.626056	3.354311
H	-0.338391	-3.932830	0.145910
H	1.577507	-3.475824	1.715261
C	-2.948374	-0.572451	1.724420
H	-3.527366	-0.660970	2.658966
H	-2.820836	0.508690	1.540017

PCMopt/16_TS_SRd

Frequencies, energies and thermodynamic properties:

Lowest Vibrational Mode (1/cm) = -271.1705

2nd Lowest Vibrational Mode (1/cm) = 17.7705

E(RM062X) (a.u.) = -2420.01976482

Thermal correction to Enthalpy (a.u.) = 0.633572

Thermal correction to Gibbs Free Energy (a.u.) = 0.525589

Total Entropy (cal/Kmol) = 227.268

Esp(RM062X+D3) (a.u.) = -2422.42775276

Esp(D3) (a.u.) = -0.0091100418

Esp(RM062X) (a.u.) = -2422.4186427182

Optimised cartesian coordinates (Angstrom):

C	7.217928	-0.837850	0.612744
C	7.212037	-2.035810	-0.114679
C	6.033997	-2.529388	-0.668531
C	4.864030	-1.795381	-0.480086
C	4.872306	-0.606519	0.252527
H	8.141410	-2.588768	-0.251864
H	6.024857	-3.460336	-1.235746
C	2.650018	-0.704407	-0.339530
C	0.993985	1.010071	0.203397
C	1.837806	1.180231	1.475289
C	3.316499	1.161909	1.129021
H	3.932219	1.099759	2.036107
S	3.246949	-2.187802	-1.039627
N	3.598682	-0.033349	0.329774
N	1.403912	-0.244566	-0.471279
C	1.421502	2.400258	2.326815
C	2.148646	3.700176	1.970618
C	1.601725	2.088379	3.813729
H	0.343414	2.546763	2.146004
H	3.210248	3.648837	2.259681
H	2.094068	3.939575	0.899401
H	1.701713	4.536878	2.525953
H	0.998407	1.220198	4.114391
H	2.656108	1.868493	4.045012
H	1.298743	2.947380	4.429081
H	1.623756	0.274448	2.066168
H	-0.034907	0.833208	0.543539
C	1.011031	2.192389	-0.739424

C	-0.052766	3.101153	-0.682452
C	2.066309	2.428121	-1.627112
C	-0.037633	4.252360	-1.469110
H	-0.898224	2.883284	-0.023803
C	2.079241	3.578615	-2.416197
H	2.886203	1.710614	-1.716878
C	1.032856	4.497810	-2.330579
H	-0.868878	4.957004	-1.415603
H	2.908647	3.755312	-3.102441
H	1.044857	5.397912	-2.946894
C	-0.856802	-0.424402	-1.366027
H	-0.931287	0.659995	-1.385877
C	-2.011007	-1.180474	-1.390101
C	-3.183986	-0.605130	-2.133483
F	-2.982420	-0.715788	-3.455814
F	-4.340447	-1.222955	-1.883283
F	-3.362133	0.694720	-1.881837
O	0.660684	-2.244633	-1.299357
C	0.384571	-1.074190	-1.077788
H	3.623329	2.052391	0.560042
C	6.050416	-0.105754	0.807879
H	6.062586	0.829189	1.366939
H	8.152963	-0.467348	1.033390
H	-1.939874	-2.269372	-1.464710
C	-0.372280	-3.895549	1.488839
H	-0.026972	-3.614698	2.493008
H	-0.585766	-4.968451	1.451412
H	0.403833	-3.631930	0.755908
C	-1.533944	-1.886548	1.150407
O	-1.587561	-3.234492	1.169137
O	-0.510012	-1.289732	1.430302
C	-3.012002	0.138415	0.890593
O	-2.194950	1.052069	0.967757
C	-2.770043	-1.287791	0.683436
C	-5.111343	-0.949465	0.882311
C	-6.502319	-1.036563	0.907932
C	-4.493964	0.302905	0.941956

C -7.247389 0.141668 1.000755
H -7.004132 -2.004902 0.857029
C -5.229122 1.483640 1.033903
C -6.619503 1.393977 1.063770
H -8.337306 0.087923 1.023940
H -4.716165 2.446458 1.077422
H -7.226336 2.298048 1.133915
C -4.079685 -2.048638 0.791610
H -4.275075 -2.721073 -0.057293
H -4.098769 -2.675377 1.698452

PCMopt/16_TS_SRe

Frequencies, energies and thermodynamic properties:

Lowest Vibrational Mode (1/cm) = -243.9716
2nd Lowest Vibrational Mode (1/cm) = 19.6754
E(RM062X) (a.u.) = -2420.01645764
Thermal correction to Enthalpy (a.u.) = 0.633433
Thermal correction to Gibbs Free Energy (a.u.) = 0.525736
Total Entropy (cal/Kmol) = 226.668
Esp(RM062X+D3) (a.u.) = -2422.42624801
Esp(D3) (a.u.) = -0.0092622237
Esp(RM062X) (a.u.) = -2422.4169857863

Optimised cartesian coordinates (Angstrom):

C -7.240602 -0.752178 -0.478746
C -7.225019 -1.988811 0.181366
C -6.034572 -2.528703 0.659095
C -4.861323 -1.801864 0.461609
C -4.879488 -0.574554 -0.203822
H -8.157139 -2.535137 0.326110
H -6.017584 -3.488838 1.175060
C -2.638494 -0.737695 0.291847
C -0.965596 0.976117 -0.233732
C -1.879353 1.266361 -1.435533
C -3.336544 1.238670 -1.008530
H -4.000018 1.264502 -1.882721
S -3.232425 -2.242952 0.942018
N -3.599929 -0.015636 -0.298627

N	-1.373791	-0.308495	0.385566
C	-1.493156	2.550437	-2.205590
C	-2.183203	3.823079	-1.705082
C	-1.759936	2.364071	-3.700609
H	-0.405391	2.673788	-2.074190
H	-3.259078	3.805806	-1.939943
H	-2.068432	3.975356	-0.622905
H	-1.754536	4.697162	-2.215213
H	-1.183320	1.521452	-4.107494
H	-2.827767	2.170172	-3.889188
H	-1.483480	3.270157	-4.257902
H	-1.728462	0.419906	-2.124705
H	0.042471	0.795348	-0.635284
C	-0.900500	2.097104	0.780898
C	0.172580	2.993824	0.713244
C	-1.895474	2.289631	1.744595
C	0.223675	4.094731	1.566994
H	0.974441	2.804471	-0.005844
C	-1.842429	3.390424	2.600327
H	-2.719124	1.577611	1.843448
C	-0.789332	4.300625	2.505182
H	1.061521	4.790712	1.505150
H	-2.625523	3.534554	3.345909
H	-0.749927	5.161956	3.173474
C	0.884212	-0.518181	1.291749
H	0.973963	0.563736	1.314773
C	2.021427	-1.289309	1.362233
C	3.185355	-0.699532	2.109499
F	2.959126	-0.775060	3.430579
F	4.343827	-1.324991	1.896719
F	3.368829	0.593222	1.826993
O	-0.653796	-2.318607	1.198015
C	-0.365339	-1.149608	0.991694
H	-3.594144	2.081459	-0.349949
C	-6.070755	-0.026557	-0.681461
H	-6.091508	0.938413	-1.186452
H	-8.185757	-0.345879	-0.839353

H	1.944310	-2.376829	1.442883
C	-0.472252	-2.377388	-1.958571
H	-0.866424	-2.979224	-1.126591
H	-1.238179	-1.681750	-2.319462
H	-0.165460	-3.047444	-2.771583
C	1.734886	-2.275650	-1.189912
O	0.621649	-1.585579	-1.517472
O	1.769162	-3.489431	-1.270672
C	2.979773	-0.027101	-0.966226
O	2.102408	0.828721	-1.079988
C	2.848579	-1.457428	-0.731912
C	5.161759	-0.939512	-0.870015
C	6.555217	-0.913545	-0.850164
C	4.446306	0.255909	-0.977899
C	7.205541	0.319152	-0.949218
H	7.131612	-1.836524	-0.760348
C	5.086403	1.490268	-1.076049
C	6.480084	1.513695	-1.062094
H	8.296420	0.354186	-0.938333
H	4.500039	2.407655	-1.158093
H	7.013314	2.462810	-1.136433
C	4.216677	-2.112258	-0.788630
H	4.438158	-2.759436	0.072955
H	4.303787	-2.748690	-1.685535

PCMopt/16_TS_SRf

Frequencies, energies and thermodynamic properties:

Lowest Vibrational Mode (1/cm) = -209.5902

2nd Lowest Vibrational Mode (1/cm) = 16.0920

E(RM062X) (a.u.) = -2420.00621907

Thermal correction to Enthalpy (a.u.) = 0.633162

Thermal correction to Gibbs Free Energy (a.u.) = 0.523628

Total Entropy (cal/Kmol) = 230.534

Esp(RM062X+D3) (a.u.) = -2422.41928245

Esp(D3) (a.u.) = -0.0091672872

Esp(RM062X) (a.u.) = -2422.4101151628

Optimised cartesian coordinates (Angstrom):

C	-6.805450	2.213190	0.102302
C	-6.470453	3.285158	-0.737412
C	-5.177867	3.429223	-1.231866
C	-4.228983	2.474892	-0.866678
C	-4.563680	1.414646	-0.022397
H	-7.232665	4.015028	-1.010478
H	-4.912989	4.259508	-1.886689
C	-2.350025	0.936652	-0.422408
C	-1.178296	-1.038771	0.437140
C	-2.132532	-0.878629	1.632260
C	-3.533565	-0.559284	1.141367
H	-4.181748	-0.266043	1.977379
S	-2.537843	2.413698	-1.326509
N	-3.467407	0.579773	0.221868
N	-1.230448	0.196930	-0.384085
C	-2.089178	-2.069915	2.617140
C	-3.079938	-3.193568	2.297813
C	-2.305583	-1.574137	4.048312
H	-1.068571	-2.483397	2.558387
H	-4.116155	-2.866797	2.477804
H	-3.005264	-3.549479	1.260900
H	-2.890241	-4.048831	2.961449
H	-1.539255	-0.841656	4.338506
H	-3.292629	-1.096891	4.154652
H	-2.264621	-2.413127	4.757049
H	-1.768778	0.009790	2.174727
H	-0.159903	-1.081464	0.848481
C	-1.406633	-2.286011	-0.389271
C	-0.600361	-3.403073	-0.137930
C	-2.415999	-2.370095	-1.353358
C	-0.831678	-4.601983	-0.810462
H	0.222265	-3.312835	0.577452
C	-2.645213	-3.570390	-2.027037
H	-3.028944	-1.497915	-1.595418
C	-1.861315	-4.690560	-1.748835
H	-0.200675	-5.468349	-0.606695
H	-3.437517	-3.628211	-2.774527

H	-2.042946	-5.628387	-2.275601
C	1.013476	-0.314506	-1.195379
H	0.791795	-1.374683	-1.100156
C	2.314074	0.085122	-1.349142
C	3.259453	-0.874816	-2.012057
F	3.093154	-0.818195	-3.345291
F	4.540099	-0.619416	-1.783096
F	3.021490	-2.143862	-1.656919
O	-0.018130	1.810755	-1.455735
C	-0.037043	0.660395	-1.055551
H	-3.998618	-1.409510	0.620639
C	-5.859802	1.262524	0.472688
H	-6.128709	0.426172	1.116907
H	-7.826493	2.117483	0.472005
H	2.552467	1.140600	-1.507121
C	3.824852	-3.418501	1.027785
H	3.101743	-3.758785	0.273228
H	4.791508	-3.905871	0.864545
H	3.440811	-3.669361	2.025888
C	2.985577	-1.232388	1.083030
O	4.054266	-2.026350	0.905312
O	1.893365	-1.693856	1.397981
C	4.497803	0.840127	0.660291
O	5.624274	0.393441	0.504643
C	3.206172	0.166615	0.811583
C	2.851054	2.488852	1.120665
C	2.331789	3.775369	1.243921
C	4.183850	2.307338	0.743530
C	3.173069	4.863914	0.991945
H	1.289900	3.935388	1.528856
C	5.032356	3.385155	0.504758
C	4.513116	4.674503	0.629753
H	2.780375	5.878524	1.079572
H	6.072510	3.210761	0.221974
H	5.147954	5.541805	0.441438
C	2.195940	1.148719	1.349348
H	2.013477	0.986820	2.426766

H 1.205571 1.070362 0.872812

PCMopt/16_TS_SSa

Frequencies, energies and thermodynamic properties:

Lowest Vibrational Mode (1/cm) = -228.4682

2nd Lowest Vibrational Mode (1/cm) = 18.2967

E(RM062X) (a.u.) = -2420.01576747

Thermal correction to Enthalpy (a.u.) = 0.633283

Thermal correction to Gibbs Free Energy (a.u.) = 0.523926

Total Entropy (cal/Kmol) = 230.161

Esp(RM062X+D3) (a.u.) = -2422.42706155

Esp(D3) (a.u.) = -0.0093384844

Esp(RM062X) (a.u.) = -2422.4177230656

Esp(RM062X+D3) a=1.00 (a.u.) = -2422.43700335

Esp(RM062X) a=1.00 (a.u.) = -2422.4276648656

Esp(RM062X+D3) a=1.05 (a.u.) = -2422.43151760

Esp(RM062X) a=1.05 (a.u.) = -2422.4221791156

Esp(RM062X+D3) a=1.10 (a.u.) = -2422.42706155

Esp(RM062X) a=1.10 (a.u.) = -2422.4177230656

Esp(RM062X+D3) a=1.15 (a.u.) = -2422.42327171

Esp(RM062X) a=1.15 (a.u.) = -2422.4139332256

Esp(RM062X+D3) a=1.20 (a.u.) = -2422.42012651

Esp(RM062X) a=1.20 (a.u.) = -2422.4107880256

Esp(RM062X+D3) SMD (a.u.) = -2422.44795042

Esp(RM062X) SMD (a.u.) = -2422.4386119356

Esp(RM062X+D3) GAS (a.u.) = -2422.39474331

Esp(RM062X) GAS (a.u.) = -2422.3854048256

Optimised cartesian coordinates (Angstrom):

C -6.439982 2.354925 0.105587

C -6.044571 3.417978 -0.718141

C -4.748860 3.487355 -1.221841

C -3.859662 2.468189 -0.883644

C -4.254806 1.415717 -0.056061

H -6.760514 4.200085 -0.971172

H -4.436939 4.311018 -1.864345

C -2.077742 0.802238 -0.486529

C -1.020672 -1.245888 0.337031

C	-1.956028	-1.045315	1.540293
C	-3.339240	-0.637055	1.065414
H	-3.963213	-0.319902	1.911262
S	-2.178153	2.309205	-1.360663
N	-3.212782	0.508290	0.162840
N	-1.014451	-0.007734	-0.475133
C	-1.983222	-2.246253	2.512571
C	-3.039141	-3.306128	2.183397
C	-2.166241	-1.752265	3.948987
H	-0.990542	-2.722361	2.450184
H	-4.054133	-2.921454	2.369623
H	-2.987411	-3.652691	1.141711
H	-2.898962	-4.179323	2.835963
H	-1.354461	-1.072675	4.243905
H	-3.120595	-1.213822	4.061258
H	-2.178341	-2.598746	4.649978
H	-1.522664	-0.187460	2.080431
H	-0.006582	-1.313488	0.752732
C	-1.319788	-2.463400	-0.510466
C	-0.597127	-3.640279	-0.286898
C	-2.329356	-2.459496	-1.479236
C	-0.903656	-4.805383	-0.988087
H	0.215629	-3.641456	0.443272
C	-2.636912	-3.624252	-2.182551
H	-2.881954	-1.542834	-1.700806
C	-1.931393	-4.801563	-1.931825
H	-0.333857	-5.716474	-0.800882
H	-3.429102	-3.609530	-2.932251
H	-2.173377	-5.711773	-2.482122
C	1.263608	-0.561071	-1.190479
H	1.084007	-1.612008	-0.982329
C	2.552456	-0.111767	-1.346239
C	3.579231	-1.103835	-1.815318
F	3.456712	-1.299035	-3.137253
F	4.839132	-0.718132	-1.611403
F	3.429619	-2.301529	-1.237466
O	0.270579	1.557247	-1.533488

C	0.215071	0.415255	-1.106868
H	-3.856865	-1.451768	0.537131
C	-5.553324	1.338873	0.449336
H	-5.869693	0.510739	1.082462
H	-7.461578	2.317865	0.484293
H	2.736346	0.910759	-1.687189
C	2.891940	-3.005157	1.864807
H	3.575278	-3.015586	2.724089
H	1.932086	-3.450099	2.148145
H	3.348301	-3.577459	1.044952
C	3.697264	-0.923408	1.168753
O	2.615789	-1.677996	1.451057
O	4.825509	-1.361171	1.288981
C	2.165573	1.124871	1.063656
O	1.062946	0.666822	1.365100
C	3.374755	0.415994	0.694774
C	3.807100	2.747386	0.562317
C	4.311811	4.029783	0.346977
C	2.461914	2.578031	0.897924
C	3.451888	5.121435	0.488035
H	5.358706	4.182259	0.076786
C	1.595694	3.661242	1.039156
C	2.103575	4.941990	0.831392
H	3.833572	6.131262	0.327204
H	0.547099	3.493090	1.292867
H	1.453290	5.812627	0.930881
C	4.499665	1.408984	0.486090
H	5.268487	1.311188	1.270794
H	5.022367	1.281528	-0.474151

PCMopt/16_TS_SSb

Frequencies, energies and thermodynamic properties:

Lowest Vibrational Mode (1/cm) = -272.7859

2nd Lowest Vibrational Mode (1/cm) = 16.3961

E(RM062X) (a.u.) = -2420.01903367

Thermal correction to Enthalpy (a.u.) = 0.633439

Thermal correction to Gibbs Free Energy (a.u.) = 0.524981

Total Entropy (cal/Kmol) = 228.270

Esp(RM062X+D3) (a.u.) = -2422.42747506

Esp(D3) (a.u.) = -0.0091529041

Esp(RM062X) (a.u.) = -2422.4183221559

Optimised cartesian coordinates (Angstrom):

C	-6.694761	2.071273	0.093789
C	-6.406921	3.087384	-0.827490
C	-5.135999	3.208716	-1.382927
C	-4.162255	2.288925	-0.997475
C	-4.450385	1.283252	-0.072355
H	-7.187378	3.791502	-1.116349
H	-4.907293	3.997165	-2.100290
C	-2.247999	0.794688	-0.532849
C	-1.024627	-1.105582	0.397939
C	-1.913472	-0.858613	1.626299
C	-3.343248	-0.585171	1.192655
H	-3.947257	-0.227314	2.037145
S	-2.486011	2.218305	-1.515042
N	-3.337053	0.475465	0.182308
N	-1.132175	0.065011	-0.505050
C	-1.801856	-1.964812	2.699051
C	-2.790970	-3.121209	2.525076
C	-1.945763	-1.357089	4.095823
H	-0.779860	-2.371333	2.617092
H	-3.821231	-2.792868	2.734437
H	-2.766081	-3.556034	1.516068
H	-2.553688	-3.919859	3.242046
H	-1.858622	-2.135144	4.867410
H	-1.171681	-0.599438	4.282465
H	-2.929840	-0.877014	4.217002
H	-1.515412	0.070754	2.065820
H	0.010146	-1.088724	0.763395
C	-1.280974	-2.409000	-0.324980
C	-0.442874	-3.495993	-0.047713
C	-2.344423	-2.575119	-1.218422
C	-0.694007	-4.742715	-0.619230
H	0.418129	-3.345502	0.609704

C	-2.593221	-3.822110	-1.792814
H	-2.985369	-1.729163	-1.480583
C	-1.775553	-4.910325	-1.485486
H	-0.037961	-5.584948	-0.394215
H	-3.427481	-3.942460	-2.485337
H	-1.972548	-5.885274	-1.933637
C	1.116533	-0.421590	-1.305215
H	0.937638	-1.476207	-1.113939
C	2.416651	0.020881	-1.438431
C	3.399133	-0.961744	-2.019195
F	3.227557	-1.041788	-3.347252
F	4.679807	-0.641896	-1.830119
F	3.226629	-2.198444	-1.537056
O	0.051088	1.673084	-1.629205
C	0.058525	0.533634	-1.190725
H	-3.829016	-1.477849	0.770823
C	-5.722835	1.155050	0.485544
H	-5.954590	0.361456	1.195091
H	-7.698289	1.991546	0.512178
H	2.609940	1.054752	-1.739274
C	4.473192	-3.014152	1.274182
H	3.919004	-3.576484	0.510225
H	5.531419	-3.292873	1.252724
H	4.044320	-3.236919	2.260424
C	3.190052	-1.068657	0.989449
O	4.419073	-1.624527	0.999735
O	2.193547	-1.719067	1.247331
C	2.132081	1.212105	0.993910
O	0.970784	0.913856	1.259355
C	3.220030	0.331138	0.588388
C	4.036571	2.563627	0.675047
C	4.755454	3.754785	0.577442
C	2.667647	2.601341	0.948176
C	4.080076	4.962664	0.767555
H	5.825238	3.748163	0.359256
C	1.984207	3.801753	1.139017
C	2.705434	4.990169	1.045965

H 4.630153 5.902694 0.696623
H 0.912052 3.794560 1.345447
H 2.203512 5.948840 1.185736
C 4.506524 1.137156 0.523210
H 5.189571 0.861139 1.343687
H 5.071563 1.001028 -0.408453

PCMopt/16_TS_SSc

Frequencies, energies and thermodynamic properties:

Lowest Vibrational Mode (1/cm) = -139.9894

2nd Lowest Vibrational Mode (1/cm) = 18.5385

E(RM062X) (a.u.) = -2420.00858139

Thermal correction to Enthalpy (a.u.) = 0.633168

Thermal correction to Gibbs Free Energy (a.u.) = 0.523508

Total Entropy (cal/Kmol) = 230.800

Esp(RM062X+D3) (a.u.) = -2422.42636594

Esp(D3) (a.u.) = -0.0095707929

Esp(RM062X) (a.u.) = -2422.4167951471

Optimised cartesian coordinates (Angstrom):

C 6.804401 -2.161809 -0.049880
C 6.392590 -3.460369 -0.385821
C 5.047166 -3.758015 -0.572033
C 4.122646 -2.725516 -0.410485
C 4.535154 -1.437348 -0.067479
H 7.137020 -4.247385 -0.506861
H 4.722524 -4.764119 -0.838160
C 2.264268 -1.113085 -0.223534
C 1.131829 1.044243 0.131480
C 2.318318 1.321478 1.071140
C 3.610322 0.841196 0.437412
H 4.434258 0.887382 1.160471
S 2.382439 -2.809851 -0.590090
N 3.450759 -0.560140 0.045043
N 1.119034 -0.397323 -0.229550
C 2.395816 2.789433 1.553121
C 3.212069 3.714273 0.643369
C 2.954402 2.846924 2.976341

H	1.358937	3.163673	1.587941
H	4.284555	3.470778	0.699452
H	2.903335	3.669274	-0.409754
H	3.098212	4.753204	0.982910
H	2.322674	2.289323	3.681408
H	3.969872	2.422881	3.019840
H	3.014616	3.888142	3.322505
H	2.140958	0.689662	1.955586
H	0.222653	1.206873	0.728568
C	1.094838	1.931367	-1.095385
C	0.315421	3.093254	-1.067215
C	1.858516	1.646767	-2.231228
C	0.329275	3.978235	-2.143272
H	-0.308325	3.305723	-0.194450
C	1.872587	2.532917	-3.309021
H	2.445459	0.726575	-2.291444
C	1.115753	3.703524	-3.263229
H	-0.279971	4.882211	-2.109127
H	2.475075	2.303631	-4.188746
H	1.127658	4.395260	-4.106448
C	-1.239926	-0.167571	-0.882569
H	-1.122839	0.911932	-0.880560
C	-2.454224	-0.688205	-1.184053
C	-3.444863	0.200886	-1.881276
F	-3.175356	0.213499	-3.198888
F	-4.704943	-0.205781	-1.764189
F	-3.374895	1.464780	-1.465295
O	-0.101275	-2.243576	-0.765347
C	-0.103861	-1.037270	-0.620818
H	3.889573	1.428422	-0.450066
C	5.885589	-1.131321	0.113420
H	6.215073	-0.123532	0.362581
H	7.865680	-1.951466	0.083210
H	-2.624525	-1.763168	-1.281963
C	-6.998577	-1.948259	-0.346919
H	-7.218615	-2.914646	0.127700
H	-7.890645	-1.313072	-0.330759

H	-6.690209	-2.133809	-1.386089
C	-4.787050	-1.848734	0.410805
O	-5.992360	-1.246912	0.355990
O	-4.635213	-2.991837	0.000556
C	-3.783549	0.342035	1.360635
O	-4.634353	1.213074	1.174644
C	-3.732851	-1.045197	0.975588
C	-1.757743	-0.597629	2.187324
C	-0.538294	-0.609234	2.866535
C	-2.479806	0.593680	2.064542
C	-0.068791	0.584089	3.421756
H	0.029428	-1.535479	2.983703
C	-1.997034	1.795256	2.583815
C	-0.781632	1.783362	3.270661
H	0.863957	0.578769	3.989363
H	-2.579544	2.711680	2.468279
H	-0.388380	2.703724	3.707604
C	-2.525673	-1.734088	1.565955
H	-1.918652	-2.288922	0.834178
H	-2.807567	-2.467766	2.342622

PCMopt/16_TS_SSd

Frequencies, energies and thermodynamic properties:

Lowest Vibrational Mode (1/cm) = -145.7555

2nd Lowest Vibrational Mode (1/cm) = 15.2490

E(RM062X) (a.u.) = -2420.00766941

Thermal correction to Enthalpy (a.u.) = 0.633193

Thermal correction to Gibbs Free Energy (a.u.) = 0.522740

Total Entropy (cal/Kmol) = 232.466

Esp(RM062X+D3) (a.u.) = -2422.42546929

Esp(D3) (a.u.) = -0.0095913615

Esp(RM062X) (a.u.) = -2422.4158779285

Optimised cartesian coordinates (Angstrom):

C	6.664295	-2.381346	0.012966
C	6.199383	-3.659797	-0.330850
C	4.845135	-3.895606	-0.539874
C	3.966118	-2.822009	-0.392472

C	4.431019	-1.554015	-0.041715
H	6.909064	-4.479962	-0.439780
H	4.478947	-4.885810	-0.811825
C	2.179114	-1.128274	-0.229303
C	1.136576	1.074046	0.127589
C	2.328686	1.300449	1.073418
C	3.602763	0.764067	0.448791
H	4.422648	0.774631	1.177691
S	2.227481	-2.826951	-0.602991
N	3.385502	-0.629037	0.055408
N	1.065341	-0.364199	-0.239862
C	2.466362	2.764608	1.553894
C	3.324022	3.652686	0.645520
C	3.021595	2.802120	2.979172
H	1.446071	3.182399	1.584079
H	4.385264	3.364662	0.705720
H	3.017209	3.619012	-0.408558
H	3.252589	4.696050	0.983042
H	2.362669	2.276851	3.684088
H	4.016838	2.333204	3.028743
H	3.127120	3.840998	3.321478
H	2.118832	0.677791	1.957463
H	0.233930	1.272270	0.723364
C	1.137140	1.964348	-1.097352
C	0.401780	3.154497	-1.069111
C	1.892547	1.653142	-2.231795
C	0.451294	4.040127	-2.143550
H	-0.215794	3.388688	-0.197472
C	1.942301	2.539897	-3.308040
H	2.445039	0.711814	-2.292193
C	1.229595	3.737910	-3.262118
H	-0.123933	4.966114	-2.109295
H	2.537847	2.289667	-4.186789
H	1.269257	4.429926	-4.104255
C	-1.285367	-0.032190	-0.878553
H	-1.136525	1.041710	-0.826373
C	-2.515563	-0.500224	-1.202792

C	-3.477343	0.451034	-1.857867
F	-3.229047	0.485167	-3.180186
F	-4.750361	0.103680	-1.725759
F	-3.336479	1.699969	-1.412278
O	-0.219715	-2.148721	-0.831609
C	-0.179242	-0.947642	-0.650606
H	3.914182	1.337781	-0.436861
C	5.790951	-1.309975	0.161720
H	6.162256	-0.318426	0.417341
H	7.731673	-2.219812	0.164189
H	-2.713222	-1.563819	-1.353459
C	-5.595248	-3.732843	-0.414930
H	-6.037468	-3.306148	-1.326185
H	-5.138261	-4.702286	-0.640077
H	-6.394369	-3.859249	0.328949
C	-4.933117	-1.636949	0.436302
O	-4.562746	-2.904406	0.083166
O	-6.083492	-1.271833	0.297130
C	-3.845965	0.490325	1.335653
O	-4.700070	1.359676	1.156511
C	-3.805089	-0.895252	0.947060
C	-1.828417	-0.460454	2.162873
C	-0.610848	-0.481199	2.845353
C	-2.540469	0.736411	2.036683
C	-0.131169	0.709869	3.396628
H	-0.052185	-1.412354	2.967598
C	-2.046631	1.935617	2.550870
C	-0.831416	1.915505	3.237900
H	0.799745	0.698227	3.966904
H	-2.620320	2.857093	2.431528
H	-0.428854	2.834106	3.669935
C	-2.602792	-1.592814	1.540971
H	-1.997749	-2.150632	0.810103
H	-2.888928	-2.324128	2.317740

PCMopt/16_TS_SSe

Frequencies, energies and thermodynamic properties:

Lowest Vibrational Mode (1/cm) = -207.1501

2nd Lowest Vibrational Mode (1/cm) = 15.4681

E(RM062X) (a.u.) = -2420.01096777

Thermal correction to Enthalpy (a.u.) = 0.633162

Thermal correction to Gibbs Free Energy (a.u.) = 0.523053

Total Entropy (cal/Kmol) = 231.744

Esp(RM062X+D3) (a.u.) = -2422.42390223

Esp(D3) (a.u.) = -0.0089931834

Esp(RM062X) (a.u.) = -2422.4149090466

Optimised cartesian coordinates (Angstrom):

C	7.203750	-1.253118	0.542864
C	7.076366	-2.454336	-0.169348
C	5.848349	-2.848694	-0.691475
C	4.751732	-2.012557	-0.484801
C	4.880562	-0.822251	0.232960
H	7.950718	-3.087680	-0.319844
H	5.743877	-3.780437	-1.247689
C	2.645437	-0.734483	-0.306305
C	1.146443	1.117374	0.270125
C	2.049315	1.236451	1.508964
C	3.505755	1.090619	1.105777
H	4.147796	1.002327	1.991771
S	3.097973	-2.263380	-1.011605
N	3.662240	-0.140822	0.329806
N	1.432494	-0.169836	-0.404995
C	1.782763	2.502900	2.354324
C	2.614734	3.723245	1.947999
C	1.994313	2.193589	3.837726
H	0.718055	2.754123	2.217516
H	3.676365	3.578035	2.201971
H	2.544738	3.951293	0.875242
H	2.266839	4.605882	2.502733
H	1.323438	1.392273	4.177831
H	3.031294	1.875689	4.029710
H	1.801929	3.086286	4.449260
H	1.785805	0.361646	2.126084
H	0.123560	1.010683	0.652046

C	1.220977	2.285554	-0.688921
C	0.278495	3.314244	-0.578698
C	2.233024	2.388935	-1.649086
C	0.366583	4.444805	-1.389048
H	-0.531109	3.230488	0.150797
C	2.321513	3.519820	-2.461201
H	2.959166	1.582407	-1.778581
C	1.393804	4.553086	-2.327297
H	-0.372675	5.240807	-1.290879
H	3.117035	3.590522	-3.204081
H	1.463975	5.436665	-2.963030
C	-0.860232	-0.172742	-1.290569
H	-0.878190	0.915698	-1.311087
C	-2.018352	-0.893982	-1.413611
C	-3.190135	-0.252352	-2.099921
F	-2.937719	-0.131124	-3.413219
F	-4.314799	-0.960072	-2.003545
F	-3.443514	0.979908	-1.648653
O	0.552609	-2.067323	-1.310836
C	0.358138	-0.896824	-1.028850
H	3.859305	1.939394	0.501407
C	6.110530	-0.419301	0.755195
H	6.219038	0.517211	1.301031
H	8.177328	-0.961685	0.937327
H	-1.982929	-1.982192	-1.505016
C	-0.344665	-3.688166	1.332677
H	0.463721	-3.348957	0.670022
H	-0.073487	-3.466545	2.373929
H	-0.505307	-4.764250	1.209102
C	-1.618094	-1.727658	1.139898
O	-1.569322	-3.062436	0.985351
O	-0.634896	-1.095472	1.512245
C	-4.135919	-1.786563	0.587434
O	-4.376528	-2.953973	0.310430
C	-2.856221	-1.103025	0.756348
C	-4.579747	0.480791	1.136201
C	-5.369880	1.607913	1.355410

C -5.181676 -0.732303 0.794216

C -6.757306 1.490343 1.233095

H -4.918303 2.566528 1.619125

C -6.563038 -0.859252 0.669398

C -7.353249 0.267674 0.894492

H -7.388266 2.364567 1.403721

H -7.000858 -1.822435 0.399069

H -8.438932 0.203888 0.804980

C -3.079737 0.321604 1.210948

H -2.717680 0.478520 2.241375

H -2.553551 1.065107 0.585887

PCMopt/16_TS_SSf

Frequencies, energies and thermodynamic properties:

Lowest Vibrational Mode (1/cm) = -190.2541

2nd Lowest Vibrational Mode (1/cm) = 16.0223

E(RM062X) (a.u.) = -2420.00674455

Thermal correction to Enthalpy (a.u.) = 0.633041

Thermal correction to Gibbs Free Energy (a.u.) = 0.523595

Total Entropy (cal/Kmol) = 230.347

Esp(RM062X+D3) (a.u.) = -2422.42190541

Esp(D3) (a.u.) = -0.0092633757

Esp(RM062X) (a.u.) = -2422.4126420343

Optimised cartesian coordinates (Angstrom):

C 7.241062 -0.942976 0.538187

C 7.177291 -2.185647 -0.109065

C 5.969881 -2.678773 -0.592738

C 4.827886 -1.898622 -0.412762

C 4.893425 -0.667184 0.240919

H 8.086144 -2.773351 -0.238623

H 5.915135 -3.642860 -1.098579

C 2.652172 -0.743021 -0.267553

C 1.044904 1.045564 0.235965

C 1.969478 1.312677 1.436501

C 3.422224 1.220055 1.005963

H 4.085270 1.236839 1.880329

S 3.188511 -2.270997 -0.907946

N	3.636795	-0.055292	0.319273
N	1.398868	-0.262191	-0.366798
C	1.649202	2.623866	2.191960
C	2.397870	3.855019	1.671708
C	1.917551	2.441021	3.687171
H	0.568420	2.802513	2.069196
H	3.471990	3.789878	1.905268
H	2.288394	3.998609	0.587803
H	2.012125	4.754973	2.170604
H	1.304100	1.631945	4.107820
H	2.976173	2.198702	3.870749
H	1.688997	3.366005	4.234669
H	1.783425	0.480912	2.135070
H	0.035726	0.904958	0.646135
C	1.021367	2.155075	-0.794056
C	0.019018	3.128713	-0.718948
C	2.001660	2.258929	-1.785917
C	0.017092	4.210073	-1.598022
H	-0.767531	3.041291	0.035239
C	2.000103	3.341395	-2.666239
H	2.772931	1.491329	-1.888333
C	1.013451	4.322556	-2.568646
H	-0.767971	4.963913	-1.527582
H	2.771596	3.414249	-3.433720
H	1.013316	5.167986	-3.257803
C	-0.868351	-0.395386	-1.303632
H	-0.921498	0.689125	-1.376625
C	-1.998959	-1.150607	-1.425380
C	-3.184145	-0.526694	-2.106830
F	-2.940708	-0.403331	-3.421462
F	-4.300247	-1.243178	-2.000850
F	-3.440347	0.703476	-1.651560
O	0.633769	-2.222993	-1.238817
C	0.378664	-1.057746	-0.998154
H	3.712203	2.039035	0.331049
C	6.103240	-0.164840	0.723246
H	6.162763	0.803850	1.217865

H	8.200094	-0.574586	0.902730
H	-1.952250	-2.241152	-1.469265
C	0.418288	-2.342091	1.948668
H	0.845864	-2.857959	1.076184
H	1.160235	-1.671185	2.397725
H	0.111267	-3.097353	2.684158
C	-1.778126	-2.220594	1.108228
O	-0.684163	-1.536745	1.564718
O	-1.736325	-3.428540	0.976178
C	-4.228150	-1.819234	0.641983
O	-4.638985	-2.947883	0.401949
C	-2.866459	-1.328806	0.794024
C	-4.337453	0.485568	1.196464
C	-4.952558	1.713890	1.428663
C	-5.110355	-0.623323	0.845960
C	-6.342841	1.802572	1.307833
H	-4.365080	2.593508	1.700412
C	-6.495040	-0.545135	0.723304
C	-7.111479	0.684266	0.958326
H	-6.838508	2.758302	1.487866
H	-7.069479	-1.431877	0.447067
H	-8.194786	0.781347	0.869445
C	-2.874678	0.109281	1.253372
H	-2.266497	0.771444	0.608019
H	-2.474457	0.225469	2.274569

PCMopt/9_TS_RRa

Frequencies, energies and thermodynamic properties:

Lowest Vibrational Mode (1/cm) = -114.5289

2nd Lowest Vibrational Mode (1/cm) = 22.5235

E(RM062X) (a.u.) = -2267.73676118

Thermal correction to Enthalpy (a.u.) = 0.606596

Thermal correction to Gibbs Free Energy (a.u.) = 0.504423

Total Entropy (cal/Kmol) = 215.042

Esp(RM062X+D3) (a.u.) = -2269.99026814

Esp(D3) (a.u.) = -0.0085298397

Esp(RM062X) (a.u.) = -2269.9817383003

Optimised cartesian coordinates (Angstrom):

C	-6.126170	-2.092530	0.990029
C	-5.627385	-3.397833	0.872170
C	-4.340027	-3.631203	0.398571
C	-3.563077	-2.528012	0.046111
C	-4.067555	-1.230950	0.154599
H	-6.254133	-4.242662	1.158035
H	-3.946849	-4.643881	0.307814
C	-1.952224	-0.784431	-0.630235
C	-1.022652	1.464241	-0.891545
C	-2.474383	1.853298	-1.233883
C	-3.432031	1.156091	-0.283728
H	-4.468244	1.270220	-0.628277
S	-1.926483	-2.524746	-0.582690
N	-3.132816	-0.275847	-0.258837
N	-0.907528	-0.010025	-0.960618
C	-2.716383	3.379696	-1.307212
C	-3.145450	4.020275	0.016929
C	-3.746164	3.691871	-2.395131
H	-1.761698	3.837128	-1.613989
H	-4.166452	3.711344	0.290296
H	-2.474456	3.772752	0.851297
H	-3.156555	5.113743	-0.092687
H	-3.408213	3.339573	-3.379762
H	-4.711378	3.210380	-2.172432
H	-3.922154	4.774774	-2.459272
H	-2.653827	1.440880	-2.240124
H	-0.398029	1.850637	-1.706918
C	-0.528987	2.029744	0.423193
C	0.161169	3.247065	0.418549
C	-0.818993	1.412634	1.643681
C	0.509905	3.867402	1.617131
H	0.419143	3.721283	-0.531836
C	-0.464976	2.030132	2.842747
H	-1.285704	0.426213	1.667811
C	0.186084	3.264229	2.833193
H	1.040778	4.820292	1.600325

H	-0.695037	1.540695	3.790019
H	0.458101	3.746363	3.773307
C	1.531478	0.179087	-1.299679
H	1.523062	1.215220	-0.969653
C	2.699360	-0.473425	-1.510791
C	3.977842	0.274352	-1.723153
F	4.079853	0.654800	-3.008511
F	4.058244	1.389402	-0.992009
F	5.057579	-0.462387	-1.465959
O	0.308861	-1.810688	-1.643663
C	0.331715	-0.637523	-1.324446
H	-3.359073	1.547308	0.741847
C	-5.356035	-0.990068	0.634164
H	-5.745823	0.021983	0.736816
H	-7.135613	-1.934043	1.369858
H	2.703656	-1.498201	-1.883366
C	3.751622	1.848441	2.044220
H	4.462693	2.236047	1.301335
H	2.962063	2.584971	2.229350
H	4.299845	1.642592	2.974633
C	3.917782	-0.355784	1.274357
O	3.106458	0.686239	1.563160
O	5.123772	-0.275884	1.452001
C	4.028518	-2.780531	0.486999
C	1.854653	-1.830720	0.935235
C	2.952985	-3.779054	0.019189
H	4.820906	-2.631540	-0.262232
C	1.686449	-3.345029	0.757415
H	3.232796	-4.825807	0.200983
H	1.642153	-3.792450	1.766695
C	3.239730	-1.523331	0.760125
O	0.907681	-1.066175	1.158829
H	4.530024	-3.145661	1.403691
H	2.792131	-3.664798	-1.064775
H	0.745950	-3.581376	0.243296

PCMopt/9_TS_RRb

Frequencies, energies and thermodynamic properties:

Lowest Vibrational Mode (1/cm) = -137.8402

2nd Lowest Vibrational Mode (1/cm) = 27.6133

E(RM062X) (a.u.) = -2267.73622347

Thermal correction to Enthalpy (a.u.) = 0.606491

Thermal correction to Gibbs Free Energy (a.u.) = 0.504522

Total Entropy (cal/Kmol) = 214.611

Esp(RM062X+D3) (a.u.) = -2269.98877910

Esp(D3) (a.u.) = -0.0083123461

Esp(RM062X) (a.u.) = -2269.9804667539

Optimised cartesian coordinates (Angstrom):

C	-6.126234	-2.170965	1.019341
C	-5.598138	-3.463754	0.893245
C	-4.309035	-3.665559	0.409448
C	-3.559722	-2.543755	0.056366
C	-4.093424	-1.258863	0.173906
H	-6.203075	-4.323957	1.180258
H	-3.893425	-4.668623	0.311933
C	-1.994964	-0.761657	-0.624361
C	-1.108965	1.505365	-0.847593
C	-2.565622	1.875917	-1.188359
C	-3.515346	1.144923	-0.254934
H	-4.551246	1.239252	-0.606679
S	-1.929704	-2.500726	-0.588386
N	-3.184198	-0.280578	-0.242210
N	-0.968285	0.035960	-0.949452
C	-2.829200	3.399688	-1.235327
C	-3.263246	4.012908	0.099925
C	-3.864530	3.716994	-2.316408
H	-1.880584	3.874331	-1.535407
H	-4.278259	3.683975	0.372219
H	-2.584901	3.762945	0.927607
H	-3.291635	5.107618	0.007091
H	-3.524821	3.383407	-3.306932
H	-4.823949	3.221665	-2.099210
H	-4.052526	4.798749	-2.363892
H	-2.733910	1.479465	-2.202885

H	-0.486307	1.919429	-1.650749
C	-0.627334	2.051567	0.479898
C	0.078388	3.259454	0.495918
C	-0.931008	1.421545	1.690909
C	0.441873	3.851359	1.704493
H	0.344302	3.744749	-0.446765
C	-0.571039	2.015319	2.900100
H	-1.418136	0.444507	1.700815
C	0.106639	3.235112	2.910309
H	0.990085	4.794345	1.703219
H	-0.813558	1.516443	3.839299
H	0.387855	3.696475	3.857954
C	1.468335	0.256391	-1.276350
H	1.450831	1.276168	-0.898894
C	2.642797	-0.385172	-1.503090
C	3.915194	0.376205	-1.711634
F	3.986867	0.802641	-2.984935
F	4.009992	1.463777	-0.943149
F	5.004419	-0.363529	-1.506033
O	0.255965	-1.719017	-1.731791
C	0.276344	-0.564802	-1.347903
H	-3.460613	1.526152	0.775570
C	-5.383857	-1.049692	0.662571
H	-5.796177	-0.047127	0.770637
H	-7.136636	-2.037149	1.406068
H	2.646308	-1.390812	-1.925015
C	5.573253	1.010874	1.834826
H	5.234734	1.227101	2.858095
H	6.659998	0.873321	1.823843
H	5.294791	1.856852	1.191010
C	3.648561	-0.220415	1.279098
O	5.008905	-0.191168	1.351591
O	2.988909	0.728636	1.659543
C	4.058071	-2.650831	0.428549
C	1.809091	-1.876009	0.842179
C	3.070818	-3.703537	-0.113492
H	4.866576	-2.429312	-0.282484

C 1.744922 -3.384569 0.580193

H 3.415213 -4.733780 0.050265

H 1.675977 -3.888006 1.561010

C 3.172000 -1.460132 0.709896

O 0.816658 -1.184041 1.092663

H 4.544127 -3.014492 1.353591

H 2.951951 -3.568023 -1.200352

H 0.846932 -3.646467 0.005274

PCMopt/9_TS_RRc

Frequencies, energies and thermodynamic properties:

Lowest Vibrational Mode (1/cm) = -144.6298

2nd Lowest Vibrational Mode (1/cm) = 23.7695

E(RM062X) (a.u.) = -2267.73354867

Thermal correction to Enthalpy (a.u.) = 0.606470

Thermal correction to Gibbs Free Energy (a.u.) = 0.503394

Total Entropy (cal/Kmol) = 216.942

Esp(RM062X+D3) (a.u.) = -2269.98751993

Esp(D3) (a.u.) = -0.0086439469

Esp(RM062X) (a.u.) = -2269.9788759831

Optimised cartesian coordinates (Angstrom):

C -6.159311 -1.951090 1.081109

C -5.741696 -3.257001 0.785714

C -4.503806 -3.495582 0.197564

C -3.694317 -2.396545 -0.089645

C -4.117951 -1.097797 0.196150

H -6.393260 -4.098208 1.022620

H -4.172341 -4.508796 -0.030532

C -2.037813 -0.666631 -0.688121

C -1.032071 1.564937 -0.848793

C -2.491185 2.029524 -1.010139

C -3.379705 1.289995 -0.027003

H -4.438297 1.486321 -0.240353

S -2.102437 -2.400070 -0.821195

N -3.160504 -0.148398 -0.176609

N -0.980478 0.092497 -1.022897

C -2.671676 3.564188 -0.938390

C	-2.929687	4.107838	0.470660
C	-3.796854	3.999353	-1.879567
H	-1.735705	4.011219	-1.310917
H	-3.927246	3.810534	0.830055
H	-2.183473	3.771354	1.203730
H	-2.908169	5.206390	0.448188
H	-3.580352	3.718897	-2.919879
H	-4.752174	3.532997	-1.591449
H	-3.933536	5.088995	-1.838348
H	-2.788394	1.707923	-2.021674
H	-0.484368	1.984983	-1.701811
C	-0.371940	2.020182	0.435552
C	0.384207	3.197518	0.428046
C	-0.547298	1.325962	1.635394
C	0.925201	3.697264	1.611183
H	0.552245	3.730015	-0.511798
C	-0.014117	1.831606	2.820683
H	-1.068780	0.367073	1.649427
C	0.715628	3.020030	2.813352
H	1.512487	4.616341	1.593396
H	-0.156677	1.283209	3.752916
H	1.135874	3.409718	3.741675
C	1.441719	0.236787	-1.438303
H	1.420395	1.309141	-1.267543
C	2.639020	-0.401466	-1.491628
C	3.864927	0.416165	-1.772745
F	4.045593	0.512617	-3.100171
F	3.753005	1.667905	-1.312115
F	4.994366	-0.091173	-1.281913
O	0.153487	-1.704147	-1.852537
C	0.224470	-0.553331	-1.471547
H	-3.173480	1.572846	1.016096
C	-5.356124	-0.852731	0.792649
H	-5.682793	0.157743	1.035282
H	-7.130569	-1.789310	1.548918
H	2.716475	-1.467354	-1.714282
C	1.035960	-3.951991	0.253888

H 0.412627 -3.667101 -0.603693
H 1.433725 -4.962881 0.108240
H 0.419392 -3.919588 1.163247
C 1.917205 -1.831325 0.790615
O 2.154627 -3.094130 0.362003
O 0.771355 -1.440918 1.003914
C 2.945929 0.331170 1.586691
C 4.446089 -1.485722 0.993514
C 4.394017 0.852352 1.705110
H 2.288474 1.021538 1.026467
C 5.272378 -0.401498 1.697745
H 4.541106 1.474332 2.599078
H 5.468115 -0.769404 2.719919
C 3.081943 -1.008747 0.915612
O 4.930719 -2.550571 0.626970
H 2.473885 0.228839 2.581460
H 4.635730 1.476221 0.834476
H 6.247899 -0.278050 1.205650

PCMopt/9_TS_RSa

Frequencies, energies and thermodynamic properties:

Lowest Vibrational Mode (1/cm) = -135.6927
2nd Lowest Vibrational Mode (1/cm) = 20.0079
E(RM062X) (a.u.) = -2267.72907895
Thermal correction to Enthalpy (a.u.) = 0.606457
Thermal correction to Gibbs Free Energy (a.u.) = 0.502126
Total Entropy (cal/Kmol) = 219.583
Esp(RM062X+D3) (a.u.) = -2269.98578474
Esp(D3) (a.u.) = -0.0081704581
Esp(RM062X) (a.u.) = -2269.9776142819

Optimised cartesian coordinates (Angstrom):

C -6.140842 -2.648210 0.785517
C -5.506475 -3.898987 0.810304
C -4.161842 -4.025949 0.477234
C -3.466015 -2.870947 0.120676
C -4.103546 -1.629922 0.087746
H -6.072868 -4.784935 1.097616

H	-3.663728	-4.995347	0.497565
C	-1.973733	-1.012642	-0.520557
C	-1.253459	1.305132	-0.837584
C	-2.688514	1.511561	-1.360706
C	-3.671718	0.776348	-0.468249
H	-4.667293	0.748888	-0.930029
S	-1.785062	-2.735445	-0.355958
N	-3.229418	-0.609008	-0.301627
N	-0.975557	-0.149374	-0.789179
C	-3.072003	2.995071	-1.575883
C	-3.714854	3.668763	-0.359263
C	-3.993750	3.122853	-2.790652
H	-2.138816	3.532522	-1.810705
H	-4.724885	3.269729	-0.177253
H	-3.125357	3.549037	0.560440
H	-3.821879	4.745370	-0.551838
H	-3.508984	2.747287	-3.702659
H	-4.924842	2.554299	-2.639142
H	-4.269189	4.173858	-2.955582
H	-2.708592	1.018403	-2.346101
H	-0.584367	1.711952	-1.605396
C	-0.976843	1.994672	0.481241
C	-0.414990	3.276313	0.468029
C	-1.352942	1.427184	1.703368
C	-0.277187	4.001013	1.651157
H	-0.092045	3.717034	-0.478302
C	-1.217860	2.152902	2.886531
H	-1.744481	0.408319	1.747592
C	-0.691850	3.445160	2.861815
H	0.157607	5.000996	1.626295
H	-1.519170	1.702247	3.833039
H	-0.588622	4.011349	3.788435
C	1.436531	0.282772	-1.074845
H	1.301397	1.307614	-0.731264
C	2.673731	-0.186021	-1.381219
C	3.799268	0.772880	-1.621322
F	3.603047	1.448836	-2.768961

F	3.911015	1.686581	-0.660695
F	4.975617	0.156428	-1.754712
O	0.439611	-1.855490	-1.280859
C	0.338883	-0.665338	-1.060972
H	-3.760274	1.237787	0.526380
C	-5.451510	-1.495464	0.424473
H	-5.947250	-0.525544	0.416156
H	-7.194074	-2.573443	1.056506
H	2.794095	-1.183540	-1.804615
C	2.116345	1.410579	2.647590
H	1.593513	0.863301	3.445989
H	2.646198	2.271387	3.071947
H	1.365056	1.755899	1.924350
C	2.639587	-0.531414	1.441643
O	3.091667	0.606787	2.018637
O	1.452681	-0.836673	1.522322
C	3.362915	-2.671605	0.231791
C	5.054534	-1.015311	0.733812
C	4.617510	-3.002716	-0.596353
H	3.286186	-3.366466	1.090565
C	5.738552	-2.237067	0.104064
H	4.497833	-2.612817	-1.620016
H	6.567485	-1.923608	-0.546184
C	3.625086	-1.255710	0.699486
O	5.689333	-0.065256	1.175274
H	2.433862	-2.774383	-0.343680
H	4.805109	-4.082216	-0.677065
H	6.172684	-2.826153	0.931014

PCMopt/9_TS_RSb

Frequencies, energies and thermodynamic properties:

Lowest Vibrational Mode (1/cm) = -166.1426

2nd Lowest Vibrational Mode (1/cm) = 21.6680

E(RM062X) (a.u.) = -2267.73017307

Thermal correction to Enthalpy (a.u.) = 0.606636

Thermal correction to Gibbs Free Energy (a.u.) = 0.502151

Total Entropy (cal/Kmol) = 219.907

Esp(RM062X+D3) (a.u.) = -2269.98403601

Esp(D3) (a.u.) = -0.0081429491

Esp(RM062X) (a.u.) = -2269.9758930609

Optimised cartesian coordinates (Angstrom):

C	6.196882	2.622942	0.844329
C	5.582029	3.883010	0.815171
C	4.249166	4.019203	0.439951
C	3.545780	2.865465	0.094798
C	4.164424	1.614983	0.115628
H	6.153615	4.768560	1.093378
H	3.765333	4.995923	0.419230
C	2.042323	1.011659	-0.541921
C	1.298488	-1.297088	-0.835332
C	2.752024	-1.547393	-1.282002
C	3.710825	-0.799478	-0.374651
H	4.724426	-0.805065	-0.796590
S	1.872775	2.741987	-0.416135
N	3.286427	0.596641	-0.271355
N	1.044815	0.160458	-0.833806
C	3.112146	-3.044876	-1.428416
C	3.676546	-3.690602	-0.158882
C	4.090975	-3.234136	-2.589261
H	2.179423	-3.569956	-1.692078
H	4.684074	-3.305707	0.063148
H	3.042726	-3.528497	0.724106
H	3.770352	-4.774920	-0.311097
H	3.661653	-2.881023	-3.537316
H	5.025431	-2.679518	-2.409424
H	4.351461	-4.295618	-2.704043
H	2.825864	-1.089991	-2.282086
H	0.668288	-1.713402	-1.629206
C	0.937660	-1.947988	0.483073
C	0.325778	-3.205932	0.473430
C	1.292634	-1.371458	1.707727
C	0.119354	-3.902258	1.664076
H	0.020251	-3.653257	-0.475839
C	1.094442	-2.070808	2.897661

H	1.717741	-0.366089	1.747353
C	0.519687	-3.342382	2.877606
H	-0.353678	-4.884885	1.642124
H	1.383455	-1.615447	3.845730
H	0.366207	-3.887756	3.809721
C	-1.362297	-0.244570	-1.214200
H	-1.211612	-1.319673	-1.146782
C	-2.628363	0.267004	-1.280825
C	-3.746996	-0.543763	-1.854824
F	-3.608359	-0.645999	-3.188576
F	-3.785712	-1.794968	-1.386424
F	-4.945154	0.007222	-1.650120
O	-0.349933	1.895647	-1.287156
C	-0.270631	0.694237	-1.113091
H	3.751477	-1.229932	0.636894
C	5.499661	1.470563	0.496163
H	5.980661	0.493800	0.530059
H	7.240657	2.540024	1.147755
H	-2.744733	1.348812	-1.376189
C	-6.110788	3.068299	0.259389
H	-5.839807	3.345602	-0.769305
H	-7.193065	2.918682	0.333445
H	-5.798084	3.881236	0.929393
C	-4.150702	1.811938	0.584188
O	-5.507829	1.842986	0.627343
O	-3.521978	2.810784	0.282572
C	-4.580900	-0.617160	1.323515
C	-2.345447	0.276088	1.507322
C	-3.644095	-1.766616	1.734667
H	-5.199200	-0.291277	2.180043
C	-2.375093	-1.072265	2.225752
H	-3.406563	-2.375740	0.851180
H	-1.445233	-1.621476	2.027586
C	-3.638712	0.493027	0.902540
O	-1.333147	0.967929	1.452360
H	-5.285722	-0.910979	0.532572
H	-4.095069	-2.431077	2.484257

H -2.408102 -0.865773 3.309923

PCMopt/9_TS_RSc

Frequencies, energies and thermodynamic properties:

Lowest Vibrational Mode (1/cm) = -94.2717

2nd Lowest Vibrational Mode (1/cm) = 25.7250

E(RM062X) (a.u.) = -2267.73152342

Thermal correction to Enthalpy (a.u.) = 0.606938

Thermal correction to Gibbs Free Energy (a.u.) = 0.504652

Total Entropy (cal/Kmol) = 215.279

Esp(RM062X+D3) (a.u.) = -2269.98579954

Esp(D3) (a.u.) = -0.0082142749

Esp(RM062X) (a.u.) = -2269.9775852651

Optimised cartesian coordinates (Angstrom):

C -6.290853 -2.094192 0.720829
C -5.813408 -3.395385 0.504213
C -4.513006 -3.615632 0.062719
C -3.701808 -2.502390 -0.157650
C -4.182894 -1.209433 0.050748
H -6.468192 -4.247363 0.687631
H -4.135741 -4.624841 -0.103051
C -2.026635 -0.742985 -0.602658
C -1.069549 1.513240 -0.708228
C -2.512068 1.923570 -1.062755
C -3.488098 1.190338 -0.162221
H -4.519742 1.341541 -0.504639
S -2.041029 -2.479474 -0.713135
N -3.210652 -0.244169 -0.232717
N -0.952238 0.038640 -0.829242
C -2.750417 3.452149 -1.076388
C -3.212850 4.033627 0.263767
C -3.754682 3.810276 -2.174058
H -1.789939 3.922577 -1.341393
H -4.240913 3.714686 0.495938
H -2.564414 3.747635 1.103521
H -3.220141 5.130908 0.203417
H -3.392331 3.501913 -3.164705

H	-4.723946	3.318377	-1.996065
H	-3.931386	4.894719	-2.194725
H	-2.674436	1.554511	-2.088649
H	-0.432731	1.930372	-1.497698
C	-0.597365	2.022028	0.636069
C	0.058635	3.256535	0.699427
C	-0.870174	1.330731	1.819996
C	0.393170	3.816270	1.930797
H	0.304458	3.789171	-0.222562
C	-0.535524	1.891085	3.052802
H	-1.326931	0.338870	1.790806
C	0.085448	3.138872	3.111381
H	0.899471	4.781654	1.967718
H	-0.755955	1.345514	3.971147
H	0.345061	3.575443	4.076710
C	1.480155	0.265998	-1.207752
H	1.450895	1.311954	-0.912157
C	2.661456	-0.321061	-1.503836
C	3.847849	0.521033	-1.859115
F	3.808251	0.827413	-3.167299
F	3.878959	1.679222	-1.197829
F	5.016144	-0.091777	-1.660072
O	0.275214	-1.743894	-1.532162
C	0.291355	-0.575703	-1.201619
H	-3.414900	1.514347	0.886540
C	-5.485618	-0.982397	0.498751
H	-5.859665	0.024517	0.679678
H	-7.311752	-1.947481	1.073719
H	2.708678	-1.362964	-1.826954
C	1.115958	-3.883890	0.775296
H	0.398919	-3.593896	-0.003265
H	1.367180	-4.946002	0.673994
H	0.658427	-3.701114	1.758215
C	2.281217	-1.830690	0.914195
O	2.323722	-3.167799	0.630786
O	1.224549	-1.295777	1.227979
C	4.819033	-2.017566	0.485922

C 3.870759 0.086414 1.279264
C 5.991222 -1.107610 0.943390
H 4.818622 -2.972389 1.034371
C 5.393577 0.272044 1.252227
H 6.779565 -1.052543 0.180581
H 5.630047 1.024204 0.483803
C 3.567660 -1.221589 0.765028
O 3.109816 0.962567 1.682458
H 4.913890 -2.283495 -0.580888
H 6.453502 -1.528147 1.847928
H 5.719222 0.690986 2.215966

PCMopt/9_TS_RSd

Frequencies, energies and thermodynamic properties:

Lowest Vibrational Mode (1/cm) = -137.8400
2nd Lowest Vibrational Mode (1/cm) = 15.4067
E(RM062X) (a.u.) = -2267.72418929
Thermal correction to Enthalpy (a.u.) = 0.606374
Thermal correction to Gibbs Free Energy (a.u.) = 0.500402
Total Entropy (cal/Kmol) = 223.036
Esp(RM062X+D3) (a.u.) = -2269.98111292
Esp(D3) (a.u.) = -0.0082574662
Esp(RM062X) (a.u.) = -2269.9728554538

Optimised cartesian coordinates (Angstrom):

C -6.194196 -2.682355 0.765925
C -5.566203 -3.934026 0.683965
C -4.235369 -4.042317 0.294567
C -3.547557 -2.867845 -0.010607
C -4.178721 -1.625354 0.062309
H -6.126603 -4.835516 0.931932
H -3.741777 -5.012262 0.232824
C -2.070046 -0.977074 -0.585494
C -1.350735 1.357517 -0.826988
C -2.813950 1.594436 -1.242186
C -3.746214 0.814070 -0.334380
H -4.770256 0.828026 -0.729368
S -1.881553 -2.706717 -0.528546

N	-3.313014	-0.583632	-0.290175
N	-1.077545	-0.101804	-0.842747
C	-3.193704	3.090370	-1.348856
C	-3.726748	3.704624	-0.050702
C	-4.208022	3.290208	-2.476896
H	-2.274326	3.630130	-1.629415
H	-4.720570	3.300228	0.196880
H	-3.061950	3.537865	0.808117
H	-3.842089	4.789663	-0.180825
H	-3.803148	2.959377	-3.443478
H	-5.131326	2.722718	-2.280620
H	-4.482261	4.350787	-2.563750
H	-2.901407	1.158336	-2.250616
H	-0.739696	1.784128	-1.631540
C	-0.953731	1.998013	0.486535
C	-0.313208	3.242014	0.468455
C	-1.262765	1.407622	1.715997
C	-0.013992	3.900859	1.659659
H	-0.048841	3.702411	-0.487133
C	-0.959767	2.064288	2.908529
H	-1.733118	0.421588	1.758933
C	-0.342625	3.314925	2.882417
H	0.482631	4.871519	1.631784
H	-1.203623	1.592647	3.861351
H	-0.106232	3.827071	3.816054
C	1.330654	0.356449	-1.079246
H	1.133016	1.412719	-0.915351
C	2.617979	-0.060756	-1.191249
C	3.646757	0.991957	-1.498698
F	3.497952	1.413397	-2.766455
F	3.507188	2.073668	-0.724639
F	4.903275	0.572022	-1.393309
O	0.359361	-1.786245	-1.371090
C	0.243756	-0.607111	-1.122168
H	-3.761877	1.211115	0.691379
C	-5.512654	-1.510061	0.457488
H	-6.003918	-0.540777	0.532552

H -7.236366 -2.622687 1.079940
H 2.883241 -1.100434 -1.395910
C 5.959070 -2.815990 -0.953442
H 6.656041 -3.195550 -0.193341
H 5.630865 -3.635690 -1.601718
H 6.481841 -2.053956 -1.549556
C 4.997113 -1.257381 0.513466
O 4.794132 -2.279699 -0.359217
O 6.128591 -0.865128 0.752760
C 3.847719 0.534653 1.920908
C 2.563765 -1.411196 1.278100
C 2.362368 0.845874 2.197095
H 4.387794 0.330185 2.865849
C 1.660186 -0.512356 2.128528
H 1.973200 1.502311 1.403034
H 0.651414 -0.478031 1.685523
C 3.785093 -0.692930 1.045807
O 2.217644 -2.532755 0.914213
H 4.369980 1.378686 1.444938
H 2.205318 1.366245 3.152139
H 1.553719 -0.985249 3.119870

PCMopt/9_TS_RSe

Frequencies, energies and thermodynamic properties:

Lowest Vibrational Mode (1/cm) = -53.0749

2nd Lowest Vibrational Mode (1/cm) = 25.4660

E(RM062X) (a.u.) = -2267.72832192

Thermal correction to Enthalpy (a.u.) = 0.606431

Thermal correction to Gibbs Free Energy (a.u.) = 0.503724

Total Entropy (cal/Kmol) = 216.165

Esp(RM062X+D3) (a.u.) = -2269.98302236

Esp(D3) (a.u.) = -0.0083855496

Esp(RM062X) (a.u.) = -2269.9746368104

Optimised cartesian coordinates (Angstrom):

C -6.526153 -1.734921 0.573094
C -6.139881 -3.077769 0.443022
C -4.836983 -3.416769 0.095622

C	-3.929123	-2.379312	-0.120943
C	-4.319558	-1.045530	-0.000061
H	-6.869837	-3.867769	0.619829
H	-4.530746	-4.458291	-0.003036
C	-2.102636	-0.774283	-0.547711
C	-0.974405	1.401999	-0.702785
C	-2.362017	1.908339	-1.136776
C	-3.435413	1.283181	-0.267653
H	-4.433692	1.492896	-0.672158
S	-2.239687	-2.509444	-0.561219
N	-3.261413	-0.170404	-0.268079
N	-0.961768	-0.081978	-0.768456
C	-2.468013	3.450405	-1.203085
C	-2.924961	4.111890	0.101082
C	-3.394585	3.859584	-2.349915
H	-1.459636	3.826514	-1.440965
H	-3.983481	3.888160	0.306148
H	-2.329891	3.800474	0.970754
H	-2.837487	5.203342	0.007037
H	-3.024702	3.488315	-3.315931
H	-4.410029	3.461475	-2.196494
H	-3.472640	4.954135	-2.409297
H	-2.505111	1.522546	-2.159251
H	-0.280998	1.744441	-1.478066
C	-0.511058	1.910010	0.644195
C	0.292871	3.050757	0.697426
C	-0.932124	1.313684	1.836579
C	0.638981	3.614191	1.922645
H	0.651146	3.505637	-0.229552
C	-0.582766	1.874459	3.064300
H	-1.534468	0.401379	1.823304
C	0.194158	3.032093	3.109033
H	1.265213	4.507005	1.951146
H	-0.919498	1.403624	3.988893
H	0.463969	3.471910	4.070291
C	1.474967	-0.035497	-1.240104
H	1.523374	1.040475	-1.096290

C	2.583819	-0.735874	-1.548018
C	3.795190	-0.060231	-2.107603
F	3.607956	0.206203	-3.412722
F	4.069019	1.108087	-1.523732
F	4.885156	-0.825170	-2.039924
O	0.164074	-2.002856	-1.199327
C	0.240024	-0.799797	-1.060933
H	-3.395039	1.641780	0.771510
C	-5.625023	-0.699189	0.354874
H	-5.929379	0.340578	0.467035
H	-7.551996	-1.495001	0.853075
H	2.547782	-1.818913	-1.679403
C	0.664294	-2.784846	1.655287
H	1.011676	-3.395698	2.501411
H	-0.285276	-2.299189	1.913494
H	0.518821	-3.439455	0.785130
C	2.838109	-2.151587	1.049290
O	1.579183	-1.747997	1.368534
O	3.096329	-3.347878	0.970145
C	5.256328	-1.421139	0.831428
C	3.571064	0.293451	1.165133
C	5.953472	-0.045049	0.751644
H	5.558067	-1.965570	1.746979
C	4.948495	0.952994	1.332208
H	6.148073	0.203171	-0.300381
H	4.964618	1.944784	0.857749
C	3.783723	-1.089782	0.864815
O	2.517445	0.920792	1.293075
H	5.536754	-2.067931	-0.014537
H	6.920626	-0.035905	1.273501
H	5.100829	1.107533	2.414762

PCMopt/9_TS_SRa

Frequencies, energies and thermodynamic properties:

Lowest Vibrational Mode (1/cm) = -260.6584

2nd Lowest Vibrational Mode (1/cm) = 21.6558

E(RM062X) (a.u.) = -2267.74320818

Thermal correction to Enthalpy (a.u.) = 0.607190

Thermal correction to Gibbs Free Energy (a.u.) = 0.504287

Total Entropy (cal/Kmol) = 216.577

Esp(RM062X+D3) (a.u.) = -2269.99037720

Esp(D3) (a.u.) = -0.0084854507

Esp(RM062X) (a.u.) = -2269.9818917493

Optimised cartesian coordinates (Angstrom):

C	6.677077	-1.100593	0.176128
C	6.567545	-2.261430	-0.601538
C	5.332776	-2.680199	-1.089637
C	4.212351	-1.909335	-0.784155
C	4.324029	-0.758210	-0.001371
H	7.459826	-2.844111	-0.830934
H	5.242610	-3.581977	-1.695699
C	2.063622	-0.736746	-0.436827
C	0.525770	1.016603	0.297740
C	1.461684	1.090325	1.513254
C	2.911232	1.026869	1.064334
H	3.582994	0.895038	1.922991
S	2.544666	-2.208409	-1.243480
N	3.085220	-0.138269	0.193132
N	0.833392	-0.220563	-0.460035
C	1.161198	2.284177	2.446512
C	1.918925	3.568809	2.098313
C	1.431708	1.893067	3.900691
H	0.080813	2.484127	2.350759
H	2.994880	3.460300	2.307710
H	1.800191	3.862130	1.045802
H	1.549407	4.395167	2.721879
H	0.812045	1.037702	4.204871
H	2.488626	1.617299	4.043715
H	1.213356	2.733163	4.575163
H	1.246976	0.167168	2.076233
H	-0.484581	0.866517	0.701592
C	0.530223	2.242058	-0.588172
C	-0.489582	3.186282	-0.415902
C	1.534313	2.482518	-1.532205

C	-0.476714	4.375860	-1.143338
H	-1.299844	2.964927	0.284733
C	1.544667	3.671688	-2.261733
H	2.315391	1.739214	-1.712986
C	0.545930	4.625181	-2.060121
H	-1.272673	5.108200	-0.999965
H	2.334557	3.852037	-2.992227
H	0.556440	5.555729	-2.629464
C	-1.485379	-0.263205	-1.206209
H	-1.502366	0.823227	-1.181659
C	-2.678700	-0.953426	-1.177695
C	-3.861545	-0.273074	-1.807679
F	-3.821120	-0.430485	-3.139276
F	-5.047272	-0.746101	-1.414866
F	-3.871219	1.044575	-1.577492
O	-0.053659	-2.150190	-1.316575
C	-0.260852	-0.980324	-1.028121
H	3.218588	1.930880	0.517522
C	5.559249	-0.332334	0.488427
H	5.651683	0.574001	1.085877
H	7.654873	-0.788112	0.543238
H	-2.684759	-2.040829	-1.294826
C	-0.932495	-3.845982	1.467015
H	-0.512216	-3.616334	2.455613
H	-1.183094	-4.909835	1.404150
H	-0.196972	-3.579260	0.694384
C	-2.050866	-1.789460	1.295299
O	-2.143114	-3.136827	1.254432
O	-0.988303	-1.239992	1.528687
C	-4.613662	-1.877056	1.106131
C	-3.474285	0.281457	1.206744
C	-5.676984	-0.783252	1.403074
H	-4.869958	-2.459154	0.207796
C	-4.967768	0.578228	1.321791
H	-6.096612	-0.935098	2.406712
H	-5.135234	1.219370	2.199421
C	-3.301134	-1.138664	0.955612

O	-2.604466	1.144943	1.262536
H	-4.551711	-2.603027	1.930730
H	-6.512908	-0.841924	0.694886
H	-5.266431	1.165481	0.439858

PCMopt/9_TS_SRb

Frequencies, energies and thermodynamic properties:

Lowest Vibrational Mode (1/cm) =	-212.5120
2nd Lowest Vibrational Mode (1/cm) =	23.8449
E(RM062X) (a.u.) =	-2267.74116899
Thermal correction to Enthalpy (a.u.) =	0.606915
Thermal correction to Gibbs Free Energy (a.u.) =	0.504900
Total Entropy (cal/Kmol) =	214.709
Esp(RM062X+D3) (a.u.) =	-2269.99033473
Esp(D3) (a.u.) =	-0.0085946364
Esp(RM062X) (a.u.) =	-2269.9817400936

Optimised cartesian coordinates (Angstrom):

C	6.688960	-1.095555	0.114612
C	6.560459	-2.307288	-0.578882
C	5.314367	-2.759036	-1.003052
C	4.200953	-1.969817	-0.717664
C	4.331660	-0.768479	-0.018303
H	7.447600	-2.903565	-0.792627
H	5.209372	-3.699330	-1.544675
C	2.060734	-0.781317	-0.387784
C	0.526381	1.005329	0.296651
C	1.523699	1.193413	1.450858
C	2.949213	1.100858	0.935360
H	3.663850	1.055808	1.767325
S	2.524066	-2.295898	-1.116983
N	3.096202	-0.138162	0.167377
N	0.820606	-0.274870	-0.391523
C	1.259900	2.464097	2.291202
C	1.990367	3.715924	1.795646
C	1.607758	2.202950	3.758186
H	0.174918	2.651708	2.234148
H	3.075953	3.628372	1.959464

H	1.817368	3.919440	0.729772
H	1.646568	4.590699	2.365386
H	1.012418	1.375420	4.169063
H	2.673138	1.946438	3.870867
H	1.416007	3.098565	4.365748
H	1.362367	0.328525	2.114789
H	-0.464530	0.865049	0.752939
C	0.467225	2.166628	-0.671275
C	-0.543968	3.120487	-0.503175
C	1.411377	2.339690	-1.688401
C	-0.580250	4.255906	-1.311386
H	-1.308885	2.948577	0.259169
C	1.372864	3.475136	-2.498429
H	2.183049	1.585533	-1.864202
C	0.384331	4.440295	-2.303631
H	-1.369053	4.996493	-1.171360
H	2.116413	3.603650	-3.286192
H	0.356426	5.328738	-2.936079
C	-1.507888	-0.333083	-1.152309
H	-1.549009	0.753252	-1.156912
C	-2.664734	-1.068485	-1.161515
C	-3.885257	-0.484758	-1.808081
F	-3.691428	-0.341997	-3.128388
F	-4.962984	-1.261717	-1.674726
F	-4.196353	0.729342	-1.341361
O	-0.059464	-2.205735	-1.227795
C	-0.268920	-1.034040	-0.955957
H	3.216312	1.952369	0.291920
C	5.579246	-0.308322	0.405519
H	5.687563	0.637194	0.935595
H	7.676024	-0.758282	0.431421
H	-2.612596	-2.158393	-1.222860
C	-0.090861	-2.413484	1.910780
H	0.165533	-3.037257	1.041900
H	0.770163	-1.798351	2.198109
H	-0.377759	-3.063997	2.746863
C	-2.346223	-2.092443	1.362464

O -1.140742 -1.516318 1.583419
O -2.482520 -3.300912 1.441011
C -4.830505 -1.684931 1.195256
C -3.379748 0.250547 1.315620
C -5.716286 -0.421844 1.151726
H -5.117526 -2.419514 0.428874
C -4.804414 0.726367 1.591837
H -6.608108 -0.523082 1.784295
H -4.867794 0.908147 2.678395
C -3.415283 -1.168720 1.028400
O -2.407976 1.003919 1.345865
H -4.926028 -2.199794 2.168301
H -6.062704 -0.246229 0.125475
H -4.998193 1.684938 1.090480

PCMopt/9_TS_SRc

Frequencies, energies and thermodynamic properties:

Lowest Vibrational Mode (1/cm) = -212.6060
2nd Lowest Vibrational Mode (1/cm) = 23.8662
E(RM062X) (a.u.) = -2267.74116897
Thermal correction to Enthalpy (a.u.) = 0.606916
Thermal correction to Gibbs Free Energy (a.u.) = 0.504909
Total Entropy (cal/Kmol) = 214.691
Esp(RM062X+D3) (a.u.) = -2269.99033153
Esp(D3) (a.u.) = -0.0085948276
Esp(RM062X) (a.u.) = -2269.9817367024

Optimised cartesian coordinates (Angstrom):

C 6.688857 -1.095446 0.114729
C 6.560391 -2.307201 -0.578733
C 5.314318 -2.758968 -1.002938
C 4.200887 -1.969750 -0.717615
C 4.331560 -0.768387 -0.018289
H 7.447544 -2.903479 -0.792425
H 5.209350 -3.699279 -1.544538
C 2.060647 -0.781248 -0.387861
C 0.526217 1.005332 0.296583
C 1.523538 1.193458 1.450779

C	2.949057	1.100955	0.935286
H	3.663684	1.055922	1.767262
S	2.524015	-2.295856	-1.116990
N	3.096093	-0.138069	0.167318
N	0.820521	-0.274819	-0.391647
C	1.259683	2.464143	2.291100
C	1.990171	3.715971	1.795568
C	1.607454	2.203038	3.758110
H	0.174705	2.651761	2.234003
H	3.075695	3.628624	1.959912
H	1.817638	3.919194	0.729562
H	1.645963	4.590840	2.364917
H	1.012373	1.375259	4.168862
H	2.672919	1.946936	3.870913
H	1.415275	3.098547	4.365695
H	1.362252	0.328572	2.114722
H	-0.464649	0.864898	0.752923
C	0.466993	2.166653	-0.671305
C	-0.544131	3.120565	-0.503105
C	1.411075	2.339680	-1.688501
C	-0.580405	4.256004	-1.311289
H	-1.309006	2.948674	0.259286
C	1.372569	3.475144	-2.498502
H	2.182688	1.585479	-1.864375
C	0.384111	4.440359	-2.303604
H	-1.369153	4.996635	-1.171188
H	2.116064	3.603629	-3.286321
H	0.356212	5.328820	-2.936026
C	-1.507944	-0.333033	-1.152476
H	-1.549134	0.753299	-1.156950
C	-2.664788	-1.068494	-1.161554
C	-3.885378	-0.484806	-1.808033
F	-3.691703	-0.342284	-3.128398
F	-4.963154	-1.261642	-1.674456
F	-4.196179	0.729417	-1.341454
O	-0.059515	-2.205675	-1.227997
C	-0.268988	-1.033971	-0.956171

H	3.216143	1.952475	0.291854
C	5.579127	-0.308210	0.405566
H	5.687416	0.637326	0.935612
H	7.675907	-0.758157	0.431566
H	-2.612573	-2.158394	-1.223037
C	-0.090255	-2.413341	1.910280
H	0.166137	-3.037017	1.041326
H	0.770724	-1.798108	2.197545
H	-0.376980	-3.063944	2.746351
C	-2.345765	-2.092527	1.362389
O	-1.140288	-1.516297	1.583065
O	-2.481978	-3.300992	1.441073
C	-4.830104	-1.685174	1.195562
C	-3.379474	0.250378	1.315791
C	-5.715968	-0.422151	1.152077
H	-5.117245	-2.419897	0.429358
C	-4.804140	0.726028	1.592327
H	-6.607818	-0.523506	1.784588
H	-4.867341	0.907438	2.678964
C	-3.414945	-1.168887	1.028382
O	-2.407757	1.003808	1.346016
H	-4.925391	-2.199880	2.168714
H	-6.062353	-0.246417	0.125846
H	-4.998177	1.684821	1.091428

PCMopt/9_TS_SRd

Frequencies, energies and thermodynamic properties:

Lowest Vibrational Mode (1/cm) = -159.3395

2nd Lowest Vibrational Mode (1/cm) = 22.5071

E(RM062X) (a.u.) = -2267.73329494

Thermal correction to Enthalpy (a.u.) = 0.606557

Thermal correction to Gibbs Free Energy (a.u.) = 0.502594

Total Entropy (cal/Kmol) = 218.808

Esp(RM062X+D3) (a.u.) = -2269.98657324

Esp(D3) (a.u.) = -0.0085336154

Esp(RM062X) (a.u.) = -2269.9780396246

Optimised cartesian coordinates (Angstrom):

C	6.855076	-1.163322	0.294549
C	6.742879	-2.386489	-0.382351
C	5.506448	-2.850855	-0.819676
C	4.385382	-2.061764	-0.564192
C	4.499620	-0.849297	0.118301
H	7.636035	-2.981868	-0.572147
H	5.414047	-3.799851	-1.348174
C	2.236256	-0.881084	-0.283588
C	0.692278	0.927579	0.323459
C	1.658122	1.131616	1.501696
C	3.095277	1.026752	1.023049
H	3.787669	0.989786	1.873977
S	2.717550	-2.400762	-0.984713
N	3.258124	-0.222882	0.275115
N	0.989853	-0.379843	-0.315729
C	1.376623	2.419987	2.308992
C	2.120366	3.660490	1.804717
C	1.688917	2.188696	3.788849
H	0.293486	2.608202	2.222362
H	3.201327	3.576733	1.998056
H	1.974630	3.841015	0.730634
H	1.762473	4.547328	2.346276
H	1.083732	1.369596	4.202135
H	2.751223	1.935197	3.932431
H	1.482566	3.096667	4.372673
H	1.477358	0.276804	2.174453
H	-0.314669	0.830919	0.754923
C	0.668593	2.060222	-0.679873
C	-0.344278	3.020810	-0.569146
C	1.641850	2.198514	-1.674430
C	-0.355516	4.129540	-1.414231
H	-1.132390	2.875831	0.175633
C	1.628127	3.307843	-2.520607
H	2.416295	1.438347	-1.805874
C	0.636624	4.280023	-2.384586
H	-1.146288	4.875150	-1.319836
H	2.393533	3.409935	-3.291008

H	0.628334	5.147378	-3.046114
C	-1.318935	-0.443064	-1.117061
H	-1.332937	0.643712	-1.153757
C	-2.492180	-1.130149	-1.172990
C	-3.679252	-0.481186	-1.820154
F	-3.619440	-0.668862	-3.151418
F	-4.844111	-0.973565	-1.424010
F	-3.701021	0.843340	-1.628657
O	0.127035	-2.326164	-1.145969
C	-0.081926	-1.155382	-0.887967
H	3.383318	1.867694	0.374856
C	5.738087	-0.376561	0.555467
H	5.833697	0.577039	1.073348
H	7.835544	-0.817333	0.622158
H	-2.506104	-2.221600	-1.191900
C	-4.793943	2.181848	0.891786
H	-4.229071	2.579588	0.037274
H	-5.863759	2.368252	0.749794
H	-4.445488	2.679652	1.807347
C	-3.386918	0.328670	1.174519
O	-4.642235	0.778351	0.984161
O	-2.448681	1.114000	1.300889
C	-1.955185	-1.670587	1.722029
C	-4.273037	-2.106805	1.228239
C	-2.088301	-3.185568	1.498675
H	-1.057237	-1.243553	1.243678
C	-3.595989	-3.445244	1.566597
H	-1.516407	-3.773433	2.229099
H	-3.914862	-3.714377	2.587763
C	-3.229511	-1.100291	1.168546
O	-5.483681	-2.003399	1.089329
H	-1.848643	-1.432289	2.796830
H	-1.695435	-3.440012	0.501868
H	-3.960289	-4.239437	0.898733

PCMopt/9_TS_SRe

Frequencies, energies and thermodynamic properties:

Lowest Vibrational Mode (1/cm) = -95.9008

2nd Lowest Vibrational Mode (1/cm) = 18.5914

E(RM062X) (a.u.) = -2267.72789129

Thermal correction to Enthalpy (a.u.) = 0.606287

Thermal correction to Gibbs Free Energy (a.u.) = 0.500889

Total Entropy (cal/Kmol) = 221.829

Esp(RM062X+D3) (a.u.) = -2269.98486106

Esp(D3) (a.u.) = -0.0086395694

Esp(RM062X) (a.u.) = -2269.9762214906

Optimised cartesian coordinates (Angstrom):

C	6.669007	-1.596825	0.347518
C	6.462861	-2.799488	-0.344922
C	5.200758	-3.147642	-0.813529
C	4.148792	-2.263877	-0.571211
C	4.355717	-1.073104	0.126576
H	7.303629	-3.470248	-0.522131
H	5.035930	-4.079122	-1.355204
C	2.107297	-0.903381	-0.318330
C	0.708106	1.022554	0.298081
C	1.685061	1.149145	1.479177
C	3.108369	0.920002	1.005151
H	3.790707	0.830942	1.859974
S	2.470965	-2.442807	-1.042132
N	3.170580	-0.341584	0.263986
N	0.900508	-0.300336	-0.347895
C	1.523043	2.464709	2.276312
C	2.382859	3.625882	1.767275
C	1.804673	2.214930	3.759160
H	0.464329	2.758648	2.185298
H	3.449681	3.444502	1.971039
H	2.263429	3.809559	0.690487
H	2.103907	4.546753	2.298042
H	1.121539	1.461122	4.174809
H	2.837034	1.861870	3.909735
H	1.684228	3.143232	4.334749
H	1.428753	0.317652	2.156344
H	-0.301007	0.996964	0.735227

C	0.782284	2.147727	-0.712740
C	-0.116876	3.214098	-0.605647
C	1.753285	2.172433	-1.719363
C	-0.024495	4.307092	-1.465243
H	-0.898435	3.187684	0.157237
C	1.846218	3.266029	-2.580389
H	2.444023	1.335509	-1.849468
C	0.963839	4.338576	-2.449517
H	-0.730611	5.132671	-1.368988
H	2.609666	3.276225	-3.359198
H	1.037875	5.192536	-3.124003
C	-1.432691	-0.188844	-1.117729
H	-1.432459	0.894945	-1.026302
C	-2.599591	-0.845875	-1.298564
C	-3.811640	-0.092041	-1.757119
F	-3.820293	-0.035365	-3.101790
F	-4.953176	-0.656739	-1.395849
F	-3.811308	1.173774	-1.326651
O	-0.053973	-2.097986	-1.372642
C	-0.203247	-0.957205	-0.982935
H	3.468628	1.729259	0.352738
C	5.622086	-0.715620	0.593793
H	5.792244	0.221006	1.123288
H	7.669236	-1.343102	0.698908
H	-2.634115	-1.926399	-1.448126
C	-3.121043	2.329051	1.739966
H	-3.916673	2.452582	2.486918
H	-2.216930	2.848608	2.079476
H	-3.470089	2.759764	0.789685
C	-3.830577	0.119615	1.326736
O	-2.776001	0.967327	1.581243
O	-4.959765	0.567476	1.247789
C	-2.024469	-1.685759	1.625874
C	-4.258965	-2.364509	1.006721
C	-1.957540	-3.165788	1.205654
H	-1.205611	-1.085002	1.187905
C	-3.414647	-3.633857	1.208435

H -1.309353 -3.762382 1.862228
H -3.702632 -4.058125 2.185683
C -3.380470 -1.234474 1.168743
O -5.465774 -2.408785 0.787373
H -1.902206 -1.575592 2.719684
H -1.539853 -3.235641 0.189564
H -3.658840 -4.388687 0.446765

PCMopt/9_TS_SRf

Frequencies, energies and thermodynamic properties:

Lowest Vibrational Mode (1/cm) = -245.3849
2nd Lowest Vibrational Mode (1/cm) = 18.8883
E(RM062X) (a.u.) = -2267.73099425
Thermal correction to Enthalpy (a.u.) = 0.606602
Thermal correction to Gibbs Free Energy (a.u.) = 0.501614
Total Entropy (cal/Kmol) = 220.967
Esp(RM062X+D3) (a.u.) = -2269.98369158
Esp(D3) (a.u.) = -0.0081295828
Esp(RM062X) (a.u.) = -2269.9755619972

Optimised cartesian coordinates (Angstrom):

C 6.532835 -2.232857 0.271036
C 6.158957 -3.425834 -0.363448
C 4.842893 -3.641054 -0.761931
C 3.910521 -2.634869 -0.512109
C 4.285225 -1.452806 0.128463
H 6.908166 -4.195195 -0.550769
H 4.547543 -4.566145 -1.257581
C 2.050456 -1.031170 -0.233145
C 0.936618 1.072035 0.315431
C 1.942753 1.120868 1.477712
C 3.316375 0.694149 0.992125
H 3.997600 0.540869 1.839344
S 2.196720 -2.640486 -0.890315
N 3.200926 -0.583015 0.287548
N 0.938075 -0.286640 -0.265265
C 1.962252 2.473347 2.227170
C 2.957131 3.495337 1.668297

C	2.226844	2.242837	3.716303
H	0.949766	2.899785	2.133724
H	3.994636	3.185264	1.868588
H	2.846527	3.652932	0.586234
H	2.806692	4.463463	2.166180
H	1.456453	1.599017	4.163648
H	3.206197	1.763696	3.872504
H	2.234283	3.198520	4.259011
H	1.585565	0.354066	2.184874
H	-0.055273	1.183783	0.767918
C	1.133284	2.139154	-0.740570
C	0.387519	3.320584	-0.660828
C	2.074844	1.996740	-1.765392
C	0.603592	4.357400	-1.566948
H	-0.370626	3.430865	0.118841
C	2.291263	3.033479	-2.673410
H	2.645198	1.070509	-1.871661
C	1.562399	4.218518	-2.571205
H	0.017082	5.273944	-1.491473
H	3.030465	2.911461	-3.466128
H	1.732580	5.028329	-3.281842
C	-1.380618	0.044976	-1.009614
H	-1.226618	1.122194	-1.039422
C	-2.653818	-0.485397	-1.045259
C	-3.736428	0.213790	-1.806243
F	-3.499967	0.144773	-3.126744
F	-4.938376	-0.338357	-1.616868
F	-3.833851	1.514062	-1.509969
O	-0.281930	-2.055892	-1.017658
C	-0.291702	-0.858469	-0.782778
H	3.765640	1.433985	0.312662
C	5.603841	-1.228964	0.526842
H	5.902282	-0.300390	1.012084
H	7.570786	-2.083628	0.569071
H	-2.729363	-1.574635	-1.085050
C	-5.960823	-3.275864	0.778519
H	-5.633494	-3.916154	1.609077

H	-7.051012	-3.177158	0.788075
H	-5.631494	-3.734264	-0.164214
C	-4.088730	-1.863478	0.899710
O	-5.436984	-1.966357	0.910740
O	-3.387955	-2.851398	0.804492
C	-4.679741	0.622205	1.240965
C	-2.431432	-0.117202	1.685368
C	-3.832376	1.854551	1.595277
H	-5.352615	0.800997	0.392019
C	-2.608518	1.270814	2.297493
H	-4.379658	2.582161	2.208930
H	-2.795878	1.123444	3.375379
C	-3.654911	-0.471145	0.985087
O	-1.377329	-0.738746	1.739757
H	-5.323219	0.339157	2.092818
H	-3.520000	2.361311	0.671278
H	-1.688442	1.867963	2.211011

PCMopt/9_TS_SSa

Frequencies, energies and thermodynamic properties:

Lowest Vibrational Mode (1/cm) =	-208.0441
2nd Lowest Vibrational Mode (1/cm) =	20.0319
E(RM062X) (a.u.) =	-2267.73882776
Thermal correction to Enthalpy (a.u.) =	0.606652
Thermal correction to Gibbs Free Energy (a.u.) =	0.502367
Total Entropy (cal/Kmol) =	219.487
Esp(RM062X+D3) (a.u.) =	-2269.99018280
Esp(D3) (a.u.) =	-0.0085591977
Esp(RM062X) (a.u.) =	-2269.9816236023

Optimised cartesian coordinates (Angstrom):

C	6.647852	-1.380539	0.291716
C	6.491266	-2.567389	-0.437741
C	5.243484	-2.948383	-0.922699
C	4.157597	-2.113564	-0.662615
C	4.315938	-0.937129	0.072299
H	7.357395	-3.200433	-0.631141
H	5.116483	-3.869726	-1.491506

C	2.061997	-0.833555	-0.383871
C	0.598047	1.016991	0.265162
C	1.538686	1.117102	1.476337
C	2.982408	0.962220	1.032260
H	3.646927	0.848603	1.898846
S	2.483784	-2.351319	-1.133200
N	3.104799	-0.254261	0.226830
N	0.851776	-0.266696	-0.428750
C	1.303318	2.377084	2.339411
C	2.121872	3.600115	1.914930
C	1.562143	2.057839	3.813098
H	0.234402	2.629209	2.237982
H	3.191486	3.451474	2.130747
H	2.015232	3.836510	0.846966
H	1.794750	4.478706	2.488523
H	0.903024	1.253531	4.168716
H	2.604969	1.739217	3.969091
H	1.389332	2.945915	4.437211
H	1.281457	0.238560	2.090589
H	-0.413591	0.916109	0.680530
C	0.656359	2.187285	-0.692461
C	-0.299243	3.202641	-0.578036
C	1.661068	2.306431	-1.659063
C	-0.230598	4.337339	-1.384658
H	-1.107224	3.099012	0.149724
C	1.730668	3.440455	-2.468446
H	2.396714	1.509831	-1.796019
C	0.791105	4.462245	-2.326587
H	-0.981045	5.122170	-1.281398
H	2.521033	3.523226	-3.215651
H	0.847242	5.348620	-2.959860
C	-1.484903	-0.258300	-1.161594
H	-1.511808	0.825275	-1.085162
C	-2.657039	-0.961802	-1.233150
C	-3.872926	-0.262250	-1.768019
F	-3.828521	-0.226437	-3.109805
F	-5.022111	-0.857147	-1.449810

F	-3.949008	1.008814	-1.356556
O	-0.082053	-2.160760	-1.300292
C	-0.261206	-0.994250	-0.991546
H	3.330455	1.819700	0.436897
C	5.564962	-0.548189	0.558285
H	5.694744	0.377083	1.118484
H	7.635660	-1.099178	0.657152
H	-2.642453	-2.037273	-1.427246
C	-3.464644	2.192793	1.627715
H	-4.172288	2.189278	2.467019
H	-2.597413	2.813978	1.878649
H	-3.974221	2.595593	0.740624
C	-3.913534	-0.065492	1.206765
O	-2.970233	0.890866	1.370779
O	-5.097662	0.204585	1.289685
C	-4.309764	-2.576916	0.855618
C	-2.061903	-1.823897	1.319279
C	-3.544408	-3.700232	1.584433
H	-5.284979	-2.355088	1.311053
C	-2.071447	-3.352393	1.367667
H	-3.815168	-4.702762	1.226528
H	-1.704479	-3.714068	0.390706
C	-3.376578	-1.385637	0.917596
O	-1.051733	-1.145933	1.503382
H	-4.512709	-2.873963	-0.188298
H	-3.770742	-3.657272	2.661092
H	-1.382990	-3.724912	2.138385

PCMopt/9_TS_SSb

Frequencies, energies and thermodynamic properties:

Lowest Vibrational Mode (1/cm) = -245.7810

2nd Lowest Vibrational Mode (1/cm) = 22.0242

E(RM062X) (a.u.) = -2267.74289945

Thermal correction to Enthalpy (a.u.) = 0.606824

Thermal correction to Gibbs Free Energy (a.u.) = 0.503502

Total Entropy (cal/Kmol) = 217.460

Esp(RM062X+D3) (a.u.) = -2269.99108533

Esp(D3) (a.u.) = -0.0084419643

Esp(RM062X) (a.u.) = -2269.9826433657

Optimised cartesian coordinates (Angstrom):

C	6.783978	-1.031093	0.312316
C	6.719559	-2.212200	-0.439399
C	5.505086	-2.676528	-0.937255
C	4.358805	-1.930608	-0.668008
C	4.425680	-0.758703	0.088527
H	7.631285	-2.774942	-0.640405
H	5.449852	-3.594090	-1.523476
C	2.172434	-0.812353	-0.381123
C	0.580845	0.922689	0.273296
C	1.490483	1.058411	1.503388
C	2.949478	1.014133	1.084025
H	3.607197	0.921769	1.958529
S	2.707676	-2.287479	-1.145772
N	3.167104	-0.169814	0.249014
N	0.928584	-0.333056	-0.433452
C	1.144749	2.275973	2.389269
C	1.879667	3.565583	2.011747
C	1.394309	1.941521	3.861178
H	0.062230	2.447515	2.265933
H	2.953069	3.490332	2.247132
H	1.776795	3.819020	0.947302
H	1.477685	4.404061	2.597931
H	0.788479	1.082650	4.182773
H	2.454121	1.696111	4.034612
H	1.142799	2.798938	4.501458
H	1.284753	0.149864	2.092769
H	-0.433035	0.762145	0.662667
C	0.580155	2.114966	-0.657439
C	-0.459928	3.045490	-0.542098
C	1.597463	2.338432	-1.591554
C	-0.456957	4.204779	-1.316996
H	-1.278293	2.837907	0.153097
C	1.598313	3.497049	-2.368774
H	2.396961	1.605418	-1.727993

C	0.577243	4.437229	-2.225140
H	-1.269550	4.926040	-1.217476
H	2.398415	3.663606	-3.091379
H	0.579951	5.343582	-2.832333
C	-1.376852	-0.453624	-1.213359
H	-1.416534	0.632568	-1.215789
C	-2.554320	-1.164291	-1.196574
C	-3.748005	-0.527917	-1.855216
F	-3.671552	-0.689080	-3.185923
F	-4.921575	-1.040807	-1.486522
F	-3.805717	0.791288	-1.634608
O	0.110074	-2.304027	-1.262858
C	-0.137015	-1.139281	-0.995980
H	3.247435	1.909024	0.517325
C	5.640334	-0.287229	0.588017
H	5.697813	0.634515	1.165919
H	7.746405	-0.683251	0.688172
H	-2.536689	-2.253712	-1.289411
C	-5.051193	1.901181	1.062720
H	-4.616849	2.400131	0.185365
H	-6.141363	2.000775	1.049264
H	-4.642033	2.363863	1.971131
C	-3.469054	0.166323	1.057155
O	-4.774989	0.512775	1.029882
O	-2.596104	1.014100	1.150087
C	-4.368954	-2.296532	1.045151
C	-2.024564	-1.848824	1.413137
C	-3.785708	-3.372043	1.983278
H	-5.304244	-1.862073	1.421246
C	-2.282600	-3.324301	1.710385
H	-4.229501	-4.363385	1.820443
H	-2.020077	-3.900127	0.804511
C	-3.260482	-1.265072	0.920736
O	-0.930570	-1.307032	1.525302
H	-4.601605	-2.749414	0.067328
H	-3.972065	-3.087868	3.030556
H	-1.641447	-3.679330	2.527921

PCMopt/9_TS_SSc

Frequencies, energies and thermodynamic properties:

Lowest Vibrational Mode (1/cm) =	-165.5859
2nd Lowest Vibrational Mode (1/cm) =	17.9919
E(RM062X) (a.u.) =	-2267.73653774
Thermal correction to Enthalpy (a.u.) =	0.606673
Thermal correction to Gibbs Free Energy (a.u.) =	0.502117
Total Entropy (cal/Kmol) =	220.056
Esp(RM062X+D3) (a.u.) =	-2269.98934632
Esp(D3) (a.u.) =	-0.0084179850
Esp(RM062X) (a.u.) =	-2269.9809283350

Optimised cartesian coordinates (Angstrom):

C	6.591049	-1.607778	0.131303
C	6.355908	-2.736338	-0.667238
C	5.082234	-3.015778	-1.152469
C	4.049928	-2.138768	-0.820542
C	4.286104	-1.021698	-0.017529
H	7.181646	-3.403587	-0.914593
H	4.894481	-3.890575	-1.775406
C	2.036269	-0.764854	-0.423201
C	0.685202	1.118169	0.377747
C	1.654401	1.085654	1.570061
C	3.076953	0.887200	1.079078
H	3.754363	0.688743	1.919669
S	2.360562	-2.249602	-1.276867
N	3.117597	-0.283430	0.200610
N	0.854594	-0.127954	-0.406253
C	1.509526	2.294093	2.522832
C	2.390180	3.494693	2.163063
C	1.780486	1.857090	3.963876
H	0.455803	2.613475	2.466716
H	3.453365	3.270778	2.342665
H	2.276275	3.810923	1.116727
H	2.126514	4.348371	2.802992
H	1.082590	1.068907	4.279442
H	2.805859	1.469340	4.071605

H	1.673113	2.707590	4.651647
H	1.366195	0.181709	2.131354
H	-0.320831	1.042260	0.807848
C	0.785205	2.348599	-0.497389
C	-0.090346	3.416231	-0.269142
C	1.758255	2.467011	-1.495596
C	0.027982	4.597174	-1.000198
H	-0.871500	3.322447	0.489612
C	1.876760	3.648112	-2.228344
H	2.429982	1.633874	-1.717489
C	1.017654	4.718032	-1.976461
H	-0.658637	5.423127	-0.810192
H	2.641579	3.729776	-3.001706
H	1.111275	5.641102	-2.550027
C	-1.486179	0.069361	-1.133317
H	-1.437933	1.154327	-1.061638
C	-2.687786	-0.562161	-1.250968
C	-3.857382	0.229843	-1.754251
F	-3.826558	0.281308	-3.095352
F	-5.045219	-0.283614	-1.432268
F	-3.835916	1.496802	-1.324650
O	-0.180982	-1.879325	-1.432402
C	-0.290388	-0.738745	-1.021230
H	3.454094	1.759836	0.525155
C	5.562470	-0.734066	0.468590
H	5.753968	0.145903	1.081559
H	7.598713	-1.406223	0.495104
H	-2.749936	-1.639840	-1.416482
C	-0.906554	-3.614535	1.174056
H	-0.566025	-3.475565	2.209633
H	-1.099363	-4.676218	0.985227
H	-0.127573	-3.250436	0.490005
C	-2.150689	-1.622317	1.226119
O	-2.132675	-2.942883	0.948477
O	-1.119764	-1.039780	1.559887
C	-3.571360	0.425396	1.601668
C	-4.691229	-1.625608	0.916494

C	-5.082306	0.723555	1.471284
H	-3.246529	0.460392	2.657020
C	-5.764548	-0.646257	1.402469
H	-5.273141	1.277673	0.543301
H	-6.648341	-0.685229	0.749975
C	-3.405828	-0.961590	1.034946
O	-4.945513	-2.757623	0.527141
H	-2.952250	1.172617	1.071229
H	-5.453237	1.342287	2.299393
H	-6.079956	-0.996359	2.400377

PCMopt/9_TS_SSd

Frequencies, energies and thermodynamic properties:

Lowest Vibrational Mode (1/cm) = -168.5781

2nd Lowest Vibrational Mode (1/cm) = 19.2849

E(RM062X) (a.u.) = -2267.73112753

Thermal correction to Enthalpy (a.u.) = 0.606483

Thermal correction to Gibbs Free Energy (a.u.) = 0.502322

Total Entropy (cal/Kmol) = 219.224

Esp(RM062X+D3) (a.u.) = -2269.98637929

Esp(D3) (a.u.) = -0.0086001150

Esp(RM062X) (a.u.) = -2269.9777791750

Optimised cartesian coordinates (Angstrom):

C	6.632263	-1.442120	0.100543
C	6.432250	-2.584009	-0.689180
C	5.165750	-2.916332	-1.158235
C	4.103820	-2.078479	-0.817708
C	4.305046	-0.948479	-0.023581
H	7.280820	-3.219533	-0.942618
H	5.005506	-3.800765	-1.775074
C	2.043667	-0.784743	-0.404334
C	0.627726	1.059093	0.390098
C	1.626220	1.108885	1.559096
C	3.043151	0.943445	1.041059
H	3.744929	0.801707	1.872602
S	2.417442	-2.254258	-1.259737
N	3.110340	-0.254852	0.202007

N	0.831214	-0.198907	-0.368709
C	1.459299	2.349383	2.467944
C	2.292184	3.562914	2.043345
C	1.776201	1.978438	3.918094
H	0.394578	2.631319	2.423157
H	3.365168	3.380574	2.210633
H	2.148338	3.835238	0.988506
H	2.011825	4.431136	2.655839
H	1.112298	1.181633	4.281633
H	2.816195	1.629482	4.016973
H	1.655442	2.852180	4.573520
H	1.396077	0.222604	2.171878
H	-0.369282	0.961752	0.839803
C	0.661593	2.272005	-0.515909
C	-0.242688	3.315909	-0.290317
C	1.605031	2.395570	-1.541037
C	-0.181600	4.480622	-1.053323
H	-1.001980	3.217818	0.489914
C	1.666653	3.561346	-2.304754
H	2.298081	1.579709	-1.761420
C	0.779193	4.608579	-2.057238
H	-0.890285	5.288252	-0.866102
H	2.409036	3.647877	-3.099035
H	0.827861	5.519297	-2.655460
C	-1.493477	-0.036506	-1.147385
H	-1.451904	1.048608	-1.087011
C	-2.690365	-0.664207	-1.288971
C	-3.849338	0.156093	-1.779418
F	-3.845355	0.178109	-3.121039
F	-5.045466	-0.302179	-1.418125
F	-3.768816	1.429973	-1.375930
O	-0.138587	-1.956533	-1.445413
C	-0.282632	-0.826481	-1.024092
H	3.374820	1.808349	0.447439
C	5.574842	-0.607329	0.445430
H	5.739778	0.283236	1.050618
H	7.635519	-1.199120	0.450884

H	-2.767559	-1.741826	-1.449311
C	-0.112910	-2.451213	1.723162
H	0.202936	-2.887227	0.764104
H	0.712354	-1.889883	2.177891
H	-0.410584	-3.270630	2.391469
C	-2.350709	-2.098678	1.081111
O	-1.181116	-1.539055	1.538025
O	-2.370331	-3.271992	0.753568
C	-3.309173	0.223479	1.669547
C	-4.796752	-1.560517	0.956141
C	-4.744133	0.794569	1.594551
H	-2.955650	0.161297	2.714093
C	-5.663907	-0.426180	1.512423
H	-4.853987	1.399723	0.685204
H	-6.561480	-0.283305	0.893900
C	-3.416131	-1.137029	1.034827
O	-5.260624	-2.627487	0.571355
H	-2.584616	0.876444	1.144747
H	-4.974415	1.447639	2.447128
H	-6.004210	-0.749912	2.511023

PCMopt/9_TS_SSe

Frequencies, energies and thermodynamic properties:

Lowest Vibrational Mode (1/cm) =	-132.9832
2nd Lowest Vibrational Mode (1/cm) =	9.8834
E(RM062X) (a.u.) =	-2267.72372193
Thermal correction to Enthalpy (a.u.) =	0.606075
Thermal correction to Gibbs Free Energy (a.u.) =	0.498630
Total Entropy (cal/Kmol) =	226.136
Esp(RM062X+D3) (a.u.) =	-2269.98096017
Esp(D3) (a.u.) =	-0.0083006621
Esp(RM062X) (a.u.) =	-2269.9726595079

Optimised cartesian coordinates (Angstrom):

C	6.646068	-2.128282	0.311991
C	6.303982	-3.340712	-0.305391
C	4.997368	-3.594493	-0.708752
C	4.040764	-2.605333	-0.479771

C	4.383170	-1.404272	0.143197
H	7.072672	-4.094767	-0.474850
H	4.726825	-4.533896	-1.191272
C	2.144083	-1.048215	-0.233139
C	0.968907	1.039548	0.289272
C	1.974056	1.126501	1.450415
C	3.357399	0.735682	0.962701
H	4.048123	0.617895	1.807493
S	2.333666	-2.654841	-0.871763
N	3.276208	-0.559082	0.281990
N	1.002415	-0.331948	-0.270279
C	1.951973	2.485455	2.188596
C	2.910937	3.533145	1.614911
C	2.233825	2.277139	3.678026
H	0.924201	2.875774	2.098204
H	3.958097	3.257614	1.814697
H	2.792256	3.677412	0.532088
H	2.731240	4.500364	2.104429
H	1.490111	1.611180	4.138168
H	3.230733	1.835456	3.832548
H	2.209940	3.237921	4.210794
H	1.652505	0.353325	2.168530
H	-0.027570	1.149279	0.736960
C	1.140040	2.093388	-0.784417
C	0.376614	3.263990	-0.712680
C	2.072739	1.948362	-1.816655
C	0.565881	4.289286	-1.637473
H	-0.371454	3.377094	0.076391
C	2.262811	2.974813	-2.741990
H	2.656521	1.029912	-1.917161
C	1.515872	4.149247	-2.649808
H	-0.034053	5.197494	-1.569188
H	2.995427	2.852332	-3.540561
H	1.665394	4.950323	-3.374739
C	-1.326272	-0.038893	-1.006180
H	-1.179544	1.036883	-1.095743
C	-2.571416	-0.573771	-1.095355

C	-3.672025	0.228379	-1.719363
F	-3.563246	0.184070	-3.060210
F	-4.883548	-0.228903	-1.427811
F	-3.613434	1.518588	-1.379563
O	-0.177304	-2.113340	-1.060622
C	-0.193285	-0.926939	-0.801157
H	3.779062	1.475029	0.265500
C	5.693925	-1.142331	0.545930
H	5.968399	-0.199578	1.017504
H	7.678376	-1.950585	0.613691
H	-2.715903	-1.656901	-1.081183
C	-6.406304	-2.732948	0.283740
H	-6.320599	-3.629021	0.913858
H	-7.444553	-2.384636	0.268529
H	-6.086183	-2.994481	-0.735488
C	-4.304689	-1.889961	0.877953
O	-5.632137	-1.666742	0.795358
O	-3.842983	-3.000643	0.647919
C	-2.186034	-0.924071	1.916021
C	-3.980654	0.621795	1.394776
C	-1.968934	0.385790	2.703506
H	-2.169133	-1.812743	2.565179
C	-2.793013	1.423017	1.943421
H	-0.905490	0.651577	2.807491
H	-2.242494	1.813286	1.065190
C	-3.511931	-0.737231	1.232033
O	-5.063650	1.128525	1.129211
H	-1.352879	-1.065664	1.199807
H	-2.374413	0.277674	3.720873
H	-3.129133	2.284409	2.535700

PCMopt/9_TS_SSf

Frequencies, energies and thermodynamic properties:

Lowest Vibrational Mode (1/cm) =	-87.4611
2nd Lowest Vibrational Mode (1/cm) =	19.4323
E(RM062X) (a.u.) =	-2267.72360889
Thermal correction to Enthalpy (a.u.) =	0.606045

Thermal correction to Gibbs Free Energy (a.u.) = 0.499743

Total Entropy (cal/Kmol) = 223.730

Esp(RM062X+D3) (a.u.) = -2269.98181968

Esp(D3) (a.u.) = -0.0083321942

Esp(RM062X) (a.u.) = -2269.9734874858

Optimised cartesian coordinates (Angstrom):

C	6.532986	-2.151274	0.253144
C	6.165360	-3.345139	-0.385733
C	4.852326	-3.567338	-0.786360
C	3.915000	-2.565921	-0.531654
C	4.282649	-1.383951	0.112757
H	6.919311	-4.109388	-0.574587
H	4.562234	-4.491704	-1.286085
C	2.048595	-0.979440	-0.243175
C	0.913080	1.120478	0.330574
C	1.935236	1.173582	1.478955
C	3.304629	0.761621	0.970916
H	4.001515	0.619880	1.806813
S	2.206681	-2.573995	-0.919614
N	3.192198	-0.521790	0.274215
N	0.918218	-0.241840	-0.255234
C	1.952350	2.526107	2.229762
C	2.929121	3.557288	1.655918
C	2.244130	2.296157	3.713945
H	0.933729	2.941366	2.154101
H	3.972168	3.257146	1.841580
H	2.801960	3.715122	0.575844
H	2.776229	4.523407	2.156628
H	1.487709	1.644851	4.174062
H	3.230914	1.827583	3.853838
H	2.250504	3.251890	4.256249
H	1.605710	0.401275	2.193878
H	-0.074266	1.237798	0.796504
C	1.088727	2.187640	-0.729331
C	0.345618	3.369477	-0.633503
C	2.009675	2.042315	-1.772161
C	0.543856	4.404617	-1.545446

H	-0.393767	3.483185	0.163432
C	2.208274	3.078416	-2.684763
H	2.578060	1.116351	-1.890751
C	1.481857	4.263557	-2.568737
H	-0.040132	5.321626	-1.458711
H	2.931400	2.955253	-3.491827
H	1.637892	5.072378	-3.283626
C	-1.410116	0.109955	-0.984954
H	-1.273833	1.190084	-0.959340
C	-2.639799	-0.419793	-1.182351
C	-3.744688	0.440652	-1.713244
F	-3.603599	0.593104	-3.043604
F	-4.947836	-0.078354	-1.520281
F	-3.724151	1.671528	-1.192198
O	-0.279702	-1.966453	-1.135317
C	-0.279384	-0.795342	-0.817373
H	3.733808	1.501009	0.278366
C	5.600394	-1.153176	0.512159
H	5.894602	-0.224632	0.999758
H	7.570166	-1.998160	0.551596
H	-2.772891	-1.498022	-1.288650
C	-3.900782	-4.270874	0.254792
H	-4.354740	-4.187510	-0.742943
H	-3.067900	-4.981803	0.226294
H	-4.671524	-4.626443	0.953445
C	-4.251861	-1.996830	0.773206
O	-3.361702	-3.033488	0.674303
O	-5.420357	-2.170664	0.481139
C	-2.278687	-0.782298	1.926120
C	-4.287993	0.464371	1.416381
C	-2.312854	0.492239	2.795155
H	-2.099597	-1.693259	2.515579
C	-3.269777	1.421465	2.051403
H	-1.314307	0.924271	2.961688
H	-2.755540	1.944815	1.223315
C	-3.602111	-0.790676	1.217387
O	-5.438148	0.791611	1.145268

H -1.424446 -0.713367 1.217815
H -2.733625 0.252768 3.783877
H -3.766471 2.179701 2.671666

PCMopt/19_TS_RRa

Frequencies, energies and thermodynamic properties:

Lowest Vibrational Mode (1/cm) = -136.5143

2nd Lowest Vibrational Mode (1/cm) = 9.3205

E(RM062X) (a.u.) = -2588.52457405

Thermal correction to Enthalpy (a.u.) = 0.663548

Thermal correction to Gibbs Free Energy (a.u.) = 0.547937

Total Entropy (cal/Kmol) = 243.324

Esp(RM062X+D3) (a.u.) = -2591.12996736

Esp(D3) (a.u.) = -0.0098726915

Esp(RM062X) (a.u.) = -2591.1200946685

Optimised cartesian coordinates (Angstrom):

C 6.749435 -2.650684 -0.973020
C 6.124040 -3.886376 -0.749837
C 4.824370 -3.951192 -0.258886
C 4.166393 -2.750036 0.006382
C 4.794883 -1.522932 -0.208467
H 6.661440 -4.809593 -0.966875
H 4.332376 -4.908723 -0.086955
C 2.742622 -0.805517 0.544445
C 2.089273 1.551760 0.781783
C 3.592366 1.765474 1.040122
C 4.404121 0.942863 0.056632
H 5.468894 0.963975 0.320995
S 2.541092 -2.533029 0.623312
N 3.961924 -0.449950 0.127200
N 1.793050 0.099191 0.852164
C 4.021932 3.251373 1.068071
C 4.449861 3.811943 -0.292230
C 5.143232 3.448571 2.090402
H 3.150819 3.828123 1.418239
H 5.406082 3.371347 -0.614808
H 3.705646 3.638212 -1.081791

H	4.604889	4.896708	-0.208871
H	4.820496	3.157306	3.099722
H	6.027023	2.846309	1.827067
H	5.455871	4.501688	2.119890
H	3.777870	1.348648	2.043584
H	1.565632	2.003240	1.633883
C	1.582776	2.176897	-0.500139
C	1.065449	3.476831	-0.460882
C	1.679698	1.515836	-1.727182
C	0.691561	4.124336	-1.636685
H	0.956043	3.989336	0.498079
C	1.302789	2.162926	-2.903976
H	2.020756	0.479495	-1.773411
C	0.819678	3.470609	-2.862972
H	0.293644	5.138992	-1.594056
H	1.381506	1.638103	-3.856943
H	0.526592	3.974255	-3.785149
C	-0.564259	0.628143	1.362784
H	-0.440302	1.667113	1.068973
C	-1.785991	0.140727	1.695554
C	-2.899674	1.044612	2.129462
F	-2.765592	1.325359	3.435851
F	-2.922916	2.209401	1.486380
F	-4.099287	0.473769	1.991655
O	0.340894	-1.549600	1.447605
C	0.490766	-0.358908	1.233860
H	4.291686	1.298824	-0.978264
C	6.096907	-1.451734	-0.707480
H	6.585660	-0.496173	-0.893184
H	7.766251	-2.625208	-1.365111
H	-1.884699	-0.876657	2.072290
C	-0.508416	-3.246824	-1.742290
H	0.312466	-3.158127	-1.017886
H	-0.784050	-4.297822	-1.876562
H	-0.180857	-2.817705	-2.699238
C	-1.523271	-1.269749	-0.989540
O	-1.665230	-2.586562	-1.260701

O -0.489961 -0.672324 -1.233926
C -3.067303 0.667302 -0.922355
C -2.709979 -0.656930 -0.429873
O -2.332212 1.585101 -1.268343
N -3.919586 -1.406103 -0.221601
C -5.016771 -0.595047 -0.573355
C -6.394045 -0.849372 -0.529312
C -4.547124 0.662754 -0.978736
C -7.254096 0.180917 -0.912975
H -6.776261 -1.815416 -0.217256
C -5.414197 1.683644 -1.356020
C -6.784089 1.436754 -1.324320
H -8.329707 -0.004741 -0.894549
H -5.007750 2.649063 -1.663807
H -7.493061 2.212156 -1.617126
C -4.063956 -2.595898 0.468851
O -5.146710 -3.150152 0.534368
C -2.875881 -3.200448 1.183110
H -2.677189 -4.180909 0.730223
H -1.952507 -2.618301 1.157828
H -3.193125 -3.371248 2.221202

PCMopt/19_TS_RRb

Frequencies, energies and thermodynamic properties:

Lowest Vibrational Mode (1/cm) = -156.4280

2nd Lowest Vibrational Mode (1/cm) = 22.9292

E(RM062X) (a.u.) = -2588.52646284

Thermal correction to Enthalpy (a.u.) = 0.663325

Thermal correction to Gibbs Free Energy (a.u.) = 0.549911

Total Entropy (cal/Kmol) = 238.700

Esp(RM062X+D3) (a.u.) = -2591.13163861

Esp(D3) (a.u.) = -0.0108730783

Esp(RM062X) (a.u.) = -2591.1207655317

Optimised cartesian coordinates (Angstrom):

C -5.928766 -2.685738 0.446967
C -5.216215 -3.855498 0.143716
C -3.917261 -3.793671 -0.350528

C	-3.347822	-2.533292	-0.534287
C	-4.062310	-1.371403	-0.234873
H	-5.684416	-4.827423	0.300014
H	-3.357512	-4.699713	-0.584997
C	-2.060536	-0.469977	-0.907031
C	-1.514566	1.923737	-0.832604
C	-3.015851	2.100349	-1.133142
C	-3.828913	1.130393	-0.293055
H	-4.877573	1.124982	-0.618086
S	-1.745548	-2.162100	-1.135290
N	-3.304152	-0.222206	-0.481700
N	-1.149465	0.507976	-1.073726
C	-3.514261	3.559381	-1.006638
C	-4.017760	3.939374	0.389705
C	-4.605519	3.827570	-2.045246
H	-2.658162	4.209173	-1.250687
H	-4.964421	3.425697	0.618774
H	-3.297159	3.705774	1.185805
H	-4.218614	5.019247	0.424487
H	-4.234164	3.667581	-3.067139
H	-5.470246	3.163840	-1.887510
H	-4.963828	4.863555	-1.967690
H	-3.137082	1.799514	-2.186503
H	-0.973466	2.503712	-1.591712
C	-1.100350	2.404686	0.542132
C	-0.614667	3.711280	0.681561
C	-1.258362	1.609256	1.679953
C	-0.342634	4.231478	1.944779
H	-0.454382	4.328479	-0.205873
C	-0.985063	2.130507	2.945658
H	-1.581143	0.567908	1.592820
C	-0.541565	3.444885	3.081493
H	0.030074	5.251982	2.041706
H	-1.100820	1.495552	3.823426
H	-0.329460	3.850791	4.071462
C	1.225592	1.133221	-1.258615
H	1.026705	2.094460	-0.794446

C	2.507059	0.744456	-1.524922
C	3.570247	1.814117	-1.513783
F	3.897799	2.127763	-2.772871
F	3.157255	2.941511	-0.924657
F	4.703585	1.464085	-0.901731
O	0.389547	-0.968470	-1.884552
C	0.187637	0.153525	-1.457063
H	-3.795999	1.376920	0.778647
C	-5.364818	-1.427485	0.264139
H	-5.919057	-0.523590	0.514336
H	-6.943368	-2.760114	0.838661
H	2.737571	-0.124624	-2.145752
C	6.605697	-0.823689	-0.064427
H	6.916031	-1.367410	0.838020
H	7.192625	-1.158537	-0.925620
H	6.760648	0.251542	0.102007
C	4.357602	-0.740135	0.564143
O	5.250358	-1.086694	-0.377902
O	4.701990	-0.302921	1.646903
C	2.678180	-2.131706	-0.699505
C	2.981935	-0.967524	0.141703
O	3.225446	-2.487434	-1.734024
N	1.977364	-0.910304	1.161826
C	1.171913	-2.066949	1.097676
C	0.121639	-2.501653	1.917516
C	1.566765	-2.833492	-0.007684
C	-0.467855	-3.732201	1.615548
H	-0.198324	-1.919391	2.775443
C	0.967097	-4.051332	-0.305428
C	-0.054108	-4.511004	0.526283
H	-1.271507	-4.097634	2.258132
H	1.304726	-4.623136	-1.172006
H	-0.533465	-5.471858	0.332268
C	1.800314	0.085264	2.109306
O	1.094254	-0.105375	3.083158
C	2.428168	1.435740	1.876258
H	2.911034	1.749072	2.810184

H 3.158484 1.463797 1.068996

H 1.604436 2.137310 1.669102

PCMopt/19_TS_RRc

Frequencies, energies and thermodynamic properties:

Lowest Vibrational Mode (1/cm) = -96.0199

2nd Lowest Vibrational Mode (1/cm) = 14.9126

E(RM062X) (a.u.) = -2588.51951277

Thermal correction to Enthalpy (a.u.) = 0.663060

Thermal correction to Gibbs Free Energy (a.u.) = 0.548193

Total Entropy (cal/Kmol) = 241.758

Esp(RM062X+D3) (a.u.) = -2591.12893432

Esp(D3) (a.u.) = -0.0108308026

Esp(RM062X) (a.u.) = -2591.1181035174

Optimised cartesian coordinates (Angstrom):

C -6.350237 -2.548257 -0.131041

C -5.697293 -3.775706 -0.322682

C -4.336707 -3.825971 -0.604617

C -3.643606 -2.617564 -0.690197

C -4.298067 -1.398878 -0.505143

H -6.263209 -4.704255 -0.246641

H -3.824155 -4.776719 -0.752227

C -2.150959 -0.666275 -0.867964

C -1.457674 1.682073 -0.728633

C -2.873157 1.967121 -1.266678

C -3.882889 1.071059 -0.574072

H -4.853202 1.117071 -1.085553

S -1.943360 -2.381829 -1.031192

N -3.421761 -0.317154 -0.638381

N -1.153940 0.245385 -0.931078

C -3.268399 3.462377 -1.220155

C -3.964395 3.897317 0.073498

C -4.146026 3.804671 -2.426012

H -2.333630 4.038967 -1.315293

H -4.972812 3.460560 0.143422

H -3.405113 3.620976 0.978391

H -4.084109 4.989654 0.073555

H	-3.622147	3.607470	-3.371710
H	-5.073231	3.210283	-2.418098
H	-4.431060	4.865729	-2.405532
H	-2.841327	1.666383	-2.326521
H	-0.774127	2.231932	-1.384532
C	-1.246567	2.127029	0.702507
C	-0.663902	3.376636	0.944391
C	-1.737924	1.383770	1.782703
C	-0.631000	3.904322	2.234812
H	-0.252639	3.953805	0.112435
C	-1.708446	1.913241	3.073288
H	-2.150710	0.383501	1.630097
C	-1.170797	3.181285	3.299164
H	-0.183303	4.883672	2.407735
H	-2.105080	1.328911	3.904991
H	-1.154127	3.596084	4.307733
C	1.250403	0.783841	-1.189034
H	1.108679	1.790471	-0.802809
C	2.471888	0.383277	-1.612250
C	3.529537	1.411535	-1.918488
F	3.653055	1.478184	-3.259068
F	3.201860	2.635535	-1.510240
F	4.741969	1.136167	-1.451716
O	0.336373	-1.354568	-1.554138
C	0.170523	-0.193656	-1.243945
H	-4.031695	1.343907	0.480776
C	-5.663782	-1.342533	-0.218621
H	-6.174919	-0.393963	-0.058515
H	-7.416701	-2.535989	0.094326
H	2.626274	-0.593674	-2.072744
C	6.703450	-1.272060	-0.910888
H	7.239128	-1.683935	-0.044390
H	6.994693	-1.808061	-1.820386
H	6.959895	-0.207087	-1.005153
C	4.755614	-0.869397	0.318215
O	5.306107	-1.437017	-0.776825
O	5.433935	-0.294618	1.145567

C 2.698377 -2.332219 -0.011537
C 3.321672 -1.083481 0.364792
O 3.029650 -3.169697 -0.852660
N 2.554131 -0.468962 1.397678
C 1.457793 -1.300005 1.706848
C 0.393551 -1.164837 2.611790
C 1.532317 -2.448373 0.900758
C -0.533163 -2.205568 2.701181
H 0.266290 -0.299213 3.253174
C 0.609672 -3.483609 1.004796
C -0.430624 -3.365551 1.921955
H -1.354000 -2.106875 3.414393
H 0.712730 -4.356280 0.356381
H -1.173253 -4.158197 2.025783
C 2.689286 0.887086 1.677945
O 3.397692 1.595520 0.994721
C 1.954288 1.438544 2.872144
H 0.890504 1.568211 2.631241
H 2.042513 0.784499 3.748402
H 2.380032 2.423164 3.091722

PCMopt/19_TS_RRd
Frequencies, energies and thermodynamic properties:
Lowest Vibrational Mode (1/cm) = -133.9823
2nd Lowest Vibrational Mode (1/cm) = 21.5280
E(RM062X) (a.u.) = -2588.52198019
Thermal correction to Enthalpy (a.u.) = 0.663262
Thermal correction to Gibbs Free Energy (a.u.) = 0.549315
Total Entropy (cal/Kmol) = 239.822
Esp(RM062X+D3) (a.u.) = -2591.12792915
Esp(D3) (a.u.) = -0.0096146037
Esp(RM062X) (a.u.) = -2591.1183145463
Optimised cartesian coordinates (Angstrom):
C 6.829900 -2.537681 -1.112850
C 6.218007 -3.791594 -0.969838
C 4.919114 -3.901848 -0.484110
C 4.246880 -2.727880 -0.144272

C	4.862408	-1.483037	-0.279975
H	6.765210	-4.693208	-1.245422
H	4.438301	-4.873958	-0.373848
C	2.800932	-0.835809	0.512803
C	2.105174	1.494225	0.823038
C	3.597929	1.725960	1.125633
C	4.447637	0.955898	0.131957
H	5.503750	0.973718	0.430130
S	2.621360	-2.568159	0.492143
N	4.018366	-0.442286	0.122012
N	1.834640	0.036742	0.850828
C	3.999133	3.217205	1.219652
C	4.456264	3.834907	-0.105842
C	5.086988	3.396222	2.280754
H	3.107718	3.765758	1.564241
H	5.431493	3.426320	-0.413317
H	3.740798	3.673617	-0.923976
H	4.584455	4.919068	0.020234
H	4.740536	3.064696	3.269584
H	5.987358	2.816933	2.022056
H	5.381417	4.452368	2.355696
H	3.766224	1.274928	2.117163
H	1.551733	1.916449	1.670825
C	1.625632	2.144362	-0.457050
C	1.087910	3.435125	-0.401528
C	1.772855	1.518671	-1.698198
C	0.745225	4.109132	-1.572041
H	0.939517	3.920205	0.566368
C	1.429052	2.192431	-2.870245
H	2.135746	0.490381	-1.761539
C	0.926261	3.492111	-2.810608
H	0.331186	5.116673	-1.516001
H	1.549931	1.695466	-3.833621
H	0.658984	4.017547	-3.728431
C	-0.540104	0.488837	1.346956
H	-0.419815	1.539097	1.093453
C	-1.770862	-0.017241	1.633877

C	-2.864216	0.889848	2.108781
F	-2.701203	1.149385	3.417133
F	-2.882590	2.069389	1.486752
F	-4.081025	0.349813	1.988058
O	0.444567	-1.659395	1.457265
C	0.540741	-0.466529	1.233399
H	4.364488	1.358723	-0.888435
C	6.163709	-1.365403	-0.771511
H	6.641746	-0.394248	-0.893807
H	7.846559	-2.475969	-1.501363
H	-1.888376	-1.060862	1.928550
C	-0.669438	-3.491155	-1.395491
H	0.091737	-3.385214	-0.610214
H	-1.003079	-4.531729	-1.463414
H	-0.233214	-3.172135	-2.352319
C	-1.603713	-1.399677	-0.924709
O	-1.813351	-2.722204	-1.072969
O	-0.532229	-0.885556	-1.191675
C	-3.015369	0.656486	-1.003668
C	-2.763638	-0.665033	-0.455892
O	-2.218955	1.510387	-1.378997
N	-4.026643	-1.324476	-0.242773
C	-5.061323	-0.449093	-0.639739
C	-6.457391	-0.584377	-0.649644
C	-4.490777	0.757300	-1.078168
C	-7.222328	0.491819	-1.103033
H	-6.970169	-1.488322	-0.339759
C	-5.260683	1.825451	-1.527198
C	-6.645616	1.691734	-1.538615
H	-8.308013	0.382188	-1.120736
H	-4.763621	2.738993	-1.859241
H	-7.281034	2.507114	-1.885771
C	-4.135472	-2.379064	0.657116
O	-3.182476	-2.744731	1.313758
C	-5.467795	-3.075768	0.780018
H	-6.169531	-2.457490	1.357199
H	-5.908967	-3.292385	-0.200021

H -5.294616 -4.008511 1.325901

PCMopt/19_TS_RRe

Frequencies, energies and thermodynamic properties:

Lowest Vibrational Mode (1/cm) = -114.4746

2nd Lowest Vibrational Mode (1/cm) = 19.1236

E(RM062X) (a.u.) = -2588.51813039

Thermal correction to Enthalpy (a.u.) = 0.663187

Thermal correction to Gibbs Free Energy (a.u.) = 0.548246

Total Entropy (cal/Kmol) = 241.913

Esp(RM062X+D3) (a.u.) = -2591.12735128

Esp(D3) (a.u.) = -0.0108287828

Esp(RM062X) (a.u.) = -2591.1165224972

Optimised cartesian coordinates (Angstrom):

C -6.473427 -2.333457 0.086748

C -5.886195 -3.585439 -0.152415

C -4.544620 -3.691280 -0.502198

C -3.802860 -2.513703 -0.606954

C -4.392792 -1.270358 -0.375229

H -6.488473 -4.489292 -0.060064

H -4.083212 -4.661476 -0.687142

C -2.235126 -0.626400 -0.832992

C -1.435454 1.688493 -0.704132

C -2.861301 2.038498 -1.172077

C -3.873525 1.179535 -0.437553

H -4.865329 1.273247 -0.898735

S -2.112138 -2.347651 -1.028322

N -3.477069 -0.226165 -0.538374

N -1.203459 0.241801 -0.931061

C -3.190789 3.548480 -1.095894

C -3.810035 3.999261 0.231034

C -4.106326 3.939023 -2.258083

H -2.238051 4.086896 -1.228486

H -4.832928 3.606530 0.340869

H -3.225141 3.688756 1.108211

H -3.881082 5.095743 0.246349

H -3.634201 3.729682 -3.228108

H	-5.056448	3.383795	-2.212868
H	-4.345338	5.010783	-2.215997
H	-2.893231	1.745941	-2.234202
H	-0.759115	2.214245	-1.387014
C	-1.141053	2.113753	0.718493
C	-0.506341	3.341895	0.938883
C	-1.606437	1.380753	1.816921
C	-0.395810	3.860448	2.228530
H	-0.115960	3.910036	0.090933
C	-1.498138	1.900690	3.107317
H	-2.060317	0.396132	1.679713
C	-0.908489	3.148684	3.313778
H	0.091673	4.823478	2.384789
H	-1.874594	1.324444	3.953926
H	-0.830613	3.556142	4.322468
C	1.208180	0.676643	-1.284535
H	1.122721	1.684523	-0.885699
C	2.398125	0.232807	-1.759811
C	3.469271	1.230668	-2.123431
F	3.538921	1.271620	-3.469353
F	3.185098	2.469625	-1.725826
F	4.693019	0.937785	-1.701873
O	0.182410	-1.412364	-1.647462
C	0.086453	-0.250683	-1.309976
H	-3.957530	1.446576	0.625967
C	-5.738743	-1.158042	-0.020403
H	-6.198388	-0.190261	0.176539
H	-7.525825	-2.277423	0.365345
H	2.489249	-0.739232	-2.247758
C	6.779227	-0.280249	0.772953
H	6.972565	0.254144	-0.167897
H	7.125783	0.317992	1.622122
H	7.316618	-1.238375	0.745861
C	4.757632	-1.140181	-0.031413
O	5.390935	-0.476841	0.958655
O	5.373949	-1.664205	-0.938038
C	2.622507	-2.432375	-0.210015

C 3.319802 -1.216137 0.143670
O 2.884365 -3.268377 -1.075347
N 2.631318 -0.577776 1.220069
C 1.516455 -1.368053 1.574609
C 0.506923 -1.202351 2.536414
C 1.498637 -2.508459 0.754256
C -0.460430 -2.201369 2.658326
H 0.451409 -0.344316 3.197608
C 0.536205 -3.503081 0.890756
C -0.451390 -3.352337 1.858928
H -1.238672 -2.077110 3.414054
H 0.567720 -4.369323 0.226654
H -1.223908 -4.111734 1.989114
C 2.808737 0.777378 1.485877
O 3.498808 1.468240 0.767528
C 2.144084 1.350062 2.710510
H 1.072037 1.495515 2.521213
H 2.265418 0.700995 3.586486
H 2.597021 2.328755 2.899885

PCMopt/19_TS_RRg
Frequencies, energies and thermodynamic properties:
Lowest Vibrational Mode (1/cm) = -204.9316
2nd Lowest Vibrational Mode (1/cm) = 9.2522
E(RM062X) (a.u.) = -2588.51657620
Thermal correction to Enthalpy (a.u.) = 0.663288
Thermal correction to Gibbs Free Energy (a.u.) = 0.548083
Total Entropy (cal/Kmol) = 242.469
Esp(RM062X+D3) (a.u.) = -2591.11880915
Esp(D3) (a.u.) = -0.0100676557
Esp(RM062X) (a.u.) = -2591.1087414943
Optimised cartesian coordinates (Angstrom):
C -6.471117 2.371880 -1.578028
C -5.830264 3.617613 -1.627978
C -4.545579 3.781131 -1.117726
C -3.917278 2.669582 -0.557796
C -4.563129 1.433395 -0.500876

H	-6.341963	4.470201	-2.074738
H	-4.042062	4.747391	-1.156728
C	-2.547678	0.887719	0.469351
C	-1.895206	-1.376489	1.099980
C	-3.393073	-1.563073	1.410188
C	-4.231267	-0.911367	0.324386
H	-5.284199	-0.855769	0.631107
S	-2.318655	2.589355	0.161973
N	-3.764294	0.458245	0.106528
N	-1.617657	0.070271	0.975130
C	-3.797998	-3.031498	1.680903
C	-4.233115	-3.813445	0.437460
C	-4.900635	-3.081968	2.740646
H	-2.909707	-3.530140	2.102535
H	-5.201851	-3.448385	0.062041
H	-3.502398	-3.759004	-0.381444
H	-4.366045	-4.872373	0.699828
H	-4.571135	-2.624455	3.684013
H	-5.800247	-2.547196	2.397403
H	-5.190464	-4.122046	2.945814
H	-3.565774	-0.996231	2.339565
H	-1.349981	-1.705814	1.993028
C	-1.409302	-2.173314	-0.093921
C	-0.806472	-3.416343	0.124553
C	-1.627244	-1.744870	-1.407578
C	-0.470512	-4.241600	-0.948486
H	-0.605209	-3.748133	1.146416
C	-1.302943	-2.573180	-2.480748
H	-2.038611	-0.753177	-1.606764
C	-0.733422	-3.827602	-2.254023
H	-0.004837	-5.210245	-0.762362
H	-1.486372	-2.231676	-3.500514
H	-0.478929	-4.473226	-3.095888
C	0.730258	-0.317274	1.569113
H	0.627558	-1.363814	1.292193
C	1.983496	0.216432	1.791145
C	3.090205	-0.739408	2.120469

F	2.876304	-1.345205	3.298066
F	3.212948	-1.711357	1.211202
F	4.280588	-0.136415	2.224004
O	-0.240750	1.828115	1.450354
C	-0.331149	0.612420	1.356118
H	-4.174469	-1.457139	-0.629230
C	-5.849020	1.261589	-1.014923
H	-6.349095	0.294198	-0.987140
H	-7.475234	2.265642	-1.988983
H	2.079738	1.203388	2.246906
C	0.886990	4.279561	-0.425030
H	1.613615	5.087694	-0.267167
H	0.235217	4.514979	-1.272256
H	0.296048	4.137452	0.490145
C	2.319572	2.552049	0.202782
O	1.547060	3.065430	-0.754016
O	2.577773	3.167780	1.210432
C	1.876612	0.354531	-0.973389
C	2.745689	1.154220	-0.129832
O	0.646844	0.395811	-1.031790
N	4.112123	0.783508	-0.439614
C	4.077236	-0.328081	-1.312901
C	5.107412	-1.128630	-1.831017
C	2.746053	-0.626911	-1.630209
C	4.747860	-2.200731	-2.648225
H	6.148066	-0.926344	-1.607695
C	2.395363	-1.701649	-2.443641
C	3.412052	-2.498797	-2.957630
H	5.541890	-2.828306	-3.057331
H	1.340665	-1.897059	-2.647327
H	3.178553	-3.352257	-3.595614
C	5.313498	1.291010	0.044995
O	6.370959	0.833872	-0.343883
C	5.293725	2.421979	1.037125
H	4.997170	3.358177	0.546460
H	4.589387	2.243770	1.853447
H	6.316587	2.521475	1.414630

PCMopt/19_TS_RRh

Frequencies, energies and thermodynamic properties:

Lowest Vibrational Mode (1/cm) = -333.8255

2nd Lowest Vibrational Mode (1/cm) = 16.7436

E(RM062X) (a.u.) = -2588.51543249

Thermal correction to Enthalpy (a.u.) = 0.662995

Thermal correction to Gibbs Free Energy (a.u.) = 0.549329

Total Entropy (cal/Kmol) = 239.231

Esp(RM062X+D3) (a.u.) = -2591.11724781

Esp(D3) (a.u.) = -0.0105205699

Esp(RM062X) (a.u.) = -2591.1067272401

Optimised cartesian coordinates (Angstrom):

C	6.425106	2.502325	0.445349
C	5.783451	3.747430	0.383741
C	4.432318	3.839696	0.062905
C	3.736849	2.658263	-0.193444
C	4.382504	1.422207	-0.139895
H	6.348153	4.656196	0.592702
H	3.928803	4.805676	0.016525
C	2.243988	0.747004	-0.666450
C	1.547573	-1.583380	-0.784154
C	2.972005	-1.837268	-1.313141
C	3.974149	-1.016427	-0.522451
H	4.949457	-1.006831	-1.027300
S	2.044895	2.478329	-0.620677
N	3.511309	0.369445	-0.439207
N	1.258964	-0.137037	-0.875805
C	3.347501	-3.335832	-1.393795
C	3.986872	-3.906306	-0.123809
C	4.263659	-3.581261	-2.594654
H	2.407827	-3.883769	-1.575246
H	4.997687	-3.496301	0.027373
H	3.394793	-3.709185	0.780793
H	4.092130	-4.995468	-0.226539
H	3.780491	-3.282078	-3.535362
H	5.201619	-3.012341	-2.496217

H	4.527268	-4.645749	-2.667195
H	2.967175	-1.442727	-2.342358
H	0.872484	-2.069086	-1.496551
C	1.294615	-2.145489	0.599150
C	0.629554	-3.369410	0.720028
C	1.803569	-1.531074	1.750176
C	0.518413	-3.997518	1.960196
H	0.204412	-3.844157	-0.167825
C	1.704473	-2.164169	2.987856
H	2.265328	-0.543678	1.696478
C	1.071860	-3.403699	3.094432
H	0.000494	-4.954330	2.038537
H	2.112468	-1.679390	3.875962
H	0.994910	-3.897410	4.064179
C	-1.133495	-0.603220	-1.217851
H	-0.988734	-1.655798	-0.991213
C	-2.424817	-0.121247	-1.411949
C	-3.420069	-1.022191	-2.085211
F	-3.160668	-1.082819	-3.402337
F	-3.368508	-2.282226	-1.633691
F	-4.677526	-0.606973	-1.968017
O	-0.167984	1.546244	-1.422769
C	-0.072168	0.349040	-1.186483
H	4.115453	-1.401541	0.497935
C	5.736251	1.321697	0.184676
H	6.238223	0.356526	0.241970
H	7.482963	2.453457	0.704248
H	-2.520254	0.918520	-1.738837
C	-5.074623	-2.829293	0.913504
H	-4.788877	-3.197880	1.907803
H	-6.157439	-2.910197	0.777901
H	-4.549704	-3.418285	0.150448
C	-3.465397	-1.135288	0.891012
O	-4.761082	-1.451471	0.778542
O	-2.615913	-1.967126	1.129857
C	-4.243369	1.288614	0.254673
C	-3.211685	0.271611	0.571098

O	-5.427300	1.158008	0.006488
N	-2.052211	0.971420	1.035662
C	-2.187041	2.330135	0.685051
C	-1.275781	3.387086	0.784613
C	-3.490578	2.565445	0.233501
C	-1.721659	4.657827	0.421060
H	-0.254766	3.217753	1.112500
C	-3.926971	3.837337	-0.126996
C	-3.027311	4.895385	-0.034828
H	-1.021947	5.493263	0.486639
H	-4.953850	3.979569	-0.468941
H	-3.329350	5.904637	-0.316397
C	-0.987853	0.548380	1.841078
O	0.094263	1.095755	1.738582
C	-1.244768	-0.456803	2.933942
H	-2.298051	-0.483553	3.233702
H	-0.950621	-1.460106	2.605748
H	-0.611583	-0.156587	3.778050

PCMopt/19_TS_RRi

Frequencies, energies and thermodynamic properties:

Lowest Vibrational Mode (1/cm) = -262.8452

2nd Lowest Vibrational Mode (1/cm) = 16.9951

E(RM062X) (a.u.) = -2588.51185117

Thermal correction to Enthalpy (a.u.) = 0.663294

Thermal correction to Gibbs Free Energy (a.u.) = 0.548345

Total Entropy (cal/Kmol) = 241.930

Esp(RM062X+D3) (a.u.) = -2591.11565093

Esp(D3) (a.u.) = -0.0099722384

Esp(RM062X) (a.u.) = -2591.1056786916

Optimised cartesian coordinates (Angstrom):

C	6.460900	-2.410234	-1.535560
C	5.807565	-3.648715	-1.596348
C	4.516427	-3.800439	-1.098103
C	3.893954	-2.684992	-0.539789
C	4.552642	-1.455940	-0.472177
H	6.314105	-4.504955	-2.042054

H	4.003338	-4.761306	-1.145720
C	2.532643	-0.891549	0.479099
C	1.890752	1.378547	1.086154
C	3.384607	1.556761	1.421370
C	4.237421	0.889932	0.355792
H	5.282914	0.822759	0.685254
S	2.288951	-2.591432	0.166695
N	3.758711	-0.474252	0.130817
N	1.605921	-0.065685	0.971023
C	3.796230	3.023739	1.689139
C	4.257911	3.794895	0.448501
C	4.881069	3.073313	2.767204
H	2.904465	3.531452	2.092398
H	5.230505	3.421182	0.092013
H	3.541075	3.740307	-0.382685
H	4.393481	4.854554	0.706627
H	4.532465	2.623743	3.707532
H	5.782684	2.530340	2.442565
H	5.174617	4.112592	2.971212
H	3.535305	0.995637	2.357994
H	1.330696	1.721149	1.964906
C	1.434232	2.167060	-0.124992
C	0.834082	3.415549	0.068978
C	1.683717	1.728572	-1.430050
C	0.533691	4.236706	-1.017838
H	0.608454	3.755419	1.083052
C	1.396005	2.553293	-2.516623
H	2.094077	0.733230	-1.612351
C	0.830909	3.813777	-2.313118
H	0.070028	5.209708	-0.850187
H	1.605351	2.204493	-3.528878
H	0.605958	4.456729	-3.165378
C	-0.750331	0.330307	1.530064
H	-0.647945	1.377491	1.254837
C	-2.008554	-0.225847	1.718480
C	-3.135481	0.685061	2.102072
F	-2.919742	1.235023	3.307278

F	-3.302498	1.700563	1.249100
F	-4.306148	0.040500	2.194545
O	0.221432	-1.816421	1.450782
C	0.308808	-0.599052	1.342771
H	4.208263	1.433756	-0.600212
C	5.844849	-1.295704	-0.973542
H	6.354385	-0.333519	-0.936855
H	7.469814	-2.312783	-1.936890
H	-2.081461	-1.206996	2.188445
C	-1.083656	-4.409934	-0.318652
H	-1.841941	-5.161644	-0.062153
H	-0.495359	-4.742852	-1.179475
H	-0.433615	-4.239399	0.550731
C	-2.387437	-2.567395	0.259606
O	-1.694200	-3.185258	-0.699501
O	-2.619748	-3.083141	1.323346
C	-1.830421	-0.397266	-0.998782
C	-2.724875	-1.161512	-0.148680
O	-0.607233	-0.491083	-1.087128
N	-4.068206	-0.722931	-0.426348
C	-4.003770	0.437808	-1.223349
C	-4.988088	1.331922	-1.669365
C	-2.663802	0.668336	-1.573548
C	-4.583372	2.426247	-2.434829
H	-6.042715	1.221335	-1.442980
C	-2.267263	1.765648	-2.332206
C	-3.240942	2.658974	-2.765174
H	-5.347801	3.125052	-2.778694
H	-1.208123	1.902956	-2.558061
H	-2.968698	3.534993	-3.355139
C	-5.197754	-1.463646	-0.091495
O	-5.098470	-2.631257	0.213533
C	-6.533049	-0.764125	-0.094576
H	-6.473127	0.219979	0.384985
H	-6.905289	-0.643215	-1.121068
H	-7.230578	-1.407274	0.451501

PCMopt/19_TS_RRj

Frequencies, energies and thermodynamic properties:

Lowest Vibrational Mode (1/cm) =	-268.7142
2nd Lowest Vibrational Mode (1/cm) =	15.7602
E(RM062X) (a.u.) =	-2588.50756871
Thermal correction to Enthalpy (a.u.) =	0.662568
Thermal correction to Gibbs Free Energy (a.u.) =	0.547046
Total Entropy (cal/Kmol) =	243.136
Esp(RM062X+D3) (a.u.) =	-2591.11209298
Esp(D3) (a.u.) =	-0.0105166299
Esp(RM062X) (a.u.) =	-2591.1015763501

Optimised cartesian coordinates (Angstrom):

C	-6.849810	-2.033719	0.220553
C	-6.352329	-3.342443	0.141768
C	-5.010347	-3.581588	-0.137653
C	-4.176920	-2.480713	-0.334455
C	-4.678296	-1.180045	-0.263634
H	-7.024066	-4.185506	0.304004
H	-4.619174	-4.597391	-0.198298
C	-2.461491	-0.742550	-0.701111
C	-1.495785	1.500801	-0.769547
C	-2.870021	1.909030	-1.331664
C	-3.975075	1.206399	-0.564508
H	-4.936942	1.320441	-1.081738
S	-2.464578	-2.484741	-0.711945
N	-3.682131	-0.226752	-0.500639
N	-1.369188	0.026069	-0.838327
C	-3.074524	3.439877	-1.420472
C	-3.672392	4.079094	-0.163261
C	-3.934846	3.781518	-2.638974
H	-2.076863	3.880064	-1.584550
H	-4.723398	3.778432	-0.030509
H	-3.121431	3.823276	0.752380
H	-3.659883	5.172886	-0.269124
H	-3.470722	3.426514	-3.569748
H	-4.932586	3.322202	-2.557402
H	-4.075597	4.868573	-2.717895

H	-2.886968	1.514482	-2.360661
H	-0.751433	1.892861	-1.472066
C	-1.200545	2.045933	0.611233
C	-0.395416	3.183451	0.731690
C	-1.777657	1.496672	1.761098
C	-0.196430	3.783720	1.974085
H	0.076369	3.609620	-0.157468
C	-1.586792	2.100678	3.003145
H	-2.372049	0.581609	1.703734
C	-0.801417	3.248752	3.111701
H	0.434390	4.669971	2.052911
H	-2.048642	1.667175	3.891694
H	-0.652318	3.719412	4.084378
C	1.066934	0.232123	-1.079877
H	1.029236	1.238328	-0.668794
C	2.262018	-0.259428	-1.580968
C	3.217217	0.798982	-2.063380
F	2.655358	1.521497	-3.046211
F	3.547803	1.666381	-1.103652
F	4.341170	0.297481	-2.583115
O	-0.115762	-1.774254	-1.464923
C	-0.095980	-0.597133	-1.145387
H	-4.083874	1.591868	0.459706
C	-6.022665	-0.933380	0.019461
H	-6.413084	0.081172	0.090138
H	-7.903887	-1.870927	0.445913
H	2.261878	-1.180065	-2.171071
C	6.791924	0.027500	-0.812424
H	6.713612	-0.007830	-1.907046
H	7.207287	0.988637	-0.492811
H	7.433980	-0.795733	-0.472473
C	4.820286	-1.192984	-0.464073
O	5.503995	-0.063684	-0.226740
O	5.281446	-2.093996	-1.127002
C	2.640327	-2.406435	0.059447
C	3.463441	-1.182911	0.090180
O	2.760926	-3.388856	-0.660748

N	2.988433	-0.351914	1.154055
C	1.770621	-0.885258	1.605580
C	0.869222	-0.393158	2.564582
C	1.565385	-2.146918	1.028532
C	-0.213374	-1.197744	2.901539
H	0.993816	0.592887	3.005921
C	0.466196	-2.935701	1.367350
C	-0.430291	-2.457963	2.313293
H	-0.927267	-0.825860	3.639507
H	0.326961	-3.900432	0.875392
H	-1.304563	-3.045750	2.596014
C	3.591712	0.695634	1.875290
O	2.907250	1.617589	2.259413
C	5.025431	0.565513	2.332547
H	5.012922	0.810744	3.403029
H	5.439316	-0.434332	2.179254
H	5.656747	1.300465	1.819836

PCMopt/19_TS_RRk

Frequencies, energies and thermodynamic properties:

Lowest Vibrational Mode (1/cm) = -191.5785

2nd Lowest Vibrational Mode (1/cm) = 19.0640

E(RM062X) (a.u.) = -2588.50800212

Thermal correction to Enthalpy (a.u.) = 0.662658

Thermal correction to Gibbs Free Energy (a.u.) = 0.547536

Total Entropy (cal/Kmol) = 242.295

Esp(RM062X+D3) (a.u.) = -2591.11236966

Esp(D3) (a.u.) = -0.0105805378

Esp(RM062X) (a.u.) = -2591.1017891222

Optimised cartesian coordinates (Angstrom):

C	-6.781181	-2.102494	0.209461
C	-6.264091	-3.403106	0.116953
C	-4.920961	-3.619959	-0.173089
C	-4.106154	-2.504382	-0.366918
C	-4.626590	-1.212156	-0.281762
H	-6.921849	-4.257453	0.277310
H	-4.514443	-4.628946	-0.243808

C	-2.420138	-0.738518	-0.725823
C	-1.488528	1.525538	-0.795762
C	-2.874287	1.905147	-1.350686
C	-3.958407	1.186702	-0.568647
H	-4.929499	1.289886	-1.070381
S	-2.397265	-2.478638	-0.753125
N	-3.645676	-0.242647	-0.515548
N	-1.337983	0.051113	-0.860824
C	-3.111866	3.431011	-1.444068
C	-3.727519	4.057977	-0.189194
C	-3.977840	3.748998	-2.664935
H	-2.124709	3.894042	-1.607278
H	-4.773023	3.736670	-0.061658
H	-3.175671	3.812125	0.728685
H	-3.735737	5.151873	-0.294225
H	-3.502608	3.404751	-3.594145
H	-4.963502	3.263984	-2.584837
H	-4.146330	4.831952	-2.745598
H	-2.893289	1.504652	-2.377338
H	-0.754951	1.928900	-1.503774
C	-1.201175	2.081145	0.581851
C	-0.463566	3.264814	0.693553
C	-1.726881	1.494483	1.737408
C	-0.287762	3.875193	1.933624
H	-0.028743	3.718194	-0.200900
C	-1.554252	2.106211	2.978714
H	-2.267176	0.546053	1.684495
C	-0.842882	3.302110	3.078001
H	0.288227	4.798543	2.006399
H	-1.973468	1.641812	3.872787
H	-0.709963	3.779926	4.049579
C	1.100947	0.300557	-1.093326
H	1.053006	1.296815	-0.656435
C	2.285370	-0.181271	-1.591238
C	3.323114	0.843724	-1.956571
F	2.860364	1.657716	-2.917446
F	3.651118	1.631289	-0.925999

F	4.445007	0.306031	-2.437379
O	-0.052087	-1.721625	-1.488257
C	-0.062063	-0.549143	-1.165620
H	-4.054763	1.566560	0.458915
C	-5.972850	-0.988071	0.012026
H	-6.378102	0.019752	0.094316
H	-7.836036	-1.958057	0.443429
H	2.312525	-1.112190	-2.162897
C	6.908291	-0.213392	-0.618937
H	6.827336	-0.184513	-1.713687
H	7.396793	0.694737	-0.249939
H	7.488036	-1.099580	-0.328832
C	4.835822	-1.275241	-0.354628
O	5.620024	-0.236225	-0.030435
O	5.237955	-2.160064	-1.079510
C	2.645239	-2.417786	0.072519
C	3.476490	-1.212974	0.178202
O	2.772587	-3.383046	-0.674237
N	2.939786	-0.386300	1.235150
C	1.726489	-0.968938	1.653958
C	0.787758	-0.541473	2.612955
C	1.556091	-2.211841	1.034516
C	-0.277145	-1.387215	2.903462
H	0.868476	0.425757	3.098859
C	0.479716	-3.048622	1.328117
C	-0.447089	-2.633520	2.273143
H	-1.010962	-1.060203	3.643692
H	0.384951	-3.999634	0.800124
H	-1.306452	-3.257532	2.522608
C	3.290827	0.882889	1.733703
O	2.410558	1.641914	2.081951
C	4.729541	1.260745	1.985424
H	5.357597	0.397654	2.218339
H	5.147293	1.762311	1.105577
H	4.704876	1.968399	2.822195

PCMopt/19_TS_RR1

Frequencies, energies and thermodynamic properties:

Lowest Vibrational Mode (1/cm) = -270.4745

2nd Lowest Vibrational Mode (1/cm) = 22.5602

E(RM062X) (a.u.) = -2588.50831026

Thermal correction to Enthalpy (a.u.) = 0.662882

Thermal correction to Gibbs Free Energy (a.u.) = 0.549955

Total Entropy (cal/Kmol) = 237.674

Esp(RM062X+D3) (a.u.) = -2591.11392194

Esp(D3) (a.u.) = -0.0106224075

Esp(RM062X) (a.u.) = -2591.1032995325

Optimised cartesian coordinates (Angstrom):

C	6.562315	2.465695	0.474863
C	5.950776	3.721366	0.350229
C	4.607583	3.830139	0.003850
C	3.891782	2.653660	-0.216471
C	4.506316	1.406662	-0.100349
H	6.533167	4.625168	0.529753
H	4.126278	4.803711	-0.091320
C	2.364753	0.755715	-0.647852
C	1.622831	-1.571151	-0.790765
C	3.076264	-1.848632	-1.213274
C	4.030121	-1.040812	-0.354438
H	5.049036	-1.087132	-0.759641
S	2.201394	2.495011	-0.657462
N	3.614429	0.361967	-0.369326
N	1.363741	-0.114188	-0.861430
C	3.433350	-3.353110	-1.256579
C	3.983100	-3.913058	0.059086
C	4.422121	-3.622060	-2.392826
H	2.501186	-3.891899	-1.493234
H	4.987768	-3.513226	0.267299
H	3.337821	-3.693750	0.920994
H	4.079138	-5.004981	-0.019964
H	4.003637	-3.329322	-3.365950
H	5.357943	-3.061504	-2.240649
H	4.678026	-4.689962	-2.435935
H	3.156999	-1.459736	-2.241453

H	0.995569	-2.025032	-1.565955
C	1.235268	-2.137914	0.558461
C	0.603188	-3.382523	0.622999
C	1.551725	-1.471197	1.746742
C	0.321155	-3.968524	1.854843
H	0.326658	-3.898591	-0.299853
C	1.263584	-2.053461	2.980873
H	2.024433	-0.485935	1.722815
C	0.654659	-3.307163	3.036838
H	-0.172457	-4.940589	1.891635
H	1.515210	-1.524112	3.901222
H	0.430255	-3.763048	4.002099
C	-1.026151	-0.563642	-1.247517
H	-0.870201	-1.624289	-1.077050
C	-2.312344	-0.108134	-1.475915
C	-3.264825	-1.037785	-2.171775
F	-2.983745	-1.077889	-3.485795
F	-3.171145	-2.297801	-1.728721
F	-4.537140	-0.670200	-2.070435
O	-0.071004	1.604921	-1.262801
C	0.028719	0.392330	-1.129278
H	4.052750	-1.390864	0.688369
C	5.851823	1.290413	0.252667
H	6.332237	0.318799	0.360429
H	7.614478	2.404293	0.753381
H	-2.450937	0.941497	-1.749803
C	-4.628902	-3.130948	0.889568
H	-4.272778	-3.457926	1.876092
H	-5.691735	-3.366927	0.776259
H	-4.042030	-3.642334	0.115266
C	-3.268928	-1.230967	0.850379
O	-4.516058	-1.724705	0.753378
O	-2.309036	-1.937504	1.061951
C	-4.350015	1.084445	0.217219
C	-3.224170	0.198847	0.547951
O	-5.522139	0.843896	-0.016676
N	-2.135049	1.007282	1.007189

C -2.397596 2.342557 0.605295
C -1.583601 3.479098 0.594160
C -3.719510 2.431042 0.148613
C -2.140922 4.678390 0.146405
H -0.537003 3.453225 0.882664
C -4.269385 3.628808 -0.295944
C -3.468418 4.768126 -0.292062
H -1.510473 5.568952 0.127050
H -5.305404 3.650827 -0.639197
H -3.862468 5.723787 -0.639187
C -1.433825 0.716951 2.190324
O -1.833592 -0.105160 2.979228
C -0.159484 1.491024 2.425238
H 0.410304 1.631783 1.497455
H -0.391444 2.481582 2.842955
H 0.432651 0.938239 3.162394

PCMopt/19_TS_RSa

Frequencies, energies and thermodynamic properties:

Lowest Vibrational Mode (1/cm) = -129.4721
2nd Lowest Vibrational Mode (1/cm) = 20.3570
E(RM062X) (a.u.) = -2588.52273927
Thermal correction to Enthalpy (a.u.) = 0.663504
Thermal correction to Gibbs Free Energy (a.u.) = 0.548767
Total Entropy (cal/Kmol) = 241.484
Esp(RM062X+D3) (a.u.) = -2591.12996214
Esp(D3) (a.u.) = -0.0100563941
Esp(RM062X) (a.u.) = -2591.1199057459

Optimised cartesian coordinates (Angstrom):

C 7.179263 -2.299631 -1.008662
C 6.665103 -3.600653 -0.901030
C 5.365794 -3.821427 -0.457692
C 4.593918 -2.708888 -0.121351
C 5.111983 -1.417375 -0.219754
H 7.290288 -4.451402 -1.172235
H 4.959658 -4.829685 -0.376491
C 2.984230 -0.949608 0.520203

C	2.106205	1.316476	0.888777
C	3.573978	1.656350	1.203707
C	4.491593	0.974852	0.207397
H	5.538864	1.072005	0.520182
S	2.938784	-2.689048	0.450814
N	4.175209	-0.453603	0.170698
N	1.942272	-0.158993	0.857998
C	3.849301	3.173575	1.328002
C	4.237559	3.856470	0.012613
C	4.928304	3.421329	2.384264
H	2.917718	3.636356	1.692276
H	5.238213	3.535741	-0.316537
H	3.526471	3.658480	-0.801474
H	4.279888	4.944014	0.164306
H	4.622672	3.036574	3.367220
H	5.874043	2.931392	2.103962
H	5.128635	4.497233	2.483314
H	3.770602	1.202744	2.188756
H	1.532936	1.666244	1.754957
C	1.560548	1.965515	-0.365237
C	0.875033	3.180867	-0.260911
C	1.786066	1.415439	-1.631312
C	0.458437	3.856541	-1.405907
H	0.670335	3.607170	0.724546
C	1.358120	2.085632	-2.777797
H	2.301045	0.457046	-1.738706
C	0.703990	3.312435	-2.667227
H	-0.071280	4.805479	-1.311837
H	1.538646	1.645828	-3.759337
H	0.372892	3.837981	-3.563896
C	-0.462084	0.122461	1.351128
H	-0.343530	1.202916	1.351679
C	-1.697559	-0.413226	1.546732
C	-2.745038	0.452891	2.193334
F	-2.439760	0.642999	3.485765
F	-2.820318	1.666866	1.638070
F	-3.966402	-0.074727	2.170772

O	0.632166	-1.975949	1.242865
C	0.671932	-0.766382	1.156738
H	4.389226	1.385959	-0.807842
C	6.413505	-1.189266	-0.671631
H	6.816812	-0.182169	-0.769088
H	8.198265	-2.151733	-1.366465
H	-1.839477	-1.496127	1.612801
C	-0.015231	-2.202663	-1.817495
H	0.655319	-1.331667	-1.881887
H	0.507347	-3.043998	-1.351857
H	-0.338086	-2.464972	-2.834325
C	-1.936658	-0.930332	-1.400207
O	-1.124732	-1.915474	-0.988172
O	-1.710996	-0.297860	-2.414833
C	-3.763149	-1.879367	0.075140
C	-3.057612	-0.737628	-0.496369
O	-3.307848	-2.918800	0.548749
N	-4.038711	0.253685	-0.853925
C	-5.321951	-0.251121	-0.576592
C	-6.589298	0.324782	-0.741274
C	-5.198200	-1.529368	-0.013858
C	-7.696291	-0.426457	-0.343880
H	-6.703745	1.313196	-1.173295
C	-6.309190	-2.266502	0.385408
C	-7.572925	-1.708434	0.212040
H	-8.691480	0.001985	-0.477354
H	-6.170607	-3.256863	0.823381
H	-8.466312	-2.260090	0.507186
C	-3.806973	1.567878	-1.219174
O	-4.726349	2.288712	-1.566842
C	-2.400902	2.112275	-1.161095
H	-1.694975	1.492297	-0.602981
H	-2.026046	2.220187	-2.186756
H	-2.466702	3.109128	-0.706374

PCMopt/19_TS_RSb

Frequencies, energies and thermodynamic properties:

Lowest Vibrational Mode (1/cm) = -173.8028

2nd Lowest Vibrational Mode (1/cm) = 15.5366

E(RM062X) (a.u.) = -2588.52103184

Thermal correction to Enthalpy (a.u.) = 0.663578

Thermal correction to Gibbs Free Energy (a.u.) = 0.549055

Total Entropy (cal/Kmol) = 241.033

Esp(RM062X+D3) (a.u.) = -2591.12481697

Esp(D3) (a.u.) = -0.0100097934

Esp(RM062X) (a.u.) = -2591.1148071766

Optimised cartesian coordinates (Angstrom):

C	6.089008	3.335135	0.901470
C	5.266510	4.462842	0.768511
C	3.956094	4.342067	0.315932
C	3.487937	3.067115	-0.000454
C	4.313168	1.948657	0.124631
H	5.655994	5.447994	1.025581
H	3.312038	5.215753	0.213643
C	2.374059	0.947017	-0.608643
C	2.070098	-1.468710	-0.855968
C	3.570893	-1.453982	-1.211843
C	4.313174	-0.530008	-0.263172
H	5.344382	-0.373233	-0.605375
S	1.897967	2.624363	-0.596519
N	3.652074	0.773844	-0.249517
N	1.564207	-0.079101	-0.907363
C	4.219999	-2.855425	-1.299922
C	4.829769	-3.355789	0.013862
C	5.281032	-2.867617	-2.402563
H	3.424950	-3.557171	-1.598978
H	5.733741	-2.781416	0.269684
H	4.132922	-3.297898	0.861558
H	5.134411	-4.405581	-0.099939
H	4.845591	-2.620189	-3.380783
H	6.077999	-2.138110	-2.188441
H	5.749306	-3.859191	-2.475304
H	3.623255	-1.001819	-2.215727
H	1.563378	-2.002036	-1.670263

C	1.760502	-2.156753	0.457125
C	1.487335	-3.529624	0.453273
C	1.833971	-1.479465	1.677747
C	1.357874	-4.228490	1.652084
H	1.391075	-4.061030	-0.496687
C	1.692887	-2.176560	2.878094
H	1.978879	-0.397228	1.701534
C	1.474163	-3.554202	2.868801
H	1.158646	-5.300966	1.635390
H	1.755487	-1.638593	3.824899
H	1.372654	-4.098410	3.808832
C	-0.733454	-0.900861	-1.272745
H	-0.445849	-1.913014	-0.998768
C	-2.050188	-0.570957	-1.476372
C	-3.030551	-1.604796	-1.941153
F	-2.615463	-2.182922	-3.077108
F	-3.237408	-2.602082	-1.073049
F	-4.217927	-1.052043	-2.214536
O	-0.088290	1.351908	-1.546799
C	0.191649	0.198687	-1.261337
H	4.340836	-0.923331	0.763875
C	5.626715	2.062143	0.582825
H	6.268058	1.189170	0.698240
H	7.110317	3.453099	1.263980
H	-2.280595	0.428887	-1.851592
C	-2.187030	-3.121535	2.210911
H	-3.014452	-3.768193	1.887457
H	-1.229844	-3.636785	2.090394
H	-2.342405	-2.850567	3.264014
C	-3.201735	-1.185991	1.398377
O	-2.116635	-1.962093	1.396114
O	-4.177893	-1.438912	2.071805
C	-1.865163	0.839566	0.829745
C	-3.027694	0.000120	0.542189
O	-0.739790	0.478819	1.166617
N	-4.148533	0.870323	0.305816
C	-3.682934	2.203711	0.281446

C -4.349690 3.408652 0.002043
C -2.315146 2.220593 0.586369
C -3.609667 4.587788 0.064673
H -5.405531 3.424590 -0.241685
C -1.587041 3.407051 0.653910
C -2.243559 4.602590 0.389728
H -4.118008 5.531448 -0.141953
H -0.524966 3.370054 0.901408
H -1.705769 5.550597 0.430847
C -5.474383 0.544046 0.039041
O -6.292653 1.426393 -0.141765
C -5.929777 -0.894476 -0.005286
H -5.144738 -1.633880 -0.149620
H -6.432142 -1.110396 0.946368
H -6.664989 -0.958552 -0.816345

PCMopt/19_TS_RSc

Frequencies, energies and thermodynamic properties:

Lowest Vibrational Mode (1/cm) = -179.5378
2nd Lowest Vibrational Mode (1/cm) = 18.5385
E(RM062X) (a.u.) = -2588.51879620
Thermal correction to Enthalpy (a.u.) = 0.663331
Thermal correction to Gibbs Free Energy (a.u.) = 0.548808
Total Entropy (cal/Kmol) = 241.034
Esp(RM062X+D3) (a.u.) = -2591.12404685
Esp(D3) (a.u.) = -0.0100480803
Esp(RM062X) (a.u.) = -2591.1139987697

Optimised cartesian coordinates (Angstrom):

C 5.923476 3.343666 0.457553
C 5.067109 4.439487 0.278275
C 3.740419 4.254471 -0.098453
C 3.291639 2.948501 -0.293101
C 4.149234 1.860643 -0.120723
H 5.443120 5.450027 0.438653
H 3.068380 5.101876 -0.236576
C 2.202252 0.767536 -0.678012
C 1.947088 -1.668463 -0.754592

C	3.434395	-1.650375	-1.154270
C	4.186438	-0.643444	-0.301933
H	5.203247	-0.495488	-0.687897
S	1.684173	2.429106	-0.764330
N	3.499951	0.646652	-0.367437
N	1.406067	-0.294727	-0.867481
C	4.099803	-3.046937	-1.156255
C	4.733210	-3.451246	0.178958
C	5.142147	-3.126359	-2.273641
H	3.306486	-3.775007	-1.391616
H	5.629132	-2.846196	0.388378
H	4.043054	-3.352704	1.028366
H	5.056143	-4.500483	0.127466
H	4.687906	-2.949126	-3.258555
H	5.937176	-2.378837	-2.124447
H	5.616880	-4.117450	-2.287335
H	3.451326	-1.274784	-2.190372
H	1.425796	-2.253534	-1.523023
C	1.673108	-2.288055	0.599968
C	1.332843	-3.643745	0.665728
C	1.810767	-1.560651	1.784869
C	1.173128	-4.274316	1.898350
H	1.192363	-4.212871	-0.256887
C	1.653009	-2.190791	3.018740
H	2.012823	-0.487759	1.755955
C	1.343998	-3.549809	3.078720
H	0.911358	-5.332548	1.936492
H	1.763722	-1.613607	3.937668
H	1.220770	-4.040585	4.045142
C	-0.868670	-1.211614	-1.072305
H	-0.542572	-2.157716	-0.653218
C	-2.200405	-0.986887	-1.324413
C	-3.054746	-2.222741	-1.488557
F	-2.527673	-3.296004	-0.886048
F	-4.310002	-2.129201	-1.061398
F	-3.127708	-2.518886	-2.799063
O	-0.352384	1.049184	-1.432140

C	0.009158	-0.084700	-1.156384
H	4.253743	-0.956065	0.750873
C	5.479601	2.039560	0.262309
H	6.148273	1.192990	0.413783
H	6.957511	3.512045	0.758933
H	-2.520623	-0.143105	-1.934813
C	-3.929094	-2.833023	2.024188
H	-4.696352	-3.196647	1.326583
H	-3.216150	-3.632545	2.249707
H	-4.419808	-2.491587	2.945593
C	-3.874925	-0.695876	1.073455
O	-3.180615	-1.788564	1.428480
O	-5.058171	-0.574633	1.298012
C	-1.752170	0.685310	1.022700
C	-3.019590	0.301638	0.417686
O	-0.855055	-0.044702	1.439856
N	-3.676315	1.509956	0.000152
C	-2.891497	2.623038	0.358295
C	-3.067999	4.001593	0.169966
C	-1.726677	2.161490	0.991993
C	-2.088111	4.864613	0.664502
H	-3.932784	4.428098	-0.327238
C	-0.759106	3.028432	1.488294
C	-0.944694	4.398422	1.326212
H	-2.231438	5.938587	0.534021
H	0.123932	2.620774	1.984248
H	-0.207663	5.107601	1.705164
C	-4.747491	1.467566	-0.885999
O	-5.033855	0.437754	-1.455681
C	-5.545441	2.729982	-1.099137
H	-5.726320	3.265095	-0.159588
H	-5.021618	3.399338	-1.795727
H	-6.496653	2.436343	-1.554651

PCMopt/19_TS_RSd

Frequencies, energies and thermodynamic properties:

Lowest Vibrational Mode (1/cm) = -128.7309

2nd Lowest Vibrational Mode (1/cm) = 14.7624

E(RM062X) (a.u.) = -2588.51313486

Thermal correction to Enthalpy (a.u.) = 0.663270

Thermal correction to Gibbs Free Energy (a.u.) = 0.547171

Total Entropy (cal/Kmol) = 244.351

Esp(RM062X+D3) (a.u.) = -2591.12210937

Esp(D3) (a.u.) = -0.0098973735

Esp(RM062X) (a.u.) = -2591.1122119965

Optimised cartesian coordinates (Angstrom):

C	6.734150	-2.794928	-1.125791
C	6.093129	-4.032092	-0.967426
C	4.809001	-4.109643	-0.437425
C	4.180556	-2.919844	-0.070660
C	4.825774	-1.691357	-0.222003
H	6.605493	-4.946973	-1.265358
H	4.306393	-5.069142	-0.314101
C	2.812965	-0.993648	0.645267
C	2.172754	1.352463	0.958565
C	3.678788	1.555882	1.215394
C	4.487926	0.758531	0.208067
H	5.550238	0.751763	0.484582
S	2.586445	-2.720223	0.632156
N	4.025277	-0.629407	0.212963
N	1.876156	-0.098852	1.001298
C	4.108539	3.040505	1.283220
C	4.525791	3.642894	-0.062369
C	5.236017	3.209425	2.303803
H	3.238334	3.605399	1.655142
H	5.480133	3.214375	-0.406118
H	3.775819	3.493057	-0.851585
H	4.679971	4.724780	0.053665
H	4.920038	2.888122	3.306103
H	6.118764	2.616388	2.017237
H	5.547466	4.261686	2.362306
H	3.864767	1.112060	2.206993
H	1.653945	1.788385	1.821652
C	1.667587	2.015193	-0.304883

C	1.117211	3.299209	-0.220751
C	1.816436	1.417243	-1.559530
C	0.763356	3.994517	-1.375268
H	0.969422	3.762657	0.757948
C	1.468402	2.116430	-2.716222
H	2.200144	0.397269	-1.646696
C	0.951108	3.408543	-2.627576
H	0.338828	4.996057	-1.295934
H	1.601237	1.647044	-3.692092
H	0.681039	3.952373	-3.533637
C	-0.493510	0.385254	1.462855
H	-0.341222	1.426603	1.189275
C	-1.747365	-0.076201	1.706730
C	-2.817240	0.891707	2.117310
F	-2.654103	1.222867	3.409889
F	-2.787039	2.032499	1.426675
F	-4.049493	0.392355	2.015823
O	0.510845	-1.736604	1.794443
C	0.590054	-0.577091	1.452628
H	4.395830	1.159174	-0.812331
C	6.112174	-1.606691	-0.756660
H	6.611978	-0.648009	-0.890114
H	7.738424	-2.759035	-1.548265
H	-1.897098	-1.113527	2.017522
C	-0.773066	-0.856498	-3.385521
H	-0.404245	-1.891625	-3.387610
H	-1.127504	-0.582524	-4.384502
H	0.046631	-0.192282	-3.082611
C	-1.642059	-0.979267	-1.212704
O	-1.877982	-0.714117	-2.513091
O	-0.566471	-1.397490	-0.837667
C	-3.451946	-1.909692	0.279178
C	-2.826942	-0.783578	-0.376868
O	-2.933303	-2.885573	0.827515
N	-3.856965	0.092165	-0.859839
C	-5.111860	-0.445241	-0.526457
C	-6.421607	0.006206	-0.742676

C	-4.906174	-1.655334	0.160481
C	-7.475092	-0.782818	-0.277125
H	-6.653080	0.927489	-1.266916
C	-5.962886	-2.431012	0.625525
C	-7.264230	-1.990584	0.400819
H	-8.495857	-0.440743	-0.456309
H	-5.750856	-3.362547	1.153857
H	-8.116607	-2.575830	0.747402
C	-3.519442	1.363083	-1.303000
O	-2.356894	1.710846	-1.332405
C	-4.628917	2.283206	-1.743284
H	-5.236781	1.828436	-2.535827
H	-5.283285	2.531296	-0.896308
H	-4.162907	3.199420	-2.118516

PCMopt/19_TS_RSe

Frequencies, energies and thermodynamic properties:

Lowest Vibrational Mode (1/cm) = -150.8183

2nd Lowest Vibrational Mode (1/cm) = 18.7599

E(RM062X) (a.u.) = -2588.51323117

Thermal correction to Enthalpy (a.u.) = 0.663380

Thermal correction to Gibbs Free Energy (a.u.) = 0.548565

Total Entropy (cal/Kmol) = 241.649

Esp(RM062X+D3) (a.u.) = -2591.12113274

Esp(D3) (a.u.) = -0.0098587244

Esp(RM062X) (a.u.) = -2591.1112740156

Optimised cartesian coordinates (Angstrom):

C	7.320234	-2.154815	-0.778687
C	6.825906	-3.467910	-0.776813
C	5.499780	-3.732041	-0.451973
C	4.679977	-2.650708	-0.127635
C	5.177538	-1.347555	-0.121823
H	7.488589	-4.293581	-1.036297
H	5.109436	-4.749931	-0.451950
C	2.989597	-0.952441	0.470508
C	2.034056	1.278564	0.780766
C	3.448057	1.663396	1.246878

C	4.491766	1.010634	0.362309
H	5.489526	1.100808	0.810900
S	2.984519	-2.686716	0.310847
N	4.194903	-0.418544	0.239578
N	1.909352	-0.199638	0.766932
C	3.647847	3.190165	1.395882
C	4.123396	3.898389	0.123806
C	4.610470	3.481096	2.549008
H	2.664870	3.608463	1.669852
H	5.160212	3.618505	-0.119745
H	3.492028	3.681699	-0.749025
H	4.111533	4.985211	0.285857
H	4.233698	3.074603	3.497997
H	5.600677	3.039197	2.356308
H	4.748727	4.564542	2.670075
H	3.548631	1.218305	2.250250
H	1.368441	1.633781	1.572140
C	1.601098	1.881010	-0.538708
C	0.746252	2.985960	-0.536729
C	2.091303	1.400046	-1.760077
C	0.408111	3.621116	-1.731230
H	0.336650	3.355206	0.406525
C	1.754404	2.034369	-2.954320
H	2.736579	0.518723	-1.796893
C	0.919122	3.152620	-2.940715
H	-0.263503	4.480336	-1.714873
H	2.144322	1.651660	-3.898276
H	0.655872	3.648977	-3.875669
C	-0.499675	-0.000442	1.295386
H	-0.416765	1.077799	1.385091
C	-1.719450	-0.574798	1.478104
C	-2.724003	0.145819	2.339175
F	-2.605674	-0.327425	3.593570
F	-2.515705	1.459135	2.407379
F	-3.998602	-0.038305	1.996783
O	0.631641	-2.061672	1.007834
C	0.645560	-0.847259	1.011719

H	4.522843	1.449314	-0.645916
C	6.506593	-1.075509	-0.452494
H	6.895116	-0.057992	-0.463856
H	8.362102	-1.972550	-1.042459
H	-1.841172	-1.661096	1.421597
C	0.049751	-1.772505	-2.036171
H	0.680023	-0.882583	-1.887494
H	0.589135	-2.672798	-1.724031
H	-0.211476	-1.832044	-3.102271
C	-1.913629	-0.643616	-1.439874
O	-1.109589	-1.701707	-1.230092
O	-1.642906	0.209297	-2.260735
C	-3.768343	-1.895878	-0.206090
C	-3.085074	-0.677664	-0.585837
O	-3.309354	-2.998459	0.097196
N	-4.061661	0.356077	-0.782052
C	-5.345184	-0.191826	-0.597764
C	-6.628542	0.364454	-0.694606
C	-5.208056	-1.545213	-0.242927
C	-7.725682	-0.462425	-0.445541
H	-6.806843	1.398317	-0.971989
C	-6.307358	-2.359486	0.008989
C	-7.582873	-1.811689	-0.096746
H	-8.726166	-0.035503	-0.534241
H	-6.148114	-3.404584	0.281866
H	-8.467151	-2.422467	0.088168
C	-3.691888	1.692397	-0.653636
O	-2.572915	1.993294	-0.299372
C	-4.721477	2.744931	-0.985035
H	-5.258075	2.514001	-1.913025
H	-5.450628	2.836205	-0.167592
H	-4.192263	3.697879	-1.086641

PCMopt/19_TS_RSf

Frequencies, energies and thermodynamic properties:

Lowest Vibrational Mode (1/cm) = -189.5664

2nd Lowest Vibrational Mode (1/cm) = 16.8288

E(RM062X) (a.u.) = -2588.51324270

Thermal correction to Enthalpy (a.u.) = 0.663251

Thermal correction to Gibbs Free Energy (a.u.) = 0.548269

Total Entropy (cal/Kmol) = 242.000

Esp(RM062X+D3) (a.u.) = -2591.12088703

Esp(D3) (a.u.) = -0.0104287436

Esp(RM062X) (a.u.) = -2591.1104582864

Optimised cartesian coordinates (Angstrom):

C	6.331938	3.026551	0.166766
C	5.590574	4.208155	0.312558
C	4.206593	4.207017	0.170474
C	3.582325	2.993599	-0.117023
C	4.324369	1.821831	-0.271665
H	6.103982	5.141660	0.543179
H	3.625951	5.122610	0.283763
C	2.200661	1.003162	-0.599859
C	1.677468	-1.369141	-0.870793
C	3.031826	-1.427963	-1.601333
C	4.063366	-0.601339	-0.855624
H	4.958972	-0.451404	-1.472912
S	1.873465	2.698551	-0.369512
N	3.507955	0.723852	-0.568140
N	1.261939	0.050486	-0.750544
C	3.512418	-2.868451	-1.898195
C	4.353964	-3.504944	-0.787526
C	4.283013	-2.898553	-3.219894
H	2.604005	-3.479830	-2.029147
H	5.337891	-3.016645	-0.709282
H	3.868404	-3.464519	0.197482
H	4.536063	-4.561374	-1.029255
H	3.660946	-2.546475	-4.054628
H	5.179015	-2.260192	-3.167779
H	4.616612	-3.920655	-3.447155
H	2.855762	-0.933861	-2.570709
H	0.948970	-1.832745	-1.545421
C	1.653518	-2.098708	0.455171
C	1.094195	-3.379994	0.510910

C	2.262059	-1.571838	1.599868
C	1.179860	-4.140555	1.676580
H	0.597730	-3.792893	-0.371147
C	2.359757	-2.336391	2.761125
H	2.663047	-0.555462	1.601717
C	1.827997	-3.625452	2.799090
H	0.742852	-5.139494	1.704540
H	2.844687	-1.917298	3.644151
H	1.904576	-4.221958	3.709238
C	-1.085387	-0.644842	-0.837977
H	-0.801227	-1.608859	-0.420428
C	-2.358386	-0.451420	-1.297291
C	-3.164768	-1.667140	-1.655787
F	-2.822196	-2.085941	-2.887564
F	-2.937504	-2.689331	-0.829968
F	-4.478031	-1.449598	-1.690878
O	-0.366071	1.609815	-1.075632
C	-0.123800	0.431387	-0.895823
H	4.368374	-1.067369	0.092529
C	5.712565	1.816551	-0.127996
H	6.292821	0.900610	-0.232750
H	7.414939	3.052345	0.287984
H	-2.617373	0.455803	-1.843359
C	-5.492246	2.176119	-2.371322
H	-6.038949	2.916091	-1.770141
H	-4.991455	2.669480	-3.210210
H	-6.201780	1.424898	-2.742957
C	-4.887240	0.941735	-0.476523
O	-4.475158	1.570024	-1.594346
O	-6.057220	0.721791	-0.266517
C	-3.815171	-0.671687	1.225110
C	-3.775005	0.492084	0.373662
O	-4.620461	-1.604002	1.266909
N	-2.726488	1.369705	0.804228
C	-1.922303	0.645823	1.707952
C	-0.689100	0.950383	2.307044
C	-2.557188	-0.573852	1.997993

C -0.146395 0.020464 3.190338
H -0.137061 1.860494 2.088001
C -1.992681 -1.503203 2.869913
C -0.781142 -1.198112 3.478681
H 0.815463 0.244527 3.655133
H -2.512687 -2.444633 3.058119
H -0.310446 -1.904131 4.163682
C -2.875533 2.752827 0.769696
O -3.920151 3.255842 0.407635
C -1.698966 3.597141 1.182513
H -1.515542 3.511656 2.262311
H -0.797231 3.286056 0.641081
H -1.943463 4.637066 0.944165

PCMopt/19_TS_RSg

Frequencies, energies and thermodynamic properties:

Lowest Vibrational Mode (1/cm) = -258.6653
2nd Lowest Vibrational Mode (1/cm) = 16.3957
E(RM062X) (a.u.) = -2588.51597833
Thermal correction to Enthalpy (a.u.) = 0.663105
Thermal correction to Gibbs Free Energy (a.u.) = 0.548236
Total Entropy (cal/Kmol) = 241.763
Esp(RM062X+D3) (a.u.) = -2591.12075261
Esp(D3) (a.u.) = -0.0103688943
Esp(RM062X) (a.u.) = -2591.1103837157

Optimised cartesian coordinates (Angstrom):

C 6.308107 2.959844 0.272148
C 5.566293 4.141472 0.410554
C 4.186240 4.145381 0.230202
C 3.564914 2.938333 -0.088217
C 4.309186 1.766140 -0.235209
H 6.075253 5.071108 0.665655
H 3.605784 5.061864 0.338152
C 2.192155 0.962882 -0.636384
C 1.659234 -1.402196 -0.916421
C 3.031274 -1.474808 -1.614713
C 4.051078 -0.651821 -0.847134

H	4.960803	-0.507887	-1.445300
S	1.859851	2.654677	-0.385277
N	3.498288	0.675520	-0.568684
N	1.256195	0.019770	-0.817672
C	3.510116	-2.918449	-1.897638
C	4.323354	-3.557703	-0.767686
C	4.309175	-2.956532	-3.202199
H	2.601185	-3.524952	-2.046828
H	5.308165	-3.074872	-0.668866
H	3.816346	-3.511701	0.206250
H	4.504520	-4.615813	-1.002951
H	3.707176	-2.603747	-4.051284
H	5.206764	-2.322059	-3.132457
H	4.642890	-3.980799	-3.419618
H	2.880779	-0.982007	-2.589002
H	0.939610	-1.863473	-1.602858
C	1.600886	-2.131471	0.409113
C	1.033947	-3.410461	0.449198
C	2.188517	-1.612470	1.567990
C	1.091949	-4.176470	1.612624
H	0.553143	-3.816067	-0.444664
C	2.258609	-2.382887	2.727861
H	2.593340	-0.597582	1.581797
C	1.719408	-3.668940	2.750556
H	0.649345	-5.173266	1.627579
H	2.728782	-1.970609	3.622036
H	1.775020	-4.269423	3.659603
C	-1.103924	-0.614660	-0.903888
H	-0.844951	-1.563621	-0.438353
C	-2.399507	-0.362455	-1.304386
C	-3.273157	-1.544108	-1.611091
F	-2.966782	-2.039463	-2.822298
F	-3.108226	-2.540429	-0.739710
F	-4.573963	-1.245014	-1.651322
O	-0.317467	1.571161	-1.395684
C	-0.121924	0.415993	-1.061752
H	4.333908	-1.121269	0.106309

C	5.692505	1.755071	-0.053007
H	6.272259	0.838119	-0.152666
H	7.387539	2.980686	0.422927
H	-2.591829	0.511543	-1.930164
C	-6.820572	1.187924	-1.240116
H	-6.597968	0.657842	-2.176258
H	-7.753896	0.817127	-0.806263
H	-6.898849	2.262601	-1.450220
C	-4.564170	1.253625	-0.645327
O	-5.806107	0.933987	-0.279206
O	-4.334238	1.854370	-1.676137
C	-3.784716	-0.415171	1.185188
C	-3.553543	0.716601	0.280822
O	-4.714860	-1.211468	1.227749
N	-2.417879	1.453371	0.759766
C	-1.753542	0.639760	1.698339
C	-0.533411	0.827605	2.369361
C	-2.551903	-0.475092	1.990668
C	-0.162360	-0.132728	3.306265
H	0.098981	1.683983	2.157335
C	-2.158110	-1.437568	2.919563
C	-0.949645	-1.263226	3.582583
H	0.785927	-0.006355	3.831884
H	-2.804440	-2.296641	3.109937
H	-0.604193	-1.998798	4.310241
C	-2.090451	2.798379	0.615282
O	-1.008576	3.206158	0.987207
C	-3.120144	3.749531	0.062867
H	-3.161386	3.678431	-1.029678
H	-4.124595	3.541362	0.452948
H	-2.803735	4.755349	0.357783

PCMopt/19_TS_RSh

Frequencies, energies and thermodynamic properties:

Lowest Vibrational Mode (1/cm) = -153.4421

2nd Lowest Vibrational Mode (1/cm) = 16.1708

E(RM062X) (a.u.) = -2588.51435193

Thermal correction to Enthalpy (a.u.) = 0.663232

Thermal correction to Gibbs Free Energy (a.u.) = 0.548013

Total Entropy (cal/Kmol) = 242.499

Esp(RM062X+D3) (a.u.) = -2591.11987098

Esp(D3) (a.u.) = -0.0097914840

Esp(RM062X) (a.u.) = -2591.1100794960

Optimised cartesian coordinates (Angstrom):

C	6.119146	3.219501	1.160584
C	5.322886	4.361877	0.997169
C	4.038507	4.267955	0.469549
C	3.569528	3.005053	0.109741
C	4.369094	1.871746	0.265545
H	5.712028	5.337218	1.289764
H	3.414414	5.152882	0.343169
C	2.459001	0.911443	-0.589047
C	2.130650	-1.494961	-0.881341
C	3.648281	-1.502448	-1.153356
C	4.352293	-0.601784	-0.154192
H	5.403123	-0.458610	-0.437786
S	2.009574	2.595606	-0.580975
N	3.712483	0.712777	-0.161463
N	1.651662	-0.095972	-0.946742
C	4.276530	-2.914410	-1.222840
C	4.798741	-3.444145	0.116692
C	5.400495	-2.931494	-2.261228
H	3.487832	-3.597146	-1.577940
H	5.694978	-2.889255	0.435307
H	4.053141	-3.386900	0.921573
H	5.092749	-4.497158	0.004590
H	5.028302	-2.662342	-3.259620
H	6.196366	-2.220074	-1.989556
H	5.854487	-3.930563	-2.321001
H	3.764796	-1.040498	-2.147371
H	1.660704	-2.011263	-1.728187
C	1.733377	-2.188716	0.404539
C	1.411721	-3.550189	0.367578
C	1.756225	-1.525311	1.634564

C	1.163680	-4.250906	1.546367
H	1.359171	-4.069394	-0.592564
C	1.512016	-2.227258	2.815077
H	1.938640	-0.449706	1.679597
C	1.225964	-3.592109	2.775032
H	0.918252	-5.312895	1.504336
H	1.536967	-1.701289	3.770395
H	1.035173	-4.138393	3.699812
C	-0.646550	-0.875925	-1.403344
H	-0.380593	-1.897680	-1.143217
C	-1.950428	-0.523595	-1.613730
C	-2.963466	-1.519414	-2.097061
F	-2.578210	-2.021995	-3.286663
F	-3.147048	-2.564443	-1.297636
F	-4.146919	-0.940079	-2.312235
O	0.058773	1.362987	-1.672814
C	0.301179	0.209714	-1.359740
H	4.317342	-1.006589	0.868195
C	5.656087	1.958355	0.798465
H	6.276043	1.073482	0.937429
H	7.119919	3.316179	1.581906
H	-2.167619	0.495969	-1.940504
C	-4.069078	-2.350287	3.053326
H	-3.148132	-2.586841	3.602945
H	-4.885436	-2.147436	3.753392
H	-4.326502	-3.205372	2.412613
C	-2.947272	-1.228382	1.334134
O	-3.906635	-1.177776	2.273321
O	-2.161487	-2.141536	1.268924
C	-1.753854	0.865765	0.721629
C	-2.887660	0.000531	0.518032
O	-0.599287	0.539917	1.022451
N	-4.047671	0.804984	0.302695
C	-3.645409	2.150020	0.197681
C	-4.350564	3.323968	-0.115610
C	-2.260974	2.223064	0.443052
C	-3.649120	4.527902	-0.132345

H -5.411994 3.337769 -0.338024
C -1.572276 3.434372 0.427362
C -2.274341 4.598245 0.139909
H -4.196635 5.442740 -0.365082
H -0.500000 3.439088 0.629397
H -1.765305 5.562420 0.117233
C -5.296514 0.226003 0.093269
O -5.414365 -0.979133 0.059064
C -6.486722 1.136825 -0.067262
H -6.536459 1.886344 0.732167
H -6.447188 1.654112 -1.035756
H -7.380497 0.505938 -0.041005

PCMopt/19_TS_RSi

Frequencies, energies and thermodynamic properties:

Lowest Vibrational Mode (1/cm) = -212.8751
2nd Lowest Vibrational Mode (1/cm) = 17.8263
E(RM062X) (a.u.) = -2588.51323710
Thermal correction to Enthalpy (a.u.) = 0.663096
Thermal correction to Gibbs Free Energy (a.u.) = 0.548970
Total Entropy (cal/Kmol) = 240.198
Esp(RM062X+D3) (a.u.) = -2591.12038904
Esp(D3) (a.u.) = -0.0103716212
Esp(RM062X) (a.u.) = -2591.1100174188

Optimised cartesian coordinates (Angstrom):

C 6.476514 2.854871 0.093134
C 5.777949 4.068184 0.173813
C 4.397088 4.111105 0.008421
C 3.732382 2.909954 -0.236004
C 4.432138 1.705832 -0.325793
H 6.322321 4.991635 0.371598
H 3.849269 5.051458 0.071648
C 2.283214 0.951416 -0.651173
C 1.674298 -1.407392 -0.830124
C 3.037956 -1.549089 -1.531703
C 4.087493 -0.730496 -0.802434
H 4.999931 -0.644465 -1.407245

S	2.017127	2.667512	-0.503151
N	3.579408	0.626361	-0.586047
N	1.312348	0.029719	-0.773739
C	3.467820	-3.018132	-1.757942
C	4.271375	-3.634071	-0.608373
C	4.253047	-3.135939	-3.065960
H	2.539003	-3.601107	-1.873951
H	5.271244	-3.178067	-0.537760
H	3.774945	-3.531758	0.366634
H	4.417966	-4.706156	-0.800143
H	3.654527	-2.800485	-3.924436
H	5.170536	-2.527712	-3.029977
H	4.552657	-4.178462	-3.242144
H	2.897774	-1.090796	-2.524199
H	0.939537	-1.869366	-1.499091
C	1.598910	-2.081613	0.522904
C	0.995465	-3.340596	0.617165
C	2.201727	-1.529505	1.658677
C	1.032470	-4.056429	1.813136
H	0.502692	-3.771682	-0.258324
C	2.250478	-2.250164	2.850916
H	2.636029	-0.527152	1.629077
C	1.675176	-3.518412	2.928156
H	0.561509	-5.038506	1.871168
H	2.731609	-1.812512	3.726990
H	1.713572	-4.080275	3.862450
C	-1.057737	-0.577338	-0.853696
H	-0.808129	-1.539766	-0.412224
C	-2.331692	-0.351576	-1.314573
C	-3.154461	-1.563042	-1.656141
F	-2.787712	-2.034838	-2.860482
F	-2.975536	-2.558664	-0.786440
F	-4.463235	-1.321066	-1.736033
O	-0.252288	1.636063	-1.180908
C	-0.061218	0.455793	-0.947976
H	4.355470	-1.165074	0.171521
C	5.816687	1.656266	-0.158084

H	6.363956	0.715881	-0.211843
H	7.557745	2.846146	0.231494
H	-2.549655	0.538685	-1.907791
C	-6.876483	0.954222	-1.460207
H	-6.575497	0.440060	-2.383586
H	-7.812883	0.533579	-1.080988
H	-7.001714	2.023444	-1.676916
C	-4.663847	1.149156	-0.743020
O	-5.906731	0.753433	-0.445008
O	-4.407134	1.747930	-1.766116
C	-3.828399	-0.449511	1.147631
C	-3.683444	0.680148	0.246440
O	-4.682006	-1.332149	1.192876
N	-2.613093	1.516046	0.702222
C	-1.883278	0.777951	1.656381
C	-0.657335	1.035215	2.290650
C	-2.595169	-0.393244	1.963608
C	-0.199239	0.107285	3.222616
H	-0.047991	1.905528	2.062306
C	-2.115606	-1.322677	2.884730
C	-0.910014	-1.064691	3.526270
H	0.756993	0.293708	3.714644
H	-2.694561	-2.226657	3.084070
H	-0.502652	-1.772037	4.249465
C	-2.699892	2.907377	0.654043
O	-3.718528	3.453560	0.288537
C	-1.484647	3.698245	1.061218
H	-1.328076	3.639579	2.147128
H	-0.591546	3.321229	0.548957
H	-1.667750	4.741858	0.786144

PCMopt/19_TS_RSj

Frequencies, energies and thermodynamic properties:

Lowest Vibrational Mode (1/cm) =	-232.2385
2nd Lowest Vibrational Mode (1/cm) =	18.7349
E(RM062X) (a.u.) =	-2588.51373980
Thermal correction to Enthalpy (a.u.) =	0.663170

Thermal correction to Gibbs Free Energy (a.u.) = 0.548378

Total Entropy (cal/Kmol) = 241.600

Esp(RM062X+D3) (a.u.) = -2591.11940275

Esp(D3) (a.u.) = -0.0103976481

Esp(RM062X) (a.u.) = -2591.1090051019

Optimised cartesian coordinates (Angstrom):

C	6.201210	3.076524	0.355622
C	5.431598	4.228890	0.569820
C	4.048423	4.204530	0.418267
C	3.452335	2.998517	0.050828
C	4.223995	1.856424	-0.171939
H	5.921547	5.157829	0.861938
H	3.446778	5.098192	0.585257
C	2.121377	1.016826	-0.570462
C	1.648053	-1.346246	-0.958732
C	3.003214	-1.343201	-1.692126
C	4.018843	-0.531603	-0.907485
H	4.907838	-0.328868	-1.519611
S	1.749736	2.682882	-0.225629
N	3.435371	0.762464	-0.545434
N	1.206464	0.057142	-0.781557
C	3.513312	-2.756001	-2.062432
C	4.369085	-3.432405	-0.986880
C	4.282635	-2.702604	-3.384179
H	2.617132	-3.378011	-2.224318
H	5.343173	-2.929134	-0.883834
H	3.884226	-3.452555	-0.000964
H	4.571940	-4.471080	-1.283166
H	3.652402	-2.321243	-4.199740
H	5.164970	-2.048847	-3.299505
H	4.637462	-3.704425	-3.663732
H	2.814262	-0.805087	-2.635236
H	0.926294	-1.795516	-1.650685
C	1.642963	-2.140002	0.330237
C	1.102221	-3.430684	0.322155
C	2.252988	-1.666786	1.497245
C	1.207628	-4.251746	1.444079

H	0.604200	-3.802147	-0.577081
C	2.371193	-2.491957	2.614651
H	2.638304	-0.645722	1.550882
C	1.857508	-3.788362	2.588046
H	0.784487	-5.256819	1.421545
H	2.859015	-2.114884	3.514851
H	1.950436	-4.431636	3.464175
C	-1.137632	-0.642697	-0.889333
H	-0.854432	-1.605187	-0.466741
C	-2.429889	-0.406690	-1.290949
C	-3.294629	-1.580529	-1.640155
F	-2.986284	-2.026453	-2.871100
F	-3.122474	-2.609987	-0.810037
F	-4.594501	-1.283172	-1.668998
O	-0.417281	1.590996	-1.250059
C	-0.181274	0.426081	-0.989250
H	4.340608	-1.040802	0.012638
C	5.610847	1.873661	-0.018807
H	6.212116	0.979167	-0.177366
H	7.282712	3.119248	0.485441
H	-2.647298	0.497825	-1.859269
C	-5.350025	2.405383	-2.273783
H	-5.937561	3.112447	-1.671637
H	-4.844657	2.929901	-3.090245
H	-6.023280	1.635719	-2.672986
C	-4.738665	1.143076	-0.391329
O	-4.327874	1.810760	-1.486877
O	-5.911894	0.979159	-0.155526
C	-3.832160	-0.611742	1.219925
C	-3.635895	0.581425	0.406388
O	-4.761919	-1.413680	1.220304
N	-2.498344	1.298946	0.907749
C	-1.806294	0.430332	1.775418
C	-0.580257	0.591396	2.442145
C	-2.583063	-0.716945	1.995943
C	-0.178431	-0.428026	3.300780
H	0.033354	1.473214	2.285690

C	-2.156354	-1.738432	2.843824
C	-0.940387	-1.591762	3.500242
H	0.773832	-0.322162	3.823294
H	-2.784063	-2.621286	2.979945
H	-0.570754	-2.372922	4.165937
C	-2.197968	2.654519	0.854973
O	-1.117654	3.059137	1.235810
C	-3.250796	3.627555	0.382402
H	-3.203822	3.732550	-0.707921
H	-4.268079	3.330747	0.665835
H	-3.005302	4.590708	0.842441

PCMopt/19_TS_RSk

Frequencies, energies and thermodynamic properties:

Lowest Vibrational Mode (1/cm) = -316.5823

2nd Lowest Vibrational Mode (1/cm) = 20.5750

E(RM062X) (a.u.) = -2588.51248008

Thermal correction to Enthalpy (a.u.) = 0.663053

Thermal correction to Gibbs Free Energy (a.u.) = 0.549598

Total Entropy (cal/Kmol) = 238.787

Esp(RM062X+D3) (a.u.) = -2591.11477816

Esp(D3) (a.u.) = -0.0098081077

Esp(RM062X) (a.u.) = -2591.1049700523

Optimised cartesian coordinates (Angstrom):

C	6.399048	3.019042	0.926780
C	5.641956	4.195750	0.842055
C	4.313079	4.161328	0.429161
C	3.759858	2.923747	0.103139
C	4.520345	1.756083	0.179686
H	6.097268	5.150632	1.105253
H	3.719034	5.073313	0.363918
C	2.503768	0.886949	-0.518834
C	2.049135	-1.495866	-0.809611
C	3.541331	-1.574228	-1.186839
C	4.357148	-0.711717	-0.240698
H	5.390442	-0.616476	-0.598908
S	2.126552	2.590372	-0.448661

N	3.779896	0.630731	-0.197786
N	1.629317	-0.078675	-0.821699
C	4.088888	-3.016631	-1.299206
C	4.671154	-3.578169	0.002099
C	5.135221	-3.091480	-2.413128
H	3.241758	-3.655174	-1.597814
H	5.613412	-3.070887	0.261953
H	3.983551	-3.487126	0.854443
H	4.903177	-4.644215	-0.130586
H	4.709104	-2.799617	-3.383227
H	5.985503	-2.424996	-2.198793
H	5.528943	-4.113551	-2.503987
H	3.610240	-1.115448	-2.186741
H	1.496622	-1.976948	-1.626541
C	1.703219	-2.186511	0.492828
C	1.288481	-3.522513	0.463772
C	1.855126	-1.545819	1.725758
C	1.071441	-4.222090	1.649450
H	1.137298	-4.021914	-0.496504
C	1.639632	-2.245623	2.912997
H	2.122480	-0.487681	1.771285
C	1.257675	-3.586848	2.878001
H	0.752090	-5.264491	1.613316
H	1.764239	-1.736699	3.869716
H	1.089864	-4.132141	3.807763
C	-0.714051	-0.749996	-1.158739
H	-0.475465	-1.790077	-0.956082
C	-2.046167	-0.353011	-1.286814
C	-2.986861	-1.344775	-1.915234
F	-2.750008	-1.414377	-3.234857
F	-2.821406	-2.585601	-1.438122
F	-4.275174	-1.027353	-1.788356
O	0.041523	1.475400	-1.336017
C	0.255009	0.287127	-1.121725
H	4.377944	-1.120414	0.780659
C	5.851505	1.782490	0.598081
H	6.443349	0.871186	0.674888

H	7.436389	3.068457	1.258365
H	-2.232415	0.662172	-1.652289
C	-4.301743	-3.270385	1.136440
H	-4.164030	-3.406920	2.217075
H	-5.307195	-3.583710	0.838822
H	-3.544809	-3.857314	0.600227
C	-3.031832	-1.301413	1.083465
O	-4.193712	-1.898653	0.787474
O	-2.120968	-1.887507	1.618030
C	-1.815209	0.954512	0.980704
C	-2.940478	0.082115	0.593311
O	-0.724668	0.617287	1.415930
N	-4.045465	0.922583	0.243925
C	-3.559062	2.236044	0.096784
C	-4.210290	3.389159	-0.365769
C	-2.238935	2.305284	0.563317
C	-3.500143	4.586124	-0.331409
H	-5.228297	3.345648	-0.743521
C	-1.538395	3.510867	0.589309
C	-2.176688	4.659166	0.138382
H	-3.988599	5.493877	-0.690287
H	-0.508506	3.526266	0.950418
H	-1.655306	5.616995	0.137924
C	-5.435175	0.724064	0.374195
O	-6.184280	1.289684	-0.388214
C	-5.972498	-0.029334	1.567793
H	-6.439149	-0.967648	1.246646
H	-5.212968	-0.242875	2.324988
H	-6.753861	0.614989	1.992492

PCMopt/19_TS_RSl

Frequencies, energies and thermodynamic properties:

Lowest Vibrational Mode (1/cm) = -269.5941

2nd Lowest Vibrational Mode (1/cm) = 11.8346

E(RM062X) (a.u.) = -2588.50423573

Thermal correction to Enthalpy (a.u.) = 0.662701

Thermal correction to Gibbs Free Energy (a.u.) = 0.547480

Total Entropy (cal/Kmol) = 242.503

Esp(RM062X+D3) (a.u.) = -2591.10894562

Esp(D3) (a.u.) = -0.0102653301

Esp(RM062X) (a.u.) = -2591.0986802899

Optimised cartesian coordinates (Angstrom):

C	7.392753	-1.468930	-0.434977
C	7.057697	-2.820701	-0.601456
C	5.746525	-3.256578	-0.441149
C	4.778760	-2.308561	-0.108731
C	5.117970	-0.966813	0.065671
H	7.833454	-3.540282	-0.863487
H	5.479937	-4.305371	-0.573339
C	2.851016	-0.869806	0.459934
C	1.620049	1.199908	0.861453
C	2.927407	1.691870	1.508122
C	4.120500	1.241409	0.688262
H	5.052024	1.391228	1.249307
S	3.063190	-2.570268	0.137132
N	4.004080	-0.190331	0.405766
N	1.672614	-0.272331	0.720214
C	2.938241	3.210377	1.803079
C	3.464265	4.079671	0.656591
C	3.737006	3.488653	3.078181
H	1.891828	3.499381	1.996255
H	4.547046	3.935240	0.517706
H	2.963064	3.879110	-0.300656
H	3.308262	5.139500	0.901725
H	3.310470	2.959977	3.942077
H	4.784412	3.167818	2.963687
H	3.742066	4.564591	3.301830
H	2.980385	1.166920	2.475907
H	0.839154	1.411220	1.596433
C	1.267400	1.878000	-0.445762
C	0.345520	2.928068	-0.441397
C	1.920847	1.551239	-1.642345
C	0.118053	3.676820	-1.596240
H	-0.197407	3.170981	0.475182

C	1.696299	2.300605	-2.796172
H	2.622289	0.714197	-1.685712
C	0.805871	3.376114	-2.770397
H	-0.610353	4.487631	-1.577082
H	2.219172	2.042326	-3.717987
H	0.632706	3.963454	-3.673178
C	-0.754260	-0.432950	1.163856
H	-0.835468	0.630560	1.368866
C	-1.881302	-1.222894	1.273078
C	-2.919207	-0.747556	2.254854
F	-2.408185	-0.788081	3.497165
F	-3.289796	0.517270	2.041932
F	-4.016289	-1.501789	2.290733
O	0.602450	-2.274912	0.605943
C	0.475140	-1.075058	0.805720
H	4.199678	1.780072	-0.267523
C	6.430887	-0.521761	-0.099842
H	6.697823	0.527519	0.019127
H	8.425996	-1.149549	-0.572026
H	-1.784094	-2.310710	1.219268
C	0.200145	-1.718525	-2.390887
H	-0.181492	-2.180602	-3.311658
H	0.910139	-0.919830	-2.635444
H	0.682209	-2.486715	-1.772549
C	-1.906850	-1.879713	-1.374942
O	-0.855848	-1.100198	-1.672395
O	-1.888956	-3.075537	-1.571942
C	-4.241885	-1.862426	-0.380099
C	-3.003487	-1.156367	-0.733458
O	-4.424939	-3.061346	-0.219626
N	-3.311407	0.243229	-0.872824
C	-4.633481	0.444440	-0.423167
C	-5.370746	1.625977	-0.261050
C	-5.235108	-0.792318	-0.164609
C	-6.697669	1.510935	0.150372
H	-4.922888	2.599433	-0.434661
C	-6.557770	-0.896634	0.258223

C -7.298426 0.269669 0.414167
H -7.282141 2.423620 0.281088
H -6.982220 -1.882452 0.457842
H -8.336544 0.227811 0.745529
C -2.641370 1.304650 -1.501833
O -2.799647 2.432309 -1.079785
C -1.886969 1.080551 -2.789710
H -2.165534 0.150015 -3.293450
H -2.124957 1.945263 -3.422406
H -0.806035 1.080640 -2.602949

PCMopt/19_TS_SRa

Frequencies, energies and thermodynamic properties:

Lowest Vibrational Mode (1/cm) = -122.3676
2nd Lowest Vibrational Mode (1/cm) = 17.4758
E(RM062X) (a.u.) = -2588.52559975
Thermal correction to Enthalpy (a.u.) = 0.663404
Thermal correction to Gibbs Free Energy (a.u.) = 0.549077
Total Entropy (cal/Kmol) = 240.622
Esp(RM062X+D3) (a.u.) = -2591.13326489
Esp(D3) (a.u.) = -0.0108409848
Esp(RM062X) (a.u.) = -2591.1224239052

Optimised cartesian coordinates (Angstrom):

C -6.975029 1.901635 0.529154
C -6.699817 3.179836 0.020419
C -5.424148 3.513540 -0.421137
C -4.429495 2.538769 -0.338735
C -4.704749 1.271090 0.175540
H -7.496643 3.921736 -0.033356
H -5.205651 4.503802 -0.821012
C -2.477109 1.029523 -0.340241
C -1.177128 -1.028957 0.003918
C -2.176677 -1.277819 1.144745
C -3.579801 -0.911434 0.699815
H -4.271774 -0.928306 1.550999
S -2.752848 2.679303 -0.825042
N -3.569081 0.453762 0.171185

N	-1.308820	0.368604	-0.472930
C	-2.090403	-2.703923	1.737269
C	-3.010355	-3.728757	1.066641
C	-2.360300	-2.660614	3.242404
H	-1.051063	-3.041161	1.594732
H	-4.067676	-3.517541	1.290683
H	-2.888231	-3.761808	-0.025169
H	-2.789919	-4.731097	1.459756
H	-1.639920	-2.012789	3.763053
H	-3.370959	-2.275084	3.450136
H	-2.292433	-3.668153	3.676536
H	-1.880010	-0.564435	1.930048
H	-0.183615	-1.087429	0.463079
C	-1.252357	-2.019681	-1.136381
C	-0.382523	-3.116504	-1.132780
C	-2.197967	-1.897271	-2.159101
C	-0.483051	-4.097907	-2.117410
H	0.379327	-3.199989	-0.350831
C	-2.297641	-2.879375	-3.144975
H	-2.862055	-1.029910	-2.201543
C	-1.446984	-3.984848	-3.120512
H	0.197422	-4.950238	-2.104319
H	-3.040911	-2.777245	-3.936646
H	-1.526230	-4.752073	-3.891785
C	1.028940	0.269248	-1.255316
H	1.006086	-0.818606	-1.284826
C	2.197235	0.930731	-1.487534
C	3.277775	0.263177	-2.286743
F	2.941177	0.304678	-3.590496
F	4.454396	0.862847	-2.187094
F	3.427603	-1.028834	-1.982376
O	-0.304282	2.218047	-1.333953
C	-0.182689	1.042193	-1.044389
H	-3.965912	-1.587031	-0.078102
C	-5.985458	0.928586	0.614314
H	-6.210635	-0.064315	1.001277
H	-7.984556	1.661361	0.863012

H	2.214703	2.021083	-1.549562
C	5.540573	3.784475	0.328440
H	6.092221	3.709919	1.275424
H	6.239102	3.891723	-0.507003
H	4.874358	4.656122	0.378851
C	3.899902	2.265921	1.013278
O	4.801017	2.599454	0.080501
O	3.666486	2.982510	1.965706
C	4.172064	-0.197673	0.565163
O	5.318860	-0.262950	0.142309
C	3.298331	0.959927	0.743329
N	2.156648	0.543147	1.501051
C	2.218444	-0.857438	1.682790
C	1.346930	-1.742220	2.335292
C	3.391107	-1.341055	1.093546
C	1.682355	-3.097774	2.339595
H	0.448065	-1.385690	2.828805
C	3.718002	-2.693366	1.102797
C	2.845266	-3.582999	1.724221
H	1.015808	-3.797327	2.849053
H	4.644496	-3.025882	0.631086
H	3.067955	-4.650226	1.749754
C	0.999464	1.261066	1.746954
O	0.048241	0.737736	2.312172
C	0.930709	2.709643	1.338210
H	1.362157	3.322030	2.139391
H	1.477867	2.929031	0.415865
H	-0.128676	2.957026	1.208507

PCMopt/19_TS_SRb

Frequencies, energies and thermodynamic properties:

Lowest Vibrational Mode (1/cm) = -187.8946

2nd Lowest Vibrational Mode (1/cm) = 17.4668

E(RM062X) (a.u.) = -2588.52262058

Thermal correction to Enthalpy (a.u.) = 0.663112

Thermal correction to Gibbs Free Energy (a.u.) = 0.547920

Total Entropy (cal/Kmol) = 242.441

Esp(RM062X+D3) (a.u.) = -2591.12879105

Esp(D3) (a.u.) = -0.0101053823

Esp(RM062X) (a.u.) = -2591.1186856677

Optimised cartesian coordinates (Angstrom):

C	7.329631	-2.141770	0.657149
C	7.031492	-3.372614	0.055072
C	5.755165	-3.640411	-0.430032
C	4.785060	-2.647101	-0.299829
C	5.083540	-1.426715	0.308021
H	7.809682	-4.130336	-0.037449
H	5.518422	-4.595028	-0.900499
C	2.874029	-1.084771	-0.236992
C	1.655309	1.002869	0.150005
C	2.581680	1.123200	1.372931
C	3.997471	0.733621	0.986721
H	4.633109	0.648530	1.877501
S	3.106377	-2.715926	-0.802933
N	3.970159	-0.579460	0.341404
N	1.745317	-0.373144	-0.384273
C	2.509871	2.494085	2.084569
C	3.484790	3.543742	1.540909
C	2.727543	2.315339	3.588909
H	1.484142	2.874222	1.937647
H	4.523164	3.283695	1.798572
H	3.420406	3.667854	0.451293
H	3.270133	4.516939	2.003839
H	1.979825	1.640875	4.030173
H	3.724571	1.895202	3.793947
H	2.662663	3.283634	4.104319
H	2.213461	0.360488	2.080307
H	0.628799	1.095165	0.528305
C	1.878527	2.046167	-0.924304
C	1.095667	3.206160	-0.915827
C	2.874099	1.902180	-1.895834
C	1.325728	4.222089	-1.841483
H	0.299277	3.317576	-0.175379
C	3.105454	2.919244	-2.822524

H	3.474339	0.990277	-1.946317
C	2.337448	4.083251	-2.792610
H	0.709720	5.121944	-1.822583
H	3.886835	2.797471	-3.573657
H	2.518935	4.876770	-3.518602
C	-0.509832	-0.127536	-1.330587
H	-0.353531	0.939981	-1.477941
C	-1.763425	-0.686245	-1.404572
C	-2.815665	-0.040843	-2.257494
F	-2.528647	-0.209706	-3.555325
F	-4.029477	-0.562930	-2.069547
F	-2.912429	1.278586	-2.056751
O	0.618258	-2.202520	-1.136861
C	0.579592	-0.998195	-0.969793
H	4.456086	1.459211	0.298234
C	6.362730	-1.150807	0.793308
H	6.603752	-0.193571	1.254054
H	8.338250	-1.952574	1.024926
H	-1.831678	-1.776804	-1.363619
C	-0.932554	0.738868	3.350056
H	-0.095704	1.200573	2.803810
H	-1.317697	1.441557	4.095126
H	-0.572535	-0.176833	3.836852
C	-1.753168	-0.424734	1.483754
O	-2.007064	0.457858	2.468731
O	-0.688030	-1.000081	1.402418
C	-3.432003	-1.947648	0.443904
O	-2.826044	-3.003817	0.326236
C	-2.866797	-0.589385	0.546918
N	-3.976832	0.324037	0.656204
C	-5.181632	-0.399673	0.624142
C	-6.511635	0.039143	0.668776
C	-4.896076	-1.766771	0.496814
C	-7.512253	-0.930992	0.601066
H	-6.751996	1.092807	0.762841
C	-5.903305	-2.725666	0.424579
C	-7.226655	-2.299548	0.482162

H -8.553946 -0.607840 0.645999
H -5.640731 -3.780331 0.323982
H -8.042277 -3.021700 0.433444
C -3.908879 1.705955 0.661700
O -4.911006 2.382565 0.792626
C -2.559849 2.370422 0.500068
H -2.276073 2.811512 1.464846
H -1.765919 1.698288 0.160081
H -2.686550 3.180195 -0.228958

PCMopt/19_TS_SRc

Frequencies, energies and thermodynamic properties:

Lowest Vibrational Mode (1/cm) = -166.3487
2nd Lowest Vibrational Mode (1/cm) = 18.3491
E(RM062X) (a.u.) = -2588.52002254
Thermal correction to Enthalpy (a.u.) = 0.662951
Thermal correction to Gibbs Free Energy (a.u.) = 0.547573
Total Entropy (cal/Kmol) = 242.833
Esp(RM062X+D3) (a.u.) = -2591.12610911
Esp(D3) (a.u.) = -0.0101724039
Esp(RM062X) (a.u.) = -2591.1159367061

Optimised cartesian coordinates (Angstrom):

C 7.433650 -1.664924 0.693010
C 7.259596 -2.920814 0.094144
C 6.019794 -3.309317 -0.404036
C 4.958903 -2.411676 -0.289508
C 5.134136 -1.166024 0.315536
H 8.106533 -3.602429 0.014297
H 5.879387 -4.283666 -0.872321
C 2.909040 -1.034646 -0.256484
C 1.490892 0.933262 0.105289
C 2.392317 1.148726 1.334571
C 3.842928 0.885552 0.970915
H 4.469089 0.861886 1.872004
S 3.300200 -2.639172 -0.812950
N 3.946037 -0.427007 0.332737
N 1.720869 -0.434372 -0.418560

C	2.194187	2.515535	2.030644
C	3.049164	3.653103	1.460560
C	2.460942	2.384435	3.532496
H	1.132399	2.786259	1.895145
H	4.112217	3.503786	1.705984
H	2.957591	3.759645	0.371384
H	2.742588	4.603647	1.919001
H	1.805359	1.638213	4.002744
H	3.503175	2.082794	3.720043
H	2.299502	3.347628	4.036183
H	2.088878	0.363423	2.047941
H	0.450200	0.941497	0.461484
C	1.636422	1.984254	-0.974945
C	0.710778	3.033499	-1.017982
C	2.691435	1.960772	-1.892745
C	0.867420	4.070101	-1.937124
H	-0.145384	3.018298	-0.337528
C	2.846159	2.998241	-2.812547
H	3.400512	1.129047	-1.906029
C	1.941404	4.060107	-2.828573
H	0.143122	4.885484	-1.962441
H	3.675054	2.973437	-3.521119
H	2.064185	4.871333	-3.547459
C	-0.524581	-0.376152	-1.390042
H	-0.451108	0.701320	-1.519876
C	-1.744739	-0.981372	-1.437669
C	-2.829702	-0.332096	-2.248049
F	-2.599429	-0.527079	-3.556013
F	-4.043811	-0.829268	-2.007383
F	-2.896233	0.990614	-2.067475
O	0.774108	-2.353126	-1.207936
C	0.629376	-1.161539	-1.022398
H	4.245663	1.646935	0.286508
C	6.376257	-0.768878	0.813136
H	6.521441	0.208984	1.270929
H	8.415684	-1.379840	1.070820
H	-1.825469	-2.068847	-1.346198

C	-0.693381	0.297935	3.390403
H	-0.013395	0.948164	2.819411
H	-1.054242	0.837280	4.272020
H	-0.154422	-0.609289	3.693661
C	-1.616768	-0.766539	1.513858
O	-1.829081	-0.010779	2.607783
O	-0.567662	-1.348749	1.333370
C	-3.558125	-2.035690	0.526100
O	-3.151096	-3.191611	0.418064
C	-2.786641	-0.812617	0.634487
N	-3.698496	0.293503	0.723790
C	-5.017930	-0.191844	0.675421
C	-6.259794	0.456255	0.701294
C	-4.971921	-1.594259	0.562719
C	-7.412058	-0.329393	0.633835
H	-6.365139	1.532930	0.785564
C	-6.126411	-2.366172	0.488203
C	-7.361746	-1.725263	0.529059
H	-8.381745	0.170020	0.668107
H	-6.042194	-3.450849	0.398605
H	-8.286856	-2.300223	0.478150
C	-3.200653	1.580516	0.643039
O	-1.999033	1.766369	0.548110
C	-4.175429	2.726402	0.669247
H	-4.835220	2.685125	-0.208442
H	-4.793794	2.702428	1.575716
H	-3.597825	3.655344	0.644664

PCMopt/19_TS_SRd

Frequencies, energies and thermodynamic properties:

Lowest Vibrational Mode (1/cm) =	-266.6194
2nd Lowest Vibrational Mode (1/cm) =	11.6579
E(RM062X) (a.u.) =	-2588.52103617
Thermal correction to Enthalpy (a.u.) =	0.663011
Thermal correction to Gibbs Free Energy (a.u.) =	0.547133
Total Entropy (cal/Kmol) =	243.886
Esp(RM062X+D3) (a.u.) =	-2591.12571373

Esp(D3) (a.u.) = -0.0105930572

Esp(RM062X) (a.u.) = -2591.1151206728

Optimised cartesian coordinates (Angstrom):

C	6.477913	-2.782170	-0.101138
C	5.888807	-3.956932	-0.590957
C	4.529134	-4.006093	-0.880394
C	3.771277	-2.855134	-0.663844
C	4.359818	-1.690889	-0.168813
H	6.503726	-4.842726	-0.750877
H	4.066226	-4.914726	-1.265989
C	2.179873	-0.984833	-0.404714
C	1.373826	1.272264	0.139870
C	2.551003	1.280722	1.131505
C	3.778094	0.658752	0.492995
H	4.571248	0.519682	1.237817
S	2.057813	-2.632180	-0.958387
N	3.423107	-0.661465	-0.025347
N	1.164063	-0.103967	-0.368854
C	2.840449	2.664508	1.756488
C	3.775410	3.555383	0.930205
C	3.419994	2.495716	3.163209
H	1.866726	3.174460	1.854159
H	4.802509	3.158396	0.942758
H	3.461513	3.664330	-0.116231
H	3.812060	4.558846	1.376924
H	2.743742	1.932050	3.820156
H	4.382583	1.961888	3.131586
H	3.602253	3.477362	3.622185
H	2.240227	0.610151	1.949751
H	0.481815	1.509628	0.733771
C	1.488250	2.267016	-0.995545
C	0.863634	3.512566	-0.871235
C	2.243662	1.992085	-2.139609
C	1.022513	4.484062	-1.857474
H	0.249656	3.723992	0.008624
C	2.403250	2.964139	-3.127660
H	2.710205	1.012925	-2.275460

C	1.800389	4.213931	-2.984494
H	0.533289	5.452778	-1.748194
H	2.998175	2.740901	-4.014159
H	1.925619	4.972930	-3.757820
C	-1.166398	0.483895	-0.910900
H	-1.007002	1.519195	-0.626425
C	-2.432044	0.038831	-1.237718
C	-3.445481	1.030927	-1.721736
F	-3.218227	1.333271	-3.010859
F	-4.696296	0.564132	-1.664269
F	-3.403460	2.181539	-1.047775
O	-0.226850	-1.644349	-1.308134
C	-0.122558	-0.495560	-0.897676
H	4.176720	1.269748	-0.331121
C	5.725639	-1.632536	0.116018
H	6.194416	-0.722098	0.487006
H	7.546822	-2.764189	0.112228
H	-2.528725	-0.944049	-1.703045
C	-6.570253	-2.126890	-0.606533
H	-6.475900	-3.220599	-0.603657
H	-7.533413	-1.827406	-0.183175
H	-6.477615	-1.764419	-1.639386
C	-4.312523	-1.718449	-0.175702
O	-5.573349	-1.532296	0.212644
O	-4.027891	-2.461621	-1.091855
C	-3.800728	0.394341	1.222585
O	-4.850295	1.015445	1.125567
C	-3.374674	-0.847412	0.563199
N	-2.142130	-1.273290	1.167553
C	-1.648322	-0.198475	1.933074
C	-0.455751	-0.062096	2.665957
C	-2.611013	0.819228	1.980391
C	-0.264664	1.123334	3.373521
H	0.294615	-0.845920	2.673653
C	-2.399847	2.006858	2.677932
C	-1.205477	2.166301	3.371746
H	0.655564	1.241728	3.948855

H -3.173870 2.776520 2.672840
H -1.002610 3.082414 3.927332
C -1.493153 -2.507596 1.153264
O -0.339698 -2.588281 1.525957
C -2.252707 -3.732678 0.726539
H -3.274851 -3.746147 1.123672
H -2.313179 -3.770814 -0.366994
H -1.690379 -4.595249 1.098506

PCMopt/19_TS_SRe
Frequencies, energies and thermodynamic properties:
Lowest Vibrational Mode (1/cm) = -247.5010
2nd Lowest Vibrational Mode (1/cm) = 17.2205
E(RM062X) (a.u.) = -2588.52333833
Thermal correction to Enthalpy (a.u.) = 0.663536
Thermal correction to Gibbs Free Energy (a.u.) = 0.547826
Total Entropy (cal/Kmol) = 243.532
Esp(RM062X+D3) (a.u.) = -2591.12576288
Esp(D3) (a.u.) = -0.0100272652
Esp(RM062X) (a.u.) = -2591.1157356148
Optimised cartesian coordinates (Angstrom):
C -6.641142 2.873456 0.195362
C -6.137839 3.930576 -0.575439
C -4.835748 3.899066 -1.066637
C -4.050535 2.786197 -0.769478
C -4.552813 1.739372 0.005462
H -6.773598 4.788083 -0.796472
H -4.439492 4.717561 -1.668086
C -2.435778 0.944941 -0.433136
C -1.575632 -1.219534 0.317939
C -2.517329 -0.991785 1.512791
C -3.848813 -0.442902 1.030658
H -4.463121 -0.114763 1.879194
S -2.385449 2.491371 -1.241191
N -3.603038 0.728802 0.189910
N -1.449998 0.043004 -0.442671
C -2.673221 -2.227070 2.429030

C	-3.814192	-3.169904	2.034169
C	-2.841764	-1.781772	3.883059
H	-1.727379	-2.789516	2.362772
H	-4.793277	-2.702023	2.221935
H	-3.773238	-3.473360	0.978695
H	-3.767351	-4.080262	2.648122
H	-1.978280	-1.193402	4.223701
H	-3.745367	-1.163322	4.002468
H	-2.946491	-2.653586	4.544083
H	-2.028548	-0.198890	2.102734
H	-0.576865	-1.391048	0.742306
C	-1.962455	-2.372253	-0.584734
C	-1.362545	-3.621378	-0.393486
C	-2.940866	-2.234079	-1.575418
C	-1.759707	-4.723390	-1.148902
H	-0.574256	-3.732089	0.354293
C	-3.339320	-3.335449	-2.333112
H	-3.397638	-1.260861	-1.771745
C	-2.756327	-4.584082	-2.115402
H	-1.284824	-5.691685	-0.985812
H	-4.106136	-3.214959	-3.099437
H	-3.068890	-5.444734	-2.708187
C	0.778327	-0.679316	-1.176526
H	0.526967	-1.720641	-0.995227
C	2.097137	-0.298979	-1.341991
C	3.062987	-1.272852	-1.949143
F	2.637149	-1.691709	-3.147097
F	4.261454	-0.713426	-2.153777
F	3.250226	-2.375756	-1.212024
O	-0.028672	1.524774	-1.442780
C	-0.181675	0.372982	-1.059209
H	-4.421189	-1.183794	0.452512
C	-5.858500	1.762958	0.497449
H	-6.259234	0.940662	1.089058
H	-7.665798	2.916481	0.565092
H	2.296090	0.728183	-1.660990
C	2.095151	-3.134366	2.111823

H	2.266851	-2.897895	3.169714
H	1.119538	-3.613149	1.987131
H	2.891982	-3.804981	1.763457
C	3.167391	-1.194923	1.392449
O	2.066243	-1.948529	1.329228
O	4.122984	-1.500898	2.068746
C	1.907216	0.907281	1.001735
O	0.783701	0.546692	1.337722
C	3.031166	0.049774	0.603083
N	4.178307	0.905290	0.436177
C	3.747047	2.249059	0.501716
C	4.439005	3.451978	0.281930
C	2.384301	2.284990	0.829184
C	3.726603	4.642148	0.415413
H	5.491897	3.458192	0.025858
C	1.681003	3.481690	0.961001
C	2.363034	4.672909	0.750750
H	4.255011	5.583310	0.252780
H	0.618674	3.457096	1.209910
H	1.847546	5.629699	0.840468
C	5.501342	0.564523	0.162641
O	6.335775	1.439057	0.032274
C	5.931129	-0.878685	0.059092
H	6.398287	-1.152622	1.013784
H	5.138658	-1.595201	-0.148045
H	6.690230	-0.915920	-0.731085

PCMopt/19_TS_SRf

Frequencies, energies and thermodynamic properties:

Lowest Vibrational Mode (1/cm) = -269.6023

2nd Lowest Vibrational Mode (1/cm) = 16.5703

E(RM062X) (a.u.) = -2588.52557942

Thermal correction to Enthalpy (a.u.) = 0.663568

Thermal correction to Gibbs Free Energy (a.u.) = 0.548583

Total Entropy (cal/Kmol) = 242.005

Esp(RM062X+D3) (a.u.) = -2591.12625054

Esp(D3) (a.u.) = -0.0099017970

Esp(RM062X) (a.u.) = -2591.1163487430

Optimised cartesian coordinates (Angstrom):

C	7.015790	-2.326315	0.394980
C	6.712099	-3.389684	-0.465803
C	5.454333	-3.496066	-1.053609
C	4.509750	-2.513289	-0.762716
C	4.813514	-1.460308	0.102891
H	7.469708	-4.143249	-0.681447
H	5.213809	-4.321285	-1.724276
C	2.648289	-0.914199	-0.459123
C	1.484352	1.094875	0.300519
C	2.326344	0.900935	1.571364
C	3.753717	0.535206	1.201606
H	4.316910	0.213281	2.087712
S	2.853393	-2.408997	-1.337782
N	3.728920	-0.592693	0.268056
N	1.564876	-0.139771	-0.513628
C	2.237324	2.087889	2.556987
C	3.287227	3.180211	2.332270
C	2.310839	1.579544	3.998189
H	1.239553	2.535226	2.412486
H	4.293208	2.819348	2.598524
H	3.313463	3.540646	1.294401
H	3.069051	4.039716	2.981721
H	1.497132	0.872935	4.214188
H	3.267488	1.066675	4.185890
H	2.237494	2.415785	4.707823
H	1.877281	0.024253	2.066771
H	0.438483	1.153391	0.628409
C	1.825714	2.329044	-0.505439
C	1.046916	3.478635	-0.326537
C	2.915130	2.370821	-1.382059
C	1.380445	4.664545	-0.979556
H	0.168055	3.427903	0.321590
C	3.246920	3.556886	-2.037597
H	3.512337	1.474297	-1.567696
C	2.486885	4.708324	-1.829282

H	0.769625	5.556145	-0.830474
H	4.100916	3.579531	-2.715993
H	2.748496	5.635553	-2.341077
C	-0.641338	0.379049	-1.407928
H	-0.431060	1.439245	-1.294402
C	-1.955316	-0.042402	-1.530482
C	-2.902050	0.837527	-2.289467
F	-2.529314	0.942133	-3.570827
F	-4.149330	0.352592	-2.306545
F	-2.955207	2.085585	-1.807134
O	0.344759	-1.773898	-1.556992
C	0.371069	-0.604534	-1.200778
H	4.290716	1.373943	0.733737
C	6.072792	-1.346645	0.692644
H	6.316327	-0.517359	1.355944
H	8.008683	-2.259860	0.840190
H	-2.137589	-1.101920	-1.736174
C	-3.628603	2.849747	2.488089
H	-3.523737	3.651453	1.744728
H	-4.555365	2.976289	3.055253
H	-2.762655	2.871798	3.162173
C	-2.699927	1.239207	1.059951
O	-3.726403	1.585143	1.843646
O	-1.695495	1.908006	0.970861
C	-1.873516	-1.057060	0.882725
O	-0.703229	-0.826491	1.163000
C	-2.869296	-0.081164	0.412432
N	-4.141620	-0.757911	0.391264
C	-3.917845	-2.136821	0.596127
C	-4.796107	-3.230669	0.536913
C	-2.561102	-2.353135	0.880682
C	-4.268102	-4.498226	0.777297
H	-5.849408	-3.096559	0.319893
C	-2.043486	-3.625298	1.121125
C	-2.910518	-4.709317	1.066816
H	-4.942101	-5.355893	0.738578
H	-0.979821	-3.743232	1.335176

H -2.544065 -5.720752 1.244882
C -5.408418 -0.239427 0.135341
O -6.378594 -0.970870 0.153842
C -5.603732 1.232517 -0.141585
H -6.052897 1.681289 0.754183
H -4.705095 1.790822 -0.402450
H -6.328665 1.296819 -0.961817

PCMopt/19_TS_SRg
Frequencies, energies and thermodynamic properties:
Lowest Vibrational Mode (1/cm) = -252.1977
2nd Lowest Vibrational Mode (1/cm) = 19.4310
E(RM062X) (a.u.) = -2588.52348235
Thermal correction to Enthalpy (a.u.) = 0.663389
Thermal correction to Gibbs Free Energy (a.u.) = 0.547941
Total Entropy (cal/Kmol) = 242.981
Esp(RM062X+D3) (a.u.) = -2591.12562243
Esp(D3) (a.u.) = -0.0103192487
Esp(RM062X) (a.u.) = -2591.1153031813
Optimised cartesian coordinates (Angstrom):
C -7.207220 -1.591688 -1.066478
C -7.142915 -2.753523 -0.283907
C -5.987004 -3.078583 0.419233
C -4.897764 -2.213123 0.322798
C -4.962623 -1.062460 -0.464974
H -8.010112 -3.411132 -0.222254
H -5.932463 -3.980225 1.029480
C -2.826030 -0.869706 0.364651
C -1.355088 1.031949 -0.064835
C -2.055210 1.069045 -1.429768
C -3.547227 0.854609 -1.253799
H -4.037901 0.679707 -2.220266
S -3.323523 -2.379531 1.078859
N -3.763738 -0.342062 -0.433659
N -1.646005 -0.269824 0.581726
C -1.699765 2.324791 -2.257119
C -2.643299 3.511306 -2.043105

C	-1.624338	1.964682	-3.741944
H	-0.687045	2.623293	-1.937268
H	-3.639086	3.303641	-2.465579
H	-2.765916	3.770849	-0.981956
H	-2.248286	4.395795	-2.562490
H	-0.864148	1.190680	-3.918716
H	-2.592828	1.585354	-4.105001
H	-1.365773	2.847878	-4.343195
H	-1.646590	0.200009	-1.972239
H	-0.281514	1.025036	-0.286270
C	-1.663287	2.196393	0.852416
C	-0.762912	3.267899	0.895958
C	-2.834237	2.256481	1.616800
C	-1.049701	4.398401	1.660550
H	0.169461	3.198100	0.330129
C	-3.118852	3.387030	2.382862
H	-3.536174	1.418815	1.629852
C	-2.231948	4.464084	2.398709
H	-0.342870	5.229121	1.685054
H	-4.036398	3.423954	2.971656
H	-2.456621	5.348016	2.997278
C	0.572207	-0.182906	1.592952
H	0.532761	0.903411	1.578451
C	1.802343	-0.797846	1.625405
C	2.920687	-0.067802	2.320415
F	2.751256	-0.142179	3.649265
F	4.131293	-0.573548	2.071631
F	2.963595	1.229008	2.009789
O	-0.827850	-2.107180	1.660263
C	-0.612166	-0.953440	1.332512
H	-4.036461	1.709510	-0.763276
C	-6.120522	-0.729457	-1.169762
H	-6.178998	0.175779	-1.772939
H	-8.125318	-1.354679	-1.604351
H	1.874382	-1.889575	1.672942
C	-0.242708	-2.783257	-1.364705
H	-0.032121	-3.007530	-2.419615

H	-0.575965	-3.684097	-0.840821
H	-1.026910	-2.014488	-1.323726
C	1.461664	-1.186025	-1.113239
O	0.930320	-2.346323	-0.702382
O	0.911754	-0.487625	-1.939784
C	3.672131	-1.993849	-0.201547
O	3.461573	-3.128096	0.205598
C	2.711678	-0.900365	-0.406589
N	3.460447	0.253578	-0.848592
C	4.828052	-0.084782	-0.933910
C	5.964313	0.667145	-1.267330
C	4.988209	-1.428424	-0.559826
C	7.206553	0.031008	-1.242995
H	5.925671	1.713931	-1.546925
C	6.230930	-2.052555	-0.528627
C	7.355394	-1.314609	-0.883994
H	8.088742	0.612877	-1.515077
H	6.296554	-3.099038	-0.225194
H	8.345705	-1.770617	-0.877475
C	2.879068	1.507292	-0.943166
O	1.765383	1.713352	-0.501955
C	3.652831	2.608724	-1.623152
H	4.411572	3.017798	-0.941532
H	4.145228	2.263581	-2.539647
H	2.938648	3.403703	-1.860134

PCMopt/19_TS_SRh

Frequencies, energies and thermodynamic properties:

Lowest Vibrational Mode (1/cm) =	-180.2417
2nd Lowest Vibrational Mode (1/cm) =	18.2828
E(RM062X) (a.u.) =	-2588.51789150
Thermal correction to Enthalpy (a.u.) =	0.663338
Thermal correction to Gibbs Free Energy (a.u.) =	0.547881
Total Entropy (cal/Kmol) =	243.001
Esp(RM062X+D3) (a.u.) =	-2591.12561372
Esp(D3) (a.u.) =	-0.0107053218
Esp(RM062X) (a.u.) =	-2591.1149083982

Optimised cartesian coordinates (Angstrom):

C	6.509478	-2.767904	-0.366619
C	5.911262	-3.928990	-0.878150
C	4.541616	-3.982359	-1.113620
C	3.784429	-2.847917	-0.821086
C	4.381553	-1.697660	-0.304090
H	6.527269	-4.800999	-1.098093
H	4.071732	-4.880133	-1.515630
C	2.190597	-1.003519	-0.424916
C	1.392690	1.227895	0.225879
C	2.592000	1.196480	1.188171
C	3.807159	0.612538	0.493188
H	4.615337	0.438198	1.214332
S	2.058004	-2.632782	-1.028729
N	3.442839	-0.683875	-0.080315
N	1.168552	-0.130877	-0.326236
C	2.879126	2.557319	1.864475
C	3.839110	3.462112	1.084514
C	3.414691	2.337429	3.280727
H	1.909650	3.075765	1.953284
H	4.861838	3.054097	1.101730
H	3.542696	3.605340	0.036538
H	3.877391	4.451441	1.561353
H	2.691026	1.797221	3.906849
H	4.350971	1.757749	3.263420
H	3.629434	3.300900	3.763568
H	2.306218	0.485770	1.979713
H	0.513041	1.444266	0.848208
C	1.497942	2.265769	-0.871833
C	0.897217	3.515366	-0.684104
C	2.223795	2.024902	-2.042273
C	1.052134	4.523125	-1.633841
H	0.302339	3.700258	0.214599
C	2.379125	3.033619	-2.993735
H	2.669682	1.044637	-2.229570
C	1.801564	4.286288	-2.787238
H	0.581609	5.494209	-1.475127

H	2.950838	2.836322	-3.901354
H	1.923651	5.073894	-3.531864
C	-1.136972	0.521358	-0.870445
H	-0.908714	1.551422	-0.616543
C	-2.406279	0.191008	-1.250467
C	-3.318571	1.282085	-1.730111
F	-3.092388	1.517341	-3.034535
F	-4.611806	0.978167	-1.626484
F	-3.113467	2.435346	-1.092845
O	-0.291042	-1.673664	-1.152121
C	-0.135611	-0.514151	-0.806186
H	4.188213	1.261722	-0.309307
C	5.757229	-1.635669	-0.073092
H	6.232570	-0.734967	0.313109
H	7.586124	-2.747123	-0.197194
H	-2.623504	-0.801300	-1.645207
C	-5.414061	-2.723877	-1.709671
H	-6.197627	-2.066905	-2.109788
H	-4.946882	-3.291842	-2.520076
H	-5.862365	-3.409213	-0.976915
C	-4.747173	-1.224088	-0.041506
O	-4.382698	-1.965828	-1.102366
O	-5.907456	-1.051324	0.247310
C	-3.679239	0.684324	1.315291
O	-4.538919	1.565174	1.263997
C	-3.609016	-0.592540	0.647606
N	-2.476673	-1.327115	1.124072
C	-1.700531	-0.452170	1.907327
C	-0.477793	-0.629833	2.577763
C	-2.389274	0.768844	2.034739
C	0.010367	0.429990	3.340404
H	0.098995	-1.548777	2.528951
C	-1.867247	1.833628	2.766829
C	-0.653573	1.664089	3.426699
H	0.944070	0.283660	3.886784
H	-2.430560	2.767071	2.825113
H	-0.225287	2.471863	4.021832

C -2.484552 -2.721161 1.209583
O -3.503423 -3.345341 1.006081
C -1.187342 -3.414088 1.535170
H -0.352409 -2.983881 0.968232
H -0.974142 -3.346183 2.610915
H -1.312157 -4.470169 1.274429

PCMopt/19_TS_SRi

Frequencies, energies and thermodynamic properties:

Lowest Vibrational Mode (1/cm) = -214.4343
2nd Lowest Vibrational Mode (1/cm) = 16.8470
E(RM062X) (a.u.) = -2588.51788505
Thermal correction to Enthalpy (a.u.) = 0.663196
Thermal correction to Gibbs Free Energy (a.u.) = 0.548366
Total Entropy (cal/Kmol) = 241.681
Esp(RM062X+D3) (a.u.) = -2591.12498671
Esp(D3) (a.u.) = -0.0106449861
Esp(RM062X) (a.u.) = -2591.1143417239

Optimised cartesian coordinates (Angstrom):

C 6.660499 -2.555774 -0.440238
C 6.114206 -3.716907 -1.006281
C 4.748864 -3.817005 -1.252847
C 3.942799 -2.729595 -0.916071
C 4.488685 -1.579876 -0.344033
H 6.767400 -4.551701 -1.260646
H 4.319549 -4.715220 -1.697173
C 2.271100 -0.970304 -0.451827
C 1.383699 1.200026 0.273199
C 2.570439 1.176407 1.251805
C 3.815510 0.665847 0.551773
H 4.619516 0.490454 1.277488
S 2.208897 -2.577307 -1.125467
N 3.507749 -0.616539 -0.082215
N 1.216026 -0.144497 -0.329444
C 2.799216 2.519855 1.983210
C 3.735108 3.488598 1.252102
C 3.326047 2.265416 3.396993

H	1.810420	2.999198	2.078635
H	4.772211	3.118610	1.269923
H	3.448425	3.660040	0.205711
H	3.729255	4.460009	1.765900
H	2.616213	1.674926	3.992655
H	4.283568	1.721895	3.369517
H	3.499014	3.216744	3.919113
H	2.301474	0.425859	2.012242
H	0.487269	1.363323	0.887445
C	1.468353	2.280278	-0.785004
C	0.823611	3.501879	-0.562147
C	2.216578	2.105511	-1.953016
C	0.956094	4.547103	-1.474046
H	0.211677	3.635282	0.334206
C	2.349523	3.151679	-2.866694
H	2.697134	1.147540	-2.167857
C	1.727223	4.376269	-2.624812
H	0.451056	5.495773	-1.288011
H	2.938732	3.005715	-3.772847
H	1.831684	5.193184	-3.339969
C	-1.100423	0.438077	-0.885468
H	-0.902843	1.469573	-0.613838
C	-2.369696	0.078798	-1.268365
C	-3.276181	1.167892	-1.769858
F	-3.018140	1.408536	-3.066418
F	-4.573021	0.862826	-1.699437
F	-3.093931	2.320084	-1.122840
O	-0.178080	-1.719867	-1.211363
C	-0.073158	-0.561437	-0.836615
H	4.183034	1.362877	-0.216114
C	5.859264	-1.470481	-0.101535
H	6.293867	-0.568286	0.327065
H	7.734548	-2.497152	-0.263289
H	-2.550372	-0.904379	-1.707078
C	-6.836428	-1.431861	-0.838563
H	-6.913672	-2.526114	-0.886875
H	-7.760593	-1.004724	-0.437667

H	-6.645450	-1.045705	-1.849342
C	-4.563372	-1.408664	-0.310920
O	-5.797150	-1.037186	0.043525
O	-4.353753	-2.135030	-1.257629
C	-3.671568	0.505451	1.229488
O	-4.546595	1.364920	1.169901
C	-3.533347	-0.752571	0.514357
N	-2.405138	-1.468248	1.025773
C	-1.682556	-0.591727	1.855822
C	-0.473788	-0.751736	2.555045
C	-2.410703	0.603434	1.997012
C	-0.038260	0.301748	3.357035
H	0.131983	-1.651067	2.495376
C	-1.942564	1.663327	2.771237
C	-0.741585	1.512596	3.458374
H	0.886444	0.170056	3.922089
H	-2.534869	2.578029	2.837745
H	-0.352865	2.316826	4.084500
C	-2.381557	-2.866317	1.105860
O	-3.386404	-3.511916	0.913798
C	-1.063432	-3.527364	1.414960
H	-0.239046	-3.051337	0.870403
H	-0.861750	-3.492645	2.494606
H	-1.153323	-4.577495	1.117559

PCMopt/19_TS_SRj

Frequencies, energies and thermodynamic properties:

Lowest Vibrational Mode (1/cm) = -230.8483

2nd Lowest Vibrational Mode (1/cm) = 18.2208

E(RM062X) (a.u.) = -2588.51817207

Thermal correction to Enthalpy (a.u.) = 0.663292

Thermal correction to Gibbs Free Energy (a.u.) = 0.547382

Total Entropy (cal/Kmol) = 243.953

Esp(RM062X+D3) (a.u.) = -2591.12206671

Esp(D3) (a.u.) = -0.0099703197

Esp(RM062X) (a.u.) = -2591.1120963903

Optimised cartesian coordinates (Angstrom):

C	-6.595558	2.925425	0.174007
C	-6.086262	3.965796	-0.615320
C	-4.786625	3.914960	-1.111572
C	-4.010056	2.799787	-0.800747
C	-4.518279	1.769712	-0.007319
H	-6.715317	4.825403	-0.847110
H	-4.385955	4.720480	-1.727467
C	-2.411378	0.947952	-0.444305
C	-1.572446	-1.215252	0.330565
C	-2.499284	-0.957588	1.530335
C	-3.828954	-0.400780	1.052177
H	-4.431071	-0.050425	1.900658
S	-2.349690	2.482204	-1.274973
N	-3.577324	0.753270	0.188778
N	-1.436402	0.035363	-0.447896
C	-2.658500	-2.176867	2.467043
C	-3.814454	-3.112417	2.099608
C	-2.805027	-1.707727	3.915930
H	-1.719501	-2.750688	2.398014
H	-4.785984	-2.630502	2.291117
H	-3.788975	-3.431999	1.048417
H	-3.771013	-4.014003	2.726642
H	-1.931187	-1.123791	4.237239
H	-3.700362	-1.077522	4.036266
H	-2.911352	-2.568075	4.591586
H	-1.993985	-0.161548	2.101849
H	-0.572945	-1.393783	0.749686
C	-1.982769	-2.374699	-0.552764
C	-1.395955	-3.628232	-0.349935
C	-2.968999	-2.238373	-1.535937
C	-1.813357	-4.735664	-1.086290
H	-0.602045	-3.737173	0.392090
C	-3.387671	-3.345152	-2.274624
H	-3.416104	-1.262501	-1.741137
C	-2.817468	-4.597617	-2.045193
H	-1.348454	-5.707287	-0.914374
H	-4.160307	-3.226038	-3.035310

H	-3.145886	-5.462463	-2.623133
C	0.781588	-0.722329	-1.177769
H	0.509183	-1.758138	-0.994264
C	2.102496	-0.373057	-1.362723
C	3.051682	-1.390632	-1.944578
F	2.501398	-1.977558	-3.023257
F	4.175674	-0.815788	-2.373244
F	3.376478	-2.385059	-1.117989
O	0.003351	1.490277	-1.463529
C	-0.164125	0.345975	-1.065959
H	-4.415826	-1.144490	0.492391
C	-5.821398	1.812790	0.489977
H	-6.226431	1.003215	1.096082
H	-7.618169	2.983364	0.547351
H	2.325965	0.644249	-1.694098
C	2.165610	-3.188485	2.049550
H	2.370446	-2.986511	3.108916
H	1.191710	-3.675785	1.942300
H	2.957728	-3.839072	1.654080
C	3.190319	-1.205393	1.380081
O	2.099499	-1.980888	1.307778
O	4.147230	-1.500808	2.055079
C	1.888168	0.893385	1.000858
O	0.758635	0.527372	1.326713
C	3.015925	0.052732	0.618702
N	4.151601	0.912330	0.445515
C	3.721560	2.250463	0.498306
C	4.385295	3.461055	0.237465
C	2.355737	2.276567	0.828586
C	3.663550	4.645872	0.363135
H	5.427218	3.513702	-0.059635
C	1.641871	3.467931	0.950160
C	2.306013	4.665540	0.720788
H	4.178789	5.588154	0.169699
H	0.581069	3.433399	1.204688
H	1.780198	5.617166	0.803360
C	5.422730	0.414515	0.153261

O	5.578104	-0.749670	-0.129538
C	6.593640	1.363117	0.240034
H	6.670721	1.961768	-0.678141
H	6.521112	2.038182	1.100731
H	7.494425	0.746245	0.321465

PCMopt/19_TS_SRk

Frequencies, energies and thermodynamic properties:

Lowest Vibrational Mode (1/cm) = -353.7918

2nd Lowest Vibrational Mode (1/cm) = 14.9061

E(RM062X) (a.u.) = -2588.51374822

Thermal correction to Enthalpy (a.u.) = 0.662788

Thermal correction to Gibbs Free Energy (a.u.) = 0.546332

Total Entropy (cal/Kmol) = 245.103

Esp(RM062X+D3) (a.u.) = -2591.11503253

Esp(D3) (a.u.) = -0.0098978069

Esp(RM062X) (a.u.) = -2591.1051347231

Optimised cartesian coordinates (Angstrom):

C	7.035033	-2.294932	0.227435
C	6.684254	-3.380577	-0.585932
C	5.392368	-3.506625	-1.090344
C	4.462152	-2.521395	-0.764501
C	4.813183	-1.445861	0.054247
H	7.431159	-4.135958	-0.830639
H	5.114779	-4.349068	-1.724302
C	2.613294	-0.922275	-0.386492
C	1.480212	1.093700	0.398278
C	2.407335	0.938292	1.614962
C	3.811050	0.575897	1.160974
H	4.435038	0.285636	2.016734
S	2.772118	-2.440313	-1.236720
N	3.735936	-0.578566	0.264150
N	1.525666	-0.154701	-0.397119
C	2.372531	2.147953	2.576246
C	3.394393	3.243184	2.256064
C	2.547595	1.675673	4.020962
H	1.363518	2.584354	2.488941

H	4.419640	2.896324	2.460131
H	3.346000	3.580705	1.211327
H	3.213098	4.115025	2.900410
H	1.757611	0.967570	4.308139
H	3.520007	1.176186	4.156747
H	2.513069	2.528598	4.713475
H	2.003100	0.069765	2.160823
H	0.456206	1.143118	0.791612
C	1.746956	2.319648	-0.447064
C	0.955933	3.456179	-0.239617
C	2.778518	2.366991	-1.390369
C	1.222787	4.636575	-0.931815
H	0.117167	3.398113	0.459867
C	3.043785	3.547794	-2.084932
H	3.380584	1.478499	-1.598336
C	2.273963	4.687356	-1.848932
H	0.602900	5.517955	-0.760438
H	3.853344	3.575596	-2.815630
H	2.483686	5.610226	-2.391550
C	-0.700231	0.341391	-1.247590
H	-0.473055	1.401376	-1.174375
C	-2.038042	-0.064025	-1.320198
C	-2.943469	0.802368	-2.163079
F	-2.752841	0.494262	-3.456541
F	-4.244687	0.644261	-1.933631
F	-2.664740	2.106487	-2.042457
O	0.253656	-1.828583	-1.314871
C	0.298783	-0.638402	-1.025209
H	4.306329	1.406182	0.635430
C	6.106542	-1.312192	0.559927
H	6.387139	-0.465340	1.185309
H	8.053640	-2.212677	0.607178
H	-2.231067	-1.124407	-1.517137
C	-3.646845	3.503234	0.469520
H	-2.791406	3.843414	-0.129743
H	-4.576223	3.923117	0.073580
H	-3.503041	3.815147	1.512274

C	-2.749338	1.374629	0.825177
O	-3.780574	2.093313	0.383175
O	-1.740192	1.885198	1.264023
C	-1.864997	-1.022742	1.085104
O	-0.717384	-0.795468	1.435733
C	-2.865850	-0.073221	0.569830
N	-4.114306	-0.775637	0.436570
C	-3.810234	-2.162837	0.457654
C	-4.600591	-3.271018	0.132056
C	-2.483202	-2.348447	0.872960
C	-4.030177	-4.537464	0.258423
H	-5.620536	-3.174982	-0.230620
C	-1.918600	-3.616768	0.989592
C	-2.705988	-4.721320	0.684261
H	-4.634597	-5.408957	0.002086
H	-0.877063	-3.717070	1.299798
H	-2.296589	-5.729155	0.757842
C	-5.284571	-0.316914	1.087169
O	-5.236544	0.549555	1.923563
C	-6.580530	-0.964862	0.673114
H	-6.601055	-1.175155	-0.402339
H	-6.724062	-1.904947	1.224846
H	-7.393574	-0.282968	0.944267

PCMopt/19_TS_SRl

Frequencies, energies and thermodynamic properties:

Lowest Vibrational Mode (1/cm) =	-379.5549
2nd Lowest Vibrational Mode (1/cm) =	15.0679
E(RM062X) (a.u.) =	-2588.51125158
Thermal correction to Enthalpy (a.u.) =	0.662743
Thermal correction to Gibbs Free Energy (a.u.) =	0.547246
Total Entropy (cal/Kmol) =	243.084
Esp(RM062X+D3) (a.u.) =	-2591.11369490
Esp(D3) (a.u.) =	-0.0104725313
Esp(RM062X) (a.u.) =	-2591.1032223687

Optimised cartesian coordinates (Angstrom):

C	-7.376575	-0.987858	-0.475560

C	-7.295446	-2.257634	0.112228
C	-6.077536	-2.759475	0.562513
C	-4.944573	-1.961211	0.412615
C	-5.028104	-0.700823	-0.181895
H	-8.196999	-2.860574	0.221624
H	-6.009258	-3.746115	1.021423
C	-2.778539	-0.775439	0.307382
C	-1.218826	1.053091	-0.097426
C	-2.122820	1.346645	-1.305322
C	-3.580582	1.222832	-0.899041
H	-4.233772	1.247182	-1.781090
S	-3.293549	-2.344906	0.870280
N	-3.779583	-0.071365	-0.242483
N	-1.540873	-0.288388	0.429185
C	-1.790438	2.678788	-2.015027
C	-2.531889	3.896375	-1.454088
C	-2.060498	2.555852	-3.516329
H	-0.705859	2.840070	-1.880434
H	-3.604926	3.846227	-1.697070
H	-2.429223	3.997982	-0.365086
H	-2.137062	4.811790	-1.916639
H	-1.462377	1.753440	-3.971243
H	-3.122877	2.337261	-3.707604
H	-1.816883	3.496563	-4.029705
H	-1.915105	0.528694	-2.016855
H	-0.200523	0.968255	-0.492141
C	-1.236142	2.106214	0.990526
C	-0.233162	3.082474	1.002261
C	-2.251394	2.155528	1.951660
C	-0.268459	4.115850	1.937711
H	0.580412	3.026082	0.273594
C	-2.285470	3.189810	2.887174
H	-3.023326	1.382521	1.985465
C	-1.299465	4.176805	2.876098
H	0.516311	4.873598	1.936648
H	-3.084142	3.221073	3.629469
H	-1.327518	4.985407	3.607811

C	0.764389	-0.494779	1.208841
H	0.850975	0.586970	1.283908
C	1.927321	-1.274802	1.226786
C	3.016654	-0.775759	2.141558
F	2.607407	-0.865395	3.415769
F	4.149078	-1.479370	2.073356
F	3.321088	0.507377	1.928017
O	-0.732745	-2.323646	1.087454
C	-0.471682	-1.133140	0.937840
H	-3.891530	2.024188	-0.211968
C	-6.246502	-0.190520	-0.631781
H	-6.318424	0.799248	-1.081255
H	-8.341732	-0.612341	-0.816092
H	1.811796	-2.362095	1.291611
C	0.456575	-3.975968	-1.492797
H	0.132726	-3.759119	-2.519169
H	0.716612	-5.033961	-1.394097
H	-0.343089	-3.703245	-0.790737
C	1.534846	-1.922931	-1.233048
O	1.640731	-3.249293	-1.179165
O	0.503978	-1.358023	-1.543739
C	4.066617	-1.836784	-0.579405
O	4.361631	-3.009438	-0.452447
C	2.713400	-1.228945	-0.712942
N	2.896249	0.170775	-0.976977
C	4.243994	0.494981	-0.733116
C	4.874917	1.742982	-0.717147
C	4.983291	-0.677206	-0.533160
C	6.251407	1.763869	-0.496570
H	4.310725	2.661906	-0.857814
C	6.356379	-0.644139	-0.302569
C	6.995230	0.591568	-0.286176
H	6.762106	2.728285	-0.476798
H	6.900202	-1.576654	-0.140671
H	8.068446	0.656500	-0.105319
C	2.098498	1.083864	-1.693758
O	2.049802	2.239291	-1.324268

C 1.473237 0.679536 -3.006167

H 1.830412 -0.285906 -3.374156

H 0.381508 0.637568 -2.911357

H 1.731361 1.478008 -3.714993

PCMopt/19_TS_SSa

Frequencies, energies and thermodynamic properties:

Lowest Vibrational Mode (1/cm) = -253.6756

2nd Lowest Vibrational Mode (1/cm) = 16.0096

E(RM062X) (a.u.) = -2588.53204846

Thermal correction to Enthalpy (a.u.) = 0.663497

Thermal correction to Gibbs Free Energy (a.u.) = 0.548149

Total Entropy (cal/Kmol) = 242.772

Esp(RM062X+D3) (a.u.) = -2591.13219402

Esp(D3) (a.u.) = -0.0099864884

Esp(RM062X) (a.u.) = -2591.1222075316

Optimised cartesian coordinates (Angstrom):

C 7.370495 -1.539124 0.619747

C 7.220747 -2.704845 -0.143988

C 5.996262 -3.027208 -0.722408

C 4.927352 -2.155643 -0.520358

C 5.077869 -0.999654 0.248602

H 8.073713 -3.367590 -0.290793

H 5.874407 -3.931752 -1.318699

C 2.869143 -0.796396 -0.366530

C 1.430907 1.092983 0.221601

C 2.277215 1.117284 1.503625

C 3.745571 0.925681 1.164543

H 4.340227 0.762229 2.073074

S 3.281513 -2.321064 -1.107924

N 3.886137 -0.271459 0.331817

N 1.691860 -0.179019 -0.492601

C 2.011369 2.353686 2.391600

C 2.906491 3.558209 2.085738

C 2.129429 1.973488 3.868831

H 0.964575 2.647212 2.206368

H 3.948408 3.359329 2.382340

H	2.897659	3.837393	1.022791
H	2.564881	4.427523	2.665133
H	1.413129	1.183599	4.135569
H	3.142436	1.608725	4.101715
H	1.934512	2.845010	4.509696
H	1.948818	0.227907	2.066373
H	0.383164	1.031666	0.544488
C	1.606251	2.295779	-0.678822
C	0.669843	3.333068	-0.590681
C	2.689723	2.424373	-1.554299
C	0.841044	4.501204	-1.332416
H	-0.202533	3.204472	0.055650
C	2.858749	3.592236	-2.298783
H	3.409354	1.609507	-1.669477
C	1.941110	4.636852	-2.180643
H	0.108386	5.305815	-1.254055
H	3.709218	3.684298	-2.975743
H	2.075576	5.550276	-2.761788
C	-0.550944	-0.032981	-1.439996
H	-0.506851	1.051216	-1.386823
C	-1.772597	-0.669675	-1.539741
C	-2.872101	0.022077	-2.295628
F	-2.640470	-0.054705	-3.612704
F	-4.077670	-0.524921	-2.096513
F	-2.969079	1.319168	-1.995117
O	0.720392	-2.033570	-1.412303
C	0.590702	-0.846310	-1.152358
H	4.164458	1.787857	0.624323
C	6.303805	-0.669676	0.827908
H	6.429680	0.239781	1.414453
H	8.340182	-1.303523	1.058716
H	-1.767113	-1.747474	-1.707464
C	-0.894122	-3.390777	2.322498
H	-0.510434	-2.834541	3.187669
H	-1.348273	-4.331637	2.646361
H	-0.065278	-3.584135	1.627795
C	-1.558907	-1.467590	1.163738

O	-1.922441	-2.655264	1.671260
O	-0.438141	-1.022807	1.302114
C	-2.699824	0.680343	0.724571
O	-1.765105	1.465142	0.822467
C	-2.627780	-0.767726	0.437835
N	-3.973290	-1.281351	0.520679
C	-4.855140	-0.219034	0.792776
C	-6.251303	-0.204850	0.922650
C	-4.131123	0.978006	0.891566
C	-6.865113	1.024835	1.157955
H	-6.830908	-1.119141	0.853688
C	-4.755291	2.201373	1.129532
C	-6.138869	2.221564	1.263647
H	-7.950647	1.050839	1.269407
H	-4.155162	3.110017	1.203009
H	-6.662575	3.159315	1.451619
C	-4.420902	-2.529037	0.107798
O	-5.563183	-2.877961	0.328392
C	-3.502967	-3.406355	-0.710102
H	-2.442034	-3.310226	-0.474188
H	-3.820811	-4.444292	-0.562909
H	-3.667100	-3.147000	-1.767281

PCMopt/19_TS_SSb

Frequencies, energies and thermodynamic properties:

Lowest Vibrational Mode (1/cm) = -222.2279

2nd Lowest Vibrational Mode (1/cm) = 16.9156

E(RM062X) (a.u.) = -2588.53131604

Thermal correction to Enthalpy (a.u.) = 0.663372

Thermal correction to Gibbs Free Energy (a.u.) = 0.548402

Total Entropy (cal/Kmol) = 241.974

Esp(RM062X+D3) (a.u.) = -2591.13230647

Esp(D3) (a.u.) = -0.0100796504

Esp(RM062X) (a.u.) = -2591.1222268196

Optimised cartesian coordinates (Angstrom):

C	-7.421578	-1.446553	-0.538786
C	-7.240042	-2.690626	0.081648

C	-5.988757	-3.079107	0.550708
C	-4.924509	-2.194031	0.383916
C	-5.107100	-0.959739	-0.242561
H	-8.089760	-3.362710	0.202262
H	-5.842185	-4.043992	1.036438
C	-2.865233	-0.835699	0.261487
C	-1.423090	1.088278	-0.232264
C	-2.378788	1.286339	-1.421004
C	-3.816503	1.066622	-0.983255
H	-4.486009	1.035294	-1.852543
S	-3.252468	-2.426407	0.861603
N	-3.914179	-0.230808	-0.310680
N	-1.667188	-0.246262	0.369228
C	-2.169408	2.624713	-2.167156
C	-3.006891	3.789576	-1.630115
C	-2.436343	2.436802	-3.662004
H	-1.104154	2.883225	-2.048864
H	-4.075617	3.639390	-1.849630
H	-2.894064	3.936199	-0.547040
H	-2.701813	4.720060	-2.129197
H	-1.768835	1.679205	-4.096022
H	-3.475978	2.118859	-3.839380
H	-2.281671	3.381080	-4.202652
H	-2.133472	0.480461	-2.131544
H	-0.402886	1.035593	-0.640477
C	-1.483627	2.195480	0.797638
C	-0.531535	3.219432	0.727493
C	-2.480780	2.251455	1.776133
C	-0.605220	4.308999	1.593533
H	0.277852	3.141453	-0.003602
C	-2.552623	3.341453	2.644588
H	-3.207171	1.440448	1.875782
C	-1.622660	4.376957	2.546970
H	0.138651	5.104406	1.529390
H	-3.336723	3.378948	3.402014
H	-1.681264	5.229200	3.225407
C	0.584644	-0.171971	1.311993

H	0.552654	0.912514	1.309646
C	1.794071	-0.819288	1.435858
C	2.875273	-0.113676	2.206704
F	2.620940	-0.184346	3.520175
F	4.091624	-0.642436	2.034048
F	2.958085	1.182839	1.898284
O	-0.695466	-2.159208	1.152938
C	-0.564238	-0.956704	0.978151
H	-4.170698	1.851509	-0.298567
C	-6.360779	-0.562651	-0.710031
H	-6.511995	0.406920	-1.183235
H	-8.412266	-1.160528	-0.892544
H	1.797207	-1.902065	1.569263
C	-0.396431	-2.127221	-2.024665
H	-0.702487	-2.889434	-1.292741
H	-1.251103	-1.494846	-2.287540
H	-0.002169	-2.616385	-2.923095
C	1.795569	-1.809172	-1.273570
O	0.578686	-1.269770	-1.444644
O	2.066390	-2.922976	-1.672725
C	2.678679	0.530873	-0.865475
O	1.691801	1.249137	-0.985391
C	2.722695	-0.911701	-0.568110
N	4.107649	-1.309767	-0.603961
C	4.905435	-0.180621	-0.858540
C	6.298935	-0.047441	-0.942829
C	4.085116	0.948816	-0.988715
C	6.812622	1.229475	-1.165854
H	6.951840	-0.908821	-0.850943
C	4.609544	2.220209	-1.214153
C	5.990062	2.358927	-1.302956
H	7.894989	1.348269	-1.242465
H	3.936193	3.073523	-1.312736
H	6.437374	3.337568	-1.479727
C	4.647154	-2.516455	-0.174150
O	5.813158	-2.778369	-0.393459
C	3.801367	-3.442841	0.666033

H 3.904008 -3.117363 1.712192
H 2.746327 -3.468197 0.389708
H 4.228167 -4.447889 0.578447

PCMopt/19_TS_SSc

Frequencies, energies and thermodynamic properties:

Lowest Vibrational Mode (1/cm) = -265.5046

2nd Lowest Vibrational Mode (1/cm) = 15.7552

E(RM062X) (a.u.) = -2588.53099944

Thermal correction to Enthalpy (a.u.) = 0.663434

Thermal correction to Gibbs Free Energy (a.u.) = 0.547978

Total Entropy (cal/Kmol) = 242.998

Esp(RM062X+D3) (a.u.) = -2591.13165693

Esp(D3) (a.u.) = -0.0098745286

Esp(RM062X) (a.u.) = -2591.1217824014

Optimised cartesian coordinates (Angstrom):

C 7.391823 -1.496497 0.610227
C 7.253849 -2.664138 -0.152639
C 6.032103 -2.999887 -0.729522
C 4.953945 -2.139976 -0.527040
C 5.092893 -0.982113 0.241302
H 8.113698 -3.317817 -0.300011
H 5.919391 -3.906157 -1.325027
C 2.880920 -0.802293 -0.371609
C 1.427346 1.073227 0.213969
C 2.271125 1.106255 1.497310
C 3.741633 0.927295 1.160990
H 4.335975 0.766963 2.070373
S 3.308867 -2.323741 -1.112345
N 3.893678 -0.266818 0.326099
N 1.698006 -0.197965 -0.497043
C 1.991865 2.340734 2.383718
C 2.876915 3.553092 2.079573
C 2.109126 1.962607 3.861554
H 0.942913 2.624242 2.195077
H 3.919794 3.363884 2.379192
H 2.868503 3.831198 1.016320

H	2.525885	4.419844	2.657238
H	1.398977	1.166596	4.126624
H	3.124604	1.606692	4.097473
H	1.904816	2.832682	4.501520
H	1.948853	0.214365	2.059553
H	0.379798	1.006038	0.535914
C	1.596026	2.275689	-0.688404
C	0.651577	3.306137	-0.604347
C	2.680736	2.411258	-1.561361
C	0.816043	4.474296	-1.347656
H	-0.221219	3.171117	0.040156
C	2.842923	3.579110	-2.307447
H	3.406945	1.601767	-1.673062
C	1.917158	4.616992	-2.193401
H	0.077243	5.273625	-1.272476
H	3.694469	3.676477	-2.982342
H	2.046265	5.530502	-2.775663
C	-0.550666	-0.080437	-1.432161
H	-0.502460	1.005130	-1.401037
C	-1.781029	-0.707160	-1.498782
C	-2.866098	-0.000534	-2.262628
F	-2.642433	-0.111028	-3.579894
F	-4.090799	-0.497717	-2.052916
F	-2.920897	1.307337	-1.991201
O	0.749033	-2.065478	-1.413802
C	0.599594	-0.881721	-1.151426
H	4.154431	1.794041	0.623353
C	6.315820	-0.638610	0.819017
H	6.432287	0.272502	1.404966
H	8.359338	-1.250105	1.048087
H	-1.828029	-1.792773	-1.604506
C	-0.888815	-3.408616	2.326089
H	-0.456835	-2.866388	3.177837
H	-1.353229	-4.339066	2.665810
H	-0.090464	-3.623224	1.602242
C	-1.542574	-1.479991	1.183637
O	-1.919614	-2.648048	1.713885

O -0.410403 -1.053715 1.297593
C -2.683284 0.684044 0.759207
O -1.752442 1.475514 0.867358
C -2.609998 -0.754242 0.482471
N -3.951764 -1.279533 0.532100
C -4.847200 -0.216951 0.771968
C -6.244264 -0.177816 0.890770
C -4.119922 0.980289 0.891939
C -6.851815 1.055293 1.128451
H -6.871001 -1.060094 0.823438
C -4.735118 2.207264 1.128449
C -6.119634 2.244596 1.246249
H -7.937895 1.084083 1.230404
H -4.123132 3.106645 1.216810
H -6.636659 3.186196 1.432409
C -4.230769 -2.517742 -0.040001
O -3.354019 -3.157697 -0.580310
C -5.641043 -3.040550 0.041150
H -6.043821 -2.971181 1.058762
H -6.293955 -2.476622 -0.639760
H -5.617209 -4.086480 -0.279644

PCMopt/19_TS_SSd

Frequencies, energies and thermodynamic properties:

Lowest Vibrational Mode (1/cm) = -183.8079

2nd Lowest Vibrational Mode (1/cm) = 19.2791

E(RM062X) (a.u.) = -2588.52852015

Thermal correction to Enthalpy (a.u.) = 0.663682

Thermal correction to Gibbs Free Energy (a.u.) = 0.549269

Total Entropy (cal/Kmol) = 240.802

Esp(RM062X+D3) (a.u.) = -2591.13324369

Esp(D3) (a.u.) = -0.0109897530

Esp(RM062X) (a.u.) = -2591.1222539370

Optimised cartesian coordinates (Angstrom):

C 6.800432 -1.802600 -0.512624
C 6.414949 -2.944670 -1.230083
C 5.083864 -3.156661 -1.572971

C	4.147167	-2.201244	-1.178138
C	4.533060	-1.070218	-0.457162
H	7.167870	-3.675125	-1.526405
H	4.778410	-4.040428	-2.133486
C	2.271289	-0.689492	-0.643028
C	1.084190	1.234832	0.327926
C	2.236589	1.203360	1.345645
C	3.556258	0.975340	0.632893
H	4.365415	0.820490	1.357259
S	2.420272	-2.211773	-1.475638
N	3.439851	-0.247742	-0.163543
N	1.124930	0.002503	-0.498017
C	2.255784	2.415444	2.303106
C	3.075859	3.606389	1.797600
C	2.763778	1.979204	3.679142
H	1.207706	2.736111	2.422876
H	4.151213	3.368626	1.798732
H	2.794372	3.923224	0.783940
H	2.932965	4.463718	2.470246
H	2.125668	1.196265	4.113803
H	3.790274	1.585024	3.613428
H	2.779253	2.831527	4.372754
H	2.054557	0.306441	1.956597
H	0.160172	1.170415	0.918806
C	1.034478	2.488479	-0.517134
C	0.215624	3.543235	-0.096397
C	1.811276	2.638631	-1.668886
C	0.202946	4.746960	-0.797806
H	-0.414640	3.410629	0.788127
C	1.798108	3.844570	-2.371432
H	2.426046	1.811774	-2.034124
C	1.001661	4.902248	-1.932497
H	-0.435906	5.564462	-0.461095
H	2.409834	3.954833	-3.267725
H	0.992494	5.843855	-2.483044
C	-1.235707	0.368107	-1.111907
H	-1.161447	1.423354	-0.865925

C	-2.450197	-0.185209	-1.440645
C	-3.518747	0.730577	-1.983604
F	-3.539756	0.618946	-3.318237
F	-4.759408	0.464326	-1.565583
F	-3.287005	2.017905	-1.708607
O	-0.039974	-1.598142	-1.628397
C	-0.076857	-0.492458	-1.118299
H	3.832503	1.811199	-0.027325
C	5.869016	-0.848864	-0.116421
H	6.179311	0.040347	0.430732
H	7.850709	-1.654021	-0.261188
H	-2.500185	-1.189084	-1.868392
C	-6.748248	-1.736184	-0.114709
H	-7.104197	-0.720119	-0.336281
H	-7.108489	-2.434560	-0.876296
H	-7.119046	-2.031316	0.875799
C	-4.690778	-1.009297	0.716933
O	-5.332902	-1.786272	-0.164753
O	-5.281592	-0.352637	1.550198
C	-2.605505	-2.366381	0.242205
O	-2.979663	-3.246816	-0.518835
C	-3.238016	-1.071695	0.545470
N	-2.404993	-0.392625	1.499489
C	-1.276745	-1.197724	1.774576
C	-0.177863	-0.977884	2.620341
C	-1.360195	-2.382635	1.036624
C	0.814209	-1.958162	2.659020
H	-0.102741	-0.079910	3.225305
C	-0.361861	-3.352297	1.076844
C	0.742334	-3.132037	1.892346
H	1.672961	-1.803968	3.316342
H	-0.459138	-4.249841	0.463524
H	1.547087	-3.866043	1.946698
C	-2.407929	0.962562	1.789181
O	-1.523735	1.456471	2.474407
C	-3.520091	1.838058	1.275509
H	-4.103972	1.416814	0.460159

H -4.208576 2.019880 2.112080
H -3.069700 2.793698 0.979933

PCMopt/19_TS_SSe

Frequencies, energies and thermodynamic properties:

Lowest Vibrational Mode (1/cm) = -226.4565

2nd Lowest Vibrational Mode (1/cm) = 18.0469

E(RM062X) (a.u.) = -2588.52916214

Thermal correction to Enthalpy (a.u.) = 0.663205

Thermal correction to Gibbs Free Energy (a.u.) = 0.548358

Total Entropy (cal/Kmol) = 241.717

Esp(RM062X+D3) (a.u.) = -2591.13092601

Esp(D3) (a.u.) = -0.0099846481

Esp(RM062X) (a.u.) = -2591.1209413619

Optimised cartesian coordinates (Angstrom):

C -7.430585 -1.411060 -0.533294
C -7.262475 -2.651857 0.097261
C -6.016287 -3.047777 0.573879
C -4.943491 -2.173670 0.404398
C -5.112747 -0.942694 -0.232348
H -8.118580 -3.315424 0.219901
H -5.880143 -4.010220 1.067477
C -2.871419 -0.834453 0.279226
C -1.411925 1.071612 -0.224164
C -2.358971 1.267457 -1.419746
C -3.801111 1.064179 -0.988501
H -4.466049 1.028768 -1.861205
S -3.274995 -2.417658 0.889447
N -3.913211 -0.225042 -0.302321
N -1.669245 -0.255753 0.386800
C -2.131507 2.596998 -2.176129
C -2.960893 3.774699 -1.654923
C -2.389964 2.398920 -3.671145
H -1.064372 2.845152 -2.052243
H -4.029563 3.633018 -1.880329
H -2.853888 3.929380 -0.572379
H -2.643426 4.697925 -2.159781

H	-1.727188	1.630806	-4.093822
H	-3.431500	2.089843	-3.853109
H	-2.221940	3.336784	-4.218987
H	-2.115258	0.453245	-2.121414
H	-0.390564	1.008638	-0.628121
C	-1.468558	2.187703	0.796351
C	-0.505245	3.201111	0.724059
C	-2.470966	2.261885	1.768271
C	-0.572933	4.298218	1.581060
H	0.307776	3.107358	-0.001234
C	-2.536651	3.359387	2.627802
H	-3.206327	1.459211	1.869718
C	-1.595338	4.384362	2.527761
H	0.179597	5.085357	1.515425
H	-3.324817	3.411003	3.380189
H	-1.649098	5.242489	3.199173
C	0.585514	-0.197259	1.318950
H	0.545946	0.887378	1.330788
C	1.804147	-0.830620	1.414608
C	2.878725	-0.100803	2.173325
F	2.645416	-0.193689	3.490965
F	4.110763	-0.580509	1.981664
F	2.913963	1.203783	1.881667
O	-0.717342	-2.174288	1.182586
C	-0.570437	-0.976223	0.996873
H	-4.152726	1.859469	-0.314483
C	-6.361139	-0.538086	-0.707372
H	-6.501849	0.429164	-1.188507
H	-8.417368	-1.118830	-0.892870
H	1.868099	-1.916628	1.500187
C	-0.442724	-2.172266	-1.986595
H	-0.713316	-2.951116	-1.258390
H	-1.319250	-1.557198	-2.218225
H	-0.063880	-2.641986	-2.902228
C	1.761054	-1.827734	-1.283380
O	0.528928	-1.301982	-1.423561
O	2.035740	-2.930831	-1.698023

C 2.659090 0.524353 -0.909293
O 1.680895 1.257248 -1.042624
C 2.691527 -0.905881 -0.613408
N 4.067667 -1.327740 -0.617972
C 4.887836 -0.205818 -0.844091
C 6.281630 -0.059195 -0.904963
C 4.073743 0.928971 -1.003711
C 6.800069 1.214951 -1.136665
H 6.971257 -0.889066 -0.794805
C 4.600814 2.197592 -1.232711
C 5.981906 2.341482 -1.300063
H 7.883808 1.328615 -1.195201
H 3.924911 3.046174 -1.352991
H 6.430947 3.318750 -1.479050
C 4.426912 -2.529919 -0.011021
O 3.609377 -3.184461 0.598114
C 5.855030 -2.991140 -0.146373
H 6.240392 -2.846342 -1.162332
H 6.494075 -2.442585 0.560296
H 5.879888 -4.053160 0.117692

PCMopt/19_TS_SSf

Frequencies, energies and thermodynamic properties:

Lowest Vibrational Mode (1/cm) = -186.6567

2nd Lowest Vibrational Mode (1/cm) = 18.0257

E(RM062X) (a.u.) = -2588.52622169

Thermal correction to Enthalpy (a.u.) = 0.663590

Thermal correction to Gibbs Free Energy (a.u.) = 0.548905

Total Entropy (cal/Kmol) = 241.374

Esp(RM062X+D3) (a.u.) = -2591.13128031

Esp(D3) (a.u.) = -0.0109747618

Esp(RM062X) (a.u.) = -2591.1203055482

Optimised cartesian coordinates (Angstrom):

C 6.881346 -1.577636 -0.280240
C 6.575032 -2.714354 -1.042850
C 5.271192 -2.966600 -1.456361
C 4.280230 -2.057368 -1.085582

C	4.587585	-0.931823	-0.319620
H	7.368789	-3.408622	-1.318919
H	5.028055	-3.846126	-2.052831
C	2.324424	-0.629206	-0.606689
C	1.023513	1.227325	0.346898
C	2.123630	1.211491	1.420964
C	3.484732	1.046821	0.771750
H	4.260711	0.899462	1.533189
S	2.570716	-2.123584	-1.466923
N	3.451388	-0.157812	-0.059889
N	1.147725	0.016516	-0.501796
C	2.050251	2.399941	2.405025
C	2.849305	3.631681	1.968294
C	2.505893	1.949009	3.794590
H	0.986218	2.679480	2.479130
H	3.931458	3.432847	2.017456
H	2.605752	3.963077	0.949485
H	2.642057	4.466493	2.652430
H	1.877554	1.132524	4.179059
H	3.548601	1.594665	3.770818
H	2.455065	2.784020	4.507305
H	1.944030	0.294460	2.002076
H	0.073794	1.118693	0.888283
C	0.973209	2.497414	-0.473330
C	0.094962	3.512889	-0.076455
C	1.805056	2.701074	-1.577451
C	0.076047	4.730920	-0.752630
H	-0.577183	3.338156	0.769151
C	1.785776	3.921288	-2.254706
H	2.468757	1.905198	-1.925349
C	0.928572	4.939886	-1.838476
H	-0.609643	5.517454	-0.434840
H	2.440699	4.073368	-3.113587
H	0.914639	5.892718	-2.369256
C	-1.192330	0.313070	-1.216021
H	-1.166747	1.362368	-0.937825
C	-2.373257	-0.268975	-1.615773

C	-3.439231	0.631271	-2.188648
F	-3.379143	0.566382	-3.525789
F	-4.689276	0.310062	-1.851945
F	-3.266100	1.915507	-1.858575
O	0.097276	-1.595577	-1.725791
C	-0.005502	-0.505400	-1.191021
H	3.765464	1.908587	0.147651
C	5.895848	-0.669678	0.091807
H	6.144862	0.216387	0.674251
H	7.911639	-1.396600	0.026467
H	-2.368613	-1.257894	-2.079836
C	-6.617766	-0.676581	1.558001
H	-6.985634	-1.702584	1.691120
H	-6.862125	-0.070970	2.436170
H	-7.083844	-0.249021	0.659766
C	-4.693386	-1.318233	0.382567
O	-5.203256	-0.660806	1.442254
O	-5.390734	-1.950623	-0.376279
C	-2.540205	-2.486447	-0.013237
O	-2.864276	-3.353622	-0.810645
C	-3.231738	-1.224213	0.294278
N	-2.468046	-0.545465	1.305400
C	-1.325510	-1.319092	1.609438
C	-0.275042	-1.085042	2.511047
C	-1.333008	-2.485599	0.837187
C	0.747535	-2.032135	2.571470
H	-0.259855	-0.201270	3.141200
C	-0.304563	-3.422036	0.900336
C	0.751790	-3.186971	1.772829
H	1.569626	-1.867110	3.271594
H	-0.340974	-4.305278	0.260209
H	1.578019	-3.894926	1.846858
C	-2.533138	0.800872	1.623823
O	-1.699757	1.312424	2.357494
C	-3.655010	1.645958	1.077620
H	-4.213293	1.204250	0.253666
H	-4.357547	1.831047	1.902119

H -3.220059 2.606749 0.775278

PCMopt/19_TS_SSg

Frequencies, energies and thermodynamic properties:

Lowest Vibrational Mode (1/cm) = -153.5861

2nd Lowest Vibrational Mode (1/cm) = 25.1220

E(RM062X) (a.u.) = -2588.52212130

Thermal correction to Enthalpy (a.u.) = 0.663601

Thermal correction to Gibbs Free Energy (a.u.) = 0.550122

Total Entropy (cal/Kmol) = 238.836

Esp(RM062X+D3) (a.u.) = -2591.13030632

Esp(D3) (a.u.) = -0.0109804810

Esp(RM062X) (a.u.) = -2591.1193258390

Optimised cartesian coordinates (Angstrom):

C 6.642381 -2.103103 -0.472375

C 6.196267 -3.197623 -1.228427

C 4.860417 -3.315029 -1.595788

C 3.979469 -2.314045 -1.184355

C 4.424998 -1.232374 -0.423379

H 6.906258 -3.965254 -1.536469

H 4.509597 -4.160188 -2.188199

C 2.195517 -0.705693 -0.623940

C 1.140306 1.302679 0.327446

C 2.274858 1.216876 1.361875

C 3.581510 0.858508 0.680904

H 4.353759 0.635601 1.427397

S 2.263868 -2.197183 -1.517034

N 3.381306 -0.351416 -0.118518

N 1.087719 0.048464 -0.460849

C 2.385464 2.465820 2.266959

C 3.319401 3.555325 1.731173

C 2.814384 2.049393 3.675341

H 1.372961 2.895748 2.342792

H 4.369397 3.225447 1.766264

H 3.084236 3.851137 0.699168

H 3.239502 4.450069 2.364096

H 2.092121 1.352962 4.125332

H	3.798799	1.555794	3.657893
H	2.895179	2.928727	4.329416
H	2.011399	0.363936	2.004681
H	0.204859	1.324079	0.903157
C	1.202762	2.519380	-0.572331
C	0.461065	3.657926	-0.239103
C	2.028921	2.547447	-1.700417
C	0.572207	4.820081	-1.000294
H	-0.212192	3.636738	0.622060
C	2.140357	3.710544	-2.462482
H	2.589691	1.658951	-2.001726
C	1.419311	4.850998	-2.108825
H	-0.009450	5.702064	-0.729602
H	2.790656	3.722268	-3.337954
H	1.506800	5.759880	-2.705406
C	-1.272042	0.547071	-1.007462
H	-1.181066	1.568848	-0.651291
C	-2.487341	0.070991	-1.396490
C	-3.574347	1.059015	-1.742922
F	-3.672779	1.092857	-3.086599
F	-4.788477	0.758378	-1.294393
F	-3.294538	2.302207	-1.356060
O	-0.139814	-1.395601	-1.720570
C	-0.129412	-0.344137	-1.111826
H	3.951689	1.659504	0.023513
C	5.767427	-1.104510	-0.059860
H	6.125449	-0.252893	0.517062
H	7.696110	-2.028670	-0.203298
H	-2.585726	-0.896291	-1.894019
C	-6.648887	-1.772321	-0.628141
H	-7.009213	-0.738389	-0.727050
H	-6.904088	-2.344772	-1.525798
H	-7.121985	-2.224685	0.254150
C	-4.723308	-1.159660	0.545136
O	-5.238430	-1.798295	-0.522738
O	-5.427928	-0.649749	1.390625
C	-2.519557	-2.377176	0.126493

O	-2.786282	-3.226028	-0.722787
C	-3.268527	-1.207803	0.542556
N	-2.552602	-0.542531	1.581573
C	-1.345752	-1.229258	1.812563
C	-0.275247	-0.985122	2.689683
C	-1.294183	-2.343446	0.958489
C	0.807728	-1.864672	2.665365
H	-0.260249	-0.158059	3.392877
C	-0.204496	-3.209550	0.938504
C	0.861052	-2.966568	1.798372
H	1.636181	-1.685000	3.353992
H	-0.204579	-4.048494	0.239762
H	1.731765	-3.623606	1.806146
C	-2.815032	0.793856	1.882960
O	-3.579736	1.442235	1.204605
C	-2.157439	1.386942	3.107091
H	-2.141516	0.683031	3.947311
H	-1.125589	1.698233	2.889041
H	-2.729340	2.280555	3.377220

PCMopt/19_TS_SSh

Frequencies, energies and thermodynamic properties:

Lowest Vibrational Mode (1/cm) = -159.7020

2nd Lowest Vibrational Mode (1/cm) = 18.9236

E(RM062X) (a.u.) = -2588.52039376

Thermal correction to Enthalpy (a.u.) = 0.663505

Thermal correction to Gibbs Free Energy (a.u.) = 0.549312

Total Entropy (cal/Kmol) = 240.340

Esp(RM062X+D3) (a.u.) = -2591.12845628

Esp(D3) (a.u.) = -0.0109849920

Esp(RM062X) (a.u.) = -2591.1174712880

Optimised cartesian coordinates (Angstrom):

C	6.713682	-1.927329	-0.289005
C	6.332754	-3.007870	-1.098815
C	5.018903	-3.147194	-1.532130
C	4.093485	-2.182794	-1.131495
C	4.473710	-1.115149	-0.317237

H	7.076665	-3.746839	-1.396730
H	4.717939	-3.981312	-2.166194
C	2.241708	-0.641046	-0.605651
C	1.086889	1.305411	0.357476
C	2.177821	1.220883	1.437730
C	3.522574	0.920418	0.804522
H	4.269342	0.700417	1.577415
S	2.392994	-2.100087	-1.541381
N	3.393125	-0.271804	-0.034929
N	1.106705	0.076116	-0.469492
C	2.212690	2.443671	2.383132
C	3.131842	3.577576	1.918687
C	2.600543	1.995931	3.793851
H	1.184123	2.838310	2.431277
H	4.189541	3.279005	1.987107
H	2.929317	3.897922	0.887191
H	2.998100	4.449330	2.574219
H	1.884371	1.263540	4.193559
H	3.600064	1.533306	3.799046
H	2.628779	2.855856	4.477447
H	1.912318	0.341611	2.043132
H	0.127043	1.282307	0.891321
C	1.148857	2.550296	-0.502643
C	0.355337	3.653118	-0.169397
C	2.022114	2.639696	-1.591335
C	0.461235	4.841828	-0.889246
H	-0.354633	3.581098	0.658907
C	2.128198	3.829234	-2.312216
H	2.623880	1.778880	-1.894028
C	1.355035	4.934546	-1.956909
H	-0.160984	5.695838	-0.619034
H	2.815296	3.889233	-3.157066
H	1.438614	5.864120	-2.521320
C	-1.244612	0.515508	-1.094811
H	-1.204937	1.524339	-0.695591
C	-2.429450	0.011404	-1.545575
C	-3.534265	0.978325	-1.901411

F	-3.598613	1.035189	-3.246594
F	-4.751407	0.642135	-1.490214
F	-3.294722	2.221373	-1.485882
O	-0.018557	-1.360105	-1.829263
C	-0.070033	-0.330605	-1.185028
H	3.895069	1.750014	0.184908
C	5.793950	-0.965033	0.112739
H	6.101689	-0.123443	0.731814
H	7.751438	-1.834819	0.031699
H	-2.470467	-0.932439	-2.093729
C	-6.752798	-0.838326	1.034610
H	-7.174487	-1.851683	0.986671
H	-7.136841	-0.313447	1.915167
H	-7.035591	-0.298564	0.119917
C	-4.674839	-1.443016	0.149721
O	-5.344104	-0.878287	1.172486
O	-5.249626	-2.004534	-0.759057
C	-2.415716	-2.491021	-0.129439
O	-2.621933	-3.319412	-1.013581
C	-3.227689	-1.361479	0.282359
N	-2.588883	-0.707415	1.380178
C	-1.369084	-1.361307	1.645998
C	-0.348188	-1.107353	2.578198
C	-1.235534	-2.445100	0.762194
C	0.765494	-1.947952	2.579308
H	-0.391739	-0.299913	3.302637
C	-0.116309	-3.272977	0.768762
C	0.898302	-3.021025	1.685247
H	1.555122	-1.759587	3.309928
H	-0.053008	-4.088363	0.045509
H	1.790637	-3.647674	1.715337
C	-2.902832	0.610186	1.715801
O	-3.650405	1.264608	1.024660
C	-2.331138	1.173075	2.996469
H	-2.320229	0.432083	3.804024
H	-1.307663	1.545944	2.844841
H	-2.959444	2.024457	3.277881

PCMopt/19_TS_SSi

Frequencies, energies and thermodynamic properties:

Lowest Vibrational Mode (1/cm) = -159.6560

2nd Lowest Vibrational Mode (1/cm) = 19.1207

E(RM062X) (a.u.) = -2588.52039374

Thermal correction to Enthalpy (a.u.) = 0.663505

Thermal correction to Gibbs Free Energy (a.u.) = 0.549325

Total Entropy (cal/Kmol) = 240.313

Esp(RM062X+D3) (a.u.) = -2591.12845759

Esp(D3) (a.u.) = -0.0109851091

Esp(RM062X) (a.u.) = -2591.1174724809

Optimised cartesian coordinates (Angstrom):

C	6.713406	-1.927583	-0.288571
C	6.332518	-3.007982	-1.098589
C	5.018728	-3.147133	-1.532141
C	4.093326	-2.182706	-1.131529
C	4.473508	-1.115204	-0.317064
H	7.076413	-3.746977	-1.396477
H	4.717797	-3.981140	-2.166366
C	2.241585	-0.640903	-0.605738
C	1.086791	1.305550	0.357426
C	2.177582	1.220881	1.437807
C	3.522385	0.920344	0.804741
H	4.269044	0.700240	1.577710
S	2.392911	-2.099803	-1.541677
N	3.392947	-0.271812	-0.034809
N	1.106599	0.076297	-0.469604
C	2.212444	2.443606	2.383289
C	3.131707	3.577484	1.918997
C	2.600162	1.995743	3.794007
H	1.183896	2.838305	2.431376
H	4.189382	3.278850	1.987510
H	2.929308	3.897893	0.887496
H	2.997946	4.449214	2.574558
H	1.883898	1.263396	4.193632
H	3.599635	1.533015	3.799235

H	2.628443	2.855623	4.477657
H	1.911933	0.341597	2.043124
H	0.126866	1.282531	0.891129
C	1.148939	2.550466	-0.502634
C	0.355441	3.653310	-0.169413
C	2.022315	2.639873	-1.591230
C	0.461475	4.842052	-0.889189
H	-0.354617	3.581274	0.658817
C	2.128536	3.829443	-2.312038
H	2.624068	1.779041	-1.893907
C	1.355393	4.934777	-1.956754
H	-0.160728	5.696082	-0.619000
H	2.815725	3.889451	-3.156813
H	1.439079	5.864375	-2.521110
C	-1.244706	0.515793	-1.094904
H	-1.205040	1.524572	-0.695549
C	-2.429545	0.011731	-1.545662
C	-3.534460	0.978647	-1.901188
F	-3.599033	1.035697	-3.246352
F	-4.751513	0.642333	-1.489833
F	-3.294909	2.221646	-1.485519
O	-0.018605	-1.359656	-1.829705
C	-0.070098	-0.330290	-1.185266
H	3.895016	1.749947	0.185219
C	5.793692	-0.965260	0.113149
H	6.101402	-0.123782	0.732390
H	7.751116	-1.835208	0.032321
H	-2.470601	-0.932061	-2.093901
C	-6.752526	-0.838443	1.035332
H	-7.174250	-1.851784	0.987374
H	-7.136357	-0.313654	1.916036
H	-7.035516	-0.298570	0.120765
C	-4.674778	-1.443073	0.149895
O	-5.343802	-0.878450	1.172883
O	-5.249796	-2.004465	-0.758814
C	-2.415694	-2.491016	-0.129929
O	-2.622008	-3.319202	-1.014249

C -3.227606 -1.361576 0.282232
N -2.588627 -0.707706 1.380065
C -1.368817 -1.361673 1.645613
C -0.347802 -1.107906 2.577725
C -1.235406 -2.445306 0.761587
C 0.765851 -1.948547 2.578572
H -0.391245 -0.300575 3.302293
C -0.116221 -3.273238 0.767909
C 0.898500 -3.021488 1.684333
H 1.555580 -1.760313 3.309118
H -0.053027 -4.088505 0.044512
H 1.790806 -3.648187 1.714225
C -2.902505 0.609841 1.715965
O -3.650085 1.264440 1.024997
C -2.330708 1.172442 2.996718
H -2.319596 0.431231 3.804067
H -1.307303 1.545492 2.845046
H -2.959079 2.023670 3.278453

PCMopt/19_TS_SSj

Frequencies, energies and thermodynamic properties:

Lowest Vibrational Mode (1/cm) = -212.8547

2nd Lowest Vibrational Mode (1/cm) = 17.6807

E(RM062X) (a.u.) = -2588.51756560

Thermal correction to Enthalpy (a.u.) = 0.663299

Thermal correction to Gibbs Free Energy (a.u.) = 0.548360

Total Entropy (cal/Kmol) = 241.910

Esp(RM062X+D3) (a.u.) = -2591.12029126

Esp(D3) (a.u.) = -0.0108754548

Esp(RM062X) (a.u.) = -2591.1094158052

Optimised cartesian coordinates (Angstrom):

C 6.886997 -1.679907 -0.270908
C 6.552360 -2.844122 -0.977952
C 5.238914 -3.092636 -1.362468
C 4.267524 -2.152237 -1.018854
C 4.602865 -0.999079 -0.307718
H 7.331162 -3.562739 -1.233583

H	4.973248	-3.993167	-1.916581
C	2.340187	-0.673372	-0.577196
C	1.086809	1.254015	0.292877
C	2.206440	1.296335	1.345011
C	3.553951	1.053518	0.692363
H	4.335413	0.932597	1.452911
S	2.554120	-2.202340	-1.385041
N	3.482655	-0.195801	-0.067579
N	1.172528	-0.014387	-0.472086
C	2.174252	2.561661	2.232003
C	2.998367	3.734108	1.691282
C	2.628862	2.213516	3.651037
H	1.119417	2.877778	2.289893
H	4.075885	3.512142	1.742717
H	2.748348	3.990743	0.652530
H	2.821067	4.624756	2.310337
H	1.978563	1.452341	4.105705
H	3.659794	1.825527	3.650742
H	2.610294	3.105924	4.292265
H	2.015792	0.432493	1.999015
H	0.144715	1.203079	0.856194
C	1.049201	2.456338	-0.625525
C	0.184535	3.512345	-0.315676
C	1.888635	2.561553	-1.738619
C	0.190418	4.675566	-1.083418
H	-0.500122	3.412094	0.531354
C	1.893495	3.725543	-2.508045
H	2.542330	1.731928	-2.020836
C	1.052340	4.787700	-2.175723
H	-0.484717	5.494717	-0.832252
H	2.555057	3.799967	-3.372081
H	1.057723	5.697550	-2.777393
C	-1.197901	0.246344	-1.078785
H	-1.204316	1.259253	-0.687590
C	-2.376943	-0.327195	-1.493104
C	-3.492824	0.617566	-1.837383
F	-3.210668	1.283488	-2.965054

F	-4.664016	0.014595	-2.043678
F	-3.676480	1.541846	-0.884940
O	0.107154	-1.619770	-1.691409
C	0.003938	-0.542072	-1.130599
H	3.849843	1.868148	0.014334
C	5.921035	-0.740368	0.073364
H	6.192408	0.166090	0.612744
H	7.924205	-1.502138	0.013677
H	-2.383453	-1.272932	-2.040936
C	-6.826841	-0.465277	0.378829
H	-7.302214	-1.381015	0.753838
H	-7.251618	0.412828	0.876380
H	-6.982975	-0.401628	-0.706383
C	-4.712845	-1.446970	0.141784
O	-5.440575	-0.456475	0.676594
O	-5.228857	-2.328168	-0.508249
C	-2.461323	-2.533403	0.118572
O	-2.675568	-3.450238	-0.665242
C	-3.271210	-1.332320	0.374249
N	-2.598193	-0.547306	1.393231
C	-1.371337	-1.185854	1.674718
C	-0.357613	-0.838388	2.588766
C	-1.272966	-2.386518	0.968068
C	0.722262	-1.704376	2.724213
H	-0.405471	0.075484	3.175905
C	-0.173454	-3.237513	1.091983
C	0.838474	-2.890129	1.974038
H	1.507767	-1.449782	3.439382
H	-0.133317	-4.148617	0.492246
H	1.714303	-3.528134	2.097791
C	-2.753418	0.798800	1.789283
O	-1.759664	1.473880	1.986285
C	-4.105605	1.377182	2.114834
H	-4.637585	1.667040	1.204145
H	-4.729141	0.656546	2.651403
H	-3.912178	2.265706	2.725261

PCMopt/19_TS_SSk

Frequencies, energies and thermodynamic properties:

Lowest Vibrational Mode (1/cm) = -384.9261

2nd Lowest Vibrational Mode (1/cm) = 14.2537

E(RM062X) (a.u.) = -2588.51905567

Thermal correction to Enthalpy (a.u.) = 0.663101

Thermal correction to Gibbs Free Energy (a.u.) = 0.549019

Total Entropy (cal/Kmol) = 240.108

Esp(RM062X+D3) (a.u.) = -2591.11995660

Esp(D3) (a.u.) = -0.0106748388

Esp(RM062X) (a.u.) = -2591.1092817612

Optimised cartesian coordinates (Angstrom):

C	-6.717982	2.088455	0.005560
C	-6.341013	3.215474	-0.737414
C	-5.033196	3.366862	-1.190061
C	-4.112429	2.366231	-0.882815
C	-4.490051	1.249950	-0.134644
H	-7.080447	3.982633	-0.967770
H	-4.735385	4.240474	-1.770474
C	-2.273751	0.751511	-0.524473
C	-1.150388	-1.275589	0.243427
C	-2.149647	-1.190640	1.409811
C	-3.528444	-0.833809	0.884426
H	-4.212053	-0.606812	1.712826
S	-2.410410	2.304220	-1.312279
N	-3.418672	0.372744	0.064298
N	-1.178047	-0.009979	-0.522330
C	-2.150623	-2.439881	2.319870
C	-3.126231	-3.539849	1.889756
C	-2.431123	-2.030183	3.767279
H	-1.130024	-2.858788	2.282493
H	-4.169091	-3.219518	2.040254
H	-3.003755	-3.833367	0.838004
H	-2.969064	-4.433565	2.509842
H	-1.677327	-1.320385	4.136693
H	-3.419441	-1.552098	3.855284
H	-2.427172	-2.909756	4.426269

H	-1.805299	-0.334379	2.013507
H	-0.146611	-1.330276	0.687188
C	-1.325992	-2.482112	-0.653401
C	-0.526914	-3.608607	-0.427094
C	-2.291659	-2.518729	-1.663989
C	-0.721848	-4.771781	-1.170111
H	0.260300	-3.561259	0.330646
C	-2.485359	-3.682239	-2.409191
H	-2.896430	-1.635074	-1.883840
C	-1.708751	-4.813563	-2.156245
H	-0.096260	-5.645845	-0.983831
H	-3.243941	-3.702863	-3.192797
H	-1.862247	-5.722888	-2.738953
C	1.121936	-0.427202	-1.210619
H	0.967776	-1.489207	-1.048677
C	2.437791	0.058255	-1.268316
C	3.442012	-0.831901	-1.948652
F	3.244335	-0.825179	-3.274569
F	4.704188	-0.460018	-1.750910
F	3.336851	-2.110134	-1.555244
O	0.040657	1.667401	-1.490480
C	0.054402	0.496782	-1.121055
H	-3.969254	-1.641286	0.280557
C	-5.800369	1.089527	0.317928
H	-6.102420	0.211132	0.887080
H	-7.749047	1.986486	0.344771
H	2.558046	1.103962	-1.571929
C	4.510406	-2.975243	1.180547
H	4.004928	-3.500614	0.360168
H	5.585255	-3.176111	1.156375
H	4.082582	-3.302029	2.137097
C	3.121074	-1.104072	1.025188
O	4.368247	-1.568802	1.023725
O	2.152487	-1.817535	1.194109
C	4.210413	1.249999	0.513960
O	5.394749	1.010493	0.426435
C	3.047609	0.319397	0.661899

N	1.939109	1.126188	1.090386
C	2.214336	2.465972	0.732196
C	1.377480	3.582939	0.711090
C	3.573534	2.588043	0.416782
C	1.956190	4.809715	0.377422
H	0.317176	3.492427	0.925710
C	4.141308	3.814573	0.088533
C	3.317292	4.938140	0.068670
H	1.319215	5.695546	0.348053
H	5.205531	3.875074	-0.146029
H	3.723994	5.915222	-0.193476
C	0.876153	0.831742	1.945788
O	-0.161401	1.461465	1.853907
C	1.069542	-0.148308	3.079217
H	0.582167	-1.106174	2.857117
H	2.122226	-0.341385	3.307148
H	0.575378	0.305188	3.948005

PCMopt/19_TS_SSl

Frequencies, energies and thermodynamic properties:

Lowest Vibrational Mode (1/cm) = -369.3854

2nd Lowest Vibrational Mode (1/cm) = 22.9114

E(RM062X) (a.u.) = -2588.51491205

Thermal correction to Enthalpy (a.u.) = 0.663363

Thermal correction to Gibbs Free Energy (a.u.) = 0.549945

Total Entropy (cal/Kmol) = 238.707

Esp(RM062X+D3) (a.u.) = -2591.11620455

Esp(D3) (a.u.) = -0.0106700603

Esp(RM062X) (a.u.) = -2591.1055344897

Optimised cartesian coordinates (Angstrom):

C	-6.715923	2.194333	0.086599
C	-6.347137	3.305156	-0.684516
C	-5.046122	3.444616	-1.159685
C	-4.124546	2.446672	-0.846685
C	-4.493190	1.346545	-0.070138
H	-7.087971	4.069656	-0.919001
H	-4.754753	4.305814	-1.761302

C	-2.286657	0.833196	-0.482000
C	-1.191866	-1.224536	0.243870
C	-2.138571	-1.115760	1.447922
C	-3.528302	-0.717239	0.985973
H	-4.163393	-0.449576	1.840827
S	-2.428786	2.372823	-1.294072
N	-3.421501	0.470200	0.133638
N	-1.189585	0.069566	-0.479009
C	-2.120520	-2.367721	2.352990
C	-3.164669	-3.426922	1.990104
C	-2.266519	-1.949364	3.817035
H	-1.120203	-2.817184	2.237267
H	-4.182888	-3.068247	2.208613
H	-3.121820	-3.720062	0.931403
H	-3.001194	-4.329152	2.596190
H	-1.452553	-1.270416	4.107913
H	-3.224893	-1.432692	3.985521
H	-2.240597	-2.827859	4.477283
H	-1.734644	-0.283547	2.046129
H	-0.181653	-1.336751	0.658615
C	-1.469634	-2.392073	-0.678226
C	-0.712294	-3.559078	-0.520879
C	-2.481255	-2.358443	-1.643859
C	-0.991872	-4.689771	-1.287170
H	0.109390	-3.565001	0.200352
C	-2.758968	-3.489336	-2.412325
H	-3.059058	-1.446151	-1.813234
C	-2.022263	-4.659812	-2.228050
H	-0.397898	-5.595202	-1.154621
H	-3.552467	-3.453389	-3.160006
H	-2.241655	-5.543581	-2.828768
C	1.064701	-0.413938	-1.258290
H	0.840966	-1.471853	-1.161551
C	2.401379	-0.017108	-1.297252
C	3.369093	-0.963599	-1.951922
F	3.171478	-0.991732	-3.277841
F	4.642428	-0.624631	-1.764983

F	3.221919	-2.222905	-1.518324
O	0.074882	1.749757	-1.380383
C	0.045506	0.558102	-1.087606
H	-4.026799	-1.517644	0.418755
C	-5.796682	1.199176	0.405543
H	-6.091424	0.333629	0.997736
H	-7.741974	2.102710	0.443371
H	2.617205	1.030216	-1.532079
C	4.061006	-3.262487	1.358210
H	3.636679	-3.721846	0.455441
H	5.107233	-3.559397	1.478673
H	3.473490	-3.578971	2.230142
C	2.865227	-1.275626	1.066257
O	4.062557	-1.848462	1.237237
O	1.842308	-1.923900	1.072672
C	4.310448	0.862485	0.635816
O	5.446756	0.436130	0.629280
C	3.004076	0.152904	0.714162
N	2.013602	1.154250	1.037567
C	2.549626	2.418168	0.663623
C	1.943966	3.659063	0.442845
C	3.929043	2.285426	0.463832
C	2.761664	4.733306	0.081438
H	0.872776	3.813818	0.506062
C	4.739862	3.356420	0.107916
C	4.145702	4.602393	-0.076342
H	2.292684	5.702514	-0.095396
H	5.810771	3.197685	-0.030625
H	4.745492	5.466589	-0.362718
C	0.948307	0.976968	1.917657
O	0.836180	-0.022170	2.596472
C	-0.072102	2.088011	1.998049
H	0.368138	2.987428	2.450145
H	-0.454707	2.355846	1.003733
H	-0.886291	1.741185	2.643645

PCMopt_microsolv/16_TS_SRa-bi_solv

Frequencies, energies and thermodynamic properties:

Lowest Vibrational Mode (1/cm) = -153.1938

2nd Lowest Vibrational Mode (1/cm) = 19.4318

E(RM062X) (a.u.) = -2995.01373804

Thermal correction to Enthalpy (a.u.) = 0.916384

Thermal correction to Gibbs Free Energy (a.u.) = 0.766593

Total Entropy (cal/Kmol) = 315.261

Esp(RM062X+D3) (a.u.) = -2998.09196882

Esp(D3) (a.u.) = -0.0160270786

Esp(RM062X) (a.u.) = -2998.0759417414

Esp(RM062X+D3) SMD (a.u.) = -2998.11322448

Esp(RM062X) SMD (a.u.) = -2998.0971974014

Optimised cartesian coordinates (Angstrom):

C	5.703440	0.022941	-0.667125
C	5.450242	-0.623472	-1.886341
C	4.148000	-0.835456	-2.323504
C	3.107367	-0.396660	-1.507290
C	3.358942	0.228129	-0.286622
H	6.285209	-0.956767	-2.503360
H	3.943035	-1.329629	-3.273782
C	1.062604	0.257107	-0.295467
C	-0.356874	1.096074	1.549954
C	0.882015	0.776566	2.403227
C	2.142944	1.215079	1.679757
H	3.035412	0.901661	2.235662
S	1.393018	-0.532026	-1.810671
N	2.174304	0.551280	0.380060
N	-0.171607	0.547073	0.179492
C	0.795044	1.298739	3.854771
C	1.355709	2.710226	4.052604
C	1.492928	0.316310	4.797612
H	-0.273483	1.313289	4.124895
H	2.447856	2.722901	3.911254
H	0.910902	3.446124	3.368652
H	1.158328	3.043073	5.081242
H	1.032807	-0.681397	4.746996
H	2.560274	0.214400	4.543911

H	1.432677	0.668816	5.836805
H	0.917280	-0.322773	2.454881
H	-1.183759	0.517200	1.985600
C	-0.733144	2.563239	1.532464
C	-1.709090	3.024471	2.423107
C	-0.082305	3.473352	0.694238
C	-2.012182	4.382827	2.497015
H	-2.238813	2.315550	3.065030
C	-0.391002	4.833177	0.767255
H	0.673233	3.136630	-0.022138
C	-1.348625	5.292010	1.671505
H	-2.773058	4.730825	3.196838
H	0.123843	5.535895	0.110109
H	-1.586172	6.355297	1.726579
C	-2.621138	0.668952	-0.150570
H	-2.746739	1.259150	0.753024
C	-3.708448	0.256328	-0.854957
C	-5.032659	0.902173	-0.558299
F	-5.004300	2.198236	-0.908747
F	-6.040023	0.355013	-1.238275
F	-5.343559	0.869319	0.738813
O	-1.120387	-0.184365	-1.755443
C	-1.307396	0.302646	-0.657081
H	2.192530	2.303656	1.522941
C	4.665115	0.461985	0.148715
H	4.874068	0.976936	1.086126
H	6.733750	0.192475	-0.351502
H	-3.610430	-0.225069	-1.829883
C	-7.441710	-2.449444	-1.802564
H	-7.897293	-1.468938	-1.603842
H	-7.536972	-2.698319	-2.864942
H	-7.961540	-3.202671	-1.193872
C	-5.729264	-2.111279	-0.244559
O	-6.057360	-2.425232	-1.517587
O	-6.591962	-1.928615	0.599723
C	-3.263469	-2.546976	-0.851644
O	-3.268737	-2.854597	-2.043330

C	-4.309908	-2.016053	-0.004715
C	-2.363827	-2.216183	1.321882
C	-1.377885	-2.217906	2.311382
C	-2.048231	-2.625835	0.024499
C	-0.087356	-2.628080	1.968603
H	-1.608507	-1.914005	3.335690
C	-0.755686	-3.014818	-0.326471
C	0.234236	-3.010003	0.655465
H	0.687159	-2.658330	2.738742
H	-0.536823	-3.300874	-1.357634
H	1.263366	-3.285336	0.408663
C	-3.820545	-1.841185	1.409748
H	-3.933312	-0.805730	1.779213
H	-4.360533	-2.485779	2.123670
C	1.842424	2.574144	-2.645958
O	2.183519	2.793581	-1.483232
N	2.757300	2.311861	-3.609165
C	0.377187	2.587201	-3.034548
H	0.178895	3.352025	-3.798399
H	0.051271	1.613817	-3.425600
H	-0.209663	2.821713	-2.138555
C	2.443077	2.086807	-5.005833
H	2.808154	2.919665	-5.627738
H	2.930164	1.162206	-5.350286
H	1.365835	1.983588	-5.158825
C	4.170310	2.402193	-3.288749
H	4.570082	3.397120	-3.546210
H	4.317279	2.222595	-2.218044
H	4.721395	1.645338	-3.862561
O	3.542943	-3.100651	-0.041585
C	4.265358	-2.764764	0.890341
C	3.672054	-2.103873	2.120517
N	5.605066	-2.984418	0.890341
H	3.578496	-2.835909	2.937573
H	2.667943	-1.755201	1.848970
H	4.275002	-1.264025	2.488633
C	6.249201	-3.536457	-0.284735

C 6.485722 -2.583643 1.970082
H 7.038145 -2.853656 -0.637323
H 6.709309 -4.510141 -0.054382
H 5.502424 -3.661661 -1.074095
H 6.874193 -1.560894 1.823429
H 5.980914 -2.637828 2.940276
H 7.339871 -3.272775 2.002434

PCMopt_microsolv/16_TS_SRa-mono_solv

Frequencies, energies and thermodynamic properties:

Lowest Vibrational Mode (1/cm) = -154.4058
2nd Lowest Vibrational Mode (1/cm) = 20.5281
E(RM062X) (a.u.) = -2707.51617704
Thermal correction to Enthalpy (a.u.) = 0.774911
Thermal correction to Gibbs Free Energy (a.u.) = 0.646253
Total Entropy (cal/Kmol) = 270.784
Esp(RM062X+D3) (a.u.) = -2710.26197331
Esp(D3) (a.u.) = -0.0127741745
Esp(RM062X) (a.u.) = -2710.2491991355
Esp(RM062X+D3) SMD (a.u.) = -2710.28120035
Esp(RM062X) SMD (a.u.) = -2710.2684261755

Optimised cartesian coordinates (Angstrom):

C 5.991903 -1.389763 1.621580
C 5.685758 -2.694316 1.206401
C 4.425889 -3.005623 0.706897
C 3.478854 -1.983445 0.641794
C 3.780045 -0.689846 1.070769
H 6.446650 -3.472616 1.267795
H 4.183733 -4.014507 0.371250
C 1.608889 -0.397455 0.384594
C 0.318334 1.704117 0.599961
C 1.335951 2.005503 1.712905
C 2.728699 1.582989 1.281734
H 3.437363 1.700546 2.110221
S 1.841600 -2.088315 0.037155
N 2.685101 0.165948 0.932579
N 0.469908 0.293679 0.149260

C	1.273823	3.455526	2.243301
C	2.179548	4.441174	1.498887
C	1.599105	3.470294	3.738324
H	0.230813	3.793150	2.127933
H	3.241337	4.214654	1.683426
H	2.011039	4.442157	0.413327
H	1.996570	5.459464	1.869798
H	0.890669	2.852066	4.308368
H	2.614468	3.085781	3.924365
H	1.555181	4.494930	4.133160
H	1.057188	1.341380	2.544513
H	-0.672064	1.751943	1.074410
C	0.359828	2.678731	-0.558499
C	-0.532629	3.756666	-0.571917
C	1.312348	2.561815	-1.575079
C	-0.456453	4.723550	-1.572888
H	-1.294998	3.842117	0.207249
C	1.383469	3.529414	-2.579184
H	2.012197	1.720892	-1.594359
C	0.506962	4.614377	-2.577047
H	-1.155292	5.561078	-1.571468
H	2.132100	3.431069	-3.366950
H	0.566688	5.369430	-3.362140
C	-1.817853	0.400084	-0.780017
H	-1.852732	1.478423	-0.652780
C	-2.924664	-0.272144	-1.196755
C	-4.047427	0.530728	-1.791091
F	-3.663663	1.067213	-2.960707
F	-5.129631	-0.200781	-2.055574
F	-4.416348	1.548953	-1.011490
O	-0.463205	-1.528167	-0.848356
C	-0.610553	-0.365768	-0.520990
H	3.102082	2.146466	0.412683
C	5.047921	-0.369684	1.560897
H	5.300600	0.640937	1.878753
H	6.989676	-1.165735	1.999402
H	-2.888777	-1.329866	-1.463820

C	-7.222955	-2.157332	-0.689746
H	-7.404202	-1.321617	-1.380508
H	-7.351937	-3.110578	-1.213433
H	-7.941561	-2.084883	0.138594
C	-5.527712	-1.020599	0.454341
O	-5.890879	-2.135177	-0.217054
O	-6.334328	-0.133574	0.682607
C	-3.256848	-2.140347	0.929940
O	-3.308710	-3.239175	0.378944
C	-4.139551	-0.994585	0.852841
C	-2.368428	-0.426863	2.315424
C	-1.452850	0.161444	3.191526
C	-2.143315	-1.721367	1.842587
C	-0.325946	-0.570226	3.575930
H	-1.615960	1.170043	3.580440
C	-1.013708	-2.451205	2.210426
C	-0.100216	-1.865373	3.084269
H	0.388679	-0.132215	4.276365
H	-0.859663	-3.453844	1.806101
H	0.794546	-2.410329	3.391669
C	-3.666309	0.107855	1.765281
H	-3.503685	1.064581	1.236224
H	-4.386197	0.319502	2.573705
C	3.255186	-0.747340	-2.552217
O	3.511585	0.227349	-1.844402
N	4.159704	-1.737361	-2.738387
C	1.907851	-0.868902	-3.236458
H	2.023236	-0.901027	-4.328963
H	1.368644	-1.771522	-2.918609
H	1.311130	0.011526	-2.970180
C	3.956570	-2.890693	-3.592129
H	4.623148	-2.849399	-4.468170
H	4.181809	-3.810881	-3.032342
H	2.923215	-2.947397	-3.943478
C	5.492873	-1.592608	-2.182049
H	6.185883	-1.163839	-2.924870
H	5.457239	-0.936360	-1.305347

H 5.869337 -2.579569 -1.882214

PCMopt_microsolv/16_TS_SSa-bi_solv

Frequencies, energies and thermodynamic properties:

Lowest Vibrational Mode (1/cm) = -217.0731

2nd Lowest Vibrational Mode (1/cm) = 19.1704

E(RM062X) (a.u.) = -2995.02267528

Thermal correction to Enthalpy (a.u.) = 0.916459

Thermal correction to Gibbs Free Energy (a.u.) = 0.767515

Total Entropy (cal/Kmol) = 313.479

Esp(RM062X+D3) (a.u.) = -2998.09476332

Esp(D3) (a.u.) = -0.0162231615

Esp(RM062X) (a.u.) = -2998.0785401585

Esp(RM062X+D3) SMD (a.u.) = -2998.11503183

Esp(RM062X) SMD (a.u.) = -2998.0988086685

Optimised cartesian coordinates (Angstrom):

C 5.832837 0.023327 -0.448163

C 5.779682 1.044557 0.511164

C 4.576120 1.383402 1.121929

C 3.428972 0.690846 0.739044

C 3.479860 -0.312897 -0.229146

H 6.692270 1.572670 0.789120

H 4.528212 2.169253 1.877194

C 1.235567 -0.319925 0.241632

C -0.458098 -1.659110 -0.939350

C 0.560385 -1.572294 -2.088426

C 1.964999 -1.808227 -1.558017

H 2.709859 -1.632087 -2.345481

S 1.793767 0.947109 1.303229

N 2.217986 -0.841292 -0.494772

N -0.036160 -0.742269 0.148974

C 0.210038 -2.464345 -3.299384

C 0.763340 -3.890844 -3.220110

C 0.679438 -1.799059 -4.594682

H -0.890259 -2.525100 -3.338287

H 1.861473 -3.890651 -3.304923

H 0.490498 -4.405208 -2.288583

H	0.374346	-4.484147	-4.059837
H	0.246717	-0.795165	-4.711415
H	1.777295	-1.705372	-4.612563
H	0.389948	-2.402392	-5.466727
H	0.528267	-0.519093	-2.414829
H	-1.386799	-1.217236	-1.322284
C	-0.730891	-3.058625	-0.433906
C	-1.849169	-3.745557	-0.919791
C	0.134336	-3.701503	0.457689
C	-2.084472	-5.068415	-0.548263
H	-2.542053	-3.238555	-1.596543
C	-0.105858	-5.024862	0.832778
H	1.001592	-3.178118	0.872234
C	-1.208054	-5.713726	0.325675
H	-2.957644	-5.594257	-0.937087
H	0.575922	-5.518176	1.527628
H	-1.391088	-6.748347	0.619487
C	-2.389706	-0.603925	0.886046
H	-2.650198	-1.523811	0.370661
C	-3.368500	0.246743	1.339373
C	-4.720390	-0.312328	1.670550
F	-4.678297	-0.943797	2.854910
F	-5.668446	0.619834	1.780409
F	-5.142693	-1.212375	0.775958
O	-0.687107	0.813590	1.679372
C	-1.040062	-0.112128	0.961462
H	2.108205	-2.826125	-1.164030
C	4.688387	-0.673056	-0.827810
H	4.743436	-1.474784	-1.563896
H	6.787806	-0.237340	-0.905377
H	-3.085255	1.096948	1.965191
C	-5.357961	-0.956868	-2.356388
H	-5.938862	-0.389265	-3.096095
H	-4.790290	-1.749722	-2.854536
H	-6.052123	-1.391959	-1.624175
C	-4.916670	0.922667	-1.038312
O	-4.408640	-0.128317	-1.710014

O	-6.110857	1.156019	-1.033364
C	-2.536855	1.810689	-0.756389
O	-1.852964	0.987495	-1.364399
C	-3.914998	1.705789	-0.327216
C	-3.043145	3.774287	0.450715
C	-2.777287	4.996034	1.069981
C	-2.013482	3.091824	-0.200765
C	-1.480205	5.511225	1.006209
H	-3.564105	5.544224	1.592397
C	-0.713304	3.593566	-0.270793
C	-0.455800	4.818909	0.341911
H	-1.258362	6.468546	1.481437
H	0.069635	3.024461	-0.782338
H	0.548009	5.247302	0.311712
C	-4.332502	2.991415	0.355126
H	-5.088443	3.533387	-0.237527
H	-4.780956	2.826977	1.348462
O	2.687063	-2.397723	1.976599
C	2.465555	-1.740419	2.992712
C	1.054103	-1.607737	3.532501
H	0.737153	-0.558093	3.599021
H	0.380655	-2.139965	2.849988
H	0.972356	-2.057596	4.532200
N	3.467638	-1.115493	3.656616
C	4.834207	-1.273869	3.192905
H	4.837282	-1.500430	2.120574
H	5.384940	-0.339585	3.366140
H	5.344828	-2.086920	3.735110
C	3.298875	-0.330101	4.861912
H	2.258368	-0.333966	5.196293
H	3.923341	-0.737125	5.671967
H	3.605020	0.713149	4.683267
C	3.444547	1.075032	-3.602194
H	3.700278	1.734821	-4.443248
H	2.647969	0.391725	-3.915998
H	4.343271	0.499631	-3.340582
C	2.924899	1.874275	-2.424206

O 1.793976 1.672310 -1.988386
N 3.744745 2.812474 -1.886189
C 3.227741 3.704947 -0.866589
H 2.378969 3.231234 -0.361681
H 4.017440 3.917753 -0.132945
H 2.895359 4.660049 -1.308037
C 5.056337 3.143888 -2.406327
H 5.459366 2.329215 -3.015032
H 5.027533 4.062577 -3.015476
H 5.745346 3.307435 -1.565104

PCMopt_microsolv/16_TS_SSa-mono_solv

Frequencies, energies and thermodynamic properties:

Lowest Vibrational Mode (1/cm) = -223.5684
2nd Lowest Vibrational Mode (1/cm) = 15.6617
E(RM062X) (a.u.) = -2707.51956416
Thermal correction to Enthalpy (a.u.) = 0.775051
Thermal correction to Gibbs Free Energy (a.u.) = 0.645472
Total Entropy (cal/Kmol) = 272.720
Esp(RM062X+D3) (a.u.) = -2710.26049691
Esp(D3) (a.u.) = -0.0124637728
Esp(RM062X) (a.u.) = -2710.2480331372
Esp(RM062X+D3) SMD (a.u.) = -2710.28151947
Esp(RM062X) SMD (a.u.) = -2710.2690556972

Optimised cartesian coordinates (Angstrom):

C 5.900388 -1.186305 2.070765
C 5.745385 -2.455020 1.494928
C 4.573292 -2.794202 0.825213
C 3.562819 -1.837499 0.749946
C 3.711214 -0.582133 1.342074
H 6.554539 -3.182307 1.564407
H 4.448757 -3.774975 0.364631
C 1.622474 -0.346995 0.412450
C 0.163784 1.619127 0.670894
C 0.938677 1.782004 1.988682
C 2.420833 1.536323 1.757013
H 2.969047 1.540448 2.707767

S	2.004709	-1.991520	-0.036816
N	2.574131	0.209212	1.168053
N	0.505877	0.306831	0.063205
C	0.649911	3.110932	2.722091
C	1.572128	4.267049	2.323100
C	0.708704	2.893644	4.235440
H	-0.384773	3.394228	2.466726
H	2.602279	4.083944	2.666957
H	1.596621	4.440057	1.238410
H	1.229814	5.193605	2.805255
H	-0.024184	2.141814	4.560299
H	1.708936	2.551296	4.545001
H	0.499331	3.831468	4.769027
H	0.573281	0.963009	2.630296
H	-0.894894	1.523614	0.950629
C	0.336195	2.762366	-0.305709
C	-0.633745	3.770341	-0.343963
C	1.465072	2.867296	-1.124893
C	-0.463391	4.887804	-1.159568
H	-1.533086	3.675959	0.269398
C	1.631434	3.984545	-1.945739
H	2.223053	2.078148	-1.137129
C	0.675647	5.000599	-1.958322
H	-1.225223	5.668422	-1.176287
H	2.516669	4.057873	-2.579685
H	0.810957	5.873369	-2.598794
C	-1.660505	0.368937	-1.091557
H	-1.709562	1.447815	-0.983741
C	-2.797699	-0.331506	-1.410122
C	-3.877535	0.399558	-2.156621
F	-3.552399	0.495428	-3.455067
F	-5.067387	-0.200492	-2.114441
F	-4.045041	1.650623	-1.712129
O	-0.299491	-1.555948	-0.987707
C	-0.499993	-0.383241	-0.706159
H	2.873142	2.282909	1.086583
C	4.887776	-0.232877	2.006137

H	5.019764	0.752806	2.450894
H	6.831113	-0.936417	2.580635
H	-2.734579	-1.396703	-1.647980
C	-4.233894	2.705641	1.334313
H	-5.069792	2.654155	2.044234
H	-3.440132	3.342312	1.740017
H	-4.603152	3.118440	0.384862
C	-4.521003	0.455050	0.764046
O	-3.660227	1.426191	1.130335
O	-5.710530	0.675222	0.636996
C	-2.661721	-1.254791	1.172147
O	-1.734724	-0.565285	1.598939
C	-3.885665	-0.832615	0.521648
C	-3.865920	-3.205598	0.602336
C	-4.086658	-4.575960	0.464528
C	-2.660305	-2.746977	1.138258
C	-3.090935	-5.461343	0.883953
H	-5.019590	-4.953905	0.041336
C	-1.660224	-3.622844	1.558529
C	-1.885997	-4.991607	1.427547
H	-3.251407	-6.536598	0.787008
H	-0.725834	-3.231309	1.966010
H	-1.124045	-5.705844	1.744016
C	-4.756520	-2.040552	0.245815
H	-5.666354	-2.026914	0.869162
H	-5.099942	-2.102557	-0.798231
O	3.744746	0.619847	-1.508235
C	3.520396	-0.279888	-2.317387
C	2.208322	-0.323452	-3.076989
H	1.651558	-1.249300	-2.876901
H	1.602466	0.531630	-2.753957
H	2.377111	-0.248921	-4.160468
N	4.429261	-1.256890	-2.550266
C	5.725626	-1.190597	-1.900291
H	5.646133	-0.601171	-0.980013
H	6.059530	-2.207071	-1.651999
H	6.475798	-0.727998	-2.562970

C 4.256484 -2.339752 -3.496886
H 3.266030 -2.310456 -3.957985
H 5.014057 -2.279910 -4.293979
H 4.372467 -3.307736 -2.985118

PCMopt_microsolv/9_TS_SRa-bi_solv
Frequencies, energies and thermodynamic properties:
Lowest Vibrational Mode (1/cm) = -244.2795
2nd Lowest Vibrational Mode (1/cm) = 20.9365
E(RM062X) (a.u.) = -2842.74958210
Thermal correction to Enthalpy (a.u.) = 0.890184
Thermal correction to Gibbs Free Energy (a.u.) = 0.747027
Total Entropy (cal/Kmol) = 301.300
Esp(RM062X+D3) (a.u.) = -2845.65828958
Esp(D3) (a.u.) = -0.0150754968
Esp(RM062X) (a.u.) = -2845.6432140832
Esp(RM062X+D3) SMD (a.u.) = -2845.67609581
Esp(RM062X) SMD (a.u.) = -2845.6610203132
Optimised cartesian coordinates (Angstrom):
C 5.708112 -0.174178 0.244763
C 5.736110 -0.388157 -1.140945
C 4.563626 -0.345913 -1.889554
C 3.368003 -0.094394 -1.218439
C 3.338398 0.080207 0.165986
H 6.686681 -0.580450 -1.639096
H 4.577075 -0.497545 -2.969954
C 1.104024 0.287308 -0.310529
C -0.690967 0.494762 1.358476
C 0.316983 -0.255281 2.241357
C 1.709057 0.325228 2.062225
H 2.444142 -0.290813 2.592633
S 1.760181 -0.002345 -1.905415
N 2.039485 0.248724 0.640826
N -0.189216 0.514083 -0.042307
C -0.114959 -0.365774 3.719432
C 0.366008 0.787488 4.605116
C 0.358174 -1.702659 4.293983

H	-1.217869	-0.367318	3.727031
H	1.460984	0.760335	4.722201
H	0.086438	1.773764	4.209045
H	-0.070314	0.690302	5.609613
H	-0.097318	-2.546717	3.756600
H	1.450108	-1.803248	4.198146
H	0.093259	-1.785567	5.357961
H	0.364406	-1.277487	1.832723
H	-1.596241	-0.127883	1.323829
C	-1.058047	1.873913	1.859451
C	-2.239078	2.014765	2.599265
C	-0.233838	2.986478	1.659489
C	-2.571020	3.243735	3.168308
H	-2.900652	1.150702	2.707025
C	-0.573327	4.217326	2.225666
H	0.676575	2.905180	1.057481
C	-1.733921	4.346662	2.989424
H	-3.490709	3.343356	3.747113
H	0.075969	5.080172	2.067069
H	-1.993447	5.309040	3.433387
C	-2.514303	0.747984	-0.781663
H	-2.783533	1.258174	0.139576
C	-3.487016	0.160583	-1.557543
C	-4.860425	0.761163	-1.588899
F	-4.820834	1.998262	-2.107523
F	-5.705219	0.066155	-2.357339
F	-5.416963	0.880161	-0.378844
O	-0.742591	0.161187	-2.230786
C	-1.149455	0.453462	-1.111893
H	1.783549	1.372404	2.392903
C	4.513113	0.062580	0.918352
H	4.498797	0.220612	1.996486
H	6.640537	-0.194616	0.810451
H	-3.199169	-0.297463	-2.507475
C	-1.102686	-3.255104	-2.405011
H	-0.652790	-3.935580	-1.668651
H	-1.163391	-3.748418	-3.380589

H	-0.487545	-2.345347	-2.471998
C	-2.570807	-2.252887	-0.873087
O	-2.432759	-2.923167	-2.040114
O	-1.600787	-1.990488	-0.182127
C	-5.099156	-2.580569	-1.270091
C	-4.318190	-1.418568	0.713543
C	-6.330633	-2.182508	-0.424495
H	-5.233270	-2.331671	-2.333056
C	-5.778298	-1.794502	0.950187
H	-7.068560	-2.993732	-0.370862
H	-5.770636	-2.650924	1.645883
C	-3.939310	-1.847451	-0.625096
O	-3.619287	-0.855491	1.548998
H	-4.930305	-3.670564	-1.223675
H	-6.830583	-1.318136	-0.879464
H	-6.317198	-0.976335	1.447839
C	0.802550	3.338096	-1.999883
H	0.686159	4.307640	-2.504541
H	0.110968	3.296889	-1.150023
H	0.539553	2.544754	-2.713372
C	2.211644	3.170244	-1.463427
O	2.410024	3.043826	-0.255985
N	3.236889	3.159919	-2.349379
C	4.599579	3.047770	-1.861652
H	4.600133	2.571484	-0.874667
H	5.184978	2.433832	-2.559515
H	5.075072	4.039589	-1.785352
C	3.091642	3.284066	-3.785115
H	3.420903	2.358349	-4.284575
H	2.053361	3.479553	-4.065107
H	3.710894	4.114112	-4.157742
C	4.846502	-3.166167	2.146436
H	4.609428	-2.838982	3.163785
H	5.697359	-2.583821	1.768583
H	5.139792	-4.225200	2.162985
C	3.609715	-2.960109	1.295662
O	2.583354	-2.499638	1.787617

N 3.684890 -3.307302 -0.015929
C 2.487693 -3.288955 -0.832711
H 1.716995 -2.680572 -0.346014
H 2.717771 -2.864143 -1.820700
H 2.097564 -4.310649 -0.977346
C 4.847145 -3.914599 -0.632461
H 5.756319 -3.702058 -0.062503
H 4.730672 -5.007445 -0.724380
H 4.977528 -3.495318 -1.640755

PCMopt_microsolv/9_TS_SRa-mono_solv

Frequencies, energies and thermodynamic properties:

Lowest Vibrational Mode (1/cm) = -253.7547
2nd Lowest Vibrational Mode (1/cm) = 18.9387
E(RM062X) (a.u.) = -2555.24668579
Thermal correction to Enthalpy (a.u.) = 0.748835
Thermal correction to Gibbs Free Energy (a.u.) = 0.625672
Total Entropy (cal/Kmol) = 259.218
Esp(RM062X+D3) (a.u.) = -2557.82359352
Esp(D3) (a.u.) = -0.0115510859
Esp(RM062X) (a.u.) = -2557.8120424341
Esp(RM062X+D3) SMD (a.u.) = -2557.84079666
Esp(RM062X) SMD (a.u.) = -2557.8292455741

Optimised cartesian coordinates (Angstrom):

C -5.933502 0.057697 -2.090858
C -5.999186 -1.327445 -1.886687
C -4.893931 -2.030572 -1.415599
C -3.725863 -1.316401 -1.156336
C -3.656205 0.059934 -1.380872
H -6.927976 -1.860359 -2.091280
H -4.940398 -3.106694 -1.244262
C -1.542506 -0.322719 -0.563852
C 0.229558 1.372446 -0.361230
C -0.507059 2.002071 -1.553677
C -2.009301 1.960274 -1.322569
H -2.552944 2.300052 -2.213623
S -2.202454 -1.941575 -0.557391

N	-2.392153	0.571143	-1.080872
N	-0.327466	0.013830	-0.115178
C	0.015337	3.405439	-1.933526
C	-0.685214	4.559481	-1.210089
C	-0.078107	3.604465	-3.447665
H	1.083176	3.425004	-1.659307
H	-1.730983	4.654379	-1.542751
H	-0.680157	4.444104	-0.117456
H	-0.181388	5.505983	-1.452398
H	0.508582	2.847513	-3.987026
H	-1.122890	3.533908	-3.789986
H	0.297651	4.597613	-3.731827
H	-0.292364	1.330095	-2.401328
H	1.263022	1.196462	-0.692580
C	0.252565	2.230651	0.884362
C	1.395980	3.001022	1.132965
C	-0.841417	2.320393	1.750947
C	1.426619	3.885521	2.210440
H	2.266980	2.881372	0.482310
C	-0.803532	3.202741	2.833083
H	-1.728063	1.697555	1.597772
C	0.322656	3.994874	3.058260
H	2.319040	4.485812	2.394554
H	-1.661882	3.268627	3.503926
H	0.346644	4.686010	3.902260
C	1.799224	-0.576881	0.929247
H	1.937185	0.455623	1.235630
C	2.894366	-1.411631	0.877929
C	4.003416	-1.124973	1.850379
F	3.698646	-1.638635	3.051834
F	5.185211	-1.648451	1.513602
F	4.194348	0.186489	2.031684
O	0.205860	-2.184246	0.238872
C	0.565928	-1.019072	0.354191
H	-2.320548	2.576228	-0.465223
C	-4.763165	0.771177	-1.845029
H	-4.722095	1.848579	-2.001106

H	-6.813857	0.591482	-2.449636
H	2.764466	-2.475178	0.659355
C	1.387060	-3.139945	-2.765434
H	1.186807	-2.573138	-3.684936
H	1.508321	-4.202673	-2.999040
H	0.550601	-2.993967	-2.066857
C	2.662019	-1.419340	-1.804758
O	2.603143	-2.720419	-2.166996
O	1.725085	-0.667637	-2.011190
C	5.118456	-1.960619	-1.267991
C	4.255551	0.292979	-0.887503
C	6.329112	-1.008377	-1.060943
H	5.135339	-2.800115	-0.556054
C	5.766514	0.369748	-0.676758
H	6.908828	-0.936592	-1.991182
H	6.167667	1.196859	-1.280327
C	3.888597	-1.094573	-1.105097
O	3.509478	1.264345	-0.816880
H	5.137556	-2.413400	-2.270747
H	7.008493	-1.393194	-0.290112
H	5.950461	0.629459	0.377074
C	-1.999724	-1.146824	2.817765
H	-1.282981	-0.327605	2.685032
H	-1.604020	-2.049578	2.331399
H	-2.107503	-1.353426	3.891800
C	-3.320337	-0.714326	2.209464
O	-3.434748	0.393679	1.687018
N	-4.360226	-1.581835	2.251766
C	-5.655851	-1.163767	1.748331
H	-5.523589	-0.351482	1.025157
H	-6.145158	-2.013695	1.253450
H	-6.304679	-0.816626	2.569552
C	-4.319020	-2.894048	2.863787
H	-3.317279	-3.129844	3.231763
H	-5.022689	-2.947885	3.709322
H	-4.607806	-3.660817	2.128206

PCMopt_microsolv/9_TS_SSa-bi_solv

Frequencies, energies and thermodynamic properties:

Lowest Vibrational Mode (1/cm) =	-220.2911
2nd Lowest Vibrational Mode (1/cm) =	14.4837
E(RM062X) (a.u.) =	-2842.74486984
Thermal correction to Enthalpy (a.u.) =	0.889882
Thermal correction to Gibbs Free Energy (a.u.) =	0.743836
Total Entropy (cal/Kmol) =	307.378
Esp(RM062X+D3) (a.u.) =	-2845.65681971
Esp(D3) (a.u.) =	-0.0151723652
Esp(RM062X) (a.u.) =	-2845.6416473448
Esp(RM062X+D3) SMD (a.u.) =	-2845.67543947
Esp(RM062X) SMD (a.u.) =	-2845.6602671048

Optimised cartesian coordinates (Angstrom):

C	-5.676620	0.359887	0.279222
C	-5.680412	0.847179	-1.035852
C	-4.506484	0.884967	-1.782152
C	-3.333037	0.436057	-1.178107
C	-3.325657	-0.014263	0.142714
H	-6.613796	1.191237	-1.482079
H	-4.501827	1.249135	-2.810557
C	-1.101429	-0.241947	-0.371396
C	0.674416	-0.833894	1.226860
C	-0.325664	-0.272445	2.248620
C	-1.730476	-0.755251	1.934930
H	-2.450556	-0.252310	2.589986
S	-1.729378	0.381097	-1.878619
N	-2.042148	-0.349389	0.568017
N	0.181354	-0.566515	-0.149977
C	0.084222	-0.509555	3.718220
C	-0.434689	-1.821296	4.314997
C	-0.369391	0.673689	4.575431
H	1.186542	-0.538080	3.743159
H	-1.530714	-1.796310	4.420794
H	-0.167363	-2.699946	3.711563
H	-0.014801	-1.961685	5.321292
H	0.101526	1.608646	4.240445

H	-1.458836	0.811838	4.503567
H	-0.108819	0.511175	5.631173
H	-0.341222	0.815293	2.076899
H	1.577550	-0.214177	1.320961
C	1.025407	-2.293368	1.419053
C	2.194931	-2.624645	2.112028
C	0.182344	-3.316963	0.972553
C	2.503619	-3.955747	2.388734
H	2.873119	-1.830735	2.434726
C	0.495185	-4.649994	1.245634
H	-0.725314	-3.084490	0.407080
C	1.649031	-4.972658	1.960850
H	3.417029	-4.199099	2.933314
H	-0.170233	-5.440830	0.895229
H	1.888803	-6.015394	2.174208
C	2.523231	-0.611603	-0.889607
H	2.819927	-1.174357	-0.010351
C	3.474946	0.053083	-1.619075
C	4.887029	-0.453409	-1.563811
F	5.025047	-1.519627	-2.368625
F	5.796245	0.434989	-1.964828
F	5.233407	-0.857277	-0.335850
O	0.738755	0.079333	-2.269359
C	1.145409	-0.337183	-1.192675
H	-1.841806	-1.847299	2.017779
C	-4.503790	-0.078125	0.887531
H	-4.509380	-0.449219	1.912096
H	-6.610935	0.322449	0.840939
H	3.209738	0.520898	-2.570312
C	4.627421	0.621946	2.502358
H	5.160559	1.460487	2.969624
H	3.912723	0.194789	3.214811
H	5.363223	-0.137839	2.202396
C	4.567078	1.732570	0.442711
O	3.873204	1.055256	1.385137
O	5.756590	1.953129	0.578989
C	4.377354	3.016322	-1.772390

C	2.344198	2.377350	-0.634626
C	3.278964	4.052504	-2.086041
H	5.316661	3.474316	-1.432570
C	1.972791	3.318471	-1.781188
H	3.335898	4.436835	-3.113473
H	1.648744	2.689755	-2.629354
C	3.767083	2.130410	-0.705735
O	1.519443	1.876413	0.127719
H	4.621502	2.438817	-2.681302
H	3.379301	4.909702	-1.402361
H	1.127340	3.962684	-1.502680
C	-0.987387	-2.933473	-2.650255
H	-0.948651	-3.787944	-3.340558
H	-0.268661	-3.100786	-1.839376
H	-0.695652	-2.029313	-3.202163
C	-2.365478	-2.796164	-2.031498
O	-2.516180	-2.892976	-0.814090
N	-3.416413	-2.561850	-2.853878
C	-4.754131	-2.479049	-2.296050
H	-4.697178	-2.190962	-1.240229
H	-5.329478	-1.722248	-2.846298
H	-5.278072	-3.445586	-2.379191
C	-3.326827	-2.424581	-4.292933
H	-3.653354	-1.417909	-4.599865
H	-2.303739	-2.579518	-4.644624
H	-3.977973	-3.161883	-4.787043
C	-4.639613	2.833474	2.756981
H	-4.880783	3.883057	2.975718
H	-4.430301	2.305838	3.692969
H	-5.513925	2.377108	2.273427
C	-3.406088	2.729488	1.882725
O	-2.417142	2.113414	2.270116
N	-3.443118	3.342471	0.670978
C	-2.240975	3.418373	-0.135931
H	-1.505277	2.687065	0.217136
H	-2.486300	3.209709	-1.187485
H	-1.795338	4.425863	-0.078781

C -4.561880 4.129829 0.192832
H -5.491641 3.845445 0.694447
H -4.390564 5.209098 0.342094
H -4.692819 3.946875 -0.883574

PCMopt_microsolv/9_TS_SSa-mono_solv
Frequencies, energies and thermodynamic properties:
Lowest Vibrational Mode (1/cm) = -200.7281
2nd Lowest Vibrational Mode (1/cm) = 16.2944
E(RM062X) (a.u.) = -2555.24239667
Thermal correction to Enthalpy (a.u.) = 0.748426
Thermal correction to Gibbs Free Energy (a.u.) = 0.623960
Total Entropy (cal/Kmol) = 261.962
Esp(RM062X+D3) (a.u.) = -2557.82352157
Esp(D3) (a.u.) = -0.0115924336
Esp(RM062X) (a.u.) = -2557.8119291364
Esp(RM062X+D3) SMD (a.u.) = -2557.84206056
Esp(RM062X) SMD (a.u.) = -2557.8304681264
Optimised cartesian coordinates (Angstrom):
C -5.904449 -0.062537 -2.209627
C -5.940652 -1.449395 -2.008032
C -4.831034 -2.125482 -1.509329
C -3.687326 -1.382643 -1.221862
C -3.646441 -0.005035 -1.443887
H -6.850547 -2.004806 -2.236318
H -4.855327 -3.202504 -1.339102
C -1.542535 -0.338617 -0.581803
C 0.193233 1.394192 -0.359807
C -0.548629 2.018599 -1.552595
C -2.050879 1.935221 -1.335695
H -2.592313 2.272856 -2.228561
S -2.167233 -1.969463 -0.578143
N -2.401952 0.535328 -1.114068
N -0.337407 0.026140 -0.120725
C -0.065530 3.441497 -1.912218
C -0.803829 4.565411 -1.178681
C -0.157280 3.655542 -3.424387

H	1.000102	3.492977	-1.633245
H	-1.849036	4.635731	-1.518581
H	-0.804311	4.436232	-0.087606
H	-0.324696	5.528520	-1.404547
H	0.454406	2.923192	-3.969707
H	-1.197773	3.558035	-3.772747
H	0.189697	4.662978	-3.694048
H	-0.308070	1.361489	-2.404804
H	1.227478	1.231160	-0.695352
C	0.186177	2.237455	0.896948
C	1.293736	3.047333	1.172149
C	-0.917023	2.272060	1.756005
C	1.287620	3.909363	2.267494
H	2.171271	2.997622	0.523333
C	-0.919448	3.131967	2.856275
H	-1.782476	1.625569	1.581716
C	0.175486	3.958552	3.109427
H	2.156192	4.538305	2.467589
H	-1.785881	3.152597	3.519445
H	0.168449	4.630878	3.968588
C	1.812448	-0.533124	0.922189
H	2.001475	0.514690	1.133958
C	2.843449	-1.430789	0.979773
C	4.026060	-1.101273	1.842899
F	3.744228	-1.367900	3.128890
F	5.120159	-1.803181	1.549322
F	4.350892	0.195496	1.786999
O	0.191212	-2.149199	0.336129
C	0.557815	-0.984037	0.381916
H	-2.386618	2.532404	-0.474286
C	-4.759130	0.679680	-1.934308
H	-4.742161	1.758025	-2.088416
H	-6.788695	0.449453	-2.589900
H	2.649586	-2.501503	0.879956
C	4.564928	2.111594	-0.839718
H	5.402487	2.182286	-1.546033
H	3.843297	2.911353	-1.041395

H	4.958915	2.207234	0.182421
C	4.632921	-0.225905	-0.954108
O	3.867625	0.889233	-0.993770
O	5.838838	-0.151679	-0.801759
C	4.615969	-2.781025	-1.210443
C	2.611874	-1.554701	-1.772983
C	3.844786	-3.552853	-2.300773
H	5.677588	-2.637044	-1.455432
C	2.419006	-3.008163	-2.209713
H	3.906320	-4.642507	-2.176445
H	1.836856	-3.514097	-1.419217
C	3.881531	-1.462076	-1.094992
O	1.755007	-0.683272	-1.917125
H	4.588132	-3.340951	-0.259040
H	4.263549	-3.305196	-3.288516
H	1.835084	-3.064720	-3.138560
C	-2.096857	-1.194225	2.803282
H	-2.240772	-1.391557	3.874869
H	-1.373864	-0.377725	2.688390
H	-1.686833	-2.100768	2.337067
C	-3.395980	-0.763113	2.149931
O	-3.493814	0.345588	1.624787
N	-4.435637	-1.631233	2.155959
C	-5.713456	-1.213744	1.608052
H	-5.556472	-0.410308	0.879772
H	-6.190461	-2.067736	1.108459
H	-6.385718	-0.854454	2.404788
C	-4.414388	-2.943950	2.768249
H	-4.665051	-3.712076	2.020153
H	-3.429237	-3.173822	3.181875
H	-5.155366	-3.001831	3.580856

SMDopt/16_TS_SRa

Frequencies, energies and thermodynamic properties:

Lowest Vibrational Mode (1/cm) = -156.9758

2nd Lowest Vibrational Mode (1/cm) = 17.3388

E(RM062X) (a.u.) = -2420.02976858

Thermal correction to Enthalpy (a.u.) = 0.632386

Thermal correction to Gibbs Free Energy (a.u.) = 0.522495

Total Entropy (cal/Kmol) = 231.286

Esp(RM062X+D3) (a.u.) = -2422.44726399

Esp(D3) (a.u.) = -0.0095321619

Esp(RM062X) (a.u.) = -2422.4377318281

Esp(RM062X+D3) PCM (a.u.) = -2422.42738533

Esp(RM062X) PCM (a.u.) = -2422.4178531681

Esp(RM062X+D3) GAS (a.u.) = -2422.37687640

Esp(RM062X) GAS (a.u.) = -2422.3673442381

Optimised cartesian coordinates (Angstrom):

C	6.440800	-2.226353	-0.671249
C	5.933964	-3.270014	-1.460395
C	4.582272	-3.331212	-1.781416
C	3.749448	-2.325622	-1.289190
C	4.254899	-1.294353	-0.496223
H	6.608380	-4.042580	-1.831663
H	4.183986	-4.137201	-2.399380
C	2.038780	-0.689203	-0.585915
C	1.082287	1.301091	0.501422
C	2.256912	1.133967	1.478783
C	3.523492	0.769262	0.730961
H	4.317406	0.491702	1.435274
S	2.027504	-2.138604	-1.555051
N	3.252381	-0.398852	-0.110868
N	0.959743	0.082693	-0.339018
C	2.447172	2.331508	2.436706
C	3.382743	3.422387	1.908770
C	2.947392	1.828894	3.791426
H	1.450256	2.775984	2.591496
H	4.424761	3.066130	1.882367
H	3.115121	3.768713	0.900327
H	3.352706	4.291844	2.581743
H	2.231276	1.130850	4.249923
H	3.910940	1.305697	3.681843
H	3.099401	2.668178	4.485874
H	1.991246	0.261503	2.094209

H	0.177377	1.328980	1.122565
C	1.141416	2.553512	-0.349799
C	0.457677	3.697682	0.077624
C	1.905749	2.612360	-1.519948
C	0.564060	4.891809	-0.633298
H	-0.161675	3.649842	0.977035
C	2.011321	3.807766	-2.232274
H	2.426067	1.725915	-1.892086
C	1.347990	4.951109	-1.786657
H	0.028056	5.777684	-0.288406
H	2.613474	3.842617	-3.141733
H	1.431684	5.885052	-2.345027
C	-1.414485	0.644494	-0.732946
H	-1.291638	1.614681	-0.258245
C	-2.647527	0.236309	-1.136467
C	-3.728233	1.273340	-1.232804
F	-3.450527	2.141343	-2.223411
F	-4.925024	0.761620	-1.519492
F	-3.847322	2.002724	-0.121488
O	-0.338233	-1.236980	-1.663703
C	-0.288517	-0.246578	-0.962002
H	3.891382	1.582746	0.087746
C	5.613229	-1.223572	-0.178394
H	6.015114	-0.411061	0.426049
H	7.506134	-2.195910	-0.439354
H	-2.793268	-0.667213	-1.731394
C	-7.089827	-1.264249	-0.407094
H	-7.239868	-0.220922	-0.721681
H	-7.451293	-1.941910	-1.189764
H	-7.663124	-1.430904	0.517045
C	-5.076912	-0.772897	0.672066
O	-5.719384	-1.550078	-0.226697
O	-5.680085	0.059975	1.328162
C	-2.966661	-2.212629	0.297981
O	-3.281736	-3.054669	-0.537602
C	-3.658006	-1.028351	0.770571
C	-1.600465	-1.153048	1.931910

C	-0.462898	-0.967303	2.721618
C	-1.661940	-2.227514	1.042141
C	0.593283	-1.874105	2.601689
H	-0.399353	-0.133581	3.426295
C	-0.606195	-3.128483	0.908096
C	0.527827	-2.945660	1.697091
H	1.483988	-1.751090	3.222364
H	-0.677752	-3.946444	0.187733
H	1.371612	-3.634582	1.616208
C	-2.875920	-0.351708	1.866660
H	-2.658399	0.710372	1.650946
H	-3.408328	-0.367285	2.833294

SMDopt/16_TS_SRb

Frequencies, energies and thermodynamic properties:

Lowest Vibrational Mode (1/cm) = -166.4626

2nd Lowest Vibrational Mode (1/cm) = 22.0284

E(RM062X) (a.u.) = -2420.02965697

Thermal correction to Enthalpy (a.u.) = 0.632335

Thermal correction to Gibbs Free Energy (a.u.) = 0.522484

Total Entropy (cal/Kmol) = 231.200

Esp(RM062X+D3) (a.u.) = -2422.44643832

Esp(D3) (a.u.) = -0.0095568599

Esp(RM062X) (a.u.) = -2422.4368814601

Optimised cartesian coordinates (Angstrom):

C	6.537601	-1.979741	-0.550844
C	6.095921	-3.038400	-1.358738
C	4.756289	-3.155213	-1.712785
C	3.868940	-2.190671	-1.234062
C	4.309348	-1.144771	-0.421712
H	6.811971	-3.778036	-1.718890
H	4.408143	-3.972459	-2.345975
C	2.071625	-0.635919	-0.562895
C	1.003858	1.305948	0.506684
C	2.158089	1.185402	1.514285
C	3.458480	0.875550	0.800888
H	4.241397	0.626204	1.527611

S	2.147855	-2.076061	-1.542922
N	3.259213	-0.296956	-0.054858
N	0.953687	0.085393	-0.337876
C	2.271965	2.389008	2.476849
C	3.171860	3.520927	1.973306
C	2.758111	1.908470	3.844630
H	1.252711	2.788969	2.604930
H	4.229397	3.212593	1.976616
H	2.917185	3.854786	0.957318
H	3.083632	4.388122	2.644077
H	2.061490	1.179806	4.285119
H	3.746449	1.428399	3.761205
H	2.854639	2.753676	4.541822
H	1.912041	0.301376	2.121437
H	0.083083	1.296627	1.104384
C	1.034970	2.559158	-0.344472
C	0.292363	3.674355	0.059593
C	1.829985	2.647500	-1.492144
C	0.368191	4.870038	-0.652767
H	-0.349616	3.602757	0.941370
C	1.905306	3.844258	-2.205816
H	2.398413	1.782850	-1.844644
C	1.181037	4.959383	-1.783922
H	-0.214262	5.733393	-0.326586
H	2.531257	3.902238	-3.097859
H	1.240468	5.894337	-2.343698
C	-1.431628	0.546905	-0.787532
H	-1.368428	1.506193	-0.280358
C	-2.632697	0.099768	-1.243534
C	-3.747849	1.100836	-1.338210
F	-3.489430	1.985138	-2.319805
F	-4.927218	0.557852	-1.636333
F	-3.896584	1.817343	-0.221633
O	-0.249343	-1.270905	-1.715029
C	-0.261736	-0.289272	-0.999611
H	3.811646	1.707234	0.172892
C	5.655594	-1.017697	-0.071463

H	6.009086	-0.193580	0.547247
H	7.594795	-1.904414	-0.293254
H	-2.721995	-0.785540	-1.875760
C	-6.986755	0.003710	0.783534
H	-7.589293	-0.870774	1.070897
H	-7.282160	0.867806	1.390709
H	-7.173487	0.217284	-0.279213
C	-5.042352	-1.231635	0.321707
O	-5.613154	-0.211248	1.024708
O	-5.715507	-1.940661	-0.398305
C	-2.816316	-2.428612	0.126768
O	-3.030677	-3.276219	-0.734244
C	-3.621124	-1.308028	0.569798
C	-1.624273	-1.289848	1.837806
C	-0.549439	-1.029767	2.692437
C	-1.556655	-2.357623	0.940182
C	0.574057	-1.857733	2.629658
H	-0.586958	-0.200748	3.404399
C	-0.432383	-3.179283	0.863060
C	0.637957	-2.923391	1.717771
H	1.416097	-1.677109	3.301918
H	-0.403780	-3.995016	0.137153
H	1.532078	-3.549981	1.683482
C	-2.952414	-0.584587	1.713443
H	-3.524150	-0.657448	2.654450
H	-2.804849	0.493472	1.524637

SMDopt/16_TS_SRc

Frequencies, energies and thermodynamic properties:

Lowest Vibrational Mode (1/cm) = -166.4605

2nd Lowest Vibrational Mode (1/cm) = 22.0288

E(RM062X) (a.u.) = -2420.02965697

Thermal correction to Enthalpy (a.u.) = 0.632335

Thermal correction to Gibbs Free Energy (a.u.) = 0.522485

Total Entropy (cal/Kmol) = 231.199

Esp(RM062X+D3) (a.u.) = -2422.44643829

Esp(D3) (a.u.) = -0.0095568523

Esp(RM062X) (a.u.) = -2422.4368814377

Optimised cartesian coordinates (Angstrom):

C	6.537590	-1.979768	-0.550852
C	6.095902	-3.038429	-1.358737
C	4.756268	-3.155238	-1.712779
C	3.868926	-2.190688	-1.234059
C	4.309343	-1.144785	-0.421716
H	6.811946	-3.778072	-1.718888
H	4.408116	-3.972485	-2.345964
C	2.071622	-0.635923	-0.562895
C	1.003867	1.305952	0.506683
C	2.158102	1.185404	1.514279
C	3.458489	0.875546	0.800877
H	4.241408	0.626201	1.527597
S	2.147841	-2.076071	-1.542915
N	3.259213	-0.296963	-0.054863
N	0.953688	0.085396	-0.337874
C	2.271986	2.389012	2.476840
C	3.171888	3.520924	1.973292
C	2.758134	1.908475	3.844622
H	1.252736	2.788980	2.604922
H	4.229421	3.212579	1.976585
H	2.917201	3.854793	0.957310
H	3.083679	4.388116	2.644070
H	2.061517	1.179806	4.285108
H	3.746475	1.428411	3.761197
H	2.854654	2.753681	4.541815
H	1.912054	0.301379	2.121434
H	0.083094	1.296637	1.104387
C	1.034981	2.559160	-0.344475
C	0.292380	3.674362	0.059589
C	1.829992	2.647496	-1.492151
C	0.368211	4.870043	-0.652773
H	-0.349595	3.602768	0.941369
C	1.905315	3.844252	-2.205827
H	2.398414	1.782842	-1.844652
C	1.181052	4.959381	-1.783932

H	-0.214236	5.733401	-0.326592
H	2.531262	3.902226	-3.097872
H	1.240485	5.894333	-2.343711
C	-1.431629	0.546915	-0.787522
H	-1.368427	1.506200	-0.280344
C	-2.632697	0.099780	-1.243526
C	-3.747851	1.100846	-1.338195
F	-3.489421	1.985173	-2.319764
F	-4.927213	0.557863	-1.636348
F	-3.896607	1.817325	-0.221603
O	-0.249353	-1.270900	-1.715019
C	-0.261738	-0.289266	-0.999603
H	3.811653	1.707227	0.172875
C	5.655591	-1.017717	-0.071473
H	6.009089	-0.193598	0.547231
H	7.594786	-1.904445	-0.293266
H	-2.721995	-0.785527	-1.875754
C	-6.986768	0.003697	0.783512
H	-7.589304	-0.870796	1.070854
H	-7.282184	0.867781	1.390700
H	-7.173488	0.217288	-0.279232
C	-5.042356	-1.231638	0.321696
O	-5.613168	-0.211258	1.024698
O	-5.715502	-1.940662	-0.398326
C	-2.816315	-2.428607	0.126771
O	-3.030669	-3.276216	-0.734242
C	-3.621130	-1.308028	0.569798
C	-1.624282	-1.289838	1.837812
C	-0.549449	-1.029749	2.692442
C	-1.556657	-2.357614	0.940189
C	0.574051	-1.857709	2.629666
H	-0.586973	-0.200728	3.404402
C	-0.432382	-3.179269	0.863070
C	0.637957	-2.923370	1.717781
H	1.416091	-1.677078	3.301923
H	-0.403774	-3.995003	0.137165
H	1.532081	-3.549955	1.683494

C -2.952426 -0.584585 1.713447
H -2.804868 0.493476 1.524643
H -3.524164 -0.657453 2.654452

SMDopt/16_TS_SRd
Frequencies, energies and thermodynamic properties:
Lowest Vibrational Mode (1/cm) = -272.9820
2nd Lowest Vibrational Mode (1/cm) = 18.4517
E(RM062X) (a.u.) = -2420.03797032
Thermal correction to Enthalpy (a.u.) = 0.632980
Thermal correction to Gibbs Free Energy (a.u.) = 0.524677
Total Entropy (cal/Kmol) = 227.943
Esp(RM062X+D3) (a.u.) = -2422.44741675
Esp(D3) (a.u.) = -0.0090726637
Esp(RM062X) (a.u.) = -2422.4383440863
Optimised cartesian coordinates (Angstrom):
C 7.245120 -0.831769 0.513968
C 7.225472 -2.035131 -0.204597
C 6.035141 -2.539180 -0.721766
C 4.867989 -1.808655 -0.504983
C 4.889667 -0.614594 0.218912
H 8.154128 -2.584241 -0.364821
H 6.015328 -3.474354 -1.283095
C 2.654496 -0.719770 -0.315151
C 1.010473 1.005604 0.238909
C 1.885746 1.195287 1.487932
C 3.356710 1.162450 1.113345
H 3.987323 1.108620 2.010759
S 3.237339 -2.210796 -1.019334
N 3.617289 -0.045446 0.325087
N 1.405992 -0.260563 -0.425614
C 1.503091 2.434385 2.327560
C 2.234194 3.719628 1.931584
C 1.726956 2.145816 3.812195
H 0.422112 2.591356 2.175752
H 3.300408 3.664762 2.203434
H 2.167260 3.938102 0.856135

H	1.802919	4.573119	2.475239
H	1.118905	1.293731	4.149702
H	2.785153	1.909638	4.009433
H	1.460192	3.020511	4.423700
H	1.681243	0.303947	2.103118
H	-0.013008	0.835185	0.601070
C	1.009593	2.174775	-0.721983
C	-0.049350	3.088742	-0.651326
C	2.044074	2.396147	-1.637768
C	-0.050964	4.229358	-1.453781
H	-0.879947	2.882854	0.029749
C	2.039465	3.535714	-2.443331
H	2.863282	1.678878	-1.736046
C	0.998166	4.459589	-2.345417
H	-0.879202	4.937431	-1.388925
H	2.852854	3.700421	-3.152228
H	0.997231	5.351327	-2.974829
C	-0.857702	-0.438279	-1.319297
H	-0.931701	0.646361	-1.339845
C	-2.010280	-1.195259	-1.354601
C	-3.173025	-0.631516	-2.118494
F	-2.954378	-0.756449	-3.438880
F	-4.332773	-1.249539	-1.880025
F	-3.362513	0.671385	-1.889124
O	0.652903	-2.262154	-1.238745
C	0.381835	-1.090644	-1.021011
H	3.661825	2.039743	0.523767
C	6.080112	-0.103649	0.737522
H	6.100276	0.835551	1.290030
H	8.190414	-0.453537	0.905217
H	-1.940635	-2.284747	-1.423058
C	-0.435020	-3.883397	1.601635
H	-0.094920	-3.594454	2.606186
H	-0.652524	-4.957111	1.578751
H	0.352527	-3.640175	0.873144
C	-1.574406	-1.874891	1.203869
O	-1.643032	-3.221408	1.261108

O -0.548398 -1.280508 1.477465
C -3.036157 0.155394 0.888979
O -2.216144 1.064839 0.958744
C -2.802945 -1.276278 0.715988
C -5.144783 -0.919006 0.867387
C -6.536882 -0.997251 0.862981
C -4.519966 0.330850 0.912231
C -7.276520 0.187337 0.912007
H -7.043698 -1.963936 0.821696
C -5.250072 1.517677 0.958818
C -6.641797 1.436738 0.959792
H -8.367455 0.140664 0.911256
H -4.734188 2.479846 0.989734
H -7.244268 2.346351 0.994216
C -4.118845 -2.025806 0.818721
H -4.305904 -2.717160 -0.017180
H -4.159497 -2.631107 1.739693

SMDopt/16_TS_SRe

Frequencies, energies and thermodynamic properties:

Lowest Vibrational Mode (1/cm) = -251.2712
2nd Lowest Vibrational Mode (1/cm) = 19.9082
E(RM062X) (a.u.) = -2420.03559700
Thermal correction to Enthalpy (a.u.) = 0.632837
Thermal correction to Gibbs Free Energy (a.u.) = 0.524894
Total Entropy (cal/Kmol) = 227.186
Esp(RM062X+D3) (a.u.) = -2422.44678286
Esp(D3) (a.u.) = -0.0092413852
Esp(RM062X) (a.u.) = -2422.4375414748

Optimised cartesian coordinates (Angstrom):

C -7.239048 -0.791369 -0.459457
C -7.212257 -2.027521 0.201583
C -6.016191 -2.558433 0.675692
C -4.849801 -1.822279 0.472267
C -4.878578 -0.595990 -0.194656
H -8.140349 -2.580604 0.350914
H -5.990650 -3.518245 1.193473

C	-2.635861	-0.735280	0.295530
C	-0.976247	0.989226	-0.242533
C	-1.904454	1.274613	-1.435463
C	-3.358285	1.232909	-1.000689
H	-4.024404	1.254481	-1.872938
S	-3.215061	-2.248636	0.949662
N	-3.605133	-0.026907	-0.293653
N	-1.374623	-0.295295	0.385839
C	-1.542018	2.564773	-2.206246
C	-2.243644	3.826610	-1.696970
C	-1.829662	2.377032	-3.696137
H	-0.454547	2.704264	-2.089831
H	-3.320805	3.796988	-1.926098
H	-2.128980	3.976058	-0.613880
H	-1.827212	4.709704	-2.203570
H	-1.242564	1.547037	-4.115739
H	-2.896778	2.159511	-3.864193
H	-1.581879	3.289662	-4.258012
H	-1.751671	0.430777	-2.127323
H	0.027901	0.809152	-0.654819
C	-0.900446	2.112873	0.769671
C	0.166500	3.015588	0.681047
C	-1.879376	2.304045	1.750361
C	0.228518	4.118987	1.531142
H	0.955884	2.830596	-0.053001
C	-1.814826	3.407060	2.602985
H	-2.701729	1.592367	1.863335
C	-0.767779	4.322111	2.487858
H	1.062263	4.819009	1.451999
H	-2.585763	3.549503	3.362313
H	-0.720130	5.185230	3.154214
C	0.892679	-0.496452	1.278898
H	0.990531	0.585241	1.279916
C	2.025146	-1.275101	1.353430
C	3.191972	-0.690170	2.096167
F	2.965504	-0.753890	3.419873
F	4.347466	-1.326078	1.892448

F	3.391197	0.600286	1.809651
O	-0.652288	-2.290464	1.229809
C	-0.363075	-1.127252	0.996606
H	-3.625773	2.066964	-0.335074
C	-6.075711	-0.056794	-0.667993
H	-6.102841	0.908167	-1.173588
H	-8.189303	-0.392384	-0.816531
H	1.942668	-2.360808	1.452991
C	-0.479545	-2.376859	-1.933450
H	-0.836323	-3.021482	-1.116731
H	-1.269007	-1.678245	-2.236096
H	-0.200265	-3.008227	-2.787990
C	1.738867	-2.283391	-1.198965
O	0.621473	-1.590616	-1.503346
O	1.781293	-3.493413	-1.307253
C	2.985033	-0.034189	-0.975041
O	2.111693	0.822696	-1.093168
C	2.853213	-1.464348	-0.737143
C	5.167966	-0.949787	-0.872905
C	6.561594	-0.925930	-0.841411
C	4.454301	0.246854	-0.984728
C	7.214817	0.306076	-0.934114
H	7.135800	-1.850362	-0.747006
C	5.097672	1.480305	-1.075915
C	6.491729	1.501538	-1.051210
H	8.306061	0.339559	-0.913947
H	4.514750	2.400165	-1.159780
H	7.026941	2.450517	-1.119654
C	4.220513	-2.120173	-0.794665
H	4.442385	-2.772028	0.063713
H	4.307890	-2.753157	-1.694310

SMDopt/16_TS_SRf

Frequencies, energies and thermodynamic properties:

Lowest Vibrational Mode (1/cm) = -212.9017

2nd Lowest Vibrational Mode (1/cm) = 15.9627

E(RM062X) (a.u.) = -2420.02555196

Thermal correction to Enthalpy (a.u.) = 0.632525

Thermal correction to Gibbs Free Energy (a.u.) = 0.522247

Total Entropy (cal/Kmol) = 232.099

Esp(RM062X+D3) (a.u.) = -2422.44006488

Esp(D3) (a.u.) = -0.0091689228

Esp(RM062X) (a.u.) = -2422.4308959572

Optimised cartesian coordinates (Angstrom):

C	-6.803302	2.176006	0.077900
C	-6.484635	3.220132	-0.802413
C	-5.198594	3.354258	-1.316544
C	-4.241592	2.416892	-0.929362
C	-4.559042	1.384501	-0.044728
H	-7.254495	3.936356	-1.091878
H	-4.946064	4.163849	-2.002716
C	-2.349430	0.900507	-0.453513
C	-1.160780	-1.046564	0.447956
C	-2.103671	-0.855424	1.647722
C	-3.510575	-0.548486	1.168304
H	-4.143874	-0.229531	2.006422
S	-2.554826	2.348280	-1.407070
N	-3.456052	0.564665	0.215289
N	-1.227409	0.165167	-0.408247
C	-2.053430	-2.018120	2.664949
C	-3.045115	-3.150379	2.384932
C	-2.268183	-1.478910	4.079400
H	-1.032948	-2.432771	2.615330
H	-4.079870	-2.821831	2.571890
H	-2.988058	-3.528316	1.354149
H	-2.843475	-3.992621	3.062988
H	-1.493851	-0.746456	4.350589
H	-3.249771	-0.985634	4.165211
H	-2.239090	-2.296146	4.815024
H	-1.732505	0.046409	2.161900
H	-0.138062	-1.071952	0.850328
C	-1.389687	-2.317853	-0.341858
C	-0.576526	-3.423609	-0.063060
C	-2.405723	-2.436131	-1.295633

C	-0.804014	-4.643038	-0.699509
H	0.248053	-3.308806	0.646588
C	-2.630354	-3.656834	-1.933958
H	-3.030555	-1.577421	-1.554989
C	-1.837931	-4.764318	-1.629673
H	-0.166871	-5.500138	-0.473899
H	-3.427833	-3.740999	-2.674307
H	-2.016794	-5.718098	-2.129233
C	1.020218	-0.348731	-1.217962
H	0.816337	-1.409366	-1.088655
C	2.313042	0.070363	-1.383843
C	3.277670	-0.881433	-2.026713
F	3.112950	-0.848634	-3.364223
F	4.553211	-0.598930	-1.803551
F	3.066293	-2.151832	-1.658956
O	-0.044022	1.748113	-1.553377
C	-0.045592	0.615654	-1.105523
H	-3.987846	-1.408073	0.674954
C	-5.848636	1.243470	0.471028
H	-6.103734	0.429512	1.149215
H	-7.820094	2.088315	0.462757
H	2.536009	1.124427	-1.570641
C	3.974024	-3.352930	1.004071
H	3.237087	-3.731512	0.281233
H	4.951921	-3.806420	0.805523
H	3.642111	-3.619047	2.018030
C	3.047501	-1.204542	1.051318
O	4.146537	-1.955051	0.869991
O	1.977946	-1.706706	1.378893
C	4.472813	0.930493	0.621283
O	5.612771	0.536433	0.440423
C	3.210607	0.203386	0.779652
C	2.768971	2.506001	1.139480
C	2.205692	3.769026	1.310198
C	4.103383	2.384449	0.744268
C	3.004237	4.895174	1.084998
H	1.161871	3.882305	1.612003

C 4.909158 3.500051 0.530461

C 4.345546 4.765643 0.701908

H 2.576629 5.892005 1.210743

H 5.952492 3.374781 0.232072

H 4.947304 5.661014 0.534670

C 2.163201 1.137407 1.330580

H 1.974657 0.944518 2.402170

H 1.179325 1.035912 0.842785

SMDopt/16_TS_SSa

Frequencies, energies and thermodynamic properties:

Lowest Vibrational Mode (1/cm) = -232.8245

2nd Lowest Vibrational Mode (1/cm) = 16.6609

E(RM062X) (a.u.) = -2420.03524788

Thermal correction to Enthalpy (a.u.) = 0.632832

Thermal correction to Gibbs Free Energy (a.u.) = 0.523360

Total Entropy (cal/Kmol) = 230.403

Esp(RM062X+D3) (a.u.) = -2422.44809058

Esp(D3) (a.u.) = -0.0093118736

Esp(RM062X) (a.u.) = -2422.4387787064

Esp(RM062X+D3) PCM (a.u.) = -2422.42682604

Esp(RM062X) PCM (a.u.) = -2422.4175141664

Esp(RM062X+D3) GAS (a.u.) = -2422.39413334

Esp(RM062X) GAS (a.u.) = -2422.3848214664

Optimised cartesian coordinates (Angstrom):

C -6.435189 2.392292 0.089850

C -6.028600 3.453654 -0.730826

C -4.731689 3.510805 -1.232954

C -3.854119 2.481724 -0.894492

C -4.259139 1.430699 -0.069650

H -6.736304 4.244363 -0.982639

H -4.410073 4.333107 -1.873749

C -2.087275 0.797658 -0.494443

C -1.036601 -1.249285 0.346670

C -1.991680 -1.048149 1.534056

C -3.367877 -0.639095 1.041843

H -4.001461 -0.327572 1.882371

S	-2.173182	2.307915	-1.371318
N	-3.226196	0.513907	0.149097
N	-1.027113	-0.016308	-0.475377
C	-2.032519	-2.247096	2.507466
C	-3.084701	-3.305745	2.166411
C	-2.242879	-1.747845	3.937198
H	-1.038548	-2.722473	2.461166
H	-4.101387	-2.920798	2.344638
H	-3.027740	-3.648611	1.123317
H	-2.950165	-4.182998	2.816214
H	-1.431767	-1.073793	4.249086
H	-3.194740	-1.199380	4.023543
H	-2.278028	-2.591227	4.642550
H	-1.570161	-0.189781	2.082149
H	-0.028103	-1.308817	0.777107
C	-1.311195	-2.477593	-0.494951
C	-0.584243	-3.645968	-0.239105
C	-2.299413	-2.495211	-1.485428
C	-0.864708	-4.822507	-0.932315
H	0.210977	-3.628134	0.510614
C	-2.579750	-3.671985	-2.181193
H	-2.859491	-1.588843	-1.729200
C	-1.870149	-4.839952	-1.900130
H	-0.291798	-5.726871	-0.720005
H	-3.355460	-3.673805	-2.948992
H	-2.091450	-5.759453	-2.444866
C	1.256302	-0.577236	-1.176184
H	1.084795	-1.623463	-0.938296
C	2.541953	-0.125630	-1.348050
C	3.568763	-1.119112	-1.807618
F	3.441769	-1.330963	-3.129545
F	4.829806	-0.729065	-1.617847
F	3.428744	-2.313439	-1.220209
O	0.251438	1.524746	-1.575272
C	0.201062	0.396132	-1.115052
H	-3.879793	-1.446254	0.496997
C	-5.559935	1.366148	0.432926

H	-5.884531	0.539719	1.064788
H	-7.458125	2.364682	0.467428
H	2.725787	0.890105	-1.708793
C	2.893286	-2.987411	1.877859
H	3.569446	-2.999427	2.744054
H	1.930208	-3.429567	2.157997
H	3.352432	-3.572075	1.067359
C	3.706538	-0.911622	1.169925
O	2.624311	-1.662687	1.456406
O	4.834693	-1.344318	1.299846
C	2.171602	1.135785	1.054434
O	1.067517	0.683729	1.354163
C	3.380708	0.425368	0.687296
C	3.820663	2.756448	0.560478
C	4.331971	4.038226	0.355185
C	2.475135	2.590613	0.898353
C	3.478492	5.133740	0.508256
H	5.379532	4.187190	0.083633
C	1.615499	3.677901	1.050534
C	2.130227	4.958234	0.853403
H	3.865643	6.143343	0.356245
H	0.566126	3.515496	1.306346
H	1.484509	5.831706	0.962752
C	4.505699	1.415494	0.469637
H	5.286359	1.311718	1.242061
H	5.013427	1.294647	-0.499905

SMDopt/16_TS_SSb

Frequencies, energies and thermodynamic properties:

Lowest Vibrational Mode (1/cm) = -157.8511

2nd Lowest Vibrational Mode (1/cm) = 15.7896

E(RM062X) (a.u.) = -2420.02782690

Thermal correction to Enthalpy (a.u.) = 0.632508

Thermal correction to Gibbs Free Energy (a.u.) = 0.522079

Total Entropy (cal/Kmol) = 232.418

Esp(RM062X+D3) (a.u.) = -2422.44660505

Esp(D3) (a.u.) = -0.0095437758

Esp(RM062X) (a.u.) = -2422.4370612742

Optimised cartesian coordinates (Angstrom):

C	6.759225	-2.270148	-0.047417
C	6.320010	-3.549885	-0.419051
C	4.970842	-3.806841	-0.636122
C	4.072717	-2.752572	-0.467074
C	4.510631	-1.483113	-0.087187
H	7.045649	-4.354493	-0.543709
H	4.624127	-4.798406	-0.930821
C	2.252410	-1.094105	-0.271296
C	1.162766	1.076016	0.138677
C	2.353085	1.303816	1.084986
C	3.636844	0.810319	0.447375
H	4.456873	0.828009	1.176154
S	2.334855	-2.785577	-0.687630
N	3.447452	-0.581245	0.028283
N	1.122575	-0.354841	-0.264816
C	2.460046	2.756936	1.601636
C	3.300996	3.682970	0.717779
C	3.017346	2.763903	3.025262
H	1.431451	3.153029	1.641363
H	4.368689	3.419276	0.780913
H	3.006211	3.659201	-0.340630
H	3.200595	4.719632	1.071278
H	2.370074	2.203662	3.715133
H	4.020699	2.309386	3.054226
H	3.106542	3.794072	3.400206
H	2.158576	0.657226	1.955233
H	0.259477	1.241793	0.742213
C	1.134778	1.993972	-1.066465
C	0.375266	3.168225	-1.004605
C	1.883803	1.723173	-2.215673
C	0.391964	4.075997	-2.061721
H	-0.235431	3.366878	-0.119254
C	1.899333	2.631828	-3.274917
H	2.459552	0.797807	-2.300063
C	1.161042	3.812670	-3.196764

H	-0.202912	4.988955	-2.002176
H	2.489126	2.411897	-4.166376
H	1.173646	4.521522	-4.026493
C	-1.245834	-0.065096	-0.863207
H	-1.128092	1.010107	-0.765665
C	-2.467087	-0.559685	-1.186372
C	-3.467496	0.387785	-1.782300
F	-3.222113	0.520602	-3.101187
F	-4.726887	-0.023137	-1.682501
F	-3.387789	1.612242	-1.260312
O	-0.115772	-2.147106	-0.927206
C	-0.107934	-0.954478	-0.690379
H	3.931859	1.401372	-0.432439
C	5.866052	-1.218423	0.123107
H	6.216812	-0.225352	0.402419
H	7.823703	-2.091459	0.109524
H	-2.639408	-1.620141	-1.386901
C	-6.932416	-2.055970	-0.511610
H	-7.134618	-3.063165	-0.118440
H	-7.848770	-1.455222	-0.471194
H	-6.601162	-2.153783	-1.556484
C	-4.740415	-1.928660	0.289391
O	-5.970048	-1.377181	0.267102
O	-4.533910	-3.027746	-0.205716
C	-3.837315	0.227758	1.419325
O	-4.722073	1.072302	1.301733
C	-3.726962	-1.125677	0.929096
C	-1.763231	-0.683311	2.162439
C	-0.524970	-0.684628	2.806984
C	-2.537503	0.480984	2.133937
C	-0.086237	0.492108	3.420080
H	0.085920	-1.590626	2.845236
C	-2.090483	1.666536	2.717810
C	-0.854332	1.665067	3.366896
H	0.868049	0.498020	3.950835
H	-2.712483	2.563750	2.674886
H	-0.484200	2.573814	3.847011

C -2.493244 -1.805322 1.472789
H -1.871660 -2.290171 0.705278
H -2.743405 -2.597168 2.202795

SMDopt/16_TS_SSc
Frequencies, energies and thermodynamic properties:
Lowest Vibrational Mode (1/cm) = -161.0352
2nd Lowest Vibrational Mode (1/cm) = 13.6185
E(RM062X) (a.u.) = -2420.02650616
Thermal correction to Enthalpy (a.u.) = 0.632419
Thermal correction to Gibbs Free Energy (a.u.) = 0.521353
Total Entropy (cal/Kmol) = 233.757
Esp(RM062X+D3) (a.u.) = -2422.44530805
Esp(D3) (a.u.) = -0.0095634773
Esp(RM062X) (a.u.) = -2422.4357445727
Optimised cartesian coordinates (Angstrom):
C 6.629704 -2.457135 0.055391
C 6.147204 -3.724297 -0.305474
C 4.791802 -3.933829 -0.534412
C 3.931588 -2.845010 -0.388985
C 4.412648 -1.587784 -0.021298
H 6.843782 -4.556698 -0.412061
H 4.411699 -4.915571 -0.820313
C 2.172812 -1.117009 -0.234962
C 1.164844 1.101082 0.123449
C 2.354431 1.305043 1.076120
C 3.623738 0.745614 0.464471
H 4.435099 0.744432 1.202963
S 2.196175 -2.816053 -0.628726
N 3.383290 -0.644995 0.069387
N 1.071423 -0.335954 -0.247979
C 2.515106 2.765667 1.557233
C 3.391973 3.638820 0.654305
C 3.069330 2.787878 2.982025
H 1.501809 3.200620 1.583656
H 4.449338 3.338583 0.727867
H 3.099903 3.600890 -0.404354

H	3.328061	4.686477	0.982968
H	2.398963	2.272048	3.684235
H	4.054113	2.295620	3.026530
H	3.197711	3.823248	3.330244
H	2.125011	0.688779	1.960166
H	0.264774	1.316410	0.715211
C	1.179793	1.989619	-1.103329
C	0.458954	3.188973	-1.076815
C	1.931993	1.668234	-2.237388
C	0.517032	4.071756	-2.153437
H	-0.153933	3.428301	-0.203082
C	1.989174	2.551905	-3.316245
H	2.478914	0.723381	-2.294316
C	1.289470	3.757850	-3.273124
H	-0.048055	5.004776	-2.120841
H	2.581397	2.293081	-4.195560
H	1.334814	4.447179	-4.118002
C	-1.285324	0.028637	-0.853206
H	-1.140063	1.098908	-0.741639
C	-2.515862	-0.428452	-1.195795
C	-3.484446	0.549923	-1.794012
F	-3.236351	0.665921	-3.115062
F	-4.754537	0.186915	-1.686852
F	-3.355469	1.774759	-1.280510
O	-0.224053	-2.088745	-0.906355
C	-0.176888	-0.895700	-0.675280
H	3.953411	1.306075	-0.423063
C	5.774351	-1.371015	0.202100
H	6.157865	-0.387901	0.473365
H	7.698239	-2.316156	0.222842
H	-2.707618	-1.481934	-1.411341
C	-5.466175	-3.814368	-0.544214
H	-5.900829	-3.392242	-1.462220
H	-4.969655	-4.764327	-0.774954
H	-6.281113	-3.988374	0.173763
C	-4.906257	-1.712664	0.360967
O	-4.478184	-2.960426	-0.004367

O -6.064180 -1.390403 0.197310
C -3.918229 0.428447 1.360195
O -4.800707 1.270547 1.210387
C -3.818074 -0.942062 0.918942
C -1.862084 -0.472551 2.157736
C -0.630637 -0.462916 2.815358
C -2.622710 0.697900 2.075875
C -0.183656 0.732066 3.385583
H -0.031459 -1.373941 2.896461
C -2.166998 1.900546 2.616496
C -0.936868 1.910920 3.276930
H 0.765177 0.747999 3.925755
H -2.777504 2.802684 2.532006
H -0.560381 2.833876 3.723718
C -2.596192 -1.613265 1.503628
H -1.969849 -2.131711 0.762348
H -2.860496 -2.374083 2.260454

SMDopt/16_TS_SSd

Frequencies, energies and thermodynamic properties:

Lowest Vibrational Mode (1/cm) = -273.1742
2nd Lowest Vibrational Mode (1/cm) = 17.8895
E(RM062X) (a.u.) = -2420.03763112
Thermal correction to Enthalpy (a.u.) = 0.632855
Thermal correction to Gibbs Free Energy (a.u.) = 0.524141
Total Entropy (cal/Kmol) = 228.807
Esp(RM062X+D3) (a.u.) = -2422.44758426
Esp(D3) (a.u.) = -0.0091158763
Esp(RM062X) (a.u.) = -2422.4384683837

Optimised cartesian coordinates (Angstrom):

C -6.697956 2.104994 0.064016
C -6.400595 3.116157 -0.859956
C -5.125746 3.230366 -1.407876
C -4.159193 2.307504 -1.011462
C -4.456116 1.306744 -0.083803
H -7.176905 3.822156 -1.157325
H -4.889849 4.015438 -2.127765

C	-2.255026	0.802569	-0.529221
C	-1.040640	-1.098057	0.420376
C	-1.951085	-0.853983	1.634281
C	-3.373084	-0.572884	1.182868
H	-3.984322	-0.217615	2.023021
S	-2.480368	2.225083	-1.522023
N	-3.348754	0.495046	0.179619
N	-1.142286	0.067863	-0.491197
C	-1.866657	-1.964326	2.705256
C	-2.859391	-3.113328	2.510108
C	-2.042083	-1.357726	4.097707
H	-0.846162	-2.377805	2.645942
H	-3.890577	-2.780781	2.709612
H	-2.828757	-3.540474	1.497522
H	-2.634669	-3.920997	3.222290
H	-1.986430	-2.137135	4.872052
H	-1.264132	-0.609396	4.308559
H	-3.023463	-0.864769	4.188740
H	-1.559951	0.071588	2.087177
H	-0.009768	-1.074477	0.799054
C	-1.280190	-2.406840	-0.300327
C	-0.444398	-3.489768	0.000233
C	-2.326660	-2.583090	-1.211866
C	-0.679225	-4.741315	-0.568007
H	0.403068	-3.332227	0.673455
C	-2.558328	-3.834955	-1.783853
H	-2.970014	-1.743485	-1.488214
C	-1.742858	-4.918501	-1.454676
H	-0.024307	-5.579957	-0.324445
H	-3.379287	-3.962874	-2.491640
H	-1.927117	-5.897198	-1.901402
C	1.105752	-0.436831	-1.292593
H	0.932350	-1.487031	-1.073447
C	2.403113	0.003773	-1.448929
C	3.379186	-0.984366	-2.026230
F	3.194692	-1.079634	-3.354339
F	4.662539	-0.663092	-1.857220

F	3.215365	-2.218429	-1.534185
O	0.030950	1.643054	-1.668542
C	0.044102	0.519452	-1.193604
H	-3.860206	-1.456717	0.744815
C	-5.732840	1.186422	0.467046
H	-5.970788	0.397273	1.180045
H	-7.705225	2.031273	0.476034
H	2.599041	1.032111	-1.766396
C	4.475004	-3.016749	1.271601
H	3.891573	-3.589214	0.536744
H	5.529519	-3.309459	1.218325
H	4.081535	-3.226327	2.276409
C	3.200491	-1.067992	0.979139
O	4.427534	-1.630631	0.982671
O	2.204110	-1.709991	1.253349
C	2.155496	1.218084	0.983448
O	0.992482	0.931629	1.248437
C	3.235750	0.330194	0.572662
C	4.074610	2.554556	0.670826
C	4.808003	3.738322	0.587918
C	2.707960	2.603626	0.955147
C	4.150217	4.951538	0.805565
H	5.876332	3.721407	0.360974
C	2.042561	3.809816	1.173241
C	2.778618	4.990857	1.096517
H	4.712226	5.885896	0.747607
H	0.972048	3.815152	1.389765
H	2.289744	5.953247	1.259116
C	4.527113	1.126075	0.492736
H	5.226456	0.835799	1.294487
H	5.071789	1.000785	-0.453053

SMDopt/16_TS_SSe

Frequencies, energies and thermodynamic properties:

Lowest Vibrational Mode (1/cm) = -213.0285

2nd Lowest Vibrational Mode (1/cm) = 14.3624

E(RM062X) (a.u.) = -2420.03012170

Thermal correction to Enthalpy (a.u.) = 0.632555

Thermal correction to Gibbs Free Energy (a.u.) = 0.522380

Total Entropy (cal/Kmol) = 231.881

Esp(RM062X+D3) (a.u.) = -2422.44449629

Esp(D3) (a.u.) = -0.0089726091

Esp(RM062X) (a.u.) = -2422.4355236809

Optimised cartesian coordinates (Angstrom):

C	7.232994	-1.203465	0.502974
C	7.108035	-2.413344	-0.195099
C	5.876831	-2.825202	-0.696189
C	4.776127	-1.996284	-0.482731
C	4.901804	-0.797302	0.221079
H	7.986808	-3.040170	-0.351226
H	5.773874	-3.764541	-1.241281
C	2.659454	-0.733328	-0.285900
C	1.151130	1.112272	0.287463
C	2.077679	1.261707	1.505570
C	3.527572	1.121805	1.080243
H	4.185050	1.058153	1.957114
S	3.117108	-2.267833	-0.984968
N	3.679437	-0.126362	0.327964
N	1.439810	-0.182627	-0.374317
C	1.815463	2.539238	2.334430
C	2.632819	3.757107	1.895413
C	2.061995	2.254416	3.816160
H	0.746387	2.779655	2.213570
H	3.698531	3.623437	2.140167
H	2.553543	3.963279	0.818404
H	2.284180	4.649934	2.434927
H	1.399494	1.458264	4.186033
H	3.103676	1.937798	3.985693
H	1.884076	3.156438	4.420011
H	1.838423	0.396159	2.144526
H	0.135043	1.005204	0.687413
C	1.193101	2.268091	-0.689399
C	0.238275	3.284849	-0.570340
C	2.182762	2.374164	-1.672365

C	0.290635	4.405763	-1.397231
H	-0.552215	3.193878	0.179281
C	2.234751	3.495739	-2.501345
H	2.921560	1.579671	-1.805752
C	1.294611	4.516866	-2.360567
H	-0.458512	5.192532	-1.292685
H	3.013085	3.568951	-3.262887
H	1.336513	5.392940	-3.010067
C	-0.855418	-0.206828	-1.254377
H	-0.883441	0.881576	-1.267151
C	-2.008428	-0.933883	-1.388661
C	-3.174449	-0.298581	-2.086092
F	-2.912819	-0.179058	-3.399962
F	-4.299976	-1.007782	-2.000835
F	-3.439958	0.935839	-1.645113
O	0.572260	-2.090391	-1.272120
C	0.368895	-0.921790	-0.989166
H	3.865956	1.955575	0.447293
C	6.134990	-0.377731	0.722597
H	6.237937	0.565013	1.259291
H	8.209567	-0.898728	0.881308
H	-1.970826	-2.021928	-1.482684
C	-0.401003	-3.743947	1.347338
H	0.422862	-3.398704	0.706912
H	-0.140880	-3.555745	2.399046
H	-0.565273	-4.817065	1.195145
C	-1.645338	-1.768548	1.160183
O	-1.615085	-3.102358	0.998870
O	-0.657236	-1.150621	1.540782
C	-4.170186	-1.785692	0.616704
O	-4.442638	-2.950697	0.372458
C	-2.875195	-1.126173	0.776967
C	-4.562393	0.503613	1.128913
C	-5.324343	1.655250	1.320846
C	-5.192114	-0.698804	0.797703
C	-6.713200	1.573751	1.182325
H	-4.849677	2.605803	1.575048

C -6.575005 -0.789170 0.656891
C -7.337401 0.362509 0.854473
H -7.322283 2.467808 1.330893
H -7.037686 -1.743475 0.394993
H -8.423565 0.326525 0.750538
C -3.067994 0.306932 1.217749
H -2.712315 0.464359 2.250754
H -2.516711 1.033194 0.593228

SMDopt/16_TS_SSf

Frequencies, energies and thermodynamic properties:

Lowest Vibrational Mode (1/cm) = -198.0259

2nd Lowest Vibrational Mode (1/cm) = 15.8719

E(RM062X) (a.u.) = -2420.02670263

Thermal correction to Enthalpy (a.u.) = 0.632408

Thermal correction to Gibbs Free Energy (a.u.) = 0.522634

Total Entropy (cal/Kmol) = 231.039

Esp(RM062X+D3) (a.u.) = -2422.44317140

Esp(D3) (a.u.) = -0.0092202221

Esp(RM062X) (a.u.) = -2422.4339511779

Optimised cartesian coordinates (Angstrom):

C -7.219906 -1.031502 -0.562063
C -7.141359 -2.262879 0.105041
C -5.929677 -2.728770 0.605252
C -4.800001 -1.932013 0.421270
C -4.879570 -0.711580 -0.251236
H -8.041930 -2.863697 0.237305
H -5.863261 -3.684368 1.126950
C -2.641612 -0.738283 0.273180
C -1.064294 1.072029 -0.241410
C -1.990664 1.312907 -1.445978
C -3.443026 1.196989 -1.023143
H -4.101809 1.195076 -1.901059
S -3.157582 -2.268839 0.935299
N -3.634248 -0.079544 -0.329460
N -1.396959 -0.237612 0.371422
C -1.691186 2.623163 -2.209846

C	-2.464284	3.843232	-1.701700
C	-1.959240	2.424093	-3.701881
H	-0.613632	2.821177	-2.087275
H	-3.535089	3.758705	-1.945975
H	-2.372402	3.991183	-0.616257
H	-2.087929	4.750148	-2.197237
H	-1.326784	1.627367	-4.119951
H	-3.012380	2.151708	-3.877031
H	-1.756326	3.350414	-4.259141
H	-1.787670	0.477912	-2.135642
H	-0.051415	0.952874	-0.647951
C	-1.057957	2.188936	0.782049
C	-0.068842	3.175485	0.693867
C	-2.036798	2.290432	1.775897
C	-0.077837	4.265738	1.562066
H	0.714620	3.085170	-0.063247
C	-2.045218	3.381679	2.646028
H	-2.801773	1.517377	1.886444
C	-1.072108	4.375132	2.535652
H	0.697438	5.029622	1.481437
H	-2.815684	3.452514	3.415689
H	-1.080593	5.227609	3.217044
C	0.882760	-0.338667	1.285706
H	0.946290	0.747623	1.299916
C	2.010023	-1.093831	1.438759
C	3.194301	-0.445541	2.093917
F	2.948862	-0.260017	3.403466
F	4.310437	-1.168087	2.028378
F	3.462288	0.764050	1.589276
O	-0.623097	-2.162450	1.312245
C	-0.370018	-1.008520	1.019051
H	-3.756005	2.009317	-0.350567
C	-6.093716	-0.237318	-0.750921
H	-6.161003	0.721713	-1.263755
H	-8.182446	-0.684789	-0.940106
H	1.960623	-2.180408	1.539377
C	-0.406975	-2.410450	-1.863784

H -0.788473 -2.949257 -0.984123
H -1.178933 -1.741672 -2.265122
H -0.130501 -3.149385 -2.629681
C 1.799359 -2.274684 -1.053835
O 0.702816 -1.600004 -1.519512
O 1.764597 -3.478398 -0.903734
C 4.252484 -1.844462 -0.597918
O 4.676724 -2.956832 -0.320193
C 2.886838 -1.368058 -0.767803
C 4.343145 0.444557 -1.232776
C 4.946836 1.671906 -1.500377
C 5.125668 -0.645119 -0.843143
C 6.335903 1.779663 -1.376366
H 4.351232 2.536944 -1.801646
C 6.509078 -0.547354 -0.717646
C 7.114164 0.680912 -0.988975
H 6.822487 2.735058 -1.583657
H 7.093687 -1.417869 -0.411217
H 8.196486 0.791977 -0.898204
C 2.884725 0.052316 -1.281017
H 2.261446 0.733093 -0.669915
H 2.492902 0.125503 -2.309947

SMDopt/9_TS_SRa

Frequencies, energies and thermodynamic properties:

Lowest Vibrational Mode (1/cm) = -216.4889

2nd Lowest Vibrational Mode (1/cm) = 20.2966

E(RM062X) (a.u.) = -2267.75772887

Thermal correction to Enthalpy (a.u.) = 0.606148

Thermal correction to Gibbs Free Energy (a.u.) = 0.503266

Total Entropy (cal/Kmol) = 216.534

Esp(RM062X+D3) (a.u.) = -2270.00820932

Esp(D3) (a.u.) = -0.0085846993

Esp(RM062X) (a.u.) = -2269.9996246207

Optimised cartesian coordinates (Angstrom):

C 6.685079 -1.118298 0.121916
C 6.554169 -2.326522 -0.577437

C	5.307281	-2.772377	-1.005703
C	4.197028	-1.979467	-0.718497
C	4.329381	-0.781016	-0.014367
H	7.440102	-2.925119	-0.792740
H	5.200118	-3.710677	-1.551761
C	2.060575	-0.780082	-0.389421
C	0.531549	1.011299	0.294607
C	1.530267	1.193508	1.448890
C	2.955657	1.095482	0.936445
H	3.666889	1.047341	1.771178
S	2.518771	-2.298426	-1.121548
N	3.096886	-0.146321	0.169961
N	0.822180	-0.269388	-0.395603
C	1.275732	2.464174	2.291023
C	2.016995	3.710417	1.799838
C	1.626711	2.195302	3.754682
H	0.192480	2.662451	2.236998
H	3.101127	3.615819	1.971316
H	1.856710	3.913479	0.731345
H	1.673822	4.590094	2.364003
H	1.019302	1.376825	4.167996
H	2.688305	1.918477	3.858365
H	1.453968	3.092504	4.367227
H	1.365241	0.327231	2.109969
H	-0.460384	0.873687	0.749754
C	0.476060	2.174037	-0.672884
C	-0.534478	3.129096	-0.505163
C	1.423652	2.349759	-1.686931
C	-0.570233	4.265555	-1.312390
H	-1.299841	2.958273	0.257235
C	1.385024	3.486019	-2.496348
H	2.201169	1.600267	-1.858079
C	0.395433	4.450812	-2.303591
H	-1.359608	5.006347	-1.172327
H	2.131076	3.616209	-3.282292
H	0.368185	5.339930	-2.936082
C	-1.510339	-0.328053	-1.151337

H	-1.557873	0.758337	-1.150053
C	-2.663078	-1.069688	-1.158916
C	-3.885904	-0.497642	-1.806689
F	-3.696290	-0.364524	-3.131146
F	-4.961370	-1.278058	-1.668622
F	-4.206087	0.719626	-1.354048
O	-0.054623	-2.192607	-1.250576
C	-0.266071	-1.025803	-0.962657
H	3.231633	1.940613	0.288519
C	5.577992	-0.327890	0.414548
H	5.685623	0.613873	0.952136
H	7.673281	-0.786353	0.442878
H	-2.607204	-2.159367	-1.223440
C	-0.097442	-2.400768	1.903794
H	0.133921	-3.060346	1.054274
H	0.776643	-1.784534	2.149285
H	-0.363512	-3.020447	2.771152
C	-2.357974	-2.085265	1.377199
O	-1.149065	-1.508101	1.574883
O	-2.501426	-3.288965	1.489386
C	-4.840730	-1.674007	1.207740
C	-3.389357	0.261517	1.306841
C	-5.726603	-0.412254	1.151033
H	-5.131179	-2.419991	0.453061
C	-4.814715	0.737635	1.583915
H	-6.620113	-0.508225	1.782996
H	-4.875431	0.920175	2.670914
C	-3.425350	-1.161220	1.032152
O	-2.420254	1.016378	1.331073
H	-4.935317	-2.175940	2.188050
H	-6.072519	-0.244703	0.122669
H	-5.011637	1.695937	1.082192

SMDopt/9_TS_SRb

Frequencies, energies and thermodynamic properties:

Lowest Vibrational Mode (1/cm) = -216.5058

2nd Lowest Vibrational Mode (1/cm) = 20.3415

E(RM062X) (a.u.) = -2267.75772887

Thermal correction to Enthalpy (a.u.) = 0.606148

Thermal correction to Gibbs Free Energy (a.u.) = 0.503271

Total Entropy (cal/Kmol) = 216.524

Esp(RM062X+D3) (a.u.) = -2270.00820888

Esp(D3) (a.u.) = -0.0085847589

Esp(RM062X) (a.u.) = -2269.9996241211

Optimised cartesian coordinates (Angstrom):

C	6.685071	-1.118275	0.122007
C	6.554188	-2.326479	-0.577386
C	5.307314	-2.772329	-1.005699
C	4.197049	-1.979434	-0.718500
C	4.329375	-0.781004	-0.014330
H	7.440131	-2.925064	-0.792684
H	5.200172	-3.710614	-1.551787
C	2.060580	-0.780071	-0.389450
C	0.531521	1.011282	0.294579
C	1.530215	1.193497	1.448881
C	2.955614	1.095482	0.936459
H	3.666839	1.047359	1.771200
S	2.518806	-2.298394	-1.121606
N	3.096870	-0.146322	0.169981
N	0.822182	-0.269386	-0.395654
C	1.275648	2.464171	2.290997
C	2.016992	3.710389	1.799873
C	1.626484	2.195282	3.754686
H	0.192409	2.662488	2.236872
H	3.101119	3.615714	1.971339
H	1.856712	3.913510	0.731389
H	1.673879	4.590060	2.364083
H	1.018964	1.376867	4.167957
H	2.688043	1.918352	3.858457
H	1.453779	3.092505	4.367211
H	1.365176	0.327225	2.109966
H	-0.460414	0.873647	0.749713
C	0.476037	2.174034	-0.672897
C	-0.534505	3.129090	-0.505184

C	1.423644	2.349765	-1.686928
C	-0.570249	4.265555	-1.312403
H	-1.299881	2.958260	0.257199
C	1.385026	3.486030	-2.496340
H	2.201164	1.600275	-1.858069
C	0.395430	4.450820	-2.303590
H	-1.359627	5.006345	-1.172347
H	2.131088	3.616225	-3.282274
H	0.368188	5.339940	-2.936077
C	-1.510337	-0.328085	-1.151373
H	-1.557878	0.758306	-1.150117
C	-2.663075	-1.069726	-1.158919
C	-3.885907	-0.497682	-1.806684
F	-3.696318	-0.364612	-3.131151
F	-4.961386	-1.278071	-1.668572
F	-4.206058	0.719608	-1.354078
O	-0.054594	-2.192618	-1.250626
C	-0.266060	-1.025818	-0.962706
H	3.231585	1.940611	0.288529
C	5.577971	-0.327884	0.414633
H	5.685581	0.613862	0.952255
H	7.673263	-0.786334	0.443005
H	-2.607204	-2.159405	-1.223433
C	-0.097340	-2.400645	1.903782
H	0.134042	-3.060246	1.054284
H	0.776730	-1.784382	2.149255
H	-0.363391	-3.020306	2.771158
C	-2.357895	-2.085224	1.377232
O	-1.148991	-1.508022	1.574841
O	-2.501324	-3.288917	1.489518
C	-4.840657	-1.674070	1.207746
C	-3.389365	0.261516	1.306818
C	-5.726590	-0.412356	1.151079
H	-5.131087	-2.420061	0.453066
C	-4.814735	0.737573	1.583927
H	-6.620062	-0.508371	1.783090
H	-4.875432	0.920104	2.670929

C	-3.425299	-1.161226	1.032136
O	-2.420300	1.016427	1.330989
H	-4.935199	-2.176018	2.188051
H	-6.072572	-0.244819	0.122735
H	-5.011704	1.695870	1.082212

SMDopt/9_TS_SRc

Frequencies, energies and thermodynamic properties:

Lowest Vibrational Mode (1/cm) = -159.4732

2nd Lowest Vibrational Mode (1/cm) = 18.7210

E(RM062X) (a.u.) = -2267.74953607

Thermal correction to Enthalpy (a.u.) = 0.605839

Thermal correction to Gibbs Free Energy (a.u.) = 0.500881

Total Entropy (cal/Kmol) = 220.902

Esp(RM062X+D3) (a.u.) = -2270.00435534

Esp(D3) (a.u.) = -0.0085046751

Esp(RM062X) (a.u.) = -2269.9958506649

Optimised cartesian coordinates (Angstrom):

C	6.847289	-1.216297	0.296739
C	6.725239	-2.432400	-0.391414
C	5.485327	-2.881568	-0.834720
C	4.372313	-2.082971	-0.573790
C	4.495473	-0.877227	0.118904
H	7.613619	-3.034537	-0.585642
H	5.384973	-3.825448	-1.372238
C	2.233750	-0.879575	-0.287221
C	0.705651	0.942304	0.326210
C	1.678424	1.135267	1.500853
C	3.113315	1.015016	1.022172
H	3.804416	0.973055	1.873950
S	2.701512	-2.401973	-1.000995
N	3.260786	-0.239513	0.277156
N	0.992138	-0.365449	-0.320251
C	1.417661	2.425931	2.310470
C	2.178137	3.655531	1.806849
C	1.737224	2.186007	3.786146
H	0.337438	2.631931	2.231129

H	3.257825	3.557522	2.002434
H	2.040741	3.836310	0.731159
H	1.829784	4.549572	2.344512
H	1.116612	1.379955	4.204132
H	2.794811	1.906079	3.917503
H	1.557041	3.097296	4.375169
H	1.488743	0.281729	2.172427
H	-0.299008	0.848793	0.763709
C	0.681929	2.078596	-0.673440
C	-0.325885	3.043324	-0.550590
C	1.649647	2.219167	-1.673382
C	-0.340576	4.156214	-1.390380
H	-1.107260	2.899290	0.201714
C	1.631962	3.332428	-2.514853
H	2.424997	1.460601	-1.810553
C	0.644461	4.307391	-2.368095
H	-1.128340	4.904506	-1.286024
H	2.392931	3.436276	-3.290268
H	0.633867	5.177812	-3.026491
C	-1.324719	-0.414930	-1.110347
H	-1.342134	0.672415	-1.130069
C	-2.493066	-1.104958	-1.185626
C	-3.684696	-0.447129	-1.811231
F	-3.637827	-0.619416	-3.148447
F	-4.847290	-0.942319	-1.413847
F	-3.706670	0.875976	-1.609899
O	0.123741	-2.292710	-1.185711
C	-0.081811	-1.128441	-0.899064
H	3.413597	1.845999	0.366915
C	5.737809	-0.420839	0.563122
H	5.838414	0.526580	1.091910
H	7.830963	-0.882797	0.629662
H	-2.513091	-2.196272	-1.217340
C	-4.780661	2.175528	0.915162
H	-4.183895	2.589944	0.089965
H	-5.843942	2.373135	0.734319
H	-4.469900	2.663748	1.850708

C -3.392151 0.310051 1.194738
O -4.642462 0.772020 0.995111
O -2.449603 1.087146 1.338600
C -1.975595 -1.707933 1.716555
C -4.299508 -2.121013 1.214821
C -2.121031 -3.217215 1.465140
H -1.077423 -1.278922 1.239605
C -3.628814 -3.467743 1.540198
H -1.545358 -3.824094 2.177946
H -3.942137 -3.741267 2.562546
C -3.249498 -1.118951 1.182885
O -5.506867 -2.012873 1.067114
H -1.857282 -1.493311 2.795991
H -1.741793 -3.452644 0.458220
H -4.001845 -4.255174 0.868523

SMDopt/9_TS_SRd
Frequencies, energies and thermodynamic properties:
Lowest Vibrational Mode (1/cm) = -261.1391
2nd Lowest Vibrational Mode (1/cm) = 25.2741
E(RM062X) (a.u.) = -2267.75871662
Thermal correction to Enthalpy (a.u.) = 0.606660
Thermal correction to Gibbs Free Energy (a.u.) = 0.504039
Total Entropy (cal/Kmol) = 215.984
Esp(RM062X+D3) (a.u.) = -2270.00734332
Esp(D3) (a.u.) = -0.0084580101
Esp(RM062X) (a.u.) = -2269.9988853099
Optimised cartesian coordinates (Angstrom):
C 6.688149 -1.107140 0.147616
C 6.571793 -2.271192 -0.624532
C 5.332593 -2.692089 -1.099393
C 4.216051 -1.918654 -0.786224
C 4.333869 -0.763796 -0.009845
H 7.462339 -2.855161 -0.860086
H 5.236972 -3.597070 -1.701096
C 2.070467 -0.741188 -0.423261
C 0.536092 1.015211 0.319050

C	1.493992	1.106555	1.516893
C	2.936452	1.036713	1.048549
H	3.617621	0.916455	1.901162
S	2.543477	-2.219208	-1.229938
N	3.097876	-0.142196	0.192002
N	0.839639	-0.225628	-0.436671
C	1.213760	2.314231	2.438562
C	1.968513	3.591166	2.060089
C	1.517305	1.941854	3.889994
H	0.132443	2.517397	2.363710
H	3.047143	3.483809	2.257060
H	1.840672	3.868863	1.003991
H	1.607544	4.429659	2.673828
H	0.897286	1.096867	4.223569
H	2.575149	1.656153	4.007405
H	1.324661	2.793276	4.559451
H	1.291301	0.193018	2.099152
H	-0.468067	0.860900	0.738624
C	0.519367	2.234907	-0.576047
C	-0.504621	3.173540	-0.395813
C	1.508867	2.476684	-1.535231
C	-0.512399	4.357595	-1.132608
H	-1.303464	2.952037	0.318021
C	1.497753	3.660051	-2.274874
H	2.297721	1.741750	-1.717115
C	0.494164	4.607329	-2.067177
H	-1.312411	5.085033	-0.982954
H	2.276321	3.841031	-3.018090
H	0.488309	5.533233	-2.645099
C	-1.479849	-0.262275	-1.186795
H	-1.497883	0.823738	-1.148908
C	-2.673715	-0.949925	-1.176534
C	-3.848158	-0.260291	-1.807470
F	-3.801286	-0.408790	-3.142358
F	-5.040116	-0.729797	-1.428677
F	-3.856513	1.056967	-1.573498
O	-0.046068	-2.145928	-1.314447

C	-0.253821	-0.981307	-1.009818
H	3.240238	1.928711	0.480608
C	5.573857	-0.336987	0.467722
H	5.669278	0.570928	1.062858
H	7.669888	-0.793670	0.504985
H	-2.689090	-2.036044	-1.304429
C	-0.977391	-3.865054	1.491023
H	-0.557077	-3.649772	2.483884
H	-1.230842	-4.928972	1.419930
H	-0.234378	-3.601098	0.724010
C	-2.076838	-1.802321	1.301475
O	-2.183197	-3.149913	1.279295
O	-1.009481	-1.260879	1.527885
C	-4.639484	-1.874783	1.096338
C	-3.489560	0.277785	1.211701
C	-5.700345	-0.776693	1.384564
H	-4.889084	-2.455463	0.194648
C	-4.983494	0.580884	1.315906
H	-6.130965	-0.929581	2.383837
H	-5.154104	1.216235	2.197615
C	-3.322024	-1.143416	0.960968
O	-2.618408	1.137321	1.279391
H	-4.590715	-2.602248	1.920927
H	-6.530612	-0.829316	0.668400
H	-5.274036	1.176800	0.436619

SMDopt/9_TS_SRe

Frequencies, energies and thermodynamic properties:

Lowest Vibrational Mode (1/cm) = -110.3919

2nd Lowest Vibrational Mode (1/cm) = 17.7724

E(RM062X) (a.u.) = -2267.74528229

Thermal correction to Enthalpy (a.u.) = 0.605813

Thermal correction to Gibbs Free Energy (a.u.) = 0.500327

Total Entropy (cal/Kmol) = 222.013

Esp(RM062X+D3) (a.u.) = -2270.00346167

Esp(D3) (a.u.) = -0.0086275392

Esp(RM062X) (a.u.) = -2269.9948341308

Optimised cartesian coordinates (Angstrom):

C	6.644471	-1.660591	0.362242
C	6.428804	-2.856936	-0.338162
C	5.165360	-3.188003	-0.816005
C	4.123231	-2.292508	-0.574493
C	4.338738	-1.107858	0.131204
H	7.262881	-3.537206	-0.514238
H	4.993779	-4.115631	-1.363919
C	2.095606	-0.905560	-0.322011
C	0.719396	1.036341	0.292341
C	1.694338	1.148641	1.475541
C	3.116333	0.899818	1.010786
H	3.791243	0.798729	1.870162
S	2.445027	-2.447893	-1.056968
N	3.162638	-0.363047	0.267349
N	0.897083	-0.288850	-0.355622
C	1.545566	2.464856	2.272440
C	2.422457	3.614140	1.768245
C	1.827603	2.208954	3.752892
H	0.490017	2.770477	2.179602
H	3.485146	3.421613	1.985209
H	2.321717	3.792634	0.688229
H	2.146882	4.542688	2.289317
H	1.131038	1.468033	4.171623
H	2.853478	1.832873	3.895208
H	1.728937	3.138472	4.332533
H	1.422344	0.320480	2.150333
H	-0.289395	1.023575	0.728983
C	0.806715	2.161388	-0.718005
C	-0.082967	3.235764	-0.607001
C	1.778136	2.180882	-1.724521
C	0.016895	4.329244	-1.465580
H	-0.860811	3.210382	0.160034
C	1.877825	3.275194	-2.584574
H	2.466518	1.341900	-1.853600
C	1.003517	4.354316	-2.452193
H	-0.682444	5.161219	-1.367185

H	2.641569	3.281121	-3.364104
H	1.082871	5.208728	-3.126530
C	-1.442642	-0.160109	-1.102815
H	-1.439267	0.921508	-0.986717
C	-2.613873	-0.808074	-1.292332
C	-3.819185	-0.037088	-1.735333
F	-3.831303	0.036478	-3.082909
F	-4.968097	-0.591381	-1.382864
F	-3.807772	1.225500	-1.295292
O	-0.068625	-2.065984	-1.405510
C	-0.212731	-0.932397	-0.991483
H	3.495078	1.699662	0.357317
C	5.606704	-0.768351	0.607971
H	5.780614	0.162942	1.146629
H	7.646013	-1.420846	0.721503
H	-2.662519	-1.884400	-1.468023
C	-3.007031	2.287394	1.810347
H	-3.766769	2.443499	2.590049
H	-2.070512	2.772144	2.114649
H	-3.376683	2.740181	0.877901
C	-3.806883	0.115303	1.357711
O	-2.721058	0.915682	1.638394
O	-4.917414	0.604723	1.289527
C	-2.049896	-1.747204	1.595108
C	-4.307660	-2.354007	0.977695
C	-2.020425	-3.211965	1.122125
H	-1.221316	-1.148246	1.172937
C	-3.486713	-3.648073	1.128102
H	-1.375922	-3.846031	1.747792
H	-3.771778	-4.098805	2.094828
C	-3.399485	-1.249413	1.166431
O	-5.515348	-2.367815	0.774375
H	-1.915348	-1.681866	2.692069
H	-1.618441	-3.251566	0.097896
H	-3.756486	-4.371512	0.344174

SMDopt/9_TS_SRf

Frequencies, energies and thermodynamic properties:

Lowest Vibrational Mode (1/cm) = -255.4191

2nd Lowest Vibrational Mode (1/cm) = 14.8717

E(RM062X) (a.u.) = -2267.74704144

Thermal correction to Enthalpy (a.u.) = 0.605840

Thermal correction to Gibbs Free Energy (a.u.) = 0.499913

Total Entropy (cal/Kmol) = 222.941

Esp(RM062X+D3) (a.u.) = -2270.00080092

Esp(D3) (a.u.) = -0.0081567789

Esp(RM062X) (a.u.) = -2269.9926441411

Optimised cartesian coordinates (Angstrom):

C	6.522824	-2.232582	0.316923
C	6.163894	-3.415073	-0.345130
C	4.856474	-3.623797	-0.774696
C	3.919703	-2.621079	-0.527838
C	4.278297	-1.448285	0.139305
H	6.917490	-4.181803	-0.528854
H	4.572196	-4.541421	-1.292212
C	2.051617	-1.021645	-0.260228
C	0.924991	1.070942	0.292608
C	1.907390	1.105033	1.473679
C	3.291516	0.688018	1.013146
H	3.951487	0.525114	1.875093
S	2.212806	-2.623519	-0.941014
N	3.190590	-0.581956	0.289392
N	0.939078	-0.280930	-0.304922
C	1.904367	2.444632	2.244352
C	2.907045	3.478213	1.725036
C	2.143968	2.186976	3.732127
H	0.891056	2.866363	2.136522
H	3.941150	3.167920	1.943707
H	2.824147	3.651103	0.642298
H	2.741432	4.440196	2.231977
H	1.361656	1.540493	4.156389
H	3.116870	1.694810	3.891684
H	2.150458	3.131999	4.295050
H	1.537651	0.326596	2.161037

H	-0.074226	1.182799	0.726064
C	1.136310	2.151611	-0.746632
C	0.372586	3.322063	-0.668106
C	2.102472	2.034054	-1.751526
C	0.591717	4.371663	-1.559028
H	-0.401120	3.407445	0.099915
C	2.320692	3.083584	-2.644972
H	2.693288	1.119712	-1.850886
C	1.571755	4.256594	-2.546223
H	-0.009262	5.279763	-1.486331
H	3.078535	2.981465	-3.423664
H	1.743548	5.076168	-3.246136
C	-1.381379	0.047770	-1.046819
H	-1.232670	1.126343	-1.062166
C	-2.653411	-0.485803	-1.058320
C	-3.749757	0.220875	-1.788478
F	-3.544086	0.164767	-3.117364
F	-4.951318	-0.327090	-1.581892
F	-3.839881	1.520622	-1.482523
O	-0.262749	-2.041605	-1.110026
C	-0.283636	-0.853604	-0.840280
H	3.757459	1.431730	0.349710
C	5.588703	-1.232452	0.569573
H	5.875247	-0.312613	1.079164
H	7.554472	-2.088479	0.640539
H	-2.732565	-1.574795	-1.102115
C	-6.007328	-3.225519	0.795721
H	-5.671145	-3.905121	1.591999
H	-7.094733	-3.100508	0.851129
H	-5.732429	-3.664037	-0.174547
C	-4.092910	-1.868675	0.881073
O	-5.444298	-1.936239	0.949534
O	-3.425532	-2.870185	0.729433
C	-4.615460	0.622267	1.282605
C	-2.374248	-0.172275	1.666816
C	-3.734090	1.829511	1.637508
H	-5.304835	0.829633	0.452738

C -2.514969 1.206335 2.312022
H -4.254523 2.562484 2.269034
H -2.698626 1.037382 3.387910
C -3.621158 -0.488982 0.986088
O -1.331394 -0.810898 1.685466
H -5.245843 0.342380 2.145666
H -3.424072 2.341783 0.714895
H -1.583168 1.786099 2.228230

SMDopt/9_TS_SSa

Frequencies, energies and thermodynamic properties:

Lowest Vibrational Mode (1/cm) = -207.5653

2nd Lowest Vibrational Mode (1/cm) = 21.3310

E(RM062X) (a.u.) = -2267.75576364

Thermal correction to Enthalpy (a.u.) = 0.606165

Thermal correction to Gibbs Free Energy (a.u.) = 0.502164

Total Entropy (cal/Kmol) = 218.888

Esp(RM062X+D3) (a.u.) = -2270.00874846

Esp(D3) (a.u.) = -0.0085310700

Esp(RM062X) (a.u.) = -2270.0002173900

Optimised cartesian coordinates (Angstrom):

C 6.644296 -1.417030 0.281368
C 6.474301 -2.608584 -0.437604
C 5.220862 -2.982858 -0.913225
C 4.144237 -2.135678 -0.654001
C 4.315216 -0.954413 0.070321
H 7.334369 -3.250953 -0.630514
H 5.083775 -3.908476 -1.474223
C 2.061076 -0.831396 -0.375179
C 0.610093 1.029915 0.279084
C 1.573238 1.140531 1.471478
C 3.008275 0.975223 1.006174
H 3.686375 0.878090 1.863945
S 2.466018 -2.360236 -1.119433
N 3.111986 -0.259163 0.223751
N 0.854198 -0.257085 -0.414451
C 1.358830 2.411443 2.323492

C	2.188228	3.619108	1.879130
C	1.635704	2.102730	3.794926
H	0.291780	2.674593	2.231510
H	3.257452	3.461018	2.092050
H	2.083278	3.841663	0.807514
H	1.872800	4.511138	2.440060
H	0.966136	1.315479	4.171051
H	2.674363	1.762258	3.934490
H	1.491863	3.000055	4.414764
H	1.328619	0.270651	2.102401
H	-0.395817	0.932247	0.709742
C	0.652469	2.195877	-0.686348
C	-0.291788	3.220091	-0.551319
C	1.633329	2.302869	-1.678734
C	-0.237507	4.348207	-1.368591
H	-1.078328	3.126888	0.201603
C	1.687798	3.430968	-2.498369
H	2.364108	1.503600	-1.826829
C	0.758409	4.459550	-2.339935
H	-0.979585	5.139747	-1.250468
H	2.459546	3.503795	-3.266679
H	0.802179	5.340952	-2.982088
C	-1.487825	-0.234271	-1.138275
H	-1.518739	0.846355	-1.029342
C	-2.658231	-0.935590	-1.233325
C	-3.871302	-0.220353	-1.747546
F	-3.830860	-0.153328	-3.091336
F	-5.024470	-0.816408	-1.444886
F	-3.945757	1.042709	-1.311854
O	-0.079143	-2.126265	-1.336535
C	-0.259413	-0.971382	-0.989674
H	3.349986	1.815016	0.382891
C	5.570399	-0.572871	0.547179
H	5.708128	0.355683	1.100837
H	7.636903	-1.141263	0.639779
H	-2.652543	-2.006962	-1.449956
C	-3.463527	2.154989	1.689320

H -4.155256 2.151356 2.543296
H -2.588519 2.770209 1.931061
H -3.987426 2.576743 0.818619
C -3.930555 -0.093758 1.233586
O -2.982152 0.854421 1.411787
O -5.113234 0.175411 1.330954
C -4.323323 -2.598675 0.822488
C -2.075291 -1.854201 1.299174
C -3.556637 -3.737898 1.523430
H -5.302018 -2.393245 1.279482
C -2.085622 -3.384630 1.306863
H -3.829930 -4.732981 1.145176
H -1.727773 -3.720707 0.317370
C -3.393516 -1.408342 0.919538
O -1.064306 -1.182705 1.495450
H -4.517478 -2.870368 -0.230492
H -3.778042 -3.717331 2.602366
H -1.392644 -3.778799 2.063297

SMDopt/9_TS_SSb

Frequencies, energies and thermodynamic properties:

Lowest Vibrational Mode (1/cm) = -244.8447
2nd Lowest Vibrational Mode (1/cm) = 21.1728
E(RM062X) (a.u.) = -2267.75895810
Thermal correction to Enthalpy (a.u.) = 0.606082
Thermal correction to Gibbs Free Energy (a.u.) = 0.502234
Total Entropy (cal/Kmol) = 218.566
Esp(RM062X+D3) (a.u.) = -2270.00859027
Esp(D3) (a.u.) = -0.0084179371
Esp(RM062X) (a.u.) = -2270.0001723329

Optimised cartesian coordinates (Angstrom):

C 6.795897 -1.032882 0.278171
C 6.724421 -2.220830 -0.462409
C 5.505018 -2.690787 -0.942697
C 4.361939 -1.942448 -0.666575
C 4.435260 -0.764176 0.079353
H 7.634843 -2.784710 -0.669289

H	5.444449	-3.613967	-1.520800
C	2.178020	-0.819099	-0.364448
C	0.590548	0.922021	0.292778
C	1.521720	1.076220	1.505344
C	2.974517	1.023128	1.068130
H	3.640721	0.941593	1.937147
S	2.705264	-2.303044	-1.125453
N	3.179187	-0.174421	0.248192
N	0.933580	-0.339787	-0.408067
C	1.197432	2.308293	2.378969
C	1.933700	3.587291	1.972279
C	1.477191	1.992291	3.848399
H	0.114152	2.486509	2.275090
H	3.009491	3.510380	2.196952
H	1.824472	3.824478	0.904257
H	1.541194	4.438934	2.547332
H	0.868046	1.145810	4.197838
H	2.537988	1.734102	3.998096
H	1.251942	2.861642	4.483816
H	1.325657	0.178715	2.114068
H	-0.417496	0.760077	0.698636
C	0.569845	2.106085	-0.649551
C	-0.471348	3.034575	-0.523620
C	1.570522	2.326637	-1.602399
C	-0.487187	4.187522	-1.308148
H	-1.277297	2.830032	0.186992
C	1.551662	3.478523	-2.390056
H	2.374480	1.599342	-1.743700
C	0.529527	4.416225	-2.237033
H	-1.301175	4.906762	-1.199769
H	2.339042	3.642158	-3.127992
H	0.517638	5.317594	-2.852451
C	-1.373931	-0.460078	-1.191047
H	-1.416261	0.626223	-1.189905
C	-2.549364	-1.171542	-1.186161
C	-3.737374	-0.537298	-1.852327
F	-3.655082	-0.706244	-3.184548

F	-4.915432	-1.046099	-1.491180
F	-3.798334	0.783975	-1.644986
O	0.119203	-2.303992	-1.254148
C	-0.130537	-1.144461	-0.973119
H	3.271302	1.905256	0.481410
C	5.655153	-0.287269	0.561135
H	5.715372	0.639675	1.131063
H	7.762892	-0.680991	0.639926
H	-2.538867	-2.261831	-1.270007
C	-5.071687	1.908284	1.016022
H	-4.599629	2.409683	0.159128
H	-6.160996	2.011193	0.951558
H	-4.709850	2.378508	1.941750
C	-3.490368	0.173522	1.051641
O	-4.797400	0.520246	1.003700
O	-2.619192	1.021707	1.146015
C	-4.391134	-2.289610	1.057869
C	-2.048571	-1.841765	1.436351
C	-3.817569	-3.354815	2.012531
H	-5.333379	-1.855099	1.417560
C	-2.313728	-3.314204	1.746233
H	-4.264100	-4.347220	1.859654
H	-2.051694	-3.899882	0.846596
C	-3.282619	-1.258831	0.939423
O	-0.953223	-1.304264	1.546938
H	-4.606800	-2.754393	0.081119
H	-4.008666	-3.055822	3.055381
H	-1.678248	-3.663989	2.571067

SMDopt/9_TS_SSc

Frequencies, energies and thermodynamic properties:

Lowest Vibrational Mode (1/cm) = -170.7351

2nd Lowest Vibrational Mode (1/cm) = 19.3034

E(RM062X) (a.u.) = -2267.75273752

Thermal correction to Enthalpy (a.u.) = 0.605790

Thermal correction to Gibbs Free Energy (a.u.) = 0.500785

Total Entropy (cal/Kmol) = 221.000

Esp(RM062X+D3) (a.u.) = -2270.00688117

Esp(D3) (a.u.) = -0.0083875257

Esp(RM062X) (a.u.) = -2269.9984936443

Optimised cartesian coordinates (Angstrom):

C	6.603123	-1.610484	0.112837
C	6.364436	-2.742121	-0.680414
C	5.086738	-3.028200	-1.151262
C	4.055766	-2.153102	-0.810470
C	4.294953	-1.031952	-0.013857
H	7.190610	-3.406930	-0.935249
H	4.896366	-3.906208	-1.770231
C	2.043024	-0.776937	-0.402027
C	0.699001	1.114656	0.390282
C	1.680013	1.095313	1.573225
C	3.098021	0.888624	1.074694
H	3.779607	0.698620	1.913635
S	2.361743	-2.269322	-1.251758
N	3.127972	-0.294572	0.210397
N	0.861523	-0.140645	-0.382529
C	1.546116	2.314646	2.512868
C	2.427875	3.507374	2.133377
C	1.835607	1.891329	3.953201
H	0.492515	2.635574	2.463536
H	3.491031	3.284299	2.316290
H	2.318236	3.806641	1.081137
H	2.165123	4.373923	2.757775
H	1.133980	1.114354	4.290196
H	2.858162	1.491071	4.045030
H	1.750034	2.749884	4.635476
H	1.398623	0.199174	2.149847
H	-0.304626	1.048494	0.827419
C	0.793124	2.338029	-0.496492
C	-0.081623	3.406256	-0.265681
C	1.759506	2.453821	-1.501647
C	0.028172	4.583884	-1.003501
H	-0.854339	3.310784	0.501642
C	1.868749	3.631986	-2.241527

H	2.436850	1.624464	-1.721401
C	1.009350	4.701683	-1.989116
H	-0.658538	5.410162	-0.811683
H	2.628408	3.711903	-3.021030
H	1.096386	5.622277	-2.568781
C	-1.478359	0.059315	-1.116686
H	-1.428738	1.144127	-1.041208
C	-2.680274	-0.566996	-1.251385
C	-3.839302	0.229586	-1.766088
F	-3.799496	0.275169	-3.109703
F	-5.035349	-0.271073	-1.453931
F	-3.815472	1.500983	-1.347012
O	-0.174555	-1.890834	-1.410590
C	-0.283247	-0.751364	-0.996550
H	3.476242	1.748554	0.502011
C	5.575324	-0.739296	0.459239
H	5.767373	0.142467	1.070264
H	7.614392	-1.404588	0.465882
H	-2.750713	-1.643865	-1.419366
C	-0.976269	-3.635734	1.181114
H	-0.638257	-3.521027	2.221651
H	-1.175999	-4.694407	0.975959
H	-0.185452	-3.274426	0.508561
C	-2.191493	-1.629463	1.230011
O	-2.193126	-2.951014	0.957967
O	-1.153896	-1.060182	1.564948
C	-3.580781	0.441604	1.591656
C	-4.736825	-1.596107	0.922969
C	-5.084661	0.767926	1.451740
H	-3.261928	0.482109	2.649297
C	-5.791538	-0.589047	1.399358
H	-5.262745	1.314116	0.515960
H	-6.679188	-0.619446	0.750818
C	-3.438129	-0.953127	1.038329
O	-5.015111	-2.725687	0.551923
H	-2.943793	1.172342	1.058778
H	-5.446310	1.405509	2.270395

H -6.109685 -0.920856 2.403093

SMDopt/9_TS_SSd

Frequencies, energies and thermodynamic properties:

Lowest Vibrational Mode (1/cm) = -169.5927

2nd Lowest Vibrational Mode (1/cm) = 20.6004

E(RM062X) (a.u.) = -2267.74782113

Thermal correction to Enthalpy (a.u.) = 0.605738

Thermal correction to Gibbs Free Energy (a.u.) = 0.501148

Total Entropy (cal/Kmol) = 220.128

Esp(RM062X+D3) (a.u.) = -2270.00440341

Esp(D3) (a.u.) = -0.0085542199

Esp(RM062X) (a.u.) = -2269.9958491901

Optimised cartesian coordinates (Angstrom):

C	6.627305	-1.499627	0.071026
C	6.408455	-2.641821	-0.713412
C	5.133703	-2.963572	-1.167470
C	4.084717	-2.113616	-0.817116
C	4.303927	-0.982371	-0.029463
H	7.248663	-3.286230	-0.974681
H	4.958225	-3.848924	-1.780348
C	2.042499	-0.791262	-0.390441
C	0.654598	1.070276	0.410283
C	1.668046	1.113745	1.566224
C	3.077022	0.932333	1.034713
H	3.784612	0.787501	1.860632
S	2.391906	-2.271757	-1.244601
N	3.120064	-0.274466	0.203729
N	0.836778	-0.193436	-0.346673
C	1.525102	2.358298	2.472300
C	2.365437	3.560971	2.034252
C	1.864447	1.986694	3.916069
H	0.462458	2.650032	2.441976
H	3.438147	3.369891	2.196019
H	2.221447	3.826135	0.977135
H	2.095979	4.438376	2.640239
H	1.192612	1.203686	4.296901

H	2.899121	1.615157	3.990973
H	1.775730	2.863944	4.573556
H	1.439720	0.230760	2.184176
H	-0.340073	0.992236	0.868883
C	0.693207	2.278160	-0.502922
C	-0.204789	3.327098	-0.274567
C	1.631369	2.394309	-1.534088
C	-0.145379	4.488643	-1.042792
H	-0.956577	3.229072	0.513126
C	1.690296	3.556964	-2.303623
H	2.325226	1.578222	-1.752744
C	0.807993	4.608611	-2.055138
H	-0.849898	5.300391	-0.853850
H	2.428081	3.638192	-3.103647
H	0.855004	5.516616	-2.658721
C	-1.493059	-0.007296	-1.114536
H	-1.453335	1.075858	-1.022185
C	-2.686138	-0.631729	-1.288415
C	-3.839287	0.195358	-1.773785
F	-3.821453	0.245580	-3.117428
F	-5.041085	-0.269235	-1.439770
F	-3.769248	1.463946	-1.349128
O	-0.143107	-1.920832	-1.460305
C	-0.281070	-0.801878	-1.008352
H	3.415273	1.784337	0.426425
C	5.582269	-0.653213	0.425586
H	5.759626	0.236669	1.029125
H	7.637229	-1.265643	0.410050
H	-2.764897	-1.705249	-1.472594
C	-0.151645	-2.436690	1.713281
H	0.141640	-2.901631	0.760441
H	0.684045	-1.858940	2.128332
H	-0.420850	-3.239994	2.414860
C	-2.395532	-2.107792	1.076868
O	-1.229368	-1.537367	1.535463
O	-2.408868	-3.281501	0.758700
C	-3.358401	0.208748	1.676306

C	-4.846531	-1.566763	0.934614
C	-4.789991	0.786801	1.591710
H	-3.019285	0.140563	2.726254
C	-5.713422	-0.429152	1.492517
H	-4.890535	1.400859	0.686746
H	-6.606622	-0.277446	0.868833
C	-3.464520	-1.148540	1.033336
O	-5.315856	-2.625399	0.540753
H	-2.620856	0.859741	1.166280
H	-5.025572	1.435171	2.447401
H	-6.062827	-0.757858	2.486880

SMDopt/9_TS_SSe

Frequencies, energies and thermodynamic properties:

Lowest Vibrational Mode (1/cm) = -147.4069

2nd Lowest Vibrational Mode (1/cm) = 15.5733

E(RM062X) (a.u.) = -2267.74204313

Thermal correction to Enthalpy (a.u.) = 0.605427

Thermal correction to Gibbs Free Energy (a.u.) = 0.498489

Total Entropy (cal/Kmol) = 225.069

Esp(RM062X+D3) (a.u.) = -2270.00065106

Esp(D3) (a.u.) = -0.0082814134

Esp(RM062X) (a.u.) = -2269.9923696466

Optimised cartesian coordinates (Angstrom):

C	6.656651	-2.139171	0.288399
C	6.308263	-3.351897	-0.324755
C	4.998535	-3.602416	-0.720211
C	4.047334	-2.609269	-0.486678
C	4.394708	-1.407040	0.131728
H	7.073997	-4.109177	-0.496869
H	4.722764	-4.542579	-1.200271
C	2.154223	-1.044393	-0.228615
C	0.982470	1.044559	0.302255
C	2.005216	1.135273	1.445871
C	3.382051	0.742233	0.945171
H	4.079328	0.632343	1.785422
S	2.337118	-2.655858	-0.869575

N	3.291067	-0.559218	0.275620
N	1.013097	-0.328939	-0.258239
C	1.995889	2.495478	2.180033
C	2.944344	3.541491	1.588012
C	2.314051	2.285900	3.660821
H	0.965805	2.883532	2.109365
H	3.994959	3.267523	1.773739
H	2.813767	3.678506	0.505091
H	2.770062	4.513099	2.072973
H	1.574642	1.628372	4.140949
H	3.308941	1.829063	3.785676
H	2.317332	3.246523	4.196452
H	1.690790	0.366561	2.171134
H	-0.005858	1.155184	0.766160
C	1.134461	2.096179	-0.776393
C	0.356829	3.257397	-0.699091
C	2.060521	1.961144	-1.815931
C	0.524449	4.283158	-1.627960
H	-0.385241	3.354555	0.098091
C	2.227584	2.987825	-2.746109
H	2.659468	1.052142	-1.915795
C	1.466112	4.152815	-2.649917
H	-0.086587	5.184538	-1.556784
H	2.954936	2.873490	-3.551600
H	1.597815	4.954145	-3.378996
C	-1.323752	-0.039717	-0.972510
H	-1.184066	1.038847	-1.034180
C	-2.567834	-0.575257	-1.074951
C	-3.660760	0.235520	-1.698264
F	-3.542550	0.200547	-3.042249
F	-4.878683	-0.216462	-1.424864
F	-3.602748	1.524839	-1.355086
O	-0.159320	-2.103769	-1.073117
C	-0.182510	-0.922660	-0.787961
H	3.799663	1.469886	0.233676
C	5.709307	-1.149353	0.526281
H	5.988253	-0.206663	0.996708

H 7.691698 -1.964194 0.584516
H -2.718663 -1.657802 -1.080053
C -6.495465 -2.625608 0.221449
H -6.479458 -3.537033 0.837133
H -7.517074 -2.229234 0.179479
H -6.162081 -2.890517 -0.793577
C -4.371701 -1.882492 0.859816
O -5.690180 -1.607747 0.775111
O -3.952359 -3.006101 0.617218
C -2.220884 -1.010513 1.918103
C -3.958378 0.610964 1.423304
C -1.954491 0.275179 2.729255
H -2.234852 -1.911286 2.551821
C -2.741840 1.355192 1.991222
H -0.880895 0.499481 2.826618
H -2.170399 1.743649 1.125514
C -3.540614 -0.764614 1.240133
O -5.016980 1.165282 1.162700
H -1.393222 -1.165385 1.198425
H -2.360499 0.162232 3.746803
H -3.050425 2.213954 2.603355

SMDopt/9_TS_SSf

Frequencies, energies and thermodynamic properties:

Lowest Vibrational Mode (1/cm) = -70.4079

2nd Lowest Vibrational Mode (1/cm) = 20.3431

E(RM062X) (a.u.) = -2267.73863405

Thermal correction to Enthalpy (a.u.) = 0.606192

Thermal correction to Gibbs Free Energy (a.u.) = 0.499059

Total Entropy (cal/Kmol) = 225.481

Esp(RM062X+D3) (a.u.) = -2269.99985163

Esp(D3) (a.u.) = -0.0080510114

Esp(RM062X) (a.u.) = -2269.9918006186

Optimised cartesian coordinates (Angstrom):

C 6.362074 -2.705879 0.187178
C 5.838313 -3.862736 -0.411153
C 4.497260 -3.938431 -0.768775

C	3.693462	-2.827504	-0.510036
C	4.213966	-1.682467	0.094104
H	6.492364	-4.714238	-0.602438
H	4.084587	-4.832828	-1.237380
C	2.033360	-1.029079	-0.199764
C	1.156607	1.197579	0.381718
C	2.243517	1.163153	1.467618
C	3.529224	0.588768	0.907655
H	4.248770	0.397817	1.713499
S	1.990074	-2.637601	-0.865207
N	3.236551	-0.696719	0.265573
N	0.981610	-0.173873	-0.169665
C	2.452238	2.522036	2.174303
C	3.492688	3.427648	1.510175
C	2.825118	2.289363	3.638581
H	1.479613	3.040476	2.153429
H	4.508195	3.021767	1.641931
H	3.318173	3.568672	0.433954
H	3.473809	4.419109	1.985745
H	2.035701	1.738599	4.170512
H	3.758710	1.709331	3.716327
H	2.982423	3.247567	4.154998
H	1.870210	0.451857	2.222597
H	0.225848	1.438457	0.907566
C	1.374863	2.209994	-0.723195
C	0.751258	3.459145	-0.624915
C	2.216498	1.949134	-1.810154
C	0.989818	4.445253	-1.580423
H	0.073814	3.657761	0.209743
C	2.453937	2.936352	-2.767177
H	2.694256	0.972837	-1.927579
C	1.847787	4.187458	-2.650535
H	0.498994	5.415854	-1.491734
H	3.113946	2.723378	-3.609679
H	2.034249	4.957668	-3.400841
C	-1.348113	0.409831	-0.763481
H	-1.151318	1.464096	-0.575301

C	-2.587069	0.008451	-1.080077
C	-3.645121	1.038947	-1.349964
F	-3.497174	1.524202	-2.598414
F	-4.873585	0.556904	-1.274106
F	-3.550038	2.087599	-0.528316
O	-0.374952	-1.714457	-1.144255
C	-0.261742	-0.586368	-0.719460
H	3.999698	1.246066	0.161233
C	5.562483	-1.599780	0.449241
H	5.977355	-0.702123	0.906654
H	7.419558	-2.669511	0.451648
H	-2.833878	-1.033015	-1.300350
C	-4.963442	-3.743617	-0.899472
H	-5.356580	-3.227531	-1.788519
H	-4.305675	-4.563307	-1.214735
H	-5.818140	-4.154455	-0.340801
C	-4.849521	-1.782196	0.411846
O	-4.186567	-2.881675	-0.099949
O	-6.021815	-1.611036	0.129417
C	-2.641942	-1.498625	1.718796
C	-4.329282	0.262136	1.806401
C	-2.037173	-0.317853	2.523025
H	-2.751299	-2.393960	2.359360
C	-3.150528	0.725523	2.684881
H	-1.184277	0.111348	1.976092
H	-2.850347	1.739535	2.373577
C	-3.974493	-0.992140	1.225890
O	-5.350518	0.941099	1.699672
H	-1.971421	-1.809921	0.900410
H	-1.639391	-0.653398	3.491454
H	-3.514527	0.813473	3.722072

SMDopt/19_TS_SRa

Frequencies, energies and thermodynamic properties:

Lowest Vibrational Mode (1/cm) = -119.9580

2nd Lowest Vibrational Mode (1/cm) = 16.5634

E(RM062X) (a.u.) = -2588.54508528

Thermal correction to Enthalpy (a.u.) = 0.662787

Thermal correction to Gibbs Free Energy (a.u.) = 0.547893

Total Entropy (cal/Kmol) = 241.814

Esp(RM062X+D3) (a.u.) = -2591.15471684

Esp(D3) (a.u.) = -0.0108280698

Esp(RM062X) (a.u.) = -2591.1438887702

Optimised cartesian coordinates (Angstrom):

C	-6.997464	-1.861531	-0.560607
C	-6.740234	-3.140731	-0.044646
C	-5.469593	-3.490179	0.399826
C	-4.462946	-2.528378	0.312359
C	-4.719410	-1.260142	-0.210547
H	-7.547745	-3.871595	0.013181
H	-5.265425	-4.481811	0.806236
C	-2.492995	-1.038439	0.311436
C	-1.184105	1.016821	-0.002308
C	-2.163177	1.277408	-1.157651
C	-3.575046	0.906049	-0.748061
H	-4.243994	0.914276	-1.618073
S	-2.788028	-2.686103	0.803400
N	-3.574452	-0.457354	-0.210241
N	-1.319143	-0.389288	0.450591
C	-2.062968	2.711232	-1.728315
C	-3.003802	3.724383	-1.071128
C	-2.292174	2.687700	-3.239041
H	-1.028015	3.046476	-1.553413
H	-4.053606	3.517100	-1.332780
H	-2.921088	3.737206	0.025370
H	-2.769945	4.735004	-1.436884
H	-1.551221	2.053468	-3.748458
H	-3.294060	2.295748	-3.477478
H	-2.221588	3.702226	-3.658795
H	-1.849297	0.576084	-1.947107
H	-0.182563	1.091446	-0.440875
C	-1.284627	1.990001	1.152238
C	-0.413380	3.085389	1.182473
C	-2.251693	1.855468	2.153431

C	-0.531131	4.052771	2.179199
H	0.363132	3.175517	0.416146
C	-2.367480	2.822747	3.152632
H	-2.923376	0.992863	2.165531
C	-1.513951	3.926448	3.162444
H	0.151318	4.904304	2.191852
H	-3.127246	2.710706	3.928009
H	-1.606552	4.682092	3.944462
C	1.008123	-0.297425	1.261189
H	0.978194	0.790241	1.300740
C	2.175058	-0.953087	1.502474
C	3.250778	-0.276622	2.296586
F	2.919046	-0.317757	3.604905
F	4.432479	-0.864430	2.197590
F	3.391223	1.018341	1.995453
O	-0.330795	-2.245162	1.317848
C	-0.203398	-1.070177	1.030341
H	-3.987018	1.577828	0.019749
C	-5.995085	-0.902084	-0.651769
H	-6.202647	0.092456	-1.046121
H	-8.004312	-1.609696	-0.896000
H	2.210408	-2.043821	1.550743
C	5.574429	-3.761955	-0.307499
H	6.149137	-3.667896	-1.239978
H	6.258862	-3.876243	0.540278
H	4.924923	-4.645411	-0.383796
C	3.935992	-2.248277	-1.005201
O	4.816107	-2.591501	-0.054951
O	3.727091	-2.953233	-1.971759
C	4.205077	0.210881	-0.544710
O	5.347787	0.275127	-0.112846
C	3.332169	-0.943935	-0.735978
N	2.193542	-0.523904	-1.496244
C	2.253625	0.875785	-1.671428
C	1.373857	1.765069	-2.307502
C	3.427629	1.357044	-1.079791
C	1.708574	3.120710	-2.306626

H	0.465737	1.415205	-2.789467
C	3.752509	2.710315	-1.083886
C	2.877103	3.602558	-1.698995
H	1.033896	3.822657	-2.802747
H	4.679334	3.044308	-0.612647
H	3.100158	4.670425	-1.720618
C	1.047076	-1.249628	-1.770731
O	0.111063	-0.737390	-2.368493
C	0.979525	-2.695321	-1.354245
H	1.419664	-3.309457	-2.150365
H	1.517906	-2.912887	-0.425827
H	-0.080092	-2.950666	-1.239703

SMDopt/19_TS_SRb

Frequencies, energies and thermodynamic properties:

Lowest Vibrational Mode (1/cm) = -193.5624

2nd Lowest Vibrational Mode (1/cm) = 20.8586

E(RM062X) (a.u.) = -2588.54503130

Thermal correction to Enthalpy (a.u.) = 0.662954

Thermal correction to Gibbs Free Energy (a.u.) = 0.549685

Total Entropy (cal/Kmol) = 238.395

Esp(RM062X+D3) (a.u.) = -2591.15277943

Esp(D3) (a.u.) = -0.0101582670

Esp(RM062X) (a.u.) = -2591.1426211630

Optimised cartesian coordinates (Angstrom):

C	7.333418	-2.072553	0.717428
C	7.066819	-3.302526	0.098916
C	5.803564	-3.586970	-0.410866
C	4.815963	-2.610151	-0.288490
C	5.082398	-1.390242	0.335321
H	7.859372	-4.046904	0.012913
H	5.591381	-4.541765	-0.894423
C	2.878933	-1.074282	-0.245660
C	1.627731	0.994925	0.129225
C	2.531559	1.121617	1.367206
C	3.958096	0.750123	1.008914
H	4.575181	0.666729	1.912792

S	3.146822	-2.700507	-0.823745
N	3.956589	-0.561137	0.356382
N	1.743297	-0.380026	-0.407707
C	2.430600	2.487398	2.082982
C	3.407143	3.547647	1.566327
C	2.623500	2.297167	3.588033
H	1.403176	2.854820	1.918403
H	4.442049	3.297462	1.848574
H	3.372281	3.671575	0.474723
H	3.171120	4.520082	2.022779
H	1.864095	1.621534	4.009079
H	3.615874	1.869133	3.802547
H	2.554513	3.261155	4.113275
H	2.157163	0.354769	2.066242
H	0.594615	1.083050	0.488616
C	1.858583	2.041693	-0.940569
C	1.054599	3.187664	-0.945948
C	2.877246	1.918094	-1.890960
C	1.283177	4.208252	-1.867271
H	0.243113	3.277974	-0.218706
C	3.106003	2.939692	-2.813935
H	3.500759	1.020790	-1.924816
C	2.314704	4.088587	-2.800047
H	0.650214	5.097292	-1.860302
H	3.905147	2.833718	-3.549580
H	2.494367	4.885607	-3.523596
C	-0.512323	-0.166483	-1.358611
H	-0.375053	0.906030	-1.488130
C	-1.762005	-0.736079	-1.415498
C	-2.820284	-0.086220	-2.254703
F	-2.542369	-0.238595	-3.559195
F	-4.034839	-0.610704	-2.071029
F	-2.921363	1.231830	-2.042763
O	0.669732	-2.215651	-1.223415
C	0.597668	-1.019918	-1.016509
H	4.425680	1.478762	0.329812
C	6.348273	-1.098416	0.846063

H	6.562064	-0.141641	1.322305
H	8.332729	-1.870673	1.105232
H	-1.829983	-1.827253	-1.375829
C	-0.856059	0.675665	3.290710
H	-0.073361	1.177775	2.701857
H	-1.228018	1.358215	4.062858
H	-0.430873	-0.223334	3.757485
C	-1.730058	-0.511255	1.462373
O	-1.966731	0.353290	2.468815
O	-0.677060	-1.101249	1.358722
C	-3.478128	-1.971582	0.428648
O	-2.922362	-3.051370	0.298978
C	-2.858632	-0.636951	0.534236
N	-3.932620	0.318961	0.659355
C	-5.164215	-0.354277	0.640638
C	-6.475910	0.136722	0.704430
C	-4.935945	-1.731735	0.501669
C	-7.516406	-0.791148	0.643913
H	-6.674379	1.198541	0.807782
C	-5.982786	-2.647702	0.436236
C	-7.287775	-2.169079	0.513274
H	-8.543746	-0.425733	0.704028
H	-5.766378	-3.712274	0.325407
H	-8.132681	-2.857976	0.470422
C	-3.808166	1.698581	0.689374
O	-4.779822	2.411691	0.846964
C	-2.436242	2.309428	0.516333
H	-2.132880	2.749956	1.476620
H	-1.663550	1.612798	0.176835
H	-2.537172	3.122739	-0.214206

SMDopt/19_TS_SRc

Frequencies, energies and thermodynamic properties:

Lowest Vibrational Mode (1/cm) =	-252.3809
2nd Lowest Vibrational Mode (1/cm) =	15.3415
E(RM062X) (a.u.) =	-2588.54425600
Thermal correction to Enthalpy (a.u.) =	0.663231

Thermal correction to Gibbs Free Energy (a.u.) = 0.547191

Total Entropy (cal/Kmol) = 244.227

Esp(RM062X+D3) (a.u.) = -2591.14842125

Esp(D3) (a.u.) = -0.0100255757

Esp(RM062X) (a.u.) = -2591.1383956743

Optimised cartesian coordinates (Angstrom):

C	-6.637487	2.900736	0.207671
C	-6.134981	3.954491	-0.568374
C	-4.835106	3.917738	-1.065194
C	-4.052785	2.802885	-0.767695
C	-4.552935	1.759527	0.013655
H	-6.769380	4.813651	-0.789670
H	-4.439247	4.734204	-1.671084
C	-2.442493	0.954763	-0.432741
C	-1.585294	-1.210008	0.319265
C	-2.525814	-0.980274	1.513624
C	-3.856006	-0.425381	1.037956
H	-4.462620	-0.096362	1.891690
S	-2.391217	2.499825	-1.250836
N	-3.605537	0.747569	0.197639
N	-1.458951	0.051386	-0.444612
C	-2.681698	-2.217455	2.426451
C	-3.832959	-3.149294	2.038941
C	-2.840556	-1.772764	3.880583
H	-1.739374	-2.784975	2.349325
H	-4.806574	-2.675065	2.240605
H	-3.810166	-3.443981	0.979952
H	-3.785664	-4.066610	2.644048
H	-1.966666	-1.198372	4.221126
H	-3.733330	-1.137798	3.999169
H	-2.958501	-2.642814	4.543224
H	-2.035449	-0.190355	2.105570
H	-0.587302	-1.386958	0.743224
C	-1.973505	-2.364267	-0.582080
C	-1.379747	-3.615046	-0.378742
C	-2.944477	-2.227329	-1.580441
C	-1.772097	-4.718436	-1.135051

H	-0.601012	-3.721504	0.380051
C	-3.337042	-3.330831	-2.339084
H	-3.402834	-1.255387	-1.780954
C	-2.757931	-4.579908	-2.113219
H	-1.301393	-5.688138	-0.963988
H	-4.097431	-3.211396	-3.112835
H	-3.065715	-5.441930	-2.707633
C	0.766484	-0.678784	-1.176130
H	0.519216	-1.715980	-0.966992
C	2.086715	-0.306488	-1.349345
C	3.039868	-1.298907	-1.941774
F	2.617609	-1.716993	-3.144131
F	4.251948	-0.766945	-2.142173
F	3.199768	-2.403990	-1.200708
O	-0.052886	1.514673	-1.493633
C	-0.196621	0.374142	-1.076593
H	-4.435609	-1.157518	0.456125
C	-5.856998	1.788951	0.510804
H	-6.255937	0.969154	1.107887
H	-7.661115	2.947713	0.581484
H	2.296313	0.711836	-1.689284
C	1.973719	-3.077027	2.145228
H	2.176975	-2.834174	3.196794
H	0.967586	-3.498727	2.048827
H	2.719578	-3.805729	1.797276
C	3.125350	-1.189501	1.400322
O	2.001209	-1.907567	1.339576
O	4.065644	-1.510571	2.090270
C	1.924885	0.938109	0.977985
O	0.792083	0.611532	1.311318
C	3.028320	0.047548	0.591980
N	4.200237	0.870412	0.428577
C	3.809352	2.225163	0.491564
C	4.537806	3.407663	0.276928
C	2.445200	2.302418	0.809323
C	3.860941	4.619373	0.405372
H	5.592503	3.384574	0.027933

C 1.777900 3.519982 0.936078
C 2.496956 4.690925 0.731049
H 4.419140 5.544351 0.246294
H 0.713296 3.529397 1.177736
H 2.009225 5.662993 0.817661
C 5.517022 0.490222 0.170825
O 6.378199 1.338501 0.049082
C 5.903785 -0.964526 0.071658
H 6.390842 -1.239735 1.016778
H 5.090955 -1.665654 -0.107801
H 6.644384 -1.033141 -0.734976

SMDopt/19_TS_SRd

Frequencies, energies and thermodynamic properties:

Lowest Vibrational Mode (1/cm) = -264.7702

2nd Lowest Vibrational Mode (1/cm) = 15.4407

E(RM062X) (a.u.) = -2588.54572600

Thermal correction to Enthalpy (a.u.) = 0.662901

Thermal correction to Gibbs Free Energy (a.u.) = 0.547592

Total Entropy (cal/Kmol) = 242.689

Esp(RM062X+D3) (a.u.) = -2591.14814177

Esp(D3) (a.u.) = -0.0098471990

Esp(RM062X) (a.u.) = -2591.1382945710

Optimised cartesian coordinates (Angstrom):

C -7.013090 2.372894 0.376105
C -6.691320 3.437632 -0.476652
C -5.428697 3.531711 -1.055739
C -4.499151 2.535125 -0.762699
C -4.819671 1.480264 0.094617
H -7.438381 4.202336 -0.692618
H -5.173653 4.358152 -1.720865
C -2.657270 0.912080 -0.454045
C -1.504233 -1.103519 0.317197
C -2.377980 -0.918448 1.568415
C -3.795574 -0.547970 1.170289
H -4.378463 -0.245854 2.050145
S -2.840604 2.415007 -1.329659

N	-3.746386	0.599505	0.260460
N	-1.577899	0.130093	-0.501148
C	-2.319415	-2.111712	2.548185
C	-3.371284	-3.194392	2.292936
C	-2.430968	-1.608225	3.987479
H	-1.321862	-2.565983	2.428284
H	-4.379778	-2.830324	2.546022
H	-3.386039	-3.542230	1.250077
H	-3.168907	-4.064790	2.934385
H	-1.612165	-0.916425	4.233660
H	-3.384197	-1.077717	4.143360
H	-2.395507	-2.447619	4.697619
H	-1.943544	-0.046194	2.083491
H	-0.463891	-1.152959	0.666186
C	-1.817540	-2.342239	-0.494695
C	-1.039863	-3.488010	-0.286342
C	-2.879723	-2.394021	-1.403728
C	-1.346099	-4.678804	-0.943869
H	-0.181951	-3.430656	0.388771
C	-3.183485	-3.585165	-2.064728
H	-3.479264	-1.503671	-1.610758
C	-2.424897	-4.732149	-1.828239
H	-0.735310	-5.566794	-0.771068
H	-4.016475	-3.615110	-2.769391
H	-2.665292	-5.663120	-2.344792
C	0.633361	-0.400692	-1.388270
H	0.434480	-1.459845	-1.247893
C	1.941339	0.027481	-1.531134
C	2.890858	-0.855450	-2.279152
F	2.518541	-0.972406	-3.562634
F	4.137093	-0.367161	-2.304080
F	2.951066	-2.101061	-1.792446
O	-0.368357	1.738669	-1.592070
C	-0.387471	0.582196	-1.199701
H	-4.321563	-1.373783	0.668325
C	-6.084981	1.379544	0.675069
H	-6.342075	0.550104	1.333600

H	-8.010238	2.316383	0.814605
H	2.121076	1.084098	-1.752751
C	3.692558	-2.806918	2.509788
H	3.644325	-3.604168	1.754374
H	4.613273	-2.900495	3.095184
H	2.818229	-2.885306	3.169911
C	2.720046	-1.221838	1.080575
O	3.742659	-1.529295	1.887223
O	1.732891	-1.913675	0.987681
C	1.882822	1.069811	0.877948
O	0.712979	0.841832	1.160051
C	2.878118	0.092037	0.417984
N	4.150653	0.766392	0.385129
C	3.932330	2.144380	0.588494
C	4.814815	3.235142	0.528077
C	2.575698	2.365757	0.875183
C	4.293786	4.505174	0.771519
H	5.867665	3.098300	0.308251
C	2.066029	3.640397	1.119453
C	2.938136	4.721117	1.065258
H	4.972081	5.360057	0.732601
H	1.003611	3.765930	1.337516
H	2.576196	5.734155	1.246648
C	5.414985	0.238364	0.130086
O	6.389085	0.963418	0.138278
C	5.598849	-1.237239	-0.133480
H	6.063436	-1.678901	0.758980
H	4.696720	-1.800268	-0.374808
H	6.312094	-1.317160	-0.963579

SMDopt/19_TS_SRe

Frequencies, energies and thermodynamic properties:

Lowest Vibrational Mode (1/cm) = -263.9181

2nd Lowest Vibrational Mode (1/cm) = 13.7755

E(RM062X) (a.u.) = -2588.54082071

Thermal correction to Enthalpy (a.u.) = 0.662656

Thermal correction to Gibbs Free Energy (a.u.) = 0.547101

Total Entropy (cal/Kmol) = 243.206

Esp(RM062X+D3) (a.u.) = -2591.14730940

Esp(D3) (a.u.) = -0.0105640750

Esp(RM062X) (a.u.) = -2591.1367453250

Optimised cartesian coordinates (Angstrom):

C	6.493628	-2.786333	-0.131564
C	5.903337	-3.957674	-0.628337
C	4.542107	-4.005842	-0.911145
C	3.785280	-2.856949	-0.680414
C	4.374374	-1.696149	-0.177289
H	6.518387	-4.841923	-0.799830
H	4.078221	-4.912178	-1.303085
C	2.195831	-0.986094	-0.398379
C	1.395429	1.269162	0.158503
C	2.576372	1.273097	1.144801
C	3.800210	0.649317	0.503362
H	4.593207	0.504185	1.247365
S	2.069465	-2.631775	-0.966216
N	3.438948	-0.669134	-0.019463
N	1.179870	-0.106683	-0.351725
C	2.869486	2.658092	1.764676
C	3.820125	3.534979	0.942968
C	3.433244	2.490093	3.176420
H	1.899028	3.176312	1.847785
H	4.846089	3.135168	0.976924
H	3.526216	3.628697	-0.111500
H	3.851745	4.546486	1.373944
H	2.734140	1.952440	3.832612
H	4.380413	1.927643	3.155279
H	3.639595	3.471406	3.628368
H	2.265608	0.605552	1.965611
H	0.507805	1.512016	0.755603
C	1.507842	2.266349	-0.975599
C	0.889888	3.514588	-0.840567
C	2.254953	1.994078	-2.125933
C	1.046795	4.490760	-1.822831
H	0.282007	3.719522	0.045106

C	2.411315	2.970881	-3.110418
H	2.719980	1.014960	-2.267922
C	1.815280	4.222933	-2.957164
H	0.562468	5.461680	-1.705223
H	2.999325	2.749410	-4.002738
H	1.938485	4.985577	-3.728100
C	-1.150874	0.489294	-0.884718
H	-0.985394	1.519724	-0.584780
C	-2.416561	0.062057	-1.231686
C	-3.410461	1.075055	-1.708563
F	-3.167179	1.396574	-2.993092
F	-4.669826	0.631317	-1.672729
F	-3.358065	2.217483	-1.019643
O	-0.218019	-1.641482	-1.289951
C	-0.109478	-0.496349	-0.873886
H	4.199787	1.255437	-0.323618
C	5.741922	-1.639090	0.100341
H	6.209657	-0.730220	0.477705
H	7.564297	-2.769262	0.076105
H	-2.524821	-0.913888	-1.709322
C	-6.607999	-2.044486	-0.683879
H	-6.544151	-3.141217	-0.703132
H	-7.571885	-1.733050	-0.267603
H	-6.500312	-1.664189	-1.709528
C	-4.349247	-1.679783	-0.223857
O	-5.610640	-1.490325	0.161075
O	-4.065373	-2.404098	-1.154902
C	-3.818939	0.400388	1.221179
O	-4.853499	1.043898	1.124577
C	-3.408692	-0.838905	0.546589
N	-2.187311	-1.295388	1.148901
C	-1.676929	-0.241902	1.930742
C	-0.476926	-0.131471	2.655937
C	-2.624985	0.789936	1.995710
C	-0.263709	1.039526	3.380536
H	0.266152	-0.921910	2.644202
C	-2.392269	1.962769	2.711962

C -1.192285 2.093491 3.403117
H 0.666977 1.138056 3.943417
H -3.153034 2.746282 2.722896
H -0.974122 2.998673 3.971967
C -1.585056 -2.554693 1.148542
O -0.442165 -2.679111 1.537520
C -2.392570 -3.748891 0.723569
H -3.416370 -3.716834 1.118131
H -2.449734 -3.796839 -0.369954
H -1.871177 -4.633720 1.105067

SMDopt/19_TS_SRf

Frequencies, energies and thermodynamic properties:

Lowest Vibrational Mode (1/cm) = -227.1419

2nd Lowest Vibrational Mode (1/cm) = 12.6648

E(RM062X) (a.u.) = -2588.54192707

Thermal correction to Enthalpy (a.u.) = 0.662665

Thermal correction to Gibbs Free Energy (a.u.) = 0.546685

Total Entropy (cal/Kmol) = 244.099

Esp(RM062X+D3) (a.u.) = -2591.14642658

Esp(D3) (a.u.) = -0.0102537526

Esp(RM062X) (a.u.) = -2591.1361728274

Optimised cartesian coordinates (Angstrom):

C -7.258365 -1.604067 -0.968264
C -7.163176 -2.782967 -0.214539
C -5.983790 -3.116583 0.444044
C -4.903481 -2.242088 0.330142
C -4.998216 -1.074235 -0.428717
H -8.024820 -3.447466 -0.140001
H -5.905588 -4.031216 1.033244
C -2.839320 -0.883474 0.338263
C -1.377175 1.030280 -0.102846
C -2.141075 1.103552 -1.433667
C -3.623318 0.877259 -1.204057
H -4.146386 0.731896 -2.158281
S -3.306530 -2.413747 1.036602
N -3.803092 -0.347840 -0.417759

N	-1.655311	-0.281469	0.533824
C	-1.840931	2.385302	-2.243080
C	-2.770047	3.562508	-1.935579
C	-1.874271	2.074362	-3.739713
H	-0.809845	2.679703	-1.985028
H	-3.786092	3.370585	-2.315698
H	-2.843793	3.783849	-0.861200
H	-2.399210	4.467022	-2.439799
H	-1.125941	1.314037	-4.006440
H	-2.865942	1.698711	-4.039458
H	-1.667460	2.979559	-4.329565
H	-1.760213	0.250673	-2.019554
H	-0.311375	1.017488	-0.366993
C	-1.635927	2.180209	0.848855
C	-0.729729	3.247747	0.868204
C	-2.771884	2.233047	1.664730
C	-0.977275	4.368295	1.660827
H	0.177608	3.182578	0.262145
C	-3.016730	3.353709	2.459290
H	-3.479982	1.400692	1.693268
C	-2.125696	4.427512	2.451675
H	-0.266176	5.196439	1.665876
H	-3.907951	3.385411	3.088480
H	-2.320173	5.303978	3.072369
C	0.566650	-0.184936	1.545410
H	0.527683	0.901713	1.536114
C	1.788492	-0.803314	1.603787
C	2.903205	-0.074849	2.301179
F	2.721510	-0.138842	3.631862
F	4.114810	-0.587768	2.071980
F	2.959302	1.220830	1.987557
O	-0.831211	-2.107058	1.624947
C	-0.620661	-0.958450	1.282257
H	-4.100595	1.709030	-0.664944
C	-6.180829	-0.732577	-1.087692
H	-6.262144	0.185524	-1.669099
H	-8.195193	-1.360915	-1.471257

H	1.864597	-1.895086	1.637047
C	-0.242699	-2.702459	-1.444843
H	-0.004181	-2.977258	-2.483102
H	-0.630983	-3.574201	-0.907880
H	-0.998511	-1.903792	-1.466788
C	1.507693	-1.161737	-1.180923
O	0.922226	-2.290792	-0.754081
O	1.013891	-0.471723	-2.048124
C	3.692395	-2.003056	-0.227138
O	3.469251	-3.140661	0.163106
C	2.750473	-0.903573	-0.455512
N	3.513432	0.249016	-0.869455
C	4.879165	-0.093249	-0.922891
C	6.026382	0.654717	-1.229293
C	5.023307	-1.440227	-0.548806
C	7.265087	0.013141	-1.177560
H	5.996131	1.702157	-1.510037
C	6.263225	-2.068498	-0.490667
C	7.399246	-1.333911	-0.817350
H	8.156403	0.591410	-1.428959
H	6.320368	-3.116637	-0.189498
H	8.387564	-1.794798	-0.788552
C	2.934347	1.506829	-0.957007
O	1.812043	1.703871	-0.535093
C	3.727270	2.615853	-1.597156
H	4.480570	2.999014	-0.893630
H	4.230633	2.288790	-2.515190
H	3.026030	3.425606	-1.826307

SMDopt/19_TS_SRg

Frequencies, energies and thermodynamic properties:

Lowest Vibrational Mode (1/cm) =	-160.1619
2nd Lowest Vibrational Mode (1/cm) =	18.2142
E(RM062X) (a.u.) =	-2588.54058228
Thermal correction to Enthalpy (a.u.) =	0.662743
Thermal correction to Gibbs Free Energy (a.u.) =	0.548653
Total Entropy (cal/Kmol) =	240.121

Esp(RM062X+D3) (a.u.) = -2591.14820184

Esp(D3) (a.u.) = -0.0101543283

Esp(RM062X) (a.u.) = -2591.1380475117

Optimised cartesian coordinates (Angstrom):

C	7.419676	-1.711339	0.715460
C	7.240572	-2.961827	0.106513
C	6.001878	-3.336721	-0.404744
C	4.948480	-2.429862	-0.292761
C	5.128284	-1.189080	0.320863
H	8.082582	-3.650791	0.029542
H	5.858212	-4.307552	-0.880994
C	2.910485	-1.030484	-0.267228
C	1.504350	0.945695	0.102799
C	2.409908	1.150589	1.330607
C	3.857848	0.876608	0.968800
H	4.481820	0.847703	1.871248
S	3.291842	-2.638064	-0.833749
N	3.948488	-0.438589	0.331043
N	1.727991	-0.419744	-0.432446
C	2.223251	2.515187	2.032259
C	3.093801	3.645133	1.473912
C	2.483597	2.366979	3.532345
H	1.164648	2.796063	1.896181
H	4.153713	3.484789	1.727408
H	3.016586	3.754629	0.383235
H	2.792028	4.599709	1.929434
H	1.807614	1.630690	3.991433
H	3.518821	2.038347	3.717116
H	2.342651	3.329126	4.046389
H	2.098021	0.368398	2.042926
H	0.466248	0.955213	0.465454
C	1.648695	2.003040	-0.971831
C	0.727385	3.057021	-1.000751
C	2.697832	1.983158	-1.896764
C	0.879667	4.099403	-1.914225
H	-0.121733	3.041657	-0.311251
C	2.847689	3.026190	-2.811665

H	3.408166	1.152285	-1.917429
C	1.946298	4.091217	-2.814798
H	0.158099	4.918271	-1.927894
H	3.672152	3.003941	-3.526433
H	2.065930	4.906721	-3.530238
C	-0.525202	-0.348397	-1.397270
H	-0.459360	0.731537	-1.512373
C	-1.739334	-0.962529	-1.450766
C	-2.832734	-0.311123	-2.243967
F	-2.605070	-0.477185	-3.559215
F	-4.042041	-0.827225	-2.014567
F	-2.918501	1.007438	-2.042571
O	0.792830	-2.315590	-1.283526
C	0.640368	-1.133499	-1.053949
H	4.271630	1.629348	0.281433
C	6.369503	-0.806475	0.832345
H	6.514589	0.167186	1.299812
H	8.401267	-1.437683	1.104438
H	-1.816213	-2.050865	-1.368462
C	-0.676914	0.306411	3.361123
H	-0.030082	0.971110	2.769760
H	-1.029583	0.842150	4.249870
H	-0.106533	-0.582629	3.663536
C	-1.615927	-0.776893	1.504176
O	-1.825768	-0.039582	2.612077
O	-0.564706	-1.347191	1.305990
C	-3.565484	-2.047887	0.519667
O	-3.166754	-3.204622	0.407919
C	-2.796274	-0.823868	0.637903
N	-3.710208	0.281000	0.737703
C	-5.027774	-0.202785	0.684539
C	-6.271032	0.444999	0.710090
C	-4.981492	-1.604917	0.560173
C	-7.422995	-0.340365	0.635047
H	-6.376068	1.521768	0.798385
C	-6.136461	-2.375973	0.478247
C	-7.372463	-1.735789	0.521429

H	-8.393110	0.159339	0.669474
H	-6.053384	-3.460519	0.380101
H	-8.297728	-2.311020	0.464716
C	-3.217223	1.573243	0.675126
O	-2.017868	1.764489	0.573940
C	-4.195615	2.713233	0.730293
H	-4.856378	2.693870	-0.148064
H	-4.814073	2.665124	1.636508
H	-3.621145	3.645405	0.728598

SMDopt/19_TS_SRh

Frequencies, energies and thermodynamic properties:

Lowest Vibrational Mode (1/cm) = -178.5570

2nd Lowest Vibrational Mode (1/cm) = 17.5755

E(RM062X) (a.u.) = -2588.53806246

Thermal correction to Enthalpy (a.u.) = 0.662914

Thermal correction to Gibbs Free Energy (a.u.) = 0.548144

Total Entropy (cal/Kmol) = 241.555

Esp(RM062X+D3) (a.u.) = -2591.14743648

Esp(D3) (a.u.) = -0.0107052412

Esp(RM062X) (a.u.) = -2591.1367312388

Optimised cartesian coordinates (Angstrom):

C	6.497605	-2.798803	-0.291247
C	5.898143	-3.962496	-0.795535
C	4.530229	-4.011599	-1.042487
C	3.777744	-2.869537	-0.768493
C	4.375258	-1.716599	-0.257835
H	6.511553	-4.840624	-1.000791
H	4.058991	-4.912115	-1.438788
C	2.190816	-1.009683	-0.400224
C	1.405734	1.236225	0.212142
C	2.598631	1.210361	1.182263
C	3.813540	0.606511	0.505826
H	4.615811	0.436149	1.234567
S	2.053531	-2.646457	-0.993896
N	3.441165	-0.696355	-0.050791
N	1.171775	-0.132756	-0.313753

C	2.894315	2.579017	1.836955
C	3.865459	3.461610	1.047001
C	3.428745	2.373541	3.254742
H	1.928277	3.105853	1.914426
H	4.886619	3.050160	1.086096
H	3.585309	3.578345	-0.009211
H	3.901393	4.464217	1.497777
H	2.700796	1.846554	3.888246
H	4.358808	1.782754	3.240616
H	3.654896	3.341033	3.726495
H	2.301009	0.517374	1.985654
H	0.525948	1.478090	0.822796
C	1.524328	2.250002	-0.906949
C	0.932147	3.507613	-0.744266
C	2.252620	1.982668	-2.070315
C	1.095348	4.495989	-1.713089
H	0.337377	3.709507	0.151003
C	2.415279	2.971997	-3.041223
H	2.697872	0.998064	-2.234955
C	1.844698	4.232081	-2.861021
H	0.630531	5.473564	-1.574193
H	2.988174	2.753510	-3.944012
H	1.972528	5.004400	-3.621462
C	-1.136681	0.517721	-0.863068
H	-0.914517	1.548480	-0.603725
C	-2.398839	0.178277	-1.257122
C	-3.318186	1.256190	-1.743911
F	-3.071061	1.511912	-3.044195
F	-4.607023	0.928067	-1.673042
F	-3.149071	2.410222	-1.096013
O	-0.284273	-1.673514	-1.147672
C	-0.130115	-0.515904	-0.795560
H	4.204401	1.234379	-0.308683
C	5.749335	-1.659282	-0.015819
H	6.223175	-0.756371	0.368093
H	7.573324	-2.782279	-0.111751
H	-2.605928	-0.817122	-1.649499

C	-5.260446	-2.897261	-1.700782
H	-6.053475	-2.300302	-2.172702
H	-4.737945	-3.487919	-2.461467
H	-5.714544	-3.566069	-0.955720
C	-4.721211	-1.309524	-0.068718
O	-4.287774	-2.065912	-1.095970
O	-5.896188	-1.182382	0.175240
C	-3.772882	0.679854	1.268725
O	-4.667445	1.519516	1.182583
C	-3.632918	-0.612523	0.638861
N	-2.479205	-1.282684	1.156034
C	-1.755246	-0.353179	1.925463
C	-0.533258	-0.454898	2.613802
C	-2.497721	0.840407	2.005276
C	-0.092197	0.653917	3.333683
H	0.079950	-1.351316	2.604929
C	-2.027490	1.951796	2.703147
C	-0.810170	1.860028	3.371497
H	0.850631	0.574900	3.878442
H	-2.629701	2.862883	2.724598
H	-0.418238	2.708642	3.934962
C	-2.424179	-2.674061	1.279439
O	-3.409693	-3.350581	1.083367
C	-1.102005	-3.294352	1.644259
H	-0.271986	-2.832677	1.095282
H	-0.920181	-3.201655	2.724825
H	-1.164685	-4.359968	1.396427

SMDopt/19_TS_SRi

Frequencies, energies and thermodynamic properties:

Lowest Vibrational Mode (1/cm) = -204.7138

2nd Lowest Vibrational Mode (1/cm) = 15.6928

E(RM062X) (a.u.) = -2588.53811167

Thermal correction to Enthalpy (a.u.) = 0.662829

Thermal correction to Gibbs Free Energy (a.u.) = 0.548170

Total Entropy (cal/Kmol) = 241.320

Esp(RM062X+D3) (a.u.) = -2591.14698976

Esp(D3) (a.u.) = -0.0106457719

Esp(RM062X) (a.u.) = -2591.1363439881

Optimised cartesian coordinates (Angstrom):

C	6.636717	-2.610094	-0.380026
C	6.084327	-3.768874	-0.945256
C	4.720800	-3.855427	-1.207144
C	3.924463	-2.756080	-0.886687
C	4.475860	-1.607494	-0.317611
H	6.730963	-4.613431	-1.186311
H	4.286277	-4.752399	-1.650656
C	2.268121	-0.972260	-0.449022
C	1.398150	1.213034	0.253458
C	2.583218	1.187523	1.233388
C	3.825011	0.657704	0.543252
H	4.626649	0.485464	1.272206
S	2.193742	-2.586146	-1.114778
N	3.504476	-0.631186	-0.074064
N	1.219218	-0.137432	-0.335920
C	2.823601	2.535631	1.950194
C	3.766873	3.488302	1.209393
C	3.355651	2.287076	3.361882
H	1.838224	3.022710	2.042453
H	4.801881	3.112056	1.237103
H	3.489342	3.644442	0.157663
H	3.764003	4.469235	1.706891
H	2.645580	1.701883	3.963702
H	4.309326	1.736032	3.328716
H	3.538440	3.240097	3.879700
H	2.303956	0.449511	2.002829
H	0.503988	1.394172	0.864650
C	1.488727	2.282291	-0.815888
C	0.847374	3.507657	-0.600938
C	2.236965	2.098193	-1.982697
C	0.979816	4.545874	-1.521100
H	0.237489	3.644144	0.296612
C	2.368766	3.137472	-2.905038
H	2.722439	1.140702	-2.188527

C	1.747850	4.364788	-2.672719
H	0.476325	5.497363	-1.341555
H	2.957192	2.983820	-3.811196
H	1.851599	5.176017	-3.395385
C	-1.102963	0.449737	-0.880898
H	-0.909987	1.479633	-0.597796
C	-2.367675	0.082910	-1.267317
C	-3.290485	1.160482	-1.755017
F	-3.031393	1.431170	-3.048849
F	-4.581290	0.829922	-1.700540
F	-3.135726	2.308584	-1.092113
O	-0.175538	-1.700373	-1.236365
C	-0.069392	-0.548411	-0.844056
H	4.199849	1.335278	-0.238305
C	5.844608	-1.512838	-0.058663
H	6.280885	-0.612385	0.372716
H	7.709569	-2.563173	-0.189027
H	-2.542535	-0.901315	-1.706090
C	-6.789939	-1.561978	-0.882403
H	-6.848415	-2.658892	-0.919464
H	-7.734099	-1.151547	-0.507795
H	-6.593452	-1.185542	-1.896607
C	-4.529818	-1.468753	-0.315656
O	-5.780991	-1.134230	0.016412
O	-4.282730	-2.192497	-1.254528
C	-3.731881	0.486414	1.224367
O	-4.639618	1.308491	1.156376
C	-3.538438	-0.774112	0.524957
N	-2.387288	-1.439296	1.050248
C	-1.704670	-0.528003	1.875685
C	-0.493618	-0.632512	2.582584
C	-2.478133	0.641080	1.997526
C	-0.094488	0.452108	3.361213
H	0.141865	-1.512652	2.543144
C	-2.050964	1.729749	2.756099
C	-0.843786	1.636896	3.442367
H	0.840101	0.371189	3.919795

H -2.676719 2.623469 2.808796
H -0.483772 2.467383 4.052110
C -2.308914 -2.836156 1.140472
O -3.286863 -3.521433 0.952625
C -0.967889 -3.440260 1.462769
H -0.150635 -2.919016 0.950647
H -0.792372 -3.418856 2.548317
H -1.000142 -4.488416 1.143789

SMDopt/19_TS_SRj

Frequencies, energies and thermodynamic properties:

Lowest Vibrational Mode (1/cm) = -223.4471

2nd Lowest Vibrational Mode (1/cm) = 16.9051

E(RM062X) (a.u.) = -2588.53851927

Thermal correction to Enthalpy (a.u.) = 0.662842

Thermal correction to Gibbs Free Energy (a.u.) = 0.546802

Total Entropy (cal/Kmol) = 244.225

Esp(RM062X+D3) (a.u.) = -2591.14422452

Esp(D3) (a.u.) = -0.0099610310

Esp(RM062X) (a.u.) = -2591.1342634890

Optimised cartesian coordinates (Angstrom):

C -6.592374 2.946278 0.207460
C -6.084687 3.987071 -0.582505
C -4.788330 3.932775 -1.086941
C -4.014743 2.813586 -0.782987
C -4.519961 1.783301 0.012418
H -6.712205 4.849983 -0.808768
H -4.388762 4.739294 -1.703591
C -2.420339 0.953008 -0.438609
C -1.583065 -1.214228 0.327264
C -2.508170 -0.959153 1.528190
C -3.836706 -0.395041 1.058930
H -4.431496 -0.048289 1.914011
S -2.358737 2.489635 -1.272176
N -3.581272 0.764466 0.201757
N -1.447595 0.038565 -0.449373
C -2.669078 -2.182117 2.458827

C	-3.832680	-3.107427	2.093205
C	-2.811898	-1.715989	3.907917
H	-1.733017	-2.759914	2.382414
H	-4.799879	-2.621385	2.297610
H	-3.822239	-3.415156	1.037751
H	-3.788857	-4.017727	2.709089
H	-1.929631	-1.145790	4.233535
H	-3.697363	-1.070549	4.024814
H	-2.933101	-2.575620	4.583461
H	-2.000821	-0.167849	2.103800
H	-0.583789	-1.398155	0.744377
C	-1.994322	-2.371418	-0.559689
C	-1.413309	-3.627161	-0.350369
C	-2.972737	-2.232176	-1.550447
C	-1.825080	-4.732728	-1.093056
H	-0.629144	-3.734902	0.402486
C	-3.384816	-3.337813	-2.295514
H	-3.421118	-1.256656	-1.755893
C	-2.818151	-4.591488	-2.063496
H	-1.363984	-5.706246	-0.917519
H	-4.150729	-3.216558	-3.063521
H	-3.141262	-5.455117	-2.647354
C	0.770595	-0.721297	-1.183499
H	0.508441	-1.755639	-0.976711
C	2.087526	-0.368267	-1.378031
C	3.043673	-1.379834	-1.949476
F	2.524329	-1.934422	-3.064110
F	4.188597	-0.810337	-2.329347
F	3.330742	-2.399963	-1.141128
O	-0.025776	1.479883	-1.507719
C	-0.182550	0.345326	-1.081639
H	-4.429965	-1.127815	0.491710
C	-5.820523	1.830300	0.517295
H	-6.223262	1.020498	1.125414
H	-7.613167	3.006914	0.587045
H	2.310720	0.647771	-1.713359
C	2.114796	-3.181903	2.043782

H	2.235451	-2.969563	3.115135
H	1.160080	-3.690406	1.871526
H	2.943410	-3.826189	1.716535
C	3.164578	-1.203295	1.398443
O	2.080639	-1.983514	1.286874
O	4.089723	-1.480892	2.121805
C	1.898344	0.906088	0.979538
O	0.761610	0.558375	1.299360
C	3.017971	0.048770	0.619734
N	4.168637	0.886661	0.452026
C	3.761614	2.230190	0.489942
C	4.449788	3.427683	0.229089
C	2.390970	2.281732	0.803256
C	3.747332	4.625881	0.337479
H	5.496589	3.458116	-0.055841
C	1.697631	3.487027	0.907896
C	2.385868	4.671527	0.678383
H	4.281440	5.558180	0.143767
H	0.633060	3.475564	1.150001
H	1.875387	5.633113	0.748298
C	5.438079	0.359710	0.191888
O	5.573902	-0.810860	-0.070886
C	6.621018	1.289625	0.284708
H	6.714851	1.881481	-0.637091
H	6.550704	1.972302	1.140431
H	7.513669	0.661598	0.379845

SMDopt/19_TS_SRk

Frequencies, energies and thermodynamic properties:

Lowest Vibrational Mode (1/cm) = -357.7970

2nd Lowest Vibrational Mode (1/cm) = 13.7217

E(RM062X) (a.u.) = -2588.53394785

Thermal correction to Enthalpy (a.u.) = 0.662380

Thermal correction to Gibbs Free Energy (a.u.) = 0.546341

Total Entropy (cal/Kmol) = 244.226

Esp(RM062X+D3) (a.u.) = -2591.13703547

Esp(D3) (a.u.) = -0.0098557117

Esp(RM062X) (a.u.) = -2591.1271797583

Optimised cartesian coordinates (Angstrom):

C	7.042605	-2.322095	0.189957
C	6.676825	-3.408312	-0.616288
C	5.379653	-3.526034	-1.108771
C	4.460989	-2.531750	-0.777069
C	4.825809	-1.455457	0.034803
H	7.415865	-4.170973	-0.864673
H	5.089729	-4.369333	-1.737461
C	2.625872	-0.917961	-0.384655
C	1.499833	1.099187	0.418936
C	2.456809	0.948156	1.613907
C	3.850377	0.585756	1.131634
H	4.492068	0.308019	1.978037
S	2.766751	-2.441424	-1.234759
N	3.756482	-0.580989	0.251224
N	1.540478	-0.145863	-0.384416
C	2.449334	2.157396	2.575831
C	3.465074	3.249868	2.230992
C	2.669648	1.680357	4.011753
H	1.439563	2.596426	2.520065
H	4.493919	2.903259	2.418035
H	3.401919	3.581780	1.184783
H	3.295595	4.127316	2.872379
H	1.881158	0.981297	4.326662
H	3.639785	1.166747	4.109022
H	2.670940	2.531506	4.708666
H	2.069305	0.080740	2.172876
H	0.482567	1.135873	0.832795
C	1.736444	2.333565	-0.424705
C	0.948930	3.465299	-0.178775
C	2.736790	2.396432	-1.400248
C	1.187151	4.654738	-0.865925
H	0.135212	3.396663	0.548642
C	2.973024	3.586618	-2.090327
H	3.339375	1.515362	-1.636027
C	2.207049	4.720461	-1.817117

H	0.569531	5.531774	-0.663487
H	3.758686	3.626229	-2.846989
H	2.394864	5.650520	-2.356588
C	-0.690924	0.357394	-1.230554
H	-0.474159	1.417845	-1.133323
C	-2.022411	-0.058535	-1.325522
C	-2.927358	0.802532	-2.170028
F	-2.713991	0.511724	-3.466927
F	-4.230036	0.620686	-1.965670
F	-2.673654	2.110908	-2.038113
O	0.273727	-1.804178	-1.333965
C	0.314967	-0.622040	-1.017914
H	4.333278	1.407229	0.581567
C	6.125498	-1.330567	0.527913
H	6.417987	-0.484227	1.149180
H	8.065708	-2.246468	0.560587
H	-2.207816	-1.118370	-1.532222
C	-3.733260	3.474886	0.453989
H	-2.862067	3.845735	-0.103751
H	-4.654273	3.874116	0.016056
H	-3.650117	3.792774	1.502614
C	-2.784914	1.368702	0.819279
O	-3.827822	2.063450	0.361682
O	-1.793810	1.899505	1.271825
C	-1.874653	-1.021685	1.083121
O	-0.728087	-0.788473	1.432101
C	-2.880588	-0.080526	0.570144
N	-4.124842	-0.785616	0.429008
C	-3.819643	-2.170874	0.466135
C	-4.613415	-3.283076	0.160117
C	-2.490662	-2.351453	0.879977
C	-4.042548	-4.547997	0.298672
H	-5.636682	-3.190320	-0.195930
C	-1.926389	-3.619166	1.008641
C	-2.715671	-4.727041	0.719300
H	-4.649021	-5.422970	0.057070
H	-0.884121	-3.718673	1.318005

H -2.306110 -5.734636 0.803321
C -5.296734 -0.331059 1.091108
O -5.239584 0.507408 1.953542
C -6.594147 -0.953217 0.649955
H -6.592688 -1.181186 -0.422375
H -6.774877 -1.880573 1.213742
H -7.400404 -0.250006 0.889466

SMDopt/19_TS_SRl

Frequencies, energies and thermodynamic properties:

Lowest Vibrational Mode (1/cm) =	-381.1376
2nd Lowest Vibrational Mode (1/cm) =	18.4780
E(RM062X) (a.u.) =	-2588.53087598
Thermal correction to Enthalpy (a.u.) =	0.662320
Thermal correction to Gibbs Free Energy (a.u.) =	0.548078
Total Entropy (cal/Kmol) =	240.441
Esp(RM062X+D3) (a.u.) =	-2591.13495821
Esp(D3) (a.u.) =	-0.0104546268
Esp(RM062X) (a.u.) =	-2591.1245035832

Optimised cartesian coordinates (Angstrom):

C -7.379297 -1.022159 -0.475881
C -7.293871 -2.287677 0.120822
C -6.074299 -2.781920 0.575371
C -4.945191 -1.979366 0.420159
C -5.032385 -0.723314 -0.183095
H -8.193528 -2.893784 0.234054
H -6.002745 -3.765636 1.041418
C -2.784173 -0.781140 0.306517
C -1.236861 1.056875 -0.101890
C -2.142451 1.341197 -1.310685
C -3.600271 1.204846 -0.912093
H -4.248364 1.215172 -1.798266
S -3.291663 -2.352887 0.880876
N -3.787808 -0.089235 -0.248772
N -1.550327 -0.285497 0.430360
C -1.819482 2.674519 -2.021112
C -2.579475 3.884373 -1.471184

C	-2.077227	2.539709	-3.522078
H	-0.737933	2.848140	-1.880738
H	-3.649344	3.823200	-1.726787
H	-2.494819	3.987584	-0.380128
H	-2.187495	4.805439	-1.927064
H	-1.458632	1.745489	-3.965425
H	-3.134304	2.297412	-3.716986
H	-1.848834	3.482044	-4.041638
H	-1.922816	0.527042	-2.022084
H	-0.219018	0.977047	-0.499184
C	-1.256669	2.115566	0.981203
C	-0.252486	3.090957	0.985778
C	-2.273646	2.176886	1.940338
C	-0.285535	4.133566	1.911110
H	0.561770	3.026123	0.258133
C	-2.304998	3.219937	2.866815
H	-3.052182	1.410411	1.978080
C	-1.317023	4.205070	2.848442
H	0.501355	4.889925	1.903244
H	-3.105268	3.260331	3.607848
H	-1.344113	5.020730	3.573182
C	0.758149	-0.477237	1.209459
H	0.841829	0.606032	1.268129
C	1.921971	-1.253883	1.232861
C	3.011970	-0.744237	2.137495
F	2.606210	-0.814523	3.416405
F	4.144810	-1.448826	2.080704
F	3.321184	0.536488	1.910800
O	-0.735358	-2.310018	1.111750
C	-0.478971	-1.121825	0.944373
H	-3.928643	2.002165	-0.228777
C	-6.252546	-0.221110	-0.637562
H	-6.324397	0.765001	-1.095834
H	-8.346307	-0.653046	-0.819977
H	1.814008	-2.341172	1.309528
C	0.457806	-3.959016	-1.503133
H	0.124761	-3.750441	-2.529322

H	0.713036	-5.019340	-1.402732
H	-0.339974	-3.686557	-0.797908
C	1.535351	-1.909690	-1.232581
O	1.645675	-3.236696	-1.200606
O	0.500183	-1.345983	-1.528380
C	4.061627	-1.854700	-0.573325
O	4.339351	-3.029212	-0.438450
C	2.719994	-1.223025	-0.715551
N	2.929348	0.174368	-0.983147
C	4.282225	0.473837	-0.741225
C	4.941095	1.708989	-0.726094
C	5.000381	-0.710984	-0.535298
C	6.317773	1.703150	-0.504623
H	4.399698	2.641267	-0.867336
C	6.373373	-0.704787	-0.302196
C	7.038129	0.517563	-0.289532
H	6.846977	2.658111	-0.486997
H	6.899164	-1.647233	-0.135130
H	8.112661	0.561278	-0.106933
C	2.135217	1.120213	-1.663627
O	2.153436	2.276261	-1.293965
C	1.432833	0.742657	-2.943150
H	1.770812	-0.214240	-3.351632
H	0.347498	0.698916	-2.788507
H	1.650903	1.555242	-3.650277

SMDopt/19_TS_SSa

Frequencies, energies and thermodynamic properties:

Lowest Vibrational Mode (1/cm) =	-224.9064
2nd Lowest Vibrational Mode (1/cm) =	17.3366
E(RM062X) (a.u.) =	-2588.55219527
Thermal correction to Enthalpy (a.u.) =	0.663129
Thermal correction to Gibbs Free Energy (a.u.) =	0.548628
Total Entropy (cal/Kmol) =	240.987
Esp(RM062X+D3) (a.u.) =	-2591.15504502
Esp(D3) (a.u.) =	-0.0100576193
Esp(RM062X) (a.u.) =	-2591.1449874007

Optimised cartesian coordinates (Angstrom):

C	-7.426852	-1.469358	-0.518132
C	-7.237811	-2.712549	0.102105
C	-5.983126	-3.095585	0.566862
C	-4.924213	-2.204784	0.396049
C	-5.114098	-0.970837	-0.228970
H	-8.084146	-3.389334	0.225411
H	-5.831362	-4.060830	1.052021
C	-2.871924	-0.831773	0.265558
C	-1.436598	1.094646	-0.244709
C	-2.410594	1.291596	-1.419495
C	-3.841560	1.067854	-0.964339
H	-4.521616	1.041834	-1.825567
S	-3.247767	-2.428826	0.866423
N	-3.926492	-0.235703	-0.300501
N	-1.675542	-0.237669	0.366830
C	-2.220624	2.631861	-2.166494
C	-3.056034	3.790875	-1.615662
C	-2.518686	2.442278	-3.654072
H	-1.155209	2.897897	-2.069498
H	-4.126466	3.638431	-1.826707
H	-2.938508	3.931366	-0.531812
H	-2.757339	4.727039	-2.109932
H	-1.849340	1.695267	-4.105207
H	-3.557155	2.106032	-3.804934
H	-2.391747	3.389188	-4.199217
H	-2.173638	0.486472	-2.133240
H	-0.422611	1.037444	-0.667886
C	-1.475656	2.207251	0.781542
C	-0.524922	3.230729	0.684228
C	-2.450730	2.270583	1.781687
C	-0.576477	4.324960	1.546177
H	0.267011	3.149240	-0.065358
C	-2.500167	3.365439	2.646315
H	-3.178562	1.463336	1.899809
C	-1.571293	4.399295	2.523046
H	0.167215	5.119302	1.460379

H	-3.267353	3.408294	3.421466
H	-1.612618	5.255160	3.199105
C	0.583985	-0.158827	1.296420
H	0.556999	0.925673	1.275886
C	1.792085	-0.806523	1.429904
C	2.869201	-0.091587	2.194397
F	2.616511	-0.155400	3.510996
F	4.090496	-0.610716	2.028130
F	2.947233	1.206080	1.883611
O	-0.705241	-2.141774	1.173220
C	-0.570959	-0.943884	0.976149
H	-4.190477	1.842343	-0.265462
C	-6.371210	-0.580015	-0.693048
H	-6.524067	0.388559	-1.168689
H	-8.420553	-1.188396	-0.869359
H	1.802369	-1.887494	1.578884
C	-0.384345	-2.150300	-2.012441
H	-0.611024	-2.980798	-1.327839
H	-1.282364	-1.543781	-2.176342
H	-0.030695	-2.554834	-2.969762
C	1.809830	-1.818830	-1.281089
O	0.587554	-1.288983	-1.434541
O	2.091069	-2.921277	-1.702380
C	2.690731	0.521504	-0.877751
O	1.707007	1.240302	-1.009196
C	2.732809	-0.918451	-0.572106
N	4.116874	-1.318472	-0.597582
C	4.918182	-0.195000	-0.854027
C	6.313114	-0.064774	-0.928354
C	4.100983	0.936669	-0.995862
C	6.832320	1.209401	-1.154675
H	6.964900	-0.926528	-0.826787
C	4.631516	2.205295	-1.223604
C	6.013485	2.339912	-1.303668
H	7.916008	1.324942	-1.223933
H	3.963297	3.062110	-1.331458
H	6.464644	3.317050	-1.482162

C 4.647748 -2.528817 -0.161116
O 5.812264 -2.798172 -0.374092
C 3.787891 -3.442540 0.675835
H 3.875900 -3.109399 1.721694
H 2.734624 -3.464184 0.390940
H 4.208891 -4.452025 0.602867

SMDopt/19_TS_SSb

Frequencies, energies and thermodynamic properties:

Lowest Vibrational Mode (1/cm) = -252.2233
2nd Lowest Vibrational Mode (1/cm) = 17.0092
E(RM062X) (a.u.) = -2588.55209251
Thermal correction to Enthalpy (a.u.) = 0.663039
Thermal correction to Gibbs Free Energy (a.u.) = 0.547845
Total Entropy (cal/Kmol) = 242.445
Esp(RM062X+D3) (a.u.) = -2591.15400451
Esp(D3) (a.u.) = -0.0099446274
Esp(RM062X) (a.u.) = -2591.1440598826

Optimised cartesian coordinates (Angstrom):

C 7.391120 -1.549777 0.571753
C 7.229286 -2.720617 -0.182001
C 5.997039 -3.044173 -0.743102
C 4.933410 -2.167835 -0.533010
C 5.095603 -1.006875 0.225890
H 8.078979 -3.387162 -0.334634
H 5.866585 -3.953335 -1.331859
C 2.880124 -0.799268 -0.359170
C 1.445924 1.091632 0.247412
C 2.329225 1.134022 1.504704
C 3.787956 0.939081 1.131225
H 4.404184 0.793944 2.028254
S 3.279044 -2.331834 -1.099820
N 3.908033 -0.274856 0.318904
N 1.701066 -0.181391 -0.470016
C 2.093363 2.381730 2.385091
C 2.980220 3.580382 2.037617
C 2.267474 2.017187 3.859683

H	1.041510	2.676094	2.234119
H	4.029795	3.384776	2.309272
H	2.947640	3.844307	0.970804
H	2.653890	4.460284	2.611402
H	1.556184	1.235822	4.165007
H	3.286579	1.645047	4.052763
H	2.105616	2.896766	4.500188
H	2.021319	0.253105	2.091202
H	0.406138	1.020938	0.595904
C	1.584594	2.294115	-0.661369
C	0.643897	3.325015	-0.543320
C	2.638844	2.431378	-1.570545
C	0.781127	4.494273	-1.290428
H	-0.206253	3.190370	0.130912
C	2.773296	3.600485	-2.321026
H	3.364666	1.625211	-1.705928
C	1.851660	4.638029	-2.174743
H	0.044563	5.293378	-1.188230
H	3.601344	3.698982	-3.025261
H	1.959733	5.552314	-2.761014
C	-0.546933	-0.030262	-1.411706
H	-0.508133	1.053286	-1.342245
C	-1.766025	-0.666953	-1.526786
C	-2.858161	0.033313	-2.281981
F	-2.621966	-0.038473	-3.601023
F	-4.069854	-0.503572	-2.094428
F	-2.950969	1.331869	-1.981317
O	0.736379	-2.022556	-1.421034
C	0.600478	-0.843366	-1.135056
H	4.194577	1.787082	0.560390
C	6.329643	-0.675907	0.787509
H	6.460721	0.237582	1.367236
H	8.367282	-1.313901	0.997523
H	-1.768506	-1.743935	-1.701794
C	-0.956675	-3.405193	2.365146
H	-0.559087	-2.854611	3.228691
H	-1.427993	-4.335523	2.699010

H	-0.132271	-3.626145	1.672449
C	-1.583734	-1.483621	1.181691
O	-1.971956	-2.655150	1.711125
O	-0.456800	-1.055885	1.315814
C	-2.711953	0.671072	0.741440
O	-1.777236	1.452672	0.854803
C	-2.643505	-0.774516	0.451940
N	-3.990594	-1.284668	0.516346
C	-4.872509	-0.223176	0.780628
C	-6.270884	-0.204949	0.891227
C	-4.146582	0.972925	0.891846
C	-6.885474	1.025292	1.121945
H	-6.853462	-1.117051	0.812459
C	-4.772155	2.196874	1.123635
C	-6.157788	2.219885	1.239772
H	-7.972787	1.053161	1.219164
H	-4.172933	3.105854	1.206565
H	-6.681867	3.159015	1.422798
C	-4.432467	-2.535851	0.101687
O	-5.576871	-2.883818	0.308185
C	-3.501619	-3.413880	-0.697899
H	-2.439862	-3.295056	-0.475788
H	-3.798288	-4.455282	-0.526239
H	-3.674024	-3.182904	-1.761081

SMDopt/19_TS_SSc

Frequencies, energies and thermodynamic properties:

Lowest Vibrational Mode (1/cm) =	-255.8761
2nd Lowest Vibrational Mode (1/cm) =	16.3879
E(RM062X) (a.u.) =	-2588.55029538
Thermal correction to Enthalpy (a.u.) =	0.662833
Thermal correction to Gibbs Free Energy (a.u.) =	0.547363
Total Entropy (cal/Kmol) =	243.028
Esp(RM062X+D3) (a.u.) =	-2591.15262983
Esp(D3) (a.u.) =	-0.0098413757
Esp(RM062X) (a.u.) =	-2591.1427884543

Optimised cartesian coordinates (Angstrom):

C	7.406720	-1.516698	0.567549
C	7.257142	-2.685446	-0.191857
C	6.028842	-3.017596	-0.756778
C	4.956855	-2.151988	-0.545062
C	5.106942	-0.992972	0.219330
H	8.113207	-3.343467	-0.345909
H	5.907586	-3.925353	-1.349676
C	2.890313	-0.803136	-0.368956
C	1.441033	1.073292	0.239247
C	2.318015	1.117472	1.500754
C	3.779925	0.935136	1.133589
H	4.392746	0.787822	2.032642
S	3.304764	-2.329361	-1.114981
N	3.912647	-0.272283	0.313510
N	1.706499	-0.196206	-0.479773
C	2.067139	2.359459	2.384950
C	2.945816	3.566937	2.047489
C	2.235959	1.990338	3.859041
H	1.013530	2.645169	2.229031
H	3.995413	3.379106	2.324559
H	2.917268	3.834743	0.981528
H	2.608779	4.441861	2.622733
H	1.529809	1.201434	4.156889
H	3.257172	1.626293	4.056489
H	2.062676	2.865675	4.502405
H	2.013170	0.231852	2.081718
H	0.400349	0.996708	0.583656
C	1.577746	2.278806	-0.665841
C	0.629642	3.303378	-0.551012
C	2.637005	2.425499	-1.567772
C	0.764658	4.475763	-1.293722
H	-0.223992	3.160092	0.117087
C	2.769054	3.597627	-2.313991
H	3.368806	1.624291	-1.700570
C	1.840056	4.629013	-2.170546
H	0.022545	5.270074	-1.193997
H	3.601029	3.703413	-3.012528

H	1.946334	5.545810	-2.753235
C	-0.546124	-0.067788	-1.412495
H	-0.502585	1.017487	-1.365152
C	-1.772385	-0.695655	-1.495761
C	-2.855033	0.017065	-2.253295
F	-2.629155	-0.079531	-3.573811
F	-4.080641	-0.481776	-2.054549
F	-2.913753	1.323752	-1.974078
O	0.761695	-2.045888	-1.434190
C	0.609515	-0.870998	-1.142606
H	4.183685	1.789970	0.570938
C	6.336764	-0.653551	0.785106
H	6.458536	0.258425	1.369223
H	8.379803	-1.273859	0.996454
H	-1.822819	-1.780515	-1.608177
C	-0.936254	-3.425485	2.345356
H	-0.487374	-2.892638	3.195358
H	-1.414657	-4.347115	2.693572
H	-0.145935	-3.663336	1.619149
C	-1.561495	-1.494203	1.190806
O	-1.957009	-2.649116	1.738045
O	-0.423351	-1.084157	1.296667
C	-2.695742	0.676683	0.776017
O	-1.765601	1.466455	0.898289
C	-2.624941	-0.758887	0.495027
N	-3.965588	-1.283043	0.530727
C	-4.862659	-0.222890	0.759833
C	-6.263023	-0.181870	0.845386
C	-4.135383	0.974269	0.894669
C	-6.873103	1.051088	1.076549
H	-6.889781	-1.062870	0.754562
C	-4.754930	2.201162	1.122685
C	-6.141572	2.239004	1.215286
H	-7.961678	1.081578	1.154141
H	-4.146015	3.101967	1.223009
H	-6.661340	3.181070	1.394706
C	-4.239856	-2.530542	-0.030578

O -3.366295 -3.158334 -0.587793
C -5.638114 -3.071452 0.095140
H -6.035863 -2.946557 1.109711
H -6.305465 -2.562312 -0.615339
H -5.600797 -4.135013 -0.163948

SMDopt/19_TS_SSd

Frequencies, energies and thermodynamic properties:

Lowest Vibrational Mode (1/cm) = -163.8651
2nd Lowest Vibrational Mode (1/cm) = 14.0204
E(RM062X) (a.u.) = -2588.54674981
Thermal correction to Enthalpy (a.u.) = 0.662994
Thermal correction to Gibbs Free Energy (a.u.) = 0.547876
Total Entropy (cal/Kmol) = 242.285
Esp(RM062X+D3) (a.u.) = -2591.15389090
Esp(D3) (a.u.) = -0.0109946719
Esp(RM062X) (a.u.) = -2591.1428962281

Optimised cartesian coordinates (Angstrom):

C 6.911288 -1.638754 -0.573508
C 6.566151 -2.777732 -1.315888
C 5.240888 -3.038734 -1.648015
C 4.271061 -2.133620 -1.217227
C 4.616096 -1.005766 -0.470593
H 7.346093 -3.466995 -1.641569
H 4.966619 -3.920552 -2.228912
C 2.341828 -0.705113 -0.632184
C 1.095747 1.175994 0.348511
C 2.237680 1.154110 1.378042
C 3.570391 0.973636 0.677015
H 4.373367 0.824409 1.409533
S 2.540650 -2.211768 -1.490480
N 3.494016 -0.237356 -0.144908
N 1.173166 -0.052675 -0.482528
C 2.217656 2.347724 2.357559
C 3.015285 3.565991 1.884876
C 2.723407 1.894926 3.727705
H 1.161319 2.642113 2.472357

H	4.096786	3.356954	1.905064
H	2.749573	3.887492	0.867823
H	2.834435	4.412733	2.563235
H	2.093556	1.093525	4.142105
H	3.755963	1.516171	3.658506
H	2.721750	2.733649	4.439312
H	2.069421	0.242298	1.972386
H	0.166068	1.085633	0.925721
C	1.017985	2.441385	-0.479073
C	0.170582	3.464721	-0.034553
C	1.781850	2.636131	-1.632994
C	0.113617	4.678896	-0.715885
H	-0.448656	3.296590	0.852063
C	1.723249	3.852964	-2.315431
H	2.424408	1.839148	-2.016290
C	0.896619	4.877739	-1.854977
H	-0.548370	5.470396	-0.360162
H	2.324673	3.997217	-3.214609
H	0.852197	5.827186	-2.391291
C	-1.177754	0.262957	-1.154695
H	-1.102064	1.329671	-0.960873
C	-2.381285	-0.294238	-1.501231
C	-3.420254	0.590523	-2.140540
F	-3.424055	0.357919	-3.462383
F	-4.674573	0.394308	-1.726474
F	-3.164382	1.893980	-1.985389
O	0.036480	-1.725921	-1.535326
C	-0.016619	-0.597120	-1.083923
H	3.833415	1.824554	0.030947
C	5.945894	-0.736133	-0.140211
H	6.224025	0.151896	0.426844
H	7.958209	-1.451285	-0.331659
H	-2.437286	-1.327361	-1.851422
C	-6.835515	-1.501617	-0.105790
H	-7.119993	-0.480916	-0.401015
H	-7.239691	-2.221797	-0.825746
H	-7.245445	-1.704549	0.893661

C -4.740184 -0.851242 0.688142
O -5.428955 -1.659199 -0.128549
O -5.290334 -0.088276 1.456982
C -2.734604 -2.364972 0.332037
O -3.144864 -3.274614 -0.371885
C -3.294971 -1.022348 0.543636
N -2.438275 -0.328702 1.462756
C -1.366199 -1.174722 1.819430
C -0.265932 -0.958176 2.664920
C -1.505208 -2.399290 1.156102
C 0.661469 -1.992516 2.796960
H -0.140924 -0.023871 3.203597
C -0.572559 -3.423674 1.292411
C 0.524551 -3.215725 2.121896
H 1.519199 -1.840786 3.456487
H -0.714023 -4.357696 0.744961
H 1.276058 -3.995746 2.253484
C -2.398637 1.035118 1.701110
O -1.571328 1.511047 2.465125
C -3.371930 1.950185 1.007357
H -3.992410 1.484519 0.245058
H -4.036273 2.371162 1.774696
H -2.785482 2.774445 0.577648

SMDopt/19_TS_SSe

Frequencies, energies and thermodynamic properties:

Lowest Vibrational Mode (1/cm) = -217.3198

2nd Lowest Vibrational Mode (1/cm) = 19.3824

E(RM062X) (a.u.) = -2588.54907014

Thermal correction to Enthalpy (a.u.) = 0.662951

Thermal correction to Gibbs Free Energy (a.u.) = 0.549097

Total Entropy (cal/Kmol) = 239.627

Esp(RM062X+D3) (a.u.) = -2591.15266236

Esp(D3) (a.u.) = -0.0099641682

Esp(RM062X) (a.u.) = -2591.1426981918

Optimised cartesian coordinates (Angstrom):

C -7.444292 -1.423908 -0.496126

C	-7.270011	-2.662955	0.136586
C	-6.020066	-3.055726	0.606148
C	-4.950856	-2.178985	0.427272
C	-5.125922	-0.949576	-0.210945
H	-8.124209	-3.328626	0.266032
H	-5.879504	-4.017788	1.100904
C	-2.882621	-0.831432	0.283659
C	-1.426082	1.072915	-0.244201
C	-2.393536	1.266348	-1.424760
C	-3.828726	1.063593	-0.972796
H	-4.505764	1.031959	-1.836375
S	-3.277304	-2.417756	0.900877
N	-3.929690	-0.229484	-0.290620
N	-1.679801	-0.250747	0.379799
C	-2.184574	2.595572	-2.186297
C	-3.010821	3.769864	-1.654498
C	-2.474527	2.391906	-3.673582
H	-1.116956	2.850963	-2.084963
H	-4.081549	3.626083	-1.870034
H	-2.898025	3.921999	-0.571700
H	-2.699407	4.696930	-2.157997
H	-1.810288	1.632407	-4.111236
H	-3.515523	2.065184	-3.827882
H	-2.333454	3.330891	-4.228902
H	-2.162420	0.450428	-2.128139
H	-0.410862	1.003166	-0.663316
C	-1.459569	2.196305	0.770557
C	-0.495558	3.207126	0.669614
C	-2.440158	2.281137	1.763801
C	-0.539392	4.310001	1.521062
H	0.300726	3.106307	-0.073131
C	-2.481541	3.384527	2.617963
H	-3.178703	1.484160	1.885078
C	-1.539303	4.405769	2.490902
H	0.214609	5.094345	1.432929
H	-3.252917	3.443985	3.387857
H	-1.574254	5.268325	3.158789

C	0.581371	-0.187305	1.304047
H	0.546881	0.897795	1.304848
C	1.795572	-0.823564	1.410595
C	2.869884	-0.094394	2.165899
F	2.636949	-0.181383	3.486570
F	4.101654	-0.576538	1.979449
F	2.911008	1.210964	1.874889
O	-0.730118	-2.159102	1.200905
C	-0.581387	-0.966365	0.991674
H	-4.172777	1.851497	-0.286634
C	-6.378176	-0.548814	-0.679515
H	-6.519294	0.416591	-1.165027
H	-8.434497	-1.134892	-0.850692
H	1.860560	-1.909300	1.499009
C	-0.424671	-2.187185	-1.983769
H	-0.627380	-3.029939	-1.306557
H	-1.336384	-1.595987	-2.128225
H	-0.080978	-2.576617	-2.951225
C	1.780688	-1.830056	-1.298576
O	0.541259	-1.316694	-1.413904
O	2.066157	-2.918916	-1.740427
C	2.674246	0.522857	-0.913556
O	1.697414	1.255828	-1.052597
C	2.707747	-0.907222	-0.625342
N	4.081870	-1.329024	-0.621506
C	4.904208	-0.208868	-0.829796
C	6.299845	-0.062165	-0.861068
C	4.091838	0.928221	-0.993502
C	6.822488	1.213020	-1.076262
H	6.986638	-0.893582	-0.737573
C	4.624807	2.197986	-1.204717
C	6.007271	2.340864	-1.248640
H	7.907658	1.327456	-1.112569
H	3.953969	3.050595	-1.329097
H	6.459956	3.319579	-1.413472
C	4.436608	-2.544459	-0.030705
O	3.621912	-3.190563	0.588623

C	5.853378	-3.022232	-0.205768
H	6.233858	-2.829783	-1.216007
H	6.508730	-2.525780	0.525064
H	5.863295	-4.098094	0.001407

SMDopt/19_TS_SSf

Frequencies, energies and thermodynamic properties:

Lowest Vibrational Mode (1/cm) = -148.9702

2nd Lowest Vibrational Mode (1/cm) = 15.0124

E(RM062X) (a.u.) = -2588.54113254

Thermal correction to Enthalpy (a.u.) = 0.662684

Thermal correction to Gibbs Free Energy (a.u.) = 0.547182

Total Entropy (cal/Kmol) = 243.094

Esp(RM062X+D3) (a.u.) = -2591.15126718

Esp(D3) (a.u.) = -0.0110177697

Esp(RM062X) (a.u.) = -2591.1402494103

Optimised cartesian coordinates (Angstrom):

C	6.649464	-2.053796	-0.439166
C	6.222395	-3.137530	-1.221185
C	4.893005	-3.255603	-1.611504
C	4.001256	-2.265849	-1.196623
C	4.426436	-1.194966	-0.408633
H	6.941690	-3.896160	-1.531908
H	4.555810	-4.092784	-2.224329
C	2.199636	-0.673033	-0.639912
C	1.111062	1.311968	0.328036
C	2.234420	1.218583	1.373082
C	3.554096	0.879257	0.709044
H	4.314385	0.656106	1.467685
S	2.290781	-2.151676	-1.558705
N	3.373591	-0.325212	-0.106381
N	1.084552	0.069884	-0.484392
C	2.322682	2.450947	2.300854
C	3.249188	3.559804	1.795198
C	2.752152	2.008583	3.699936
H	1.302751	2.862990	2.377677
H	4.302860	3.241715	1.840185

H	3.028947	3.866689	0.762704
H	3.148876	4.446403	2.438282
H	2.034432	1.295182	4.132396
H	3.740070	1.521398	3.669641
H	2.825532	2.872646	4.376477
H	1.968806	0.355272	2.000629
H	0.168775	1.316661	0.893415
C	1.165168	2.546976	-0.548265
C	0.401168	3.663940	-0.190804
C	1.996253	2.618982	-1.671007
C	0.493868	4.847282	-0.921314
H	-0.274582	3.601534	0.666615
C	2.087967	3.803470	-2.403310
H	2.579510	1.751548	-1.990718
C	1.344610	4.921705	-2.025404
H	-0.105415	5.712000	-0.631326
H	2.742205	3.849241	-3.275562
H	1.417644	5.847018	-2.599388
C	-1.275331	0.546555	-1.035836
H	-1.193499	1.558338	-0.651188
C	-2.487191	0.070338	-1.426881
C	-3.585473	1.054322	-1.738656
F	-3.735138	1.082566	-3.080954
F	-4.784390	0.762029	-1.247512
F	-3.294056	2.302737	-1.373988
O	-0.113946	-1.365630	-1.784142
C	-0.120719	-0.329493	-1.150205
H	3.927857	1.685544	0.060131
C	5.762674	-1.067289	-0.022089
H	6.105802	-0.224273	0.576715
H	7.699020	-1.978102	-0.152156
H	-2.593851	-0.893683	-1.928809
C	-6.715463	-1.666280	-0.440264
H	-7.050840	-0.619489	-0.487919
H	-7.045098	-2.200530	-1.338910
H	-7.160452	-2.132420	0.450995
C	-4.706766	-1.131192	0.620681

O	-5.305669	-1.746257	-0.417983
O	-5.343008	-0.605021	1.508811
C	-2.564155	-2.422006	0.085248
O	-2.891929	-3.262950	-0.747898
C	-3.256985	-1.231505	0.540605
N	-2.473081	-0.589950	1.544622
C	-1.284248	-1.318653	1.727767
C	-0.169913	-1.108302	2.556910
C	-1.305214	-2.432997	0.871527
C	0.878911	-2.026216	2.494848
H	-0.091860	-0.271151	3.244308
C	-0.249388	-3.338516	0.814281
C	0.854927	-3.135104	1.635440
H	1.743574	-1.869162	3.144087
H	-0.303910	-4.178452	0.118309
H	1.700316	-3.825079	1.613215
C	-2.676609	0.755540	1.859599
O	-3.436839	1.435758	1.208164
C	-1.962895	1.312882	3.067953
H	-1.935816	0.596516	3.898061
H	-0.931087	1.601831	2.817177
H	-2.502934	2.216393	3.372583

SMDopt/19_TS_SSg

Frequencies, energies and thermodynamic properties:

Lowest Vibrational Mode (1/cm) = -175.5015

2nd Lowest Vibrational Mode (1/cm) = 11.3614

E(RM062X) (a.u.) = -2588.54434267

Thermal correction to Enthalpy (a.u.) = 0.663048

Thermal correction to Gibbs Free Energy (a.u.) = 0.547428

Total Entropy (cal/Kmol) = 243.343

Esp(RM062X+D3) (a.u.) = -2591.15138659

Esp(D3) (a.u.) = -0.0109723965

Esp(RM062X) (a.u.) = -2591.1404141935

Optimised cartesian coordinates (Angstrom):

C	6.934704	-1.502007	-0.277556
C	6.644733	-2.646112	-1.035579

C	5.343829	-2.920638	-1.444094
C	4.340819	-2.025038	-1.072534
C	4.630887	-0.891774	-0.310792
H	7.449083	-3.328547	-1.312671
H	5.113462	-3.806558	-2.037748
C	2.364802	-0.618203	-0.600171
C	1.031338	1.222910	0.346171
C	2.137769	1.229177	1.413921
C	3.498446	1.083470	0.760685
H	4.279196	0.963545	1.521694
S	2.630836	-2.119155	-1.450386
N	3.483883	-0.134694	-0.053064
N	1.178686	0.011974	-0.501985
C	2.055487	2.420072	2.393415
C	2.835836	3.659253	1.947313
C	2.528896	1.977203	3.778318
H	0.989424	2.688498	2.475238
H	3.921165	3.476244	1.994494
H	2.588518	3.979440	0.925224
H	2.617153	4.498002	2.624487
H	1.906873	1.158959	4.171103
H	3.572192	1.624370	3.741003
H	2.483845	2.813830	4.490941
H	1.974130	0.312501	2.001518
H	0.086814	1.090301	0.891406
C	0.934128	2.496518	-0.467338
C	0.030146	3.479488	-0.044120
C	1.734303	2.738872	-1.587233
C	-0.046438	4.701674	-0.708909
H	-0.618618	3.274145	0.812869
C	1.655804	3.963138	-2.254045
H	2.422349	1.973175	-1.954549
C	0.773307	4.948683	-1.812124
H	-0.752468	5.461323	-0.368925
H	2.286348	4.144415	-3.126190
H	0.713809	5.904394	-2.335699
C	-1.165559	0.262423	-1.221712

H	-1.153543	1.313325	-0.947590
C	-2.335330	-0.328244	-1.635315
C	-3.390048	0.556746	-2.246486
F	-3.310230	0.457184	-3.583587
F	-4.646752	0.250504	-1.925086
F	-3.221033	1.851838	-1.954275
O	0.151529	-1.636461	-1.698027
C	0.032459	-0.541756	-1.177393
H	3.758212	1.937531	0.117424
C	5.936768	-0.608115	0.095610
H	6.172958	0.284089	0.675119
H	7.963730	-1.303687	0.024853
H	-2.325352	-1.327554	-2.075948
C	-6.592292	-0.502145	1.600946
H	-7.030272	-1.499608	1.745959
H	-6.793696	0.122983	2.478014
H	-7.040621	-0.047391	0.705861
C	-4.721845	-1.263159	0.407255
O	-5.181884	-0.578126	1.476518
O	-5.461384	-1.868047	-0.331149
C	-2.613470	-2.523912	0.019055
O	-2.976470	-3.408276	-0.739341
C	-3.258510	-1.231598	0.300098
N	-2.467910	-0.554568	1.288689
C	-1.344464	-1.349167	1.600248
C	-0.268414	-1.114959	2.471935
C	-1.394713	-2.535061	0.857661
C	0.728817	-2.087852	2.544551
H	-0.213158	-0.212094	3.072170
C	-0.393834	-3.499498	0.937697
C	0.682169	-3.269943	1.788277
H	1.569950	-1.920551	3.221637
H	-0.464548	-4.402190	0.327598
H	1.487081	-4.001606	1.874408
C	-2.526838	0.789229	1.618948
O	-1.733939	1.274872	2.411194
C	-3.583665	1.667068	0.999485

H -4.153905 1.216090 0.188160
H -4.285734 1.953251 1.795465
H -3.081228 2.578994 0.649227

SMDopt/19_TS_SSh
Frequencies, energies and thermodynamic properties:
Lowest Vibrational Mode (1/cm) = -157.5916
2nd Lowest Vibrational Mode (1/cm) = 14.7130
E(RM062X) (a.u.) = -2588.53937821
Thermal correction to Enthalpy (a.u.) = 0.662593
Thermal correction to Gibbs Free Energy (a.u.) = 0.546242
Total Entropy (cal/Kmol) = 244.882
Esp(RM062X+D3) (a.u.) = -2591.14930184
Esp(D3) (a.u.) = -0.0110490437
Esp(RM062X) (a.u.) = -2591.1382527963
Optimised cartesian coordinates (Angstrom):
C 6.727357 -1.847598 -0.224355
C 6.375515 -2.926469 -1.049304
C 5.073121 -3.074028 -1.513668
C 4.131755 -2.118634 -1.128995
C 4.481719 -1.051507 -0.299689
H 7.132719 -3.657831 -1.334264
H 4.793798 -3.907902 -2.159294
C 2.253543 -0.594355 -0.637863
C 1.039996 1.316976 0.334525
C 2.130254 1.248426 1.416318
C 3.485148 0.983797 0.790517
H 4.232755 0.790988 1.569348
S 2.440380 -2.049043 -1.579887
N 3.387882 -0.219574 -0.039952
N 1.106548 0.105032 -0.519869
C 2.131369 2.459556 2.375157
C 3.017086 3.623812 1.923932
C 2.541588 2.004357 3.775935
H 1.090507 2.819346 2.431536
H 4.083227 3.355897 1.995068
H 2.813966 3.944680 0.892298

H	2.854103	4.488838	2.583430
H	1.847841	1.246567	4.170303
H	3.553090	1.567352	3.764501
H	2.552421	2.854447	4.473763
H	1.883869	0.358580	2.014063
H	0.081025	1.248227	0.866143
C	1.048844	2.585734	-0.493928
C	0.212841	3.642240	-0.113764
C	1.899817	2.749788	-1.591536
C	0.255099	4.857954	-0.793637
H	-0.480991	3.505856	0.720518
C	1.940745	3.966808	-2.273707
H	2.537577	1.929796	-1.931386
C	1.126779	5.025502	-1.870996
H	-0.400112	5.674584	-0.485772
H	2.611323	4.084850	-3.126556
H	1.160689	5.976147	-2.405902
C	-1.250704	0.491311	-1.143916
H	-1.227508	1.493419	-0.727724
C	-2.431287	-0.027713	-1.581014
C	-3.556525	0.923241	-1.905780
F	-3.672001	0.966825	-3.251378
F	-4.756621	0.586205	-1.450300
F	-3.315765	2.176089	-1.517248
O	0.023016	-1.342861	-1.905830
C	-0.055449	-0.326675	-1.243927
H	3.836010	1.814882	0.160208
C	5.790976	-0.894880	0.162038
H	6.077445	-0.056180	0.795916
H	7.756871	-1.748739	0.122079
H	-2.478585	-0.974887	-2.122663
C	-6.646709	-0.818628	1.270375
H	-7.105462	-1.817916	1.253616
H	-6.963883	-0.282554	2.172295
H	-6.976756	-0.269009	0.376585
C	-4.645260	-1.466192	0.249406
O	-5.236317	-0.898409	1.322209

O -5.289537 -2.015512 -0.617125
C -2.416974 -2.549330 -0.152102
O -2.675266 -3.380298 -1.017110
C -3.191900 -1.407340 0.296008
N -2.488452 -0.752921 1.353199
C -1.274830 -1.431590 1.576400
C -0.216193 -1.197603 2.470127
C -1.198524 -2.522337 0.692918
C 0.875872 -2.065634 2.439789
H -0.212595 -0.380770 3.185561
C -0.100852 -3.378210 0.668378
C 0.948867 -3.149218 1.551855
H 1.696448 -1.888935 3.139206
H -0.081679 -4.201444 -0.049114
H 1.825029 -3.800025 1.557137
C -2.763559 0.574711 1.689301
O -3.532413 1.234910 1.027252
C -2.121830 1.136608 2.934958
H -2.114252 0.409827 3.756584
H -1.087444 1.456848 2.739310
H -2.701891 2.019986 3.224369

SMDopt/19_TS_SSi

Frequencies, energies and thermodynamic properties:

Lowest Vibrational Mode (1/cm) = -157.5410

2nd Lowest Vibrational Mode (1/cm) = 14.6871

E(RM062X) (a.u.) = -2588.53937820

Thermal correction to Enthalpy (a.u.) = 0.662592

Thermal correction to Gibbs Free Energy (a.u.) = 0.546250

Total Entropy (cal/Kmol) = 244.863

Esp(RM062X+D3) (a.u.) = -2591.14930222

Esp(D3) (a.u.) = -0.0110493797

Esp(RM062X) (a.u.) = -2591.1382528403

Optimised cartesian coordinates (Angstrom):

C 6.727988 -1.846473 -0.224208
C 6.376349 -2.925648 -1.048845
C 5.073985 -3.073577 -1.513176

C	4.132441	-2.118243	-1.128787
C	4.482205	-1.050806	-0.299790
H	7.133686	-3.656959	-1.333580
H	4.794818	-3.907697	-2.158554
C	2.253928	-0.594202	-0.638072
C	1.039759	1.316800	0.334311
C	2.130313	1.248804	1.415844
C	3.485155	0.984705	0.789703
H	4.233108	0.792549	1.568358
S	2.441048	-2.049110	-1.579694
N	3.388207	-0.219012	-0.040301
N	1.106765	0.104935	-0.520181
C	2.131140	2.459989	2.374611
C	3.016206	3.624619	1.923077
C	2.541940	2.005051	3.775302
H	1.090132	2.819316	2.431257
H	4.082485	3.357153	1.993818
H	2.812568	3.945415	0.891521
H	2.853103	4.489568	2.582647
H	1.848649	1.246955	4.169886
H	3.553645	1.568521	3.763630
H	2.552554	2.855179	4.473087
H	1.884511	0.358881	2.013713
H	0.080934	1.247397	0.866115
C	1.047651	2.585661	-0.493987
C	0.211066	3.641575	-0.113451
C	1.898209	2.750405	-1.591808
C	0.252367	4.857414	-0.793152
H	-0.482496	3.504611	0.720964
C	1.938172	3.967551	-2.273816
H	2.536365	1.930846	-1.931941
C	1.123651	5.025673	-1.870723
H	-0.403286	5.673579	-0.484996
H	2.608430	4.086142	-3.126840
H	1.156810	5.976415	-2.405503
C	-1.250583	0.490563	-1.144242
H	-1.227556	1.492777	-0.728302

C	-2.431112	-0.028729	-1.581155
C	-3.556442	0.922033	-1.906175
F	-3.671883	0.965276	-3.251788
F	-4.756515	0.584977	-1.450648
F	-3.315828	2.175000	-1.517946
O	0.023522	-1.343493	-1.905827
C	-0.055159	-0.327202	-1.244115
H	3.835379	1.815764	0.159005
C	5.791432	-0.893811	0.161900
H	6.077744	-0.054881	0.795546
H	7.757480	-1.747330	0.122213
H	-2.478337	-0.976074	-2.122516
C	-6.646360	-0.818928	1.270917
H	-7.105054	-1.818248	1.254504
H	-6.963487	-0.282596	2.172700
H	-6.976518	-0.269600	0.376989
C	-4.644959	-1.466699	0.249976
O	-5.235958	-0.898605	1.322650
O	-5.289276	-2.016370	-0.616301
C	-2.416593	-2.549747	-0.151386
O	-2.674873	-3.381041	-1.016088
C	-3.191602	-1.407698	0.296417
N	-2.488131	-0.752851	1.353325
C	-1.274397	-1.431273	1.576605
C	-0.215668	-1.196808	2.470101
C	-1.198045	-2.522302	0.693476
C	0.876530	-2.064674	2.439893
H	-0.212081	-0.379700	3.185226
C	-0.100238	-3.378008	0.669067
C	0.949570	-3.148550	1.552315
H	1.697197	-1.887578	3.139105
H	-0.081035	-4.201478	-0.048153
H	1.825840	-3.799210	1.557687
C	-2.763372	0.574848	1.689056
O	-3.532257	1.234793	1.026791
C	-2.121819	1.137109	2.934643
H	-2.114068	0.410455	3.756379

H -1.087516 1.457626 2.739015
H -2.702140 2.020369 3.223901

SMDopt/19_TS_SSj
Frequencies, energies and thermodynamic properties:
Lowest Vibrational Mode (1/cm) = -391.7051
2nd Lowest Vibrational Mode (1/cm) = 11.4275
E(RM062X) (a.u.) = -2588.53787619
Thermal correction to Enthalpy (a.u.) = 0.662568
Thermal correction to Gibbs Free Energy (a.u.) = 0.547966
Total Entropy (cal/Kmol) = 241.200
Esp(RM062X+D3) (a.u.) = -2591.14045797
Esp(D3) (a.u.) = -0.0106771277
Esp(RM062X) (a.u.) = -2591.1297808423
Optimised cartesian coordinates (Angstrom):
C -6.703457 2.101553 0.019999
C -6.325618 3.223978 -0.729698
C -5.020146 3.365871 -1.191805
C -4.104228 2.359996 -0.886432
C -4.481324 1.247944 -0.131125
H -7.062167 3.995499 -0.957290
H -4.721056 4.236549 -1.777547
C -2.270330 0.737975 -0.529863
C -1.147077 -1.291546 0.238715
C -2.147071 -1.204026 1.403586
C -3.525326 -0.843507 0.881461
H -4.204861 -0.618937 1.713512
S -2.404806 2.289170 -1.326233
N -3.412719 0.367667 0.065423
N -1.174585 -0.024809 -0.527607
C -2.150749 -2.450680 2.315457
C -3.128650 -3.548341 1.887528
C -2.438214 -2.034779 3.758436
H -1.129223 -2.866727 2.283209
H -4.170693 -3.228730 2.047612
H -3.018900 -3.836148 0.832305
H -2.966350 -4.447638 2.499805

H	-1.680146	-1.329983	4.130522
H	-3.423424	-1.547160	3.835340
H	-2.446629	-2.911799	4.422438
H	-1.798619	-0.350537	2.008161
H	-0.145561	-1.346655	0.687128
C	-1.319689	-2.499254	-0.658706
C	-0.528674	-3.629158	-0.418360
C	-2.272902	-2.537181	-1.681404
C	-0.717123	-4.794496	-1.159793
H	0.245482	-3.584140	0.352694
C	-2.459367	-3.702973	-2.425784
H	-2.876969	-1.655482	-1.910557
C	-1.690154	-4.836361	-2.159857
H	-0.097313	-5.670616	-0.960979
H	-3.208360	-3.723627	-3.219412
H	-1.838911	-5.747434	-2.742111
C	1.129244	-0.441444	-1.214272
H	0.983569	-1.501488	-1.030920
C	2.439993	0.052567	-1.272562
C	3.456938	-0.835580	-1.932370
F	3.271084	-0.844328	-3.263184
F	4.715060	-0.452984	-1.731307
F	3.361334	-2.112633	-1.532648
O	0.036148	1.641908	-1.522340
C	0.053596	0.479234	-1.132796
H	-3.970491	-1.643264	0.270708
C	-5.790392	1.097835	0.330436
H	-6.093658	0.224171	0.907016
H	-7.733168	2.007210	0.367345
H	2.561365	1.095475	-1.584729
C	4.538664	-2.937545	1.206110
H	4.017522	-3.480993	0.406901
H	5.614977	-3.131770	1.153277
H	4.145949	-3.264818	2.178633
C	3.127988	-1.084078	1.038722
O	4.381127	-1.534364	1.044492
O	2.167618	-1.808487	1.206178

C	4.195852	1.277216	0.520270
O	5.382823	1.053200	0.439768
C	3.042980	0.337493	0.674248
N	1.921159	1.134317	1.085057
C	2.185352	2.474274	0.723072
C	1.337180	3.583411	0.690742
C	3.544765	2.609711	0.412968
C	1.904178	4.815025	0.354741
H	0.276541	3.484142	0.899710
C	4.100765	3.840800	0.081215
C	3.265362	4.956146	0.052168
H	1.257500	5.694178	0.318210
H	5.165578	3.913751	-0.149343
H	3.663703	5.936551	-0.212718
C	0.859873	0.840060	1.946425
O	-0.176387	1.470840	1.855110
C	1.055189	-0.138951	3.077917
H	0.553049	-1.090969	2.860143
H	2.108190	-0.337328	3.302419
H	0.570474	0.319363	3.950385

SMDopt/19_TS_SSk

Frequencies, energies and thermodynamic properties:

Lowest Vibrational Mode (1/cm) =	-221.4147
2nd Lowest Vibrational Mode (1/cm) =	14.5245
E(RM062X) (a.u.) =	-2588.53422864
Thermal correction to Enthalpy (a.u.) =	0.662438
Thermal correction to Gibbs Free Energy (a.u.) =	0.547073
Total Entropy (cal/Kmol) =	242.808
Esp(RM062X+D3) (a.u.) =	-2591.13808216
Esp(D3) (a.u.) =	-0.0108528397
Esp(RM062X) (a.u.) =	-2591.1272293203

Optimised cartesian coordinates (Angstrom):

C	6.904004	-1.682297	-0.275388
C	6.564281	-2.850670	-0.973159
C	5.249534	-3.096079	-1.355162
C	4.283635	-2.148050	-1.016828

C	4.622639	-0.990836	-0.313447
H	7.340141	-3.574973	-1.223997
H	4.979917	-4.000245	-1.903212
C	2.362038	-0.654821	-0.586263
C	1.102438	1.271191	0.289909
C	2.239545	1.327453	1.322984
C	3.579119	1.081452	0.657347
H	4.368250	0.980766	1.412464
S	2.570138	-2.191754	-1.387261
N	3.505562	-0.182811	-0.078874
N	1.194079	0.004129	-0.479485
C	2.224574	2.600472	2.198030
C	3.032722	3.769254	1.628185
C	2.718801	2.263547	3.605171
H	1.171490	2.916077	2.280135
H	4.112593	3.554264	1.664150
H	2.767541	4.009681	0.588831
H	2.859448	4.669412	2.236117
H	2.079730	1.506593	4.083836
H	3.748184	1.871319	3.575148
H	2.721393	3.160585	4.241753
H	2.060059	0.471037	1.990723
H	0.171923	1.201718	0.870919
C	1.015118	2.478569	-0.621611
C	0.144109	3.516445	-0.267758
C	1.804054	2.610579	-1.768684
C	0.093480	4.685690	-1.024492
H	-0.503440	3.397026	0.605477
C	1.750897	3.780467	-2.528270
H	2.465965	1.799820	-2.084148
C	0.904294	4.823606	-2.152603
H	-0.586977	5.489092	-0.736998
H	2.373410	3.874130	-3.419790
H	0.865294	5.737862	-2.747358
C	-1.190044	0.234981	-1.046346
H	-1.210303	1.236136	-0.626080
C	-2.365191	-0.338406	-1.473822

C	-3.483513	0.612315	-1.785840
F	-3.184284	1.357831	-2.862109
F	-4.639532	0.009015	-2.067684
F	-3.709237	1.473895	-0.785143
O	0.133369	-1.600504	-1.706184
C	0.022903	-0.533701	-1.126683
H	3.860180	1.880906	-0.044439
C	5.943269	-0.735391	0.063527
H	6.219531	0.173764	0.597103
H	7.942895	-1.507024	0.006955
H	-2.368911	-1.265210	-2.052926
C	-6.800602	-0.403666	0.271682
H	-7.317263	-1.283139	0.680339
H	-7.207895	0.509230	0.721666
H	-6.944205	-0.381735	-0.817595
C	-4.714093	-1.462333	0.117843
O	-5.421422	-0.425160	0.593873
O	-5.243240	-2.354697	-0.502457
C	-2.468331	-2.582169	0.123991
O	-2.693178	-3.511225	-0.638039
C	-3.273885	-1.375083	0.375816
N	-2.598379	-0.589103	1.390423
C	-1.363141	-1.213638	1.656755
C	-0.337434	-0.850383	2.551686
C	-1.268788	-2.421292	0.959331
C	0.747979	-1.709948	2.683268
H	-0.376701	0.071141	3.127674
C	-0.164805	-3.266364	1.081425
C	0.856345	-2.906140	1.948621
H	1.544145	-1.440130	3.381109
H	-0.127095	-4.185212	0.492737
H	1.735856	-3.540666	2.069244
C	-2.784273	0.739233	1.833134
O	-1.809434	1.446882	1.999889
C	-4.139266	1.247518	2.248874
H	-4.677838	1.660705	1.389158
H	-4.754924	0.460573	2.692796

H -3.949192 2.050991 2.970387

SMDopt/19_TS_SSl

Frequencies, energies and thermodynamic properties:

Lowest Vibrational Mode (1/cm) = -358.0496

2nd Lowest Vibrational Mode (1/cm) = 18.0660

E(RM062X) (a.u.) = -2588.53276707

Thermal correction to Enthalpy (a.u.) = 0.662452

Thermal correction to Gibbs Free Energy (a.u.) = 0.547207

Total Entropy (cal/Kmol) = 242.553

Esp(RM062X+D3) (a.u.) = -2591.13658366

Esp(D3) (a.u.) = -0.0106420523

Esp(RM062X) (a.u.) = -2591.1259416077

Optimised cartesian coordinates (Angstrom):

C	6.746862	-2.201380	-0.015583
C	6.352748	-3.326302	-0.753208
C	5.036677	-3.473014	-1.182461
C	4.127478	-2.467765	-0.856542
C	4.520602	-1.353185	-0.113024
H	7.085251	-4.096425	-0.997791
H	4.725027	-4.345459	-1.758717
C	2.303547	-0.842890	-0.463591
C	1.215827	1.216441	0.282624
C	2.217110	1.126700	1.445382
C	3.589170	0.734758	0.928539
H	4.262052	0.492952	1.761475
S	2.417716	-2.400135	-1.252323
N	3.456585	-0.472573	0.107758
N	1.206376	-0.079167	-0.440338
C	2.240824	2.386190	2.340163
C	3.254341	3.450004	1.911300
C	2.488916	1.982973	3.793965
H	1.232115	2.827878	2.284592
H	4.285386	3.106052	2.091031
H	3.166281	3.724156	0.850176
H	3.106641	4.362623	2.507268
H	1.698045	1.311277	4.158558

H	3.455269	1.463396	3.897164
H	2.513690	2.869045	4.445368
H	1.853497	0.292776	2.067209
H	0.218097	1.313987	0.733227
C	1.438885	2.389071	-0.649436
C	0.688239	3.553241	-0.443775
C	2.396610	2.364136	-1.668494
C	0.923364	4.690219	-1.215576
H	-0.093329	3.552237	0.320671
C	2.628970	3.501672	-2.443232
H	2.969063	1.455699	-1.873594
C	1.901365	4.669387	-2.211492
H	0.335279	5.593622	-1.044060
H	3.381088	3.472784	-3.233642
H	2.086380	5.558333	-2.817118
C	-1.048608	0.409492	-1.221708
H	-0.827725	1.466651	-1.112415
C	-2.381794	0.013847	-1.299595
C	-3.331081	0.962265	-1.972617
F	-3.097825	0.998826	-3.296047
F	-4.609088	0.622372	-1.826509
F	-3.199038	2.221787	-1.534291
O	-0.066339	-1.755771	-1.335967
C	-0.031754	-0.565862	-1.040838
H	4.054018	1.524709	0.319947
C	5.839936	-1.198931	0.315788
H	6.154444	-0.322601	0.882160
H	7.784855	-2.104208	0.305227
H	-2.598762	-1.031791	-1.540031
C	-4.058489	3.280725	1.309796
H	-3.571071	3.739122	0.438200
H	-5.103649	3.603708	1.366830
H	-3.522751	3.587431	2.219127
C	-2.894696	1.270974	1.059529
O	-4.085435	1.868400	1.187239
O	-1.856884	1.894092	1.103742
C	-4.344785	-0.863017	0.587593

O	-5.481109	-0.440228	0.543281
C	-3.044788	-0.154718	0.715487
N	-2.057582	-1.145760	1.059905
C	-2.577301	-2.413565	0.686978
C	-1.961172	-3.658445	0.519889
C	-3.950029	-2.287632	0.433587
C	-2.760623	-4.740561	0.142069
H	-0.894365	-3.812284	0.642285
C	-4.741697	-3.368176	0.062014
C	-4.136813	-4.614936	-0.079218
H	-2.283264	-5.712794	0.005158
H	-5.808036	-3.219638	-0.120155
H	-4.722669	-5.485629	-0.376816
C	-1.050672	-0.963986	2.015093
O	-1.041959	-0.005983	2.754623
C	0.019373	-2.025145	2.087173
H	-0.381875	-2.934659	2.557887
H	0.401911	-2.293273	1.093395
H	0.826087	-1.641463	2.722075

References

1. L. C. Morrill, J. Douglas, T. Lebl, A. M. Z. Slawin, D. J. Fox and A. D. Smith, *Chemical Science*, 2013, **4**, 4146-4155.
2. *Pure and Applied Chemistry*, 1974, **37**, 445-462.
3. W. C. Still, M. Kahn and A. Mitra, *The Journal of Organic Chemistry*, 1978, **43**, 2923-2925.
4. G. R. Fulmer, A. J. M. Miller, N. H. Sherden, H. E. Gottlieb, A. Nudelman, B. M. Stoltz, J. E. Bercaw and K. I. Goldberg, *Organometallics*, 2010, **29**, 2176-2179.
5. H. E. Gottlieb, V. Kotlyar and A. Nudelman, *The Journal of Organic Chemistry*, 1997, **62**, 7512-7515.
6. R. K. Harris, E. D. Becker, S. M. Cabral de Menezes, P. Granger, R. E. Hoffman and K. W. Zilm, *Pure and Applied Chemistry*, 2008, **80**, 59-84.
7. R. K. Harris, E. D. Becker, S. M. Cabral de Menezes, R. Goodfellow and P. Granger, *Pure and Applied Chemistry*, 2001, **73**, 1795-1818.
8. *Pure and Applied Chemistry*, 1976, **45**, 217-220.
9. *Pure and Applied Chemistry*, 1972, **29**, 625-628.
10. R. K. Harris, J. Kowalewski and S. M. Cabral de Menezes, *Pure and Applied Chemistry*, 1997, **69**, 2489-2496.
11. K. K. Murray, R. K. Boyd, M. N. Eberlin, G. J. Langley, L. Li and Y. Naito, *Pure and Applied Chemistry*, 2013, **85**, 1515-1609.

12. J. F. J. Todd, *Pure and Applied Chemistry*, 1991, **63**, 1541-1566.
13. H. A. Favre and W. H. Powell, *Nomenclature of Organic Chemistry: IUPAC Recommendations and Preferred Names 2013*, Royal Society of Chemistry, Cambridge, UK, 2014.
14. M. A. Brimble, D. S. Black, R. Hartshorn, A. P. Rauter, C.-K. Sha and L. K. Sydnes, *Pure and Applied Chemistry*, 2013, **85**, 307-313.
15. W. Mormann and K.-H. Hellwich, *Pure and Applied Chemistry*, 2008, **80**, 201-232.
16. J. Brecher, *Pure and Applied Chemistry*, 2008, **80**, 277-410.
17. J. Brecher, *Pure and Applied Chemistry*, 2006, **78**, 1897-1970.
18. G. P. Moss, *Pure and Applied Chemistry*, 1999, **71**, 531-558.
19. H. A. Favre, K.-H. Hellwich, G. P. Moss, W. H. Powell and J. G. Trynham, *Pure and Applied Chemistry*, 1999, **71**, 1327-1330.
20. G. P. Moss, *Pure and Applied Chemistry*, 1998, **70**, 143-216.
21. G. P. Moss, *Pure and Applied Chemistry*, 1996, **68**, 2193-2222.
22. W. H. Powell, *Pure and Applied Chemistry*, 1993, **65**, 1357-1455.
23. W. Drenth, *Pure and Applied Chemistry*, 1992, **64**, 989-990.
24. G. P. Moss, *Pure and Applied Chemistry*, 1989, **61**, 1783-1822.
25. J. S. Littler, *Pure and Applied Chemistry*, 1989, **61**, 57-81.
26. R. A. Y. Jones and J. F. Bunnett, *Pure and Applied Chemistry*, 1989, **61**, 725-768.
27. R. D. Guthrie, *Pure and Applied Chemistry*, 1989, **61**, 23-56.
28. W. H. Powell, *Pure and Applied Chemistry*, 1988, **60**, 1395-1401.
29. J. F. Bunnett and R. A. Y. Jones, *Pure and Applied Chemistry*, 1988, **60**, 1115-1116.
30. G. P. Moss, *Pure and Applied Chemistry*, 1987, **59**, 779-832.
31. N. Lozac'h, *Pure and Applied Chemistry*, 1986, **58**, 1693-1696.
32. W. H. Powell, *Pure and Applied Chemistry*, 1984, **56**, 769-778.
33. *Pure and Applied Chemistry*, 1984, **56**, 595-624.
34. W. H. Powell, *Pure and Applied Chemistry*, 1983, **55**, 409-416.
35. J. E. Merritt and K. L. Loening, *Pure and Applied Chemistry*, 1983, **51**, 2251-2304.
36. N. Lozac'h, *Pure and Applied Chemistry*, 1983, **55**, 1463-1466.
37. W. H. Powell, *Pure and Applied Chemistry*, 1982, **54**, 217-228.
38. *Pure and Applied Chemistry*, 1982, **54**, 211-216.
39. *Pure and Applied Chemistry*, 1982, **54**, 207-210.
40. J. F. Bunnett, *Pure and Applied Chemistry*, 1981, **53**, 305-322.
41. *Pure and Applied Chemistry*, 1981, **53**, 1901-1905.
42. *Pure and Applied Chemistry*, 1979, **51**, 1995-2003.
43. *Pure and Applied Chemistry*, 1979, **51**, 353-380.
44. L. C. Cross and W. Klyne, *Pure and Applied Chemistry*, 1976, **45**, 11-30.
45. *Pure and Applied Chemistry*, 1974, **40**, 315-332.
46. *Pure and Applied Chemistry*, 1972, **31**, 283-322.
47. *Pure and Applied Chemistry*, 1972, **31**, 639-646.
48. *Pure and Applied Chemistry*, 1971, **28**, 1-110.
49. *Pure and Applied Chemistry*, 1965, **11**, 1-260.
50. H. Liu, A. M. Z. Slawin and A. D. Smith, *Organic Letters*, 2020, **22**, 1301-1305.
51. J. Bitai, A. J. Nimmo, A. M. Z. Slawin and A. D. Smith, *Angewandte Chemie International Edition*, 2022, **61**, e202202621.
52. J. E. Lapetaje, C. M. Young, C. Shu and A. D. Smith, *Chemical Communications*, 2022, **58**, 6886-6889.
53. H.-L. Liu, X.-T. Li, H.-Z. Tian and X.-W. Sun, *Organic Letters*, 2021, **23**, 4579-4583.
54. Y.-N. Duan, L.-Q. Cui, L.-H. Zuo and C. Zhang, *Chemistry – A European Journal*, 2015, **21**, 13052-13057.
55. L. Zhao, G. Huang, B. Guo, L. Xu, J. Chen, W. Cao, G. Zhao and X. Wu, *Organic Letters*, 2014, **16**, 5584-5587.

56. *CrystalClear-SM Expert* v2.1., Rigaku Americas, *The Woodlands*, *Texas*, *USA*, and Rigaku Corporation, *Tokyo*, *Japan*, 2015.
57. *CrysAlisPro* v1.171.42.49, v1.171.42.82a, v1.171.42.93a, Rigaku Oxford Diffraction, Rigaku Corporation, *Oxford*, *U.K.*, 2020-2023.
58. M. C. Burla, R. Caliandro, M. Camalli, B. Carrozzini, G. L. Cascarano, C. Giacovazzo, M. Mallamo, A. Mazzone, G. Polidori and R. Spagna, *Journal of Applied Crystallography*, 2012, **45**, 357-361.
59. G. Sheldrick, *Acta Crystallographica Section A*, 2015, **71**, 3-8.
60. G. Sheldrick, *Acta Crystallographica Section C*, 2015, **71**, 3-8.
61. O. V. Dolomanov, L. J. Bourhis, R. J. Gildea, J. A. K. Howard and H. Puschmann, *Journal of Applied Crystallography*, 2009, **42**, 339-341.
62. J. Wu, C. M. Young, A. A. Watts, A. M. Z. Slawin, G. R. Boyce, M. Bühl and A. D. Smith, *Organic Letters*, 2022, **24**, 4040-4045.
63. C. Wang, S.-J. Li, Q.-C. Zhang, D. Wei and L. Ding, *Catalysis Science & Technology*, 2020, **10**, 3664-3669.
64. Y. Zhao and D. G. Truhlar, *Theoretical Chemistry Accounts*, 2008, **120**, 215-241.
65. A. Schäfer, H. Horn and R. Ahlrichs, *The Journal of Chemical Physics*, 1992, **97**, 2571-2577.
66. A. Schäfer, C. Huber and R. Ahlrichs, *The Journal of Chemical Physics*, 1994, **100**, 5829-5835.
67. F. Weigend and R. Ahlrichs, *Physical Chemistry Chemical Physics*, 2005, **7**, 3297-3305.
68. F. Weigend, *Physical Chemistry Chemical Physics*, 2006, **8**, 1057-1065.
69. A. V. Marenich, C. J. Cramer and D. G. Truhlar, *The Journal of Physical Chemistry B*, 2009, **113**, 6378-6396.
70. B. Mennucci and J. Tomasi, *The Journal of Chemical Physics*, 1997, **106**, 5151-5158.
71. J. Tomasi, B. Mennucci and E. Cancès, *Journal of Molecular Structure: THEOCHEM*, 1999, **464**, 211-226.
72. M. J. Frisch, G. W. Trucks, H. B. Schlegel, G. E. Scuseria, M. A. Robb, J. R. Cheeseman, G. Scalmani, V. Barone, G. A. Petersson, H. Nakatsuji, X. Li, M. Caricato, A. V. Marenich, J. Bloino, B. G. Janesko, R. Gomperts, B. Mennucci, H. P. Hratchian, J. V. Ortiz, A. F. Izmaylov, J. L. Sonnenberg, Williams, F. Ding, F. Lipparini, F. Egidi, J. Goings, B. Peng, A. Petrone, T. Henderson, D. Ranasinghe, V. G. Zakrzewski, J. Gao, N. Rega, G. Zheng, W. Liang, M. Hada, M. Ehara, K. Toyota, R. Fukuda, J. Hasegawa, M. Ishida, T. Nakajima, Y. Honda, O. Kitao, H. Nakai, T. Vreven, K. Throssell, J. A. Montgomery Jr., J. E. Peralta, F. Ogliaro, M. J. Bearpark, J. J. Heyd, E. N. Brothers, K. N. Kudin, V. N. Staroverov, T. A. Keith, R. Kobayashi, J. Normand, K. Raghavachari, A. P. Rendell, J. C. Burant, S. S. Iyengar, J. Tomasi, M. Cossi, J. M. Millam, M. Klene, C. Adamo, R. Cammi, J. W. Ochterski, R. L. Martin, K. Morokuma, O. Farkas, J. B. Foresman and D. J. Fox, *Gaussian 16 Rev. C.01* Wallingford, CT, 2016.
73. C. Y. Legault, *CYLview20* Build 0001, Sherbrooke, Canada, 2020.
74. R. A. Boto, F. Peccati, R. Laplaza, C. Quan, A. Carbone, J.-P. Piquemal, Y. Maday and J. Contreras-García, *Journal of Chemical Theory and Computation*, 2020, **16**, 4150-4158.
75. W. Humphrey, A. Dalke and K. Schulten, *Journal of Molecular Graphics*, 1996, **14**, 33-38.
76. M. C. Rezende and M. Domínguez, *Journal of Molecular Modeling*, 2015, **21**, 212.
